# Deep Sequencing Analysis of Virome Components, Viral Gene Expression and Antiviral RNAi Responses in *Myzus persicae* Aphids

**DOI:** 10.3390/ijms252313199

**Published:** 2024-12-08

**Authors:** Natalia Sukhikh, Victor Golyaev, Nathalie Laboureau, Gabriel Clavijo, Camille Rustenholz, Aurelie Marmonier, Quentin Chesnais, Mylène Ogliastro, Martin Drucker, Veronique Brault, Mikhail M. Pooggin

**Affiliations:** 1 PHIM Plant Health Institute, University of Montpellier, INRAE, CIRAD, IRD, Institute Agro, 34398 Montpellier, France; sukhikh.natalie@gmail.com (N.S.); victor.golyaev@gmail.com (V.G.); nathalie.laboureau@cirad.fr (N.L.); 2 SVQV, INRAE, Université de Strasbourg, 68000 Colmar, France; gaclavijo@hotmail.com (G.C.); camille.rustenholz@inrae.fr (C.R.); aurelie.marmonier@inrae.fr (A.M.); quentin.chesnais@inrae.fr (Q.C.); martin.drucker@inrae.fr (M.D.); veronique.brault@inrae.fr (V.B.); 3 DGIMI, INRAE, Université de Montpellier, 34095 Montpellier, France; marie-helene.ogliastro@inrae.fr

**Keywords:** *Myzus persicae*, aphid, densovirus, flavivirus, polerovirus, RNAi, small RNA, siRNA, piRNA

## Abstract

The green peach aphid (*Myzus persicae*) is a generalist pest damaging crops and transmitting viral pathogens. Using Illumina sequencing of small (s)RNAs and poly(A)-enriched long RNAs, we analyzed aphid virome components, viral gene expression and antiviral RNA interference (RNAi) responses. Myzus persicae densovirus (family *Parvoviridae*), a single-stranded (ss)DNA virus persisting in the aphid population, produced 22 nucleotide sRNAs from both strands of the entire genome, including 5′- and 3′-inverted terminal repeats. These sRNAs likely represent Dicer-dependent small interfering (si)RNAs, whose double-stranded RNA precursors are produced by readthrough transcription beyond poly(A) signals of the converging leftward and rightward transcription units, mapped here with Illumina reads. Additionally, the densovirus produced 26–28 nucleotide sRNAs, comprising those enriched in 5′-terminal uridine and mostly derived from readthrough transcripts and those enriched in adenosine at position 10 from their 5′-end and mostly derived from viral mRNAs. These sRNAs likely represent PIWI-interacting RNAs generated by a ping-pong mechanism. A novel ssRNA virus, reconstructed from sRNAs and classified into the family *Flaviviridae*, co-persisted with the densovirus and produced 22 nucleotide siRNAs from the entire genome. Aphids fed on plants versus artificial diets exhibited distinct RNAi responses affecting densovirus transcription and flavivirus subgenomic RNA production. In aphids vectoring turnip yellows virus (family *Solemoviridae*), a complete virus genome was reconstituted from 21, 22 and 24 nucleotide viral siRNAs likely acquired with plant phloem sap. Collectively, deep-sequencing analysis allowed for the identification and de novo reconstruction of *M. persicae* virome components and uncovered RNAi mechanisms regulating viral gene expression and replication.

## 1. Introduction

The green peach aphid *Myzus persicae* Sulzer (Hemiptera: family Aphididae) is a globally distributed crop pest. It has a wide host range and causes direct and indirect damage to host plants through feeding and the transmission of viral pathogens, respectively [1]. Although *M. persicae* is an efficient vector of more than a hundred plant viruses, only one insect virus was identified in this aphid species so far. Some clonal populations of *M. persicae* were indeed found to be persistently infected with Myzus persicae densovirus (MpDV), a single-stranded DNA (ssDNA) virus of the genus *Hemiambidensovirus* (family *Parvoviridae*), which can be transmitted vertically (transovarially) and horizontally via saliva and honeydew [2,3,4]. A distinct variant of MpDV was also identified in the tobacco aphid *M. persicae nicotianae*, a subspecies of *M. persicae* [5]. Interestingly, endogenous virus elements (EVEs) with partial low homology to MpDV and other densoviruses were identified in the *M. persicae* genome [6]. This indicates long-standing interactions and co-evolution of densoviruses with *M. persicae,* which have resulted in viral integrations in the aphid genome and eventually led to persistent (rather than acute) infections with episomal densoviruses. Indeed, EVEs are implicated in the sequence-specific defense against cognate episomal viruses, which is mediated by RNA interference (RNAi) [7].

RNAi is an evolutionarily conserved mechanism that regulates gene expression and defends against invasive nucleic acids such as transposons, transgenes, EVEs, viruses and virus-like agents. In most eukaryotes, including animals, fungi and plants, RNAi is mediated by Dicer or Dicer-like family proteins that generate 21–24-nucleotide (nt) small RNA (sRNA) duplexes from various double-stranded RNA (dsRNA) precursors and Argonaute (AGO) family proteins that bind guide strands of the sRNA duplexes and form RNA-induced silencing complexes (RISCs). RISCs guided by sRNAs can target complementary mRNAs or viral RNA for cleavage or translational repression and can also target cognate chromosomal DNA or episomal viral DNA for transcriptional silencing. Dicer- and AGO-dependent sRNAs are classified into miRNAs, expressed from MIR genes to regulate expression of other genes in trans, and diverse small interfering RNAs (siRNAs), produced either from miRNA-target genes to repress other genes in trans or from transposons, transgenes, EVEs and episomal viruses to repress their expression and proliferation [8,9,10,11]. In addition, animals produce 25–30 nt PIWI-associated RNAs (piRNAs) of endogenous and viral origin by a Dicer-independent, ping-pong mechanism. This mechanism involves the 5′-3′ endonuclease Zucchini that fragments endogenous or viral transcripts and thereby generates piRNA precursors and two distinct types of the PIWI subfamily of Argonaute proteins, which are associated with mature piRNAs possessing either 5′-terminal uridine (1U) or adenosine at position 10 from the 5′-end (10A), respectively. Primary 1U-piRNAs are produced from the transcripts fragmented by Zucchini, while 10A-piRNAs are produced from the complementary transcripts targeted by 1U-piRNA-PIWI complexes. In completing the ping-pong cycle, 10A-piRNA-PIWI complexes target the sense transcripts back, thereby generating precursors of secondary 1U-piRNAs [12,13].

The siRNA- and piRNA-generating pathways have been most extensively studied in model insects such as the fruit fly *Drosophila melanogaster* (Diptera), the mosquitoes *Aedes aegypti* and *Ae. albopictus* (Diptera) and the silkworm *Bombyx mori* (Lepidoptera). While both siRNA- and piRNA-generating machineries are involved in antiviral defenses in mosquitoes and *B. mori*, the piRNA machinery of Drosophila appears to play a minor role in defenses against episomal viruses and is mostly involved in the control of transposons and endogenous retroviruses [9,12,13,14].

Nothing is known about the RNAi machinery of *M. persicae*, except that exogenous RNAi can knock down the expression of the RNAi-targeted aphid genes [15,16,17,18,19,20]. Although MpDV-derived sRNAs of a broad size range were identified in *M. persicae* aphids, their sequence features were not thoroughly analyzed [21]. Genomes of the pea aphid *Acyrthosiphon pisum* and the Russian wheat aphid *Diuraphis noxia* encode the key protein components of miRNA-, siRNA- and piRNA-generating machineries [9], but no comprehensive analysis of endogenous or viral sRNAs has so far been reported for these or other aphids. In other hemipteran insects, such as whiteflies, planthoppers, leafhoppers and psyllids, siRNA- and piRNA-generating pathways are involved in the control of transposons and EVEs, as well as in defense against episomal viruses [13,22]. In the Asian citrus psyllid *Diaphorina citri*, an episomal ssDNA ambisense densovirus produced 21 nt siRNAs and less abundant 28–30 nt 1U- and 10A-piRNAs of both polarities, whereas the densoviral EVEs produced mostly 1U-piRNAs of a broader size range and one polarity [23]. By contrast, a single-stranded RNA (ssRNA) flavivirus produced mostly 21 nt siRNAs but no piRNAs [23].

Unlike insects and other animals, plants do not possess the Zucchini or PIWI subfamily homologs required for piRNA biogenesis, and their antiviral RNAi pathways are mediated by virus-derived siRNAs of three major size classes (21, 22 and 24 nt). These siRNAs are generated from dsRNA precursors by four types of Dicer-like proteins and then associated with distinct types of AGO family proteins that sort sRNAs based on 5′-nucleotide identity and size [11,24,25,26]. On the other hand, plants (but not insects or other animals) possess RNA-directed RNA polymerase (RDR) family proteins that reinforce RNAi by converting aberrant RNAs to dsRNA precursors of secondary siRNAs [10,11,26].

In this study, we used Illumina sequencing analysis of sRNAs and poly(A)-enriched long RNAs from *M. persicae* aphids persistently infected with the ssDNA densovirus MpDV and those viruliferous for a circulative ssRNA polerovirus acquired from plants or artificial diets in order to characterize aphid RNAi responses to persistent densovirus infection and circulation of the plant virus. We uncovered the aphid siRNA- and piRNA-generating pathways regulating MpDV gene expression in response to aphid feeding conditions and found no evidence for the replication of the plant virus, which would have induced aphid RNAi. We also discovered a novel ssRNA insect virus co-persisting with MpDV in the aphid population and producing virus-derived siRNAs but no piRNAs.

## 2. Results and Discussion

### 2.1. Myzus Persicae Densovirus (MpDV) Genome, Transcriptome and sRNAome

#### 2.1.1. MpDV Genome

**Figure 1:**
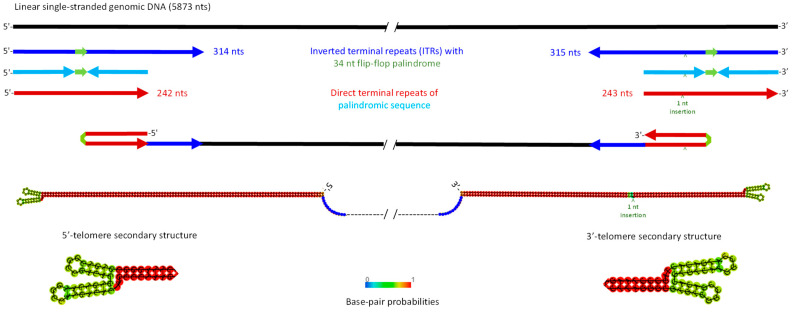
Structures of Myzus persicae densovirus (MpDV) 5′- and 3′-inverted terminal repeats (ITRs) forming symmetrical Y-shaped hairpin telomeres. The 5873 nt linear single-stranded genomic DNA encapsidated in MpDV virions is depicted as a solid black line. Imperfect inverted repeats of the 314 nt 5′-ITR and the 315 nt 3′-ITR sequences that differ by an inversion of a 34 nt flip-flop palindrome and a 1 nt insertion are depicted as blue arrows. Small green arrows indicate the positions of the flip-flop palindrome within each ITR. Near-perfect 242 and 243 nt direct terminal repeats of the palindromic sequences that differ by the 1 nt insertion in the 3′-repeat are depicted as red arrows with the inserted nucleotide position indicated and also as converging cyan arrows connected by small green arrows representing the flip-flop palindrome. Secondary structures of the 5′ and 3′-telomers, predicted using Matthews model’s DNA parameters at 30 °C at Webserver http://rna.tbi.univie.ac.at/cgi-bin/RNAWebSuite/RNAfold.cgi (accessed on 1 October 2024), are depicted both schematically and as images exported from the Webserver. Symmetrical Y-shaped tops of the 5′- and 3′-telomers are enlarged. Color code indicates base pair probabilities ranging from 0 (blue) to 1 (red).

The French strain of MpDV (MpDV1) was identified in a clonal population of *M. persicae* maintained at INRAE-Colmar [2] and its genome sequence of 5499 nts with partial 5′- and 3′-inverted terminal repeats (ITRs) was determined by direct sequencing with primer walking on purified virion DNA [3]. To determine the complete genome sequence of MpDV1, we performed Illumina sequencing of virion DNA purified from the same aphid colony (Mp-Col), followed by de novo assembly of paired-end reads (Figure S1a). MpDV1 was found to possess 5′-ITR of 314 nts and 3′-ITR of 315 nts. These ITRs contain 242 nt and 243 nt direct terminal repeats of the palindromic sequences, differing by a 1 nt insertion within the 3′-repeat and forming symmetrical Y-shaped hairpin telomeres (Figure 1; File S1a,b), similar to those of homotelomeric ambisense densoviruses ([27,28]; https://ictv.global/report/chapter/parvoviridae/parvoviridae (accessed on 1 October 2024)). This reconstruction was consistent with our restriction analysis of virion DNA. Since genomic and antigenomic viral DNA molecules of homotelomeric ambisense densoviruses are encapsidated in separate virions in equal proportions, the purified virion DNA of MpDV forms linear dsDNA migrating in 1% agarose as a single band of ca. 5–6 Kbp [3]. Digestion of the virion DNA with PauI and SpeI enzymes, whose unique restriction sites are located downstream of the 5′-ITR and upstream of the 3′-ITR, respectively, yielded major digestion products of expected sizes (Figure S1b). In support of our finding of 314 and 315 nt long ITRs in MpDV, other ambisense densoviruses have ITRs ranging in size from 122 to 550 nts [29].

Besides having reconstructed complete ITRs, we found approximately 1% divergence between the genome sequences of the previous isolate of MpDV1 (NCBI GenBank accession NC_005040.1) and its new isolate with the complete genome of 5873 nts (deposited in GenBank under PQ247848), which included 40 single-nucleotide polymorphisms (SNPs) and 8 indels (insertions or deletions) of 1 to 4 nts in both non-coding and coding sequences. This divergence indicates an on-going evolution of the virus quasispecies in the aphid population. The Chinese strain of MpDV (MpDV2) identified in *M. persicae* aphids [4] (OQ201577.1) has a smaller genome of 5727 nts due to shorter 5′- and 3′-ITRs, both lacking 78 nts at the respective termini. The rest of MpDV2 sequence differs by 120 SNPs and 9 indels of 1 to 22 nts (File S1c) and shares 97.9% overall identity with MpDV1. Like MpDV1, MpDV2 has symmetrical Y-shaped hairpin telomeres, although two small stem-loops at hairpins’ tops in MpDV2 and MpDV1 differ in sizes and stabilities due to differences in the size of a flip-flop palindrome (33 vs. 34 nts) and SNPs in surrounding nucleotides (File S1a,b). The four main ORFs in MpDV1 and MpDV2 have the same lengths and encode proteins sharing 97.4 to 98.0% amino acid identity. It should be noted that Myzus persicae nicotianae densovirus (MpnDV) isolated from *M. persicae nicotianae* aphids in China [5] is a genetic variant of MpDV with incomplete ITRs of 112 nts. MpnDV has a higher homology to MpDV2 in each of the four main ORFs’ encoded proteins (98.3 to 99.0% amino acid identity), as well as in the shared non-coding sequences (File S1c).

#### 2.1.2. MpDV Transcriptome and Gene Expression

**Figure 2:**
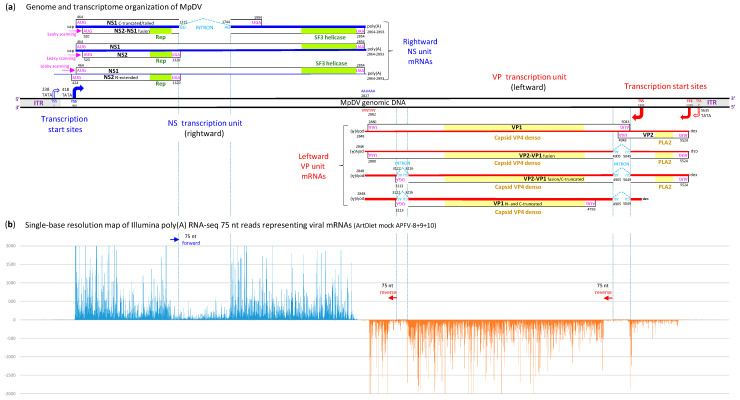
Genome organization and transcriptome of Myzus persicae densovirus (MpDV) revealed by de novo assembly and quantitative analysis of Illumina mRNA-seq reads from *M. persicae* aphids. (**a**) Genome and transcriptome organization of MpDV. Viral genomic (forward and reverse) ssDNA molecules encapsidated in virions are depicted as black lines, with the inverted terminal repeats (ITRs) shown as gray boxes. Major and minor transcription start sites (TSSs) of the rightward non-structural (NS) and leftward virion protein (VP) genes are shown with blue and red bent arrows, respectively. Spliced and unspliced mRNAs transcribed from the NS and VP units are depicted with blue and red lines, respectively, with positions of 5′-cap and poly(A) sites, ORFs’ start and stop codons and 5′- and 3′-splice sites of introns indicated. The encoded proteins are named and their domains are colored and named. Pink arrows indicate ribosome leaky scanning that allows for the translation of overlapping ORFs from NS mRNAs. The thickness of the blue and red lines is roughly proportional to the relative abundance of viral mRNAs estimated by the quantitation of Illumina reads mapped to exons vs introns. (**b**) Single-base resolution map of Illumina 75 nt reads representing viral mRNAs. The reads were mapped to MpDV genome, and the mapping data were analyzed using MISIS-2 [30] and visualized using Excel (Dataset S2a). The map of combined reads from 3 biological replicates under one of the 4 feeding conditions (ArtDiet mock APFV-8+9+10; Dataset S1) is presented as a histogram that plots the numbers of 75 nt reads at each nucleotide position of the 5873 nt MpDV genome; blue bars above the axis represent forward reads starting at each respective position, while red bars below the axis represent reverse reads ending at the respective position. Vertical dotted lines indicate positions of the splice sites of NS and VP introns. Blue and red arrows indicate the orientation and length of the forward and reverse reads, respectively. Bars exceeding the value of 2000 unique reads are cut off. Complete histograms for all conditions are in Dataset S2a.

To analyze MpDV gene expression, we performed Illumina sequencing of a poly(A)-enriched fraction of aphid RNA, followed by de novo assembly of viral mRNAs using Trinity (Figure S2) and quantitative analysis of redundant reads mapped on the viral genome (Datasets S1 and S2a). The results revealed converging Pol II transcription units spanning the non-structural (NS) protein genes in the rightward direction and the virion protein (VP) genes in the leftward direction (Figure 2), which is typical for ambisense densoviruses ([27,28]; https://ictv.global/report/chapter/parvoviridae/parvoviridae (accessed on 1 October 2024)).

In the NS (rightward) transcription unit, a major transcription start site was identified at position 460 as a 5′-border of highly abundant reads covering the unit, with one lower-coverage gap corresponding to an intron (see below) (Figure 2b). This start site is located downstream of a canonical TATA-box TATATAA (positions 418–424) and 8 nts upstream of the NS1 ORF start codon. On the other hand, the 5′-ends of Trinity contigs were mapped to several positions from 269 to 428 (File S1d). These minor transcription events supported by low-abundance reads (Figure S3; Dataset S2a) could be driven by another canonical TATA-box located within the 5′-ITR at positions 238–244 (TATATAA). In MpDV2, 5′-RACE analysis of NS transcripts revealed two start sites, 7 nts upstream of the 5′-ITR TATA-box and 29 nts downstream of the second TATA-box [4]. The latter is in close vicinity of the main start site in MpDV1. In MpnDV, a single NS transcription start site was identified 29 nts downstream of the second TATA-box [5]. Analysis of the Trinity contigs with poly(A) tails revealed the NS polyadenylation sites at positions 2864, 2866, 2870, 2876, 2880 and 2888, all downstream of a canonical poly(A) signal AAUAAA (2827–2832) and NS1 ORF stop codon (2852–2854) (File S1d). These sites are in close vicinity to a single poly(A) site identified in MpnDV [5] and one of the two poly(A) sites in MpDV2 [4].

Further analysis of Trinity contigs (Figure S2; File S1d) revealed that the NS unit contains a 430 nt intron (positions 1351–1744) with canonical 5′-GU and 3′-AG termini. Based on a quantitative analysis of mapped reads, this intron is spliced out in a major fraction of viral mRNAs (Figure 2b; Dataset S2a). An intron of the same length and at the same position was identified in MpDV2 [4], whereas no intron was found in the NS unit of MpnDV [5]. Inspection of MpDV1 sequence revealed that the unspliced NS mRNA can potentially be translated into NS1 protein of 796 amino acids and, via ribosome leaky scanning, into NS2 protein of 269 amino acids, as no AUG occurs between the AUG start codons of the NS1 and NS2 ORFs. A minor mRNA, potentially transcribed from the 5′-ITR-based promoter, can be translated into an N-terminally extended version of NS2 protein from an upstream in-frame AUG (424–426) and, via leaky scanning, into NS1 protein from the downstream AUG (464–466) (Figure 2a). Because the 5′-splice site is located 14 nts upstream of the NS2 ORF stop codon and the intron removal fuses the NS2 and NS1 translation frames, the major spliced mRNA can be translated into a C-terminally truncated NS1 protein and, via leaky scanning, into a NS2-NS1 fusion protein of 666 amino acids (Figure 2a). In all the variants of MpDV (MpDN1, MpDN2 and MpnDV), the NS2 ORF-encoded protein contains replication initiator (Rep) motifs (amino acids 214–276) conserved in the subfamily *Densovirinae* [4,5], while a C-terminal portion of the NS1 protein contains a superfamily 3 (SF3) helicase domain (amino acids 622–774; identified using InterPro: https://www.ebi.ac.uk/interpro/ (accessed on 1 October 2024); [31]). Thus, the NS2-NS1 fusion proteins translated from the major and minor spliced RNAs possess both functional domains of the replication initiator protein that can mediate the rolling-hairpin replication of MpDV. Viral replication may also be regulated by a C-terminally truncated NS1 protein lacking the helicase domain, which is translated from the spliced mRNAs, and complete NS1 and NS2 proteins translated from the unspliced mRNAs (Figure 2a).

In the VP (leftward) transcription unit, one start site was identified at position 5140, 97 nts upstream of the VP1 ORF AUG start codon, as a 5′-border of highly abundant reverse reads spanning VP1 ORF (with two low-coverage gaps corresponding to introns; see below). Another start site was identified at position 5531, 7 nts upstream of the VP2 ORF AUG start codon, as a 5′-border of less abundant reverse reads spanning VP2 ORF (Figure 2b; Dataset S2a). Both start sites are far away from the only canonical TATA-box TATATAA (5635–5629) located within the 3′-ITR. In MpnDV, the 5′-RACE has revealed a single start site 13 nt upstream of the VP2 ORF start codon [5], which is in close vicinity of the upstream start site in MpDV1. In MpDV2, one start site is located 7 nt upstream of the VP1 ORF, that is at the same position as the downstream site in MpDV1, while another one is in the 3′-ITR, 15 nts upstream of the TATA-box [4]. Our Trinity contigs also provide evidence for minor transcription initiation events within the 3′-ITR (File S1d), which are supported by very-low-abundance reads (Figure S3; Dataset S2a). It remains to be investigated whether transcription initiation events at the two main start sites of the MpDV1 VP unit, supported by higher-abundance reads, are driven by non-canonical core promoters. Analysis of the Trinity contigs with poly(A) tails (File S1d) revealed a single VP polyadenylation site at position 2848, downstream of the canonical poly(A) signal AAUAAA (2882–2877) and the VP1 ORF stop codon (2880–2878). A single poly(A) site at the same position was also found in MpDV2 [4], while six poly(A) sites located 12 to 23 nts downstream of the poly(A) signal were identified in MpnDV [5].

Further analysis of Trinity contigs (Figure S2; File S1d) revealed two short introns of 145 nts (5049–4905) and 95 nts (3122–3216), both having the canonical 5′-GU and 3′-AG termini. Based on quantitative analysis of mapped reads, these introns are spliced out in major but unequal fractions of viral transcripts, with the first/upstream intron removed much more efficiently (Figure 2b; Dataset S2a). This suggests the existence of two main spliced mRNAs lacking both introns, one transcribed from the upstream start site and another one from the downstream start site, as well as minor mRNAs in which the first intron or both introns are retained (Figure 2a). Introns of the same lengths and at the same positions were identified in both MpDV2 [4] and MpnDV [5]. Notably, the first intron spans the overlapping sequences of VP2 and VP1 ORFs and its removal fuses the VP2 and VP1 translational frames, while the splicing of the second intron at the 3′-end of VP1 ORF shifts the translation frame, leading to a premature termination codon (Figure 2a). Thus, in the case of transcription initiation at the upstream site, the main spliced mRNAs lacking the first or both introns can be translated into a VP2-VP1 fusion protein of 833 amino acids or its C-terminally truncated version of 723 amino acids, respectively. In contrast, the minor unspliced VP2-VP1 mRNA can be translated only into the VP2 protein of 193 amino acids, as several out-of-frame AUGs between the VP2 and VP1 start codons would prevent leaky scanning. In the case of transcription initiation at the downstream site, the main spliced mRNAs lacking the first or both introns can be translated into an N-terminally truncated VP1 protein of 625 amino acids or its C-terminally truncated version of 515 amino acids (Figure 2a). Finally, a complete VP1 ORF can be translated from a minor unspliced mRNA transcribed from the downstream start site. In all the variants of MpDV, the VP2 ORF-encoded protein contains conserved densoviral Phospholipase A2 (PLA2) motifs (amino acids 40–90; identified by Guo et al. [4]), while the VP1 ORF-encoded protein contains a Capsid VP4 densovirus domain (amino acids 125–514; identified using InterPro). Thus, all the VP2-VP1 fusion proteins possess both functional domains of the structural proteins, whereas all the versions of VP1 possess only the capsid domain (Figure 2a). We assume that the truncated VP1 proteins expressed from the more abundant spliced mRNAs transcribed from the downstream promoter and the VP2-V1 fusion proteins expressed from the less abundant spliced mRNAs transcribed from the upstream promoter will be assembled into viral particles in, respectively, higher and lower proportions. The PLA2 domain of less abundant VP2-VP1 fusion proteins will be exposed outside of the particles to enable clathrin-mediated endocytosis, followed by trafficking to the nucleus, as shown for an insect ambisense densovirus [32].

#### 2.1.3. MpDV sRNAome and Antiviral RNAi

**Figure 3:**
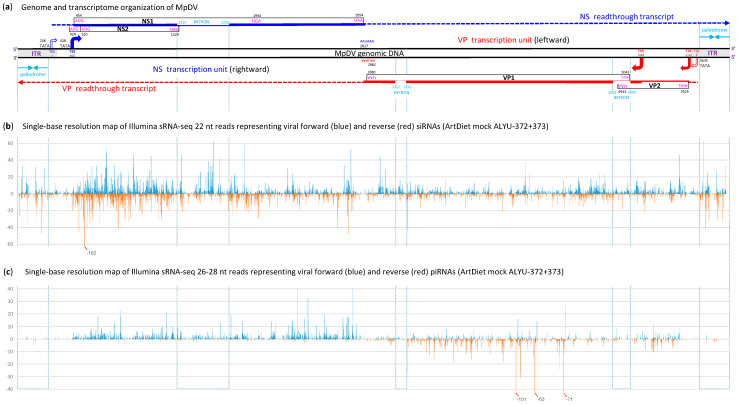
Small RNAome of Myzus persicae densovirus (MpDV) revealed by Illumina sRNA-seq analysis of *M. persicae* aphids. (**a**) Genome and transcriptome organization of MpDV. Viral genomic (forward and reverse) ssDNA molecules are depicted as black lines, with positions of the inverted terminal repeats (ITRs) shown as gray boxes. Major and minor transcription start sites (TSSs) of the rightward non-structural (NS) and leftward virion protein (VP) genes are shown with blue and red bent arrows, respectively. Viral mRNAs transcribed from the NS and VP units are depicted with blue and red solid lines, respectively, with positions of ORFs and introns indicated. Dashed lines depict the rightward and leftward readthrough transcripts and the transcripts initiated within ITRs. (**b**,**c**) Single-base resolution map of Illumina 22 nt reads (**b**) and combined 26–28 nt reads (**c**) representing viral siRNAs and piRNAs, respectively. The reads were mapped to the MpDV genome, and the mapping data were analyzed using MISIS-2 [30] and visualized using Excel (Dataset S4a). The maps of combined reads from 2 biological replicates under one of the 4 feeding conditions (ArtDiet mock ALYU-372+373; Dataset S3) are presented as histograms that plot the numbers of 22 nt (**b**) and 26–28 nt (**c**) reads at each nucleotide position of the 5873 nt MpDV genome: blue bars above the axis represent forward reads starting at each respective position, while red bars below the axis represent reverse reads ending at the respective position. Vertical dotted lines indicate positions of the 5′- and 3′-palindromes and the 5′- and 3′-splice sites of NS and VP introns. The red bars exceeding the values of 60 (**b**) or 40 (**c**) unique reads were cut off and the reads’ numbers indicated. Complete histograms for each of the 4 conditions are shown in Dataset S4a.

**Figure 4:**
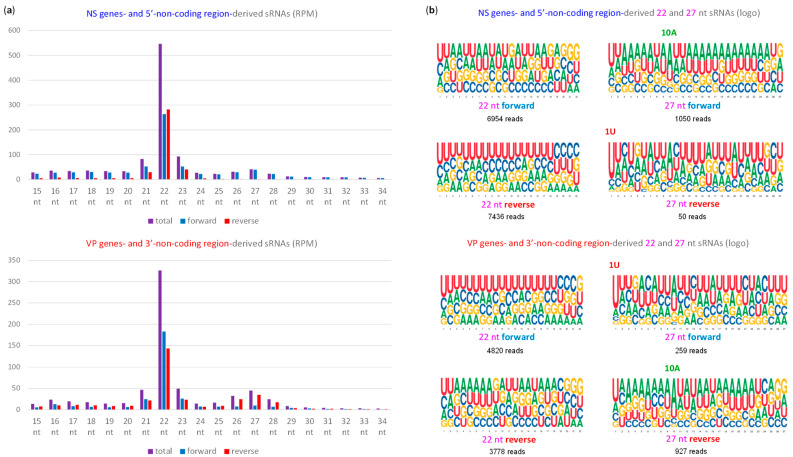
Size profiles and nucleotide compositions of Myzus persicae densovirus (MpDV)-derived sRNAs. Illumina sRNA-seq 15–34 nt reads from *M. persicae* aphids fed on plants or artificial diets were mapped with zero mismatches to the reference sequences of NS and VP parts of the 5873 nt MpDV genome (positions 1–2893 and 2847–5873, respectively) and the mapped reads were sorted by size and polarity (forward, reverse, total) and counted in reads per million (RPM) of total (host + viral) 15–34 nt reads (Dataset S3). (**a**) Counting results for combined reads from 2 biological replicates under one of the 4 feeding conditions (ArtDiet mock ALYU-372+373) are shown as bar graphs representing counts of each size class of sRNAs derived from the NS (upper graph) and the VP (lower graph) parts of MpDV genome. Counting results for the other 3 conditions are shown in Figure S4a. (**b**) Nucleotide compositions of 22 nt and 27 nt forward and reverse sRNAs derived from the NS and VP parts of MpDV genome under the ArtDiet mock condition are presented as RNA logos with the numbers of reads indicated. Positions 1 and 10 of 27 nt reads, enriched in uridine (1U) and adenosine (10A), respectively, are indicated. Nucleotide compositions of 21–23 nt and 26–28 nt viral sRNAs under all feeding conditions are shown in Figure S6.

To investigate the antiviral RNAi responses induced by MpDV in *M. persicae*, we performed Illumina sequencing of aphid sRNAs, followed by bioinformatics analysis of the size, nucleotide composition and genome coverage profiles of MpDV-derived sRNAs (Datasets S3 and S4a). Mapping of 15–34 nt reads to the MpDV genome with zero mismatches revealed that both strands of the entire virus genome are covered with abundant sRNAs that belong predominantly to the 22 nt class, followed by less abundant 21 and 23 nt classes (Figure 3b, Figure 4a, Figures S4a and S5; Datasets S3 and S4a). In both strands of both NS and VP units, the 5′-terminal position of 22 nt sRNAs is dominated by uridine, followed by adenosine and cytosine (Figure 4b and Figure S6). These findings together with the mechanistic knowledge obtained for other insects [9,12,13] suggest that MpDV-derived 22 nt sRNAs are bona fide siRNAs processed by the aphid homolog of Dicer-2 from dsRNA precursors in a form of duplexes. The duplexes are then sorted by the aphid AGO proteins that bind one of the duplex strands (guide strand) based on the 5′-nucleotide identity, while the second (passenger) strand is discarded and degraded. Stabilization of siRNA guide strands by AGOs explains local hotspots and strand biases in the viral genome coverage with siRNAs, while no overall (genome-wide) strand bias is evident (Figure 3b and Figure 4a).

Since viral siRNAs cover both strands of the entire MpDV genome without gaps, we hypothesized that their precursors are produced by Pol II readthrough transcription beyond the poly(A) signals in both leftward and rightward orientation, reaching and going through the 5′- and 3′-ITRs, respectively (Figure 3a). Unprocessed readthrough transcripts spanning the viral genome from the transcription start site to the ITR in each orientation may pair to each other and thereby form long dsRNA substrates for Dicer-2. The readthrough transcripts covering the ITRs may form hairpin substrates for Dicer-2. In fact, analysis of the Illumina mRNA-seq data revealed low-abundance reads that cover the antisense strands of both NS and VP transcription units as well as parts of 5′- and 3′-ITRs (Figure S3; Dataset S2a), which likely represent (remnants of) the readthrough transcripts. Since, in both NS and VP units, the introns generate nearly as abundant siRNAs as the exons (Figure 3b; Dataset S4), we assume that unprocessed pre-mRNA readthrough transcripts form dsRNA precursors of viral siRNAs. In support of our hypothesis, both rightward and leftward readthrough transcripts covering a (near)-complete 5 Kb genome of Diaphorina densovirus (DcDV), an ambisense member of *Parvoviridae*, were identified in DcDV-infected *D. citri* psyllids by RNA blot hybridization and RT-PCR sequencing [33].

In addition to siRNAs, both NS and VP units produce less abundant sRNAs of longer size range, which belong predominantly to 26, 27 and 28 nt classes and exhibit strong (NS unit) or less strong (VP unit) biases to the mRNA sense strand (Figure 3c, Figure 4a, Figures S4a and S5; Datasets S3 and S4a). Analysis of the nucleotide composition of 26–28 nt reads revealed the predominance of uridine and adenosine at positions 1 and 10 from the 5-end, which is typical for PIWI-associated (pi)RNAs generated by a ping-pong mechanism. Thus, the antisense reads of both NS and VP units have a strong bias to uridine at position 1 (1U) (Figure 4b and Figure S6), which is typical for primary piRNAs. Conversely, the sense reads of both NS and VP unit have a strong bias to adenosine at position 10 (10A) (Figure 4b and Figure S6), which is typical for secondary piRNAs. By analogy with other animals where the ping-pong mechanism has been dissected, which include Diptera (Drosophila and Aedes) and Lepidoptera (Bombyx) insects [9,12,13], we hypothesize that precursors of the densoviral primary 1U-piRNAs are produced from the antisense readthrough transcripts by the aphid Zucchini-like endonuclease and then become associated with a 5′U-specific member of the aphid PIWI subfamily of Argonaute proteins. Upon 3′-end trimming by the aphid Trimmer-like exonuclease, the mature 26–28 nt 1U-piRNA-PIWI complexes target the complementary (sense) viral mRNAs and/or pre-mRNAs for cleavage between complementary sequence positions 10 and 11 (counting from the 5′-end of 1U-piRNA), which generates precursors of secondary 10A-piRNAs. These precursors become associated with another member of the PIWI subfamily and, upon 3′-end trimming, the mature 26–28 nt 10A-piRNA-PIWI complexes target the antisense readthrough transcripts for cleavage between complementary sequence positions 10 and 11 (counting from the 5′-end of 10A-piRNA). This cleavage generates precursors of 1U-piRNAs, thus completing the first ping-pong cycle and initiating the next one. Higher abundance of the viral sense (processed and polyadenylated) mRNAs expressed from the NS and VP units, compared to the respective antisense readthrough transcripts, would explain higher accumulation of the secondary 10A-piRNAs from both units.

Notably, uridine at position 1 is the second most frequent nucleotide in 26–28 nt sRNAs derived from the mRNA sense strands of each unit (Figure 4b and Figure S6), suggesting that the viral mRNAs may additionally but less efficiently be targeted by the Zucchini-like endonuclease generating precursors of primary 1U-piRNAs. Likewise, adenosine at position 10 is the second most frequent nucleotide in 26–28 nt sRNAs derived from the antisense strands of each unit (Figure 4b and Figure S6), suggesting that the antisense readthrough transcripts can additionally but less efficiently generate secondary 10A-piRNAs, following cleavage by the less abundant primary 1U-piRNAs produced from (pre-)mRNAs. Notably, 26–28 nt reads cover not only exons and introns but also exon–intron junctions at both 5′ and 3′ splice sites of NS and VP units (Figure 3c and Figure S5; Dataset S4a), suggesting that unspliced polyadenylated mRNAs or unprocessed pre-mRNAs can also serve as piRNA precursors.

Consistent with our findings for MpDV, the ambisense densovirus DcDV produced 28–30 nt piRNAs with the ping-pong 1U-10A signature from both NS and VP units in DcDV-infected *D. citri* psyllids [23]. Moreover, the rightward and leftward readthrough transcripts identified for DcDV [33] were proposed (i) to serve as the sense and antisense precursors required for the piRNA ping-pong cycle and (ii) to form dsRNA precursors of the viral siRNAs produced in DcDV-infected psyllids in addition to the viral piRNAs [23].

We assume that both siRNA and piRNA pathways can individually silence densoviral gene expression, because both antisense siRNAs and antisense piRNAs can target viral mRNAs or pre-mRNAs for cleavage, resulting in post-transcriptional silencing. Furthermore, siRNA and piRNA pathways can potentially compete with each other, since the same viral transcripts can serve as precursors of both siRNAs and piRNAs. It is also conceivable that viral siRNAs can initiate piRNA biogenesis by targeting viral transcripts. Indeed, siRNAs can initiate endogenous piRNA cluster formation in Drosophila [34]. Additionally, siRNAs derived from the promoter regions (Figure 3b) can potentially target viral DNA and interfere with Pol II binding, resulting in transcriptional silencing. In summary, the production and functional activity of siRNAs and piRNAs derived from different regions (and strands) of the viral genome can potentially regulate expression rates of the NS and VP genes that mediate virus replication and encapsidation, respectively.

#### 2.1.4. Impact of Aphid Feeding Conditions on MpDV Gene Expression and Antiviral RNAi

**Figure 5:**
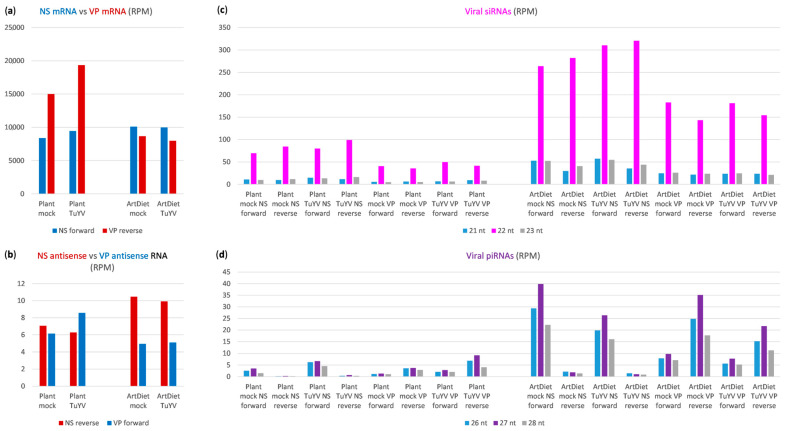
Impact of aphid feeding conditions on Myzus persicae densovirus (MpDV) gene expression and antiviral RNAi responses. Illumina mRNA- and sRNA-seq reads from aphids fed on mock-inoculated or TuYV-infected plants (Plant mock, Plant TuYV) or artificial diets without or with TuYV virions (ArtDiet mock, ArtDiet TuYV) were mapped with zero mismatches to the reference sequences of NS and VP parts of the 5873 nt MpDV genome (positions 1–2893 and 2847–5873, respectively) and the mapped reads were sorted by size and polarity (forward, reverse) and counted in reads per million (RPM) of total (host + viral) reads (Datasets S1 and S3). Counting results for combined reads from three (mRNA-seq) and two (sRNA-seq) biological replicates per feeding condition are presented as bar graphs. (**a**) Counts of mRNA-seq forward and reverse reads representing NS and VP mRNAs (blue and red bars, respectively), (**b**) Counts of mRNA-seq reverse and forward reads representing NS and VP antisense RNAs (red and blue bars, respectively). (**c**) Counts of each size class of 21–23 nt forward and reverse sRNA reads representing viral siRNAs derived from the NS and VP parts of MpDV genome. (**d**) Counts of each size class of 26–28 nt forward and reverse sRNA reads representing viral piRNAs derived from the NS and VP parts of the MpDV genome.

To test whether aphid feeding conditions and/or food composition can influence MpDV gene expression and antiviral RNAi responses, we compared the accumulation levels of viral mRNAs, siRNAs and piRNAs in aphids fed on mock-inoculated or turnip yellows virus (TuYV)-infected *Arabidopsis thaliana* plants and in those fed on artificial diets without or with purified TuYV virions. The accumulation levels were normalized in reads per million (RPM) of total (host + viral) reads derived from three (mRNA-seq) or two (sRNA-seq) biological replicates per condition (Datasets S1 and S3). In aphids feeding on plants, VP mRNAs accumulated at ca. two times higher levels than NS mRNAs, and the presence of TuYV slightly and proportionally increased accumulation of both NS and VP mRNAs (Figure 5a). The higher accumulation of VP mRNAs coincided with lower accumulation of VP siRNAs of both polarities, whose levels were ca. two times lower than those of NS siRNAs, and the presence of TuYV slightly and proportionally increased accumulation levels of both NS- and VP-derived siRNAs of both polarities (Figure 5c).

In contrast to siRNAs, VP unit-derived piRNAs accumulated at higher levels than NS unit-derived piRNAs. This was much more pronounced for the less abundant antisense piRNAs derived from the readthrough transcripts (and dominated by primary piRNAs) than for the more abundant sense piRNAs derived from mRNAs (and dominated by secondary piRNAs) (Figure 5d). Similar to siRNAs, piRNAs of both polarities from both units accumulated at slightly and proportionally higher levels in the aphids fed on TuYV-infected plants than those fed mock-inoculated plants (Figure 5d). Interestingly, the readthrough transcripts within VP and NS units accumulated at comparable levels in the aphids fed on mock-inoculated plants and these levels were only slightly affected by TuYV (Figure 5b). In summary, under plant feeding conditions, lower siRNA production from the VP genes was compensated by higher piRNA production and, vice versa, higher siRNA production from the NS genes was compensated by lower piRNA production. This supports our hypothesis on the competition of siRNA and piRNA pathways for the readthrough transcripts as precursors of both siRNAs and piRNAs.

In the aphids fed on artificial diets with or without TuYV, NS mRNAs accumulated at comparable levels that were slightly higher than those in the aphids fed on plants. In contrast, VP mRNAs accumulated at much lower levels compared to plants, and at slightly lower levels than NS mRNAs (Figure 5a). This coincided with a decreased accumulation of rightward readthrough transcripts and an increased accumulation of leftward readthrough transcripts (Figure 5b). Furthermore, the accumulation levels of viral siRNAs of both polarities from both NS and VP genes were strongly and almost proportionally increased in aphids fed on the artificial diets with or without TuYV, compared to aphids fed on the respective plants (Figure 5c). Moreover, a much stronger increase was observed in the accumulation levels of viral piRNAs, and this increase was more pronounced in the absence of TuYV for both VP and NS piRNAs of both polarities (Figure 5d). Despite the strong and proportional increase in accumulation of both sense and antisense siRNAs and piRNAs from both NS and VP genes, only VP mRNA levels were concomitantly decreased, whereas NS mRNA levels were even slightly increased (Figure 5a). Thus, neither siRNA nor piRNA pathways appeared to repress NS gene expression, whereas VP gene expression appeared to be affected by both pathways, as the accumulation levels of both siRNAs and piRNAs from VP genes inversely correlate with the accumulation levels of VP mRNAs. It remains to be investigated how NS genes evade silencing directed by viral siRNAs and piRNAs. Since NS genes encode the proteins mediating viral replication, while VP genes encode the structural proteins, RNAi-mediated regulation of VP gene expression may contribute to the transition from viral DNA replication to its encapsidation, which occurs at later stages of host cell infection. The observed differences in the relative abundance of VP and NS mRNAs under different conditions of aphid feeding likely reflect differences in proportions of the aphid cells more actively replicating viral DNA and those more actively encapsidating viral DNA.

### 2.2. A Novel RNA Virus Co-Persisting with MpDV in the Aphid Population

#### 2.2.1. Discovery and Phylogenomic Analysis of a Novel Flavivirus

**Figure 6:**
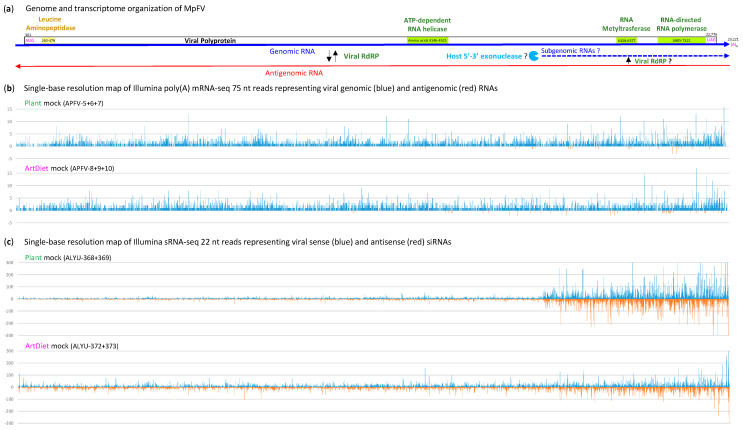
Genome, transcriptome and sRNAome of Myzus persicae flavivirus (MpFV). (**a**) MpFV genome and transcriptome organization. Viral genomic (g) and antigenomic (ag) RNAs are depicted as, respectively, solid blue and red lines with arrowheads. A single large ORF encoding the viral polyprotein is boxed, with the start and stop codon positions indicated. The polyprotein’s catalytic domains likely mediating polyprotein processing (leucine aminopeptidase) and viral RNA replication (ATP-dependent RNA helicase, RNA methyltransferase and RNA-dependent RNA polymerase (RdRP)) are highlighted in yellow and green, respectively, with their amino acid positions indicated. Putative subgenomic (sg) RNA(s) likely generated by the host 5′-3′ exonuclease-mediated partial degradation of viral gRNA is depicted as a dotted blue line. Black arrows indicate viral RdRP activities generating agRNA on the gRNA template and vice versa. (**b**) Single-base resolution map of Illumina mRNA-seq 75 nt reads representing gRNA (blue) and agRNA (red). (**c**) Single-base resolution map of Illumina sRNA-seq 22 nt reads representing viral sense (blue) and antisense (red) siRNAs. The mRNA-seq and sRNA-seq reads were mapped to the MpFV genome and the mapping data were analyzed using MISIS-2 [30] and visualized using Excel (Datasets S2b and S4b). The maps of combined reads from three (mRNA-seq) or two (sRNA-seq) biological replicates under two of the 4 feeding conditions (Plant mock and ArtDiet mock; Datasets S1 and S3) are presented as histograms that plot the numbers of 75 nt mRNA-seq (**b**) and 22 nt sRNA-seq (**c**) reads at each nucleotide position of the 23,221 nt MpFV genome; blue bars above the axis represent forward reads starting at each respective position, while red bars below the axis represent reverse reads ending at the respective position. Bars representing reads exceeding the value of 300 unique reads are cut off. Complete histograms for all feeding conditions are shown in Datasets S2b and S4b and Figure S9.

De novo (Velvet/Oases) assembly of sRNA reads from the above-described aphid samples, followed by filtering of the resulting contigs through the *M. persicae* and MpDV genomes, revealed large contigs representing a novel virus. SeqMan scaffolding of the Oases contigs and the mRNA-seq Trinity contigs, followed by consensus sequence verification with redundant sRNA and mRNA reads, yielded a 23,221 nt genome encoding a single large polyprotein of 7491 amino acids (Figure 6a). Nucleotide BLASTn and protein BLAST analyses revealed distant similarities to Macrosiphum euphorbiae virus 1 (MeV-1) [75% nt identities at 39% coverage of its genome (NC_028137.1) and 58% amino acid identity at 95% coverage of its polyprotein (YP_009175071)] and Sitobion miscanthi flavi-like virus 1 (SnFLV-1) [66% nt identities at 26% coverage of its genome (MH7781489) and 48% amino acid identity at 94% coverage of its polyprotein (QCI31815)]. These viruses are positive-sense ssRNA viruses classified into the family *Flaviviridae* and have both been identified in aphids, the potato aphid *Macrosiphum euphorbiae* [35] and the Indian grain aphid *Sitobion miscanthi* [36], respectively. We therefore named our new virus Myzus persicae flavivirus (MpFV; deposited in NCBI Genbank under accession PQ247847) and tentatively classified it as a new species into the family *Flaviviridae*.

Interestingly, the *M. euphorbiae* aphid population carrying the flavivirus MeV-1 was co-infected with a densovirus and a tombus-like virus and exhibited no obvious pathology [37]. Thus, members of *Flaviviridae* and *Parvoviridae* can co-persist in their Aphididae hosts. Analysis of the Illumina-seq data generated in this study (20 aphid pools), as well as in our previous study of another clonal population of *M. persicae* (WMp2, originating from the Netherlands) maintained at INRAE-Colmar (18 aphid pools; [38]), revealed that each aphid pool was positive for both MpDV and MpFV (Datasets S1, S3, S5 and S6). Thus, the two independent clonal populations of *M. persicae* (the French Mp-Col and the Dutch WMp2) are persistently co-infected with MpDV and MpFL. On the other hand, our Illumina sRNA-seq analysis of the third aphid population originating from a single *M. persicae* Mp-Col aphid negative for MpDV [6], which was maintained in an MpDV-free chamber at INRAE-Colmar, was found to be also negative for MpFV (Figure S4d; Dataset S3; ALYU-187 vs. ALYU-188). Thus, the flavivirus’ persistence in aphids may depend on the densovirus or vice versa.

InterPro analysis of the MpFV-encoded polyprotein revealed ATP-dependent RNA helicase (amino acids 4146–4525), SAM-dependent RNA methlytransferase (amino acids 6358–6577) and RNA-directed RNA polymerase (RdRP; amino acids 6805–7321) domains (Figure 6a). These catalytic domains are present at similar positions in both MeV-1 and SnFLV-1 polyproteins (Figure S7b,c) and likely mediate viral replication. In addition, the N-terminal part of MpFV polyprotein contains a Macro domain-like leucine aminopeptidase (amino acids 240–378) (Figure 6a) that may participate in polyprotein processing. Interestingly, this domain is present at the same N-terminal position in MeV-1, while it is located at another position in SnFLV-1 (Figure S7b,c). All three viruses encode within the N-terminal and middle parts of their polyproteins the cytoplasmic domains surrounded by the transmembrane domains (Figure S7), which may constitute the structural proteins involved in formation of enveloped virions, as shown for other flaviviruses ([39]; https://ictv.global/report/chapter/flaviviridae/flaviviridae (accessed on 1 October 2024)).

The genomic (g)RNAs of MpFV, MeV-1 and SnFLV-1 contain 5′-untranslated regions (UTRs) of comparable lengths (300, 290 and 298 nts, respectively) which do not possess any AUG and can form relatively stable secondary structures (Figure S8a). Likewise, the gRNA 3′-UTRs of the three viruses are long (448, 491 and 447 nts, respectively) and structured (Figure S8b). The long structured 5′- and 3′-UTRs are likely involved in the regulation of gRNA replication and translation. MpFV gRNA appears to be polyadenylated since the reconstructed MpFV genome contains a 6 nt poly(A) tract at the 3′-terminus and is covered with Illumina reads obtained from a poly(A)-enriched fraction of aphid RNA (Figure 6b and Figure S9a). MeV-1 gRNA was also found to be polyadenylated [35], while SnFLV-1 gRNA assembled from Illumina Ribo-Zero RNA-seq reads had no poly(A) tail [36].

Analysis of single-base resolution maps of the Illumina mRNA-seq reads (Dataset S4b) revealed that the entire positive-sense (gRNA) strand of MpFV genome is almost uniformly covered with low-abundance reads, with the 5′- and 3′-terminal regions exhibiting lower and higher read density, respectively (Figure 6b and Figure S9a; Dataset S4a). On the other hand, the negative-sense strand (antigenomic RNA) is represented by a very low number of reads, mostly derived from the 3′-part of the MpFV genome (Figure 6b and Figure S9a; Dataset S4b). This finding likely reflects the low abundance of antigenomic RNA as the replicative intermediate (Figure 6a).

Three common features of MpFV, MeV-1 and SnFLV-1 justify their classification into a new genus of the family *Flaviviridae*, for which we propose the name *Aphidiflavivirus*. Firstly, these viruses infect aphids from different genera of the family Aphididae feeding on plants, unlike flaviviruses of the genus *Orthoflavivirus*, which infect mosquitoes and ticks feeding on vertebrate animals. Secondly, they have genomes of comparable sizes (23.2, 22.8 and 23.1 Kb, respectively) which greatly exceed the genome sizes of orthoflaviviruses and other established genera of *Flaviviridae* (ca. 9–13 Kb; https://ictv.global/report/chapter/flaviviridae/flaviviridae (accessed on 1 October 2024); [39]). Thirdly, they encode single large polyproteins possessing replicase domains and potential structural domains at similar positions. Another notable difference between aphidiflaviviruses and orthoflaviviruses is that the former appear to be restricted to insect hosts, unlike the latter. Indeed, MeV-1 deposited from aphids to leaves was unable to replicate locally or spread systemically in the aphid-infested plant [35]. Likewise, MpFV did not appear to replicate in the plants infested by *M. persicae* aphids: our Illumina mRNA-seq and sRNA-seq analysis of the leaf samples taken from these plants [40] revealed very low numbers of MpFV-derived reads, likely representing remnants of aphid infestation (Datasets S7 and S8), whereas all the aphid samples collected from these plants were positive for MpFV (Datasets S5 and S6).

#### 2.2.2. Host RNAi Responses to MpFV

**Figure 7:**
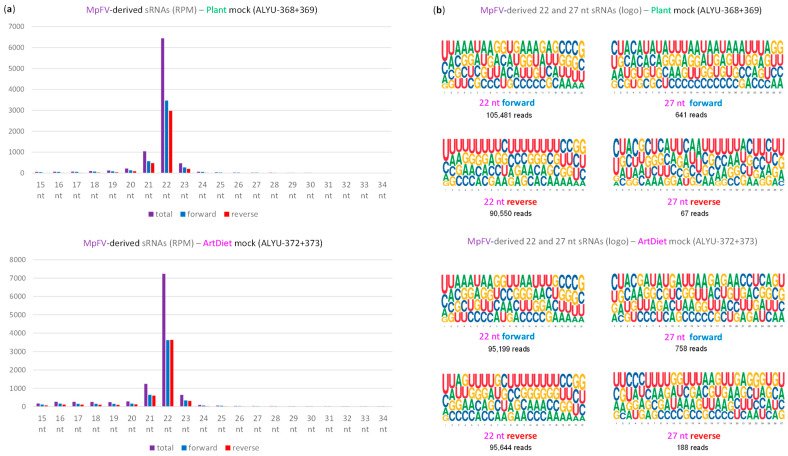
Size profiles and nucleotide compositions of Myzus persicae flavivirus (MpFV)-derived sRNAs in *M. persicae* aphids fed on plants vs. artificial diets. Illumina 15–34 nt reads were mapped with zero mismatches to the MpFV genome and the mapped reads were sorted by size and polarity (forward, reverse, total) and counted in reads per million (RPM) of total (host + viral) reads (Dataset S3). (**a**) Counting results for combined reads from 2 biological replicates under two of the 4 feeding conditions (plant mock ALYU-368+369 and ArtDiet mock ALYU-372+373) are shown as bar graphs representing counts of each size class of viral sRNAs. (**b**) Nucleotide compositions of 22 nt and 27 nt forward and reverse sRNAs derived from MpFV under the two feeding conditions are presented as RNA logos with the numbers of reads shown under each logo. Size profiles and nucleotide compositions MpFV sRNAs under the other two conditions are shown in Figure S10.

Analysis of size profiles, nucleotide compositions and single-base resolution maps of the Illumina sRNA reads revealed that both strands of MpFV genome are covered with abundant 22 nt sRNAs, followed by less abundant 21 and 23 nt classes, with the most frequent 5′-nucleotide of these three size classes being U, followed by C and A (Figure 6c, Figure 7, Figures S9 and S10; Dataset S4b). Such size and 5′-nucleotide frequency profiles, together with complete genome coverage and no overall strand bias, suggest that genomic-antigenomic dsRNA intermediates of MpFV replication (Figure 6a) are targeted by the aphid Dicer-2 generating 22 nt sRNA duplexes. The aphid AGOs sort these duplexes and stabilize guide strands of viral siRNAs based on 5′-nucleotide identity. Notably, the relative abundance and nucleotide composition of 21, 22 and 23 nt sRNAs of sense and antisense polarities derived from MpFV and MpDV are very similar (Figure 7 and Figure S10 vs. Figure 4 and Figure S6), indicating that both viruses are targeted by the same siRNA-generating machinery. However, unlike MpDV, MpFV does not appear to be targeted by the piRNA-generating machinery, since 26–28 nt sRNAs are under-represented and do not display the 1U-10A ping-pong signature of piRNAs (Figure 7b and Figure S10b).

Comparison of MpFV sRNA genome coverage profiles under different aphid feeding conditions revealed a striking difference between the plant and the artificial diet, which was independent of TuYV. Under the plant feeding condition, a 3′-terminal 6 Kb region (positions 17,167–23,221) produced much more abundant siRNAs of both polarities than the preceding three quarters of the MpFV genome, whereas under the artificial diet condition, the relative rates of siRNA production from the respective regions differed much less pronouncedly (Figure 6c and Figure S9b; Dataset S4b). We hypothesize that the 6 Kb region of MpFV genome produces a positive-sense subgenomic RNA (sgRNA) that can be converted to dsRNA by viral RdRP. Dicer-2 processing of the resulting dsRNA would generate siRNAs spanning the 6 Kb region, while Dicer-2 processing of the genomic-antigenomic dsRNA replicative intermediates would generate siRNAs spanning the entire genome. Relative abundance of siRNAs produced from the 6 Kb region and the rest of the viral genome would therefore depend on sgRNA production rate, which could be regulated during the viral infection cycle and be affected by the aphid feeding conditions.

Theoretically, sgRNAs can be produced by viral RdRP-mediated transcription of the antigenomic RNA, which is initiated at an internal promoter, and/or by host 5′-3′-exonuclease-mediated partial degradation of the genomic RNA, which is terminated at internal structural elements. In mammalian and insect flaviviruses, 3′-coterminal sgRNAs are known to be products of incomplete degradation of viral gRNA by the host 5′-3′ exonuclease XRN1 that stalls in highly structured elements in the 3′-UTR [41]. We found that a 5′-end of the presumptive 6 Kb sgRNA of MpFV is imbedded into a secondary structure composed of five hairpins. The two hairpins located downstream of the 5′-terminus are more stable than the others (Figure S11) and can therefore stall the presumptive 5′-3′ exonuclease and stabilize the 6 Kb sgRNA. If the exonuclease overcomes the stalling, it will proceed with gRNA processing until the next structural element and eventually reach the stable secondary structure within 3′-UTR (Figure S7b). This process may create 3′-coterminal sgRNAs of different lengths, whose steady-state levels would depend on the exonuclease’s processivity. In turn, the relative abundance of the long (6 Kbp) and shorter dsRNAs produced by viral RdRP on the respective sgRNA templates and hence the resulting siRNAs would differ. This hypothesis is supported by the single-base resolution maps of MpFV siRNAs showing a gradual increase in siRNA density along the 6 Kb region and the hottest spots of siRNA production within the 3′-UTR (Figure 6c and Figure S9b).

**Figure 8:**
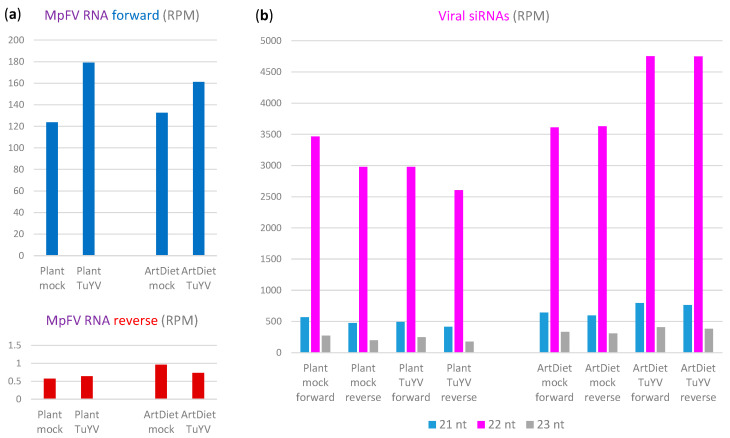
Impact of aphid feeding conditions on Myzus persicae flavivirus (MpFV) replication and antiviral RNAi responses. Illumina mRNA- and sRNA-seq reads from aphids fed on mock-inoculated or TuYV-infected plants (Plant mock, Plant TuYV) or artificial diets without or with TuYV virions (ArtDiet mock, ArtDiet TuYV) were mapped with zero mismatches to the MpFV genome and the mapped reads were sorted by size and polarity (forward, reverse) and counted in reads per million (RPM) of total (host + viral) reads (Datasets S1 and S3). Counting results for combined reads from three (mRNA-seq) and two (sRNA-seq) biological replicates per feeding condition are presented as bar graphs. (**a**) Counts of mRNA-seq forward reads representing MpFV genomic RNA (blue bars) and mRNA-seq reverse reads representing MpFV antigenomic RNA (red bars). (**b**) Counts of each size class of 21–23 nt forward and reverse sRNA reads representing viral sense and antisense siRNAs derived from MpFV genomic and antigenomic RNAs, respectively.

The drastic difference in relative rates of siRNA production from the 6 Kb sgRNA region and the preceding part of the 23.2 Kb MpFV genome under the plant versus artificial diet conditions did not coincide with any substantial changes in viral gRNA accumulation, although the presence of TuYV resulted in its increase under both conditions (Figure 8a). Concomitant with the increased accumulation of MpFV gRNA, the accumulation of total viral siRNAs (derived from the entire virus genome) is almost proportionally decreased in plants infected with TuYV and almost proportionally increased on the artificial diet with TuYV virions, compared to the respective controls without TuYV (Figure 8b). Furthermore, whereas sense and antisense viral siRNAs accumulated at similar levels under the artificial diet conditions, under the plant feeding conditions, the antisense siRNAs accumulated at somewhat lower levels than the sense siRNAs (Figure 8b). Based on these findings, the functionality (if any) of the higher-abundance siRNAs produced from the 6 Kb region of MpFV cannot be conclusively inferred. We speculate that both the presumptive sgRNAs and the dsRNA precursors of viral siRNAs produced from this region may serve as decoys diverting the antiviral RNAi machinery from the gRNA molecules engaged in translation and/or replication. It is conceivable that the antiviral activity of the host 5′-3 exonuclease degrading viral gRNA is counteracted by the gRNA structural elements stalling the exonuclease and thereby generating sgRNA decoys for antiviral RNAi.

### 2.3. M. persicae Aphids Fed on TuYV (Positive-Sense ssRNA Polerovirus)-Infected Plants Accumulate Sense and Antisense sRNAs Reconstituting a Complete TuYV Genome

**Figure 9:**
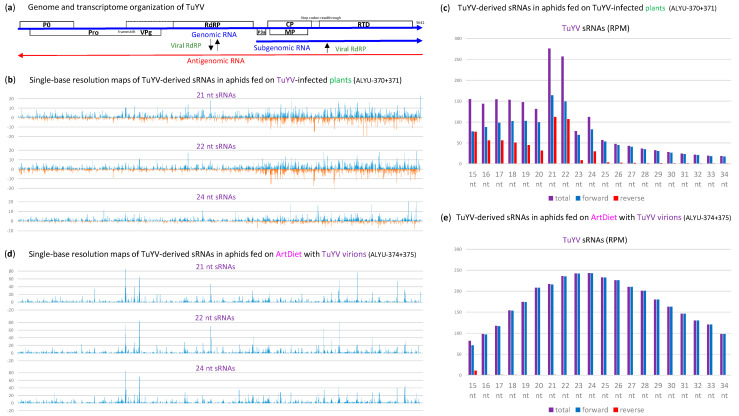
Turnip yellows virus (TuYV)-derived sRNAs in *M. persicae* aphids fed on TuYV-infected *A. thaliana* plants versus artificial diet with purified TuYV virions. (**a**) TuYV genome and transcriptome organization. Viral genomic (g), subgenomic (sg) and antigenomic (ag) RNAs are depicted as blue (gRNA and sgRNA) and red (agRNA) lines, with ORFs boxed and the encoded proteins named. Black arrows indicate viral RdRP activities generating agRNA on the gRNA template and vice versa as well as sgRNA on the agRNA template. (**b**–**e**) Single-base resolution maps and size profiles of TuYV-derived sRNAs identified in aphids fed on TuYV-infected plants or an artificial diet with TuYV virions. Illumina 15–34 reads from two biological replicates under each feeding condition were mapped to the TuYV genome with zero mismatches and the mapped reads were sorted by size and polarity (forward, reverse, total) and counted in reads per million (RPM) of total (host + viral) reads (Dataset S3). The mapping data were analyzed using MISIS-2 [30] and visualized using Excel (Dataset S4c). (**b**,**d**) Single-base resolution map of TuYV-derived 21, 22 and 24 nt sense (blue) and antisense (red) sRNAs from aphids fed on TuYV-infected plants (**b**) or on the artificial diet with TuYV virions (**d**). In both panels, histograms plot the reads of respective size classes at each nucleotide position of the 5641 nt TuYV genome: blue bars above the axis represent forward reads starting at each respective position, while red bars below the axis represent reverse reads ending at the respective position. (**c**,**e**) Relative abundance of TuYV-derived 15 to 34 nt sRNAs from aphids fed on TuYV-infected plants (**c**) or the artificial diet with TuYV virions (**e**). In both panels, bars represent counts of total (purple), forward (blue) and reverse (red) sRNA reads of each size class.

Besides sRNA contigs reconstituting the MpDV and MpFV genomes, large sRNA contigs covering a complete genome of TuYV were obtained by Velvet/Oases assembly of Illumina sRNA-seq reads from the *M. persicae* aphids viruliferous for this plant virus. Analysis of single-base resolution maps revealed that both strands of the entire TuYV genome (Figure 9a) are covered with sRNA reads from the aphids fed on TuYV-infected plants (Figure 9b; Dataset S4c). TuYV-derived sRNAs belong to a broad size range (from 15 to 34 nts) and each size class (except 15 nt) exhibits a bias to the positive-sense (gRNA) strand, but the 21 and 22 nt classes are the most abundant on both strands and exhibit much less pronounced biases to the sense strand (Figure 9c).

Theoretically, TuYV-derived 21 and 22 nt sRNAs of sense and antisense polarities could be produced by the plant Dicer-like (DCL) 4 and DCL2, respectively [11,24,42], and then acquired by the aphids feeding on the phloem sap of TuYV-infected plants. Indeed, plant phloem sap was found to contain viral siRNAs derived from a positive-sense ssRNA virus of the family *Closteroviridae* [43]. Alternatively or additionally, TuYV sRNAs could be produced in aphid cells by the aphid RNAi machinery. The polerovirus TuYV and other positive-sense ssRNA viruses of the family *Solemoviridae* are known to be transmitted by their insect vectors in a circulative non-propagative manner, in which virions containing gRNA, acquired with the phloem sap of infected plant, circulate in the insect body without replication ([44]; https://ictv.global/report/chapter/solemoviridae/solemoviridae/ (accessed on 1 October 2024)). Nonetheless, it is conceivable that viral gRNA could be released from virions during the transcytosis of the aphid midgut and salivary gland cells to undergo limited translation and replication, which would be restricted by the antiviral RNAi generating virus-derived siRNAs. Our analysis of the Illumina sRNA-seq reads from the viruliferous aphids fed on the artificial diet with purified TuYV virions revealed abundant viral sRNAs of a broad size range, which were derived almost exclusively from TuYV gRNA (Figure 9d,e). This finding is indicative of non-RNAi degradation of viral gRNA in the aphid body. The negligible amounts of sRNA reads representing TuYV antigenomic RNA (Figure 9e; Datasets S3 and S4c) suggest that viral gRNA did not undergo any substantial replication that would trigger antiviral RNAi. In further support of this hypothesis, the nucleotide compositions of 21–23 nt and 26–28 nt reads representing TuYV in the viruliferous aphids did not resemble those of MpFV- and MpDV-derived 21–23 nt siRNAs and MpDV-derived 26–28 nt piRNAs, respectively (Figure S12 vs. Figures S6 and S10b).

Our analysis of the Illumina mRNA-seq data revealed very-low-abundance reads representing TuYV gRNA and no reads derived from antigenomic RNA in aphids fed on TuYV-infected plants. More abundant reads covering TuYV gRNA and negligible amounts of reads representing its antigenomic RNA were detected in aphids fed on the artificial diet supplied with purified TuYV virions (Figure S13d; Dataset S2c). These findings further support the non-propagative circulation of TuYV virions in the viruliferous aphids without any substantial replication of viral gRNA in aphid cells.

Taken together, the above findings suggest that the aphids feeding on TuYV-infected plants acquire phloem-mobile viral siRNAs produced by the plant RNAi machinery (likely by DCL4 and DCL2) from dsRNA intermediates of viral gRNA replication and, in addition, accumulate broad-size-range products of non-RNAi degradation of viral gRNA. The latter process explains the much stronger positive strand bias observed for viral sRNAs in a size range of 25 to 34 nts (which is longer than the plant siRNA size range). The viral sense and antisense sRNAs of shorter-than-plant siRNA size-range, which also accumulate in aphids (Figure 9c), likely comprise partial degradation products of the viral siRNAs produced in planta. In further support of our mobile siRNA hypothesis, the densities and relative abundances of 21 and 22 nt sRNAs of both polarities derived from the TuYV sgRNA region in the aphids fed on TuYV-infected plants are much higher than those derived from the preceding part of TuYV genome (Figure 9b). This is similar to TuYV genome coverage with the respective size classes of viral siRNAs produced in TuYV-infected *A. thaliana* [42]. Furthermore, the viruliferous aphids from TuYV-infected plants accumulate 24 nt sRNAs of lower abundance, which are also derived from both strands of the entire TuYV genome and are enriched in the sgRNA region (Figure 9b,c). In *A. thaliana* plants, this size class of viral and endogenous siRNAs is generated by DCL3 [10,11,24] and the endogenous 24 nt siRNAs are phloem-mobile and functional in systemic silencing [45]. Collectively, our findings, together with those of previous studies, suggest that viral 21, 22 and 24 nt siRNAs are produced by the respective plant DCLs from dsRNA intermediates of TuYV gRNA replication and sgRNA transcription and then are ingested by aphids with the phloem sap. It remains to be investigated whether DCLs and other components of the plant RNAi machinery are indeed involved in the biogenesis of viral sRNAs ingested by *M. persicae* aphids and whether these sRNAs have any function in TuYV transmission by the aphids.

### 2.4. Concluding Remarks

In this study, we show that deep sRNA sequencing is applicable for the identification and de novo reconstruction of the *M. persicae* virome components. Indeed, the known ssDNA densovirus MpDV and the novel ssRNA flavivirus MpFV, which co-persisted in the aphid population, were found to produce siRNAs densely covering the entire genomes of both viruses so that de novo assembled sRNA contigs could be scaffolded into complete viral genomes. Likewise, the aphids fed on plants infected with the ssRNA virus TuYV accumulated sRNAs densely covering the entire genome of TuYV and building large contigs that reconstitute the complete viral genome. This plant virus is transmitted by *M. persicae* aphids in a circulative non-propagative manner [44] and we found no evidence for its replication in the aphid cells, which would trigger aphid antiviral RNAi responses to generate viral siRNAs or piRNAs. Instead, our findings suggest that TuYV siRNAs produced by the plant RNAi machinery could be acquired by the aphids feeding on the plant phloem sap. The sRNAomics approach based on deep sRNA sequencing and bioinformatics has been established as a universal tool for identification and de novo reconstruction of all types of RNA and DNA viruses, viral satellites and viroids that infect land plants [11]. It remains to be further investigated whether this approach can also be used as a universal tool for identification and de novo reconstruction of all types of RNA and DNA viruses replicating in the insect cells and all types of plant viruses transmitted by insect vectors. In fact, the insect RNAi machinery generates viral siRNAs not only from the entire genomes of ssDNA and positive-sense ssRNA viruses as shown here and elsewhere [23,46], but also from the entire genomes of negative-sense ssRNA [47] and dsRNA [48] viruses, as well as from near-complete (or big portions of) genomes of large dsDNA viruses [49,50,51,52]. In the latter case, readthrough transcription on both strands of the viral dsDNA genome was implicated in the production of dsRNA substrates for Dicer-2 [49].

Our sRNAomics and transcriptomics analyses of *M. persicae* aphids persistently co-infected with the densovirus MpDV and the flavivirus MpFV revealed that MpDV is targeted by both siRNA- and piRNA-generating RNAi machineries, while MpFV is targeted only by the siRNA-generating machinery. Based on the current knowledge of other insect flaviviruses and densoviruses, it is likely that MpFV gRNA replicates in the cytoplasm and does not visit the nucleus, whereas MpDV gDNA is transcribed in the nucleus and its mRNAs are translated in the cytoplasm. Based on the above-described results, we hypothesize that MpFV-derived siRNAs are generated by Dicer-2 processing of the dsRNA substrates produced in the cytoplasm by viral RdRP during gRNA replication and sgRNA transcription. By contrast, MpDV-derived siRNAs are generated by Dicer-2 processing of the dsRNA substrates produced in the nucleus from sense and antisense readthrough transcripts. On the other hand, MpDV-derived piRNAs are generated by a ping-pong mechanism engaging both nuclear readthrough transcripts and cytoplasmic mRNAs. This hypothesis implies nuclear-cytoplasmic partitioning of different components of the piRNA- and siRNA-generating machineries. We found that MpDV- and MpFV-derived siRNAs have very similar 5′-nucleotide frequency and size profiles, suggesting that the same siRNA-generating machinery is involved in the biogenesis of densoviral and flaviviral siRNAs. However, in Drosophila cells, Dicer-2 and its cofactor R2D2 are localized exclusively in the cytoplasm [53]. It remains to be established whether the *M. persicae* homolog of Dicer-2 can be (re-)localized in the nucleus where the densoviral readthrough transcripts would form dsRNA precursors of viral siRNAs or those transcripts or precursors could be transported to the cytoplasm. The nuclear-cytoplasmic partitioning of the piRNA- and siRNA-generating machineries is supported by the study of Aedes albopictus densovirus 1 (AalDNV-1). During persistent infection in *Ae. aegypti* cells, this monosense densovirus produced predominantly 21 nt siRNAs from both strands of its genome and 27–30 nt piRNAs mostly from the sense strand. The low-abundance primary 1U-piRNAs derived from the antisense strand accumulated in both the nucleus and the cytoplasm, whereas the ping-pong-dependent 10A-piRNAs derived from the sense strand accumulated only in the cytoplasm. In contrast, viral siRNAs of both polarities accumulated in both the nucleus (where they were more abundant than primary piRNAs) and the cytoplasm (where they were as abundant as the ping-pong piRNAs) [46]. Notably, in Dicer-2 knockout-mutant *Ae. aegypti* cells, siRNAs derived from a defective Aedes albopictus densovirus disappeared, but production of densoviral piRNAs was increased [46], thus supporting the hypothesis that the siRNA- and piRNA-generating machineries compete for the same viral transcripts, possibly in the same compartment(s).

Our analysis of viral sRNAs revealed not only similarities between the RNAi machineries of *M. persicae* aphid and other insects but also notable differences. Thus, MpDV siRNAs were dominated by the 22 nt class, while its piRNAs were dominated by the 26, 27 and 28 nt classes, with the 27 nt class being the most abundant in all aphid samples (Figure S4a). In contrast, the related ambisense densovirus DcDV predominantly produced 21 nt siRNAs in DcDV-infected *D. citri* psyllids, while DcDV piRNAs were dominated by the 28, 29 and 30 nt classes, with the 29 nt class being the most abundant [23]. This size profile difference is also evident for the monosense densovirus AalDNV-1 in mosquitoes, as described above [46], and implies structural differences in *M. persicae* versus *D. citri* and mosquito homologs of Dicer-2 and PIWI proteins. Indeed, Dicer-2 and PIWIs serve as molecular rulers determining the size of siRNA duplexes (processed by Dicer-2 from dsRNA) and mature piRNAs (bound to PIWIs following 3′-end trimming).

Our Illumina sequencing analysis of MpDV sRNAs and poly(A)-enriched long RNAs provide strong evidence for bidirectional readthrough transcription of the entire densovirus genome mediated by Pol II. Notably, the readthrough transcription is reaching and going through the 5′- and 3′-terminal telomere sequences, which are completely and densely covered with viral siRNA reads. Since hairpin structures of both telomers are formed by near-identical direct terminal repeats of the palindromic sequences differing by the 1-nt insertion in an ascending arm of the 3′-telomer (Figure 1) and this insertion is represented in a large proportion of siRNA reads (ca. 50 to 70%; Dataset S9), we cannot definitively infer whether readthrough transcripts cover the entire hairpin sequences or only their ascending arms. In the case of a heterotelomeric monosense densovirus in *Culex pipiens molestus* mosquitoes, viral siRNAs were derived only from the inverted repeats (but not direct repeats) present at both termini of the virus genome [54], suggesting that readthrough transcription may proceed through the inverted repeats and the resulting transcript may fold into hairpin dsRNA substrate for Dicer-2. In the homotelomeric ambisense MpDV, bidirectional readthrough transcription likely occurs on circular covalently closed dsDNA molecules produced by the nuclear DNA repair machinery on the viral genomic and antigenomic ssDNA templates with symmetrical Y-shaped hairpins, following their release from virions. In fact, homotelomeric ambisense members of the *Parvoviridae* encapsidate both genomic and antigenomic ssDNA molecules that are converted in the nucleus into circular dsDNA templates for both Pol II transcription and rolling-hairpin replication ([27,28]; https://ictv.global/report/chapter/parvoviridae/parvoviridae (accessed on 1 October 2024)). It is conceivable that the entire circular dsDNA is transcribed by Pol II that initiates at the rightward or leftward promoters, proceeds along the genomic or antigenomic DNA template, goes through the telomere palindrome sequence and continues on the antigenomic or genomic template, respectively. Such readthrough transcription would produce partial or complete genomic-antigenomic ssRNA concatemers folding into dsRNA and serving as substrates for Dicer-2. Even if readthrough transcription is retarded by and terminated within the telomers, those forward and reverse transcripts spanning the MpDV genome in both directions but lacking complete palindromic sequences (Figure S3) can pair to each other (or to VP and NS mRNAs, respectively) and thereby form dsRNA precursors of siRNAs or can directly serve as precursors of primary piRNAs.

In further support of readthrough transcripts’ involvement in the biogenesis of both siRNAs and piRNAs, chromatin profiling in mosquitoes has identified readthrough transcription as a conserved mechanism generating primary 1U-piRNAs at multiple non-retroviral EVEs that represent different viral families [55]. EVE-derived piRNAs likely play a role in defenses against cognate episomal viruses as demonstrated for the EVE cognate to a flavivirus in *Ae. aegypti* [7].

We found that *M. persicae* feeding conditions strongly affect RNAi responses to both MpDV and MpFV. Indeed, the piRNAs derived from MpDV and the siRNAs derived from both viruses accumulated at higher levels in aphids fed on artificial diets than in those fed on plants, which coincided with alterations in MpDV rightward-to-leftward transcription ratio and MpFV sgRNA production rate. Moreover, the presence of TuYV in plants and artificial diets further modulated the RNAi responses and viral gene expression. In a previous study of *M. persicae* aphids [21], alterations in size profiles of MpDV-derived viral sRNAs and endogenous sRNAs were observed in response to potato leaf roll virus (PLRV). Thus, aphids fed on the PLRV-infected plant or the artificial diet with purified PLRV accumulated higher levels of 26–27 nt endogenous sRNAs at the expense of 22 nt sRNAs, compared to those fed on mock-inoculated plants or artificial diet without PLRV, in which the 22 nt class was the most abundant. However, MpDV sRNA size profiles differed from those observed in our study in that the 22 nt class was not predominant, showing higher accumulation only in aphids fed on mock-inoculated plants but not in aphids fed on PLRV-infected plants or on the artificial diet with PLRV virions [21]. Interestingly, the size profile of endogenous sRNAs in *Aphis gossypii* cotton-melon aphids fed on aphid-susceptible melon plants was dominated by 22 nt reads, whereas in those fed on aphid-resistant melon plants, 26–27 nt reads were over-represented [56]. Our analysis of the global size profile of endogenous sRNAs in *M. persicae* aphids did not reveal any notable differences under different feeding conditions: in all cases, a bimodal size profile was observed, with the most predominant 22 nt class likely representing endogenous siRNAs and miRNAs and the less abundant 26–28 nt classes likely representing endogenous piRNAs (Figure S4b,c). Further analysis of our RNA-seq data is required to address the question of whether aphid feeding conditions might affect siRNA or piRNA production at specific loci in the aphid genome, such as those containing transposons or EVEs.

It is worth noting that persistent co-infection of *M. persicae* aphids with MpDV and MpFV did not interfere with the acquisition of the circulative ssRNA (TuYV) or non-circulative dsDNA-RT (CaMV) viruses from TuYV- and CaMV-infected *A. thaliana* or *Camelina sativa* plants [38,57] and the transmission of these viruses to recipient plants (our unpublished data), as all the aphid samples from those plants were positive for both MpDV and MpFV. In support of this notion, infection of *M. persicae* aphids with MpnDV isolated from *M. persicae nicotianae* [5] was reported to facilitate circulative transmission of a positive-sense ssRNA potyvirus [19]. It remains to be investigated whether MpDV and/or MpFV infection of *M. persicae* aphids can influence circulative and/or non-circulative transmission of plant viruses.

Our finding that MpDV’s persistence in the aphid population may depend on MpFV, or vice versa, raises a question on possible synergistic interactions between the densovirus and the flavivirus. It is conceivable that putative densoviral and flaviviral effector proteins may synergistically suppress antiviral defenses based on RNAi and/or innate immunity by targeting and inactivating different components of the RNAi and/or innate immunity machineries. Both viruses may also evade (through mutation) the sequence-specific RNAi and immune responses targeting non-self viral nucleic acids and proteins. In fact, we observed the ongoing evolution of MpDV quasispecies that has accumulated multiple SNPs and short indels in both protein-coding and non-coding sequences of the virus genome over many years since the first isolate of this virus was purified from the clonal aphid population and sequenced [3]. We cannot exclude, however, that those indels in the protein-coding sequences that shifted the translation frame and resulted in shorter NS1 and VP1 proteins in the first MpDV isolate were sequencing errors. Indeed, comparative analysis of our French strain of MpDV (MpDV1) and other strains of MpDV isolated from *M. persicae* (MpDV2) and *M. persicae nicotianae* (MpnDV) aphids in China revealed that all these variants of MpDV have the four main ORFs of the same length, while they differ by indels in the shared non-coding sequences (File S1c). Notably, the complete and seemingly incomplete (78 nt shorter) ITRs found in MpDV1 and MpDV2, respectively, have imperfect direct repeats of the palindromic sequences of, respectively, longer and shorter size that form symmetrical Y-shaped hairpin telomeres with long and shorter hairpins and distinct small stem-loops at the hairpins’ top (Figure S1b). Since parvoviral telomeres are the origins of replication and encapsidation which are specifically recognized by the viral replication initiator (NS/Rep) and virion (VP) proteins, it remains to be investigated whether and how the differences in the primary sequences and secondary structures of the telomeres of the French and Chinese strains of MpDV regulate their replication and encapsidation. We also found that all the variants of MpDV (MpDV1, MpDV2 and MpnDV) preserve cis-elements driving viral mRNA transcription, splicing and polyadenylation in both NS and VP units (File S1c), suggesting conserved strategies of the NS and VP gene expression. Indeed, our mRNA-seq analysis of MpDV1 transcripts and previous RACE-PCR analyses of MpDV2 and MpnDV transcripts revealed general similarities in transcription initiation, splicing and polyadenylation sites, with only a few differences described above. In addition, our Trinity assembly and quantitative analysis of the mRNA-seq data revealed complex regulation of viral gene expression in both NS and VP units involving major and minor transcription start sites for major and minor alternatively spliced and unspliced mRNAs that can potentially be translated into different versions of NS1, NS2, VP1 and VP2 proteins, as well as NS2-NS1 and VP2-VP1 fusion proteins (Figure 2a). All these proteins can potentially mediate or regulate viral replication and encapsidation, as well as virus–host interactions.

Finally, our comparative analysis of genome organization and the polyprotein catalytic and structural domains of the novel flavivirus MpFV and two related flaviviruses identified in other aphid species allowed us to propose a new genus in the family *Flaviviridae*—*Aphidiflavivirus*—comprising the three aphid flaviviruses, which have much larger genomes and other features distinguishing them from the established genera of flavivirids. It remains to be further investigated whether MpFV and other aphidiflaviviruses have any impact on the development and performance of aphids and/or on their ability to host other viruses (such as densoviruses) and transmit plant viruses and non-viral pathogens.

## 3. Materials and Methods

### 3.1. Aphids

Two genotypes of the green peach aphid (*Myzus persicae* Sulzer, 1776) were used in our experiments. The clone Mp-Col originated from Colmar (France) and was maintained at INRAE-Colmar since 1975 on sweet pepper (*Capsicum annuum*). It was shown to be persistently infected with MpDV [2,3] and coinfected with MpFV (this study). An MpDV-free population of Mp-Col aphids was obtained by transferring individual larvae directly after birth onto beet (*Beta vulgaris*) plants to avoid contamination by contact, followed by PCR analysis [6]. Since then, one Mp-Col aphid colony cured of MpDV was maintained on sweet pepper in a separate chamber and, in this study, it was found to be negative for both MpDV and MpFV (Figures S4d and S14). The *M. persicae* clone WMp2 was isolated in the Netherlands [58] and maintained at INRAE-Colmar since 1992 on Chinese cabbage (*Brassica rapa* L. pekinensis var. Granaat). It was shown to be co-infected with MpDV and MpFV (this study). All aphids were maintained in growth chambers at 20 °C and a 16 h photoperiod.

### 3.2. MpDV Virion DNA Sequencing and Reconstruction of Its 5′- and 3′-ITRs

MpDV virion purification from *M. persicae* Mp-Col aphids was performed as described previously [3]. Purified virions were incubated in an extraction buffer (100 mM EDTA, 10 mM Tris-HCl, 0.1% SDS, 100 mg/mL proteinase K, pH 8.0) for 2 h at 55 °C. The mixture was centrifuged at 14,000 rpm for 2 min to pellet debris. DNA was extracted from the supernatant with QIaAMP mini kit (Qiagen, Venlo, The Netherlands) following the manufacturer’s protocol and resuspended in 10 mM Tris-HCl (pH 8.5). The concentration and purity of viral DNA were determined using NanoDrop (Thermo Fisher Scientific, Waltham, MA, USA).

Sequencing of viral DNA was performed using a custom library preparation protocol with inserts of ca. 255 nts and an Illumina HiSeq2500 100 nt paired-end run. After adapter trimming with Trimmomatic v0.32 [59] and quality control with FastQC (www.bioinformatics.babraham.ac.uk/projects/fastqc/ (accessed on 1 May 2020)), the paired-end reads were assembled using SOAPdenovo2 [60] with a k-mer of 91. The resulting contigs (*n* = 11,151) were aligned against the published MpDV genome with partial ITRs (NC_005040.1) using Geneious 6.1.8 (http://www.geneious.com (accessed on 1 May 2020); [61]). A consensus sequence based on 10,459 contigs was built and cut at the CCCCCCGCCCCCC and GGGGGGCGGGGGG sequences flanking the MpDV NC_005040.1 genome, and then further verified by mapping redundant reads. The 5′- and 3′-ITRs were assembled separately (as shown schematically in Figure S1a). To this end, the whole dataset of trimmed reads was aligned as single end reads to each of the two 300 nt unique sequences located downstream of the 5′-ITR and upstream of the 3′-ITR, respectively, using gsnap (version 2013-11-27; [62]) with the following parameters: -B 4 -N 0 -n 3. The second read of the pairs, for which one read was mapped to the 300 nt sequences in forward or reverse orientation, was retrieved using an in-house script and the retrieved second reads were aligned using gsnap against the MpDV internal genomic sequence lacking the two 300 nt unique sequences. The reads that did not map to the internal MpDV sequence were retained for further analysis as potentially representing ITRs. The pairs of reads anchored to each of the two 300 nt unique sequences were assembled separately using SOAPdenovo2 with k-mers of 91 and 83 for the 3′- and 5′-ITRs, respectively. To extend the SOAPdenovo2 contigs, the same pairs of reads were assembled using PRICE v1.2 [63] with k-mers of 91 and 83 for the 3′ and 5′-ITRs, respectively, and the following command: PriceTI -fp <Reads_1.fastq> <Reads_2.fastq> 265 -icf <Contig_to_extend.fasta> 1 1 5 -nc 10 -dbk <kmer> -lenf 100 1 -target 100 1 1 1. The consensus sequences of the 5′- and 3′-ITRs were the contigs that displayed the greatest depth of coverage at each extremity.

To verify the lengths of the reconstructed ITRs, the purified virion DNA was digested using PauI and SpeI, whose unique restriction sites are located downstream of the 5′-ITR and upstream of the 3′-ITR, respectively. The digestion products were separated by 1% agarose gel electrophoresis and stained with ethidium bromide (Figure S1b).

### 3.3. Aphid Feeding Conditions and Total RNA Extraction

The experiments with aphid feeding on *A. thaliana* Col-0 plants and on an artificial medium MP148, followed by total RNA extraction and Illumina stranded mRNA-seq analysis have been described by Marmonier et al. [57]. Briefly, viruliferous 10-day-old Mp-Col aphids were obtained by feeding 8-day-old aphids for 48 h on TuYV-infected *A. thaliana* plants or on the artificial medium containing 200 ng/µL of TuYV virions. The TuYV isolate TuYV-FL1 (NC_003743.1; [64]) was inoculated to *A. thaliana* by agroinoculation. To this end, *Agrobacterium tumefaciens* harboring the binary plasmid with TuYV-FL were grown to an optical density of 0.5 at 600 nm and agroinfiltrated into leaves as previously described [65], except that a needleless syringe was used. The virions were purified from TuYV-infected *Montia perfoliata* plants as described previously [66]. Control non-viruliferous Mp-Col aphids were obtained in parallel by feeding the aphids on mock-inoculated *A. thaliana* plants or virus-free artificial medium. For sRNA sequencing, 10-day-old Mp-Col aphids were obtained by feeding 2-day-old aphids for 8 days on mock-inoculated or TuYV-infected infected plants or on artificial medium without or with TuYV virions at 200 ng/µL. After feeding, pools of 30 aphids were collected for each condition in replicates and total RNA was extracted using RNeasy minikit (Qiagen, Hilden, Germany). Total RNA concentration was measured using Qubit (Thermo Fisher Scientific, USA) and RNA integrity was verified with Bioanalyser 2100 (Agilent Technologies, Santa Clara, CA, USA).

### 3.4. Illumina Sequencing of Transcriptome and sRNAome

Illumina sequencing of small RNAs and poly(A)-enriched long RNAs was performed at Fasteris (Plan-les-Ouates, Switzerland) using total RNA samples from biological replicates of the same aphid feeding experiment [57]. After a poly(A) RNA enrichment step, 12 cDNA libraries (4 conditions, 3 biological replicates per condition) were generated using Illumina TruSeq Stranded mRNA Sample Preparation Protocol. Libraries were then multiplexed and sequenced in one flowcell of Illumina HiSeq 3000/4000 as paired-end 75 nt reads, yielding ca. 44 to 66 M reads per library (APFV-5-16; Dataset S1). The raw data for 12 mRNA-seq libraries were deposited at the European Nucleotide Archive (ENA) under the accession number PRJEB46814 (www.ebi.ac.uk/ena/browser/view/PRJEB46814?show=reads (accessed on 1 October 2024)). For sRNA sequencing, 8 cDNA libraries (4 conditions, 2 biological replicates per condition) were prepared using the Illumina Small RNA gel-free protocol and then multiplexed and sequenced in one flowcell of Illumina NextSeq, yielding ca. 21 to 27 M reads per library (ALYU-368-375; Dataset S3). The raw data for the sRNA-seq libraries were deposited at the NCBI Sequence Read Archive (SRA) under BioProject ID PRJNA1153880.

The raw data of our previous Illumina mRNA-seq analysis of 18 pools of *M. persicae* WMp2 aphids fed on *A. thaliana* and *C. sativa* plants infected with TuYV, CaMV or mock-inoculated ([38]; ALYU-113-130; Dataset S5) were deposited at the ENA under the accession number ERP139639. The raw data of our previous Illumina mRNA-seq analysis of 18 pools of leaf samples from the respective *A. thaliana* and *C. sativa* plants infested by *M. persicae* WMp2 aphids ([40]; ALYU-85-102; Dataset S7) were deposited at the ENA under the number PRJEB49403. The Illumina sRNA-seq analyses of the total RNAs extracted from the same 18 pools of aphids ([38]) and two additional pools of the aphid populations, one PCR-positive and another PCR-negative for MpDV DNA (Figure S14), as well as of total RNAs extracted from the same 18 leaf samples [40], were performed using the Illumina TruSeq protocol for library preparation and multiplexing the libraries in two separates flowcells of NovaSeq, yielding ca. 29 to 56 M reads per aphid library (ALYU-169-188; Dataset S6) and ca. 22 to 45 M reads per plant library (ALYU-141-158; Dataset S8).

### 3.5. Bioinformatics Analysis of Illumina mRNA-Seq and sRNA-Seq Data

To reconstruct the MpDV transcriptome, 75 nt paired-end reads of the stranded mRNA-seq libraries were mapped to the *M. persicae* genome G006 v2.0 (downloaded at the AphidBase: https://bipaa.genouest.org/is/aphidbase/myzus_persicae/ (accessed on 1 October 2022)) using TopHat2 [67]. Unmapped reads were de novo assembled using Trinity v2.8.5 [68] and the resulting Trinity contigs were mapped to the MpDV genome. The mapped contigs were visualized (Figure S2) and further analyzed using multiple sequence alignments (File S1d).

To reconstruct *M. persicae* virome components, we used a pipeline developed previously [69]. Briefly, redundant or non-redundant sRNA-seq reads ranging from 15 to 34 nts were de novo assembled using Velvet v1.2.10 [70], followed by Oases v0.2.09 [71], with different k-mers (13, 15, 17, 19 and 21) and a minimum contig length set to 50 nts. The resulting Oases contigs were combined and mapped on the *M. persicae* nuclear genome G006 v2.0 using BWA-MEM 0.7.12 [72]. The unmapped contigs were scaffolded using SeqMan (Lasergene DNASTAR v12.0.0 Core Suit, Madison, WI, USA). The resulting SeqMan contigs were analyzed with the NCBI BLASTn and BLASTx to search for viral contigs. The reconstructed viral genome sequences were verified by BWA mapping of redundant 15–34 nts reads from the respective sRNA-seq libraries. The mapping data were then analyzed using MISIS-2 [30] and corrected manually (if needed), followed by a new round of sRNA mapping to obtain a consensus virus genome sequence and identify SNPs (if any) present in the viral quasispecies population. The MpFV genome sequence was also verified by mapping the redundant 75 nt mRNA-seq paired-end reads and their contigs assembled with Trinity.

For quantitative analysis of *M. persicae* endogenous and viral transcriptome and sRNAome, the 75 nt reads of the mRNA-seq libraries and 15–34 nt reads of the sRNA-seq libraries were mapped with or without mismatches to the reference genomes of *M. persicae* (nuclear genome G006 v2.0 and mitochondrion genome NC_029727.1), *Buchnera aphidicola* (a natural symbiont of *M. persicae*; GCF_000009605.1) and three viruses (MpDV, MpFV and TuYV) and the mapped reads were sorted and counted using in-house scripts (Datasets S1 and S3). The mapped mRNA-seq reads were sorted by polarity (forward, reverse, total), whereas the mapped sRNA-seq reads were sorted by size (15 to 35 nts, total 15–34 nt), polarity (forward, reverse, total) and 5′-nucleotide identity (5′A, 5′U, 5′G, 5′C) and for each reference genome counted in reads per million (RPM) of total (viral and host) reads in each library. In the case of MpDV, the mapping and counting were also performed separately for NS and VP parts of the MpDV genome (positions 1–2893 and 2847–5873, respectively) (Dataset S3).

Single-base resolution maps of viral mRNA-seq reads (Dataset S2) and viral sRNA-seq reads (Dataset S4), as well as SNP tables of MpDV sRNA-seq reads (Dataset S9), were generated using MISIS-2 [30] and transferred to Excel for further analysis and visualization. Nucleotide compositions of 21, 22, 23, 26, 27, 28 and 29 nt forward and reverse sRNAs derived from the NS and VP parts of MpDV genome (positions 1–2893 and 2847–5873, respectively) and from complete MpFV and TuYV genomes were determined using in-house scripts and presented as RNA logos in Figure 4b, Figure 7b, Figures S6, S10 and S12.

## Data Availability

The genome sequences of MpDV and MpFV were deposited in the NCBI GenBank under accessions PQ247848 (MpDV) and PQ247847 (MpFV). The Illumina mRNA-seq data were deposited at the European Nucleotide Archive (ENA) under the accession numbers PRJEB46814 (APFV-5-16), ERP139639 (ALYU-113-130) and PRJEB49403 (ALYU-85-102), while the sRNA-seq raw data were deposited at the NCBI Sequence Read Archive (SRA) under the BioProject ID PRJNA1153880 (ALYU-368-375; ALYU-187-188).

## Supplementary Materials

The following supporting information can be downloaded at https://www.mdpi.com/article/10.3390/ijms252313199/s1.

## References

[1] Ali J., Bayram A., Mukarram M., Zhou F., Karim M.F., Hafez M.M.A., Mahamood M., Yusuf A.A., King P.J.H., Adil M.F. (2023). Peach–Potato Aphid *Myzus persicae*: Current Management Strategies, Challenges, and Proposed Solutions. Sustainability.

[2] van Munster M., Dullemans A.M., Verbeek M., van den Heuvel J.F., Reinbold C., Brault V., Clérivet A., van der Wilk F. (2003). Characterization of a new densovirus infecting the green peach aphid *Myzus persicae*. J. Invertebr. Pathol..

[3] van Munster M., Dullemans A.M., Verbeek M., van den Heuvel J.F.J.M., Reinbold C., Brault V., Clérivet A., van der Wilk F. (2003). A new virus infecting *Myzus persicae* has a genome organization similar to the species of the genus Densovirus. J. Gen. Virol..

[4] Guo Y., Zhao Y., Yang Y., Zhang Y., Li Y., Tian H., Liu T.X., Li Z. (2024). Plants affect the horizontal transmission of a new densovirus infecting the green peach aphid *Myzus persicae* by modulating honeydew production. Insect Sci..

[5] Tang S., Song X., Xue L., Wang X., Wang X., Xu P., Ren G. (2016). Characterization and Distribution Analysis of a Densovirus Infecting Myzus persicae nicotianae (Hemiptera: Aphididae). J. Econ. Entomol..

[6] Clavijo G., van Munster M., Monsion B., Bochet N., Brault V. (2016). Transcription of densovirus endogenous sequences in the Myzus persicae genome. J. Gen. Virol..

[7] Suzuki Y., Baidaliuk A., Miesen P., Frangeul L., Crist A.B., Merkling S.H., Fontaine A., Lequime S., Moltini-Conclois I., Blanc H. (2020). Non-retroviral Endogenous Viral Element Limits Cognate Virus Replication in Aedes aegypti Ovaries. Curr. Biol..

[8] Ghildiyal M., Zamore P. (2009). Small silencing RNAs: An expanding universe. Nat. Rev. Genet..

[9] Mongelli V., Saleh M.C. (2016). Bugs Are Not to Be Silenced: Small RNA Pathways and Antiviral Responses in Insects. Annu. Rev. Virol..

[10] Borges F., Martienssen R.A. (2015). The expanding world of small RNAs in plants. Nat. Rev. Mol. Cell Biol..

[11] Pooggin M.M. (2018). Small RNA-Omics for Plant Virus Identification, Virome Reconstruction, and Antiviral Defense Characterization. Front. Microbiol..

[12] Kolliopoulou A., Santos D., Taning C.N.T., Wynant N., Vanden Broeck J., Smagghe G., Swevers L. (2019). PIWI pathway against viruses in insects. Wiley Interdiscip. Rev. RNA.

[13] Santos D., Feng M., Kolliopoulou A., Taning C.N.T., Sun J., Swevers L. (2023). What Are the Functional Roles of Piwi Proteins and piRNAs in Insects?. Insects.

[14] van Lopik J., Alizada A., Trapotsi M.A., Hannon G.J., Bornelöv S., Czech Nicholson B. (2023). Unistrand piRNA clusters are an evolutionarily conserved mechanism to suppress endogenous retroviruses across the Drosophila genus. Nat. Commun..

[15] Pitino M., Coleman A.D., Maffei M.E., Ridout C.J., Hogenhout S.A. (2011). Silencing of aphid genes by dsRNA feeding from plants. PLoS ONE.

[16] Mulot M., Monsion B., Boissinot S., Rastegar M., Meyer S., Bochet N., Brault V. (2018). Transmission of Turnip yellows virus by Myzus persicae Is Reduced by Feeding Aphids on Double-Stranded RNA Targeting the Ephrin Receptor Protein. Front. Microbiol..

[17] Webster C.G., Pichon E., van Munster M., Monsion B., Deshoux M., Gargani D., Calevro F., Jimenez J., Moreno A., Krenz B. (2018). Identification of Plant Virus Receptor Candidates in the Stylets of Their Aphid Vectors. J. Virol..

[18] Chen Y., Singh A., Kaithakottil G.G., Mathers T.C., Gravino M., Mugford S.T., van Oosterhout C., Swarbreck D., Hogenhout S.A. (2020). An aphid RNA transcript migrates systemically within plants and is a virulence factor. Proc. Natl. Acad. Sci. USA.

[19] Dong Y., Li T., Hou Y., Wilson K., Wang X., Su C., Li Y., Ren G., Xu P. (2024). Densovirus infection facilitates plant-virus transmission by an aphid. New Phytol..

[20] Feng H., Jander G. (2022). Rapid Screening of Myzus persicae (Green Peach Aphid) RNAi Targets Using Tobacco Rattle Virus. Methods Mol. Biol..

[21] Pinheiro P.V., Wilson J.R., Xu Y., Zheng Y., Rebelo A.R., Fattah-Hosseini S., Kruse A., Santos Dos Silva R., Xu Y., Kramer M. (2019). Plant viruses transmitted in two different modes produce differing effects on small RNA-mediated processes in their aphid vector. Phytobiomes J..

[22] Sattar S., Thompson G.A. (2016). Small RNA Regulators of Plant-Hemipteran Interactions: Micromanagers with Versatile Roles. Front. Plant Sci..

[23] Nigg J.C., Kuo Y.W., Falk B.W. (2020). Endogenous Viral Element-Derived Piwi-Interacting RNAs (piRNAs) Are Not Required for Production of Ping-Pong-Dependent piRNAs from Diaphorina citri Densovirus. mBio.

[24] Blevins T., Rajeswaran R., Shivaprasad P.V., Beknazariants D., Si-Ammour A., Park H.S., Vazquez F., Robertson D., Meins F., Hohn T. (2006). Four plant Dicers mediate viral small RNA biogenesis and DNA virus induced silencing. Nucleic Acids Res..

[25] Carbonell A., Carrington J.C. (2015). Antiviral roles of plant ARGONAUTES. Curr. Opin. Plant Biol..

[26] Lopez-Gomollon S., Baulcombe D.C. (2022). Roles of RNA silencing in viral and non-viral plant immunity and in the crosstalk between disease resistance systems. Nat. Rev. Mol. Cell Biol..

[27] Tijssen P., Pénzes J.J., Yu Q., Pham H.T., Bergoin M. (2016). Diversity of small, single-stranded DNA viruses of invertebrates and their chaotic evolutionary past. J. Invertebr. Pathol..

[28] Cotmore S.F., Agbandje-McKenna M., Canuti M., Chiorini J.A., Eis-Hubinger A.M., Hughes J., Mietzsch M., Modha S., Ogliastro M., Pénzes J.J. (2019). ICTV Virus Taxonomy Profile: Parvoviridae. J. Gen. Virol..

[29] Laugel M., Lecomte E., Ayuso E., Adjali O., Mével M., Penaud-Budloo M., Fonseca-Alves C.E., Payan-Carreira R. (2023). The Diversity of Parvovirus Telomeres. Recent Advances in Canine Medicine.

[30] Seguin J., Otten P., Baerlocher L., Farinelli L., Pooggin M.M. (2016). MISIS-2: A bioinformatics tool for in-depth analysis of small RNAs and representation of consensus master genome in viral quasispecies. J. Virol. Methods.

[31] Paysan-Lafosse T., Blum M., Chuguransky S., Grego T., Pinto B.L., Salazar G.A., Bileschi M.L., Bork P., Bridge A., Colwell L. (2023). InterPro in 2022. Nucleic Acids Res..

[32] Vendeville A., Ravallec M., Jousset F.X., Devise M., Mutuel D., López-Ferber M., Fournier P., Dupressoir T., Ogliastro M. (2009). Densovirus infectious pathway requires clathrin-mediated endocytosis followed by trafficking to the nucleus. J. Virol..

[33] Nigg J.C., Falk B.W. (2020). Diaphorina citri densovirus is a persistently infecting virus with a hybrid genome organization and unique transcription strategy. J. Gen. Virol..

[34] Luo Y., He P., Kanrar N., Fejes Toth K., Aravin A.A. (2023). Maternally inherited siRNAs initiate piRNA cluster formation. Mol. Cell.

[35] Teixeira M., Sela N., Ng J., Casteel C.L., Peng H.C., Bekal S., Girke T., Ghanim M., Kaloshian I. (2016). A novel virus from *Macrosiphum euphorbiae* with similarities to members of the family Flaviviridae. J. Gen. Virol..

[36] Kondo H., Fujita M., Hisano H., Hyodo K., Andika I.B., Suzuki N. (2020). Virome Analysis of Aphid Populations That Infest the Barley Field: The Discovery of Two Novel Groups of Nege/Kita-Like Viruses and Other Novel RNA Viruses. Front. Microbiol..

[37] Teixeira M.A., Sela N., Atamian H.S., Bao E., Chaudhary R., MacWilliams J., He J., Mantelin S., Girke T., Kaloshian I. (2018). Sequence analysis of the potato aphid *Macrosiphum euphorbiae* transcriptome identified two new viruses. PLoS ONE.

[38] Chesnais Q., Golyaev V., Velt A., Rustenholz C., Verdier M., Brault V., Pooggin M.M., Drucker M. (2022). Transcriptome responses of the aphid vector *Myzus persicae* are shaped by identities of the host plant and the virus. Peer Community J..

[39] Simmonds P., Becher P., Bukh J., Gould E.A., Meyers G., Monath T., Muerhoff S., Pletnev A., Rico-Hesse R., Smith D.B. (2017). ICTV Virus Taxonomy Profile: Flaviviridae. J. Gen. Virol..

[40] Chesnais Q., Golyaev V., Velt A., Rustenholz C., Brault V., Pooggin M.M., Drucker M. (2022). Comparative Plant Transcriptome Profiling of Arabidopsis thaliana Col-0 and Camelina sativa var. Celine Infested with *Myzus persicae* Aphids Acquiring Circulative and Noncirculative Viruses Reveals Virus- and Plant-Specific Alterations Relevant to Aphid Feeding Behavior and Transmission. Microbiol. Spectr..

[41] Slonchak A., Khromykh A.A. (2018). Subgenomic flaviviral RNAs: What do we know after the first decade of research. Antivir. Res..

[42] Clavel M., Lechner E., Incarbone M., Vincent T., Cognat V., Smirnova E., Lecorbeiller M., Brault V., Ziegler-Graff V., Genschik P. (2021). Atypical molecular features of RNA silencing against the phloem-restricted polerovirus TuYV. Nucleic Acids Res..

[43] Yoo B.C., Kragler F., Varkonyi-Gasic E., Haywood V., Archer-Evans S., Lee Y.M., Lough T.J., Lucas W.J. (2004). A systemic small RNA signaling system in plants. Plant Cell.

[44] Brault V., Herrbach E., Reinbold C. (2007). Electron microscopy studies on luteovirid transmission by aphids. Micron.

[45] Lewsey M.G., Hardcastle T.J., Melnyk C.W., Molnar A., Valli A., Urich M.A., Nery J.R., Baulcombe D.C., Ecker J.R. (2016). Mobile small RNAs regulate genome-wide DNA methylation. Proc. Natl. Acad. Sci. USA.

[46] Parry R., Bishop C., De Hayr L., Asgari S. (2019). Density-dependent enhanced replication of a densovirus in Wolbachia-infected Aedes cells is associated with production of piRNAs and higher virus-derived siRNAs. Virology.

[47] Mueller S., Gausson V., Vodovar N., Deddouche S., Troxler L., Perot J., Pfeffer S., Hoffmann J.A., Saleh M.C., Imler J.L. (2010). RNAi-mediated immunity provides strong protection against the negative-strand RNA vesicular stomatitis virus in Drosophila. Proc. Natl. Acad. Sci. USA.

[48] van Cleef K.W., van Mierlo J.T., Miesen P., Overheul G.J., Fros J.J., Schuster S., Marklewitz M., Pijlman G.P., Junglen S., van Rij R.P. (2014). Mosquito and Drosophila entomobirnaviruses suppress dsRNA- and siRNA-induced RNAi. Nucleic Acids Res..

[49] Bronkhorst A.W., van Cleef K.W., Vodovar N., Ince I.A., Blanc H., Vlak J.M., Saleh M.C., van Rij R.P. (2012). The DNA virus Invertebrate iridescent virus 6 is a target of the Drosophila RNAi machinery. Proc. Natl. Acad. Sci. USA.

[50] Jayachandran B., Hussain M., Asgari S. (2012). RNA interference as a cellular defense mechanism against the DNA virus baculovirus. J. Virol..

[51] Kemp C., Mueller S., Goto A., Barbier V., Paro S., Bonnay F., Dostert C., Troxler L., Hetru C., Meignin C. (2013). Broad RNA interference-mediated antiviral immunity and virus-specific inducible responses in Drosophila. J. Immunol..

[52] Karamipour N., Fathipour Y., Talebi A.A., Asgari S., Mehrabadi M. (2018). Small interfering RNA pathway contributes to antiviral immunity in *Spodoptera frugiperda* (Sf9) cells following Autographa californica multiple nucleopolyhedrovirus infection. Insect Biochem. Mol. Biol..

[53] Nishida K.M., Miyoshi K., Ogino A., Miyoshi T., Siomi H., Siomi M.C. (2013). Roles of R2D2, a cytoplasmic D2 body component, in the endogenous siRNA pathway in Drosophila. Mol. Cell.

[54] Ma M., Huang Y., Gong Z., Zhuang L., Li C., Yang H., Tong Y., Liu W., Cao W. (2011). Discovery of DNA viruses in wild-caught mosquitoes using small RNA high throughput sequencing. PLoS ONE.

[55] Qu J., Betting V., van Iterson R., Kwaschik F.M., van Rij R.P. (2023). Chromatin profiling identifies transcriptional readthrough as a conserved mechanism for piRNA biogenesis in mosquitoes. Cell Rep..

[56] Sattar S., Addo-Quaye C., Song Y., Anstead J.A., Sunkar R., Thompson G.A. (2012). Expression of Small RNA in Aphis gossypii and Its Potential Role in the Resistance Interaction with Melon. PLoS ONE.

[57] Marmonier A., Velt A., Villeroy C., Rustenholz C., Chesnais Q., Brault V. (2022). Differential gene expression in aphids following virus acquisition from plants or from an artificial medium. BMC Genom..

[58] Reinink K., Dieleman F.L., Jansen J., Montenarie A.M. (1989). Interactions between plant and aphid genotypes in resistance of lettuce to *Myzus persicae* and *Macrosiphum euphorbiae*. Euphytica.

[59] Bolger A.M., Lohse M., Usadel B. (2014). Trimmomatic: A flexible trimmer for Illumina sequence data. Bioinformatics.

[60] Luo R., Liu B., Xie Y., Li Z., Huang W., Yuan J., He G., Chen Y., Pan Q., Liu Y. (2012). SOAPdenovo2: An empirically improved memory-efficient short-read de novo assembler. Gigascience.

[61] Kearse M., Moir R., Wilson A., Stones-Havas S., Cheung M., Sturrock S., Buxton S., Cooper A., Markowitz S., Duran C. (2012). Geneious Basic: An integrated and extendable desktop software platform for the organization and analysis of sequence data. Bioinformatics.

[62] Wu T.D., Nacu S. (2010). Fast and SNP-tolerant detection of complex variants and splicing in short reads. Bioinformatics.

[63] Ruby J.G., Bellare P., Derisi J.L. (2013). PRICE: Software for the targeted assembly of components of (Meta) genomic sequence data. G3 Genes Genomes Genet..

[64] Veidt I., Lot H., Leiser M., Scheidecker D., Guilley H., Richards K., Jonard G. (1988). Nucleotide sequence of beet western yellows virus RNA. Nucleic Acids Res..

[65] Leiser R.M., Ziegler-Graff V., Reutenauer A., Herrbach E., Lemaire O., Guilley H., Richards K., Jonard G. (1992). Agroinfection as an alternative to insects for infecting plants with beet western yellows luteovirus. Proc. Natl. Acad. Sci. USA.

[66] van den Heuvel J.F., Boerma T.M., Peters D. (1991). Transmission of potato leafroll virus from plants and artificial diets by *Myzus persicae*. Phytopathology.

[67] Kim D., Pertea G., Trapnell C., Pimentel H., Kelley R., Salzberg S.L. (2013). TopHat2: Accurate alignment of transcriptomes in the presence of insertions, deletions and gene fusions. Genome Biol..

[68] Grabherr M.G., Haas B.J., Yassour M., Levin J.Z., Thompson D.A., Amit I., Adiconis X., Fan L., Raychowdhury R., Zeng Q. (2011). Full-length transcriptome assembly from RNA-Seq data without a reference genome. Nat. Biotechnol..

[69] Seguin J., Rajeswaran R., Malpica-López N., Martin R.R., Kasschau K., Dolja V.V., Otten P., Farinelli L., Pooggin M.M. (2014). De novo reconstruction of consensus master genomes of plant RNA and DNA viruses from siRNAs. PLoS ONE.

[70] Zerbino D.R., Birney E. (2008). Velvet: Algorithms for de novo short read assembly using de Bruijn graphs. Genome Res..

[71] Schulz M.H., Zerbino D.R., Vingron M., Birney E. (2012). Oases: Robust de novo RNA-seq assembly across the dynamic range of expression levels. Bioinformatics.

[72] Li H., Durbin R. (2010). Fast and Accurate Long-Read Alignment with Burrows–Wheeler Transform. Bioinformatics.

