# Supplementary material for: Deep Sequencing Analysis of Virome Components, Viral Gene Expression and Antiviral RNAi Responses in Myzus persicae Aphids
Source: Int J Mol Sci. 2024 Dec 8;25(23):13199. doi: 10.3390/ijms252313199 (PMC11642819; doi:10.3390/ijms252313199)
Supplement: Supplementary file 1 [file ijms-25-13199-s001.zip › Fig S1.pdf]

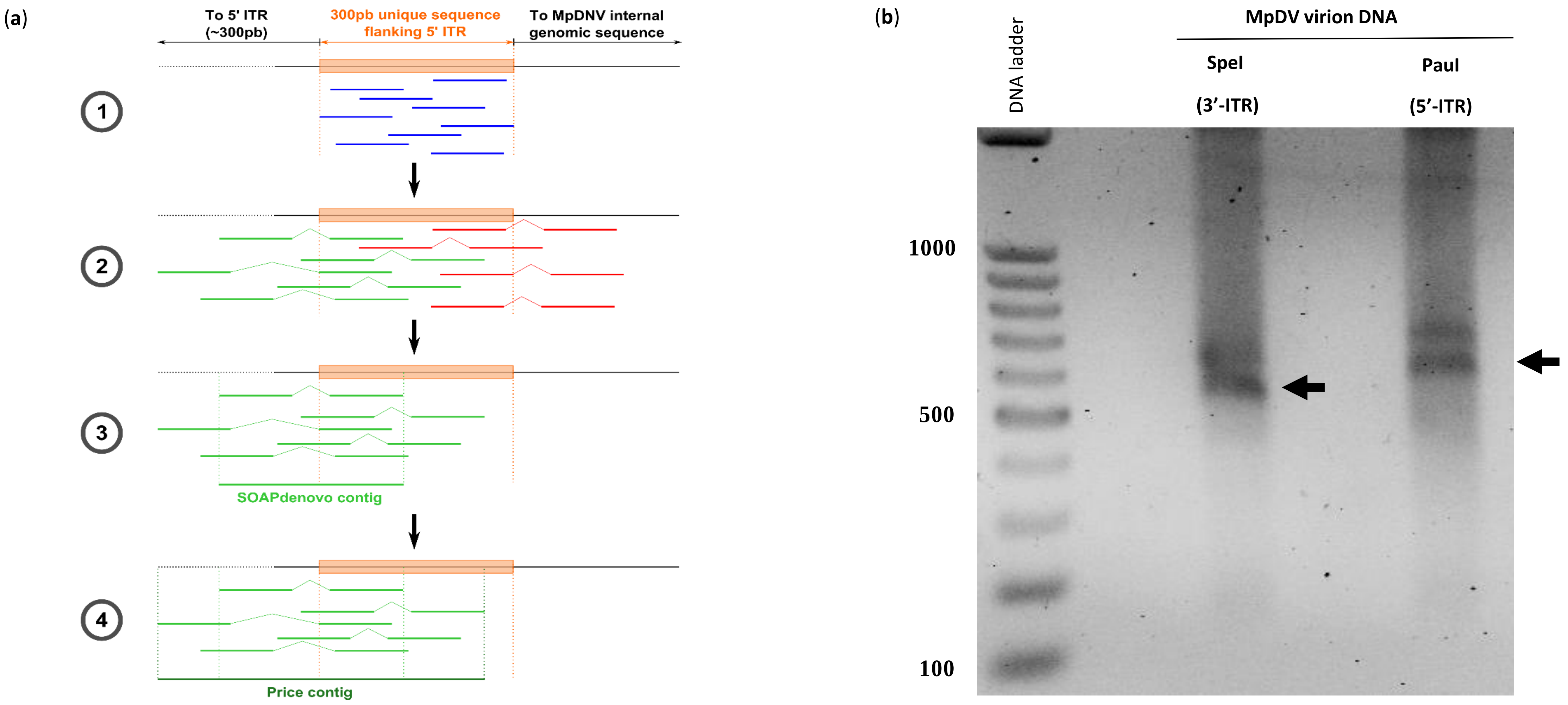

Figure S1. Illumina sequencing and restriction analysis of Myzus persicae densovirus (MpDV) inverted terminal repeats (ITRs). (a) Scheme for iterative assembly of the MpDV 5′ ITR from the Illumina paired-end reads. (1) Trimmed reads of the whole dataset were aligned as single-end reads against a 300 nt unique sequence flanking the 5′ ITR (reads in blue and 300 nt sequence in orange). (2) For the reads aligned to the 300 sequence in forward or reverse orientation, the second reads of the pairs were retrieved using an in-house script and aligned against the MpDV internal genomic sequence lacking the 300 nt unique region. Those pairs that had the second read aligned to the internal sequence (shown in red) were deleted, while the other pairs specific to the 5′ ITR (shown in green) were retained. (3) Contigs of the retained pairs of reads were assembled using SOAPdenovo2 that requires high sequencing depth. (4) The SOAPdenovo2 contigs were extended with lower coverage reads using Price. (b) Restriction analysis of MpDV virion DNA using PauI and SpeI, the unique restriction sites located downstream of the 5′-ITR and upstream of the 3′-ITR, respectively. Purified virion DNA was digested with SpeI or PauI, separated on 1% agarose gel and stained with EtBr. The digestion products of expected sizes (532 bp and 619 bp, respectively) are indicated by arrows. Note that genomic and antigenomic viral DNA molecules of homotelomeric ambisense densoviruses are encapsidated in separate virions in equal proportions and therefore purified virion DNA is linear double-stranded DNA that migrates in 1% agarose as a single band of ca. 5-6 Kbp [3]. Interestingly, besides the main digestion products of expected sizes, less abundant products of ca. 70-80 bp longer sizes are observed for SpeI and PauI digestions. These products may represent a small fraction of viral gDNA with the 5′- and 3′-ITRs elongated at their 5′- and 3′-termini, respectively, with palindromic extensions on the 314 nt and 315 nt ITR templates to create longer Y-shape telomeres (Figure 1, blue templates in the middle).
