# Supplementary material for: Deep Sequencing Analysis of Virome Components, Viral Gene Expression and Antiviral RNAi Responses in Myzus persicae Aphids
Source: Int J Mol Sci. 2024 Dec 8;25(23):13199. doi: 10.3390/ijms252313199 (PMC11642819; doi:10.3390/ijms252313199)

Figure S2. **Trinity assembly of Myzus persicae densovirus (MpDV) transcripts using Illumina stranded mRNA-seq 75 nt paired-end reads from *M. persicae* aphids**. Illumina stranded mRNA-seq reads from 12 samples of *M. persicae* aphids fed on mock-inoculated or TuYV-infected plants (Plant mock APFV-5-7, Plant TuYV APFV-14-16) or artificial diets without or with TuYV virions (ArtDiet mock APFV-8-10, ArtDiet TuYV APFV-11-13) were mapped the *M. persicae* reference genome using TopHat2 and the unmapped reads were de novo assembled using Trinity. The resulting Trinity contigs were mapped to the MpDV reference genome and the mapping results for each of the three biological replicates per aphid condition are presented.

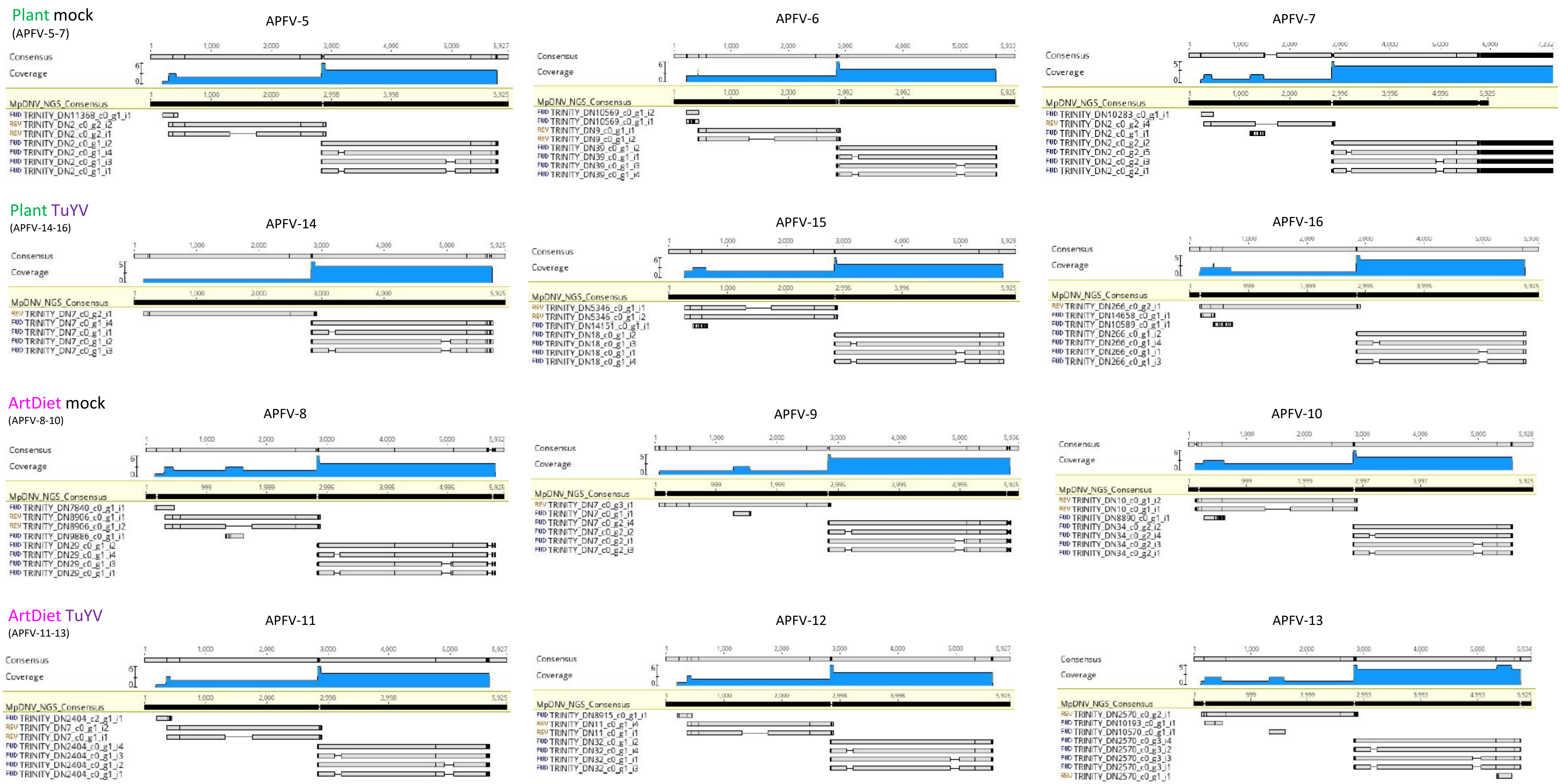

Supplement: Supplementary file 1 [file ijms-25-13199-s001.zip › Fig S2.pdf]
