# Supplementary material for: Deep Sequencing Analysis of Virome Components, Viral Gene Expression and Antiviral RNAi Responses in Myzus persicae Aphids
Source: Int J Mol Sci. 2024 Dec 8;25(23):13199. doi: 10.3390/ijms252313199 (PMC11642819; doi:10.3390/ijms252313199)

Figure S3. Single-base resolution maps of Illumina stranded mRNA-seq reads representing rightward and leftward readthrough transcripts of Myzus persicae densovirus (MpDV) and transcripts initiated within inverted terminal repeats (ITRs) of MpDV. (a) Genome and transcriptome organization of MpDV. Viral genomic (forward and reverse) ssDNA molecules are depicted as black lines, with positions of the ITRs indicated with gray boxes and the major and minor transcription start sites (TSS) of the rightward/forward strand non-structural (NS) and leftward/reverse strand virion protein (VP) genes shown with bent blue and red arrows, respectively. Viral mRNAs transcribed from the NS and VP units are depicted with blue and red solid lines, respectively, with positions of main ORFs (NS1, NS2, VP1, VP2) and introns indicated on each mRNA. Dotted lines depict rightward and leftward readthrough transcripts and transcripts initiated within ITRs. (b) Single-base resolution map of Illumina stranded mRNA-seq 75 nt reads representing MpDV rightward and leftward readthrough transcripts and ITR transcripts. The mRNA-seq reads from M. persicae aphids fed on plants or artificial diets were mapped to the MpDV reference sequence and the mapping data were analyzed using MISIS-2 [30] and visualized using Excel (Dataset S2a). The map of combined reads from all biological replicates at all four feeding conditions is presented as histogram that plots the numbers of 75 nt reads at each nucleotide position of the 5873 nt MpDV genome: blue bars above the axis represent forward reads starting at each respective position, while red bars below the axis represent reverse reads ending at the respective position. Forward reads representing the rightward NS unit mRNAs and reverse reads representing the leftward VP unit mRNAs transcribed from the major start sites located downstream of the 5-ITR (NS forward reads) and the 3'-ITR (VP reverse reads) are masked. Vertical dotted lines indicate positions of the major transcription start sites of the NS and VP units. Blue and red arrows indicate orientation and length of the forward and reverse reads, respectively.

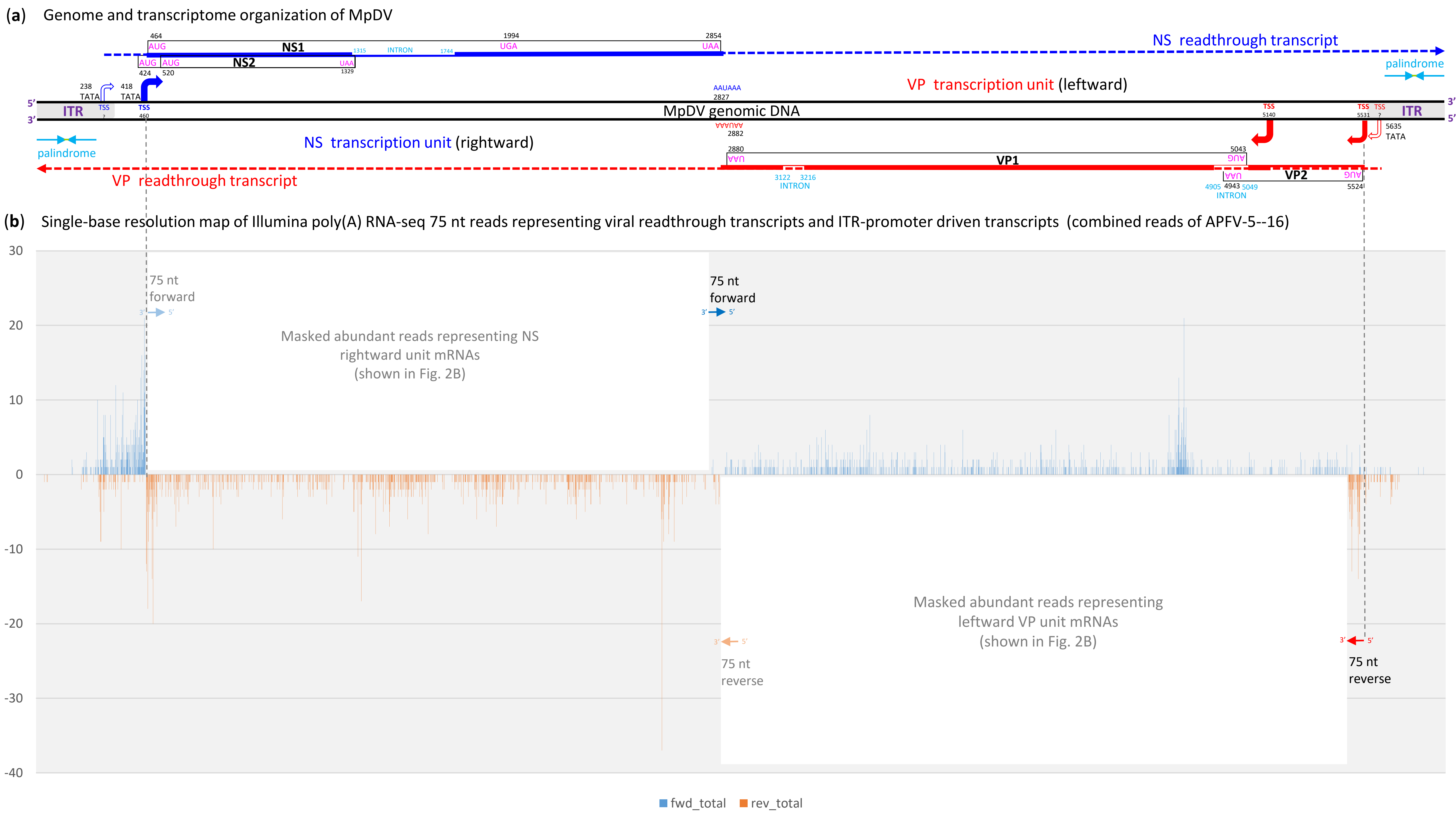

Supplement: Supplementary file 1 [file ijms-25-13199-s001.zip › Fig S3.pdf]
