# Supplementary material for: Deep Sequencing Analysis of Virome Components, Viral Gene Expression and Antiviral RNAi Responses in Myzus persicae Aphids
Source: Int J Mol Sci. 2024 Dec 8;25(23):13199. doi: 10.3390/ijms252313199 (PMC11642819; doi:10.3390/ijms252313199)
Supplement: Supplementary file 1 [file ijms-25-13199-s001.zip › Fig S4.pdf]

Figure S4. Size profiles and relative abundance of Myzus persicae densovirus (MpDV)-derived and endogenous aphid sRNAs from *M. persicae* aphids at different feeding conditions and levels of Myzus persicae flavivirus (MpFV)-derived sRNAs in aphids positive or negative for MpDV. Illumina sRNA-seq 13-34 nt reads from *M. persicae* aphids fed on artificial diet (ArtDiet) without or with turnip yellows virus (TuYV) virions or on mock-inoculated or TuYV infected *A. thaliana* plants, or those from *M. persicae* aphids positive or negative for MpDV and fed on virus-free sweet pepper plants, were mapped to the reference sequences of *M. persicae* G006 v2.0 genome and viral (MpDV and MpFV) genomes and the mapped reads were sorted by size and polarity (forward, reverse, total) and counted in reads per million (RPV) of total (host and viral) 15-34 nt reads (Dataset S3). Relative abundances of each size-class of MpDV-derived and endogenous aphid sRNA reads are presented bar graphs plotting counts in RPM of total, forward and reverse reads. (a) Size profiles and relative abundances of MpDV sRNAs at plant and ArtDiet feeding conditions. (b) Size profiles and relative abundance of endogenous aphid sRNAs at plant and ArtDiet feeding conditions. (c) Size profiles and relative abundance endogenous aphid sRNAs in aphids negative or positive for MpDV and sRNA size profile MpDV-derived sRNAs in MpDV-positive aphids fed on sweet pepper plants. (d) Relative abundance of total 15-34 nt sRNAs derived from MpDV and MpFV in aphids positive for MpDV (ALYU-188) or aphids negative for MpDV (ALYU-187).

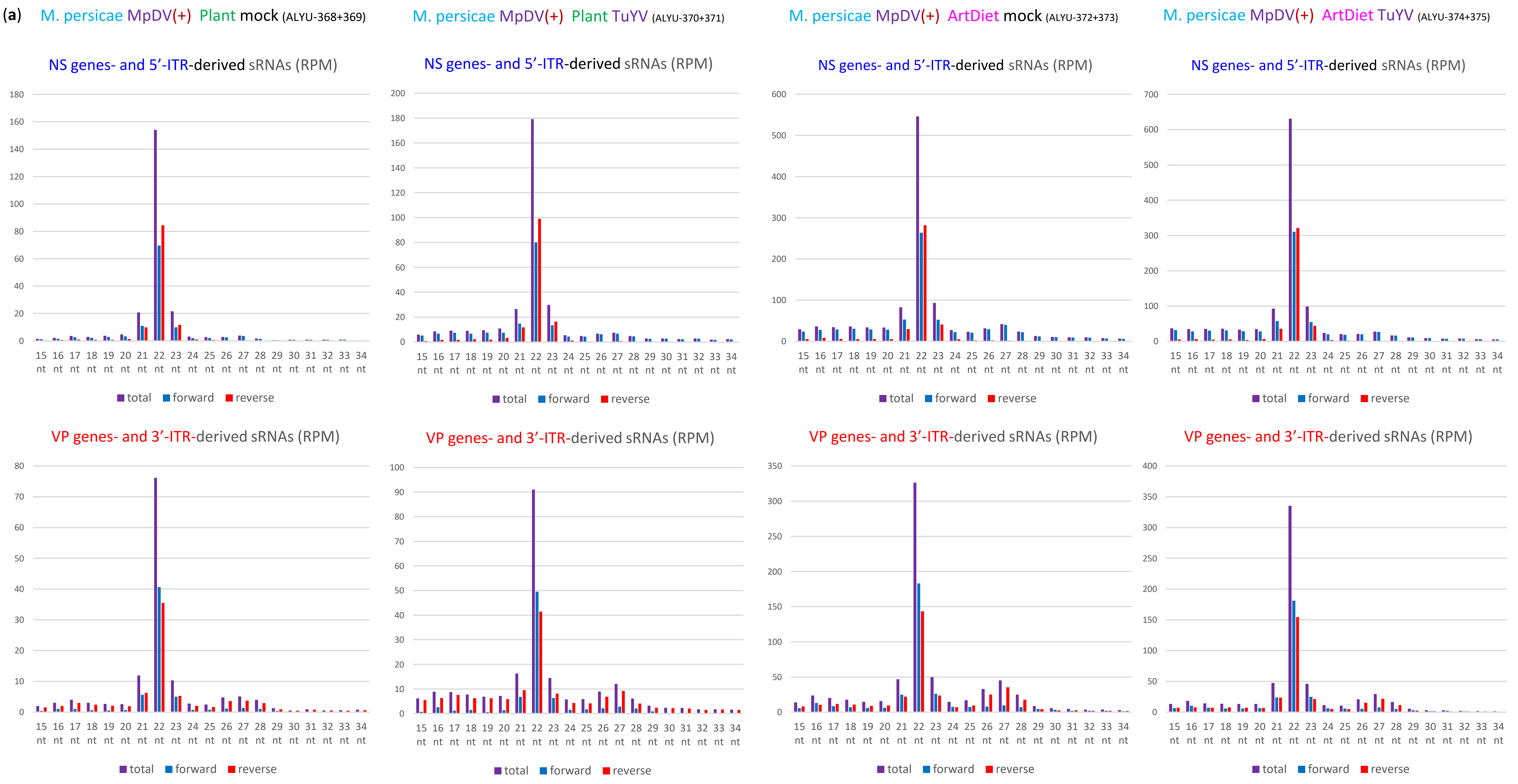

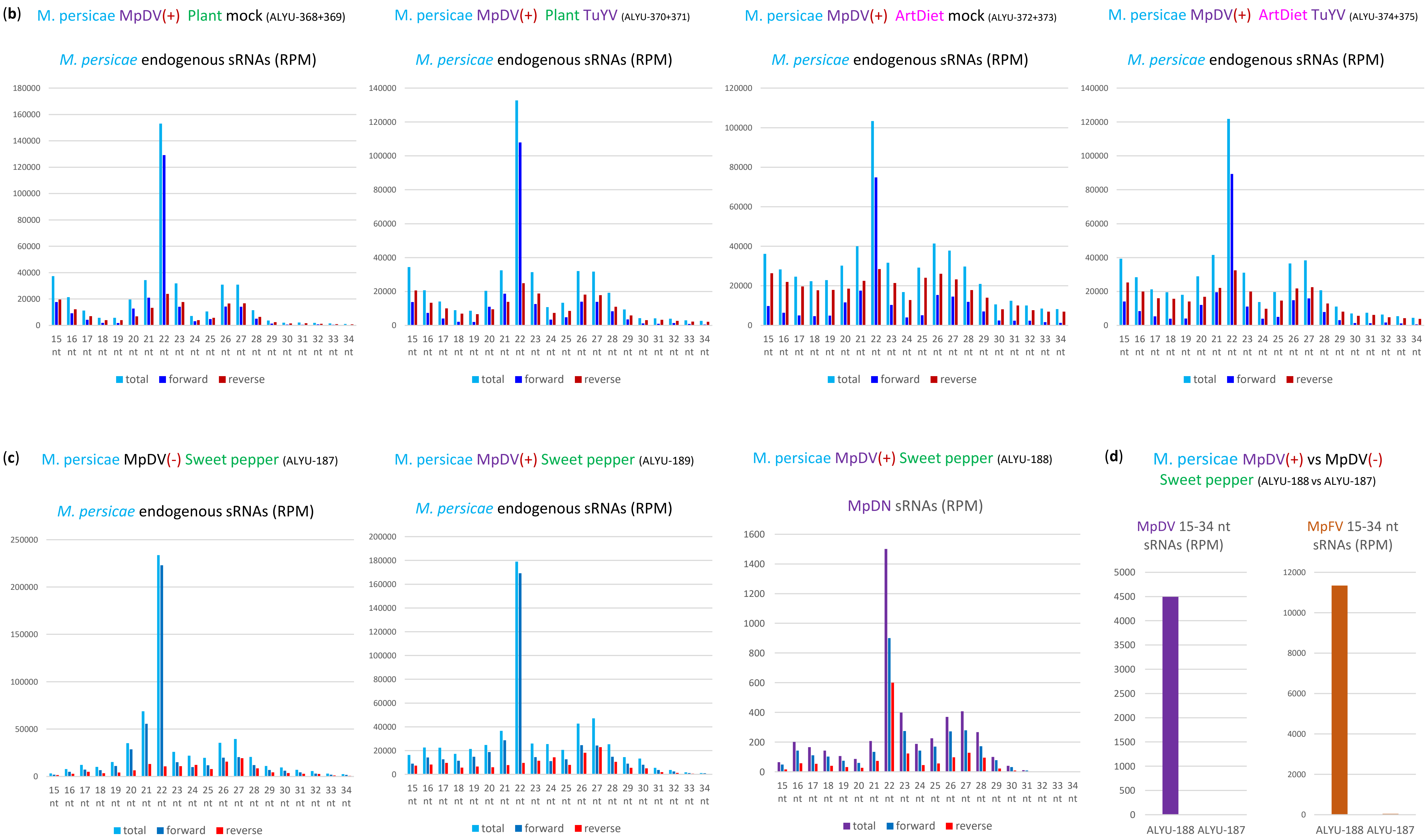
(b)
M. persicae MpDV(+) Plant mock (ALYU-368+369)
M. persicae endogenous sRNAs (RPM)
M. persicae MpDV(+) Plant TuYV (ALYU-370+371)
M. persicae endogenous sRNAs (RPM)
M. persicae MpDV(+) ArtDiet mock (ALYU-372+373)
M. persicae endogenous sRNAs (RPM)
M. persicae MpDV(+) ArtDiet TuYV (ALYU-374+375)
M. persicae endogenous sRNAs (RPM)
15 nt 16 nt 17 nt 18 nt 19 nt 20 nt 21 nt 22 nt 23 nt 24 nt 25 nt 26 nt 27 nt 28 nt 29 nt 30 nt 31 nt 32 nt 33 nt 34 nt
total forward reverse
(c)
M. persicae MpDV(-) Sweet pepper (ALYU-187)
M. persicae endogenous sRNAs (RPM)
M. persicae MpDV(+) Sweet pepper (ALYU-189)
M. persicae endogenous sRNAs (RPM)
M. persicae MpDV(+) Sweet pepper (ALYU-188)
MpDN sRNAs (RPM)
(d)
M. persicae MpDV(+) vs MpDV(-)
Sweet pepper (ALYU-188 vs ALYU-187)
MpDV 15-34 nt sRNAs (RPM)
MpFV 15-34 nt sRNAs (RPM)
ALYU-188 ALYU-187
