# Supplementary material for: Deep Sequencing Analysis of Virome Components, Viral Gene Expression and Antiviral RNAi Responses in Myzus persicae Aphids
Source: Int J Mol Sci. 2024 Dec 8;25(23):13199. doi: 10.3390/ijms252313199 (PMC11642819; doi:10.3390/ijms252313199)

Figure S5. Single-base resolution maps of Myzus persicae densovirus (MpDV)-derived 21-23 nt and 26-28 nt sRNAs accumulating in *M. persicae* aphids fed on mock-inoculated or turnip yellows virus (TuYV)-infected *A. thaliana* plants or on artificial diets (ArtDiet) without (mock) or with purified TuYV virions. The Illumina sRNA-seq reads from *M. persicae* aphids fed on plants or artificial diets (samples ALYU-368-374) were mapped to the MpDV reference sequence and the mapping data were analyzed using MISIS-2 [30] and visualized using Excel (Dataset S4a). The maps of combined reads from two biological replicates at each of the four feeding conditions are presented as histograms that plot the numbers of combined 21-23 nt reads and combined 26-28 nt reads at each nucleotide position of the 5873 nt MpDV genome: blue bars above the axis represent forward reads starting at each respective position, while red bars below the axis represent reverse reads ending at the respective position.

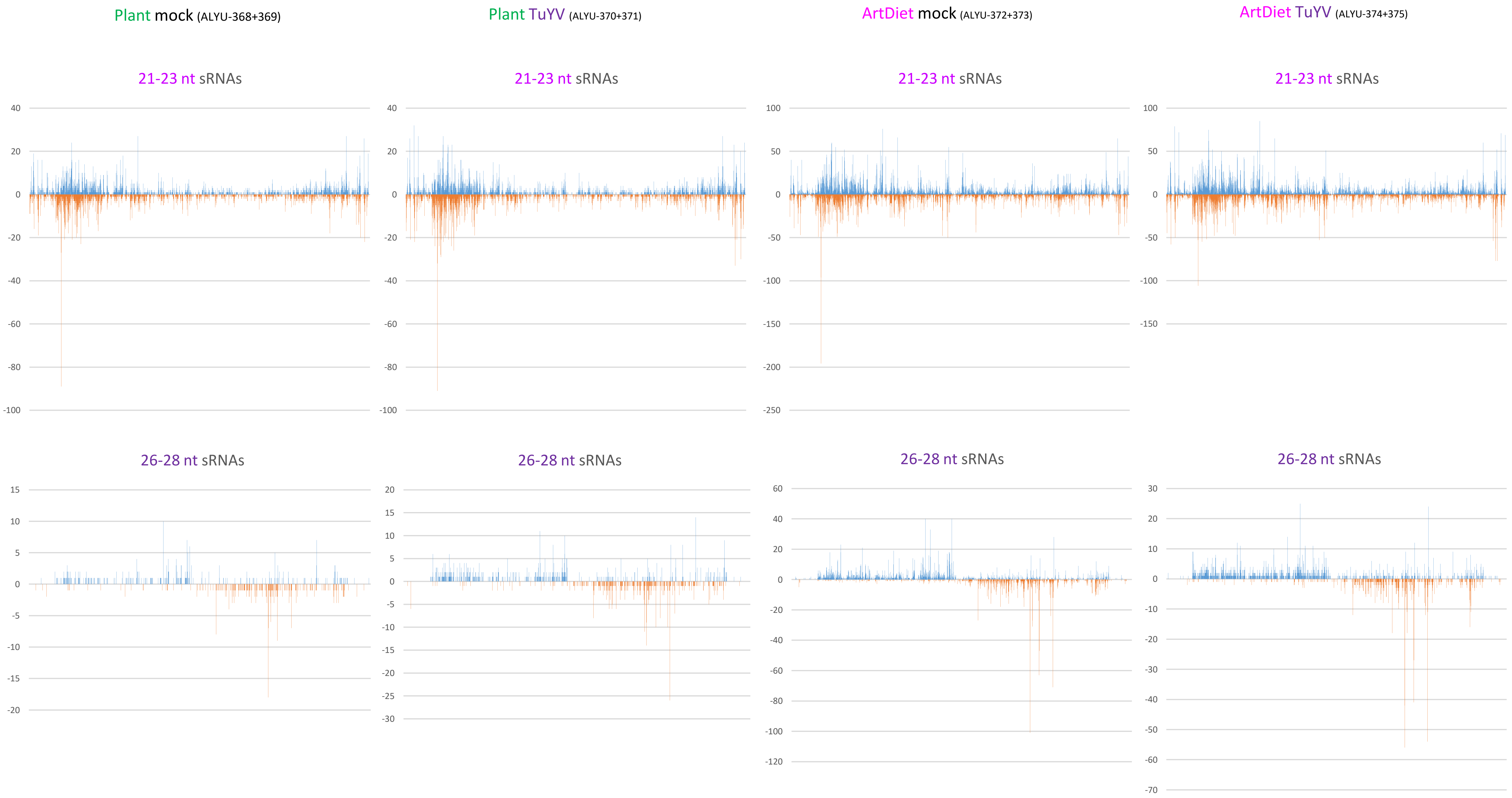

Supplement: Supplementary file 1 [file ijms-25-13199-s001.zip › Fig S5.pdf]
