# Supplementary material for: Deep Sequencing Analysis of Virome Components, Viral Gene Expression and Antiviral RNAi Responses in Myzus persicae Aphids
Source: Int J Mol Sci. 2024 Dec 8;25(23):13199. doi: 10.3390/ijms252313199 (PMC11642819; doi:10.3390/ijms252313199)

Figure S6. Nucleotide compositions of Myzus persicae densovirus (MpDV)-derived 21-23 nt and 26-28 nt sRNAs accumulating in *M. persicae* aphids fed on mock-inoculated or turnip yellows virus (TuYV)-infected *A. thaliana* plants or on artificial diets (ArtDiet) without (mock) or with purified TuYV virions. The Illumina sRNA-seq reads from *M. persicae* aphids fed on plants or artificial diets (samples ALYU-368-374) were mapped to the reference sequence of NS and VP parts of the MpDV genome (positions 1-2893 and 2847-5873, respectively) and the mapped reads were sorted by size and polarity (forward, reverse) and then counted (Dataset S3). For each size class and polarity, nucleotide compositions of combined reads from two biological replicates at each of the four feeding conditions are presented as RNA logos. Nucleotide positions of 1 and 10 of the 26, 27 and 28 nt reads with predominant uridine (1U) and adenosine (10A), respectively, are indicated.

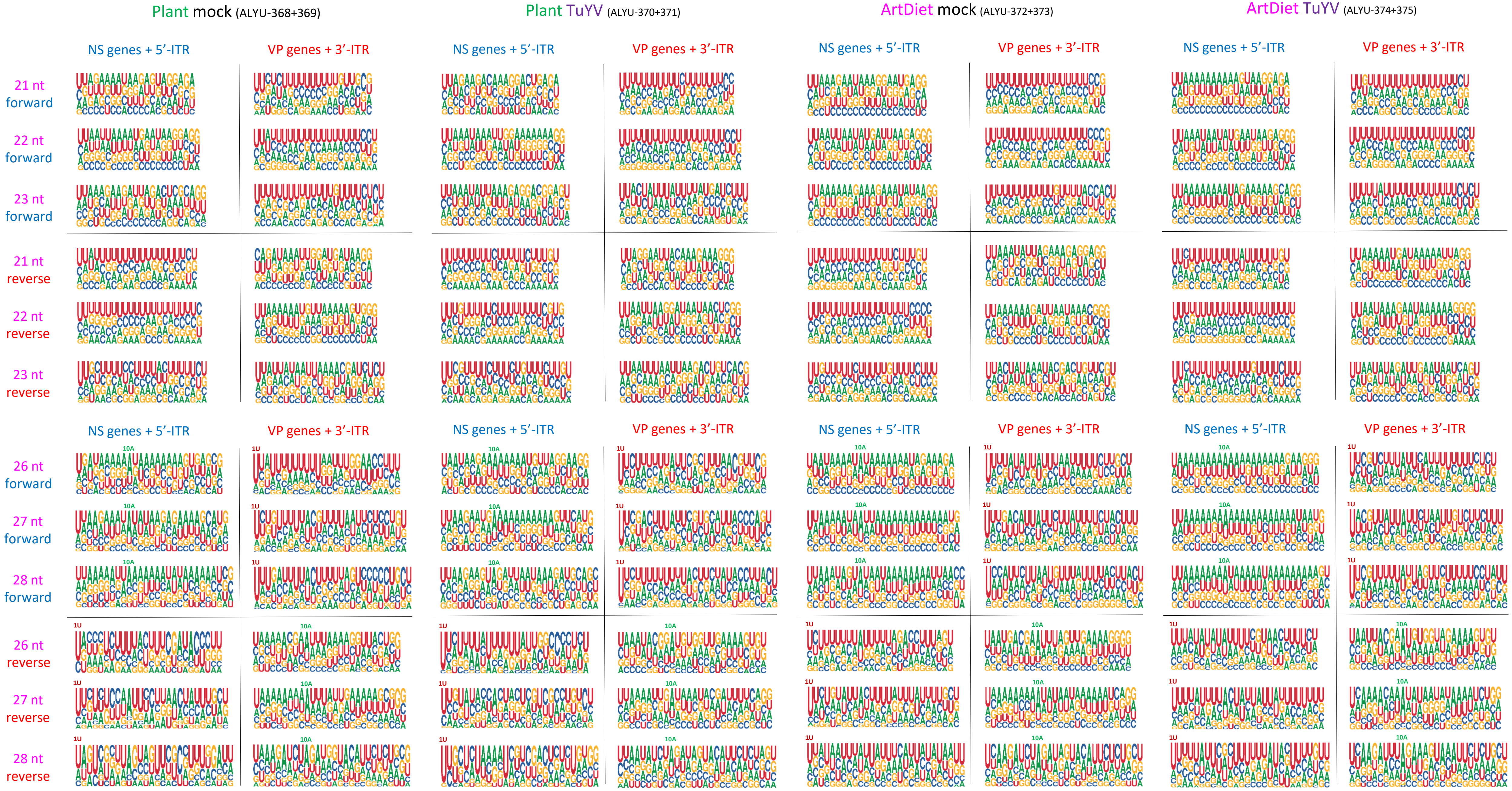

Supplement: Supplementary file 1 [file ijms-25-13199-s001.zip › Fig S6.pdf]
