# Supplementary material for: Deep Sequencing Analysis of Virome Components, Viral Gene Expression and Antiviral RNAi Responses in Myzus persicae Aphids
Source: Int J Mol Sci. 2024 Dec 8;25(23):13199. doi: 10.3390/ijms252313199 (PMC11642819; doi:10.3390/ijms252313199)

Figure S7. Functional domains/motifs and structural elements in the polyproteins encoded by Myzus persicae flavivirus (MpFV) (a), Macrosiphum euphorbiae virus 1 (MeV-1) (b) and Sitobion miscanthi flavi-like virus 1 (SnFLV-1) (c). Single large polyproteins encoded by MpFV, MeV-1 and SnFLV-1 were analyzed using InterPro at the Webserver https://www.ebi.ac.uk/interpro/ and the results are presented in panels A, B and C, respectively, as images exported from the Webserver.

(a) MpFV polyprotein

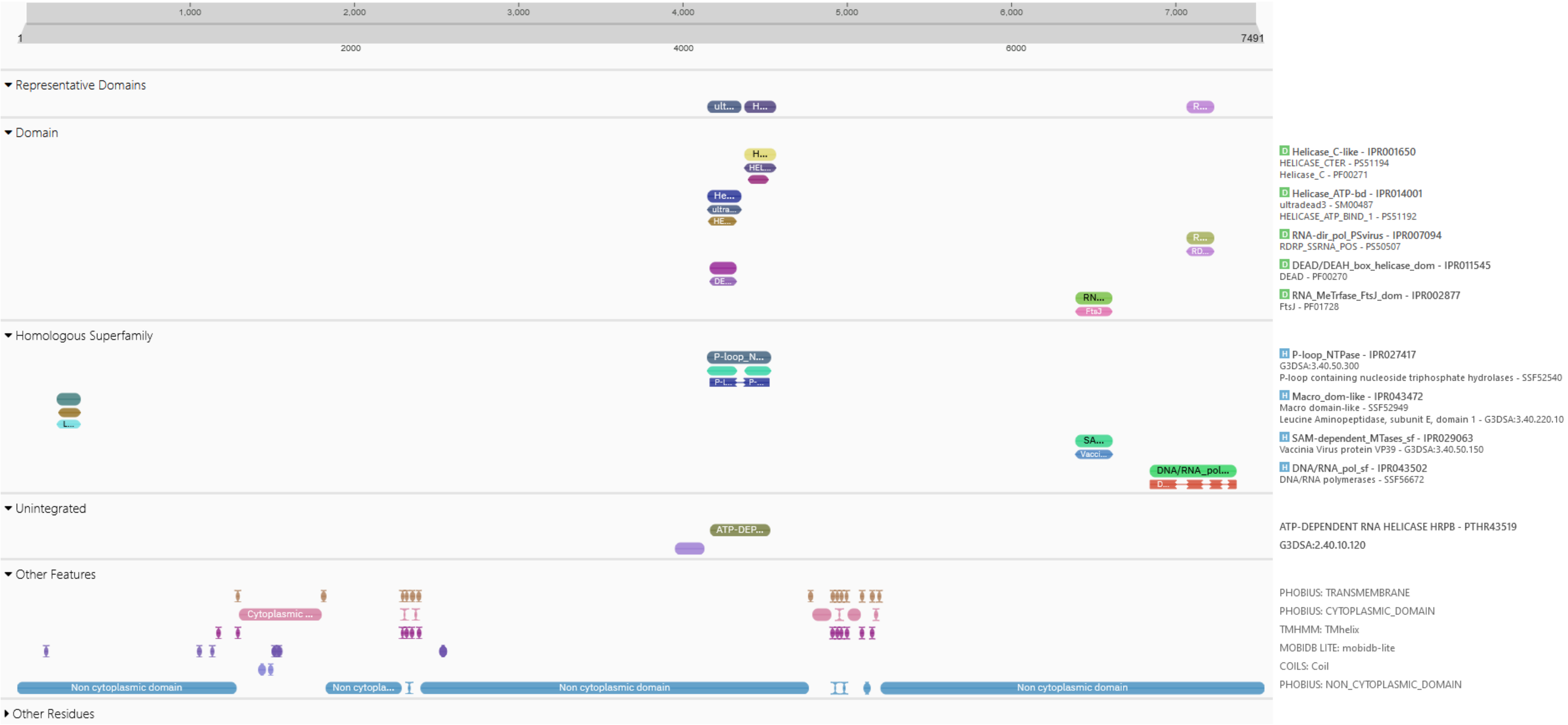

(**b**) MeV-1 polyprotein

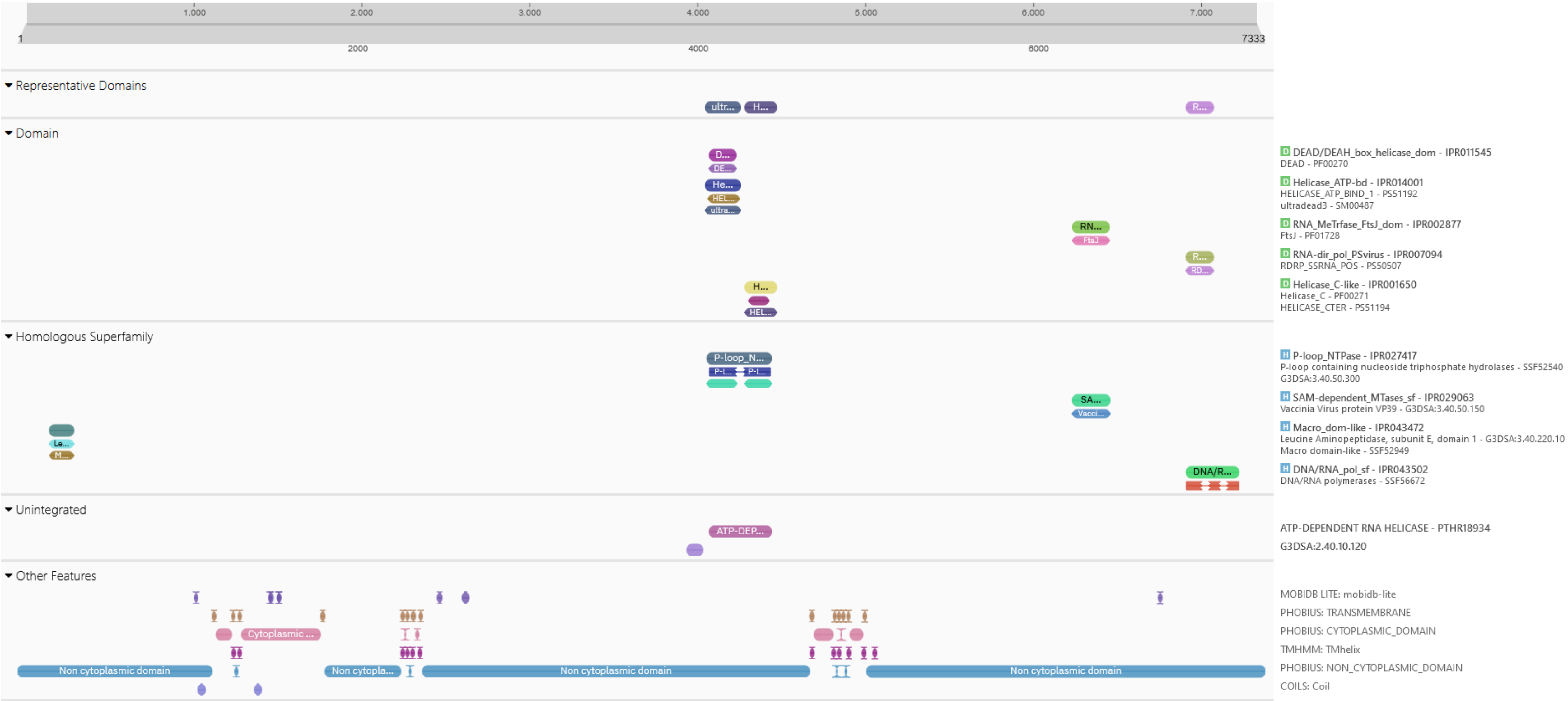

(c) SnFLV-1 polyprotein

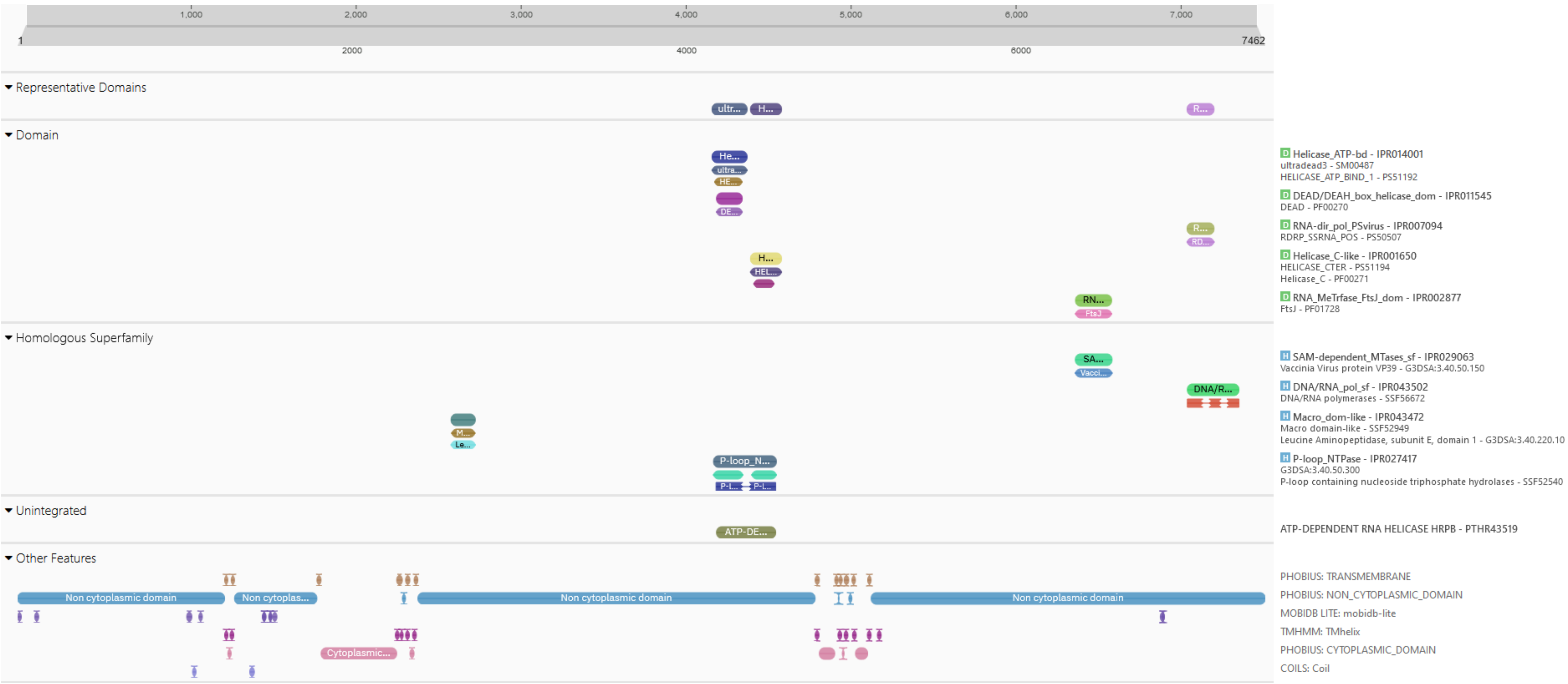

Supplement: Supplementary file 1 [file ijms-25-13199-s001.zip › Fig S7.pdf]
