# Supplementary material for: Deep Sequencing Analysis of Virome Components, Viral Gene Expression and Antiviral RNAi Responses in Myzus persicae Aphids
Source: Int J Mol Sci. 2024 Dec 8;25(23):13199. doi: 10.3390/ijms252313199 (PMC11642819; doi:10.3390/ijms252313199)
Supplement: Supplementary file 1 [file ijms-25-13199-s001.zip › Fig S8.pdf]

Figure S8. Secondary structure of the genomic RNA 5'- and 3'-untranslated regions (UTRs) of Myzus persicae flavivirus (MpFV), Macrosiphum euphorbiae virus 1 (MeV-1) and Sitobion miscanthi flavi-like virus 1 (SnFLV-1). Secondary structures of the 5'-UTRs (a) and 3'-UTRs (b) of MpFV, MeV-1 and SnFLV-1 genomic RNAs determined using RNA parameters of Turner model 2004 at 30ºC at Webserver http://rna.tbi.univie.ac.at/cgi-bin/RNAWebSuite/RNAfold.cgi, are presented as images exported from the Webserver. Positions of 5'- and 3'-termini of the UTR sequences and the AUG start codons at the 5'-UTR ends are indicated. Color code indicates base pair probabilities ranging from 0 (blue) to 1 (red).

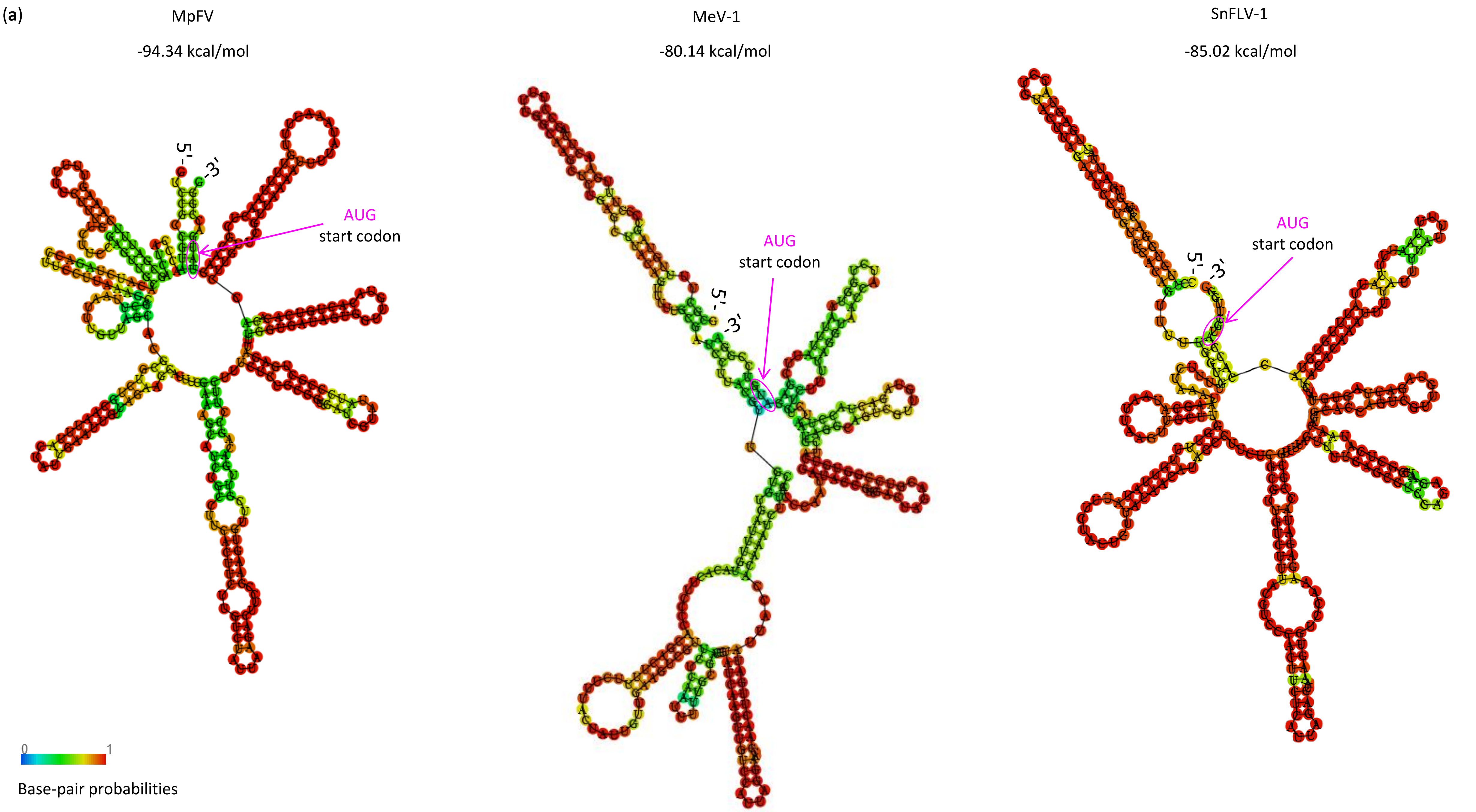

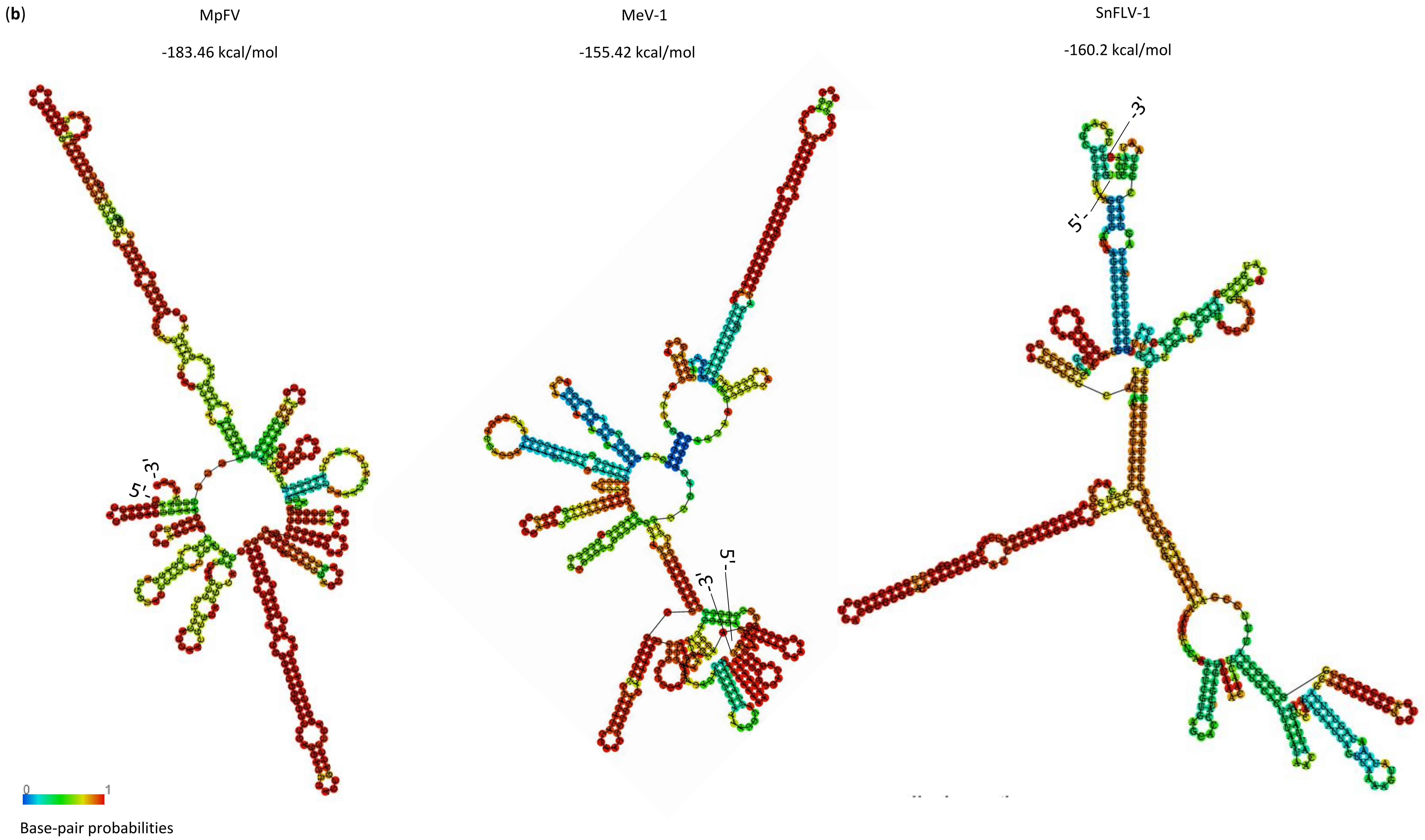
(b)
MpFV
-183.46 kcal/mol
MeV-1
-155.42 kcal/mol
SnFLV-1
-160.2 kcal/mol
5'-
-3'
0
1
Base-pair probabilities
