# Supplementary material for: Deep Sequencing Analysis of Virome Components, Viral Gene Expression and Antiviral RNAi Responses in Myzus persicae Aphids
Source: Int J Mol Sci. 2024 Dec 8;25(23):13199. doi: 10.3390/ijms252313199 (PMC11642819; doi:10.3390/ijms252313199)

Figure S9. Myzus persicae flavivirus (MpFV) transcriptome and sRNAome. (a) Single-base resolution maps of Illumina stranded mRNA-seq 75 nt reads representing viral genomic (blue) and antigenomic (red) RNAs. (b) Single-base resolution maps of Illumina sRNA-seq 22 nt reads representing viral sense (blue) and antisense (red) siRNAs. In each panel, MpFV genome and transcriptome organization is shown above the maps. Viral genomic (g) and antigenomic (ag) RNAs are depicted as respectively blue and red solid lines. Putative subgenomic (sg) RNA(s) generated by host 5'-3' exonuclease-mediated partial degradation of gRNA is depicted as dotted blue line. Viral RdRP activity generating agRNA on the gRNA template and vice versa are indicated by black arrows. To obtain single-base resolution maps, the mRNA-seq and sRNA-seq reads from *M. persicae* aphids fed on plants or artificial diets were mapped to the MpFV reference genome and the mapping data were analyzed using MISIS-2 [30] and visualized using Excel (Datasets S2b and S4b). The maps of combined reads from three (mRNA-seq) or two (sRNA-seq) biological replicates at the four feeding conditions (Plant mock, Plant TuYV, ArtDiet mock, ArtDiet TuYV) are presented as histograms that plot the numbers of 75 nt mRNA-seq (A) and 22 nt sRNA-seq (B) reads at each nucleotide position of the 23,221 nt MpFV genome: blue bars above the axis represent forward reads starting at each respective position, while red bars below the axis represent reverse reads ending at the respective position. In panel B, bars exceeding the values of 600 (red) and 400 (blue) unique sRNA reads are cut off and the numbers of respective reads are indicated. Complete histograms are shown in S4B Dataset.

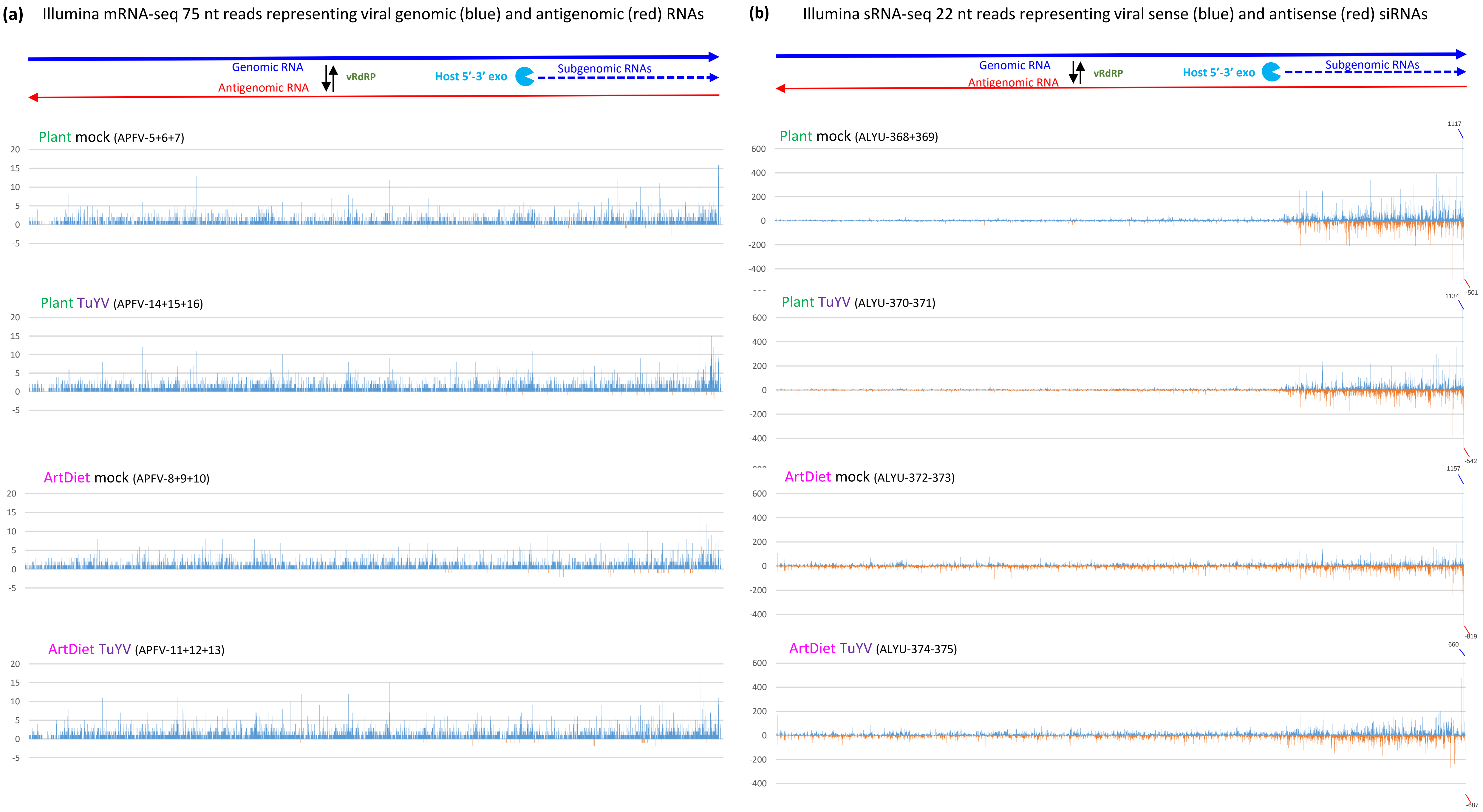

Supplement: Supplementary file 1 [file ijms-25-13199-s001.zip › Fig S9.pdf]
