# Supplementary material for: Deep Sequencing Analysis of Virome Components, Viral Gene Expression and Antiviral RNAi Responses in Myzus persicae Aphids
Source: Int J Mol Sci. 2024 Dec 8;25(23):13199. doi: 10.3390/ijms252313199 (PMC11642819; doi:10.3390/ijms252313199)

Figure S10. Size profiles and nucleotide compositions of Myzus persicae flavivirus (MpFV)-derived small (s)RNAs in *M. persicae* aphids at different feeding conditions. Illumina sRNA-seq 15-34 nt reads from *M. persicae* aphids fed on plants or artificial diets were mapped with zero mismatches to the reference sequence of the 23,221 nt MpFV genome and the mapped reads were sorted by size and polarity (forward, reverse, total) and counted in reads per million (RPM) of total (host and viral) 15-34 nt reads (Dataset S3). (a) Counting results for combined reads from two biological replicates at each of the four feeding conditions (Plant mock ALYU-368+369, Plant TuYV ALYU-370+371, ArtDiet mock ALYU-372+373, ArtDiet TuYV ALYU-374+375) are shown as bar graphs representing counts in RPM of each size-class of MpFV-derived sRNAs. (b) Nucleotide compositions of 21, 22, 23, 26, 27, 28 nt forward and reverse sRNAs derived from MpFV at the four feeding conditions are presented as RNA logos.

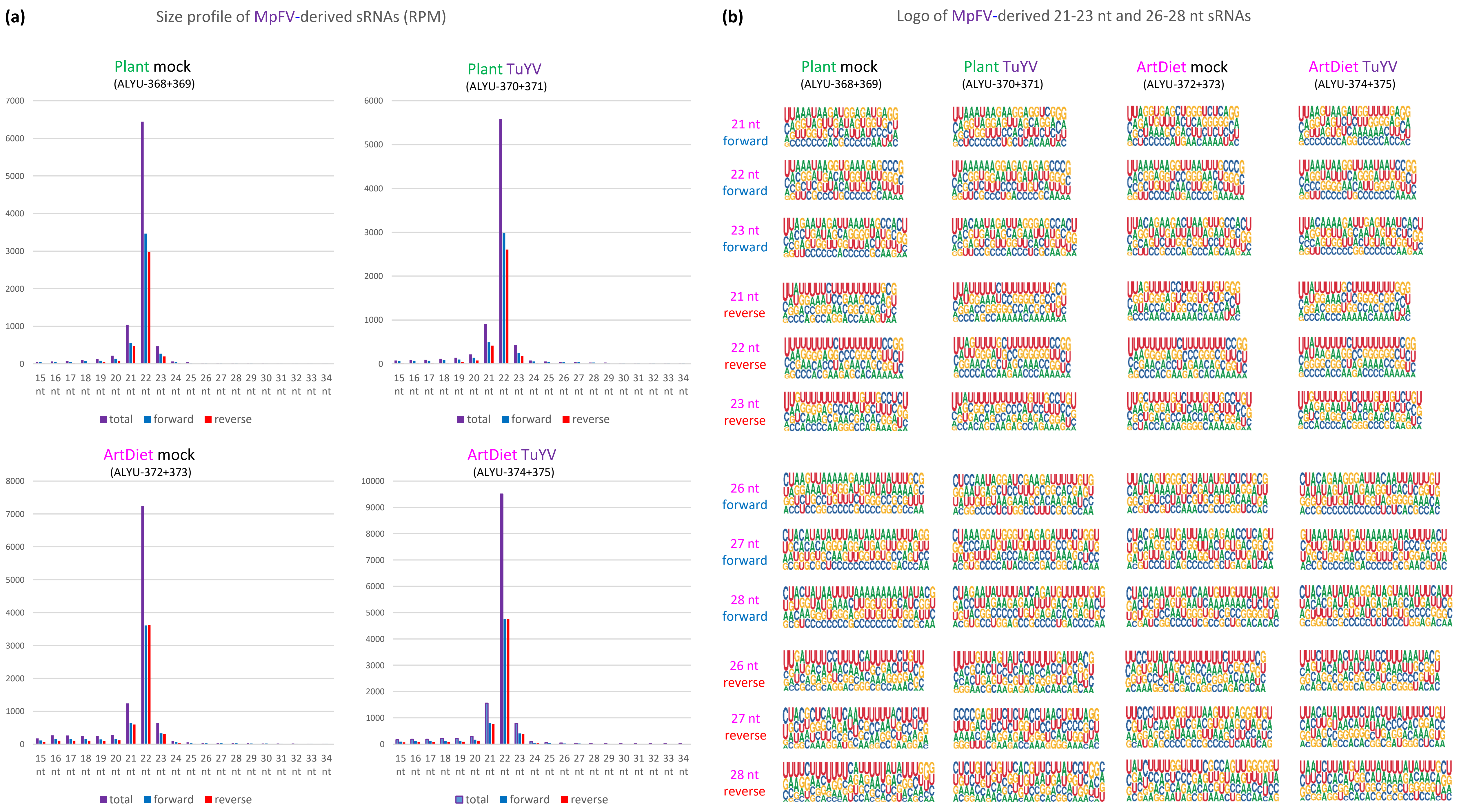

Supplement: Supplementary file 1 [file ijms-25-13199-s001.zip › Fig S10.pdf]
