# Supplementary material for: Deep Sequencing Analysis of Virome Components, Viral Gene Expression and Antiviral RNAi Responses in Myzus persicae Aphids
Source: Int J Mol Sci. 2024 Dec 8;25(23):13199. doi: 10.3390/ijms252313199 (PMC11642819; doi:10.3390/ijms252313199)

Figure S11. Myzus persicae flavivirus (MpFV) genomic (g)RNA secondary structures upstream and downstream of a predicted 5'-end of subgenomic (sg)RNA. Secondary structures of the gRNA sequences surrounding the 5'-end of sgRNA determined using Turner model 2004 at 30ºC at the Webserver http://rna.tbi.univie.ac.at/cgi-bin/RNAWebSuite/RNAfold.cgi are presented as images exported from the Webserver. Positions of 5'- and 3'-termini of the folded gRNA sequences are indicated and the sgRNA 5'-terminal nucleotide is circled. The two most stable hairpins located downstream of the sgRNA 5'-end are indicated with pink ovals. Color code indicates base pair probabilities ranging from 0 (blue) to 1 (red).

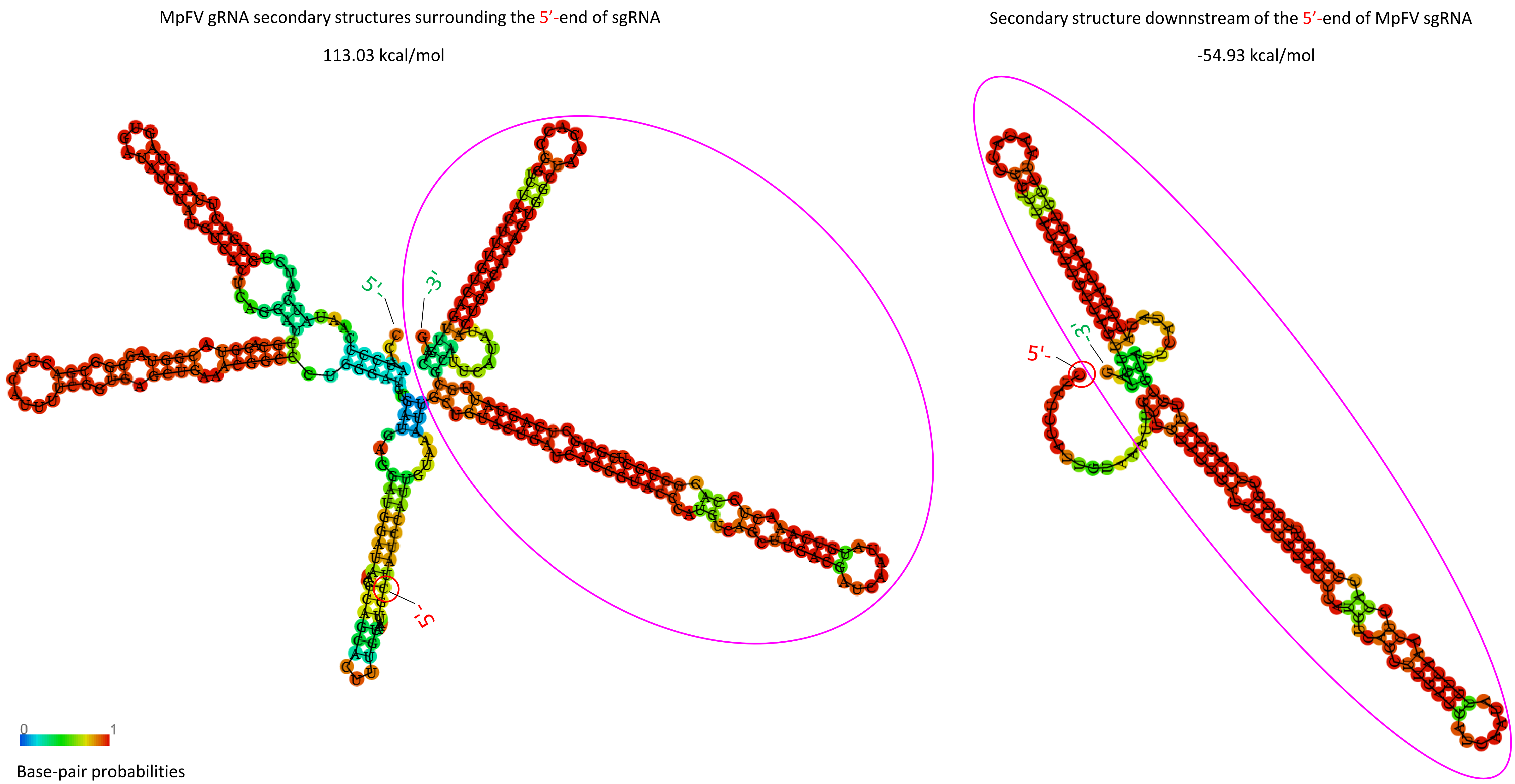

Supplement: Supplementary file 1 [file ijms-25-13199-s001.zip › Fig S11.pdf]
