# Supplementary material for: Deep Sequencing Analysis of Virome Components, Viral Gene Expression and Antiviral RNAi Responses in Myzus persicae Aphids
Source: Int J Mol Sci. 2024 Dec 8;25(23):13199. doi: 10.3390/ijms252313199 (PMC11642819; doi:10.3390/ijms252313199)

Figure S12. Nucleotide compositions of turnip yellows virus (TuYV)-derived 21-23 nt (a) and 26-28 nt (b) small (s)RNAs accumulating in *M. persicae* aphids fed on TuYV-infected *A. thaliana* plants or artificial diet with purified TuYV virions. Illumina sRNA-seq 15-34 nt reads from *M. persicae* aphids fed on plants or artificial diets were mapped with zero mismatches to the TuYV reference genome of the mapped reads were sorted by size and polarity (forward, reverse) and counted (Dataset S3). For each size-class and polarity, nucleotide compositions of combined reads from two biological replicates at each of the four feeding conditions were determined are presented as RNA logos with numbers of reads indicated below each logo.

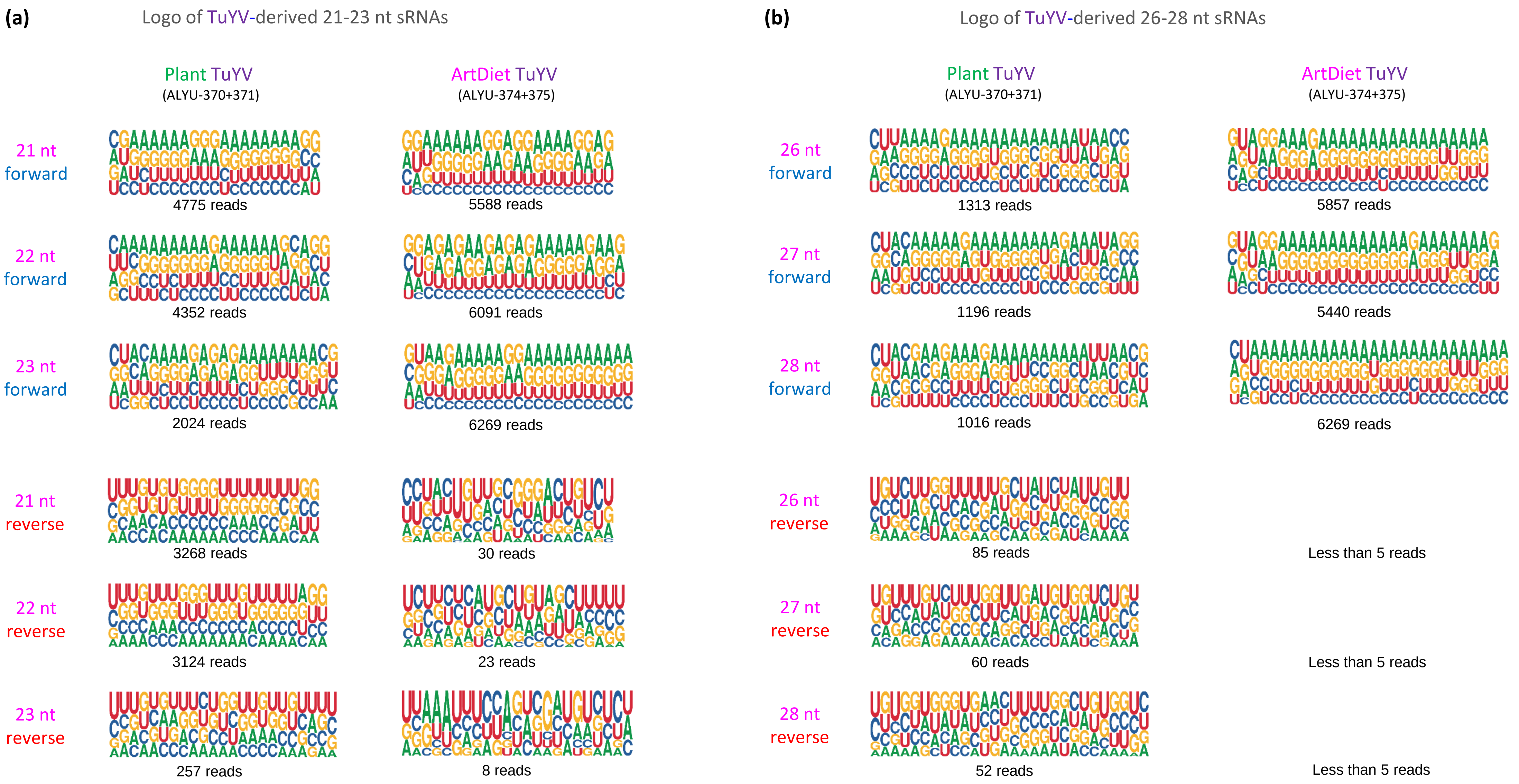

Supplement: Supplementary file 1 [file ijms-25-13199-s001.zip › Fig S12.pdf]
