# Supplementary material for: Deep Sequencing Analysis of Virome Components, Viral Gene Expression and Antiviral RNAi Responses in Myzus persicae Aphids
Source: Int J Mol Sci. 2024 Dec 8;25(23):13199. doi: 10.3390/ijms252313199 (PMC11642819; doi:10.3390/ijms252313199)

Figure S13. Single-base resolution maps and relative abundance of Illumina stranded mRNA-seq reads representing turnip yellows virus (TuYV) from *M. persicae* aphids fed on TuYV-infected *A. thaliana* plants and an artificial diet with purified TuYV virions. (a) TuYV genome and transcriptome organization. Viral genomic (g), subgenomic (sg) and antigenomic (ag) RNAs are depicted as blue (gRNA and sgRNA) and red (agRNA) lines, with ORFs boxed and the encoded proteins named. Viral RdRP activity generating agRNA on the gRNA template and vice versa as well as sgRNA on the agRNA template are indicated by black arrows. (b-c) Single-base resolution maps of TuYV long RNA-derived 75 nt reads from aphids fed on TuYV-infected plants (b) or artificial diet with TuYV virions (c). (d) Relative abundance of TuYV long RNAs in aphids. Illumina mRNA-seq 75 nt reads from the *M. persicae* aphids fed on plants or artificial diets were mapped with zero mismatches to the TuYV reference genome of the mapped reads were sorted by size and polarity (forward, reverse, total) and counted in reads per million (RPM) of total (host and viral) reads (Dataset S3). To generate single-base resolution maps, the mapping data were analyzed using MISIS-2 [30] and visualized using Excel (Datasets S2C). The maps of combined reads from three biological replicates at the two feeding conditions, Plant TuYV (b) and ArtDiet TuYV (c), are presented as histograms that plot the numbers of 75 nt mRNA-seq reads at each nucleotide position of the 5641 nt TuYV genome: blue bars above the axis represent forward reads starting at each respective position, while red bars below the axis represent reverse reads ending at the respective position. Counts in RPM of forward (blue) and reverse (red) reads derived from TuYV genomic and antigenomic RNA, respectively, are presented as bar graphs (d).

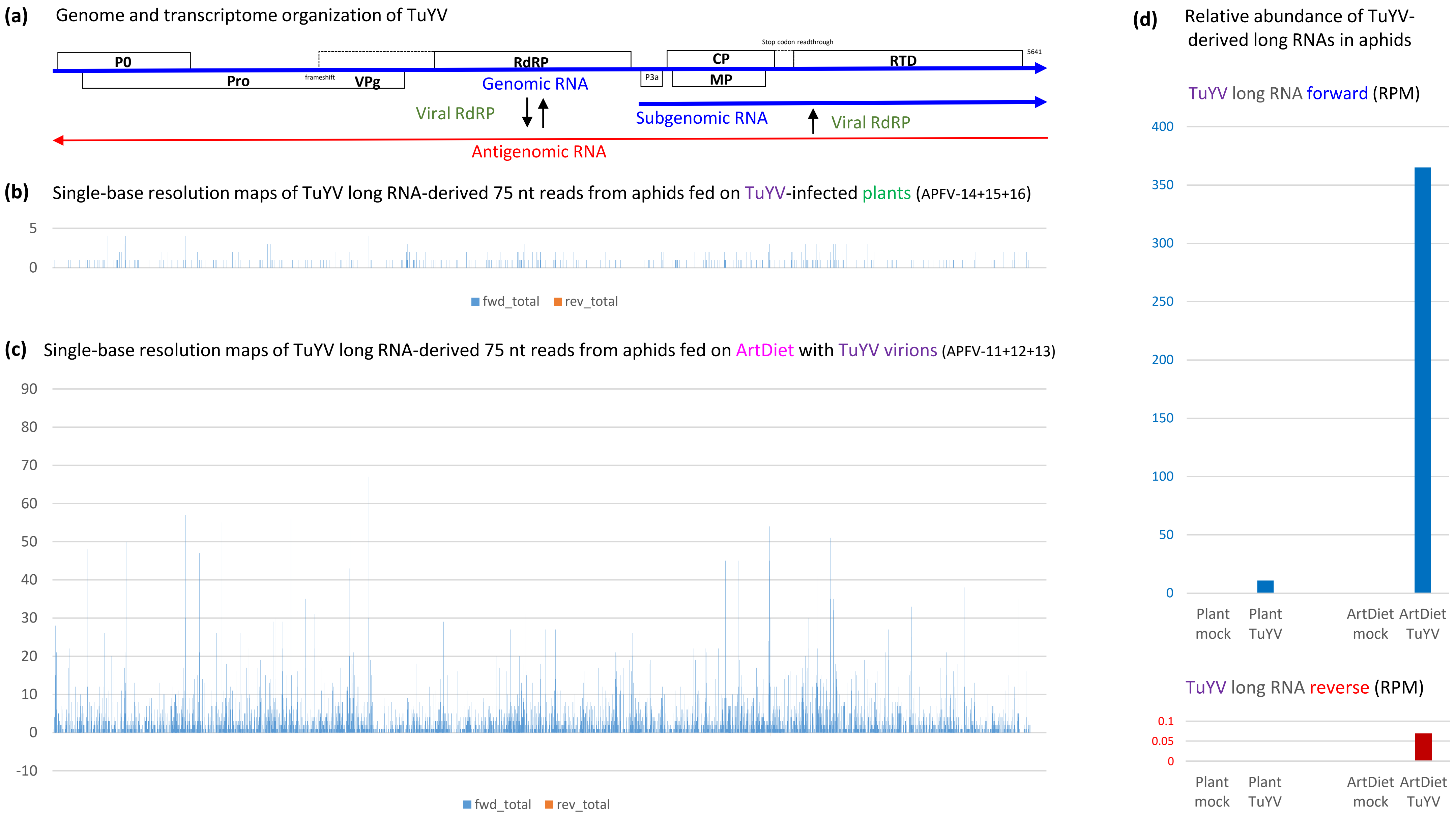

Supplement: Supplementary file 1 [file ijms-25-13199-s001.zip › Fig S13.pdf]
