# Supplementary material for: Deep Sequencing Analysis of Virome Components, Viral Gene Expression and Antiviral RNAi Responses in Myzus persicae Aphids
Source: Int J Mol Sci. 2024 Dec 8;25(23):13199. doi: 10.3390/ijms252313199 (PMC11642819; doi:10.3390/ijms252313199)

Figure S14. PCR analysis of total DNA extracted from two populations of the *M. persicae* aphid clone Mp-Col, one positive for Myzus persicae densovirus (MpDV) and another negative for MpDV. Total DNA was extracted from two biological replicates (pools) of MpDV-negative and MpDV-positive aphids and used for PCR analysis with MpDV-specific primers 5'-AGTTCTGTGGAACGAACC and 5'-AAGCGTCAAACATCCATGA. The PCR products were separated using 1% agarose gel electrophoresis and stained with EtBr. 1 Kb DNA ladder was used as size marker. Position of MpDV-specific PCR product of expected size (648 bp) is indicated by arrow.

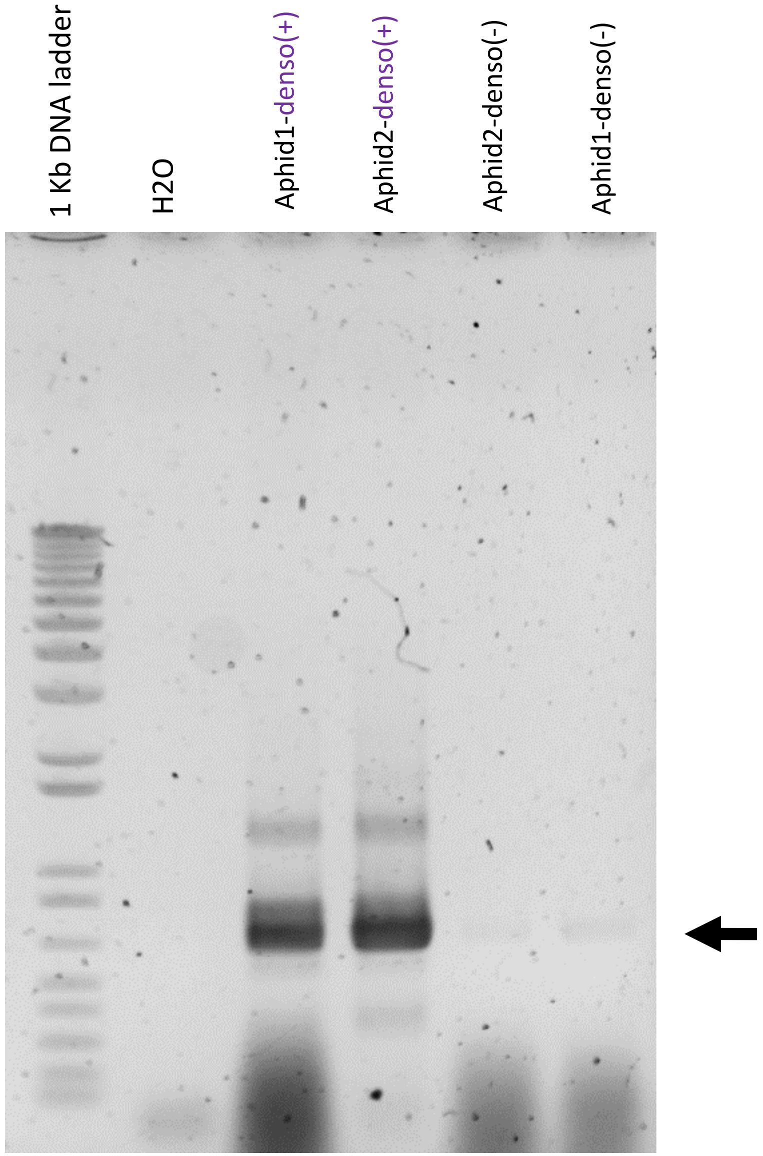

Supplement: Supplementary file 1 [file ijms-25-13199-s001.zip › Fig S14.pdf]
