# Supplementary material for: Deep Sequencing Analysis of Virome Components, Viral Gene Expression and Antiviral RNAi Responses in Myzus persicae Aphids
Source: Int J Mol Sci. 2024 Dec 8;25(23):13199. doi: 10.3390/ijms252313199 (PMC11642819; doi:10.3390/ijms252313199)
Supplement: Supplementary file 1 [file ijms-25-13199-s001.zip › File S1.pdf]

File S1. Sequence analysis of MpDV genome and transcriptome. (**a**) Analysis of the 5’- and 3’-inverted terminal repeat (ITR) sequences of MpDN1 and MpDN2 with Needle pairwise alignments. (**b**) Secondary structures of the 5’- and 3’-telomers of MpDV1 and MpDV2 predicted using RNAFold WebServer: (http://rna.tbi.univie.ac.at/cgi-bin/RNAWebSuite/RNAfold.cgi) with DNA parameters of Matthews model 2004 at 30ºC. (**c**) Multiple sequence alignment of MpDV1, MpDV2 and MpnDV using Muscle v3.8 with annotations (SNPs, indels and cis-elements driving transcription, splicing and translation). (**d**) Multiple sequence alignment of Trinity contigs representing MpDV1 mRNAs. The contigs were assembled from Illumina mRNA-seq reads obtained from *M. persicae* aphids fed on *A. thaliana* mock-inoculated (APFV-5-6-7) or TuYV-infected plants (APFV-11-12-13) or on artificial diets with TuYV virions (APFV-8-9-10) or without virions (APFV-14-15-16).

(a) **Analysis of the 5’- and 3’-inverted terminal repeats (ITR) of MpDN1 and MpDN2** with Needle pairwise alignments

MpDV1

The 5’- and 3’-ITRs of **MpDV1** are imperfect inverted repeats that differ by flop-flop inversion of the 34 nt palindrome sequence and one 1-nt insertion in the 3’-ITR

```
MpDV1 5’ITR              1 ATATAAAAAGATCCCCTACTAATAATAACCACTACCTACTTCCGGGGGGC     50
                           ||||||||||||||||||||||||||||||||||||||||||||||||||
MpDV1 3’ITR rev-com      1 ATATAAAAAGATCCCCTACTAATAATAACCACTACCTACTTCCGGGGGGC     50

MpDV1 5’ITR             51 GGGGGGTATCCTTGTTATCTATACTTATTGTCAACACTAGCCTTATCTTA    100
                           ||||||||||||||||||||||||||||||||||||||||||||||||||
MpDV1 3’ITR rev-com     51 GGGGGGTATCCTTGTTATCTATACTTATTGTCAACACTAGCCTTATCTTA    100

MpDV1 5’ITR            101 GGGC--GTGTC--GGCC--GACACGTGTCATGGCCATGACACTAGCCCTA    144
                           ||||  |||||  ||||  ||||||||||  ||||  |||||  ||||||
MpDV1 3’ITR rev-com    101 GGGCTAGTGTCATGGCCATGACACGTGTC--GGCC--GACAC--GCCCTA    144

MpDV1 5’ITR            145 AGATAAGGCTAGTGTTGACAATAAGTATAGATAAC-AAGGATACCCCCCG    193
                           ||||||||||||||||||||||||||||||||||| ||||||||||||||
MpDV1 3’ITR rev-com    145 AGATAAGGCTAGTGTTGACAATAAGTATAGATAACAAAGGATACCCCCCG    194

MpDV1 5’ITR            194 CCCCCCGGAAGTAGGTAGTGGTTATTATTAGTAGGGGATCTTTTTATATA    243
                           ||||||||||||||||||||||||||||||||||||||||||||||||||
MpDV1 3’ITR rev-com    195 CCCCCCGGAAGTAGGTAGTGGTTATTATTAGTAGGGGATCTTTTTATATA    244

MpDV1 5’ITR            244 ATAAAAAGATAGACAAGTATCCAGTAGGCGACTAGGTGGGGGTGACGTCA    293
                           ||||||||||||||||||||||||||||||||||||||||||||||||||
MpDV1 3’ITR rev-com    245 ATAAAAAGATAGACAAGTATCCAGTAGGCGACTAGGTGGGGGTGACGTCA    294

MpDV1 5’ITR            294 TCACTATTTACTCAAGTGTTT    314
                           |||||||||||||||||||||
MpDV1 3’ITR rev-com    295 TCACTATTTACTCAAGTGTTT    315
```

The 242 nt 5’-terminal sequence and the 243 nt 3’-terminal sequence of the **MpDV1** genome are direct repeats differing by one nucleotide insertion in the 3’-repeat sequence

```
MpDV1 5’ITR 5’-term      1 ATATAAAAAGATCCCCTACTAATAATAACCACTACCTACTTCCGGGGGGC     50
                           ||||||||||||||||||||||||||||||||||||||||||||||||||
MpDV1 3’ITR 3’-term      1 ATATAAAAAGATCCCCTACTAATAATAACCACTACCTACTTCCGGGGGGC     50

MpDV1 5’ITR 5’-term     51 GGGGGGTATCC-TTGTTATCTATACTTATTGTCAACACTAGCCTTATCTT     99
                           ||||||||||| ||||||||||||||||||||||||||||||||||||||
MpDV1 3’ITR 3’-term     51 GGGGGGTATCCTTTGTTATCTATACTTATTGTCAACACTAGCCTTATCTT    100

MpDV1 5’ITR 5’-term    100 AGGGCGTGTCGGCCGACACGTGTCATGGCCATGACACTAGCCCTAAGATA    149
                           ||||||||||||||||||||||||||||||||||||||||||||||||||
MpDV1 3’ITR 3’-term    101 AGGGCGTGTCGGCCGACACGTGTCATGGCCATGACACTAGCCCTAAGATA    150

MpDV1 5’ITR 5’-term    150 AGGCTAGTGTTGACAATAAGTATAGATAACAAGGATACCCCCCGCCCCCC    199
                           ||||||||||||||||||||||||||||||||||||||||||||||||||
MpDV1 3’ITR 3’-term    151 AGGCTAGTGTTGACAATAAGTATAGATAACAAGGATACCCCCCGCCCCCC    200

MpDV1 5’ITR 5’-term    200 GGAAGTAGGTAGTGGTTATTATTAGTAGGGGATCTTTTTATAT    242
                           |||||||||||||||||||||||||||||||||||||||||||
MpDV1 3’ITR 3’-term    201 GGAAGTAGGTAGTGGTTATTATTAGTAGGGGATCTTTTTATAT    243
```

The 242 nt 5’-terminal sequence of the **MpDV1** genome is an imperfect palindrome due three 2-nt (TA) insertions creating **short** and **longer more stable** **stem-loop**s at the hairpin’s top

```
MpDV1 5’-term            1 ATATAAAAAGATCCCCTACTAATAATAACCACTACCTACTTCCGGGGGGC     50
                           ||||||||||||||||||||||||||||||||||||||||||||||||||
MpDV1 5’-term rc         1 ATATAAAAAGATCCCCTACTAATAATAACCACTACCTACTTCCGGGGGGC     50

MpDV1 5’-term           51 GGGGGGTATCCTTGTTATCTATACTTATTGTCAACACTAGCCTTATCTTA    100
                           ||||||||||||||||||||||||||||||||||||||||||||||||||
MpDV1 5’-term rc        51 GGGGGGTATCCTTGTTATCTATACTTATTGTCAACACTAGCCTTATCTTA    100

                                 ---->  loop  <----------->loop<------
MpDV1 5’-term          101 GGGC--GTGTC--GGCC--GACACGTGTCATGGCCATGACACTAGCCCTA    144
```

```
                          ||||  |||||  ||||  ||||||||||  ||||  |||||  ||||||
MpDV1 5'-term rc      101 GGGCTAGTGTCATGGCCATGACACGTGTC--GGCC--GACAC--GCCCTA    144

MpDV1 5'-term         145 AGATAAGGCTAGTGTTGACAATAAGTATAGATAACAAGGATACCCCCCGC    194
                          ||||||||||||||||||||||||||||||||||||||||||||||||||
MpDV1 5'-term rc      145 AGATAAGGCTAGTGTTGACAATAAGTATAGATAACAAGGATACCCCCCGC    194

MpDV1 5'-term         195 CCCCCGGAAGTAGGTAGTGGTTATTATTAGTAGGGGATCTTTTTATAT    242
                          ||||||||||||||||||||||||||||||||||||||||||||||||
MpDV1 5'-term rc      195 CCCCCGGAAGTAGGTAGTGGTTATTATTAGTAGGGGATCTTTTTATAT    242
```

The 243 nt 3'-terminal sequence of the **MpDV1** genome is an imperfect palindrome due one 1-nt (T) insertion and three 2-nt (TA) insertions creating **longer more stable** **stem-loop**s at the hairpin's top

```
MpDV1 3'-term           1 ATATAAAAAGATCCCCTACTAATAATAACCACTACCTACTTCCGGGGGGC     50
                          ||||||||||||||||||||||||||||||||||||||||||||||||||
MpDV1 3'-term rc        1 ATATAAAAAGATCCCCTACTAATAATAACCACTACCTACTTCCGGGGGGC     50

MpDV1 3'-term          51 GGGGGGTATCCTTTGTTATCTATACTTATTGTCAACACTAGCCTTATCTT    100
                          ||||||||||| ||||||||||||||||||||||||||||||||||||||
MpDV1 3'-term rc       51 GGGGGGTATCC-TTGTTATCTATACTTATTGTCAACACTAGCCTTATCTT     99

                                 ---->  loop  <----------->loop<------
MpDV1 3'ITR           101 AGGGC--GTGTC--GGCC--GACACGTGTCATGGCCATGACACTAGCCCT    144
                          |||||  |||||  ||||  ||||||||||  ||||  |||||  |||||
MpDV1 3'-term rc      100 AGGGCTAGTGTCATGGCCATGACACGTGTC--GGCC--GACAC--GCCCT    143

MpDV1 3'-term         145 AAGATAAGGCTAGTGTTGACAATAAGTATAGATAAC-AAGGATACCCCCC    193
                          |||||||||||||||||||||||||||||||||||| |||||||||||||
MpDV1 3'-term rc      144 AAGATAAGGCTAGTGTTGACAATAAGTATAGATAACAAAGGATACCCCCC    193

MpDV1 3'-term         194 GCCCCCCGGAAGTAGGTAGTGGTTATTATTAGTAGGGGATCTTTTTATAT    243
                          ||||||||||||||||||||||||||||||||||||||||||||||||||
MpDV1 3'-term rc      194 GCCCCCCGGAAGTAGGTAGTGGTTATTATTAGTAGGGGATCTTTTTATAT    243
```

MpDV2

The 5'- and 3'-ITRs of **MpDV2** are imperfect inverted repeats that differ by flop-flop inversion of the 33 nt flop-flop palindrome sequence, one 1-nt insertion in the 5'-ITR and one SNP

```
MpDV2 5'ITR             1 TGTCAACACTAGCCCTTATCTTAGGCC-GTGTC--GGCC--GACACGTGT     45
                          ||||||||||||||||||||||||||| |||||  ||||  |||||||||
MpDV2 3'ITR rev-com     1 TGTCAACACTAGCCCTTATCTTAGGCCAGTGTCATGGCCATGACACGTGT     50

MpDV2 5'ITR            46 CATGGCCATGACACTAGCCCTAAGATAAGGGCTAGTGTTGACAATAAGTA     95
                          |  ||||  |||||  ||||||||||||||||||||||||||||||||||
MpDV2 5'ITR rev-com    51 C--GGCC--GACAC--GCCCTAAGATAAGGGCTAGTGTTGACAATAAGTA     94

MpDV2 5'ITR            96 TAGATAACAAAGGATACCCCCCGCCCCCCGGAAGTAGGTAGTGGGTATTA    145
                          ||||||||||||||||||||||||||||||||||||||||||||||||||
MpDV2 5'ITR rev-com    95 TAGATAACAAAGGATACCCCCCGCCCCCCGGAAGTAGGTAGTGGGTATTA    144

MpDV2 5'ITR           146 GTAGGGGATCTTTTTATATAATAAAAAGATAGACAAGTATCCAGCAGGCG    195
                          ||||||||||||||||||||||||||||||||||||||||||||||||||
MpDV2 3'ITR rev-com   145 GTAGGGGATCTTTTTATATAATAAAAAGATAGACAAGTATCCAGCAGGCG    194

MpDV2 5'ITR           196 ACTAGGTGGGGGTGACGTCATCATTATTTACTCAAGTGTTT    236
                          |||||||||||||||||||||||.|||||||||||||||||
MpDV2 3'ITR rev-com   195 ACTAGGTGGGGGTGACGTCATCACTATTTACTCAAGTGTTT    235
```

The 88 nt 5'-terminal sequence and the 87 nt 3'-terminal sequence of the **MpDV2** genome are imperfect direct repeats differing by one 1-nt insertion in the 5'-repeat sequence and 2 SNPs

```
MpDV2 5'ITR 5'-term     1 TGTCAACACTAGCCCTTATCTTAGGCCGTGTCGGCCGACACGTGTCATGG     50
                          |||||||||||||||||||||||||.||||||||||||||||||||||||
MpDV2 3'ITR 3'-term     1 TGTCAACACTAGCCCTTATCTTAGGGCGTGTCGGCCGACACGTGTCATGG     50

MpDV2 5'ITR 5'-term    51 CCATGACACTAGCCCTAAGATAAGGGCTAGTGTTGACA     88
                          |||||||||| |.|||||||||||||||||||||||||
MpDV2 3'ITR 3'-term    51 CCATGACACT-GGCCTAAGATAAGGGCTAGTGTTGACA     87
```

The 88 nt 5'-terminal sequence of the **MpDV2** genome is an imperfect palindrome due to 2 SNPs and three 2-nt (TA) insertions creating **stable** and **less stable** **stem-loop**s of same size and shape at the hairpin's top

```
                                                     -  ---->  loop  <---------->loop<-----
MpDV2 5'-term           1 TGTCAACACTAGCCCTTATCTTAGGCC--GTGTC--GGCC--GACACGTGTCATGGCCATGACACTAGCCC     65
                          |||||||||||||||||||||||||.|  |||||  ||||  ||||||||||  ||||  |||||  |.||
MpDV2 5'-term rc         1 TGTCAACACTAGCCCTTATCTTAGGGCTAGTGTCATGGCCATGACACGTGTC--GGCC--GACAC--GGCC     65

MpDV2 5'-term          66 TAAGATAAGGGCTAGTGTTGACA     88
                          |||||||||||||||||||||||
MpDV2 5'-term rc       66 TAAGATAAGGGCTAGTGTTGACA     88
```

The 87 nt 3′-terminal sequence of the **MpDV2** genome is an imperfect palindrome due to 2 SNPs and two 2-nt (AT) and one 1-nt (T) insertions creating **stable** and **less stable** **stem-loop**s of same size and shape at the hairpin's top

```
                                                              - ---->  loop  <----------->loop<-----
MpDV2 3′-term          1 TGTCAACACTAGCCCTTATCTTAGGGC-GTGTC--GGCC--GACACGTGTCATGGCCATGACACTGGCCTA     66
                         |||||||||||||||||||||||||.| |||||  ||||  ||||||||||  ||||  ||||| |.||||
MpDV2 3′-term rc       1 TGTCAACACTAGCCCTTATCTTAGGCCAGTGTCATGGCCATGACACGTGTC--GGCC--GACAC-GCCCTA     66


MpDV2 3′-term         67 AGATAAGGGCTAGTGTTGACA     87
                         |||||||||||||||||||||
MpDV2 3′-term rc      67 AGATAAGGGCTAGTGTTGACA     87
```

(**b**) **Secondary structures of the 5′- and 3′-telomers of MpDV1 and MpDV2** predicted using RNAFold WebServer: (http://rna.tbi.univie.ac.at/cgi-bin/RNAWebSuite/RNAfold.cgi) with DNA parameters of Matthews model 2004 at 30ºC.

MpDV1 5′-telomer (-43.49 kcal/mol)

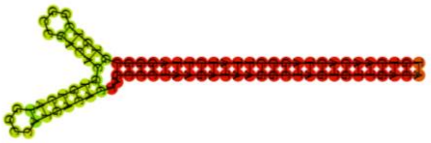

MpDV1 3′-telomer (-43.49 kcal/mol)

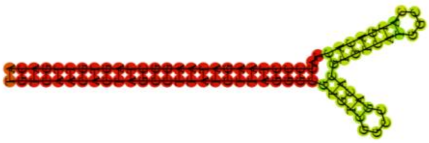

MpDV2 5′-telomer (-41.44 kcal/mol)

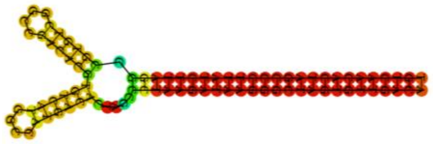

MpDV2 3′-telomer (-44.21 kcal/mol)

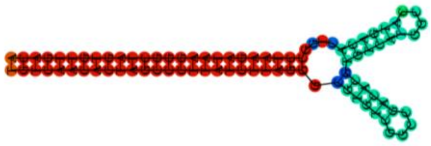

Base-pair probabilities: **0** [color scale] **1**

(**c**) **Multiple sequence alignment of MpDV1, MpDV2 and MpnDV genomes** using Muscle v3.8 with annotations (SNPs, indels and **cis-elements** driving **transcription**, **splicing** and **translation**)

```
CLUSTAL multiple sequence alignment by MUSCLE (3.8)

MpDN1           ATATAAAAAGATCCCCTACTAATAATAACCACTACCTACTTCCGGGGGGCGGGGGGTATC
MpDV2           ------------------------------------------------------------
MpnDV           ------------------------------------------------------------


MpDN1           CTTGTTATCTATACTTATTGTCAACACTAG-CCTTATCTTAGGGCGTGTCGGCCGACACG
MpDV2           ------------------TGTCAACACTAGCCCTTATCTTAGGCCGTGTCGGCCGACACG
MpnDV           ------------------------------------------------------------


MpDN1           TGTCATGGCCATGACACTAGCCCTAAGATAA-GGCTAGTGTTGACAATAAGTATAGATAA
MpDV2           TGTCATGGCCATGACACTAGCCCTAAGATAAGGGCTAGTGTTGACAATAAGTATAGATAA
MpnDV           ------------------------------------------------------------


MpDN1           C-AAGGATACCCCCCGCCCCCCGGAAGTAGGTAGTGGTTATTATTAGTAGGGGATCTTTT
MpDV2           CAAAGGATACCCCCCGCCCCCCGGAAGTAGGTAGTGG---GTATTAGTAGGGGATCTTTT
MpnDV           ----------------------GGAAGCAGGCAGTGG---GTATTAGTAGGGGATCTTTT
                                      ***** *** *****    *******************

MpDN1           TATATAATAAAAAGATAGACAAGTATCCAGTAGGCGACTAGGTGGGGGTGACGTCATCAC 5′ITR-based TATA-box
MpDV2           TATATAATAAAAAGATAGACAAGTATCCAGCAGGCGACTAGGTGGGGGTGACGTCATCAT
MpnDV           TATATAATAAAAAGATAGACAAGTATCCAGCAGGCGACTAGGTGGGGGTGACGTCATCAC
                ****************************** ***************************** 

MpDN1           TATTTACTCAAGTGTTTTGTTATTCGGC----------------------GTCAAGTGTT
MpDV2           TATTTACTCAAGTGTTTTGTTATTCGGCGTCAAGTGGTTTGTTATTCAATGTCAAGTGTT
MpnDV           TATTTACTCAAGTGTTTTGTTATTCGGCGTCAAGTGGTTTGTTATTCAACGTCAAGTGTT
                ****************************                      **********

MpDN1           GTGTTGACACTTGAAAAGTGGCGTGATTGATTATTTACAAGTACGTCAGATAAGATAAAC
MpDV2           GTGTTGACACTTGAAAAGTAGCGTGATTGATTATTTACAAGTACGTCAGATAAGATAGAC
```

```
MpnDV          GTGTTGACACTTGAAAAATGGCGTGATTGATTATTTACAAGTACGTCAGATAAGATAAAC
               ***************** * ************************************* **

MpDN1          ATAAGTGGTACTTGACTCGGAATATATAATGAAGTGTGGAACCTACAACAATATCAGTAC main TATA-box of NS unit and
MpDV2          ACAAGTGGTACTTGACTCGGAATATATAATGAAGTGTGGAACCTACAACAACATCAGTAC first AUG codon of NS2 ORF
MpnDV          ACAAGTGGTACTTGACTCGGAATATATAATGAAGTGTGGAACCTACAACAACATCAGTAC
               * ************************************************* ********

MpDN1          TCATCAAAATGTCAAACTCGCAAGAAGTGAACCAACAACAATTCAACGACGAGGAGGAAA downstream AUG of NS2 ORF
MpDV2          TCATCAAAATGTCAATCTCGCAAGAAGTGAACCAACAACAATTCAACGACGAGGAGGAAA
MpnDV          TCATCAAAATGTCAAACTCGCAAGAAGTGAACCAACAACAGTTCAACGACGAGGAGGAAA
               *************** ************************ *******************

MpDN1          TACCATGTGGCCAACGGTATCCTACAGCGGCGACGATTCAAACGGACCACCAACAGACTG AUG codon of NS1 ORF
MpDV2          TACCATGTGGCCAACGGTATACTACAGCGGCGACGATTCAAACGGACCACCAACAGACTG
MpnDV          TACCATGTGGCCAACGGTATCCTACAGCGGCGACGATTCAAACGGACCACCAACAGACTG
               ******************** ***************************************

MpDN1          GCGTGAATGGATCGACCCCGATGTTCGAACTCAAATCGACAGCGCGCAAAGAATATGGGA
MpDV2          GCGTGAATGGATCGACCCCGATGTTCGAACTCAAATCGACAGCGCGCAAAGAATATGGGA
MpnDV          GCGTGAATGGATCGACCCCGATGTTCGAACTCAAATCGACAGCGCGCAAAGAATATGGGA
               ************************************************************

MpDN1          CAGCCATTTCAGCGGACACGGCACTGACACTGAAGACGCTGTGCAAAGTGTTGGAGTGCA
MpDV2          CAAGCATTTCAGCGGACATGGCACTGACACTGAAGACGCTGTGCAAAGTGTTGGAGTGCA
MpnDV          CAAGCATTTCAGCGGACATGGCACTGACACTGAAGACGCTGTGCAAAGTGTTGGAGTGCA
               **  ************** *****************************************

MpDN1          AGGCAACGGAACTAACGGTAACATGCATTATTCCTCTGATCGCGTTGCTATTACCCGACC
MpDV2          AGGCAACGGAACTAACGGTAACATGCATTATTCCTCTGATCGCGTTGCTATTACCCGACC
MpnDV          AGGCAACGGAGCTAACGGTAACGTGCATTATTCCTCTGATCGCGTTGCTATTACCCGACC
               ********** *********** *************************************

MpDN1          ATATGTGGTCAGAGGCGACGAGCAGCGATCGCTGGCGATACCGACTGAGAAACGCGACCT
MpDV2          ATATGTGGTCAGAGGCGACGAGCAGCGATCGCTGGCGATACCGACTAAGAAATGCGACCT
MpnDV          ATATGTGGTCAGAGGCGACGAGCAGCGATCGCTGGCGATACCGACTAAGAAATGCGACCT
               ********************************************** ***** *******

MpDN1          ACTGGATTTCCAATCCAACGCGGGAATCCGAATATCAGGCTTTACTACGATGGATGGAGG
MpDV2          ACTGGATTTCCAATCCAACGCGGGAATCCGAATATCAGGCTTTACTACGATGGATGGAGG
MpnDV          ACTGGATTTCCAATCCAACGCGGGAATCCGAATATCAGGCTTTACTACGATGGATGGAGG
               ************************************************************

MpDN1          AGAAAATGCCGATACCTTTAGCGAAATGGCAAGGTGCATGTCTACCGCGGTATGGGGAGG
MpDV2          AGAAAATGCCGATACCTTTAGCGAAATGGCAAGGTGCATGTCTACCGCGGTATGGGGAGG
MpnDV          AGAAAATGCCGATACCTTTAGCGAAATGGCAAGGTGCATGTCTACCGCGGTATGGGGAGG
               ************************************************************

MpDN1          CAATGTCAGATATGTTTCTGACATCCTTGTACCCGGAGGAAATTGGTCAATTAATGGAAT
MpDV2          CAATGTCAGATATGTTTCTGACATCCTTGTACCCGGAGGAAATTGGTCAATTAATGGAAT
MpnDV          CAATGTCAGATATGTTTCTGACATCCTTGTACCCGGAGGAAATTGGTCAATTAATGGAAT
               ************************************************************

MpDN1          TGTTGAGGACCTACGCCGGAGTATCGCAGAGCGAATGCCAAAATGCATGTACGTCGTCAG
MpDV2          TGTTGAGGACCTACGCCGGAGTATCGCAGAGCGAATGCCAAAATGCATGTACGTCGTCAG
MpnDV          TGTTGAGGACCTACGCCGGAGTATCGCAGAGCGAATGCCAAAATGCATGTACGTCGTCAG
               ************************************************************

MpDN1          CGAACACGGCGACCACGTCCACGTCGTACACACCTGTAACTACGCCACGGGCAGCTGTAG
MpDV2          CGAACACGGCGACCACGTCCACGTCGTACACACCTGTAACTACGCCACGGGCAGCTGTAG
MpnDV          CGAACACGGCGACCACGTCCACGTCGTGCACACCTGTAACTACGCCACGGGCAGCTGCAG
               *************************** ****************************** **

MpDN1          ATGTAGTTTCTTACTCAACGCCGCTGCTTTCCAACTATGTAGACGACGACGCATTCGAAA
MpDV2          ATGTAGTTTCTTACTCAACGCCGCTGCTTTCCAACTATGTAGACGACGACGCATTCGAAA
MpnDV          ATGTAGTTTCTTACTCAACGCCGCTGCTTTCCAACTATGTAGACGACGACGCATTCGAAA
               ************************************************************

MpDN1          GAATGTTAGAGTCATCCAACTATCAGAAAGAGACTGGTCCCGTATCTTTCAATACTTATG
MpDV2          GAATGTTAGAGTCATCCAACTATCAGAAAGAGACTGGTCCCGTATCTTTCAATACTTATG
MpnDV          GAATGTTAGAGTCATCCAACTATCAGAAAGAGATTGGTCCCGTATCTTTCAATACTTATG
               ********************************* **************************

MpDN1          TTCATCGCCCAGGAATGTCAAAGAAGTGGGCGGCCAGTACTTTGATGGAGGATTACGTAG
MpDV2          TTCATCGCCCAGGAATGTCAAAGAAGTGGGCGGCCAGTACTTTGATGGAGGATTACGTAG
MpnDV          TTCATCGCCCAGGAATGTCAAAGAAGTGGGCGGCCAGTACTTTGATGGAGGATTACGTAG
               ************************************************************

MpDN1          TAGATATTCACATATATCAGTTAGTATTGCGTAATTTTTTTATATACTTGGATTGGTGA 5′ splice site of NS intron
MpDV2          TAGATATACACATATATCAGTTAGTATTGCGTAATTTTTTTATATACTTGGATTGGTGA Stop codon of NS2 ORF
MpnDV          TAGATATACACATATATCAGTTAGTATTGCGTAATTTTTTTATATACTTGGATTGGTGA
               ******* ****************************************************

MpDN1          CGGGTATAGAAGCGAATAGATGCGTTTCATATATTAAAACTGGTATGCCTGGCAGGAGTT
MpDV2          CGGGTATAGAAGCAAATAGATGCGTTTCATATATTAAAACCGGTATGCCTGGAATGAGTT
MpnDV          CGGGTATAGAAGCAAATAGATGCGTTTCATATATTAAAACCGGTATGCCTGGAATGAGTT
               ************* ************************** *********** * *****
```

```
MpDN1          ACGTTAAAAGCGTGATGCCAAAAAATTATTGGCGTAATCCTAAAGAATTTCACTTAGCGC
MpDV2          ACGTTAAAAGTGTGATTCCGAAAAATTATTGGCGTAATCCTAAAGAATTTCACTTAGCGC
MpnDV          ACGTTAAAAGTGTGATGCCGAAAAATTATTGGCGTAATCTTAAAGAGATTCACTTAGCGC
               ********** ***** ** ******************* ******  ************

MpDN1          TTGATAAATTAGAGTTGCTCGTTAAACGACATTTCGGTGATTTCACGTGTTTGGAAAATT
MpDV2          TTGATAAATTAGAGTTGCTCGTTAAACGACATTTCGGTGATTTCACGTGTTTGGAAAATT
MpnDV          TTGATAAATTAGAGTTGCTCGTTAAACGACATTTCGGTGATTTCACGTGTTTGGAAAATT
               ************************************************************

MpDN1          TTTTACCGCAATATCGTTGGCATAATTGCATCACATGGTTTGTGAACGTGATGTCCTGTC
MpDV2          TTTTACCGCAATATCGTTGGCATAATTGCATAACATGGTTTGTGAACGTGATGTGCTGTC
MpnDV          TTTTACCGCAATATCGTTGGCATAATTGCATCACATGGTTTGTGAACGTGATGTGCTGTC
               ******************************* ********************** *****

MpDN1          CGTTGGACCCGTGGACTGTCGAATGTGCTGGTTGTGGTTTTCAACATATTCCTAATATAT
MpDV2          CGTTGGACCCGTGGACTGTCGAATGTGGTGGTTGTGGTTTTCAACATATTCCTAATGTAT
MpnDV          CGTTGGACCCGTGGACTGTCGAATGTGGTGGTTGTGGTTTTCAACATATTCCTAATGTAT
               *************************** **************************** ***

MpDN1          CTAAATGCGAACTACTACGAGACTATCCGTTCATGAACGAGGGCACTATAAATCATATAC
MpDV2          CTAAATGCGAACTACTACGAGACTATCCGTTCATGAACGAGGGCACTATAAATCATATAT
MpnDV          CTAAATGCGAACTACTACGAGACTATCCGTTCATGAACGAGGGCACTATAAATCATATAT
               *********************************************************** 

MpDN1          TAATCGAATGCATTAAAATTTACAAACAGGAAGGATCAAATACAGAATGTCGACCCGAAC 3’ splice site of NS intron
MpDV2          TAATCGAATGCATTAAAATTTACAAACAGGAAGGATCAAATCCAGAATGTCGACCCGAAC
MpnDV          TAATCGAATGCATTAAAATTTACAAACAGGAAGGATCAAATCCAGAATGTCGACCTGAAC
               ***************************************** ************* ****

MpDN1          GATTGGTGGAAACATGCCTTCTGCAGGGCTCGGGGGAGCTTCAATCTGAACAGTCCATAC
MpDV2          GATTGGTGGAAACATGCCTTCTGCAGGGCTCGGGGGAGCTTCAATCTGAACAGTCCTTAC
MpnDV          GATTGGTGGAAACATGCCTTCTGCAGGGCTCGGGGGAGCTTCAATCTGAACAGTCCTTAC
               ******************************************************** ***

MpDN1          GTGAAAATGGCCAAAACCATACAACAGGAAGTGTTTCGCAGGATGACCAAGATGGAGTAC
MpDV2          GTGAAAATGGCCAAAACCATACAACAGGAAGTGTTTCGCAGGATGACCAAGATGGAGTAC
MpnDV          GTGAAAATGGCCAAAACCATACAACAGGAAGTGTTTCGCAGGATGACCAAGATGGAGTAC
               ************************************************************

MpDN1          AAGATAACGGATCCACAAATGTTTTTCAAGAAGAAGAAGAGGGCAGCCAAGAGGGCCCGC
MpDV2          AAGATAACGGATCCACAAATGTTTTTCAAGAAGAAGAAGAAGGCAGCCAAGAGGGCCCGC
MpnDV          AAGATAACGGATCCACAAATGTTTTTCAAGAAGAAGAAGAAAGCAGCCAAGAGGGCCCGC
               ****************************************  ******************

MpDN1          GGCAAAAGAAGTCGCGACGGAAGCAGCGATTCCGAATGATTAGGGAAATAGATGTTAGTA Stop codon of C-truncated NS1
MpDV2          GGCAAAAGAAGTCGCGACGGAAGCAGCGATTCCGAATGATTAGGGAAATAGATGTTAGTA translated from spliced mRNA
MpnDV          GGCAAAAGAAGTCGCGACGGAAGCAGCGATTCCGAATGATTAGGGAAATAGATGTTAGTA
               ************************************************************

MpDN1          AACCGCAATCTATCGAAGAACTTATATATAGGTATCCTTGTTGCCCCCCGGAAGCTTTCT
MpDV2          AACCGCAATCTATCGAAGAACTTATATATAGGTATCCTTGTTGCCCACCGGAAGCTTTCT
MpnDV          AACCGCAATCTATCGAAGAACTTATATATAGATATCCTTGTTGTCCACCGGAAGCTTTCT
               ******************************* *********** ** *************

MpDN1          ATAACATTCCCGAATTTTACGCTAATACAAATATAAACTGGATAGACATGAAAGATTTTA
MpDV2          ATAACATTCCCGAATTTTACGCTAATAAAAATATAAACTGGATAGACATGAAAGATTTTA
MpnDV          ATAACATTCCCGAATTTTACGCTAATAAAAATATAAACTGGATAGACATGAAAGATTTTA
               ************************** *********************************

MpDN1          AAGTAACGATACCGTTACGTAATTGGGCGGCCACTTTACGCAGATGGTCTATTCATGATT
MpDV2          AAGTAACGATACCGTTGCGTAATTGGGCGGCGACTTTACGTAGATGGTCTATTCATGATT
MpnDV          AAGTAACGATACCGTTACGTAATTGGGCGGCGACTTTACGTAGATGGTCTATTCATGATT
               **************** ************** ******** *******************

MpDN1          TCAATAAATATTATAATGACTCTACAGTTTTTCCATATTTCAACGCGTACGGTTCAGATA
MpDV2          TCAATAAATATTATAATGACTCTACAGTTTTTCCATATTTCAACGCGTACGGTTCAGATA
MpnDV          TCAATAAATATTATAATGACTCTACAGTTTTTCCATATTTCAACGCGTACGGTTCAGATA
               ************************************************************

MpDN1          TTTGCAATATGTATTACAGTATTTCGGAGAGTCTGACAATAGCAAAAGAATTACTCAACT
MpDV2          TTCGCAATATGTATTACAGTATTTCGGAGAGTTTAACAATAGCAAAAGAATTACTCAACT
MpnDV          TTTGCAATATGTATTACAGTATTTCGGAGAGTTTAACAATAGCAAAAGAATTACTCAACT
               ** ***************************** * *************************

MpDN1          ATCAATTTGGCGATGACCCTGAAGTAGTAATTGAATTTTTAACAACACTATATAATGTCA
MpDV2          ATCAATTTGGCGATGACCCTGAAGTAGTAATTGAATTTTTAACAACACTATATAATGTCA
MpnDV          ATCAATTTGGCGATGACCCCGAGGTAGTAATTGAATTTTTAACAACGCTATATAATGTCA
               ******************* ** *********************** *************

MpDN1          TAGACAAAAGAGTTCCGAAATTAAATAGTATATGTATAAAAAGTCCCCCTTCGGCAGGTA
MpDV2          TAGACAAAAGAATTCCGAAATTAAATAGTATATGTGTAAAAAGTCCCCCTTCGGCAGGTA
MpnDV          TAGACAAAAGAGTTCCGAAATTAAATAGTATATGTATAAAAAGTCCCCCTTCGGCAGGTA
               *********** *********************** ************************

MpDN1          AGAACTTTTTTTTCGATGCCGTTGCATCGTATTTGCTTTCATATGGTATGTTCGGTACAG
MpDV2          AAAACTTTTTTTTCGATGCCGTTGCATCGTATTTGCTTTCATATGGTATGTTCGGTACAG
MpnDV          AAAATTTTTTTTTCGATGCCGTTGCATCGTATTTGCTTTCATATGGTATGTTCGGTACAG
```

```
                      * ** *******************************************************

MpDN1                 CCAATAAAAACAATAATTTTTCGTGGGCAGACGGAGCGGGTAAACGATTAGTTCTGTGGA
MpDV2                 CCAATAAAAACAATAATTTTTCGTGGGCAGACGGAGCGGGTAAACGATTAGTTCTATGGA
MpnDV                 CCAATAAAAACAATAATTTTTCGTGGGCAGACGGAGCGGGTAAACGATTAGTTTTATGGA
                      ***************************************************** * ****

MpDN1                 ACGAACCAAACTATGAACAATACCATATAGAAAAAATAAAAGAACTTTTGGGGGGAGATA
MpDV2                 ACGAACCAAACTATGAACAATACCATATAGAAAAAATAAAAGAACTCTTGGGGGGAGATA
MpnDV                 ACGAACCAAACTATGAACAATACCATATAGAAAAAATAAAAGAACTCTTAGGGGGAGATA
                      ********************************************** ** **********

MpDN1                 CAACAAGAATACATGTCAAATATGCAAACGACGTTAGTGTGCAAAGGGTACCCATTATTA
MpDV2                 CAACAAGAATACATGTAAAATATGCAAACGACGTTAGTGTGCAAAGGGTACCCATTATTA
MpnDV                 CAACAAGAATACATGTAAAATATGCAAACGACGTTAGTGTGCAAAGGGTACCCATTATTA
                      **************** *******************************************

MpDN1                 TATTAACAAATAACCACTTGAATATTATTAGTCACCCAGCTTTTAACGATAGATTGCGGA
MpDV2                 TATTAACAAATAACCATTTGAATATTATTAACCACCCAGCTTTTAACGATAGATTGCGGA
MpnDV                 TATTAACAAATAACCACTTGAATATTATTAGTCACCCAGCTTTTAACGATAGATTGCGGA
                      **************** *************  ****************************

MpDN1                 GTTACGAATGGATGTCAGCTGGCTTCTTGAAGGACTACGATAAAAAACTGCATCCGCTTA
MpDV2                 GTTACGAATGGATGTCAGCTGGCTTCTTGAAGGACTACGATAAAAAACTGCATCCGCTTA
MpnDV                 GTTACGAATGGATGTCAGCTGGCTTCTTGAAGGACTATGATAAAAAACTGCATCCGCTTA
                      ************************************** *********************

MpDN1                 TGTTTTATGAATTACTTAAAGATTATGGTGTAATAAATGATAACAATATGATTGATTAAT Poly(A) signal of NS unit
MpDV2                 TGTTTTATGAATTACTTAAAGATTATGGTGTAATAAATGATAACAATATGATTGATTAA- Stop codon of NS1 ORF
MpnDV                 TGTTTTATGAATTACTTAAAGATTATGGTGTAATAAATGATAACAATATGATCGATTAA-
                      ***************************************************** *****

MpDN1                 ATATATATATATATATTGATTTTTATTTACGTAAAAAATCTACAGGTTCATTGACAATGG Poly(A) signal of VP unit
MpDV2                 ---------TATATATTGCTTTTTATTTACGTAAAAAATCTACAGGTTCATTGACAATGG Stop codon of VP1 ORF
MpnDV                 ---------TATATATTGCTTTTTATTTACGTAAAAAATCTACAGGTTCATTGACAATGG
                               ********* *****************************************

MpDN1                 GTATATTCATTGACCTTCGCCACAATGTTAAATCACGGCAAAAAGCTAAATACGTTGAAT
MpDV2                 GTATATTCATTGACCTTCGCCACAATGTTAAATCACGACAAAAAGCTAAATATGTAGAAT
MpnDV                 GTATATTCATTGACCTTCGCCACAATGTTAAATCACGGCAAAAAGCTAAATATGTAGAAT
                      ************************************** ************* ** ****

MpDN1                 ATGACTTGAACAATCGTTCCTCTGGCCACCAATTCGGATTATTGTAGTCGCCCCAAGAAG
MpDV2                 ATGACTTGAACAATCGTTCCTCTGGCCACCAATTCGGATTATTGTAGTCGCCCCAAGAAG
MpnDV                 ATGACTTGAACAATCGTTCCTCTGGCCACCAATTCGGATTATTGTAGTCGCCCCAAGAAG
                      ************************************************************

MpDN1                 TGTTCATGATTTCTTCGGTTGTGGGTGGAGTAGTGGTATATCGATGCAAATTAATTAGGG
MpDV2                 TGTTCATGATTTCTTCGGTTGTGGGTGGAGTAGTGGTGTATTTGTGCAAATTAATTAGGG
MpnDV                 TGTTCATGATTTCTTCGGTAGTGGGTGGAGTAGTGGTGTATTTGTGCAAATTAATTAGGG
                      ******************* ***************** ***   ****************

MpDN1                 TTTCTTTTGACGCACGATCACTTTCTCTGTAAACATAATTGGGTATTTTAATAGGTGCTT 3′ splice site of VP intron 2
MpDV2                 TTTCTTTTGACGCACGATCACTTTCTCTGTAAACATAATTGGGTATTTTAATAGGTGCTT
MpnDV                 TTTCTTTTGACGCACGATCACTTTCTCTGTAAACATAATTGGGTATTTTAATAGGTGCTT
                      ************************************************************

MpDN1                 TGACTCGAACCTCATGGATGTTTGACGCTTCAACCTCCATGGGGTCGTCGTCCATATTTA
MpDV2                 TGACTCGAACCTCATGGATGTTTGATGCTTCAACCTCCATGGGGTCGTCGTCCATATTTA
MpnDV                 TGACTCGAACCTCATGGATGTTTGATGCTTCAACCTCCATGGGGTCGTCGTCCATATTTA
                      ************************* **********************************

MpDN1                 CCCGAACAGACGTAGGTACAGGTGTAGTGACAGTTGCGTCGACTGGAGTAGTAAATGAAT 5′ splice site of VP intron 2
MpDV2                 CCCGAACAGACGTAGGTACAGGTGTAGTGACAGTTGCGTCGGCTGGAGTAGTAAATGAAT
MpnDV                 CCCGAACAGACGCAGGTACAGGTGTAGTGACAGTTGCGTCGGCTGGAGTAGTAAATGAAT
                      ************ **************************** ******************

MpDN1                 CAATGTTCTTTGTAGTGGAAGCTTTGCCCATTTCTTTAGTTAGTCCCGAAGAGTGTGCAG
MpDV2                 CAATGTTCTTTGTAGTGGAAGCTTTGCCCATTTCTTTTGTTAGTCCCGAAGAGTGTGCAG
MpnDV                 CAATGTTCTTTGTAGTGGAAGCTTTGCCCATTTCTTTTGTTAGTCCCGAAGAGTGTGCAG
                      ************************************** *********************

MpDN1                 CGGCGTTTTGATTGTCAGCTACTTTTTCAGGAGCCACGTACAATCCGTGAACAATAGATA
MpDV2                 CGGCGTTTTGATTGTCAGCTACTTTTTCAGGAGCCACGTACAATCCGTGAACAATAGATA
MpnDV                 CGGCGTTTTGATTGTCAGCTACTTTTTCAGGAGCCACGTACAATCCGTGAACAATAGATA
                      ************************************************************

MpDN1                 AATCTCCACGATTGTATAGATTTTGATTGTAATTGTCGTCGGCCAGCAACCATGTCTTTT
MpDV2                 AATCTGCACGATTGTATAGATTTTGATTGTAATTGTCGTCAGCCAGCAACCATGTTTTTT
MpnDV                 AATCTGCACGATTGTATAGATTTTGATTGTAATTGTCGTCAGCCAGCAACCATGTCTTTT
                      ***** ********************************** ************** ****

MpDN1                 CGTATTCCACGTGAAGCCTGTCAAACGCTGTTCTGCTTTGCGGACATCCAAAACTAACTG
MpDV2                 CGTATTCCACATGAAGCCTGTCAAACGCTGTTCTGCTTTGCGGACATCCAAAACTAACTG
MpnDV                 CGTATTCCACATGAAGCCTGTCAAACGCTGTTCTGCTTTGCGGACATCCAAAACTAACTG
                      ********** *************************************************

MpDN1                 TCATCTCAGTTTCGAAATCCCATGTGCATTCTACGTCAGTAAAATTTACAGGGACTATAC
```

```
MpDV2           TCATCTCAGTTTCGAAATCCCATGTGCATTCTACATCAGTAAAATTTACAGGGACTATAC
MpnDV           TCATCTCAGTTTCGAAATCCCATGTGCATTCTACATCAGTAAAATTTACAGGAACTATAC
                ******************************** ******************* *******

MpDN1           TATTAGTGGCAGTAGTTAACTTATGCACTGGATATATTCCTACATGTACACTAGGTTGTA
MpDV2           TATTAGTGGCAGTAGTTAACTTATGCACTGGATATATTCCTACATGTACACTAGGTTGCA
MpnDV           TATTAGTGGCAGTAGTTAACTTATGCACTGGATATATTCCTACATGTACACTGGGTTGCA
                **************************************************** ***** *

MpDN1           CTTTATTAGAATATTCATGTTTATCGAACATACGAAAATATTGGCCTACGTCTATGTTAG
MpDV2           CTTTAGTAGAATATTCATGTTTATCGAACATACGAAAATATTGGCCTACGTCTATGTTAG
MpnDV           CTTTATTAGAATATTCATGTTTATCGAACATACGAAAATATTGTCCTACGTCTATGTTAG
                ***** ************************************* ****************

MpDN1           TACAATATCTATCATTGCCGAATATGCGTTTCCAACCGTTTTCACCCACAAATGTCAATT
MpDV2           TACAATATCTATCATTGCCAAATATGCGTTTCCAACCGTTTTCAGCTACATATGTTAATT
MpnDV           TACAATATCTATCATTGCCAAATATGCGTTTCCAACCGTTTTCAGCCACATATGTTAATT
                ******************* ************************ * *** **** ****

MpDN1           TTTGATCCGATCTCCTTTCGAACAAGCCACTGGCAGCGTTTATAAAATCTTGACACTCTG
MpDV2           TTTGATCCGATTTCCTTTCGAATAAGCCACTGGCAGCGTTTATAAAATCTTGACACTCTG
MpnDV           TTTGATCCGATCTCCTTTCGAATAAGCCACTGGCAGCGTTTATAAAATCTTGACACTCTG
                *********** ********** *************************************

MpDN1           GTTCGTTACCAGCATTGCGTAACATAGCAAATTTACTGGAATTAGGCTCCTGTACGCTGA
MpDV2           GTTCGTTACCAGCATTGCGTAACATAGCAAATTTACTAGAATTAGGCTCTTGTACGCTAA
MpnDV           GTTCGTTACCAGCATTGCGTAACATAGCAAATTTACTAGAATTAGGCTCCTGTACGCTGA
                ************************************* *********** ******** *

MpDN1           CACACGCATCGAATATTGGATCGTGTGGTAGAGTGAGTATACCGACATGAGGCTTGTACG
MpDV2           CACATGCATCGAATATTGGATCGTGTGGTAGAGTAAGTATACCAACATGAGGCTTGTACG
MpnDV           CACATGCATCGAATATTGGATCGTGTGGTAGAGTAAGTATTCCAACATGAGGCTTGTACG
                **** ***************************** ***** ** ****************

MpDN1           AATAATTAAGTACAGTGGTGCCGACAGTAAACGAAGCGTCGACCTTGGTGATGTGTTCAT
MpDV2           AATAATTAAGTATAGTGGTGCCGACAGTAAACGAAGCGTCAACCTTGGTGATGTGTTCAT
MpnDV           AATAATTAAGTACAGTGGTGCCGACAGTAAACGAAGCGTCAACCTTGGTGATGTGTTCAT
                ************ *************************** *******************

MpDN1           TAAACCGTTCCCATCCGAGTTTTCCAGAATTATGATTTCTATTTACAAAATTACAAAAAT
MpDV2           TAAATCGTTCCCATCCAAGTTTTCCAGAATTATGATTTCTATTTACAAAATTACAAAAAT
MpnDV           TAAATCGTTCCCATCCAAGTTTTCCAGAATTATGATTTCTATTTACAAAATTACAAAAAT
                **** *********** *******************************************

MpDN1           AACTGTTATACATCAACGGTAAGTTCATGTAGGACGCAGGCAGTTCATATCCCTCTTTAG
MpDV2           AACTGTTGTACATCAACGGTAAGTTCATGTAGGACGCAGGCAATTCATATCCCTCTTTAG
MpnDV           AACTGTTATACATCAACGGTAAGTTCATGTAGGACGCAGGCAGTTCATATCCCTCTTTAG
                ******* ********************************** *****************

MpDN1           CAGAGAATGTACCATCTAGATCTTGACCGTACAATTTGCGAACGAATTCTTTCATAGACG
MpDV2           CAGAGAATGAACCATCTAGATCTTGACCGTACAATTTGCGAACGAATTCTTTCATAGACG
MpnDV           CAGAGAATGAACCATCTAGATCTTGACCGTACAATTTGCGAACGAACTCTTTCATAGACG
                ********* ************************************ *************

MpDN1           ACTGATCGTTCTCGGTATTCGTTGGATCCATGGGCTCCGATGAATTGCCAAATGTGAGTT
MpDV2           ACTGATCGTTTTCGGTATTTGTTGGATCCATGGGCTCCGATGAATTACCAAATGTGAGTT
MpnDV           ATTGATCGTTTTCGGTATTTGTTGGATCCATGGGCTCCGATGAATTACCAAATGTGAGTT
                * ******** ******** ************************** *************

MpDN1           TTCGGTTAAATCCCCTTGTTTTTAAATTCAAAGCGTCCGCTTTTACCATAAATTTGTTCT
MpDV2           TTCGGTTAATTCCCCTTGTTTTTAAATTCAAAGCGTCCGCTTTTACCATAAATTTGTTCT
MpnDV           TTCGATTAAATCCCCTTGTTTTTAAATTCAAAGCGTCCGCTTTTACCATAAATTTGTTCT
                **** **** **************************************************

MpDN1           GATTCAATGTGGCCAGGGAGCTCGTAGAGCTATTGGTTTCAAAAGCTGTGCGAGGATTAC
MpDV2           GATTTAATGTGGCCAGGGAGCTCGTAGAGCTATTGGTTTCAAAAGCTGTGCGAGGATTAC
MpnDV           GATTTAATGTGGCCAGGGAGCTCGTAGAACTATTGGTTTCAAAAGCTGTGCGAGGATTAC
                **** *********************** *******************************

MpDN1           GCATGACACCCTTGCATTTGACTTCAGTGCATATAGCACCTCTGGGTAAAAATTTAAACT
MpDV2           GCATGACACCCTTGCATTTGACTTCAGTGCATATAGCGCCTCTGGGTAAAAATTTAAACT
MpnDV           GCATGACACCCTTGCATTTGACTTCAGTGCATATAGCACCTCTGGGTAAAAATTTAAACT
                ************************************* **********************

MpDN1           CGGACGGCGACATATAGAACCAAGGTTGGTCCACGGGCACACTCATTAAACTCGTTGTCC
MpDV2           CGGACGGCGACATATAGAACCAAGGTTGGTCCACAGGCACACTCATTAAACTCGTTGTCC
MpnDV           CGGACGGCGACATATAGAACCAAGGTTGGTCCACAGGCACACTCATTAAACTCGTTGTCC
                ********************************** *************************

MpDN1           CAAGATAGGCGTTATCAGTGCTAAGATATCGCAACACTTTCCAACCACATCCGTAACTGA
MpDV2           CAAGATAGGCGTTATCAGTGTTAAGATATCGCAACACTTTCCAACCACATCCGTAACTGA
MpnDV           CAAGATAGGCGTTATCAGTGTTAAGATATCGCAACACTTTCCAACCACATCCGTAACTGA
                ******************** ***************************************

MpDN1           TGAAAGTATGTACTTTTCGGTAAACCATAGTATATCCTCCCGTGTGACCGATTGGCCTAG
MpDV2           TGAAAGTATGTACTTTTCGGTAAACCATAGTATATCCTCCCGTGTGACCGATTGGCCTAG
MpnDV           TGAAAGTATGTACTTTTCGGTAAACCATAGTATATCCTCCCGTGTGACCGATTGGCCTAG
                ************************************************************
```

```
MpDN1             GTATAACAGCGTTTTCAGAGCTAGGATTGCCAGTATCTGGGTCCCCATCGTTCGCAGCAG
MpDV2             GTATAACAGCGTTTTCAGCGCTAGGATTGCCAGTATCTGGGTCCCCATCGTTCGCAGCAG
MpnDV             GTATAACAGCGTTTTCAGAGCTAGGATTGCCGGTATCTGGGTCCCCATCGTTCGCAGCAG
                  ****************** ************ ****************************

MpDN1             CCGTCCCAGGAAGCGAGGTGCCACCCGCACGTTTAGGAGCGGGTTCAGAACCGGAGCTAT
MpDV2             CCGTCCCAGGAAGCGAGGTGCCACCCGCGCGTTTAGGAGCGGGTTCAGAACCGGAGCTAT
MpnDV             CCGTCCCAGGAAGCGAGGTGCCACCCGCACGTTTAGGAGCGGGTTCAGAACCGGAGCTAT
                  **************************** *******************************

MpDN1             CGGCAGGTCGTTTGCTACTTGATCCGCTAGAGCTTGCCATGGATCCCGATGCGCTTCCCC AUG codon of N-truncated VP1
MpDV2             CGGCAGGTCGTTTGCTACCTGATCCGCTAGAGCTTGCCATGGATCCCGATGCGCTTCCCC translated from spliced mRNA
MpnDV             CGGCAGGTCGTTTGCTACCTGATCCGCTAGAGCTTGCCATGGATCCCGATGAGCTTCCCC
                  ****************** ******************************** ********

MpDN1             CGTTGCCGTTGCTAGTGCTGTTGTCCGGGGCTGAGGTGTTTGCCACGTGGTCGCCACTCG
MpDV2             CGTTGCCGTTGCTAGTGATGTTGTCCGGGGCTGAGGTGTTTGCCACGTGATCGCCACTCG
MpnDV             CGTTGCCGTTGCTAGTGCTGTTGTCCGGGGCTGAGGTGTTTGCCACGTGGTCGCCACTCG
                  ***************** ******************************* **********

MpDN1             CTTCACCGCTGTTAGGATTGCCGTTACCGTCGTAGTCCCAATCCAAATCAGGATCCTCCT
MpDV2             CTTCACCGCTGTTAGGATTGCCGTTACCGTCGTAGTCCCAATCCAAATCAGGATCCTCCT
MpnDV             CTTCACCGCTGTTAGGATTGCCGTTACCGTCGTAGTCCCAATCCAAATCAGGATCCTCCT
                  ************************************************************

MpDN1             CCACGCCACCTAAAACATACGGTGGTAATGAACGACGTGCTAAAGCCTTATTGTATTGTT 3′ splice site of VP intron 1
MpDV2             CCACGCCACCTAAAACATACGGTGGTAATGAACGACGTGCTAAAGCCTTATTGTATTGTT
MpnDV             CCACGCCACCTAAAACATACGGTGGTAATGAACGACGTGCTAAAGCCTTATTGTATTGCT
                  ********************************************************** *

MpDN1             CTAGCGCGTAACGCTGCTGCGATGAATTTAAATTTTGCCAATTGGCCCGCTCCGGTGGAG
MpDV2             CTAGCGAATAACGCTGCTGCGATGAATTTAGATTTTGCCAATTGGGCCGCTCCGGTGGAG
MpnDV             CTAGCGAATAACGCTGCTGCGATGAATTTAGATTTTGCCAATTGGGCCGCTCCGGTGGAG
                  ******  ********************** ************** **************

MpDN1             GAGGACCTAAATAACGAAATTCTCGCATACTTACGGCTACTGTCTGCTGCCGCTAAATAC AUG codon of VP1 ORF
MpDV2             GAGGACCTAAATAACGAAATTCTCGCATACTTACGGCTACTGTCTGCTGCCGCTAAATAC 5′ splice site of VP intron 1
MpnDV             GAGGACCTAAATAACGAAATTCTCGCATACTTACGGCTACTGTCTGCTGCCGCTAAATAC
                  ************************************************************

MpDN1             GGTGTTGTATCAAAATATGTTCCGCTAGTGTTAGGTTTGTTCGGGTATATTGCTGTCTCG
MpDV2             GGTGTTGTATCGAAATATATTCCGCTAGTGTTAGGTTTGTTCGGATATATTGCTGTCTCG
MpnDV             GGTGTTGTATCGAAATATATTCCGCTAGTGTTAGGTTTGTTCGGGTATATTGCTGTCTCG
                  *********** ****** ************************* ***************

MpDN1             CTGCCTGTCAAGAACTGATCGGCTAAATCGACACCACGCTTTGCGATCAATCCGAGAGCT
MpDV2             CTGCCTGTCAAGAACTGATCGGCCAAATCGACACCACGCTTTGCAATCAATCCAAGAGCT
MpnDV             CTGCCTGTCAAGAACTGATCGGCCAAATCGACACCACGCTTTGCAATCAATCCAAGAGCT
                  *********************** ******************** ******** ******

MpDN1             GTCGCAATGTTGCCATAATATGGGTCGCTGACTTGCAGCGCTCCCCGTATCGCTTTTTTG
MpDV2             GTTGCAATGTTGCCATAATATGGGTCGCTGACTTGCAGCGCTCCCCGTATCGCTTTTTTG
MpnDV             GTTGCAATGTTGCCATAATATGGGTCGCTGACTTGCAGCGCTCCCCGTATCGCTTTTTTG
                  ** *********************************************************

MpDN1             TCTAATTCCCATATGTTATCCTTGCTAACATCCGTAGCATTAAAATAATCGGCATCGTGT
MpDV2             TCTAATTCCCATATGTTATCCTTGCTAACATCCGTAGCATTAAAATAATCGGCATCGTGT
MpnDV             TCTAATTCCCATATGTTATCCTTGCTAACATCCGTAGCATTAAAATAATCGGCATCGTGT
                  ************************************************************

MpDN1             TGAAATGCGACCTTATCTATATCACTAGTAGGTAACGCCTTAGCATTAAAATGACCTTCA
MpDV2             TGAAATGCAACCTTATCTATATCACTAGTAGGTAACGCCTTAGCATTAAAATGACCTTCA
MpnDV             TGAAATGCAACCTTATCTATATCACTAGTAGGTAACGCCTTAGCATTAAAATGACCTTCA
                  ******** ***************************************************

MpDN1             CTGTCACACAACAGATTACCTGGACCCACGAAGCTACTACCGAACCACGTGATACCTTCA
MpDV2             CTGTCACACAATAAATTTCCTGGACCCACGAAGCTACTACCGAACCACGTGATACCTTCA
MpnDV             CTGTCACACAACAAATTACCTGGACCCACGAAGCTACTACCAAACCACGTGATACCTTTA
                  *********** * *** *********************** **************** *

MpDN1             TTGCTAGTACCTGAAGCACCTAATTTAATACGTTTTCTCTCGTTATTCTCCTCTTCAACA
MpDV2             TTGCTAGTACCTGAAGTACCTAATTTAATACGTTTTCTCTCATTATTCTCCTCTTCAACA
MpnDV             TTGCTAGTACCTGAAGTACCTAATTTAATACGTTTTCTCTCATTATTCTCCTCTTCAACA
                  **************** ************************ ******************

MpDN1             CTATAAAATTCGTCGTTTACCGACGACATGTTAAACGTAACTGATTAAATATAAGTAAAA AUG codon of VP2 ORF
MpDV2             CTATAAAATTCGTCGTTTACCGACGACATGTTGAACGTAACTGATTAAATATAAGTAAAA
MpnDV             CTATAAAATTCGTCGTTTACCGACGACATGTTGAACGTAACTGATTAAATATAAGTAAAA
                  ******************************** ***************************

MpDN1             TGTAAACACTTGAGTAAATAGTGATGACGTCACCCCCACCTAGTCGCCTACTGGATACTT
MpDV2             TGTAAACACTTGAGTAAATAGTGATGACGTCACCCCCACCTAGTCGCCTGCTGGATACTT
MpnDV             TGTAAACACTTGAGTAAATAGTGATGACGTCACCCCCACCTAGTCGCCTGCTGGATACTT
                  ************************************************* **********

MpDN1             GTCTATCTTTTTATTATATAAAAAGATCCCCTACTAATAATAACCACTACCTACTTCCGG 3′ITR-based TATA box
MpDV2             GTCTATCTTTTTATTATATAAAAAGATCCCCTAC---TAATACCCACTACCTACTTCCGG
```

```
MpnDV           GTCTATCTTTTTATTATATAAAAAGATCCCCTAA---TAATAACCACTACCTACTTCC--
                *********************************    ***** ***************

MpDN1           GGGGCGGGGGGTATCCTTTGTTATCTATACTTATTGTCAACACTAG-CCTTATCTTAGGG
MpDV2           GGGGCGGGGGGTATCCTTTGTTATCTATACTTATTGTCAACACTAGCCCTTATCTTAGGG
MpnDV           ------------------------------------------------------------


MpDN1           CGTGTCGGCCGACACGTGTCATGGCCATGACACTAGCCCTAAGATAAGG-CTAGTGTTGAC
MpDV2           CGTGTCGGCCGACACGTGTCATGGCCATGACACT-GGCCTAAGATAAGGGCTAGTGTTGAC
MpnDV           -------------------------------------------------------------


MpDN1           AATAAGTATAGATAACAAGGATACCCCCCGCCCCCCGGAAGTAGGTAGTGGTTATTATTA
MpDV2           A-----------------------------------------------------------
MpnDV           ------------------------------------------------------------


MpDN1           GTAGGGGATCTTTTTATAT
MpDV2           -------------------
MpnDV           -------------------
```

(**d**) **Multiple sequence alignment of Trinity contigs representing MpDV1 mRNAs.** The contigs were assembled from Illumina mRNA-seq reads obtained from *M. persicae* aphids fed on *A. thaliana* mock-inoculated (APFV-5-6-7) or TuYV-infected plants (APFV-11-12-13) or on artificial diets with TuYV virions (APFV-8-9-10) or without virions (APFV-14-15-16).

APFV-5-6-7 (A. thaliana mock-inoculated)

```
MpDNV_mRNA_seq_consensus      ATAATAAAAAGATAGACAAGTATCCAGTAGGCGACTAGGTGGGGGTGACGTCATCACTAT
TRINITY_DN2_c0_g2_i4  APFV-7  ---------------------------------------------------CATCACTAT NS 5'-end
TRINITY_DN2_c0_g2_i2  APFV-5  ----------------------------------------------------------AT NS 5'-end
TRINITY_DN2_c0_g2_i1  APFV-5  ----------------------------------------------------------AT NS 5'-end
TRINITY_DN9_c0_g1_i1  APFV-6  ------------------------------------------------------------
TRINITY_DN9_c0_g1_i2  APFV-6  ------------------------------------------------------------

MpDNV_mRNA_seq_consensus      TTACTCAAGTGTTTTGTTATTCGGCGTCAAGTGTTGTGTTGACACTTGAAAAGTGGCGTG
TRINITY_DN2_c0_g2_i4          TTACTCAAGTGTTTTGTTATTCGGCGTCAAGTGTTGTGTTGACACTTGAAAAGTGGCGTG
TRINITY_DN2_c0_g2_i2          TTACTCAAGTGTTTTGTTATTCGGCGTCAAGTGTTGTGTTGACACTTGAAAAGTGGCGTG
TRINITY_DN2_c0_g2_i1          TTACTCAAGTGTTTTGTTATTCGGCGTCAAGTGTTGTGTTGACACTTGAAAAGTGGCGTG
TRINITY_DN9_c0_g1_i1          ------------------------------------------------------------
TRINITY_DN9_c0_g1_i2          ------------------------------------------------------------

MpDNV_mRNA_seq_consensus      ATTGATTATTTACAAGTACGTCAGATAAGATAAACATAAGTGGTACTTGACTCGGAATAT 5'ITR TATA-box
TRINITY_DN2_c0_g2_i4          ATTGATTATTTACAAGTACGTCAGATAAGATAAACATAAGTGGTACTTGACTCGGAATAT
TRINITY_DN2_c0_g2_i2          ATTGATTATCTACAAGTACGTCAGATAAGATAAACATAAGTGGTACTTGACTCGGAATAT
TRINITY_DN2_c0_g2_i1          ATTGATTATCTACAAGTACGTCAGATAAGATAAACATAAGTGGTACTTGACTCGGAATAT
TRINITY_DN9_c0_g1_i1          ------------------------------------------------------------
TRINITY_DN9_c0_g1_i2          ------------------------------------------------------------

MpDNV_mRNA_seq_consensus      ATAATGAAGTGTGGAACCTACAACAATATCAGTACTCATCAAAATGTCAAACTCGCAAGA
TRINITY_DN2_c0_g2_i4          ATAATGAAGTGTGGAACCTACAACAATGTCAGTACTCATCAAAATGTCAAACTCGCAAGA
TRINITY_DN2_c0_g2_i2          ATAATGAAGTGTGGAACCTACAACAATATCAGTACTCATCAAAATGTCAAACTCGCAAGA
TRINITY_DN2_c0_g2_i1          ATAATGAAGTGTGGAACCTACAACAATATCAGTACTCATCAAAATGTCAAACTCGCAAGA
TRINITY_DN9_c0_g1_i1          -------AGTGTGGAACCTACAACAATGTCAGTACTCATCAAAATGTCAAACTCGCAAGA NS 5'-end
TRINITY_DN9_c0_g1_i2          -------AGTGTGGAACCTACAACAATGTCAGTACTCATCAAAATGTCAAACTCGCAAGA NS 5'-end
                                     ******************** ********************************

MpDNV_mRNA_seq_consensus      AGTGAACCAACAACAATTCAACGACGAGGAGGAAATACCATGTGGCCAACGGTATCCTAC
TRINITY_DN2_c0_g2_i4          AGTGAACCAACAACAATTCAACGACGAGGAGGAAATACCATGTGGCCAACGGTATCCTAC
TRINITY_DN2_c0_g2_i2          AGTGAACCAACAACAATTCAACGACGAGGAGGAAATACCATGTGGCCAACGGTATCCTAC
TRINITY_DN2_c0_g2_i1          AGTGAACCAACAACAATTCAACGACGAGGAGGAAATACCATGTGGCCAACGGTATCCTAC
TRINITY_DN9_c0_g1_i1          AGTGAACCAACAACAATTCAACGACGAGGAGGAAATACCATGTGGCCAACGGTATCCTAC
TRINITY_DN9_c0_g1_i2          AGTGAACCAACAACAATTCAACGACGAGGAGGAAATACCATGTGGCCAACGGTATCCTAC
                              ************************************************************

MpDNV_mRNA_seq_consensus      AGCGGCGACGATTCAAACGGACCACCAACAGACTGGCGTGAATGGATCGACCCCGATGTT
TRINITY_DN2_c0_g2_i4          AGCGGCGACGATTCAAACGGACCACCAACAGACTGGCGTGAATGGATCGACCCCGATGTT
TRINITY_DN2_c0_g2_i2          AGCGGCGACGATTCAAACGGACCACCAACAGAGTGGCGTGAATGGATCGACCCCGATGTT
TRINITY_DN2_c0_g2_i1          AGCGGCGACGATTCAAACGGACCACCAACAGAGTGGCGTGAATGGATCGACCCCGATGTT
TRINITY_DN9_c0_g1_i1          AGCGGCGACGATTCAAACGGACCACCAACAGAGTGGCGTGAATGGATCGACCCCGATGTT
TRINITY_DN9_c0_g1_i2          AGCGGCGACGATTCAAACGGACCACCAACAGAGTGGCGTGAATGGATCGACCCCGATGTT
                              ******************************** ***************************

MpDNV_mRNA_seq_consensus      CGAACTCAAATCGACAGCGCGCAAAGAATATGGGACAGCCATTTCAGCGGACACGGCACT
TRINITY_DN2_c0_g2_i4          CGAACTCAAATCGACAGCGCGCAAAGAATATGGGACAGCCATTTCAGCGGACACGGCACT
TRINITY_DN2_c0_g2_i2          CGAACTCAAATCGACAGCGCGCAAAGAATATGGGACAGCCATTTCAGCGGACACGGCACT
TRINITY_DN2_c0_g2_i1          CGAACTCAAATCGACAGCGCGCAAAGAATATGGGACAGCCATTTCAGCGGACACGGCACT
TRINITY_DN9_c0_g1_i1          CGAACTCAAATCGACAGCGCGCAAAGAATATGGGACAGCCATTTCAGCGGACACGGCACT
TRINITY_DN9_c0_g1_i2          CGAACTCAAATCGACAGCGCGCAAAGAATATGGGACAGCCATTTCAGCGGACACGGCACT
                              ************************************************************

MpDNV_mRNA_seq_consensus      GACACTGAAGACGCTGTGCAAAGTGTTGGAGTGCAAGGCAACGGAACTAACGGTAACATG
TRINITY_DN2_c0_g2_i4          GACACTGAAGACGCTGTGCAAAGTGTTGGAGTGCAAGGCAACGGAACTAACGGTAACATG
TRINITY_DN2_c0_g2_i2          GACACTGAAGACGCTGTGCAAAGTGTTGGAGTGCAAGGCAACGGAACTAACGGTAACATG
TRINITY_DN2_c0_g2_i1          GACACTGAAGACGCTGTGCAAAGTGTTGGAGTGCAAGGCAACGGAACTAACGGTAACATG
```

```
TRINITY_DN9_c0_g1_i1          GACACTGAAGACGCTGTGCAAAGTGTTGGAGTGCAAGGCAACGGAACTAACGGTAACATG
TRINITY_DN9_c0_g1_i2          GACACTGAAGACGCTGTGCAAAGTGTTGGAGTGCAAGGCAACGGAACTAACGGTAACATG
                              ************************************************************

MpDNV_mRNA_seq_consensus      CATTATTCCTCTGATCGCGTTGCTATTACCCGACCATATGTGGTCAGAGGCGACGAGCAG
TRINITY_DN2_c0_g2_i4          CATTATTCCTCTGATCGCGTTGCTATTACCCGACCATATGTGGTCAGAGGCGACGAGCAG
TRINITY_DN2_c0_g2_i2          CATTATTCCTCTGATCGCGTTGCTATTACCCGACCATATGTGGTCAGAGGCGACGAGCAG
TRINITY_DN2_c0_g2_i1          CATTATTCCTCTGATCGCGTTGCTATTACCCGACCATATGTGGTCAGAGGCGACGAGCAG
TRINITY_DN9_c0_g1_i1          CATTATTCCTCTGATCGCGTTGCTATTACCCGACCATATGTGGTCAGAGGCGACGAGCAG
TRINITY_DN9_c0_g1_i2          CATTATTCCTCTGATCGCGTTGCTATTACCCGACCATATGTGGTCAGAGGCGACGAGCAG
                              ************************************************************

MpDNV_mRNA_seq_consensus      CGATCGCTGGCGATACCGACTGAGAAACGCGACCTACTGGATTTCCAATCCAACGCGGGA
TRINITY_DN2_c0_g2_i4          CGATCGCTGGCGATACCGACTGAGAAACGCGACCTACTGGATTTCCAATCCAACGCGGGA
TRINITY_DN2_c0_g2_i2          CGATCGCTGGCGATACCGACTGAGAAACGCGACCTACTGGATTTCCAATCCAACGCGGGA
TRINITY_DN2_c0_g2_i1          CGATCGCTGGCGATACCGACTGAGAAACGCGACCTACTGGATTTCCAATCCAACGCGGGA
TRINITY_DN9_c0_g1_i1          CGATCGCTGGCGATACCGACTGAGAAACGCGACCTACTGGATTTCCAATCCAACGCGGGA
TRINITY_DN9_c0_g1_i2          CGATCGCTGGCGATACCGACTGAGAAACGCGACCTACTGGATTTCCAATCCAACGCGGGA
                              ************************************************************

MpDNV_mRNA_seq_consensus      ATCCGAATATCAGGCTTTACTACGATGGATGGAGGAGAAAATGCCGATACCTTTAGCGAA
TRINITY_DN2_c0_g2_i4          ATCCGAATATCAGGCTTTACTACGATGGATGGAGGAGAAAATGCCGATACCTTTAGCGAA
TRINITY_DN2_c0_g2_i2          ATCCGAATATCAGGCTTTACTACGATGGATGGAGGAGAAAATGCCGATACCTTTAGCGAA
TRINITY_DN2_c0_g2_i1          ATCCGAATATCAGGCTTTACTACGATGGATGGAGGAGAAAATGCCGATACCTTTAGCGAA
TRINITY_DN9_c0_g1_i1          ATCCGAATATCAGGCTTTACTACGATGGATGGAGGAGAAAATGCCGATACCTTTAGCGAA
TRINITY_DN9_c0_g1_i2          ATCCGAATATCAGGCTTTACTACGATGGATGGAGGAGAAAATGCCGATACCTTTAGCGAA
                              ************************************************************

MpDNV_mRNA_seq_consensus      ATGGCAAGGTGCATGTCTACCGCGGTATGGGGAGGCAATGTCAGATATGTTTCTGACATC
TRINITY_DN2_c0_g2_i4          ATGGCAAGGTGCATGTCTACCGCGGTATGGGGAGGCAATGTCAGATATGTTTCTGACATC
TRINITY_DN2_c0_g2_i2          ATGGCAAGGTGCATGTCTACCGCGGTATGGGGAGGCAATGTCAGATATGTTTCTGACATC
TRINITY_DN2_c0_g2_i1          ATGGCAAGGTGCATGTCTACCGCGGTATGGGGAGGCAATGTCAGATATGTTTCTGACATC
TRINITY_DN9_c0_g1_i1          ATGGCAAGGTGCATGTCTACCGCGGTATGGGGAGGCAATGTCAGATATGTTTCTGACATC
TRINITY_DN9_c0_g1_i2          ATGGCAAGGTGCATGTCTACCGCGGTATGGGGAGGCAATGTCAGATATGTTTCTGACATC
                              ************************************************************

MpDNV_mRNA_seq_consensus      CTTGTACCCGGAGGAAATTGGTCAATTAATGGAATTGTTGAGGACCTACGCCGGAGTATC
TRINITY_DN2_c0_g2_i4          CTTGTACCCGGAGGAAATTGGTCAATTAATGGAATTGTTGAGGACCTACGCCGGAGTATC
TRINITY_DN2_c0_g2_i2          CTTGTACCCGGAGGAAATTGGTCAATTAATGGAATTGTTGAGGACCTACGCCGGAGTATC
TRINITY_DN2_c0_g2_i1          CTTGTACCCGGAGGAAATTGGTCAATTAATGGAATTGTTGAGGACCTACGCCGGAGTATC
TRINITY_DN9_c0_g1_i1          CTTGTACCCGGAGGAAATTGGTCAATTAATGGAATTGTTGAGGACCTACGCCGGAGTATC
TRINITY_DN9_c0_g1_i2          CTTGTACCCGGAGGAAATTGGTCAATTAATGGAATTGTTGAGGACCTACGCCGGAGTATC
                              ************************************************************

MpDNV_mRNA_seq_consensus      GCAGAGCGAATGCCAAAATGCATGTACGTCGTCAGCGAACACGGCGACCACGTCCACGTC
TRINITY_DN2_c0_g2_i4          GCAGAGCGAATGCCAAAATGCATGTACGTCGTCAGCGAACACGGCGACCACGTCCACGTC
TRINITY_DN2_c0_g2_i2          GCAGAGCGAATGCCAAAATGCATGTACGTCGTCAGCGAACACGGCGACCACGTCCACGTC
TRINITY_DN2_c0_g2_i1          GCAGAGCGAATGCCAAAATGCATGTACGTCGTCAGCGAACACGGCGACCACGTCCACGTC
TRINITY_DN9_c0_g1_i1          GCAGAGCGAATGCCAAAATGCATGTACGTCGTCAGCGAACACGGCGACCACGTCCACGTC
TRINITY_DN9_c0_g1_i2          GCAGAGCGAATGCCAAAATGCATGTACGTCGTCAGCGAACACGGCGACCACGTCCACGTC
                              ************************************************************

MpDNV_mRNA_seq_consensus      GTACACACCTGTAACTACGCCACGGGCAGCTGTAGATGTAGTTTCTTACTCAACGCCGCT
TRINITY_DN2_c0_g2_i4          GTACACACCTGTAACTACGCCACGGGCAGCTGTAGATGTAGTTTCTTACTCAACGCCGCT
TRINITY_DN2_c0_g2_i2          GTACACACCTGTAACTACGCCACGGGCAGCTGTAGATGTAGTTTCTTACTCAACGCCGCT
TRINITY_DN2_c0_g2_i1          GTACACACCTGTAACTACGCCACGGGCAGCTGTAGATGTAGTTTCTTACTCAACGCCGCT
TRINITY_DN9_c0_g1_i1          GTACACACCTGTAACTACGCCACGGGCAGCTGTAGATGTAGTTTCTTACTCAACGCCGCT
TRINITY_DN9_c0_g1_i2          GTACACACCTGTAACTACGCCACGGGCAGCTGTAGATGTAGTTTCTTACTCAACGCCGCT
                              ************************************************************

MpDNV_mRNA_seq_consensus      GCTTTCCAACTATGTAGACGACGACGCATTCGAAAGAATGTTAGAGTCATCCAACTATCA
TRINITY_DN2_c0_g2_i4          GCTTTCCAACTATGTAGACGACGACGCATTCGAAAGAATGTTAGAGTCATCCAACTATCA
TRINITY_DN2_c0_g2_i2          GCTTTCCAACTATGTAGACGACGACGCATTCGAAAGAATGTTAGAGTCATCCAACTATCA
TRINITY_DN2_c0_g2_i1          GCTTTCCAACTATGTAGACGACGACGCATTCGAAAGAATGTTAGAGTCATCCAACTATCA
TRINITY_DN9_c0_g1_i1          GCTTTCCAACTATGTAGACGACGACGCATTCGAAAGAATGTTAGAGTCATCCAACTATCA
TRINITY_DN9_c0_g1_i2          GCTTTCCAACTATGTAGACGACGACGCATTCGAAAGAATGTTAGAGTCATCCAACTATCA
                              ************************************************************

MpDNV_mRNA_seq_consensus      GAAAGAGACTGGTCCCGTATCTTTCAATACTTATGTTCATCGCCCAGGAATGTCAAAGAA
TRINITY_DN2_c0_g2_i4          GAAAGAGACTGGTCCCGTATCTTTCAATACTTATGTTCATCGCCCAGGAATGTCAAAGAA
TRINITY_DN2_c0_g2_i2          GAAAGAGACTGGTCCCGTATCTTTCAATACTTATGTTCATCGCCCAGGAATGTCAAAGAA
TRINITY_DN2_c0_g2_i1          GAAAGAGACTGGTCCCGTATCTTTCAATACTTATGTTCATCGCCCAGGAATGTCAAAGAA
TRINITY_DN9_c0_g1_i1          GAAAGAGACTGGTCCCGTATCTTTCAATACTTATGTTCATCGCCCAGGAATGTCAAAGAA
TRINITY_DN9_c0_g1_i2          GAAAGAGACTGGTCCCGTATCTTTCAATACTTATGTTCATCGCCCAGGAATGTCAAAGAA
                              ************************************************************

MpDNV_mRNA_seq_consensus      GTGGGCGGCCAGTACTTTGATGGAGGATTACGTAGTAGATATTCACATATATCAGTTAGT 5′ splice site
TRINITY_DN2_c0_g2_i4   APFV-7 GTGGGCGGCCAGTACTTTGATGGAGGATTACGTAGTAGATATTCACATATATCA------ spliced intron
TRINITY_DN2_c0_g2_i2   APFV-5 GTGGGCGGCCAGTACTTTGATGGAGGATTACGTAGTAGATATTCACATATATCAGTTAGT unspliced
TRINITY_DN2_c0_g2_i1   APFV-5 GTGGGCGGCCAGTACTTTGATGGAGGATTACGTAGTAGATATTCACATATATCA------ spliced intron
TRINITY_DN9_c0_g1_i1   APFV-6 GTGGGCGGCCAGTACTTTGATGGAGGATTACGTAGTAGATATTCACATATATCAGTTAGT unspliced
TRINITY_DN9_c0_g1_i2   APFV-6 GTGGGCGGCCAGTACTTTGATGGAGGATTACGTAGTAGATATTCACATATATCA------ spliced intron
                              ******************************************************

MpDNV_mRNA_seq_consensus      ATTGCGTAATTTTTTTATATACTTGGATTGGTTGACGGGTATAGAAGCGAATAGATGCGT
TRINITY_DN2_c0_g2_i4          ------------------------------------------------------------
TRINITY_DN2_c0_g2_i2          ATTGCGTAATTTTTTTATATACTTGGATTGGTTGACGGGTATAGAAGCGAATAGATGCGT
TRINITY_DN2_c0_g2_i1          ------------------------------------------------------------
```

```
TRINITY_DN9_c0_g1_i1          ATTGCGTAATTTTTTATATACTTGGATTGGTTGACGGGTATAGAAGCGAATAGATGCGT
TRINITY_DN9_c0_g1_i2          ------------------------------------------------------------

MpDNV_mRNA_seq_consensus      TTCATATATTAAAACTGGTATGCCTGGCAGGAGTTACGTTAAAAGCGTGATGCCAAAAAA
TRINITY_DN2_c0_g2_i4          ------------------------------------------------------------
TRINITY_DN2_c0_g2_i2          TTCATATATTAAAACTGGTATGCCTGGCAGGAGTTACGTTAAAAGCGTGATGCCAAAAAA
TRINITY_DN2_c0_g2_i1          ------------------------------------------------------------
TRINITY_DN9_c0_g1_i1          TTCATATATTAAAACTGGTATGCCTGGCAGGAGTTACGTTAAAAGCGTGATGCCAAAAAA
TRINITY_DN9_c0_g1_i2          ------------------------------------------------------------

MpDNV_mRNA_seq_consensus      TTATTGGCGTAATCCTAAAGAATTTCACTTAGCGCTTGATAAATTAGAGTTGCTCGTTAA
TRINITY_DN2_c0_g2_i4          ------------------------------------------------------------
TRINITY_DN2_c0_g2_i2          TTATTGGCGTAATCCTAAAGAATTTCACTTAGCGCTTGATAAATTAGAGTTGCTCGTTAA
TRINITY_DN2_c0_g2_i1          ------------------------------------------------------------
TRINITY_DN9_c0_g1_i1          TTATTGGCGTAATCCTAAAGAATTTCACTTAGCGCTTGATAAATTAGAGTTGCTCGTTAA
TRINITY_DN9_c0_g1_i2          ------------------------------------------------------------

MpDNV_mRNA_seq_consensus      ACGACATTTCGGTGATTTCACGTGTTTGGAAAATTTTTTACCGCAATATCGTTGGCATAA
TRINITY_DN2_c0_g2_i4          ------------------------------------------------------------
TRINITY_DN2_c0_g2_i2          ACGACATTTCGGTGATTTCACGTGTTTGGAAAATTTTTTACCGCAATATCGTTGGCATAA
TRINITY_DN2_c0_g2_i1          ------------------------------------------------------------
TRINITY_DN9_c0_g1_i1          ACGACATTTCGGTGATTTCACGTGTTTGGAAAATTTTTTACCGCAATATCGTTGGCATAA
TRINITY_DN9_c0_g1_i2          ------------------------------------------------------------

MpDNV_mRNA_seq_consensus      TTGCATCACATGGTTTGTGAACGTGATGTCCTGTCCGTTGGACCCGTGGACTGTCGAATG
TRINITY_DN2_c0_g2_i4          ------------------------------------------------------------
TRINITY_DN2_c0_g2_i2          TTGCATCACATGGTTTGTGAACGTGATGTCCTGTCCGTTGGACCCGTGGACTGTCGAATG
TRINITY_DN2_c0_g2_i1          ------------------------------------------------------------
TRINITY_DN9_c0_g1_i1          TTGCATCACATGGTTTGTGAACGTGATGTCCTGTCCGTTGGACCCGTGGACTGTCGAATG
TRINITY_DN9_c0_g1_i2          ------------------------------------------------------------

MpDNV_mRNA_seq_consensus      TGCTGGTTGTGGTTTTCAACATATTCCTAATATATCTAAATGCGAACTACTACGAGACTA
TRINITY_DN2_c0_g2_i4          ------------------------------------------------------------
TRINITY_DN2_c0_g2_i2          TGCTGGTTGTGGTTTTCAACATATTCCTAATATATCTAAATGCGAACTACTACGAGACTA
TRINITY_DN2_c0_g2_i1          ------------------------------------------------------------
TRINITY_DN9_c0_g1_i1          TGCTGGTTGTGGTTTTCAACATATTCCTAATATATCTAAATGCGAACTACTACGAGACTA
TRINITY_DN9_c0_g1_i2          ------------------------------------------------------------

MpDNV_mRNA_seq_consensus      TCCGTTCATGAACGAGGGCACTATAAATCATATACTAATCGAATGCATTAAAATTTACAA
TRINITY_DN2_c0_g2_i4          ------------------------------------------------------------
TRINITY_DN2_c0_g2_i2          TCCGTTCATGAACGAGGGCACTATAAATCATATACTAATCGAATGCATTAAAATTTACAA
TRINITY_DN2_c0_g2_i1          ------------------------------------------------------------
TRINITY_DN9_c0_g1_i1          TCCGTTCATGAACGAGGGCACTATAAATCATATACTAATCGAATGCATTAAAATTTACAA
TRINITY_DN9_c0_g1_i2          ------------------------------------------------------------

MpDNV_mRNA_seq_consensus      ACAGGAAGGATCAAATACAGAATGTCGACCCGAACGATTGGTGGAAACATGCCTTCTGCA 3' splice site
TRINITY_DN2_c0_g2_i4          ----GAAGGATCAAATACAGAATGTCGACCCGAACGATTGGTGGAAACATGCCTTCTGCA spliced intron
TRINITY_DN2_c0_g2_i2          ACAGGAAGGATCAAATACAGAATGTCGACCCGAACGATTGGTGGAAACATGCCTTCTGCA unspliced
TRINITY_DN2_c0_g2_i1          ----GAAGGATCAAATACAGAATGTCGACCCGAACGATTGGTGGAAACATGCCTTCTGCA spliced intron
TRINITY_DN9_c0_g1_i1          ACAGGAAGGATCAAATACAGAATGTCGACCCGAACGATTGGTGGAAACATGCCTTCTGCA unspliced
TRINITY_DN9_c0_g1_i2          ----GAAGGATCAAATACAGAATGTCGACCCGAACGATTGGTGGAAACATGCCTTCTGCA spliced intron
                                  ********************************************************

MpDNV_mRNA_seq_consensus      GGGCTCGGGGGAGCTTCAATCTGAACAGTCCATACGTGAAAATGGCCAAAACCATACAAC
TRINITY_DN2_c0_g2_i4          GGGCTCGGGGGAGCTTCAATCTGAACAGTCCATACGTGAAAATGGCCAAAACCATACAAC
TRINITY_DN2_c0_g2_i2          GGGCTCGGGGGAGCTTCAATCTGAACAGTCCATACGTGAAAATGGCCAAAACCATACAAC
TRINITY_DN2_c0_g2_i1          GGGCTCGGGGGAGCTTCAATCTGAACAGTCCATACGTGAAAATGGCCAAAACCATACAAC
TRINITY_DN9_c0_g1_i1          GGGCTCGGGGGAGCTTCAATCTGAACAGTCCATACGTGAAAATGGCCAAAACCATACAAC
TRINITY_DN9_c0_g1_i2          GGGCTCGGGGGAGCTTCAATCTGAACAGTCCATACGTGAAAATGGCCAAAACCATACAAC
                              ************************************************************

MpDNV_mRNA_seq_consensus      AGGAAGTGTTTCGCAGGATGACCAAGATGGAGTACAAGATAACGGATCCACAAATGTTTT
TRINITY_DN2_c0_g2_i4          AGGAAGTGTTTCGCAGGATGACCAAGATGGAGTACAAGATAACGGATCCACAAATGTTTT
TRINITY_DN2_c0_g2_i2          AGGAAGTGTTTCGCAGGATGACCAAGATGGAGTACAAGATAACGGATCCACAAATGTTTT
TRINITY_DN2_c0_g2_i1          AGGAAGTGTTTCGCAGGATGACCAAGATGGAGTACAAGATAACGGATCCACAAATGTTTT
TRINITY_DN9_c0_g1_i1          AGGAAGTGTTTCGCAGGATGACCAAGATGGAGTACAAGATAACGGATCCACAAATGTTTT
TRINITY_DN9_c0_g1_i2          AGGAAGTGTTTCGCAGGATGACCAAGATGGAGTACAAGATAACGGATCCACAAATGTTTT
                              ************************************************************

MpDNV_mRNA_seq_consensus      TCAAGAAGAAGAAGAGGGCAGCCAAGAGGGCCCGCGGCAAAAGAAGTCGCGACGGAAGCA
TRINITY_DN2_c0_g2_i4          TCAAGAAGAAGAAGAGGGCAGCCAAGAGGGCCCGCGGCAAAAGAAGTCGCGACGGAAGCA
TRINITY_DN2_c0_g2_i2          TCAAGAAGAAGAAGAGGGCAGCCAAGAGGGCCCGCGGCAAAAGAAGTCGCGACGGAAGCA
TRINITY_DN2_c0_g2_i1          TCAAGAAGAAGAAGAGGGCAGCCAAGAGGGCCCGCGGCAAAAGAAGTCGCGACGGAAGCA
TRINITY_DN9_c0_g1_i1          TCAAGAAGAAGAAGAGGGCAGCCAAGAGGGCCCGCGGCAAAAGAAGTCGCGACGGAAGCA
TRINITY_DN9_c0_g1_i2          TCAAGAAGAAGAAGAGGGCAGCCAAGAGGGCCCGCGGCAAAAGAAGTCGCGACGGAAGCA
                              ************************************************************

MpDNV_mRNA_seq_consensus      GCGATTCCGAATGATTAGGGAAATAGATGTTAGTAAACCGCAATCTATCGAAGAACTTAT
TRINITY_DN2_c0_g2_i4          GCGATTCCGAATGATTAGGGAAATAGATGTTAGTAAACCGCAATCTATCGAAGAACTTAT
TRINITY_DN2_c0_g2_i2          GCGATTCCGAATGATTAGGGAAATAGATGTTAGTAAACCGCAATCTATCGAAGAACTTAT
TRINITY_DN2_c0_g2_i1          GCGATTCCGAATGATTAGGGAAATAGATGTTAGTAAACCGCAATCTATCGAAGAACTTAT
```

```
TRINITY_DN9_c0_g1_i1         GCGATTCCGAATGATTAGGGAAATAGATGTTAGTAAACCGCAATCTATCGAAGAACTTAT
TRINITY_DN9_c0_g1_i2         GCGATTCCGAATGATTAGGGAAATAGATGTTAGTAAACCGCAATCTATCGAAGAACTTAT
                             ************************************************************

MpDNV_mRNA_seq_consensus     ATATAGGTATCCTTGTTGCCCCCCGGAAGCTTTCTATAACATTCCCGAATTTTACGCTAA
TRINITY_DN2_c0_g2_i4         ATATAGGTATCCTTGTTGCCCCCCGGAAGCTTTCTATAACATTCCCGAATTTTACGCTAA
TRINITY_DN2_c0_g2_i2         ATATAGGTATCCTTGTTGCCCCCCGGAAGCTTTCTATAACATTCCCGAATTTTACGCTAA
TRINITY_DN2_c0_g2_i1         ATATAGGTATCCTTGTTGCCCCCCGGAAGCTTTCTATAACATTCCCGAATTTTACGCTAA
TRINITY_DN9_c0_g1_i1         ATATAGGTATCCTTGTTGCCCCCCGGAAGCTTTCTATAACATTCCCGAATTTTACGCTAA
TRINITY_DN9_c0_g1_i2         ATATAGGTATCCTTGTTGCCCCCCGGAAGCTTTCTATAACATTCCCGAATTTTACGCTAA
                             ************************************************************

MpDNV_mRNA_seq_consensus     TACAAATATAAACTGGATAGACATGAAAGATTTAAAGTAACGATACCGTTACGTAATTG
TRINITY_DN2_c0_g2_i4         TACAAATATAAACTGGATAGACATGAAAGATTTAAAGTAACGATACCGTTACGTAATTG
TRINITY_DN2_c0_g2_i2         TACAAATATAAACTGGATAGACATGAAAGATTTAAAGTAACGATACCGTTACGTAATTG
TRINITY_DN2_c0_g2_i1         TACAAATATAAACTGGATAGACATGAAAGATTTAAAGTAACGATACCGTTACGTAATTG
TRINITY_DN9_c0_g1_i1         TACAAATATAAACTGGATAGACATGAAAGATTTAAAGTAACGATACCGTTACGTAATTG
TRINITY_DN9_c0_g1_i2         TACAAATATAAACTGGATAGACATGAAAGATTTAAAGTAACGATACCGTTACGTAATTG
                             ************************************************************

MpDNV_mRNA_seq_consensus     GGCGGCCACTTTACGCAGATGGTCTATTCATGATTTCAATAAATATTATAATGACTCTAC
TRINITY_DN2_c0_g2_i4         GGCGGCCACTTTACGCAGATGGTCTATTCATGATTTCAATAAATATTATAATGACTCTAC
TRINITY_DN2_c0_g2_i2         GGCGGCCACTTTACGCAGATGGTCTATTCATGATTTCAATAAATATTATAATGACTCTAC
TRINITY_DN2_c0_g2_i1         GGCGGCCACTTTACGCAGATGGTCTATTCATGATTTCAATAAATATTATAATGACTCTAC
TRINITY_DN9_c0_g1_i1         GGCGGCCACTTTACGCAGATGGTCTATTCATGATTTCAATAAATATTATAATGACTCTAC
TRINITY_DN9_c0_g1_i2         GGCGGCCACTTTACGCAGATGGTCTATTCATGATTTCAATAAATATTATAATGACTCTAC
                             ************************************************************

MpDNV_mRNA_seq_consensus     AGTTTTTCCATATTTCAACGCGTACGGTTCAGATATTTGCAATATGTATTACAGTATTTC
TRINITY_DN2_c0_g2_i4         AGTTTTTCCATATTTCAACGCGTACGGTTCAGATATTTGCAATATGTATTACAGTATTTC
TRINITY_DN2_c0_g2_i2         AGTTTTTCCATATTTCAACGCGTACGGTTCAGATATTTGCAATATGTATTACAGTATTTC
TRINITY_DN2_c0_g2_i1         AGTTTTTCCATATTTCAACGCGTACGGTTCAGATATTTGCAATATGTATTACAGTATTTC
TRINITY_DN9_c0_g1_i1         AGTTTTTCCATATTTCAACGCGTACGGTTCAGATATTTGCAATATGTATTACAGTATTTC
TRINITY_DN9_c0_g1_i2         AGTTTTTCCATATTTCAACGCGTACGGTTCAGATATTTGCAATATGTATTACAGTATTTC
                             ************************************************************

MpDNV_mRNA_seq_consensus     GGAGAGTCTGACAATAGCAAAAGAATTACTCAACTATCAATTTGGCGATGACCCTGAAGT
TRINITY_DN2_c0_g2_i4         GGAGAGTCTGACAATAGCAAAAGAATTACTCAACTATCAATTTGGCGATGACCCTGAAGT
TRINITY_DN2_c0_g2_i2         GGAGAGTCTGACAATAGCAAAAGAATTACTCAACTATCAATTTGGCGATGACCCTGAAGT
TRINITY_DN2_c0_g2_i1         GGAGAGTCTGACAATAGCAAAAGAATTACTCAACTATCAATTTGGCGATGACCCTGAAGT
TRINITY_DN9_c0_g1_i1         GGAGAGTCTGACAATAGCAAAAGAATTACTCAACTATCAATTTGGCGATGACCCTGAAGT
TRINITY_DN9_c0_g1_i2         GGAGAGTCTGACAATAGCAAAAGAATTACTCAACTATCAATTTGGCGATGACCCTGAAGT
                             ************************************************************

MpDNV_mRNA_seq_consensus     AGTAATTGAATTTTTAACAACACTATATAATGTCATAGACAAAAGAGTTCCGAAATTAAA
TRINITY_DN2_c0_g2_i4         AGTAATTGAATTTTTAACAACACTATATAATGTCATAGACAAAAGAGTTCCGAAATTAAA
TRINITY_DN2_c0_g2_i2         AGTAATTGAATTTTTAACAACACTATATAATGTCATAGACAAAAGAGTTCCGAAATTAAA
TRINITY_DN2_c0_g2_i1         AGTAATTGAATTTTTAACAACACTATATAATGTCATAGACAAAAGAGTTCCGAAATTAAA
TRINITY_DN9_c0_g1_i1         AGTAATTGAATTTTTAACAACACTATATAATGTCATAGACAAAAGAGTTCCGAAATTAAA
TRINITY_DN9_c0_g1_i2         AGTAATTGAATTTTTAACAACACTATATAATGTCATAGACAAAAGAGTTCCGAAATTAAA
                             ************************************************************

MpDNV_mRNA_seq_consensus     TAGTATATGTATAAAAAGTCCCCCTTCGGCAGGTAAGAACTTTTTTTTCGATGCCGTTGC
TRINITY_DN2_c0_g2_i4         TAGTATATGTATAAAAAGTCCCCCTTCGGCAGGTAAGAACTTTTTTTTCGATGCCGTTGC
TRINITY_DN2_c0_g2_i2         TAGTATATGTATAAAAAGTCCCCCTTCGGCAGGTAAGAACTTTTTTTTCGATGCCGTTGC
TRINITY_DN2_c0_g2_i1         TAGTATATGTATAAAAAGTCCCCCTTCGGCAGGTAAGAACTTTTTTTTCGATGCCGTTGC
TRINITY_DN9_c0_g1_i1         TAGTATATGTATAAAAAGTCCCCCTTCGGCAGGTAAGAACTTTTTTTTCGATGCCGTTGC
TRINITY_DN9_c0_g1_i2         TAGTATATGTATAAAAAGTCCCCCTTCGGCAGGTAAGAACTTTTTTTTCGATGCCGTTGC
                             ************************************************************

MpDNV_mRNA_seq_consensus     ATCGTATTTGCTTTCATATGGTATGTTCGGTACAGCCAATAAAAACAATAATTTTTCGTG
TRINITY_DN2_c0_g2_i4         ATCGTATTTGCTTTCATATGGTATGTTCGGTACAGCCAATAAAAACAATAATTTTTCGTG
TRINITY_DN2_c0_g2_i2         ATCGTATTTGCTTTCATATGGCATGTTCGGTACAGCCAATAAAAACAATAATTTTTCGTG
TRINITY_DN2_c0_g2_i1         ATCGTATTTGCTTTCATATGGCATGTTCGGTACAGCCAATAAAAACAATAATTTTTCGTG
TRINITY_DN9_c0_g1_i1         ATCGTATTTGCTTTCATATGGCATGTTCGGTACAGCCAATAAAAACAATAATTTTTCGTG
TRINITY_DN9_c0_g1_i2         ATCGTATTTGCTTTCATATGGCATGTTCGGTACAGCCAATAAAAACAATAATTTTTCGTG
                             ********************* **************************************

MpDNV_mRNA_seq_consensus     GGCAGACGGAGCGGGTAAACGATTAGTTCTGTGGAACGAACCAAACTATGAACAATACCA
TRINITY_DN2_c0_g2_i4         GGCAGACGGAGCGGGTAAACGATTAGTTCTGTGGAACGAACCAAACTATGAACAATACCA
TRINITY_DN2_c0_g2_i2         GGCAGACGGAGCGGGTAAACGATTAGTTCTGTGGAACGAACCAAACTATGAACAATACCA
TRINITY_DN2_c0_g2_i1         GGCAGACGGAGCGGGTAAACGATTAGTTCTGTGGAACGAACCAAACTATGAACAATACCA
TRINITY_DN9_c0_g1_i1         GGCAGACGGAGCGGGTAAACGATTAGTTCTGTGGAACGAACCAAACTATGAACAATACCA
TRINITY_DN9_c0_g1_i2         GGCAGACGGAGCGGGTAAACGATTAGTTCTGTGGAACGAACCAAACTATGAACAATACCA
                             ************************************************************

MpDNV_mRNA_seq_consensus     TATAGAAAAAAATAAAAGAACTTTTGGGGGGAGATACAACAAGAATACATGTCAAATATGC
TRINITY_DN2_c0_g2_i4         TATAGAAAAAAATAAAAGAACTTTTGGGGGGAGATACAACAAGAATACATGTCAAATATGC
TRINITY_DN2_c0_g2_i2         TATAGAAAAAAATAAAAGAACTTTTGGGGGGAGATACAACAAGAATACATGTCAAATATGC
TRINITY_DN2_c0_g2_i1         TATAGAAAAAAATAAAAGAACTTTTGGGGGGAGATACAACAAGAATACATGTCAAATATGC
TRINITY_DN9_c0_g1_i1         TATAGAAAAAAATAAAAGAACTTTTGGGGGGAGATACAACAAGAATACATGTCAAATATGC
TRINITY_DN9_c0_g1_i2         TATAGAAAAAAATAAAAGAACTTTTGGGGGGAGATACAACAAGAATACATGTCAAATATGC
                             ************************************************************

MpDNV_mRNA_seq_consensus     AAACGACGTTAGTGTGCAAAGGGTACCCATTATTATATTAACAAATAACCACTTGAATAT
TRINITY_DN2_c0_g2_i4         AAACGACGTTAGTGTGCAAAGGGTACCCATTATTATATTAACAAATAACCACTTGAATAT
TRINITY_DN2_c0_g2_i2         AAACGACGTTAGTGTGCAAAGGGTACCCATTATTATATTAACAAATAACCACTTGAATAT
TRINITY_DN2_c0_g2_i1         AAACGACGTTAGTGTGCAAAGGGTACCCATTATTATATTAACAAATAACCACTTGAATAT
```

```
TRINITY_DN9_c0_g1_i1          AAACGACGTTAGTGTGCAAAGGGTACCCATTATTATATTAACAAATAACCACTTGAATAT
TRINITY_DN9_c0_g1_i2          AAACGACGTTAGTGTGCAAAGGGTACCCATTATTATATTAACAAATAACCACTTGAATAT
                              ************************************************************

MpDNV_mRNA_seq_consensus      TATTAGTCACCCAGCTTTTAACGATAGATTGCGGAGTTACGAATGGATGTCAGCTGGCTT
TRINITY_DN2_c0_g2_i4          TATTAGTCACCCAGCTTTTAACGATAGATTGCGGAGTTACGAATGGATGTCAGCTGGCTT
TRINITY_DN2_c0_g2_i2          TATTAGTCACCCAGCTTTTAACGATAGATTGCGGAGTTACGAATGGATGTCAGCTGGCTT
TRINITY_DN2_c0_g2_i1          TATTAGTCACCCAGCTTTTAACGATAGATTGCGGAGTTACGAATGGATGTCAGCTGGCTT
TRINITY_DN9_c0_g1_i1          TATTAGTCACCCAGCTTTTAACGATAGATTGCGGAGTTACGAATGGATGTCAGCTGGCTT
TRINITY_DN9_c0_g1_i2          TATTAGTCACCCAGCTTTTAACGATAGATTGCGGAGTTACGAATGGATGTCAGCTGGCTT
                              ************************************************************

MpDNV_mRNA_seq_consensus      CTTGAAGGACTACGATAAAAAACTGCATCCGCTTATGTTTTATGAATTACTTAAAGATTA
TRINITY_DN2_c0_g2_i4          CTTGAAGGACTACGATAAAAAACTGCATCCGCTTATGTTTTATGAATTACTTAAAGATTA
TRINITY_DN2_c0_g2_i2          CTTGAAGGACTACGATAAAAAACTGCATCCGCTTATGTTTTATGAATTACTTAAAGATTA
TRINITY_DN2_c0_g2_i1          CTTGAAGGACTACGATAAAAAACTGCATCCGCTTATGTTTTATGAATTACTTAAAGATTA
TRINITY_DN9_c0_g1_i1          CTTGAAGGACTACGATAAAAAACTGCATCCGCTTATGTTTTATGAATTACTTAAAGATTA
TRINITY_DN9_c0_g1_i2          CTTGAAGGACTACGATAAAAAACTGCATCCGCTTATGTTTTATGAATTACTTAAAGATTA
                              ************************************************************

MpDNV_mRNA_seq_consensus      TGGTGTAATAAATGATAACAATATGATTGATTAATATATATATATATATATTGATTTTTA NS polyA signal
TRINITY_DN2_c0_g2_i4  APFV-7  TGGTGTAATAAATGATAACAATATGATTGATTA--ATATATATATATATATTGATTTTTA
TRINITY_DN2_c0_g2_i2  APFV-5  TGGTGTAATAAATGATAACAATATGATTGATTA--ATATATATATATATATTGATTTTTA
TRINITY_DN2_c0_g2_i1          TGGTGTAATAAATGATAACAATATGATTGATTA--ATATATATATATATATTGATTTTTA
TRINITY_DN9_c0_g1_i1  APFV-6  TGGTGTAATAAATGATAACAATATGATTGATTAATATATATATATATATATTGATTTTTA
TRINITY_DN9_c0_g1_i2          TGGTGTAATAAATGATAACAATATGATTGATTAATATATATATATATATATTGATTTTTA
                              *********************************  *************************

MpDNV_mRNA_seq_consensus      TTTACGTAAAAAATCTACAGGTTCATTGACAATGGGTATATTCATTGACCTTCGCCACAA
TRINITY_DN2_c0_g2_i4  APFV-7  AAAAAAA----------------------------------------------------- NS poly(A) tail
TRINITY_DN2_c0_g2_i2  APFV-5  TTTACGTAAAAAAAAA-------------------------------------------- NS poly(A) tail
TRINITY_DN2_c0_g2_i1  APFV-5  TTTACGTAAAAAAAAA-------------------------------------------- NS poly(A) tail
TRINITY_DN9_c0_g1_i1  APFV-6  TTTACGTAAAAAAAAAAAAA---------------------------------------- NS poly(A) tail
TRINITY_DN9_c0_g1_i2  APFV-6  TTTACGTAAAAAAAAAAAAA---------------------------------------- NS poly(A) tail
                                     ******

MpDNV_mRNA_seq_consensus      TGGTGTAATAAATGATAACAATATGATTGATTAATATATATATATATATATTGATTTTTA VP polyA signal
TRINITY_DN2_c0_g1_i2  APFV-5  ----------------TTTTTTTTTTTTGATTAATATATATATATATATATTGATTTTTA VP poly(A) tail
TRINITY_DN2_c0_g1_i3  APFV-5  ----------------TTTTTTTTTTTTGATTAATATATATATATATATATTGATTTTTA VP poly(A) tail
TRINITY_DN2_c0_g1_i4  APFV-5  ----------------TTTTTTTTTTTTGATTAATATATATATATATATATTGATTTTTA VP poly(A) tail
TRINITY_DN2_c0_g1_i1  APFV-5  ----------------TTTTTTTTTTTTGATTAATATATATATATATATATTGATTTTTA VP poly(A) tail
TRINITY_DN39_c0_g1_i4 APFV-6  ----------------TTTTTTTTTTTTGATTAATATATATATATATATATTGATTTTTA VP poly(A) tail
TRINITY_DN39_c0_g1_i3 APFV-6  ----------------TTTTTTTTTTTTGATTAATATATATATATATATATTGATTTTTA VP poly(A) tail
TRINITY_DN39_c0_g1_i1 APFV-6  ----------------TTTTTTTTTTTTGATTAATATATATATATATATATTGATTTTTA VP poly(A) tail
TRINITY_DN39_c0_g1_i2 APFV-6  ----------------TTTTTTTTTTTTGATTAATATATATATATATATATTGATTTTTA VP poly(A) tail
TRINITY_DN2_c0_g2_i3  APFV-7  ----------------TTTTTTTTTTTTGATTA--ATATATATATATATATTGATTTTTA VP poly(A) tail
TRINITY_DN2_c0_g2_i2  APFV-7  ----------------TTTTTTTTTTTTGATTA--ATATATATATATATATTGATTTTTA VP poly(A) tail
TRINITY_DN2_c0_g2_i1  APFV-7  ----------------TTTTTTTTTTTTGATTA--ATATATATATATATATTGATTTTTA VP poly(A) tail
TRINITY_DN2_c0_g2_i5  APFV-7  ----------------TTTTTTTTTTTTGATTA--ATATATATATATATATTGATTTTTA VP poly(A) tail
                                                        *******  *************************

MpDNV_mRNA_seq_consensus      TTTACGTAAAAAATCTACAGGTTCATTGACAATGGGTATATTCATTGACCTTCGCCACAA VP polyA signal
TRINITY_DN2_c0_g1_i2          TTTACGTAAAAAATCTACAGGTTCATTGACAATGGGTATATTCATTGACCTTCGCCACAA
TRINITY_DN2_c0_g1_i3          TTTACGTAAAAAATCTACAGGTTCATTGACAATGGGTATATTCATTGACCTTCGCCACAA
TRINITY_DN2_c0_g1_i4          TTTACGTAAAAAATCTACAGGTTCATTGACAATGGGTATATTCATTGACCTTCGCCACAA
TRINITY_DN2_c0_g1_i1          TTTACGTAAAAAATCTACAGGTTCATTGACAATGGGTATATTCATTGACCTTCGCCACAA
TRINITY_DN39_c0_g1_i4         TTTACGTAAAAAATCTACAGGTTCATTGACAATGGGTATATTCATTGACCTTCGCCACAA
TRINITY_DN39_c0_g1_i3         TTTACGTAAAAAATCTACAGGTTCATTGACAATGGGTATATTCATTGACCTTCGCCACAA
TRINITY_DN39_c0_g1_i1         TTTACGTAAAAAATCTACAGGTTCATTGACAATGGGTATATTCATTGACCTTCGCCACAA
TRINITY_DN39_c0_g1_i2         TTTACGTAAAAAATCTACAGGTTCATTGACAATGGGTATATTCATTGACCTTCGCCACAA
TRINITY_DN2_c0_g2_i3          TTTACGTAAAAAATCTACAGGTTCATTGACAATGGGTATATTCATTGACCTTCGCCACAA
TRINITY_DN2_c0_g2_i2          TTTACGTAAAAAATCTACAGGTTCATTGACAATGGGTATATTCATTGACCTTCGCCACAA
TRINITY_DN2_c0_g2_i1          TTTACGTAAAAAATCTACAGGTTCATTGACAATGGGTATATTCATTGACCTTCGCCACAA
TRINITY_DN2_c0_g2_i5          TTTACGTAAAAAATCTACAGGTTCATTGACAATGGGTATATTCATTGACCTTCGCCACAA
                              ************************************************************

MpDNV_mRNA_seq_consensus      TGTTAAATCACGGCAAAAAGCTAAATACGTTGAATATGACTTGAACAATCGTTCCTCTGG
TRINITY_DN2_c0_g1_i2          TGTTAAATCACGGCAAAAAGCTAAATACGTTGAATATGACTTGAACAATCGTTCCTCTGG
TRINITY_DN2_c0_g1_i3          TGTTAAATCACGGCAAAAAGCTAAATACGTTGAATATGACTTGAACAATCGTTCCTCTGG
TRINITY_DN2_c0_g1_i4          TGTTAAATCACGGCAAAAAGCTAAATACGTTGAATATGACTTGAACAATCGTTCCTCTGG
TRINITY_DN2_c0_g1_i1          TGTTAAATCACGGCAAAAAGCTAAATACGTTGAATATGACTTGAACAATCGTTCCTCTGG
TRINITY_DN39_c0_g1_i4         TGTTAAATCACGGCAAAAAGCTAAATACGTTGAATATGACTTGAACAATCGTTCCTCTGG
TRINITY_DN39_c0_g1_i3         TGTTAAATCACGGCAAAAAGCTAAATACGTTGAATATGACTTGAACAATCGTTCCTCTGG
TRINITY_DN39_c0_g1_i1         TGTTAAATCACGGCAAAAAGCTAAATACGTTGAATATGACTTGAACAATCGTTCCTCTGG
TRINITY_DN39_c0_g1_i2         TGTTAAATCACGGCAAAAAGCTAAATACGTTGAATATGACTTGAACAATCGTTCCTCTGG
TRINITY_DN2_c0_g2_i3          TGTTAAATCACGGCAAAAAGCTAAATACGTTGAATATGACTTGAACAATCGTTCCTCTGG
TRINITY_DN2_c0_g2_i2          TGTTAAATCACGGCAAAAAGCTAAATACGTTGAATATGACTTGAACAATCGTTCCTCTGG
TRINITY_DN2_c0_g2_i1          TGTTAAATCACGGCAAAAAGCTAAATACGTTGAATATGACTTGAACAATCGTTCCTCTGG
TRINITY_DN2_c0_g2_i5          TGTTAAATCACGGCAAAAAGCTAAATACGTTGAATATGACTTGAACAATCGTTCCTCTGG
                              ************************************************************

MpDNV_mRNA_seq_consensus      CCACCAATTCGGATTATTGTAGTCGCCCCAAGAAGTGTTCATGATTTCTTCGGTTGTGGG
TRINITY_DN2_c0_g1_i2          CCACCAATTCGGATTATTGTAGTCGCCCCAAGAAGTGTTCATGATTTCTTCGGTTGTGGG
TRINITY_DN2_c0_g1_i3          CCACCAATTCGGATTATTGTAGTCGCCCCAAGAAGTGTTCATGATTTCTTCGGTTGTGGG
TRINITY_DN2_c0_g1_i4          CCACCAATTCGGATTATTGTAGTCGCCCCAAGAAGTGTTCATGATTTCTTCGGTTGTGGG
TRINITY_DN2_c0_g1_i1          CCACCAATTCGGATTATTGTAGTCGCCCCAAGAAGTGTTCATGATTTCTTCGGTTGTGGG
TRINITY_DN39_c0_g1_i4         CCACCAATTCGGATTATTGTAGTCGCCCCAAGAAGTGTTCATGATTTCTTCGGTTGTGGG
```

```
TRINITY_DN39_c0_g1_i3         CCACCAATTCGGATTATTGTAGTCGCCCCAAGAAGTGTTCATGATTTCTTCGGTTGTGGG
TRINITY_DN39_c0_g1_i1         CCACCAATTCGGATTATTGTAGTCGCCCCAAGAAGTGTTCATGATTTCTTCGGTTGTGGG
TRINITY_DN39_c0_g1_i2         CCACCAATTCGGATTATTGTAGTCGCCCCAAGAAGTGTTCATGATTTCTTCGGTTGTGGG
TRINITY_DN2_c0_g2_i3          CCACCAATTCGGATTATTGTAGTCGCCCCAAGAAGTGTTCATGATTTCTTCGGTTGTGGG
TRINITY_DN2_c0_g2_i2          CCACCAATTCGGATTATTGTAGTCGCCCCAAGAAGTGTTCATGATTTCTTCGGTTGTGGG
TRINITY_DN2_c0_g2_i1          CCACCAATTCGGATTATTGTAGTCGCCCCAAGAAGTGTTCATGATTTCTTCGGTTGTGGG
TRINITY_DN2_c0_g2_i5          CCACCAATTCGGATTATTGTAGTCGCCCCAAGAAGTGTTCATGATTTCTTCGGTTGTGGG
                              ************************************************************

MpDNV_mRNA_seq_consensus      TGGAGTAGTGGTATATCGATGCAAATTAATTAGGGTTTCTTTTGACGCACGATCACTTTC
TRINITY_DN2_c0_g1_i2          TGGAGTAGTGGTATATCGATGCAAATTAATTAGGGTTTCTTTTGACGCACGATCACTTTC
TRINITY_DN2_c0_g1_i3          TGGAGTAGTGGTATATCGATGCAAATTAATTAGGGTTTCTTTTGACGCACGATCACTTTC
TRINITY_DN2_c0_g1_i4          TGGAGTAGTGGTATATCGATGCAAATTAATTAGGGTTTCTTTTGACGCACGATCACTTTC
TRINITY_DN2_c0_g1_i1          TGGAGTAGTGGTATATCGATGCAAATTAATTAGGGTTTCTTTTGACGCACGATCACTTTC
TRINITY_DN39_c0_g1_i4         TGGAGTAGTGGTATATCGATGCAAATTAATTAGGGTTTCTTTTGACGCACGATCACTTTC
TRINITY_DN39_c0_g1_i3         TGGAGTAGTGGTATATCGATGCAAATTAATTAGGGTTTCTTTTGACGCACGATCACTTTC
TRINITY_DN39_c0_g1_i1         TGGAGTAGTGGTATATCGATGCAAATTAATTAGGGTTTCTTTTGACGCACGATCACTTTC
TRINITY_DN39_c0_g1_i2         TGGAGTAGTGGTATATCGATGCAAATTAATTAGGGTTTCTTTTGACGCACGATCACTTTC
TRINITY_DN2_c0_g2_i3          TGGAGTAGTGGTATATCGATGCAAATTAATTAGGGTTTCTTTTGACGCACGATCACTTTC
TRINITY_DN2_c0_g2_i2          TGGAGTAGTGGTATATCGATGCAAATTAATTAGGGTTTCTTTTGACGCACGATCACTTTC
TRINITY_DN2_c0_g2_i1          TGGAGTAGTGGTATATCGATGCAAATTAATTAGGGTTTCTTTTGACGCACGATCACTTTC
TRINITY_DN2_c0_g2_i5          TGGAGTAGTGGTATATCGATGCAAATTAATTAGGGTTTCTTTTGACGCACGATCACTTTC
                              ************************************************************

MpDNV_mRNA_seq_consensus      TCTGTAAACATAATTGGGTATTTTAATAGGTGCTTTGACTCGAACCTCATGGATGTTTGA 3' splice site
TRINITY_DN2_c0_g1_i2          TCTGTAAACATAATTGGGTATTTTAATAGGTGCTTTGACTCGAACCTCATGGATGTTTGA unspliced
TRINITY_DN2_c0_g1_i3          TCTGTAAACATAATTGGGTATTTTAATAGGTGCTTTGACTCGAACCTCATGGATGTTTGA unspliced
TRINITY_DN2_c0_g1_i4          T----------------------------------------------------------- spliced intron 2
TRINITY_DN2_c0_g1_i1          T----------------------------------------------------------- spliced intron 2
TRINITY_DN39_c0_g1_i4         T----------------------------------------------------------- spliced intron 2
TRINITY_DN39_c0_g1_i3         TCTGTAAACATAATTGGGTATTTTAATAGGTGCTTTGACTCGAACCTCATGGATGTTTGA unspliced
TRINITY_DN39_c0_g1_i1         T----------------------------------------------------------- spliced intron 2
TRINITY_DN39_c0_g1_i2         TCTGTAAACATAATTGGGTATTTTAATAGGTGCTTTGACTCGAACCTCATGGATGTTTGA unspliced
TRINITY_DN2_c0_g2_i3          TCTGTAAACATAATTGGGTATTTTAATAGGTGCTTTGACTCGAACCTCATGGATGTTTGA unspliced
TRINITY_DN2_c0_g2_i2          TCTGTAAACATAATTGGGTATTTTAATAGGTGCTTTGACTCGAACCTCATGGATGTTTGA unspliced
TRINITY_DN2_c0_g2_i1          T----------------------------------------------------------- spliced intron 2
TRINITY_DN2_c0_g2_i5          T----------------------------------------------------------- spliced intron 2
                              *

MpDNV_mRNA_seq_consensus      CGCTTCAACCTCCATGGGGTCGTCGTCCATATTTACCCGAACAGACGTAGGTACAGGTGT 5' splice site
TRINITY_DN2_c0_g1_i2          CGCTTCAACCTCCATGGGGTCGTCGTCCATATTTACCCGAACAGACGTAGGTACAGGTGT unspliced
TRINITY_DN2_c0_g1_i3          CGCTTCAACCTCCATGGGGTCGTCGTCCATATTTACCCGAACAGACGTAGGTACAGGTGT unspliced
TRINITY_DN2_c0_g1_i4          ------------------------------------CCGAACAGACGTAGGTACAGGTGT spliced intron 2
TRINITY_DN2_c0_g1_i1          ------------------------------------CCGAACAGACGTAGGTACAGGTGT spliced intron 2
TRINITY_DN39_c0_g1_i4         ------------------------------------CCGAACAGACGTAGGTACAGGTGT spliced intron 2
TRINITY_DN39_c0_g1_i3         CGCTTCAACCTCCATGGGGTCGTCGTCCATATTTACCCGAACAGACGTAGGTACAGGTGT unspliced
TRINITY_DN39_c0_g1_i1         ------------------------------------CCGAACAGACGTAGGTACAGGTGT spliced intron 2
TRINITY_DN39_c0_g1_i2         CGCTTCAACCTCCATGGGGTCGTCGTCCATATTTACCCGAACAGACGTAGGTACAGGTGT unspliced
TRINITY_DN2_c0_g2_i3          CGCTTCAACCTCCATGGGGTCGTCGTCCATATTTACCCGAACAGACGTAGGTACAGGTGT unspliced
TRINITY_DN2_c0_g2_i2          CGCTTCAACCTCCATGGGGTCGTCGTCCATATTTACCCGAACAGACGTAGGTACAGGTGT unspliced
TRINITY_DN2_c0_g2_i1          ------------------------------------CCGAACAGACGTAGGTACAGGTGT spliced intron 2
TRINITY_DN2_c0_g2_i5          ------------------------------------CCGAACAGACGTAGGTACAGGTGT spliced intron 2
                                                                  ************************

MpDNV_mRNA_seq_consensus      AGTGACAGTTGCGTCGACTGGAGTAGTAAATGAATCAATGTTCTTTGTAGTGGAAGCTTT
TRINITY_DN2_c0_g1_i2          AGTGACAGTTGCGTCGACTGGAGTAGTAAATGAATCAATGTTCTTTGTAGTGGAAGCTTT
TRINITY_DN2_c0_g1_i3          AGTGACAGTTGCGTCGACTGGAGTAGTAAATGAATCAATGTTCTTTGTAGTGGAAGCTTT
TRINITY_DN2_c0_g1_i4          AGTGACAGTTGCGTCGACTGGAGTAGTAAATGAATCAATGTTCTTTGTAGTGGAAGCTTT
TRINITY_DN2_c0_g1_i1          AGTGACAGTTGCGTCGACTGGAGTAGTAAATGAATCAATGTTCTTTGTAGTGGAAGCTTT
TRINITY_DN39_c0_g1_i4         AGTGACAGTTGCGTCGACTGGAGTAGTAAATGAATCAATGTTCTTTGTAGTGGAAGCTTT
TRINITY_DN39_c0_g1_i3         AGTGACAGTTGCGTCGACTGGAGTAGTAAATGAATCAATGTTCTTTGTAGTGGAAGCTTT
TRINITY_DN39_c0_g1_i1         AGTGACAGTTGCGTCGACTGGAGTAGTAAATGAATCAATGTTCTTTGTAGTGGAAGCTTT
TRINITY_DN39_c0_g1_i2         AGTGACAGTTGCGTCGACTGGAGTAGTAAATGAATCAATGTTCTTTGTAGTGGAAGCTTT
TRINITY_DN2_c0_g2_i3          AGTGACAGTTGCGTCGACTGGAGTAGTAAATGAATCAATGTTCTTTGTAGTGGAAGCTTT
TRINITY_DN2_c0_g2_i2          AGTGACAGTTGCGTCGACTGGAGTAGTAAATGAATCAATGTTCTTTGTAGTGGAAGCTTT
TRINITY_DN2_c0_g2_i1          AGTGACAGTTGCGTCGACTGGAGTAGTAAATGAATCAATGTTCTTTGTAGTGGAAGCTTT
TRINITY_DN2_c0_g2_i5          AGTGACAGTTGCGTCGACTGGAGTAGTAAATGAATCAATGTTCTTTGTAGTGGAAGCTTT
                              ************************************************************

MpDNV_mRNA_seq_consensus      GCCCATTTCTTTAGTTAGTCCCGAAGAGTGTGCAGCGGCGTTTTGATTGTCAGCTACTTT
TRINITY_DN2_c0_g1_i2          GCCCATTTCTTTAGTTAGTCCCGAAGAGTGTGCAGCGGCGTTTTGATTGTCAGCTACTTT
TRINITY_DN2_c0_g1_i3          GCCCATTTCTTTAGTTAGTCCCGAAGAGTGTGCAGCGGCGTTTTGATTGTCAGCTACTTT
TRINITY_DN2_c0_g1_i4          GCCCATTTCTTTAGTTAGTCCCGAAGAGTGTGCAGCGGCGTTTTGATTGTCAGCTACTTT
TRINITY_DN2_c0_g1_i1          GCCCATTTCTTTAGTTAGTCCCGAAGAGTGTGCAGCGGCGTTTTGATTGTCAGCTACTTT
TRINITY_DN39_c0_g1_i4         GCCCATTTCTTTAGTTAGTCCCGAAGAGTGTGCAGCGGCGTTTTGATTGTCAGCTACTTT
TRINITY_DN39_c0_g1_i3         GCCCATTTCTTTAGTTAGTCCCGAAGAGTGTGCAGCGGCGTTTTGATTGTCAGCTACTTT
TRINITY_DN39_c0_g1_i1         GCCCATTTCTTTAGTTAGTCCCGAAGAGTGTGCAGCGGCGTTTTGATTGTCAGCTACTTT
TRINITY_DN39_c0_g1_i2         GCCCATTTCTTTAGTTAGTCCCGAAGAGTGTGCAGCGGCGTTTTGATTGTCAGCTACTTT
TRINITY_DN2_c0_g2_i3          GCCCATTTCTTTAGTTAGTCCCGAAGAGTGTGCAGCGGCGTTTTGATTGTCAGCTACTTT
TRINITY_DN2_c0_g2_i2          GCCCATTTCTTTAGTTAGTCCCGAAGAGTGTGCAGCGGCGTTTTGATTGTCAGCTACTTT
TRINITY_DN2_c0_g2_i1          GCCCATTTCTTTAGTTAGTCCCGAAGAGTGTGCAGCGGCGTTTTGATTGTCAGCTACTTT
TRINITY_DN2_c0_g2_i5          GCCCATTTCTTTAGTTAGTCCCGAAGAGTGTGCAGCGGCGTTTTGATTGTCAGCTACTTT
                              ************************************************************

MpDNV_mRNA_seq_consensus      TTCAGGAGCCACGTACAATCCGTGAACAATAGATAAATCTCCACGATTGTATAGATTTTG
TRINITY_DN2_c0_g1_i2          TTCAGGAGCCACGTACAATCCGTGAACAATAGATAAATCTCCACGATTGTATAGATTTTG
TRINITY_DN2_c0_g1_i3          TTCAGGAGCCACGTACAATCCGTGAACAATAGATAAATCTCCACGATTGTATAGATTTTG
TRINITY_DN2_c0_g1_i4          TTCAGGAGCCACGTACAATCCGTGAACAATAGATAAATCTCCACGATTGTATAGATTTTG
```

```
TRINITY_DN2_c0_g1_i1         TTCAGGAGCCACGTACAATCCGTGAACAATAGATAAATCTCCACGATTGTATAGATTTTG
TRINITY_DN39_c0_g1_i4        TTCAGGAGCCACGTACAATCCGTGAACAATAGATAAATCTCCACGATTGTATAGATTTTG
TRINITY_DN39_c0_g1_i3        TTCAGGAGCCACGTACAATCCGTGAACAATAGATAAATCTCCACGATTGTATAGATTTTG
TRINITY_DN39_c0_g1_i1        TTCAGGAGCCACGTACAATCCGTGAACAATAGATAAATCTCCACGATTGTATAGATTTTG
TRINITY_DN39_c0_g1_i2        TTCAGGAGCCACGTACAATCCGTGAACAATAGATAAATCTCCACGATTGTATAGATTTTG
TRINITY_DN2_c0_g2_i3         TTCAGGAGCCACGTACAATCCGTGAACAATAGATAAATCTCCACGATTGTATAGATTTTG
TRINITY_DN2_c0_g2_i2         TTCAGGAGCCACGTACAATCCGTGAACAATAGATAAATCTCCACGATTGTATAGATTTTG
TRINITY_DN2_c0_g2_i1         TTCAGGAGCCACGTACAATCCGTGAACAATAGATAAATCTCCACGATTGTATAGATTTTG
TRINITY_DN2_c0_g2_i5         TTCAGGAGCCACGTACAATCCGTGAACAATAGATAAATCTCCACGATTGTATAGATTTTG
                             ************************************************************

MpDNV_mRNA_seq_consensus     ATTGTAATTGTCGTCGGCCAGCAACCATGTCTTTTCGTATTCCACGTGAAGCCTGTCAAA
TRINITY_DN2_c0_g1_i2         ATTGTAATTGTCGTCGGCCAGCAACCATGTCTTTTCGTATTCCACGTGAAGCCTGTCAAA
TRINITY_DN2_c0_g1_i3         ATTGTAATTGTCGTCGGCCAGCAACCATGTCTTTTCGTATTCCACGTGAAGCCTGTCAAA
TRINITY_DN2_c0_g1_i4         ATTGTAATTGTCGTCGGCCAGCAACCATGTCTTTTCGTATTCCACGTGAAGCCTGTCAAA
TRINITY_DN2_c0_g1_i1         ATTGTAATTGTCGTCGGCCAGCAACCATGTCTTTTCGTATTCCACGTGAAGCCTGTCAAA
TRINITY_DN39_c0_g1_i4        ATTGTAATTGTCGTCGGCCAGCAACCATGTCTTTTCGTATTCCACGTGAAGCCTGTCAAA
TRINITY_DN39_c0_g1_i3        ATTGTAATTGTCGTCGGCCAGCAACCATGTCTTTTCGTATTCCACGTGAAGCCTGTCAAA
TRINITY_DN39_c0_g1_i1        ATTGTAATTGTCGTCGGCCAGCAACCATGTCTTTTCGTATTCCACGTGAAGCCTGTCAAA
TRINITY_DN39_c0_g1_i2        ATTGTAATTGTCGTCGGCCAGCAACCATGTCTTTTCGTATTCCACGTGAAGCCTGTCAAA
TRINITY_DN2_c0_g2_i3         ATTGTAATTGTCGTCGGCCAGCAACCATGTCTTTTCGTATTCCACGTGAAGCCTGTCAAA
TRINITY_DN2_c0_g2_i2         ATTGTAATTGTCGTCGGCCAGCAACCATGTCTTTTCGTATTCCACGTGAAGCCTGTCAAA
TRINITY_DN2_c0_g2_i1         ATTGTAATTGTCGTCGGCCAGCAACCATGTCTTTTCGTATTCCACGTGAAGCCTGTCAAA
TRINITY_DN2_c0_g2_i5         ATTGTAATTGTCGTCGGCCAGCAACCATGTCTTTTCGTATTCCACGTGAAGCCTGTCAAA
                             ************************************************************

MpDNV_mRNA_seq_consensus     CGCTGTTCTGCTTTGCGGACATCCAAAACTAACTGTCATCTCAGTTTCGAAATCCCATGT
TRINITY_DN2_c0_g1_i2         CGCTGTTCTGCTTTGCGGACATCCAAAACTAACTGTCATCTCAGTTTCGAAATCCCATGT
TRINITY_DN2_c0_g1_i3         CGCTGTTCTGCTTTGCGGACATCCAAAACTAACTGTCATCTCAGTTTCGAAATCCCATGT
TRINITY_DN2_c0_g1_i4         CGCTGTTCTGCTTTGCGGACATCCAAAACTAACTGTCATCTCAGTTTCGAAATCCCATGT
TRINITY_DN2_c0_g1_i1         CGCTGTTCTGCTTTGCGGACATCCAAAACTAACTGTCATCTCAGTTTCGAAATCCCATGT
TRINITY_DN39_c0_g1_i4        CGCTGTTCTGCTTTGCGGACATCCAAAACTAACTGTCATCTCAGTTTCGAAATCCCATGT
TRINITY_DN39_c0_g1_i3        CGCTGTTCTGCTTTGCGGACATCCAAAACTAACTGTCATCTCAGTTTCGAAATCCCATGT
TRINITY_DN39_c0_g1_i1        CGCTGTTCTGCTTTGCGGACATCCAAAACTAACTGTCATCTCAGTTTCGAAATCCCATGT
TRINITY_DN39_c0_g1_i2        CGCTGTTCTGCTTTGCGGACATCCAAAACTAACTGTCATCTCAGTTTCGAAATCCCATGT
TRINITY_DN2_c0_g2_i3         CGCTGTTCTGCTTTGCGGACATCCAAAACTAACTGTCATCTCAGTTTCGAAATCCCATGT
TRINITY_DN2_c0_g2_i2         CGCTGTTCTGCTTTGCGGACATCCAAAACTAACTGTCATCTCAGTTTCGAAATCCCATGT
TRINITY_DN2_c0_g2_i1         CGCTGTTCTGCTTTGCGGACATCCAAAACTAACTGTCATCTCAGTTTCGAAATCCCATGT
TRINITY_DN2_c0_g2_i5         CGCTGTTCTGCTTTGCGGACATCCAAAACTAACTGTCATCTCAGTTTCGAAATCCCATGT
                             ************************************************************

MpDNV_mRNA_seq_consensus     GCATTCTACGTCAGTAAAATTTACAGGGACTATACTATTAGTGGCAGTAGTTAACTTATG
TRINITY_DN2_c0_g1_i2         GCATTCTACGTCAGTAAAATTTACAGGGACTATACTATTAGTGGCAGTAGTTAACTTATG
TRINITY_DN2_c0_g1_i3         GCATTCTACGTCAGTAAAATTTACAGGGACTATACTATTAGTGGCAGTAGTTAACTTATG
TRINITY_DN2_c0_g1_i4         GCATTCTACGTCAGTAAAATTTACAGGGACTATACTATTAGTGGCAGTAGTTAACTTATG
TRINITY_DN2_c0_g1_i1         GCATTCTACGTCAGTAAAATTTACAGGGACTATACTATTAGTGGCAGTAGTTAACTTATG
TRINITY_DN39_c0_g1_i4        GCATTCTACGTCAGTAAAATTTACAGGGACTATACTATTAGTGGCAGTAGTTAACTTATG
TRINITY_DN39_c0_g1_i3        GCATTCTACGTCAGTAAAATTTACAGGGACTATACTATTAGTGGCAGTAGTTAACTTATG
TRINITY_DN39_c0_g1_i1        GCATTCTACGTCAGTAAAATTTACAGGGACTATACTATTAGTGGCAGTAGTTAACTTATG
TRINITY_DN39_c0_g1_i2        GCATTCTACGTCAGTAAAATTTACAGGGACTATACTATTAGTGGCAGTAGTTAACTTATG
TRINITY_DN2_c0_g2_i3         GCATTCTACGTCAGTAAAATTTACAGGGACTATACTATTAGTGGCAGTAGTTAACTTATG
TRINITY_DN2_c0_g2_i2         GCATTCTACGTCAGTAAAATTTACAGGGACTATACTATTAGTGGCAGTAGTTAACTTATG
TRINITY_DN2_c0_g2_i1         GCATTCTACGTCAGTAAAATTTACAGGGACTATACTATTAGTGGCAGTAGTTAACTTATG
TRINITY_DN2_c0_g2_i5         GCATTCTACGTCAGTAAAATTTACAGGGACTATACTATTAGTGGCAGTAGTTAACTTATG
                             ************************************************************

MpDNV_mRNA_seq_consensus     CACTGGATATATTCCTACATGTACACTAGGTTGTACTTTATTAGAATATTCATGTTTATC
TRINITY_DN2_c0_g1_i2         CACTGGATATATTCCTACATGTACACTAGGTTGTACTTTATTAGAATATTCATGTTTATC
TRINITY_DN2_c0_g1_i3         CACTGGATATATTCCTACATGTACACTAGGTTGTACTTTATTAGAATATTCATGTTTATC
TRINITY_DN2_c0_g1_i4         CACTGGATATATTCCTACATGTACACTAGGTTGTACTTTATTAGAATATTCATGTTTATC
TRINITY_DN2_c0_g1_i1         CACTGGATATATTCCTACATGTACACTAGGTTGTACTTTATTAGAATATTCATGTTTATC
TRINITY_DN39_c0_g1_i4        CACTGGATATATTCCTACATGTACACTAGGTTGTACTTTATTAGAATATTCATGTTTATC
TRINITY_DN39_c0_g1_i3        CACTGGATATATTCCTACATGTACACTAGGTTGTACTTTATTAGAATATTCATGTTTATC
TRINITY_DN39_c0_g1_i1        CACTGGATATATTCCTACATGTACACTAGGTTGTACTTTATTAGAATATTCATGTTTATC
TRINITY_DN39_c0_g1_i2        CACTGGATATATTCCTACATGTACACTAGGTTGTACTTTATTAGAATATTCATGTTTATC
TRINITY_DN2_c0_g2_i3         CACTGGATATATTCCTACATGTACACTAGGTTGTACTTTATTAGAATATTCATGTTTATC
TRINITY_DN2_c0_g2_i2         CACTGGATATATTCCTACATGTACACTAGGTTGTACTTTATTAGAATATTCATGTTTATC
TRINITY_DN2_c0_g2_i1         CACTGGATATATTCCTACATGTACACTAGGTTGTACTTTATTAGAATATTCATGTTTATC
TRINITY_DN2_c0_g2_i5         CACTGGATATATTCCTACATGTACACTAGGTTGTACTTTATTAGAATATTCATGTTTATC
                             ************************************************************

MpDNV_mRNA_seq_consensus     GAACATACGAAAATATTGGCCTACGTCTATGTTAGTACAATATCTATCATTGCCGAATAT
TRINITY_DN2_c0_g1_i2         GAACATACGAAAATATTGGCCTACGTCTATGTTAGTACAATATCTATCATTGCCGAATAT
TRINITY_DN2_c0_g1_i3         GAACATACGAAAATATTGGCCTACGTCTATGTTAGTACAATATCTATCATTGCCGAATAT
TRINITY_DN2_c0_g1_i4         GAACATACGAAAATATTGGCCTACGTCTATGTTAGTACAATATCTATCATTGCCGAATAT
TRINITY_DN2_c0_g1_i1         GAACATACGAAAATATTGGCCTACGTCTATGTTAGTACAATATCTATCATTGCCGAATAT
TRINITY_DN39_c0_g1_i4        GAACATACGAAAATATTGGCCTACGTCTATGTTAGTACAATATCTATCATTGCCGAATAT
TRINITY_DN39_c0_g1_i3        GAACATACGAAAATATTGGCCTACGTCTATGTTAGTACAATATCTATCATTGCCGAATAT
TRINITY_DN39_c0_g1_i1        GAACATACGAAAATATTGGCCTACGTCTATGTTAGTACAATATCTATCATTGCCGAATAT
TRINITY_DN39_c0_g1_i2        GAACATACGAAAATATTGGCCTACGTCTATGTTAGTACAATATCTATCATTGCCGAATAT
TRINITY_DN2_c0_g2_i3         GAACATACGAAAATATTGGCCTACGTCTATGTTAGTACAATATCTATCATTGCCGAATAT
TRINITY_DN2_c0_g2_i2         GAACATACGAAAATATTGGCCTACGTCTATGTTAGTACAATATCTATCATTGCCGAATAT
TRINITY_DN2_c0_g2_i1         GAACATACGAAAATATTGGCCTACGTCTATGTTAGTACAATATCTATCATTGCCGAATAT
TRINITY_DN2_c0_g2_i5         GAACATACGAAAATATTGGCCTACGTCTATGTTAGTACAATATCTATCATTGCCGAATAT
                             ************************************************************

MpDNV_mRNA_seq_consensus     GCGTTTCCAACCGTTTTCACCCACAAATGTCAATTTTTGATCCGATCTCCTTTCGAACAA
TRINITY_DN2_c0_g1_i2         GCGTTTCCAACCGTTTTCACCCACAAATGTCAATTTTTGATCCGATCTCCTTTCGAACAA
```

```
TRINITY_DN2_c0_g1_i3         GCGTTTCCAACCGTTTTCACCCACAAATGTCAATTTTTGATCCGATCTCCTTTCGAACAA
TRINITY_DN2_c0_g1_i4         GCGTTTCCAACCGTTTTCACCCACAAATGTCAATTTTTGATCCGATCTCCTTTCGAACAA
TRINITY_DN2_c0_g1_i1         GCGTTTCCAACCGTTTTCACCCACAAATGTCAATTTTTGATCCGATCTCCTTTCGAACAA
TRINITY_DN39_c0_g1_i4        GCGTTTCCAACCGTTTTCACCCACAAATGTCAATTTTTGATCCGATCTCCTTTCGAACAA
TRINITY_DN39_c0_g1_i3        GCGTTTCCAACCGTTTTCACCCACAAATGTCAATTTTTGATCCGATCTCCTTTCGAACAA
TRINITY_DN39_c0_g1_i1        GCGTTTCCAACCGTTTTCACCCACAAATGTCAATTTTTGATCCGATCTCCTTTCGAACAA
TRINITY_DN39_c0_g1_i2        GCGTTTCCAACCGTTTTCACCCACAAATGTCAATTTTTGATCCGATCTCCTTTCGAACAA
TRINITY_DN2_c0_g2_i3         GCGTTTCCAACCGTTTTCACCCACAAATGTCAATTTTTGATCCGATCTCCTTTCGAACAA
TRINITY_DN2_c0_g2_i2         GCGTTTCCAACCGTTTTCACCCACAAATGTCAATTTTTGATCCGATCTCCTTTCGAACAA
TRINITY_DN2_c0_g2_i1         GCGTTTCCAACCGTTTTCACCCACAAATGTCAATTTTTGATCCGATCTCCTTTCGAACAA
TRINITY_DN2_c0_g2_i5         GCGTTTCCAACCGTTTTCACCCACAAATGTCAATTTTTGATCCGATCTCCTTTCGAACAA
                             ************************************************************

MpDNV_mRNA_seq_consensus     GCCACTGGCAGCGTTTATAAAATCTTGACACTCTGGTTCGTTACCAGCATTGCGTAACAT
TRINITY_DN2_c0_g1_i2         GCCACTGGCAGCGTTTATAAAATCTTGACACTCTGGTTCGTTACCAGCATTGCGTAACAT
TRINITY_DN2_c0_g1_i3         GCCACTGGCAGCGTTTATAAAATCTTGACACTCTGGTTCGTTACCAGCATTGCGTAACAT
TRINITY_DN2_c0_g1_i4         GCCACTGGCAGCGTTTATAAAATCTTGACACTCTGGTTCGTTACCAGCATTGCGTAACAT
TRINITY_DN2_c0_g1_i1         GCCACTGGCAGCGTTTATAAAATCTTGACACTCTGGTTCGTTACCAGCATTGCGTAACAT
TRINITY_DN39_c0_g1_i4        GCCACTGGCAGCGTTTATAAAATCTTGACACTCTGGTTCGTTACCAGCATTGCGTAACAT
TRINITY_DN39_c0_g1_i3        GCCACTGGCAGCGTTTATAAAATCTTGACACTCTGGTTCGTTACCAGCATTGCGTAACAT
TRINITY_DN39_c0_g1_i1        GCCACTGGCAGCGTTTATAAAATCTTGACACTCTGGTTCGTTACCAGCATTGCGTAACAT
TRINITY_DN39_c0_g1_i2        GCCACTGGCAGCGTTTATAAAATCTTGACACTCTGGTTCGTTACCAGCATTGCGTAACAT
TRINITY_DN2_c0_g2_i3         GCCACTGGCAGCGTTTATAAAATCTTGACACTCTGGTTCGTTACCAGCATTGCGTAACAT
TRINITY_DN2_c0_g2_i2         GCCACTGGCAGCGTTTATAAAATCTTGACACTCTGGTTCGTTACCAGCATTGCGTAACAT
TRINITY_DN2_c0_g2_i1         GCCACTGGCAGCGTTTATAAAATCTTGACACTCTGGTTCGTTACCAGCATTGCGTAACAT
TRINITY_DN2_c0_g2_i5         GCCACTGGCAGCGTTTATAAAATCTTGACACTCTGGTTCGTTACCAGCATTGCGTAACAT
                             ************************************************************

MpDNV_mRNA_seq_consensus     AGCAAATTTACTGGAATTAGGCTCCTGTACGCTGACACACGCATCGAATATTGGATCGTG
TRINITY_DN2_c0_g1_i2         AGCAAATTTACTGGAATTAGGCTCCTGTACGCTGACACACGCATCGAATATTGGATCGTG
TRINITY_DN2_c0_g1_i3         AGCAAATTTACTGGAATTAGGCTCCTGTACGCTGACACACGCATCGAATATTGGATCGTG
TRINITY_DN2_c0_g1_i4         AGCAAATTTACTGGAATTAGGCTCCTGTACGCTGACACACGCATCGAATATTGGATCGTG
TRINITY_DN2_c0_g1_i1         AGCAAATTTACTGGAATTAGGCTCCTGTACGCTGACACACGCATCGAATATTGGATCGTG
TRINITY_DN39_c0_g1_i4        AGCAAATTTACTGGAATTAGGCTCCTGTACGCTGACACACGCATCGAATATTGGATCGTG
TRINITY_DN39_c0_g1_i3        AGCAAATTTACTGGAATTAGGCTCCTGTACGCTGACACACGCATCGAATATTGGATCGTG
TRINITY_DN39_c0_g1_i1        AGCAAATTTACTGGAATTAGGCTCCTGTACGCTGACACACGCATCGAATATTGGATCGTG
TRINITY_DN39_c0_g1_i2        AGCAAATTTACTGGAATTAGGCTCCTGTACGCTGACACACGCATCGAATATTGGATCGTG
TRINITY_DN2_c0_g2_i3         AGCAAATTTACTGGAATTAGGCTCCTGTACGCTGACACACGCATCGAATATTGGATCGTG
TRINITY_DN2_c0_g2_i2         AGCAAATTTACTGGAATTAGGCTCCTGTACGCTGACACACGCATCGAATATTGGATCGTG
TRINITY_DN2_c0_g2_i1         AGCAAATTTACTGGAATTAGGCTCCTGTACGCTGACACACGCATCGAATATTGGATCGTG
TRINITY_DN2_c0_g2_i5         AGCAAATTTACTGGAATTAGGCTCCTGTACGCTGACACACGCATCGAATATTGGATCGTG
                             ************************************************************

MpDNV_mRNA_seq_consensus     TGGTAGAGTGAGTATACCGACATGAGGCTTGTACGAATAATTAAGTACAGTGGTGCCGAC
TRINITY_DN2_c0_g1_i2         TGGTAGAGTGAGTATACCGACATGAGGCTTGTACGAATAATTAAGTACAGTGGTGCCGAC
TRINITY_DN2_c0_g1_i3         TGGTAGAGTGAGTATACCGACATGAGGCTTGTACGAATAATTAAGTACAGTGGTGCCGAC
TRINITY_DN2_c0_g1_i4         TGGTAGAGTGAGTATACCGACATGAGGCTTGTACGAATAATTAAGTACAGTGGTGCCGAC
TRINITY_DN2_c0_g1_i1         TGGTAGAGTGAGTATACCGACATGAGGCTTGTACGAATAATTAAGTACAGTGGTGCCGAC
TRINITY_DN39_c0_g1_i4        TGGTAGAGTGAGTATACCGACATGAGGCTTGTACGAATAATTAAGTACAGTGGTGCCGAC
TRINITY_DN39_c0_g1_i3        TGGTAGAGTGAGTATACCGACATGAGGCTTGTACGAATAATTAAGTACAGTGGTGCCGAC
TRINITY_DN39_c0_g1_i1        TGGTAGAGTGAGTATACCGACATGAGGCTTGTACGAATAATTAAGTACAGTGGTGCCGAC
TRINITY_DN39_c0_g1_i2        TGGTAGAGTGAGTATACCGACATGAGGCTTGTACGAATAATTAAGTACAGTGGTGCCGAC
TRINITY_DN2_c0_g2_i3         TGGTAGAGTGAGTATACCGACATGAGGCTTGTACGAATAATTAAGTACAGTGGTGCCGAC
TRINITY_DN2_c0_g2_i2         TGGTAGAGTGAGTATACCGACATGAGGCTTGTACGAATAATTAAGTACAGTGGTGCCGAC
TRINITY_DN2_c0_g2_i1         TGGTAGAGTGAGTATACCGACATGAGGCTTGTACGAATAATTAAGTACAGTGGTGCCGAC
TRINITY_DN2_c0_g2_i5         TGGTAGAGTGAGTATACCGACATGAGGCTTGTACGAATAATTAAGTACAGTGGTGCCGAC
                             ************************************************************

MpDNV_mRNA_seq_consensus     AGTAAACGAAGCGTCGACCTTGGTGATGTGTTCATTAAACCGTTCCCATCCGAGTTTTCC
TRINITY_DN2_c0_g1_i2         AGTAAACGAAGCGTCGACCTTGGTGATGTGTTCATTAAACCGTTCCCATCCGAGTTTTCC
TRINITY_DN2_c0_g1_i3         AGTAAACGAAGCGTCGACCTTGGTGATGTGTTCATTAAACCGTTCCCATCCGAGTTTTCC
TRINITY_DN2_c0_g1_i4         AGTAAACGAAGCGTCGACCTTGGTGATGTGTTCATTAAACCGTTCCCATCCGAGTTTTCC
TRINITY_DN2_c0_g1_i1         AGTAAACGAAGCGTCGACCTTGGTGATGTGTTCATTAAACCGTTCCCATCCGAGTTTTCC
TRINITY_DN39_c0_g1_i4        AGTAAACGAAGCGTCGACCTTGGTGATGTGTTCATTAAACCGTTCCCATCCGAGTTTTCC
TRINITY_DN39_c0_g1_i3        AGTAAACGAAGCGTCGACCTTGGTGATGTGTTCATTAAACCGTTCCCATCCGAGTTTTCC
TRINITY_DN39_c0_g1_i1        AGTAAACGAAGCGTCGACCTTGGTGATGTGTTCATTAAACCGTTCCCATCCGAGTTTTCC
TRINITY_DN39_c0_g1_i2        AGTAAACGAAGCGTCGACCTTGGTGATGTGTTCATTAAACCGTTCCCATCCGAGTTTTCC
TRINITY_DN2_c0_g2_i3         AGTAAACGAAGCGTCGACCTTGGTGATGTGTTCATTAAACCGTTCCCATCCGAGTTTTCC
TRINITY_DN2_c0_g2_i2         AGTAAACGAAGCGTCGACCTTGGTGATGTGTTCATTAAACCGTTCCCATCCGAGTTTTCC
TRINITY_DN2_c0_g2_i1         AGTAAACGAAGCGTCGACCTTGGTGATGTGTTCATTAAACCGTTCCCATCCGAGTTTTCC
TRINITY_DN2_c0_g2_i5         AGTAAACGAAGCGTCGACCTTGGTGATGTGTTCATTAAACCGTTCCCATCCGAGTTTTCC
                             ************************************************************

MpDNV_mRNA_seq_consensus     AGAATTATGATTTCTATTTACAAAATTACAAAAATAACTGTTATACATCAACGGTAAGTT
TRINITY_DN2_c0_g1_i2         AGAATTATGATTTCTATTTACAAAATTACAAAAATAACTGTTATACATCAACGGTAAGTT
TRINITY_DN2_c0_g1_i3         AGAATTATGATTTCTATTTACAAAATTACAAAAATAACTGTTATACATCAACGGTAAGTT
TRINITY_DN2_c0_g1_i4         AGAATTATGATTTCTATTTACAAAATTACAAAAATAACTGTTATACATCAACGGTAAGTT
TRINITY_DN2_c0_g1_i1         AGAATTATGATTTCTATTTACAAAATTACAAAAATAACTGTTATACATCAACGGTAAGTT
TRINITY_DN39_c0_g1_i4        AGAATTATGATTTCTATTTACAAAATTACAAAAATAACTGTTATACATCAACGGTAAGTT
TRINITY_DN39_c0_g1_i3        AGAATTATGATTTCTATTTACAAAATTACAAAAATAACTGTTATACATCAACGGTAAGTT
TRINITY_DN39_c0_g1_i1        AGAATTATGATTTCTATTTACAAAATTACAAAAATAACTGTTATACATCAACGGTAAGTT
TRINITY_DN39_c0_g1_i2        AGAATTATGATTTCTATTTACAAAATTACAAAAATAACTGTTATACATCAACGGTAAGTT
TRINITY_DN2_c0_g2_i3         AGAATTATGATTTCTATTTACAAAATTACAAAAATAACTGTTATACATCAACGGTAAGTT
TRINITY_DN2_c0_g2_i2         AGAATTATGATTTCTATTTACAAAATTACAAAAATAACTGTTATACATCAACGGTAAGTT
TRINITY_DN2_c0_g2_i1         AGAATTATGATTTCTATTTACAAAATTACAAAAATAACTGTTATACATCAACGGTAAGTT
TRINITY_DN2_c0_g2_i5         AGAATTATGATTTCTATTTACAAAATTACAAAAATAACTGTTATACATCAACGGTAAGTT
                             ************************************************************
```

```
MpDNV_mRNA_seq_consensus      CATGTAGGACGCAGGCAGTTCATATCCCTCTTTAGCAGAGAATGTACCATCTAGATCTTG
TRINITY_DN2_c0_g1_i2          CATGTAGGACGCAGGCAGTTCATATCCCTCTTTAGCAGAGAATGTACCATCTAGATCTTG
TRINITY_DN2_c0_g1_i3          CATGTAGGACGCAGGCAGTTCATATCCCTCTTTAGCAGAGAATGTACCATCTAGATCTTG
TRINITY_DN2_c0_g1_i4          CATGTAGGACGCAGGCAGTTCATATCCCTCTTTAGCAGAGAATGTACCATCTAGATCTTG
TRINITY_DN2_c0_g1_i1          CATGTAGGACGCAGGCAGTTCATATCCCTCTTTAGCAGAGAATGTACCATCTAGATCTTG
TRINITY_DN39_c0_g1_i4         CATGTAGGACGCAGGCAGTTCATATCCCTCTTTAGCAGAGAATGTACCATCTAGATCTTG
TRINITY_DN39_c0_g1_i3         CATGTAGGACGCAGGCAGTTCATATCCCTCTTTAGCAGAGAATGTACCATCTAGATCTTG
TRINITY_DN39_c0_g1_i1         CATGTAGGACGCAGGCAGTTCATATCCCTCTTTAGCAGAGAATGTACCATCTAGATCTTG
TRINITY_DN39_c0_g1_i2         CATGTAGGACGCAGGCAGTTCATATCCCTCTTTAGCAGAGAATGTACCATCTAGATCTTG
TRINITY_DN2_c0_g2_i3          CATGTAGGACGCAGGCAGTTCATATCCCTCTTTAGCAGAGAATGTACCATCTAGATCTTG
TRINITY_DN2_c0_g2_i2          CATGTAGGACGCAGGCAGTTCATATCCCTCTTTAGCAGAGAATGTACCATCTAGATCTTG
TRINITY_DN2_c0_g2_i1          CATGTAGGACGCAGGCAGTTCATATCCCTCTTTAGCAGAGAATGTACCATCTAGATCTTG
TRINITY_DN2_c0_g2_i5          CATGTAGGACGCAGGCAGTTCATATCCCTCTTTAGCAGAGAATGTACCATCTAGATCTTG
                              ************************************************************

MpDNV_mRNA_seq_consensus      ACCGTACAATTTGCGAACGAATTCTTTCATAGACGACTGATCGTTCTCGGTATTCGTTGG
TRINITY_DN2_c0_g1_i2          ACCGTACAATTTGCGAACGAATTCTTTCATAGACGACTGATCGTTCTCGGTATTCGTTGG
TRINITY_DN2_c0_g1_i3          ACCGTACAATTTGCGAACGAATTCTTTCATAGACGACTGATCGTTCTCGGTATTCGTTGG
TRINITY_DN2_c0_g1_i4          ACCGTACAATTTGCGAACGAATTCTTTCATAGACGACTGATCGTTCTCGGTATTCGTTGG
TRINITY_DN2_c0_g1_i1          ACCGTACAATTTGCGAACGAATTCTTTCATAGACGACTGATCGTTCTCGGTATTCGTTGG
TRINITY_DN39_c0_g1_i4         ACCGTACAATTTGCGAACGAATTCTTTCATAGACGACTGATCGTTCTCGGTATTCGTTGG
TRINITY_DN39_c0_g1_i3         ACCGTACAATTTGCGAACGAATTCTTTCATAGACGACTGATCGTTCTCGGTATTCGTTGG
TRINITY_DN39_c0_g1_i1         ACCGTACAATTTGCGAACGAATTCTTTCATAGACGACTGATCGTTCTCGGTATTCGTTGG
TRINITY_DN39_c0_g1_i2         ACCGTACAATTTGCGAACGAATTCTTTCATAGACGACTGATCGTTCTCGGTATTCGTTGG
TRINITY_DN2_c0_g2_i3          ACCGTACAATTTGCGAACGAATTCTTTCATAGACGACTGATCGTTCTCGGTATTCGTTGG
TRINITY_DN2_c0_g2_i2          ACCGTACAATTTGCGAACGAATTCTTTCATAGACGACTGATCGTTCTCGGTATTCGTTGG
TRINITY_DN2_c0_g2_i1          ACCGTACAATTTGCGAACGAATTCTTTCATAGACGACTGATCGTTCTCGGTATTCGTTGG
TRINITY_DN2_c0_g2_i5          ACCGTACAATTTGCGAACGAATTCTTTCATAGACGACTGATCGTTCTCGGTATTCGTTGG
                              ************************************************************

MpDNV_mRNA_seq_consensus      ATCCATGGGCTCCGATGAATTGCCAAATGTGAGTTTTCGGTTAAATCCCCTTGTTTTTAA
TRINITY_DN2_c0_g1_i2          ATCCATGGGCTCCGATGAATTGCCAAATGTGAGTTTTCGGTTAAATCCCCTTGTTTTTAA
TRINITY_DN2_c0_g1_i3          ATCCATGGGCTCCGATGAATTGCCAAATGTGAGTTTTCGGTTAAATCCCCTTGTTTTTAA
TRINITY_DN2_c0_g1_i4          ATCCATGGGCTCCGATGAATTGCCAAATGTGAGTTTTCGGTTAAATCCCCTTGTTTTTAA
TRINITY_DN2_c0_g1_i1          ATCCATGGGCTCCGATGAATTGCCAAATGTGAGTTTTCGGTTAAATCCCCTTGTTTTTAA
TRINITY_DN39_c0_g1_i4         ATCCATGGGCTCCGATGAATTGCCAAATGTGAGTTTTCGGTTAAATCCCCTTGTTTTTAA
TRINITY_DN39_c0_g1_i3         ATCCATGGGCTCCGATGAATTGCCAAATGTGAGTTTTCGGTTAAATCCCCTTGTTTTTAA
TRINITY_DN39_c0_g1_i1         ATCCATGGGCTCCGATGAATTGCCAAATGTGAGTTTTCGGTTAAATCCCCTTGTTTTTAA
TRINITY_DN39_c0_g1_i2         ATCCATGGGCTCCGATGAATTGCCAAATGTGAGTTTTCGGTTAAATCCCCTTGTTTTTAA
TRINITY_DN2_c0_g2_i3          ATCCATGGGCTCCGATGAATTGCCAAATGTGAGTTTTCGGTTAAATCCCCTTGTTTTTAA
TRINITY_DN2_c0_g2_i2          ATCCATGGGCTCCGATGAATTGCCAAATGTGAGTTTTCGGTTAAATCCCCTTGTTTTTAA
TRINITY_DN2_c0_g2_i1          ATCCATGGGCTCCGATGAATTGCCAAATGTGAGTTTTCGGTTAAATCCCCTTGTTTTTAA
TRINITY_DN2_c0_g2_i5          ATCCATGGGCTCCGATGAATTGCCAAATGTGAGTTTTCGGTTAAATCCCCTTGTTTTTAA
                              ************************************************************

MpDNV_mRNA_seq_consensus      ATTCAAAGCGTCCGCTTTTACCATAAATTTGTTCTGATTCAATGTGGCCAGGGAGCTCGT
TRINITY_DN2_c0_g1_i2          ATTCAAAGCGTCCGCTTTTACCATAAATTTGTTCTGATTCAATGTGGCCAGGGAGCTCGT
TRINITY_DN2_c0_g1_i3          ATTCAAAGCGTCCGCTTTTACCATAAATTTGTTCTGATTCAATGTGGCCAGGGAGCTCGT
TRINITY_DN2_c0_g1_i4          ATTCAAAGCGTCCGCTTTTACCATAAATTTGTTCTGATTCAATGTGGCCAGGGAGCTCGT
TRINITY_DN2_c0_g1_i1          ATTCAAAGCGTCCGCTTTTACCATAAATTTGTTCTGATTCAATGTGGCCAGGGAGCTCGT
TRINITY_DN39_c0_g1_i4         ATTCAAAGCGTCCGCTTTTACCATAAATTTGTTCTGATTCAATGTGGCCAGGGAGCTCGT
TRINITY_DN39_c0_g1_i3         ATTCAAAGCGTCCGCTTTTACCATAAATTTGTTCTGATTCAATGTGGCCAGGGAGCTCGT
TRINITY_DN39_c0_g1_i1         ATTCAAAGCGTCCGCTTTTACCATAAATTTGTTCTGATTCAATGTGGCCAGGGAGCTCGT
TRINITY_DN39_c0_g1_i2         ATTCAAAGCGTCCGCTTTTACCATAAATTTGTTCTGATTCAATGTGGCCAGGGAGCTCGT
TRINITY_DN2_c0_g2_i3          ATTCAAAGCGTCCGCTTTTACCATAAATTTGTTCTGATTCAATGTGGCCAGGGAGCTCGT
TRINITY_DN2_c0_g2_i2          ATTCAAAGCGTCCGCTTTTACCATAAATTTGTTCTGATTCAATGTGGCCAGGGAGCTCGT
TRINITY_DN2_c0_g2_i1          ATTCAAAGCGTCCGCTTTTACCATAAATTTGTTCTGATTCAATGTGGCCAGGGAGCTCGT
TRINITY_DN2_c0_g2_i5          ATTCAAAGCGTCCGCTTTTACCATAAATTTGTTCTGATTCAATGTGGCCAGGGAGCTCGT
                              ************************************************************

MpDNV_mRNA_seq_consensus      AGAGCTATTGGTTTCAAAAGCTGTGCGAGGATTACGCATGACACCCTTGCATTTGACTTC
TRINITY_DN2_c0_g1_i2          AGAGCTATTGGTTTCAAAAGCTGTGCGAGGATTACGCATGACACCCTTGCATTTGACTTC
TRINITY_DN2_c0_g1_i3          AGAGCTATTGGTTTCAAAAGCTGTGCGAGGATTACGCATGACACCCTTGCATTTGACTTC
TRINITY_DN2_c0_g1_i4          AGAGCTATTGGTTTCAAAAGCTGTGCGAGGATTACGCATGACACCCTTGCATTTGACTTC
TRINITY_DN2_c0_g1_i1          AGAGCTATTGGTTTCAAAAGCTGTGCGAGGATTACGCATGACACCCTTGCATTTGACTTC
TRINITY_DN39_c0_g1_i4         AGAGCTATTGGTTTCAAAAGCTGTGCGAGGATTACGCATGACACCCTTGCATTTGACTTC
TRINITY_DN39_c0_g1_i3         AGAGCTATTGGTTTCAAAAGCTGTGCGAGGATTACGCATGACACCCTTGCATTTGACTTC
TRINITY_DN39_c0_g1_i1         AGAGCTATTGGTTTCAAAAGCTGTGCGAGGATTACGCATGACACCCTTGCATTTGACTTC
TRINITY_DN39_c0_g1_i2         AGAGCTATTGGTTTCAAAAGCTGTGCGAGGATTACGCATGACACCCTTGCATTTGACTTC
TRINITY_DN2_c0_g2_i3          AGAGCTATTGGTTTCAAAAGCTGTGCGAGGATTACGCATGACACCCTTGCATTTGACTTC
TRINITY_DN2_c0_g2_i2          AGAGCTATTGGTTTCAAAAGCTGTGCGAGGATTACGCATGACACCCTTGCATTTGACTTC
TRINITY_DN2_c0_g2_i1          AGAGCTATTGGTTTCAAAAGCTGTGCGAGGATTACGCATGACACCCTTGCATTTGACTTC
TRINITY_DN2_c0_g2_i5          AGAGCTATTGGTTTCAAAAGCTGTGCGAGGATTACGCATGACACCCTTGCATTTGACTTC
                              ************************************************************

MpDNV_mRNA_seq_consensus      AGTGCATATAGCACCTCTGGGTAAAAATTTAAACTCGGACGGCGACATATAGAACCAAGG
TRINITY_DN2_c0_g1_i2          AGTGCATATAGCACCTCTGGGTAAAAATTTAAACTCGGACGGCGACATATAGAACCAAGG
TRINITY_DN2_c0_g1_i3          AGTGCATATAGCACCTCTGGGTAAAAATTTAAACTCGGACGGCGACATATAGAACCAAGG
TRINITY_DN2_c0_g1_i4          AGTGCATATAGCACCTCTGGGTAAAAATTTAAACTCGGACGGCGACATATAGAACCAAGG
TRINITY_DN2_c0_g1_i1          AGTGCATATAGCACCTCTGGGTAAAAATTTAAACTCGGACGGCGACATATAGAACCAAGG
TRINITY_DN39_c0_g1_i4         AGTGCATATAGCACCTCTGGGTAAAAATTTAAACTCGGACGGCGACATATAGAACCAAGG
TRINITY_DN39_c0_g1_i3         AGTGCATATAGCACCTCTGGGTAAAAATTTAAACTCGGACGGCGACATATAGAACCAAGG
TRINITY_DN39_c0_g1_i1         AGTGCATATAGCACCTCTGGGTAAAAATTTAAACTCGGACGGCGACATATAGAACCAAGG
TRINITY_DN39_c0_g1_i2         AGTGCATATAGCACCTCTGGGTAAAAATTTAAACTCGGACGGCGACATATAGAACCAAGG
TRINITY_DN2_c0_g2_i3          AGTGCATATAGCACCTCTGGGTAAAAATTTAAACTCGGACGGCGACATATAGAACCAAGG
TRINITY_DN2_c0_g2_i2          AGTGCATATAGCACCTCTGGGTAAAAATTTAAACTCGGACGGCGACATATAGAACCAAGG
TRINITY_DN2_c0_g2_i1          AGTGCATATAGCACCTCTGGGTAAAAATTTAAACTCGGACGGCGACATATAGAACCAAGG
TRINITY_DN2_c0_g2_i5          AGTGCATATAGCACCTCTGGGTAAAAATTTAAACTCGGACGGCGACATATAGAACCAAGG
```

```
                                    ************************************************************

MpDNV_mRNA_seq_consensus            TTGGTCCACGGGCACACTCATTAAACTCGTTGTCCCAAGATAGGCGTTATCAGTGCTAAG
TRINITY_DN2_c0_g1_i2                TTGGTCCACGGGCACACTCATTAAACTCGTTGTCCCAAGATAGGCGTTATCAGTGCTAAG
TRINITY_DN2_c0_g1_i3                TTGGTCCACGGGCACACTCATTAAACTCGTTGTCCCAAGATAGGCGTTATCAGTGCTAAG
TRINITY_DN2_c0_g1_i4                TTGGTCCACGGGCACACTCATTAAACTCGTTGTCCCAAGATAGGCGTTATCAGTGCTAAG
TRINITY_DN2_c0_g1_i1                TTGGTCCACGGGCACACTCATTAAACTCGTTGTCCCAAGATAGGCGTTATCAGTGCTAAG
TRINITY_DN39_c0_g1_i4               TTGGTCCACGGGCACACTCATTAAACTCGTTGTCCCAAGATAGGCGTTATCAGTGCTAAG
TRINITY_DN39_c0_g1_i3               TTGGTCCACGGGCACACTCATTAAACTCGTTGTCCCAAGATAGGCGTTATCAGTGCTAAG
TRINITY_DN39_c0_g1_i1               TTGGTCCACGGGCACACTCATTAAACTCGTTGTCCCAAGATAGGCGTTATCAGTGCTAAG
TRINITY_DN39_c0_g1_i2               TTGGTCCACGGGCACACTCATTAAACTCGTTGTCCCAAGATAGGCGTTATCAGTGCTAAG
TRINITY_DN2_c0_g2_i3                TTGGTCCACGGGCACACTCATTAAACTCGTTGTCCCAAGATAGGCGTTATCAGTGCTAAG
TRINITY_DN2_c0_g2_i2                TTGGTCCACGGGCACACTCATTAAACTCGTTGTCCCAAGATAGGCGTTATCAGTGCTAAG
TRINITY_DN2_c0_g2_i1                TTGGTCCACGGGCACACTCATTAAACTCGTTGTCCCAAGATAGGCGTTATCAGTGCTAAG
TRINITY_DN2_c0_g2_i5                TTGGTCCACGGGCACACTCATTAAACTCGTTGTCCCAAGATAGGCGTTATCAGTGCTAAG
                                    ************************************************************

MpDNV_mRNA_seq_consensus            ATATCGCAACACTTTCCAACCACATCCGTAACTGATGAAAGTATGTACTTTTCGGTAAAC
TRINITY_DN2_c0_g1_i2                ATATCGCAACACTTTCCAACCACATCCGTAACTGATGAAAGTATGTACTTTTCGGTAAAC
TRINITY_DN2_c0_g1_i3                ATATCGCAACACTTTCCAACCACATCCGTAACTGATGAAAGTATGTACTTTTCGGTAAAC
TRINITY_DN2_c0_g1_i4                ATATCGCAACACTTTCCAACCACATCCGTAACTGATGAAAGTATGTACTTTTCGGTAAAC
TRINITY_DN2_c0_g1_i1                ATATCGCAACACTTTCCAACCACATCCGTAACTGATGAAAGTATGTACTTTTCGGTAAAC
TRINITY_DN39_c0_g1_i4               ATATCGCAACACTTTCCAACCACATCCGTAACTGATGAAAGTATGTACTTTTCGGTAAAC
TRINITY_DN39_c0_g1_i3               ATATCGCAACACTTTCCAACCACATCCGTAACTGATGAAAGTATGTACTTTTCGGTAAAC
TRINITY_DN39_c0_g1_i1               ATATCGCAACACTTTCCAACCACATCCGTAACTGATGAAAGTATGTACTTTTCGGTAAAC
TRINITY_DN39_c0_g1_i2               ATATCGCAACACTTTCCAACCACATCCGTAACTGATGAAAGTATGTACTTTTCGGTAAAC
TRINITY_DN2_c0_g2_i3                ATATCGCAACACTTTCCAACCACATCCGTAACTGATGAAAGTATGTACTTTTCGGTAAAC
TRINITY_DN2_c0_g2_i2                ATATCGCAACACTTTCCAACCACATCCGTAACTGATGAAAGTATGTACTTTTCGGTAAAC
TRINITY_DN2_c0_g2_i1                ATATCGCAACACTTTCCAACCACATCCGTAACTGATGAAAGTATGTACTTTTCGGTAAAC
TRINITY_DN2_c0_g2_i5                ATATCGCAACACTTTCCAACCACATCCGTAACTGATGAAAGTATGTACTTTTCGGTAAAC
                                    ************************************************************

MpDNV_mRNA_seq_consensus            CATAGTATATCCTCCCGTGTGACCGATTGGCCTAGGTATAACAGCGTTTTCAGAGCTAGG
TRINITY_DN2_c0_g1_i2                CATAGTATATCCTCCCGTGTGACCGATTGGCCTAGGTATAACAGCGTTTTCAGAGCTAGG
TRINITY_DN2_c0_g1_i3                CATAGTATATCCTCCCGTGTGACCGATTGGCCTAGGTATAACAGCGTTTTCAGAGCTAGG
TRINITY_DN2_c0_g1_i4                CATAGTATATCCTCCCGTGTGACCGATTGGCCTAGGTATAACAGCGTTTTCAGAGCTAGG
TRINITY_DN2_c0_g1_i1                CATAGTATATCCTCCCGTGTGACCGATTGGCCTAGGTATAACAGCGTTTTCAGAGCTAGG
TRINITY_DN39_c0_g1_i4               CATAGTATATCCTCCCGTGTGACCGATTGGCCTAGGTATAACAGCGTTTTCAGAGCTAGG
TRINITY_DN39_c0_g1_i3               CATAGTATATCCTCCCGTGTGACCGATTGGCCTAGGTATAACAGCGTTTTCAGAGCTAGG
TRINITY_DN39_c0_g1_i1               CATAGTATATCCTCCCGTGTGACCGATTGGCCTAGGTATAACAGCGTTTTCAGAGCTAGG
TRINITY_DN39_c0_g1_i2               CATAGTATATCCTCCCGTGTGACCGATTGGCCTAGGTATAACAGCGTTTTCAGAGCTAGG
TRINITY_DN2_c0_g2_i3                CATAGTATATCCTCCCGTGTGACCGATTGGCCTAGGTATAACAGCGTTTTCAGAGCTAGG
TRINITY_DN2_c0_g2_i2                CATAGTATATCCTCCCGTGTGACCGATTGGCCTAGGTATAACAGCGTTTTCAGAGCTAGG
TRINITY_DN2_c0_g2_i1                CATAGTATATCCTCCCGTGTGACCGATTGGCCTAGGTATAACAGCGTTTTCAGAGCTAGG
TRINITY_DN2_c0_g2_i5                CATAGTATATCCTCCCGTGTGACCGATTGGCCTAGGTATAACAGCGTTTTCAGAGCTAGG
                                    ************************************************************

MpDNV_mRNA_seq_consensus            ATTGCCAGTATCTGGGTCCCCATCGTTCGCAGCAGCCGTCCCAGGAAGCGAGGTGCCACC
TRINITY_DN2_c0_g1_i2                ATTGCCAGTATCTGGGTCCCCATCGTTCGCAGCAGCCGTCCCAGGAAGCGAGGTGCCACC
TRINITY_DN2_c0_g1_i3                ATTGCCAGTATCTGGGTCCCCATCGTTCGCAGCAGCCGTCCCAGGAAGCGAGGTGCCACC
TRINITY_DN2_c0_g1_i4                ATTGCCAGTATCTGGGTCCCCATCGTTCGCAGCAGCCGTCCCAGGAAGCGAGGTGCCACC
TRINITY_DN2_c0_g1_i1                ATTGCCAGTATCTGGGTCCCCATCGTTCGCAGCAGCCGTCCCAGGAAGCGAGGTGCCACC
TRINITY_DN39_c0_g1_i4               ATTGCCAGTATCTGGGTCCCCATCGTTCGCAGCAGCCGTCCCAGGAAGCGAGGTGCCACC
TRINITY_DN39_c0_g1_i3               ATTGCCAGTATCTGGGTCCCCATCGTTCGCAGCAGCCGTCCCAGGAAGCGAGGTGCCACC
TRINITY_DN39_c0_g1_i1               ATTGCCAGTATCTGGGTCCCCATCGTTCGCAGCAGCCGTCCCAGGAAGCGAGGTGCCACC
TRINITY_DN39_c0_g1_i2               ATTGCCAGTATCTGGGTCCCCATCGTTCGCAGCAGCCGTCCCAGGAAGCGAGGTGCCACC
TRINITY_DN2_c0_g2_i3                ATTGCCAGTATCTGGGTCCCCATCGTTCGCAGCAGCCGTCCCAGGAAGCGAGGTGCCACC
TRINITY_DN2_c0_g2_i2                ATTGCCAGTATCTGGGTCCCCATCGTTCGCAGCAGCCGTCCCAGGAAGCGAGGTGCCACC
TRINITY_DN2_c0_g2_i1                ATTGCCAGTATCTGGGTCCCCATCGTTCGCAGCAGCCGTCCCAGGAAGCGAGGTGCCACC
TRINITY_DN2_c0_g2_i5                ATTGCCAGTATCTGGGTCCCCATCGTTCGCAGCAGCCGTCCCAGGAAGCGAGGTGCCACC
                                    ************************************************************

MpDNV_mRNA_seq_consensus            CGCACGTTTAGGAGCGGGTTCAGAACCGGAGCTATCGGCAGGTCGTTTGCTACTTGATCC
TRINITY_DN2_c0_g1_i2                CGCACGTTTAGGAGCGGGTTCAGAACCGGAGCTATCGGCAGGTCGTTTGCTACTTGATCC
TRINITY_DN2_c0_g1_i3                CGCACGTTTAGGAGCGGGTTCAGAACCGGAGCTATCGGCAGGTCGTTTGCTACTTGATCC
TRINITY_DN2_c0_g1_i4                CGCACGTTTAGGAGCGGGTTCAGAACCGGAGCTATCGGCAGGTCGTTTGCTACTTGATCC
TRINITY_DN2_c0_g1_i1                CGCACGTTTAGGAGCGGGTTCAGAACCGGAGCTATCGGCAGGTCGTTTGCTACTTGATCC
TRINITY_DN39_c0_g1_i4               CGCACGTTTAGGAGCGGGTTCAGAACCGGAGCTATCGGCAGGTCGTTTGCTACTTGATCC
TRINITY_DN39_c0_g1_i3               CGCACGTTTAGGAGCGGGTTCAGAACCGGAGCTATCGGCAGGTCGTTTGCTACTTGATCC
TRINITY_DN39_c0_g1_i1               CGCACGTTTAGGAGCGGGTTCAGAACCGGAGCTATCGGCAGGTCGTTTGCTACTTGATCC
TRINITY_DN39_c0_g1_i2               CGCACGTTTAGGAGCGGGTTCAGAACCGGAGCTATCGGCAGGTCGTTTGCTACTTGATCC
TRINITY_DN2_c0_g2_i3                CGCACGTTTAGGAGCGGGTTCAGAACCGGAGCTATCGGCAGGTCGTTTGCTACTTGATCC
TRINITY_DN2_c0_g2_i2                CGCACGTTTAGGAGCGGGTTCAGAACCGGAGCTATCGGCAGGTCGTTTGCTACTTGATCC
TRINITY_DN2_c0_g2_i1                CGCACGTTTAGGAGCGGGTTCAGAACCGGAGCTATCGGCAGGTCGTTTGCTACTTGATCC
TRINITY_DN2_c0_g2_i5                CGCACGTTTAGGAGCGGGTTCAGAACCGGAGCTATCGGCAGGTCGTTTGCTACTTGATCC
                                    ************************************************************

MpDNV_mRNA_seq_consensus            GCTAGAGCTTGCCATGGATCCCGATGCGCTTCCCCCGTTGCCGTTGCTAGTGCTGTTGTC
TRINITY_DN2_c0_g1_i2                GCTAGAGCTTGCCATGGATCCCGATGCGCTTCCCCCGTTGCCGTTGCTAGTGCTGTTGTC
TRINITY_DN2_c0_g1_i3                GCTAGAGCTTGCCATGGATCCCGATGCGCTTCCCCCGTTGCCGTTGCTAGTGCTGTTGTC
TRINITY_DN2_c0_g1_i4                GCTAGAGCTTGCCATGGATCCCGATGCGCTTCCCCCGTTGCCGTTGCTAGTGCTGTTGTC
TRINITY_DN2_c0_g1_i1                GCTAGAGCTTGCCATGGATCCCGATGCGCTTCCCCCGTTGCCGTTGCTAGTGCTGTTGTC
TRINITY_DN39_c0_g1_i4               GCTAGAGCTTGCCATGGATCCCGATGCGCTTCCCCCGTTGCCGTTGCTAGTGCTGTTGTC
TRINITY_DN39_c0_g1_i3               GCTAGAGCTTGCCATGGATCCCGATGCGCTTCCCCCGTTGCCGTTGCTAGTGCTGTTGTC
TRINITY_DN39_c0_g1_i1               GCTAGAGCTTGCCATGGATCCCGATGCGCTTCCCCCGTTGCCGTTGCTAGTGCTGTTGTC
TRINITY_DN39_c0_g1_i2               GCTAGAGCTTGCCATGGATCCCGATGCGCTTCCCCCGTTGCCGTTGCTAGTGCTGTTGTC
TRINITY_DN2_c0_g2_i3                GCTAGAGCTTGCCATGGATCCCGATGCGCTTCCCCCGTTGCCGTTGCTAGTGCTGTTGTC
TRINITY_DN2_c0_g2_i2                GCTAGAGCTTGCCATGGATCCCGATGCGCTTCCCCCGTTGCCGTTGCTAGTGCTGTTGTC
```

```
TRINITY_DN2_c0_g2_i1          GCTAGAGCTTGCCATGGATCCCGATGCGCTTCCCCCGTTGCCGTTGCTAGTGCTGTTGTC
TRINITY_DN2_c0_g2_i5          GCTAGAGCTTGCCATGGATCCCGATGCGCTTCCCCCGTTGCCGTTGCTAGTGCTGTTGTC
                              ************************************************************

MpDNV_mRNA_seq_consensus      CGGGGCTGAGGTGTTTGCCACGTGGTCGCCACTCGCTTCACCGCTGTTAGGATTGCCGTT
TRINITY_DN2_c0_g1_i2          CGGGGCTGAGGTGTTTGCCACGTGGTCGCCACTCGCTTCACCGCTGTTAGGATTGCCGTT
TRINITY_DN2_c0_g1_i3          CGGGGCTGAGGTGTTTGCCACGTGGTCGCCACTCGCTTCACCGCTGTTAGGATTGCCGTT
TRINITY_DN2_c0_g1_i4          CGGGGCTGAGGTGTTTGCCACGTGGTCGCCACTCGCTTCACCGCTGTTAGGATTGCCGTT
TRINITY_DN2_c0_g1_i1          CGGGGCTGAGGTGTTTGCCACGTGGTCGCCACTCGCTTCACCGCTGTTAGGATTGCCGTT
TRINITY_DN39_c0_g1_i4         CGGGGCTGAGGTGTTTGCCACGTGGTCGCCACTCGCTTCACCGCTGTTAGGATTGCCGTT
TRINITY_DN39_c0_g1_i3         CGGGGCTGAGGTGTTTGCCACGTGGTCGCCACTCGCTTCACCGCTGTTAGGATTGCCGTT
TRINITY_DN39_c0_g1_i1         CGGGGCTGAGGTGTTTGCCACGTGGTCGCCACTCGCTTCACCGCTGTTAGGATTGCCGTT
TRINITY_DN39_c0_g1_i2         CGGGGCTGAGGTGTTTGCCACGTGGTCGCCACTCGCTTCACCGCTGTTAGGATTGCCGTT
TRINITY_DN2_c0_g2_i3          CGGGGCTGAGGTGTTTGCCACGTGGTCGCCACTCGCTTCACCGCTGTTAGGATTGCCGTT
TRINITY_DN2_c0_g2_i2          CGGGGCTGAGGTGTTTGCCACGTGGTCGCCACTCGCTTCACCGCTGTTAGGATTGCCGTT
TRINITY_DN2_c0_g2_i1          CGGGGCTGAGGTGTTTGCCACGTGGTCGCCACTCGCTTCACCGCTGTTAGGATTGCCGTT
TRINITY_DN2_c0_g2_i5          CGGGGCTGAGGTGTTTGCCACGTGGTCGCCACTCGCTTCACCGCTGTTAGGATTGCCGTT
                              ************************************************************

MpDNV_mRNA_seq_consensus      ACCGTCGTAGTCCCAATCCAAATCAGGATCCTCCTCCACGCCACCTAAAACATACGGTGG 3’ splice site
TRINITY_DN2_c0_g1_i2          ACCGTCGTAGTCCCAATCCAAATCAGGATCCTCCTCCACGCCACCTAAAACATACGGTGG unspliced
TRINITY_DN2_c0_g1_i3          ACCGTCGTAGTCCCAATCCAAATCAGGATCCTCCTCCACGCCAC---------------- spliced intron 1
TRINITY_DN2_c0_g1_i4          ACCGTCGTAGTCCCAATCCAAATCAGGATCCTCCTCCACGCCACCTAAAACATACGGTGG unspliced
TRINITY_DN2_c0_g1_i1          ACCGTCGTAGTCCCAATCCAAATCAGGATCCTCCTCCACGCCAC---------------- spliced intron 1
TRINITY_DN39_c0_g1_i4         ACCGTCGTAGTCCCAATCCAAATCAGGATCCTCCTCCACGCCAC---------------- spliced intron 1
TRINITY_DN39_c0_g1_i3         ACCGTCGTAGTCCCAATCCAAATCAGGATCCTCCTCCACGCCAC---------------- spliced intron 1
TRINITY_DN39_c0_g1_i1         ACCGTCGTAGTCCCAATCCAAATCAGGATCCTCCTCCACGCCACCTAAAACATACGGTGG unspliced
TRINITY_DN39_c0_g1_i2         ACCGTCGTAGTCCCAATCCAAATCAGGATCCTCCTCCACGCCACCTAAAACATACGGTGG unspliced
TRINITY_DN2_c0_g2_i3          ACCGTCGTAGTCCCAATCCAAATCAGGATCCTCCTCCACGCCAC---------------- spliced intron 1
TRINITY_DN2_c0_g2_i2          ACCGTCGTAGTCCCAATCCAAATCAGGATCCTCCTCCACGCCACCTAAAACATACGGTGG unspliced
TRINITY_DN2_c0_g2_i1          ACCGTCGTAGTCCCAATCCAAATCAGGATCCTCCTCCACGCCAC---------------- spliced intron 1
TRINITY_DN2_c0_g2_i5          ACCGTCGTAGTCCCAATCCAAATCAGGATCCTCCTCCACGCCACCTAAAACATACGGTGG unspliced
                              ********************************************

MpDNV_mRNA_seq_consensus      TAATGAACGACGTGCTAAAGCCTTATTGTATTGTTCTAGCGCGTAACGCTGCTGCGATGA
TRINITY_DN2_c0_g1_i2          TAATGAACGACGTGCTAAAGCCTTATTGTATTGTTCTAGCGCGTAACGCTGCTGCGATGA
TRINITY_DN2_c0_g1_i3          ------------------------------------------------------------
TRINITY_DN2_c0_g1_i4          TAATGAACGACGTGCTAAAGCCTTATTGTATTGTTCTAGCGCGTAACGCTGCTGCGATGA
TRINITY_DN2_c0_g1_i1          ------------------------------------------------------------
TRINITY_DN39_c0_g1_i4         ------------------------------------------------------------
TRINITY_DN39_c0_g1_i3         ------------------------------------------------------------
TRINITY_DN39_c0_g1_i1         TAATGAACGACGTGCTAAAGCCTTATTGTATTGTTCTAGCGCGTAACGCTGCTGCGATGA
TRINITY_DN39_c0_g1_i2         TAATGAACGACGTGCTAAAGCCTTATTGTATTGTTCTAGCGCGTAACGCTGCTGCGATGA
TRINITY_DN2_c0_g2_i3          ------------------------------------------------------------
TRINITY_DN2_c0_g2_i2          TAATGAACGACGTGCTAAAGCCTTATTGTATTGTTCTAGCGCGTAACGCTGCTGCGATGA
TRINITY_DN2_c0_g2_i1          ------------------------------------------------------------
TRINITY_DN2_c0_g2_i5          TAATGAACGACGTGCTAAAGCCTTATTGTATTGTTCTAGCGCGTAACGCTGCTGCGATGA


MpDNV_mRNA_seq_consensus      ATTTAAATTTTGCCAATTGGCCCGCTCCGGTGGAGGAGGACCTAAATAACGAAATTCTCG
TRINITY_DN2_c0_g1_i2          ATTTAAATTTTGCCAATTGGCCCGCTCCGGTGGAGGAGGACCTAAATAACGAAATTCTCG
TRINITY_DN2_c0_g1_i3          ------------------------------------------------------------
TRINITY_DN2_c0_g1_i4          ATTTAAATTTTGCCAATTGGCCCGCTCCGGTGGAGGAGGACCTAAATAACGAAATTCTCG
TRINITY_DN2_c0_g1_i1          ------------------------------------------------------------
TRINITY_DN39_c0_g1_i4         ------------------------------------------------------------
TRINITY_DN39_c0_g1_i3         ------------------------------------------------------------
TRINITY_DN39_c0_g1_i1         ATTTAAATTTTGCCAATTGGCCCGCTCCGGTGGAGGAGGACCTAAATAACGAAATTCTCG
TRINITY_DN39_c0_g1_i2         ATTTAAATTTTGCCAATTGGCCCGCTCCGGTGGAGGAGGACCTAAATAACGAAATTCTCG
TRINITY_DN2_c0_g2_i3          ------------------------------------------------------------
TRINITY_DN2_c0_g2_i2          ATTTAAATTTTGCCAATTGGCCCGCTCCGGTGGAGGAGGACCTAAATAACGAAATTCTCG
TRINITY_DN2_c0_g2_i1          ------------------------------------------------------------
TRINITY_DN2_c0_g2_i5          ATTTAAATTTTGCCAATTGGCCCGCTCCGGTGGAGGAGGACCTAAATAACGAAATTCTCG


MpDNV_mRNA_seq_consensus      CATACTTACGGCTACTGTCTGCTGCCGCTAAATACGGTGTTGTATCAAAATATGTTCCGC 5’ splice site
TRINITY_DN2_c0_g1_i2          CATACTTACGGCTACTGTCTGCTGCCGCTAAATACGGTGTTGTATCAAAATATGTTCCGC unspliced
TRINITY_DN2_c0_g1_i3          ---------GGCTACTGTCTGCTGCCGCTAAATACGGTGTTGTATCAAAATATGTTCCGC spliced intron 1
TRINITY_DN2_c0_g1_i4          CATACTTACGGCTACTGTCTGCTGCCGCTAAATACGGTGTTGTATCAAAATATGTTCCGC unspliced
TRINITY_DN2_c0_g1_i1          ---------GGCTACTGTCTGCTGCCGCTAAATACGGTGTTGTATCAAAATATGTTCCGC spliced intron 1
TRINITY_DN39_c0_g1_i4         ---------GGCTACTGTCTGCTGCCGCTAAATACGGTGTTGTATCAAAATATGTTCCGC spliced intron 1
TRINITY_DN39_c0_g1_i3         ---------GGCTACTGTCTGCTGCCGCTAAATACGGTGTTGTATCAAAATATGTTCCGC spliced intron 1
TRINITY_DN39_c0_g1_i1         CATACTTACGGCTACTGTCTGCTGCCGCTAAATACGGTGTTGTATCAAAATATGTTCCGC unspliced
TRINITY_DN39_c0_g1_i2         CATACTTACGGCTACTGTCTGCTGCCGCTAAATACGGTGTTGTATCAAAATATGTTCCGC unspliced
TRINITY_DN2_c0_g2_i3          ---------GGCTACTGTCTGCTGCCGCTAAATACGGTGTTGTATCAAAATATGTTCCGC spliced intron 1
TRINITY_DN2_c0_g2_i2          CATACTTACGGCTACTGTCTGCTGCCGCTAAATACGGTGTTGTATCAAAATATGTTCCGC unspliced
TRINITY_DN2_c0_g2_i1          ---------GGCTACTGTCTGCTGCCGCTAAATACGGTGTTGTATCAAAATATGTTCCGC spliced intron 1
TRINITY_DN2_c0_g2_i5          CATACTTACGGCTACTGTCTGCTGCCGCTAAATACGGTGTTGTATCAAAATATGTTCCGC unspliced
                                       ***************************************************

MpDNV_mRNA_seq_consensus      TAGTGTTAGGTTTGTTCGGGTATATTGCTGTCTCGCTGCCTGTCAAGAACTGATCGGCTA
TRINITY_DN2_c0_g1_i2          TAGTGTTAGGTTTGTTCGGGTATATTGCTGTCTCGCTGCCTGTCAAGAACTGATCGGCTA
TRINITY_DN2_c0_g1_i3          TAGTGTTAGGTTTGTTCGGGTATATTGCTGTCTCGCTGCCTGTCAAGAACTGATCGGCTA
TRINITY_DN2_c0_g1_i4          TAGTGTTAGGTTTGTTCGGGTATATTGCTGTCTCGCTGCCTGTCAAGAACTGATCGGCTA
TRINITY_DN2_c0_g1_i1          TAGTGTTAGGTTTGTTCGGGTATATTGCTGTCTCGCTGCCTGTCAAGAACTGATCGGCTA
TRINITY_DN39_c0_g1_i4         TAGTGTTAGGTTTGTTCGGGTATATTGCTGTCTCGCTGCCTGTCAAGAACTGATCGGCTA
TRINITY_DN39_c0_g1_i3         TAGTGTTAGGTTTGTTCGGGTATATTGCTGTCTCGCTGCCTGTCAAGAACTGATCGGCTA
TRINITY_DN39_c0_g1_i1         TAGTGTTAGGTTTGTTCGGGTATATTGCTGTCTCGCTGCCTGTCAAGAACTGATCGGCTA
TRINITY_DN39_c0_g1_i2         TAGTGTTAGGTTTGTTCGGGTATATTGCTGTCTCGCTGCCTGTCAAGAACTGATCGGCTA
```

```
TRINITY_DN2_c0_g2_i3         TAGTGTTAGGTTTGTTCGGGTATATTGCTGTCTCGCTGCCTGTCAAGAACTGATCGGCTA
TRINITY_DN2_c0_g2_i2         TAGTGTTAGGTTTGTTCGGGTATATTGCTGTCTCGCTGCCTGTCAAGAACTGATCGGCTA
TRINITY_DN2_c0_g2_i1         TAGTGTTAGGTTTGTTCGGGTATATTGCTGTCTCGCTGCCTGTCAAGAACTGATCGGCTA
TRINITY_DN2_c0_g2_i5         TAGTGTTAGGTTTGTTCGGGTATATTGCTGTCTCGCTGCCTGTCAAGAACTGATCGGCTA
                             ************************************************************

MpDNV_mRNA_seq_consensus     AATCGACACCACGCTTTGCGATCAATCCGAGAGCTGTCGCAATGTTGCCATAATATGGGT
TRINITY_DN2_c0_g1_i2         AATCGACACCACGCTTTGCGATCAATCCGAGAGCTGTCGCAATGTTGCCATAATATGGGT
TRINITY_DN2_c0_g1_i3         AATCGACACCACGCTTTGCGATCAATCCGAGAGCTGTCGCAATGTTGCCATAATATGGGT
TRINITY_DN2_c0_g1_i4         AATCGACACCACGCTTTGCGATCAATCCGAGAGCTGTCGCAATGTTGCCATAATATGGGT
TRINITY_DN2_c0_g1_i1         AATCGACACCACGCTTTGCGATCAATCCGAGAGCTGTCGCAATGTTGCCATAATATGGGT
TRINITY_DN39_c0_g1_i4        AATCGACACCACGCTTTGCGATCAATCCGAGAGCTGTCGCAATGTTGCCATAATATGGGT
TRINITY_DN39_c0_g1_i3        AATCGACACCACGCTTTGCGATCAATCCGAGAGCTGTCGCAATGTTGCCATAATATGGGT
TRINITY_DN39_c0_g1_i1        AATCGACACCACGCTTTGCGATCAATCCGAGAGCTGTCGCAATGTTGCCATAATATGGGT
TRINITY_DN39_c0_g1_i2        AATCGACACCACGCTTTGCGATCAATCCGAGAGCTGTCGCAATGTTGCCATAATATGGGT
TRINITY_DN2_c0_g2_i3         AATCGACACCACGCTTTGCGATCAATCCGAGAGCTGTCGCAATGTTGCCATAATATGGGT
TRINITY_DN2_c0_g2_i2         AATCGACACCACGCTTTGCGATCAATCCGAGAGCTGTCGCAATGTTGCCATAATATGGGT
TRINITY_DN2_c0_g2_i1         AATCGACACCACGCTTTGCGATCAATCCGAGAGCTGTCGCAATGTTGCCATAATATGGGT
TRINITY_DN2_c0_g2_i5         AATCGACACCACGCTTTGCGATCAATCCGAGAGCTGTCGCAATGTTGCCATAATATGGGT
                             ************************************************************

MpDNV_mRNA_seq_consensus     CGCTGACTTGCAGCGCTCCCCGTATCGCTTTTTTGTCTAATTCCCATATGTTATCCTTGC
TRINITY_DN2_c0_g1_i2         CGCTGACTTGCAGCGCTCCCCGTATCGCTTTTTTGTCTAATTCCCATATGTTATCCTTGC
TRINITY_DN2_c0_g1_i3         CGCTGACTTGCAGCGCTCCCCGTATCGCTTTTTTGTCTAATTCCCATATGTTATCCTTGC
TRINITY_DN2_c0_g1_i4         CGCTGACTTGCAGCGCTCCCCGTATCGCTTTTTTGTCTAATTCCCATATGTTATCCTTGC
TRINITY_DN2_c0_g1_i1         CGCTGACTTGCAGCGCTCCCCGTATCGCTTTTTTGTCTAATTCCCATATGTTATCCTTGC
TRINITY_DN39_c0_g1_i4        CGCTGACTTGCAGCGCTCCCCGTATCGCTTTTTTGTCTAATTCCCATATGTTATCCTTGC
TRINITY_DN39_c0_g1_i3        CGCTGACTTGCAGCGCTCCCCGTATCGCTTTTTTGTCTAATTCCCATATGTTATCCTTGC
TRINITY_DN39_c0_g1_i1        CGCTGACTTGCAGCGCTCCCCGTATCGCTTTTTTGTCTAATTCCCATATGTTATCCTTGC
TRINITY_DN39_c0_g1_i2        CGCTGACTTGCAGCGCTCCCCGTATCGCTTTTTTGTCTAATTCCCATATGTTATCCTTGC
TRINITY_DN2_c0_g2_i3         CGCTGACTTGCAGCGCTCCCCGTATCGCTTTTTTGTCTAATTCCCATATGTTATCCTTGC
TRINITY_DN2_c0_g2_i2         CGCTGACTTGCAGCGCTCCCCGTATCGCTTTTTTGTCTAATTCCCATATGTTATCCTTGC
TRINITY_DN2_c0_g2_i1         CGCTGACTTGCAGCGCTCCCCGTATCGCTTTTTTGTCTAATTCCCATATGTTATCCTTGC
TRINITY_DN2_c0_g2_i5         CGCTGACTTGCAGCGCTCCCCGTATCGCTTTTTTGTCTAATTCCCATATGTTATCCTTGC
                             ************************************************************

MpDNV_mRNA_seq_consensus     TAACATCCGTAGCATTAAAATAATCGGCATCGTGTTGAAATGCGACCTTATCTATATCAC
TRINITY_DN2_c0_g1_i2         TAACATCCGTAGCATTAAAATAATCGGCATCGTGTTGGAATGCGACCTTATCTATATCAC
TRINITY_DN2_c0_g1_i3         TAACATCCGTAGCATTAAAATAATCGGCATCGTGTTGGAATGCGACCTTATCTATATCAC
TRINITY_DN2_c0_g1_i4         TAACATCCGTAGCATTAAAATAATCGGCATCGTGTTGGAATGCGACCTTATCTATATCAC
TRINITY_DN2_c0_g1_i1         TAACATCCGTAGCATTAAAATAATCGGCATCGTGTTGGAATGCGACCTTATCTATATCAC
TRINITY_DN39_c0_g1_i4        TAACATCCGTAGCATTAAAATAATCGGCATCGTGTTGAAATGCGACCTTATCTATATCAC
TRINITY_DN39_c0_g1_i3        TAACATCCGTAGCATTAAAATAATCGGCATCGTGTTGAAATGCGACCTTATCTATATCAC
TRINITY_DN39_c0_g1_i1        TAACATCCGTAGCATTAAAATAATCGGCATCGTGTTGAAATGCGACCTTATCTATATCAC
TRINITY_DN39_c0_g1_i2        TAACATCCGTAGCATTAAAATAATCGGCATCGTGTTGAAATGCGACCTTATCTATATCAC
TRINITY_DN2_c0_g2_i3         TAACATCCGTAGCATTAAAATAATCGGCATCGTGTTGGAATGCGACCTTATCTATATCAC
TRINITY_DN2_c0_g2_i2         TAACATCCGTAGCATTAAAATAATCGGCATCGTGTTGGAATGCGACCTTATCTATATCAC
TRINITY_DN2_c0_g2_i1         TAACATCCGTAGCATTAAAATAATCGGCATCGTGTTGGAATGCGACCTTATCTATATCAC
TRINITY_DN2_c0_g2_i5         TAACATCCGTAGCATTAAAATAATCGGCATCGTGTTGGAATGCGACCTTATCTATATCAC
                             ************************************* **********************

MpDNV_mRNA_seq_consensus     TAGTAGGTAACGCCTTAGCATTAAAATGACCTTCACTGTCACACAACAGATTACCTGGAC
TRINITY_DN2_c0_g1_i2         TAGTAGGTAACGCCTTAGCATTAAAATGACCTTCACTGTCACACAACAGATTACCTGGAC
TRINITY_DN2_c0_g1_i3         TAGTAGGTAACGCCTTAGCATTAAAATGACCTTCACTGTCACACAACAGATTACCTGGAC
TRINITY_DN2_c0_g1_i4         TAGTAGGTAACGCCTTAGCATTAAAATGACCTTCACTGTCACACAACAGATTACCTGGAC
TRINITY_DN2_c0_g1_i1         TAGTAGGTAACGCCTTAGCATTAAAATGACCTTCACTGTCACACAACAGATTACCTGGAC
TRINITY_DN39_c0_g1_i4        TAGTAGGTAACGCCTTAGCATTAAAATGACCTTCACTGTCACACAACAGATTACCTGGAC
TRINITY_DN39_c0_g1_i3        TAGTAGGTAACGCCTTAGCATTAAAATGACCTTCACTGTCACACAACAGATTACCTGGAC
TRINITY_DN39_c0_g1_i1        TAGTAGGTAACGCCTTAGCATTAAAATGACCTTCACTGTCACACAACAGATTACCTGGAC
TRINITY_DN39_c0_g1_i2        TAGTAGGTAACGCCTTAGCATTAAAATGACCTTCACTGTCACACAACAGATTACCTGGAC
TRINITY_DN2_c0_g2_i3         TAGTAGGTAACGCCTTAGCATTAAAATGACCTTCACTGTCACACAACAGATTACCTGGAC
TRINITY_DN2_c0_g2_i2         TAGTAGGTAACGCCTTAGCATTAAAATGACCTTCACTGTCACACAACAGATTACCTGGAC
TRINITY_DN2_c0_g2_i1         TAGTAGGTAACGCCTTAGCATTAAAATGACCTTCACTGTCACACAACAGATTACCTGGAC
TRINITY_DN2_c0_g2_i5         TAGTAGGTAACGCCTTAGCATTAAAATGACCTTCACTGTCACACAACAGATTACCTGGAC
                             ************************************************************

MpDNV_mRNA_seq_consensus     CCACGAAGCTACTACCGAACCACGTGATACCTTCATTGCTAGTACCTGAAGCACCTAATT
TRINITY_DN2_c0_g1_i2         CCACGAAGCTACTACCGAACCACGTGATACCTTCATTGCTAGTACCTGAAGCACCTAATT
TRINITY_DN2_c0_g1_i3         CCACGAAGCTACTACCGAACCACGTGATACCTTCATTGCTAGTACCTGAAGCACCTAATT
TRINITY_DN2_c0_g1_i4         CCACGAAGCTACTACCGAACCACGTGATACCTTCATTGCTAGTACCTGAAGCACCTAATT
TRINITY_DN2_c0_g1_i1         CCACGAAGCTACTACCGAACCACGTGATACCTTCATTGCTAGTACCTGAAGCACCTAATT
TRINITY_DN39_c0_g1_i4        CCACGAAGCTACTACCGAACCACGTGATACCTTCATTGCTAGTACCTGAAGCACCTAATT
TRINITY_DN39_c0_g1_i3        CCACGAAGCTACTACCGAACCACGTGATACCTTCATTGCTAGTACCTGAAGCACCTAATT
TRINITY_DN39_c0_g1_i1        CCACGAAGCTACTACCGAACCACGTGATACCTTCATTGCTAGTACCTGAAGCACCTAATT
TRINITY_DN39_c0_g1_i2        CCACGAAGCTACTACCGAACCACGTGATACCTTCATTGCTAGTACCTGAAGCACCTAATT
TRINITY_DN2_c0_g2_i3         CCACGAAGCTACTACCGAACCACGTGATACCTTCATTGCTAGTACCTGAAGCACCTAATT
TRINITY_DN2_c0_g2_i2         CCACGAAGCTACTACCGAACCACGTGATACCTTCATTGCTAGTACCTGAAGCACCTAATT
TRINITY_DN2_c0_g2_i1         CCACGAAGCTACTACCGAACCACGTGATACCTTCATTGCTAGTACCTGAAGCACCTAATT
TRINITY_DN2_c0_g2_i5         CCACGAAGCTACTACCGAACCACGTGATACCTTCATTGCTAGTACCTGAAGCACCTAATT
                             ************************************************************

MpDNV_mRNA_seq_consensus     TAATACGTTTTCTCTCGTTATTCTCCTCTTCAACACTATAAAATTCGTCGTTTACCGACG
TRINITY_DN2_c0_g1_i2         TAATACGTTTTCTCTCGTTATTCTCCTCTTCAACACTATAAAATTCGTCGTTTACCGACG
TRINITY_DN2_c0_g1_i3         TAATACGTTTTCTCTCGTTATTCTCCTCTTCAACACTATAAAATTCGTCGTTTACCGACG
TRINITY_DN2_c0_g1_i4         TAATACGTTTTCTCTCGTTATTCTCCTCTTCAACACTATAAAATTCGTCGTTTACCGACG
TRINITY_DN2_c0_g1_i1         TAATACGTTTTCTCTCGTTATTCTCCTCTTCAACACTATAAAATTCGTCGTTTACCGACG
TRINITY_DN39_c0_g1_i4        TAATACGTTTTCTCTCGTTATTCTCCTCTTCAACACTATAAAATTCGTCGTTTACCGACG
TRINITY_DN39_c0_g1_i3        TAATACGTTTTCTCTCGTTATTCTCCTCTTCAACACTATAAAATTCGTCGTTTACCGACG
```

```
TRINITY_DN39_c0_g1_i1         TAATACGTTTTCTCTCGTTATTCTCCTCTTCAACACTATAAAATTCGTCGTTTACCGACG
TRINITY_DN39_c0_g1_i2         TAATACGTTTTCTCTCGTTATTCTCCTCTTCAACACTATAAAATTCGTCGTTTACCGACG
TRINITY_DN2_c0_g2_i3          TAATACGTTTTCTCTCGTTATTCTCCTCTTCAACACTATAAAATTCGTCGTTTACCGACG
TRINITY_DN2_c0_g2_i2          TAATACGTTTTCTCTCGTTATTCTCCTCTTCAACACTATAAAATTCGTCGTTTACCGACG
TRINITY_DN2_c0_g2_i1          TAATACGTTTTCTCTCGTTATTCTCCTCTTCAACACTATAAAATTCGTCGTTTACCGACG
TRINITY_DN2_c0_g2_i5          TAATACGTTTTCTCTCGTTATTCTCCTCTTCAACACTATAAAATTCGTCGTTTACCGACG
                              ************************************************************

MpDNV_mRNA_seq_consensus      ACATGTTAAACGTAACTGATTAAATATAAGTAAAATGTAAACACTTGAGTAAATAGTGAT
TRINITY_DN2_c0_g1_i2          ACATGTTAAACGTAACTGATTAAATATAAGTAAAATGTAAACACTTGAGTAAATAGTGAT
TRINITY_DN2_c0_g1_i3          ACATGTTAAACGTAACTGATTAAATATAAGTAAAATGTAAACACTTGAGTAAATAGTGAT
TRINITY_DN2_c0_g1_i4          ACATGTTAAACGTAACTGATTAAATATAAGTAAAATGTAAACACTTGAGTAAATAGTGAT
TRINITY_DN2_c0_g1_i1          ACATGTTAAACGTAACTGATTAAATATAAGTAAAATGTAAACACTTGAGTAAATAGTGAT
TRINITY_DN39_c0_g1_i4         ACATGTTAAACGTAACTGATTAAATATAAGTAAAATGTAAACACTTGAGTAAATAGTGAT
TRINITY_DN39_c0_g1_i3         ACATGTTAAACGTAACTGATTAAATATAAGTAAAATGTAAACACTTGAGTAAATAGTGAT
TRINITY_DN39_c0_g1_i1         ACATGTTAAACGTAACTGATTAAATATAAGTAAAATGTAAACACTTGAGTAAATAGTGAT
TRINITY_DN39_c0_g1_i2         ACATGTTAAACGTAACTGATTAAATATAAGTAAAATGTAAACACTTGAGTAAATAGTGAT
TRINITY_DN2_c0_g2_i3          ACATGTTAAACGTAACTGATTAAATATAAGTAAAATGTAAACACTTGAGTAAATAGTGAT
TRINITY_DN2_c0_g2_i2          ACATGTTAAACGTAACTGATTAAATATAAGTAAAATGTAAACACTTGAGTAAATAGTGAT
TRINITY_DN2_c0_g2_i1          ACATGTTAAACGTAACTGATTAAATATAAGTAAAATGTAAACACTTGAGTAAATAGTGAT
TRINITY_DN2_c0_g2_i5          ACATGTTAAACGTAACTGATTAAATATAAGTAAAATGTAAACACTTGAGTAAATAGTGAT
                              ************************************************************

MpDNV_mRNA_seq_consensus      GACGTCACCCCCACCTAGTCGCCTACTGGATACTTGTCTATCTTTTTATTATATAAAAAG 3'ITR TATA box
TRINITY_DN2_c0_g1_i2          GACGTCACCCCCACCTAGTCGCCTACTGGATACTTGTCTATCTTTTTATTATATAAAAAG
TRINITY_DN2_c0_g1_i3          GACGTCACCCCCACCTAGTCGCCTACTGGATACTTGTCTATCTTTTTATTATATAAAAAG
TRINITY_DN2_c0_g1_i4          GACGTCACCCCCACCTAGTCGCCTACTGGATACTTGTCTATCTTTTTATTATATAAAAAG
TRINITY_DN2_c0_g1_i1          GACGTCACCCCCACCTAGTCGCCTACTGGATACTTGTCTATCTTTTTATTATATAAAAAG
TRINITY_DN39_c0_g1_i4         GACGTCACCCCCACCTAGTCGCCCACTG-------------------------------- VP 5'-end
TRINITY_DN39_c0_g1_i3         GACGTCACCCCCACCTAGTCGCCCACTG-------------------------------- VP 5'-end
TRINITY_DN39_c0_g1_i1         GACGTCACCCCCACCTAGTCGCCCACTG-------------------------------- VP 5'-end
TRINITY_DN39_c0_g1_i2         GACGTCACCCCCACCTAGTCGCCCACTG-------------------------------- VP 5'-end
TRINITY_DN2_c0_g2_i3          GACGTCACCCCCACCTAGTCGCCTACTGGATACTTGTCTATCTTTTTATTATATAAAAAG
TRINITY_DN2_c0_g2_i2          GACGTCACCCCCACCTAGTCGCCTACTGGATACTTGTCTATCTTTTTATTATATAAAAAG
TRINITY_DN2_c0_g2_i1          GACGTCACCCCCACCTAGTCGCCTACTGGATACTTGTCTATCTTTTTATTATATAAAAAG
TRINITY_DN2_c0_g2_i5          GACGTCACCCCCACCTAGTCGCCTACTGGATACTTGTCTATCTTTTTATTATATAAAAAG
                              *********************** ****

MpDNV_mRNA_seq_consensus      ATCCCCTACTAATAATAACCACTACCTACTTCCGGGGGGCGGGGGGTATCCTTTGTTATC
TRINITY_DN2_c0_g1_i2          ATCCCCCACTAATAATAACCACTACCTACTTCCGGGGGGCGGGGGGTATCCTTTGTTATC
TRINITY_DN2_c0_g1_i3          ATCCCCCACTAATAATAACCACTACCTACTTCCGGGGGGCGGGGGGTATCCTTTGTTATC
TRINITY_DN2_c0_g1_i4          ATCCCCCACTAATAATAACCACTACCTACTTCCGGGGGGCGGGGGGTATCCTTTGTTATC
TRINITY_DN2_c0_g1_i1          ATCCCCCACTAATAATAACCACTACCTACTTCCGGGGGGCGGGGGGTATCCTTTGTTATC
TRINITY_DN39_c0_g1_i4         ------------------------------------------------------------
TRINITY_DN39_c0_g1_i3         ------------------------------------------------------------
TRINITY_DN39_c0_g1_i1         ------------------------------------------------------------
TRINITY_DN39_c0_g1_i2         ------------------------------------------------------------
TRINITY_DN2_c0_g2_i3          ATCCCCTACTAATAATAACCACTACCTACTTCCGGGGGGCGGGGGGTATCCTTTGTTATC
TRINITY_DN2_c0_g2_i2          ATCCCCTACTAATAATAACCACTACCTACTTCCGGGGGGCGGGGGGTATCCTTTGTTATC
TRINITY_DN2_c0_g2_i1          ATCCCCTACTAATAATAACCACTACCTACTTCCGGGGGGCGGGGGGTATCCTTTGTTATC
TRINITY_DN2_c0_g2_i5          ATCCCCTACTAATAATAACCACTACCTACTTCCGGGGGGCGGGGGGTATCCTTTGTTATC

MpDNV_mRNA_seq_consensus      TATACTTATTGTCAACACTAGCCTTATCTTAGGGCGTGTCGGCCGACACGTGTCATGGCC
TRINITY_DN2_c0_g1_i2          TATACTTATTGTCAACACTAGCCTTATAC------------------------------- VP 5'-end
TRINITY_DN2_c0_g1_i3          TATACTTATTGTCAACACTAGCCTTATAC------------------------------- VP 5'-end
TRINITY_DN2_c0_g1_i4          TATACTTATTGTCAACACTAGCCTTATAC------------------------------- VP 5'-end
TRINITY_DN2_c0_g1_i1          TATACTTATTGTCAACACTAGCCTTATAC------------------------------- VP 5'-end
TRINITY_DN39_c0_g1_i4         ------------------------------------------------------------
TRINITY_DN39_c0_g1_i3         ------------------------------------------------------------
TRINITY_DN39_c0_g1_i1         ------------------------------------------------------------
TRINITY_DN39_c0_g1_i2         ------------------------------------------------------------
TRINITY_DN2_c0_g2_i3          TATACTTATTGTCAACACTAGCCTTATCTTAGGGCT------------AGTGTCATGGCC
TRINITY_DN2_c0_g2_i2          TATACTTATTGTCAACACTAGCCTTATCTTAGGGCT------------AGTGTCATGGCC
TRINITY_DN2_c0_g2_i1          TATACTTATTGTCAACACTAGCCTTATCTTAGGGCT------------AGTGTCATGGCC
TRINITY_DN2_c0_g2_i5          TATACTTATTGTCAACACTAGCCTTATCTTAGGGCT------------AGTGTCATGGCC

MpDNV_mRNA_seq_consensus      ATGACACTAGCCCTA---------------------------------------------
TRINITY_DN2_c0_g1_i2          ------------------------------------------------------------
TRINITY_DN2_c0_g1_i3          ------------------------------------------------------------
TRINITY_DN2_c0_g1_i4          ------------------------------------------------------------
TRINITY_DN2_c0_g1_i1          ------------------------------------------------------------
TRINITY_DN39_c0_g1_i4         ------------------------------------------------------------
TRINITY_DN39_c0_g1_i3         ------------------------------------------------------------
TRINITY_DN39_c0_g1_i1         ------------------------------------------------------------
TRINITY_DN39_c0_g1_i2         ------------------------------------------------------------
TRINITY_DN2_c0_g2_i3          ATGACACGGACATTCTGTATTTGATCCTTCCTGTTTGTAAATTTTAATGCATTCGATTAG VP 5'-end
TRINITY_DN2_c0_g2_i2          ATGACACGGACATTCTGTATTTGATCCTTCCTGTTTGTAAATTTTAATGCATTCGATTAG VP 5'-end
TRINITY_DN2_c0_g2_i1          ATGACACGGACATTCTGTATTTGATCCTTCCTGTTTGTAAATTTTAATGCATTCGATTAG VP 5'-end
TRINITY_DN2_c0_g2_i5          ATGACACGGACATTCTGTATTTGATCCTTCCTGTTTGTAAATTTTAATGCATTCGATTAG
```

APFV-8-9-10 (Artificial diet without virus)

```
MpDNV_mRNA_seq_consensus      CTTGTTATCTATACTTATTGTCAACACTAGCCTTATCTTAGGGCGTGTCGGCCGACACGT
TRINITY_DN8906_c0_g1_i1 APFV-8------------------------------------------------------------
TRINITY_DN8906_c0_g1_i2 APFV-8------------------------------------------------------------
```

```
TRINITY_DN7_c0_g3_i1    APFV-9-------------CTTGGAGACAACACTAGCCTTATCTTAGGGCGTGTCGGCCGACACGT NS 5’-end
TRINITY_DN10_c0_g1_i2  APFV-10-------------------------------------------CGTGTCGGCCGACAC-- NS 5’-end
TRINITY_DN10_c0_g1_i1  APFV-10-------------------------------------------CGTGTCGGCCGACAC-- NS 5’-end


MpDNV_mRNA_seq_consensus      GTCATGGCCATGACACTAGCCCTAAGATAAGGCTAGTGTTGACAATAAGTATAGATAAC-
TRINITY_DN8906_c0_g1_i1       ------------------------------------------------------------
TRINITY_DN8906_c0_g1_i2       ------------------------------------------------------------
TRINITY_DN7_c0_g3_i1          GTCATGGCCATGACACTAGCCCTAAGATAAGGCTAGTGTTGACAATAAGTATAGATAACA
TRINITY_DN10_c0_g1_i2         ------------------GCCCTAAGATAAGGCTAGTGTTGACAATAAGTATAGATAACA
TRINITY_DN10_c0_g1_i1         ------------------GCCCTAAGATAAGGCTAGTGTTGACAATAAGTATAGATAACA


MpDNV_mRNA_seq_consensus      AAGGATACCCCCCGCCCCCCGGAAGTAGGTAGTGGTTATTATTAGTAGGGGATCTTTTTA 5’ITR TATA-box
TRINITY_DN8906_c0_g1_i1       ------------------------------------------------------------
TRINITY_DN8906_c0_g1_i2       ------------------------------------------------------------
TRINITY_DN7_c0_g3_i1          AAGGATACCCCCCGCCCCCCGGAAGTAGGTAGTGGTTATTATTAGTAGGGGATCTTTTTA
TRINITY_DN10_c0_g1_i2         AAGGATACCCCCCGCCCCCCGGAAGTAGGTAGTGGTTATTATTAGTGGGGGATCTTTTTA
TRINITY_DN10_c0_g1_i1         AAGGATACCCCCCGCCCCCCGGAAGTAGGTAGTGGTTATTATTAGTGGGGGATCTTTTTA


MpDNV_mRNA_seq_consensus      TATAATAAAAAGATAGACAAGTATCCAGTAGGCGACTAGGTGGGGGTGACGTCATCACTA
TRINITY_DN8906_c0_g1_i1       ------------------------------------------------------------
TRINITY_DN8906_c0_g1_i2       ------------------------------------------------------------
TRINITY_DN7_c0_g3_i1          TATAATAAAAAGATAGACAAGTATCCAGTAGGCGACTAGGTGGGGGTGACGTCATCACTA
TRINITY_DN10_c0_g1_i2         TATAATAAAAAGATAGACAAGTATCCAGTAGGCGACTAGGTGGGGGTGACGTCATCACTA
TRINITY_DN10_c0_g1_i1         TATAATAAAAAGATAGACAAGTATCCAGTAGGCGACTAGGTGGGGGTGACGTCATCACTA


MpDNV_mRNA_seq_consensus      TTTACTCAAGTGTTTTGTTATTCGGCGTCAAGTGTTGTGTTGACACTTGAAAAGTGGCGT
TRINITY_DN8906_c0_g1_i1       ------------TTTTGTTATTCGGCGTCAAGTGTTGTGTTGACACTTGAAAAGTGGCGT NS 5’-end
TRINITY_DN8906_c0_g1_i2       ------------TTTTGTTATTCGGCGTCAAGTGTTGTGTTGACACTTGAAAAGTGGCGT NS 5’-end
TRINITY_DN7_c0_g3_i1          TTTACTCAAGTGTTTTGTTATTCGGCGTCAAGTGTTGTGTTGACACTTGAAAAGTGGCGT
TRINITY_DN10_c0_g1_i2         TTTACTCAAGTGTTTTGTTATTCGGCGTCAAGTGTTGTGTTGACACTTGAAAAGTGGCGT
TRINITY_DN10_c0_g1_i1         TTTACTCAAGTGTTTTGTTATTCGGCGTCAAGTGTTGTGTTGACACTTGAAAAGTGGCGT
                                          ************************************************

MpDNV_mRNA_seq_consensus      GATTGATTATTTACAAGTACGTCAGATAAGATAAACATAAGTGGTACTTGACTCGGAATA
TRINITY_DN8906_c0_g1_i1       GATTGATTATTTACAAGTACGTCAGATAAGATAAACATAAGTGGTACTTGACTCGGAATA
TRINITY_DN8906_c0_g1_i2       GATTGATTATTTACAAGTACGTCAGATAAGATAAACATAAGTGGTACTTGACTCGGAATA
TRINITY_DN7_c0_g3_i1   APFV-9 GATTGATTATCTACAAGTACGTCAGATAAGATAAACATAAGTGGTACTTGACTCGGAATA
TRINITY_DN10_c0_g1_i2         GATTGATTATTTACAAGTACGTCAGATAAGATAAACATAAGTGGTACTTGACTCGGAATA
TRINITY_DN10_c0_g1_i1         GATTGATTATTTACAAGTACGTCAGATAAGATAAACATAAGTGGTACTTGACTCGGAATA
                              ********** *************************************************

MpDNV_mRNA_seq_consensus      TATAATGAAGTGTGGAACCTACAACAATATCAGTACTCATCAAAATGTCAAACTCGCAAG
TRINITY_DN8906_c0_g1_i1APFV-8 TATAATGAAGTGTGGAACCTACAACAATGTCAGTACTCATCAAAATGTCAAACTCGCAAG
TRINITY_DN8906_c0_g1_i2APFV-8 TATAATGAAGTGTGGAACCTACAACAATGTCAGTACTCATCAAAATGTCAAACTCGCAAG
TRINITY_DN7_c0_g3_i1          TATAATGAAGTGTGGAACCTACAACAATATCAGTACTCATCAAAATGTCAAACTCGCAAG
TRINITY_DN10_c0_g1_i2         TATAATGAAGTGTGGAACCTACAACAATATCAGTACTCATCAAAATGTCAAACTCGCAAG
TRINITY_DN10_c0_g1_i1         TATAATGAAGTGTGGAACCTACAACAATATCAGTACTCATCAAAATGTCAAACTCGCAAG
                              **************************** *******************************

MpDNV_mRNA_seq_consensus      AAGTGAACCAACAACAATTCAACGACGAGGAGGAAATACCATGTGGCCAACGGTATCCTA
TRINITY_DN8906_c0_g1_i1       AAGTGAACCAACAACAATTCAACGACGAGGAGGAAATACCATGTGGCCAACGGTATCCTA
TRINITY_DN8906_c0_g1_i2       AAGTGAACCAACAACAATTCAACGACGAGGAGGAAATACCATGTGGCCAACGGTATCCTA
TRINITY_DN7_c0_g3_i1          AAGTGAACCAACAACAATTCAACGACGAGGAGGAAATACCATGTGGCCAACGGTATCCTA
TRINITY_DN10_c0_g1_i2         AAGTGAACCAACAACAATTCAACGACGAGGAGGAAATACCATGTGGCCAACGGTATCCTA
TRINITY_DN10_c0_g1_i1         AAGTGAACCAACAACAATTCAACGACGAGGAGGAAATACCATGTGGCCAACGGTATCCTA
                              ************************************************************

MpDNV_mRNA_seq_consensus      CAGCGGCGACGATTCAAACGGACCACCAACAGACTGGCGTGAATGGATCGACCCCGATGT
TRINITY_DN8906_c0_g1_i1       CAGCGGCGACGATTCAAACGGACCACCAACAGAGTGGCGTGAATGGATCGACCCCGATGT
TRINITY_DN8906_c0_g1_i2       CAGCGGCGACGATTCAAACGGACCACCAACAGAGTGGCGTGAATGGATCGACCCCGATGT
TRINITY_DN7_c0_g3_i1          CAGCGGCGACGATTCAAACGGACCACCAACAGAGTGGCGTGAATGGATCGACCCCGATGT
TRINITY_DN10_c0_g1_i2         CAGCGGCGACGATTCAAACGGACCACCAACAGAGTGGCGTGAATGGATCGACCCCGATGT
TRINITY_DN10_c0_g1_i1         CAGCGGCGACGATTCAAACGGACCACCAACAGAGTGGCGTGAATGGATCGACCCCGATGT
                              ********************************* **************************

MpDNV_mRNA_seq_consensus      TCGAACTCAAATCGACAGCGCGCAAAGAATATGGGACAGCCATTTCAGCGGACACGGCAC
TRINITY_DN8906_c0_g1_i1       TCGAACTCAAATCGACAGCGCGCAAAGAATATGGGACAGCCATTTCAGCGGACACGGCAC
TRINITY_DN8906_c0_g1_i2       TCGAACTCAAATCGACAGCGCGCAAAGAATATGGGACAGCCATTTCAGCGGACACGGCAC
TRINITY_DN7_c0_g3_i1          TCGAACTCAAATCGACAGCGCGCAAAGAATATGGGACAGCCATTTCAGCGGACACGGCAC
TRINITY_DN10_c0_g1_i2         TCGAACTCAAATCGACAGCGCGCAAAGAATATGGGACAGCCATTTCAGCGGACACGGCAC
TRINITY_DN10_c0_g1_i1         TCGAACTCAAATCGACAGCGCGCAAAGAATATGGGACAGCCATTTCAGCGGACACGGCAC
                              ************************************************************

MpDNV_mRNA_seq_consensus      TGACACTGAAGACGCTGTGCAAAGTGTTGGAGTGCAAGGCAACGGAACTAACGGTAACAT
TRINITY_DN8906_c0_g1_i1       TGACACTGAAGACGCTGTGCAAAGTGTTGGAGTGCAAGGCAACGGAACTAACGGTAACAT
TRINITY_DN8906_c0_g1_i2       TGACACTGAAGACGCTGTGCAAAGTGTTGGAGTGCAAGGCAACGGAACTAACGGTAACAT
TRINITY_DN7_c0_g3_i1          TGACACTGAAGACGCTGTGCAAAGTGTTGGAGTGCAAGGCAACGGAACTAACGGTAACAT
TRINITY_DN10_c0_g1_i2         TGACACTGAAGACGCTGTGCAAAGTGTTGGAGTGCAAGGCAACGGAACTAACGGTAACAT
TRINITY_DN10_c0_g1_i1         TGACACTGAAGACGCTGTGCAAAGTGTTGGAGTGCAAGGCAACGGAACTAACGGTAACAT
                              ************************************************************

MpDNV_mRNA_seq_consensus      GCATTATTCCTCTGATCGCGTTGCTATTACCCGACCATATGTGGTCAGAGGCGACGAGCA
TRINITY_DN8906_c0_g1_i1       GCATTATTCCTCTGATCGCGTTGCTATTACCCGACCATATGTGGTCAGAGGCGACGAGCA
TRINITY_DN8906_c0_g1_i2       GCATTATTCCTCTGATCGCGTTGCTATTACCCGACCATATGTGGTCAGAGGCGACGAGCA
```

```
TRINITY_DN7_c0_g3_i1         GCATTATTCCTCTGATCGCGTTGCTATTACCCGACCATATGTGGTCAGAGGCGACGAGCA
TRINITY_DN10_c0_g1_i2        GCATTATTCCTCTGATCGCGTTGCTATTACCCGACCATATGTGGTCAGAGGCGACGAGCA
TRINITY_DN10_c0_g1_i1        GCATTATTCCTCTGATCGCGTTGCTATTACCCGACCATATGTGGTCAGAGGCGACGAGCA
                             ************************************************************

MpDNV_mRNA_seq_consensus     GCGATCGCTGGCGATACCGACTGAGAAACGCGACCTACTGGATTTCCAATCCAACGCGGG
TRINITY_DN8906_c0_g1_i1      GCGATCGCTGGCGATACCGACTGAGAAACGCGACCTACTGGATTTCCAATCCAACGCGGG
TRINITY_DN8906_c0_g1_i2      GCGATCGCTGGCGATACCGACTGAGAAACGCGACCTACTGGATTTCCAATCCAACGCGGG
TRINITY_DN7_c0_g3_i1         GCGATCGCTGGCGATACCGACTGAGAAACGCGACCTACTGGATTTCCAATCCAACGCGGG
TRINITY_DN10_c0_g1_i2        GCGATCGCTGGCGATACCGACTGAGAAACGCGACCTACTGGATTTCCAATCCAACGCGGG
TRINITY_DN10_c0_g1_i1        GCGATCGCTGGCGATACCGACTGAGAAACGCGACCTACTGGATTTCCAATCCAACGCGGG
                             ************************************************************

MpDNV_mRNA_seq_consensus     AATCCGAATATCAGGCTTTACTACGATGGATGGAGGAGAAAATGCCGATACCTTTAGCGA
TRINITY_DN8906_c0_g1_i1      AATCCGAATATCAGGCTTTACTACGATGGATGGAGGAGAAAATGCCGATACCTTTAGCGA
TRINITY_DN8906_c0_g1_i2      AATCCGAATATCAGGCTTTACTACGATGGATGGAGGAGAAAATGCCGATACCTTTAGCGA
TRINITY_DN7_c0_g3_i1         AATCCGAATATCAGGCTTTACTACGATGGATGGAGGAGAAAATGCCGATACCTTTAGCGA
TRINITY_DN10_c0_g1_i2        AATCCGAATATCAGGCTTTACTACGATGGATGGAGGAGAAAATGCCGATACCTTTAGCGA
TRINITY_DN10_c0_g1_i1        AATCCGAATATCAGGCTTTACTACGATGGATGGAGGAGAAAATGCCGATACCTTTAGCGA
                             ************************************************************

MpDNV_mRNA_seq_consensus     AATGGCAAGGTGCATGTCTACCGCGGTATGGGGAGGCAATGTCAGATATGTTTCTGACAT
TRINITY_DN8906_c0_g1_i1      AATGGCAAGGTGCATGTCTACCGCGGTATGGGGAGGCAATGTCAGATATGTTTCTGACAT
TRINITY_DN8906_c0_g1_i2      AATGGCAAGGTGCATGTCTACCGCGGTATGGGGAGGCAATGTCAGATATGTTTCTGACAT
TRINITY_DN7_c0_g3_i1         AATGGCAAGGTGCATGTCTACCGCGGTATGGGGAGGCAATGTCAGATATGTTTCTGACAT
TRINITY_DN10_c0_g1_i2        AATGGCAAGGTGCATGTCTACCGCGGTATGGGGAGGCAATGTCAGATATGTTTCTGACAT
TRINITY_DN10_c0_g1_i1        AATGGCAAGGTGCATGTCTACCGCGGTATGGGGAGGCAATGTCAGATATGTTTCTGACAT
                             ************************************************************

MpDNV_mRNA_seq_consensus     CCTTGTACCCGGAGGAAATTGGTCAATTAATGGAATTGTTGAGGACCTACGCCGGAGTAT
TRINITY_DN8906_c0_g1_i1      CCTTGTACCCGGAGGAAATTGGTCAATTAATGGAATTGTTGAGGACCTACGCCGGAGTAT
TRINITY_DN8906_c0_g1_i2      CCTTGTACCCGGAGGAAATTGGTCAATTAATGGAATTGTTGAGGACCTACGCCGGAGTAT
TRINITY_DN7_c0_g3_i1         CCTTGTACCCGGAGGAAATTGGTCAATTAATGGAATTGTTGAGGACCTACGCCGGAGTAT
TRINITY_DN10_c0_g1_i2        CCTTGTACCCGGAGGAAATTGGTCAATTAATGGAATTGTTGAGGACCTACGCCGGAGTAT
TRINITY_DN10_c0_g1_i1        CCTTGTACCCGGAGGAAATTGGTCAATTAATGGAATTGTTGAGGACCTACGCCGGAGTAT
                             ************************************************************

MpDNV_mRNA_seq_consensus     CGCAGAGCGAATGCCAAAATGCATGTACGTCGTCAGCGAACACGGCGACCACGTCCACGT
TRINITY_DN8906_c0_g1_i1      CGCAGAGCGAATGCCAAAATGCATGTACGTCGTCAGCGAACACGGCGACCACGTCCACGT
TRINITY_DN8906_c0_g1_i2      CGCAGAGCGAATGCCAAAATGCATGTACGTCGTCAGCGAACACGGCGACCACGTCCACGT
TRINITY_DN7_c0_g3_i1         CGCAGAGCGAATGCCAAAATGCATGTACGTCGTCAGCGAACACGGCGACCACGTCCACGT
TRINITY_DN10_c0_g1_i2        CGCAGAGCGAATGCCAAAATGCATGTACGTCGTCAGCGAACACGGCGACCACGTCCACGT
TRINITY_DN10_c0_g1_i1        CGCAGAGCGAATGCCAAAATGCATGTACGTCGTCAGCGAACACGGCGACCACGTCCACGT
                             ************************************************************

MpDNV_mRNA_seq_consensus     CGTACACACCTGTAACTACGCCACGGGCAGCTGTAGATGTAGTTTCTTACTCAACGCCGC
TRINITY_DN8906_c0_g1_i1      CGTACACACCTGTAACTACGCCACGGGCAGCTGTAGATGTAGTTTCTTACTCAACGCCGC
TRINITY_DN8906_c0_g1_i2      CGTACACACCTGTAACTACGCCACGGGCAGCTGTAGATGTAGTTTCTTACTCAACGCCGC
TRINITY_DN7_c0_g3_i1         CGTACACACCTGTAACTACGCCACGGGCAGCTGTAGATGTAGTTTCTTACTCAACGCCGC
TRINITY_DN10_c0_g1_i2        CGTACACACCTGTAACTACGCCACGGGCAGCTGTAGATGTAGTTTCTTACTCAACGCCGC
TRINITY_DN10_c0_g1_i1        CGTACACACCTGTAACTACGCCACGGGCAGCTGTAGATGTAGTTTCTTACTCAACGCCGC
                             ************************************************************

MpDNV_mRNA_seq_consensus     TGCTTTCCAACTATGTAGACGACGACGCATTCGAAAGAATGTTAGAGTCATCCAACTATC
TRINITY_DN8906_c0_g1_i1      TGCTTTCCAACTATGTAGACGACGACGCATTCGAAAGAATGTTAGAGTCATCCAACTATC
TRINITY_DN8906_c0_g1_i2      TGCTTTCCAACTATGTAGACGACGACGCATTCGAAAGAATGTTAGAGTCATCCAACTATC
TRINITY_DN7_c0_g3_i1         TGCTTTCCAACTATGTAGACGACGACGCATTCGAAAGAATGTTAGAGTCATCCAACTATC
TRINITY_DN10_c0_g1_i2        TGCTTTCCAACTATGTAGACGACGACGCATTCGAAAGAATGTTAGAGTCATCCAACTATC
TRINITY_DN10_c0_g1_i1        TGCTTTCCAACTATGTAGACGACGACGCATTCGAAAGAATGTTAGAGTCATCCAACTATC
                             ************************************************************

MpDNV_mRNA_seq_consensus     AGAAAGAGACTGGTCCCGTATCTTTCAATACTTATGTTCATCGCCCAGGAATGTCAAAGA
TRINITY_DN8906_c0_g1_i1      AGAAAGAGACTGGTCCCGTATCTTTCAATACTTATGTTCATCGCCCAGGAATGTCAAAGA
TRINITY_DN8906_c0_g1_i2      AGAAAGAGACTGGTCCCGTATCTTTCAATACTTATGTTCATCGCCCAGGAATGTCAAAGA
TRINITY_DN7_c0_g3_i1         AGAAAGAGACTGGTCCCGTATCTTTCAATACTTATGTTCATCGCCCAGGAATGTCAAAGA
TRINITY_DN10_c0_g1_i2        AGAAAGAGACTGGTCCCGTATCTTTCAATACTTATGTTCATCGCCCAGGAATGTCAAAGA
TRINITY_DN10_c0_g1_i1        AGAAAGAGACTGGTCCCGTATCTTTCAATACTTATGTTCATCGCCCAGGAATGTCAAAGA
                             ************************************************************

MpDNV_mRNA_seq_consensus     AGTGGGCGGCCAGTACTTTGATGGAGGATTACGTAGTAGATATTCACATATATCAGTTAG 5' splice site
TRINITY_DN8906_c0_g1_i1      AGTGGGCGGCCAGTACTTTGATGGAGGATTACGTAGTAGATATTCACATATATCAGTTAG NS unspliced
TRINITY_DN8906_c0_g1_i2      AGTGGGCGGCCAGTACTTTGATGGAGGATTACGTAGTAGATATTCACATATATCA----- spliced intron
TRINITY_DN7_c0_g3_i1         AGTGGGCGGCCAGTACTTTGATGGAGGATTACGTAGTAGATATTCACATATATCAGTTAG NS unspliced
TRINITY_DN10_c0_g1_i2        AGTGGGCGGCCAGTACTTTGATGGAGGATTACGTAGTAGATATTCACATATATCAGTTAG NS unspliced
TRINITY_DN10_c0_g1_i1        AGTGGGCGGCCAGTACTTTGATGGAGGATTACGTAGTAGATATTCACATATATCA----- spliced intron
                             *******************************************************

MpDNV_mRNA_seq_consensus     TATTGCGTAATTTTTTTATATACTTGGATTGGTTGACGGGTATAGAAGCGAATAGATGCG
TRINITY_DN8906_c0_g1_i1      TATTGCGTAATTTTTTTATATACTTGGATTGGTTGACGGGTATAGAAGCGAATAGATGCG
TRINITY_DN8906_c0_g1_i2      ------------------------------------------------------------
TRINITY_DN7_c0_g3_i1         TATTGCGTAATTTTTTTATATACTTGGATTGGTTGACGGGTATAGAAGCGAATAGATGCG
TRINITY_DN10_c0_g1_i2        TATTGCGTAATTTTTTTATATACTTGGATTGGTTGACGGGTATAGAAGCGAATAGATGCG
TRINITY_DN10_c0_g1_i1        ------------------------------------------------------------

MpDNV_mRNA_seq_consensus     TTTCATATATTAAAACTGGTATGCCTGGCAGGAGTTACGTTAAAAGCGTGATGCCAAAAA
TRINITY_DN8906_c0_g1_i1      TTTCATATATTAAAACTGGTATGCCTGGCAGGAGTTACGTTAAAAGCGTGATGCCAAAAA
TRINITY_DN8906_c0_g1_i2      ------------------------------------------------------------
```

```
TRINITY_DN7_c0_g3_i1         TTTCATATATTAAAACTGGTATGCCTGGCAGGAGTTACGTTAAAAGCGTGATGCCAAAAA
TRINITY_DN10_c0_g1_i2        TTTCATATATTAAAACTGGTATGCCTGGCAGGAGTTACGTTAAAAGCGTGATGCCAAAAA
TRINITY_DN10_c0_g1_i1        ------------------------------------------------------------

MpDNV_mRNA_seq_consensus     ATTATTGGCGTAATCCTAAAGAATTTCACTTAGCGCTTGATAAATTAGAGTTGCTCGTTA
TRINITY_DN8906_c0_g1_i1      ATTATTGGCGTAATCCTAAAGAATTTCACTTAGCGCTTGATAAATTAGAGTTGCTCGTTA
TRINITY_DN8906_c0_g1_i2      ------------------------------------------------------------
TRINITY_DN7_c0_g3_i1         ATTATTGGCGTAATCCTAAAGAATTTCACTTAGCGCTTGATAAATTAGAGTTGCTCGTTA
TRINITY_DN10_c0_g1_i2        ATTATTGGCGTAATCCTAAAGAATTTCACTTAGCGCTTGATAAATTAGAGTTGCTCGTTA
TRINITY_DN10_c0_g1_i1        ------------------------------------------------------------

MpDNV_mRNA_seq_consensus     AACGACATTTCGGTGATTTCACGTGTTTGGAAAATTTTTTACCGCAATATCGTTGGCATA
TRINITY_DN8906_c0_g1_i1      AACGACATTTCGGTGATTTCACGTGTTTGGAAAATTTTTTACCGCAATATCGTTGGCATA
TRINITY_DN8906_c0_g1_i2      ------------------------------------------------------------
TRINITY_DN7_c0_g3_i1         AACGACATTTCGGTGATTTCACGTGTTTGGAAAATTTTTTACCGCAATATCGTTGGCATA
TRINITY_DN10_c0_g1_i2        AACGACATTTCGGTGATTTCACGTGTTTGGAAAATTTTTTACCGCAATATCGTTGGCATA
TRINITY_DN10_c0_g1_i1        ------------------------------------------------------------

MpDNV_mRNA_seq_consensus     ATTGCATCACATGGTTTGTGAACGTGATGTCCTGTCCGTTGGACCCGTGGACTGTCGAAT
TRINITY_DN8906_c0_g1_i1      ATTGCATCACATGGTTTGTGAACGTGATGTCCTGTCCGTTGGACCCGTGGACTGTCGAAT
TRINITY_DN8906_c0_g1_i2      ------------------------------------------------------------
TRINITY_DN7_c0_g3_i1         ATTGCATCACATGGTTTGTGAACGTGATGTCCTGTCCGTTGGACCCGTGGACTGTCGAAT
TRINITY_DN10_c0_g1_i2        ATTGCATCACATGGTTTGTGAACGTGATGTCCTGTCCGTTGGACCCGTGGACTGTCGAAT
TRINITY_DN10_c0_g1_i1        ------------------------------------------------------------

MpDNV_mRNA_seq_consensus     GTGCTGGTTGTGGTTTTCAACATATTCCTAATATATCTAAATGCGAACTACTACGAGACT
TRINITY_DN8906_c0_g1_i1      GTGCTGGTTGTGGTTTTCAACATATTCCTAATATATCTAAATGCGAACTACTACGAGACT
TRINITY_DN8906_c0_g1_i2      ------------------------------------------------------------
TRINITY_DN7_c0_g3_i1         GTGCTGGTTGTGGTTTTCAACATATTCCTAATATATCTAAATGCGAACTACTACGAGACT
TRINITY_DN10_c0_g1_i2        GTGCTGGTTGTGGTTTTCAACATATTCCTAATATATCTAAATGCGAACTACTACGAGACT
TRINITY_DN10_c0_g1_i1        ------------------------------------------------------------

MpDNV_mRNA_seq_consensus     ATCCGTTCATGAACGAGGGCACTATAAATCATATACTAATCGAATGCATTAAAATTTACA
TRINITY_DN8906_c0_g1_i1      ATCCGTTCATGAACGAGGGCACTATAAATCATATACTAATCGAATGCATTAAAATTTACA
TRINITY_DN8906_c0_g1_i2      ------------------------------------------------------------
TRINITY_DN7_c0_g3_i1         ATCCGTTCATGAACGAGGGCACTATAAATCATATACTAATCGAATGCATTAAAATTTACA
TRINITY_DN10_c0_g1_i2        ATCCGTTCATGAACGAGGGCACTATAAATCATATACTAATCGAATGCATTAAAATTTACA
TRINITY_DN10_c0_g1_i1        ------------------------------------------------------------

MpDNV_mRNA_seq_consensus     AACAGGAAGGATCAAATACAGAATGTCGACCCGAACGATTGGTGGAAACATGCCTTCTGC 3' splice site
TRINITY_DN8906_c0_g1_i1      AACAGGAAGGATCAAATACAGAATGTCGACCCGAACGATTGGTGGAAACATGCCTTCTGC NS unspliced
TRINITY_DN8906_c0_g1_i2      -----GAAGGATCAAATACAGAATGTCGACCCGAACGATTGGTGGAAACATGCCTTCTGC spliced intron
TRINITY_DN7_c0_g3_i1         AACAGGAAGGATCAAATACAGAATGTCGACCCGAACGATTGGTGGAAACATGCCTTCTGC NS unspliced
TRINITY_DN10_c0_g1_i2        AACAGGAAGGATCAAATACAGAATGTCGACCCGAACGATTGGTGGAAACATGCCTTCTGC NS unspliced
TRINITY_DN10_c0_g1_i1        -----GAAGGATCAAATACAGAATGTCGACCCGAACGATTGGTGGAAACATGCCTTCTGC spliced intron
                                  *******************************************************

MpDNV_mRNA_seq_consensus     AGGGCTCGGGGGAGCTTCAATCTGAACAGTCCATACGTGAAAATGGCCAAAACCATACAA
TRINITY_DN8906_c0_g1_i1      AGGGCTCGGGGGAGCTTCAATCTGAACAGTCCATACGTGAAAATGGCCAAAACCATACAA
TRINITY_DN8906_c0_g1_i2      AGGGCTCGGGGGAGCTTCAATCTGAACAGTCCATACGTGAAAATGGCCAAAACCATACAA
TRINITY_DN7_c0_g3_i1         AGGGCTCGGGGGAGCTTCAATCTGAACAGTCCATACGTGAAAATGGCCAAAACCATACAA
TRINITY_DN10_c0_g1_i2        AGGGCTCGGGGGAGCTTCAATCTGAACAGTCCATACGTGAAAATGGCCAAAACCATACAA
TRINITY_DN10_c0_g1_i1        AGGGCTCGGGGGAGCTTCAATCTGAACAGTCCATACGTGAAAATGGCCAAAACCATACAA
                             ************************************************************

MpDNV_mRNA_seq_consensus     CAGGAAGTGTTTCGCAGGATGACCAAGATGGAGTACAAGATAACGGATCCACAAATGTTT
TRINITY_DN8906_c0_g1_i1      CAGGAAGTGTTTCGCAGGATGACCAAGATGGAGTACAAGATAACGGATCCACAAATGTTT
TRINITY_DN8906_c0_g1_i2      CAGGAAGTGTTTCGCAGGATGACCAAGATGGAGTACAAGATAACGGATCCACAAATGTTT
TRINITY_DN7_c0_g3_i1         CAGGAAGTGTTTCGCAGGATGACCAAGATGGAGTACAAGATAACGGATCCACAAATGTTT
TRINITY_DN10_c0_g1_i2        CAGGAAGTGTTTCGCAGGATGACCAAGATGGAGTACAAGATAACGGATCCACAAATGTTT
TRINITY_DN10_c0_g1_i1        CAGGAAGTGTTTCGCAGGATGACCAAGATGGAGTACAAGATAACGGATCCACAAATGTTT
                             ************************************************************

MpDNV_mRNA_seq_consensus     TTCAAGAAGAAGAAGAGGGCAGCCAAGAGGGCCCGCGGCAAAAGAAGTCGCGACGGAAGC
TRINITY_DN8906_c0_g1_i1      TTCAAGAAGAAGAAGAGGGCAGCCAAGAGGGCCCGCGGCAAAAGAAGTCGCGACGGAAGC
TRINITY_DN8906_c0_g1_i2      TTCAAGAAGAAGAAGAGGGCAGCCAAGAGGGCCCGCGGCAAAAGAAGTCGCGACGGAAGC
TRINITY_DN7_c0_g3_i1         TTCAAGAAGAAGAAGAGGGCAGCCAAGAGGGCCCGCGGCAAAAGAAGTCGCGACGGAAGC
TRINITY_DN10_c0_g1_i2        TTCAAGAAGAAGAAGAGGGCAGCCAAGAGGGCCCGCGGCAAAAGAAGTCGCGACGGAAGC
TRINITY_DN10_c0_g1_i1        TTCAAGAAGAAGAAGAGGGCAGCCAAGAGGGCCCGCGGCAAAAGAAGTCGCGACGGAAGC
                             ************************************************************

MpDNV_mRNA_seq_consensus     AGCGATTCCGAATGATTAGGGAAATAGATGTTAGTAAACCGCAATCTATCGAAGAACTTA
TRINITY_DN8906_c0_g1_i1      AGCGATTCCGAATGATTAGGGAAATAGATGTTAGTAAACCGCAATCTATCGAAGAACTTA
TRINITY_DN8906_c0_g1_i2      AGCGATTCCGAATGATTAGGGAAATAGATGTTAGTAAACCGCAATCTATCGAAGAACTTA
TRINITY_DN7_c0_g3_i1         AGCGATTCCGAATGATTAGGGAAATAGATGTTAGTAAACCGCAATCTATCGAAGAACTTA
TRINITY_DN10_c0_g1_i2        AGCGATTCCGAATGATTAGGGAAATAGATGTTAGTAAACCGCAATCTATCGAAGAACTTA
TRINITY_DN10_c0_g1_i1        AGCGATTCCGAATGATTAGGGAAATAGATGTTAGTAAACCGCAATCTATCGAAGAACTTA
                             ************************************************************

MpDNV_mRNA_seq_consensus     TATATAGGTATCCTTGTTGCCCCCCGGAAGCTTTCTATAACATTCCCGAATTTTACGCTA
TRINITY_DN8906_c0_g1_i1      TATATAGGTATCCTTGTTGCCCCCCGGAAGCTTTCTATAACATTCCCGAATTTTACGCTA
TRINITY_DN8906_c0_g1_i2      TATATAGGTATCCTTGTTGCCCCCCGGAAGCTTTCTATAACATTCCCGAATTTTACGCTA
```

```
TRINITY_DN7_c0_g3_i1         TATATAGGTATCCTTGTTGCCCCCCGGAAGCTTTCTATAACATTCCCGAATTTTACGCTA
TRINITY_DN10_c0_g1_i2        TATATAGGTATCCTTGTTGCCCCCCGGAAGCTTTCTATAACATTCCCGAATTTTACGCTA
TRINITY_DN10_c0_g1_i1        TATATAGGTATCCTTGTTGCCCCCCGGAAGCTTTCTATAACATTCCCGAATTTTACGCTA
                             ************************************************************

MpDNV_mRNA_seq_consensus     ATACAAATATAAACTGGATAGACATGAAAGATTTTAAAGTAACGATACCGTTACGTAATT
TRINITY_DN8906_c0_g1_i1      ATACAAATATAAACTGGATAGACATGAAAGATTTTAAAGTAACGATACCGTTACGTAATT
TRINITY_DN8906_c0_g1_i2      ATACAAATATAAACTGGATAGACATGAAAGATTTTAAAGTAACGATACCGTTACGTAATT
TRINITY_DN7_c0_g3_i1         ATACAAATATAAACTGGATAGACATGAAAGATTTTAAAGTAACGATACCGTTACGTAATT
TRINITY_DN10_c0_g1_i2        ATACAAATATAAACTGGATAGACATGAAAGATTTTAAAGTAACGATACCGTTACGTAATT
TRINITY_DN10_c0_g1_i1        ATACAAATATAAACTGGATAGACATGAAAGATTTTAAAGTAACGATACCGTTACGTAATT
                             ************************************************************

MpDNV_mRNA_seq_consensus     GGGCGGCCACTTTACGCAGATGGTCTATTCATGATTTCAATAAATATTATAATGACTCTA
TRINITY_DN8906_c0_g1_i1      GGGCGGCCACTTTACGCAGATGGTCTATTCATGATTTCAATAAATATTATAATGACTCTA
TRINITY_DN8906_c0_g1_i2      GGGCGGCCACTTTACGCAGATGGTCTATTCATGATTTCAATAAATATTATAATGACTCTA
TRINITY_DN7_c0_g3_i1         GGGCGGCCACTTTACGCAGATGGTCTATTCATGATTTCAATAAATATTATAATGACTCTA
TRINITY_DN10_c0_g1_i2        GGGCGGCCACTTTACGCAGATGGTCTATTCATGATTTCAATAAATATTATAATGACTCTA
TRINITY_DN10_c0_g1_i1        GGGCGGCCACTTTACGCAGATGGTCTATTCATGATTTCAATAAATATTATAATGACTCTA
                             ************************************************************

MpDNV_mRNA_seq_consensus     CAGTTTTTCCATATTTCAACGCGTACGGTTCAGATATTTGCAATATGTATTACAGTATTT
TRINITY_DN8906_c0_g1_i1      CAGTTTTTCCATATTTCAACGCGTACGGTTCAGATATTTGCAATATGTATTACAGTATTT
TRINITY_DN8906_c0_g1_i2      CAGTTTTTCCATATTTCAACGCGTACGGTTCAGATATTTGCAATATGTATTACAGTATTT
TRINITY_DN7_c0_g3_i1         CAGTTTTTCCATATTTCAACGCGTACGGTTCAGATATTTGCAATATGTATTACAGTATTT
TRINITY_DN10_c0_g1_i2        CAGTTTTTCCATATTTCAACGCGTACGGTTCAGATATTTGCAATATGTATTACAGTATTT
TRINITY_DN10_c0_g1_i1        CAGTTTTTCCATATTTCAACGCGTACGGTTCAGATATTTGCAATATGTATTACAGTATTT
                             ************************************************************

MpDNV_mRNA_seq_consensus     CGGAGAGTCTGACAATAGCAAAAGAATTACTCAACTATCAATTTGGCGATGACCCTGAAG
TRINITY_DN8906_c0_g1_i1      CGGAGAGTCTGACAATAGCAAAAGAATTACTCAACTATCAATTTGGCGATGACCCTGAAG
TRINITY_DN8906_c0_g1_i2      CGGAGAGTCTGACAATAGCAAAAGAATTACTCAACTATCAATTTGGCGATGACCCTGAAG
TRINITY_DN7_c0_g3_i1         CGGAGAGTCTGACAATAGCAAAAGAATTACTCAACTATCAATTTGGCGATGACCCTGAAG
TRINITY_DN10_c0_g1_i2        CGGAGAGTCTGACAATAGCAAAAGAATTACTCAACTATCAATTTGGCGATGACCCTGAAG
TRINITY_DN10_c0_g1_i1        CGGAGAGTCTGACAATAGCAAAAGAATTACTCAACTATCAATTTGGCGATGACCCTGAAG
                             ************************************************************

MpDNV_mRNA_seq_consensus     TAGTAATTGAATTTTTAACAACACTATATAATGTCATAGACAAAAGAGTTCCGAAATTAA
TRINITY_DN8906_c0_g1_i1      TAGTAATTGAATTTTTAACAACACTATATAATGTCATAGACAAAAGAGTTCCGAAATTAA
TRINITY_DN8906_c0_g1_i2      TAGTAATTGAATTTTTAACAACACTATATAATGTCATAGACAAAAGAGTTCCGAAATTAA
TRINITY_DN7_c0_g3_i1         TAGTAATTGAATTTTTAACAACACTATATAATGTCATAGACAAAAGAGTTCCGAAATTAA
TRINITY_DN10_c0_g1_i2        TAGTAATTGAATTTTTAACAACACTATATAATGTCATAGACAAAAGAGTTCCGAAATTAA
TRINITY_DN10_c0_g1_i1        TAGTAATTGAATTTTTAACAACACTATATAATGTCATAGACAAAAGAGTTCCGAAATTAA
                             ************************************************************

MpDNV_mRNA_seq_consensus     ATAGTATATGTATAAAAAGTCCCCCTTCGGCAGGTAAGAACTTTTTTTTCGATGCCGTTG
TRINITY_DN8906_c0_g1_i1      ATAGTATATGTATAAAAAGTCCCCCTTCGGCAGGTAAGAACTTTTTTTTCGATGCCGTTG
TRINITY_DN8906_c0_g1_i2      ATAGTATATGTATAAAAAGTCCCCCTTCGGCAGGTAAGAACTTTTTTTTCGATGCCGTTG
TRINITY_DN7_c0_g3_i1         ATAGTATATGTATAAAAAGTCCCCCTTCGGCAGGTAAGAACTTTTTTTTCGATGCCGTTG
TRINITY_DN10_c0_g1_i2        ATAGTATATGTATAAAAAGTCCCCCTTCGGCAGGTAAGAACTTTTTTTTCGATGCCGTTG
TRINITY_DN10_c0_g1_i1        ATAGTATATGTATAAAAAGTCCCCCTTCGGCAGGTAAGAACTTTTTTTTCGATGCCGTTG
                             ************************************************************

MpDNV_mRNA_seq_consensus     CATCGTATTTGCTTTCATATGGTATGTTCGGTACAGCCAATAAAAACAATAATTTTTCGT
TRINITY_DN8906_c0_g1_i1      CATCGTATTTGCTTTCATATGGCATGTTCGGTACAGCCAATAAAAACAATAATTTTTCGT
TRINITY_DN8906_c0_g1_i2      CATCGTATTTGCTTTCATATGGCATGTTCGGTACAGCCAATAAAAACAATAATTTTTCGT
TRINITY_DN7_c0_g3_i1         CATCGTATTTGCTTTCATATGGCATGTTCGGTACAGCCAATAAAAACAATAATTTTTCGT
TRINITY_DN10_c0_g1_i2        CATCGTATTTGCTTTCATATGGCATGTTCGGTACAGCCAATAAAAACAATAATTTTTCGT
TRINITY_DN10_c0_g1_i1        CATCGTATTTGCTTTCATATGGCATGTTCGGTACAGCCAATAAAAACAATAATTTTTCGT
                             ********************** *************************************

MpDNV_mRNA_seq_consensus     GGGCAGACGGAGCGGGTAAACGATTAGTTCTGTGGAACGAACCAAACTATGAACAATACC
TRINITY_DN8906_c0_g1_i1      GGGCAGACGGAGCGGGTAAACGATTAGTTCTGTGGAACGAACCAAACTATGAACAATACC
TRINITY_DN8906_c0_g1_i2      GGGCAGACGGAGCGGGTAAACGATTAGTTCTGTGGAACGAACCAAACTATGAACAATACC
TRINITY_DN7_c0_g3_i1         GGGCAGACGGAGCGGGTAAACGATTAGTTCTGTGGAACGAACCAAACTATGAACAATACC
TRINITY_DN10_c0_g1_i2        GGGCAGACGGAGCGGGTAAACGATTAGTTCTGTGGAACGAACCAAACTATGAACAATACC
TRINITY_DN10_c0_g1_i1        GGGCAGACGGAGCGGGTAAACGATTAGTTCTGTGGAACGAACCAAACTATGAACAATACC
                             ************************************************************

MpDNV_mRNA_seq_consensus     ATATAGAAAAAATAAAAGAACTTTTGGGGGGAGATACAACAAGAATACATGTCAAATATG
TRINITY_DN8906_c0_g1_i1      ATATAGAAAAAATAAAAGAACTTTTGGGGGGAGATACAACAAGAATACATGTCAAATATG
TRINITY_DN8906_c0_g1_i2      ATATAGAAAAAATAAAAGAACTTTTGGGGGGAGATACAACAAGAATACATGTCAAATATG
TRINITY_DN7_c0_g3_i1         ATATAGAAAAAATAAAAGAACTTTTGGGGGGAGATACAACAAGAATACATGTCAAATATG
TRINITY_DN10_c0_g1_i2        ATATAGAAAAAATAAAAGAACTTTTGGGGGGAGATACAACAAGAATACATGTCAAATATG
TRINITY_DN10_c0_g1_i1        ATATAGAAAAAATAAAAGAACTTTTGGGGGGAGATACAACAAGAATACATGTCAAATATG
                             ************************************************************

MpDNV_mRNA_seq_consensus     CAAACGACGTTAGTGTGCAAAGGGTACCCATTATTATATTAACAAATAACCACTTGAATA
TRINITY_DN8906_c0_g1_i1      CAAACGACGTTAGTGTGCAAAGGGTACCCATTATTATATTAACAAATAACCACTTGAATA
TRINITY_DN8906_c0_g1_i2      CAAACGACGTTAGTGTGCAAAGGGTACCCATTATTATATTAACAAATAACCACTTGAATA
TRINITY_DN7_c0_g3_i1         CAAACGACGTTAGTGTGCAAAGGGTACCCATTATTATATTAACAAATAACCACTTGAATA
TRINITY_DN10_c0_g1_i2        CAAACGACGTTAGTGTGCAAAGGGTACCCATTATTATATTAACAAATAACCACTTGAATA
TRINITY_DN10_c0_g1_i1        CAAACGACGTTAGTGTGCAAAGGGTACCCATTATTATATTAACAAATAACCACTTGAATA
                             ************************************************************

MpDNV_mRNA_seq_consensus     TTATTAGTCACCCAGCTTTTAACGATAGATTGCGGAGTTACGAATGGATGTCAGCTGGCT
TRINITY_DN8906_c0_g1_i1      TTATTAGTCACCCAGCTTTTAACGATAGATTGCGGAGTTACGAATGGATGTCAGCTGGCT
TRINITY_DN8906_c0_g1_i2      TTATTAGTCACCCAGCTTTTAACGATAGATTGCGGAGTTACGAATGGATGTCAGCTGGCT
```

```
TRINITY_DN7_c0_g3_i1          TTATTAGTCACCCAGCTTTTAACGATAGATTGCGGAGTTACGAATGGATGTCAGCTGGCT
TRINITY_DN10_c0_g1_i2         TTATTAGTCACCCAGCTTTTAACGATAGATTGCGGAGTTACGAATGGATGTCAGCTGGCT
TRINITY_DN10_c0_g1_i1         TTATTAGTCACCCAGCTTTTAACGATAGATTGCGGAGTTACGAATGGATGTCAGCTGGCT
                              ************************************************************

MpDNV_mRNA_seq_consensus      TCTTGAAGGACTACGATAAAAAACTGCATCCGCTTATGTTTTATGAATTACTTAAAGATT
TRINITY_DN8906_c0_g1_i1       TCTTGAAGGACTACGATAAAAAACTGCATCCGCTTATGTTTTATGAATTACTTAAAGATT
TRINITY_DN8906_c0_g1_i2       TCTTGAAGGACTACGATAAAAAACTGCATCCGCTTATGTTTTATGAATTACTTAAAGATT
TRINITY_DN7_c0_g3_i1          TCTTGAAGGACTACGATAAAAAACTGCATCCGCTTATGTTTTATGAATTACTTAAAGATT
TRINITY_DN10_c0_g1_i2         TCTTGAAGGACTACGATAAAAAACTGCATCCGCTTATGTTTTATGAATTACTTAAAGATT
TRINITY_DN10_c0_g1_i1         TCTTGAAGGACTACGATAAAAAACTGCATCCGCTTATGTTTTATGAATTACTTAAAGATT
                              ************************************************************

MpDNV_mRNA_seq_consensus      ATGGTGTAATAAATGATAACAATATGATTGATTAATATATATATATATATATATTGATTTTT NS polyA signal
TRINITY_DN8906_c0_g1_i1 APFV8 ATGGTGTAATAAATGATAACAATATGATTGATTAATATATATATATATATAAAAAAAAA NS poly(A) tail
TRINITY_DN8906_c0_g1_i2 APFV8 ATGGTGTAATAAATGATAACAATATGATTGATTAATATATATATATATATAAAAAAAAA NS poly(A) tail
TRINITY_DN7_c0_g3_i1    APFV9 ATGGTGTAATAAATGATAACAATATGATTGATTAATATATATATATAAAAAAAAAA--- NS poly(A) tail
TRINITY_DN10_c0_g1_i2  APFV10 ATGGTGTAATAAATGATAACAATATGATTGATTAATATATATATATATAT--TGATTTTT
TRINITY_DN10_c0_g1_i1  APFV10 ATGGTGTAATAAATGATAACAATATGATTGATTAATATATATATATATAT--TGATTTTT
                              ***********************************************

MpDNV_mRNA_seq_consensus      ATTTACGTAAAAAATCTACAGGTTCATTGACAATGGGTATATTCATTGACCTTCGCCACA
TRINITY_DN8906_c0_g1_i1       AA----------------------------------------------------------
TRINITY_DN8906_c0_g1_i2       AA----------------------------------------------------------
TRINITY_DN7_c0_g3_i1          ------------------------------------------------------------
TRINITY_DN10_c0_g1_i2         ATTTACGTAAAAAAAAA------------------------------------------- NS poly(A) tail
TRINITY_DN10_c0_g1_i1         ATTTACGTAAAAAAAAA------------------------------------------- NS poly(A) tail

MpDNV_mRNA_seq_consensus      TGGTGTAATAAATGATAACAATATGATTGATTAATATATATATATATATATATTGATTTTTA VP polyA signal
TRINITY_DN34_c0_g2_i3  APFV10 ----------------TTTTTTTTTTTTGATTAATATATATATATATATATATTGATTTTTA VP poly(A) tail
TRINITY_DN34_c0_g2_i2  APFV10 ----------------TTTTTTTTTTTTGATTAATATATATATATATATATATTGATTTTTA VP poly(A) tail
TRINITY_DN34_c0_g2_i1  APFV10 ----------------TTTTTTTTTTTTGATTAATATATATATATATATATATTGATTTTTA VP poly(A) tail
TRINITY_DN34_c0_g2_i4  APFV10 ----------------TTTTTTTTTTTTGATTAATATATATATATATATATATTGATTTTTA VP poly(A) tail
TRINITY_DN29_c0_g1_i2  APFV8  ---------------TTTTTTTTTTTTTGATTAATATATATATATATATATATTGATTTTTA VP poly(A) tail
TRINITY_DN29_c0_g1_i3  APFV8  ---------------TTTTTTTTTTTTTGATTAATATATATATATATATATATTGATTTTTA VP poly(A) tail
TRINITY_DN29_c0_g1_i4  APFV8  ---------------TTTTTTTTTTTTTGATTAATATATATATATATATATATTGATTTTTA VP poly(A) tail
TRINITY_DN29_c0_g1_i1  APFV8  ---------------TTTTTTTTTTTTTGATTAATATATATATATATATATATTGATTTTTA VP poly(A) tail
TRINITY_DN7_c0_g2_i1   APFV9  ----------------TTTTTTTTTTTTGATTAATATATATATATATATATATTGATTTTTA VP poly(A) tail
TRINITY_DN7_c0_g2_i4   APFV9  ----------------TTTTTTTTTTTTGATTAATATATATATATATATATATTGATTTTTA VP poly(A) tail
TRINITY_DN7_c0_g2_i3   APFV9  ----------------TTTTTTTTTTTTGATTAATATATATATATATATATATTGATTTTTA VP poly(A) tail
TRINITY_DN7_c0_g2_i2   APFV9  ----------------TTTTTTTTTTTTGATTAATATATATATATATATATATTGATTTTTA VP poly(A) tail
                                                          **********************************

MpDNV_mRNA_seq_consensus      TTTACGTAAAAAATCTACAGGTTCATTGACAATGGGTATATTCATTGACCTTCGCCACAA
TRINITY_DN34_c0_g2_i3         TTTACGTAAAAAATCTACAGGTTCATTGACAATGGGTATATTCATTGACCTTCGCCACAA
TRINITY_DN34_c0_g2_i2         TTTACGTAAAAAATCTACAGGTTCATTGACAATGGGTATATTCATTGACCTTCGCCACAA
TRINITY_DN34_c0_g2_i1         TTTACGTAAAAAATCTACAGGTTCATTGACAATGGGTATATTCATTGACCTTCGCCACAA
TRINITY_DN34_c0_g2_i4         TTTACGTAAAAAATCTACAGGTTCATTGACAATGGGTATATTCATTGACCTTCGCCACAA
TRINITY_DN29_c0_g1_i2         TTTACGTAAAAAATCTACAGGTTCATTGACAATGGGTATATTCATTGACCTTCGCCACAA
TRINITY_DN29_c0_g1_i3         TTTACGTAAAAAATCTACAGGTTCATTGACAATGGGTATATTCATTGACCTTCGCCACAA
TRINITY_DN29_c0_g1_i4         TTTACGTAAAAAATCTACAGGTTCATTGACAATGGGTATATTCATTGACCTTCGCCACAA
TRINITY_DN29_c0_g1_i1         TTTACGTAAAAAATCTACAGGTTCATTGACAATGGGTATATTCATTGACCTTCGCCACAA
TRINITY_DN7_c0_g2_i1          TTTACGTAAAAAATCTACAGGTTCATTGACAATGGGTATATTCATTGACCTTCGCCACAA
TRINITY_DN7_c0_g2_i4          TTTACGTAAAAAATCTACAGGTTCATTGACAATGGGTATATTCATTGACCTTCGCCACAA
TRINITY_DN7_c0_g2_i3          TTTACGTAAAAAATCTACAGGTTCATTGACAATGGGTATATTCATTGACCTTCGCCACAA
TRINITY_DN7_c0_g2_i2          TTTACGTAAAAAATCTACAGGTTCATTGACAATGGGTATATTCATTGACCTTCGCCACAA
                              ************************************************************

MpDNV_mRNA_seq_consensus      TGTTAAATCACGGCAAAAAGCTAAATACGTTGAATATGACTTGAACAATCGTTCCTCTGG
TRINITY_DN34_c0_g2_i3         TGTTAAATCACGGCAAAAAGCTAAATACGTTGAATATGACTTGAACAATCGTTCCTCTGG
TRINITY_DN34_c0_g2_i2         TGTTAAATCACGGCAAAAAGCTAAATACGTTGAATATGACTTGAACAATCGTTCCTCTGG
TRINITY_DN34_c0_g2_i1         TGTTAAATCACGGCAAAAAGCTAAATACGTTGAATATGACTTGAACAATCGTTCCTCTGG
TRINITY_DN34_c0_g2_i4         TGTTAAATCACGGCAAAAAGCTAAATACGTTGAATATGACTTGAACAATCGTTCCTCTGG
TRINITY_DN29_c0_g1_i2         TGTTAAATCACGGCAAAAAGCTAAATACGTTGAATATGACTTGAACAATCGTTCCTCTGG
TRINITY_DN29_c0_g1_i3         TGTTAAATCACGGCAAAAAGCTAAATACGTTGAATATGACTTGAACAATCGTTCCTCTGG
TRINITY_DN29_c0_g1_i4         TGTTAAATCACGGCAAAAAGCTAAATACGTTGAATATGACTTGAACAATCGTTCCTCTGG
TRINITY_DN29_c0_g1_i1         TGTTAAATCACGGCAAAAAGCTAAATACGTTGAATATGACTTGAACAATCGTTCCTCTGG
TRINITY_DN7_c0_g2_i1          TGTTAAATCACGGCAAAAAGCTAAATACGTTGAATATGACTTGAACAATCGTTCCTCTGG
TRINITY_DN7_c0_g2_i4          TGTTAAATCACGGCAAAAAGCTAAATACGTTGAATATGACTTGAACAATCGTTCCTCTGG
TRINITY_DN7_c0_g2_i3          TGTTAAATCACGGCAAAAAGCTAAATACGTTGAATATGACTTGAACAATCGTTCCTCTGG
TRINITY_DN7_c0_g2_i2          TGTTAAATCACGGCAAAAAGCTAAATACGTTGAATATGACTTGAACAATCGTTCCTCTGG
                              ************************************************************

MpDNV_mRNA_seq_consensus      CCACCAATTCGGATTATTGTAGTCGCCCCAAGAAGTGTTCATGATTTCTTCGGTTGTGGG
TRINITY_DN34_c0_g2_i3         CCACCAATTCGGATTATTGTAGTCGCCCCAAGAAGTGTTCATGATTTCTTCGGTTGTGGG
TRINITY_DN34_c0_g2_i2         CCACCAATTCGGATTATTGTAGTCGCCCCAAGAAGTGTTCATGATTTCTTCGGTTGTGGG
TRINITY_DN34_c0_g2_i1         CCACCAATTCGGATTATTGTAGTCGCCCCAAGAAGTGTTCATGATTTCTTCGGTTGTGGG
TRINITY_DN34_c0_g2_i4         CCACCAATTCGGATTATTGTAGTCGCCCCAAGAAGTGTTCATGATTTCTTCGGTTGTGGG
TRINITY_DN29_c0_g1_i2         CCACCAATTCGGATTATTGTAGTCGCCCCAAGAAGTGTTCATGATTTCTTCGGTTGTGGG
TRINITY_DN29_c0_g1_i3         CCACCAATTCGGATTATTGTAGTCGCCCCAAGAAGTGTTCATGATTTCTTCGGTTGTGGG
TRINITY_DN29_c0_g1_i4         CCACCAATTCGGATTATTGTAGTCGCCCCAAGAAGTGTTCATGATTTCTTCGGTTGTGGG
TRINITY_DN29_c0_g1_i1         CCACCAATTCGGATTATTGTAGTCGCCCCAAGAAGTGTTCATGATTTCTTCGGTTGTGGG
TRINITY_DN7_c0_g2_i1          CCACCAATTCGGATTATTGTAGTCGCCCCAAGAAGTGTTCATGATTTCTTCGGTTGTGGG
TRINITY_DN7_c0_g2_i4          CCACCAATTCGGATTATTGTAGTCGCCCCAAGAAGTGTTCATGATTTCTTCGGTTGTGGG
TRINITY_DN7_c0_g2_i3          CCACCAATTCGGATTATTGTAGTCGCCCCAAGAAGTGTTCATGATTTCTTCGGTTGTGGG
TRINITY_DN7_c0_g2_i2          CCACCAATTCGGATTATTGTAGTCGCCCCAAGAAGTGTTCATGATTTCTTCGGTTGTGGG
                              ************************************************************
```

```
MpDNV_mRNA_seq_consensus      TGGAGTAGTGGTATATCGATGCAAATTAATTAGGGTTTCTTTTGACGCACGATCACTTTC
TRINITY_DN34_c0_g2_i3         TGGAGTAGTGGTATATCGATGCAAATTAATTAGGGTTTCTTTTGACGCACGATCACTTTC
TRINITY_DN34_c0_g2_i2         TGGAGTAGTGGTATATCGATGCAAATTAATTAGGGTTTCTTTTGACGCACGATCACTTTC
TRINITY_DN34_c0_g2_i1         TGGAGTAGTGGTATATCGATGCAAATTAATTAGGGTTTCTTTTGACGCACGATCACTTTC
TRINITY_DN34_c0_g2_i4         TGGAGTAGTGGTATATCGATGCAAATTAATTAGGGTTTCTTTTGACGCACGATCACTTTC
TRINITY_DN29_c0_g1_i2         TGGAGTAGTGGTATATCGATGCAAATTAATTAGGGTTTCTTTTGACGCACGATCACTTTC
TRINITY_DN29_c0_g1_i3         TGGAGTAGTGGTATATCGATGCAAATTAATTAGGGTTTCTTTTGACGCACGATCACTTTC
TRINITY_DN29_c0_g1_i4         TGGAGTAGTGGTATATCGATGCAAATTAATTAGGGTTTCTTTTGACGCACGATCACTTTC
TRINITY_DN29_c0_g1_i1         TGGAGTAGTGGTATATCGATGCAAATTAATTAGGGTTTCTTTTGACGCACGATCACTTTC
TRINITY_DN7_c0_g2_i1          TGGAGTAGTGGTATATCGATGCAAATTAATTAGGGTTTCTTTTGACGCACGATCACTTTC
TRINITY_DN7_c0_g2_i4          TGGAGTAGTGGTATATCGATGCAAATTAATTAGGGTTTCTTTTGACGCACGATCACTTTC
TRINITY_DN7_c0_g2_i3          TGGAGTAGTGGTATATCGATGCAAATTAATTAGGGTTTCTTTTGACGCACGATCACTTTC
TRINITY_DN7_c0_g2_i2          TGGAGTAGTGGTATATCGATGCAAATTAATTAGGGTTTCTTTTGACGCACGATCACTTTC
                              ************************************************************

MpDNV_mRNA_seq_consensus      TCTGTAAACATAATTGGGTATTTTAATAGGTGCTTTGACTCGAACCTCATGGATGTTTGA 3' splice site
TRINITY_DN34_c0_g2_i3         TCTGTAAACATAATTGGGTATTTTAATAGGTGCTTTGACTCGAACCTCATGGATGTTTGA VP unspliced
TRINITY_DN34_c0_g2_i2         TCTGTAAACATAATTGGGTATTTTAATAGGTGCTTTGACTCGAACCTCATGGATGTTTGA VP unspliced
TRINITY_DN34_c0_g2_i1         T----------------------------------------------------------- spliced intron 2
TRINITY_DN34_c0_g2_i4         T----------------------------------------------------------- spliced intron 2
TRINITY_DN29_c0_g1_i2         TCTGTAAACATAATTGGGTATTTTAATAGGTGCTTTGACTCGAACCTCATGGATGTTTGA VP unspliced
TRINITY_DN29_c0_g1_i3         TCTGTAAACATAATTGGGTATTTTAATAGGTGCTTTGACTCGAACCTCATGGATGTTTGA VP unspliced
TRINITY_DN29_c0_g1_i4         T----------------------------------------------------------- spliced intron 2
TRINITY_DN29_c0_g1_i1         T----------------------------------------------------------- spliced intron 2
TRINITY_DN7_c0_g2_i1          TCTGTAAACATAATTGGGTATTTTAATAGGTGCTTTGACTCGAACCTCATGGATGTTTGA VP unspliced
TRINITY_DN7_c0_g2_i4          TCTGTAAACATAATTGGGTATTTTAATAGGTGCTTTGACTCGAACCTCATGGATGTTTGA VP unspliced
TRINITY_DN7_c0_g2_i3          T----------------------------------------------------------- spliced intron 2
TRINITY_DN7_c0_g2_i2          T----------------------------------------------------------- spliced intron 2
                              *

MpDNV_mRNA_seq_consensus      CGCTTCAACCTCCATGGGGTCGTCGTCCATATTTACCCGAACAGACGTAGGTACAGGTGT 5' splice site
TRINITY_DN34_c0_g2_i3         CGCTTCAACCTCCATGGGGTCGTCGTCCATATTTACCCGAACAGACGTAGGTACAGGTGT VP unspliced
TRINITY_DN34_c0_g2_i2         CGCTTCAACCTCCATGGGGTCGTCGTCCATATTTACCCGAACAGACGTAGGTACAGGTGT VP unspliced
TRINITY_DN34_c0_g2_i1         ------------------------------------CCGAACAGACGTAGGTACAGGTGT spliced intron 2
TRINITY_DN34_c0_g2_i4         ------------------------------------CCGAACAGACGTAGGTACAGGTGT spliced intron 2
TRINITY_DN29_c0_g1_i2         CGCTTCAACCTCCATGGGGTCGTCGTCCATATTTACCCGAACAGACGTAGGTACAGGTGT VP unspliced
TRINITY_DN29_c0_g1_i3         CGCTTCAACCTCCATGGGGTCGTCGTCCATATTTACCCGAACAGACGTAGGTACAGGTGT VP unspliced
TRINITY_DN29_c0_g1_i4         ------------------------------------CCGAACAGACGTAGGTACAGGTGT spliced intron 2
TRINITY_DN29_c0_g1_i1         ------------------------------------CCGAACAGACGTAGGTACAGGTGT spliced intron 2
TRINITY_DN7_c0_g2_i1          CGCTTCAACCTCCATGGGGTCGTCGTCCATATTTACCCGAACAGACGTAGGTACAGGTGT VP unspliced
TRINITY_DN7_c0_g2_i4          CGCTTCAACCTCCATGGGGTCGTCGTCCATATTTACCCGAACAGACGTAGGTACAGGTGT VP unspliced
TRINITY_DN7_c0_g2_i3          ------------------------------------CCGAACAGACGTAGGTACAGGTGT spliced intron 2
TRINITY_DN7_c0_g2_i2          ------------------------------------CCGAACAGACGTAGGTACAGGTGT spliced intron 2
                                                                  ************************

MpDNV_mRNA_seq_consensus      AGTGACAGTTGCGTCGACTGGAGTAGTAAATGAATCAATGTTCTTTGTAGTGGAAGCTTT
TRINITY_DN34_c0_g2_i3         AGTGACAGTTGCGTCGACTGGAGTAGTAAATGAATCAATGTTCTTTGTAGTGGAAGCTTT
TRINITY_DN34_c0_g2_i2         AGTGACAGTTGCGTCGACTGGAGTAGTAAATGAATCAATGTTCTTTGTAGTGGAAGCTTT
TRINITY_DN34_c0_g2_i1         AGTGACAGTTGCGTCGACTGGAGTAGTAAATGAATCAATGTTCTTTGTAGTGGAAGCTTT
TRINITY_DN34_c0_g2_i4         AGTGACAGTTGCGTCGACTGGAGTAGTAAATGAATCAATGTTCTTTGTAGTGGAAGCTTT
TRINITY_DN29_c0_g1_i2         AGTGACAGTTGCGTCGACTGGAGTAGTAAATGAATCAATGTTCTTTGTAGTGGAAGCTTT
TRINITY_DN29_c0_g1_i3         AGTGACAGTTGCGTCGACTGGAGTAGTAAATGAATCAATGTTCTTTGTAGTGGAAGCTTT
TRINITY_DN29_c0_g1_i4         AGTGACAGTTGCGTCGACTGGAGTAGTAAATGAATCAATGTTCTTTGTAGTGGAAGCTTT
TRINITY_DN29_c0_g1_i1         AGTGACAGTTGCGTCGACTGGAGTAGTAAATGAATCAATGTTCTTTGTAGTGGAAGCTTT
TRINITY_DN7_c0_g2_i1          AGTGACAGTTGCGTCGACTGGAGTAGTAAATGAATCAATGTTCTTTGTAGTGGAAGCTTT
TRINITY_DN7_c0_g2_i4          AGTGACAGTTGCGTCGACTGGAGTAGTAAATGAATCAATGTTCTTTGTAGTGGAAGCTTT
TRINITY_DN7_c0_g2_i3          AGTGACAGTTGCGTCGACTGGAGTAGTAAATGAATCAATGTTCTTTGTAGTGGAAGCTTT
TRINITY_DN7_c0_g2_i2          AGTGACAGTTGCGTCGACTGGAGTAGTAAATGAATCAATGTTCTTTGTAGTGGAAGCTTT
                              ************************************************************

MpDNV_mRNA_seq_consensus      GCCCATTTCTTTAGTTAGTCCCGAAGAGTGTGCAGCGGCGTTTTGATTGTCAGCTACTTT
TRINITY_DN34_c0_g2_i3         GCCCATTTCTTTAGTTAGTCCCGAAGAGTGTGCAGCGGCGTTTTGATTGTCAGCTACTTT
TRINITY_DN34_c0_g2_i2         GCCCATTTCTTTAGTTAGTCCCGAAGAGTGTGCAGCGGCGTTTTGATTGTCAGCTACTTT
TRINITY_DN34_c0_g2_i1         GCCCATTTCTTTAGTTAGTCCCGAAGAGTGTGCAGCGGCGTTTTGATTGTCAGCTACTTT
TRINITY_DN34_c0_g2_i4         GCCCATTTCTTTAGTTAGTCCCGAAGAGTGTGCAGCGGCGTTTTGATTGTCAGCTACTTT
TRINITY_DN29_c0_g1_i2         GCCCATTTCTTTAGTTAGTCCCGAAGAGTGTGCAGCGGCGTTTTGATTGTCAGCTACTTT
TRINITY_DN29_c0_g1_i3         GCCCATTTCTTTAGTTAGTCCCGAAGAGTGTGCAGCGGCGTTTTGATTGTCAGCTACTTT
TRINITY_DN29_c0_g1_i4         GCCCATTTCTTTAGTTAGTCCCGAAGAGTGTGCAGCGGCGTTTTGATTGTCAGCTACTTT
TRINITY_DN29_c0_g1_i1         GCCCATTTCTTTAGTTAGTCCCGAAGAGTGTGCAGCGGCGTTTTGATTGTCAGCTACTTT
TRINITY_DN7_c0_g2_i1          GCCCATTTCTTTAGTTAGTCCCGAAGAGTGTGCAGCGGCGTTTTGATTGTCAGCTACTTT
TRINITY_DN7_c0_g2_i4          GCCCATTTCTTTAGTTAGTCCCGAAGAGTGTGCAGCGGCGTTTTGATTGTCAGCTACTTT
TRINITY_DN7_c0_g2_i3          GCCCATTTCTTTAGTTAGTCCCGAAGAGTGTGCAGCGGCGTTTTGATTGTCAGCTACTTT
TRINITY_DN7_c0_g2_i2          GCCCATTTCTTTAGTTAGTCCCGAAGAGTGTGCAGCGGCGTTTTGATTGTCAGCTACTTT
                              ************************************************************

MpDNV_mRNA_seq_consensus      TTCAGGAGCCACGTACAATCCGTGAACAATAGATAAATCTCCACGATTGTATAGATTTTG
TRINITY_DN34_c0_g2_i3         TTCAGGAGCCACGTACAATCCGTGAACAATAGATAAATCTCCACGATTGTATAGATTTTG
TRINITY_DN34_c0_g2_i2         TTCAGGAGCCACGTACAATCCGTGAACAATAGATAAATCTCCACGATTGTATAGATTTTG
TRINITY_DN34_c0_g2_i1         TTCAGGAGCCACGTACAATCCGTGAACAATAGATAAATCTCCACGATTGTATAGATTTTG
TRINITY_DN34_c0_g2_i4         TTCAGGAGCCACGTACAATCCGTGAACAATAGATAAATCTCCACGATTGTATAGATTTTG
TRINITY_DN29_c0_g1_i2         TTCAGGAGCCACGTACAATCCGTGAACAATAGATAAATCTCCACGATTGTATAGATTTTG
TRINITY_DN29_c0_g1_i3         TTCAGGAGCCACGTACAATCCGTGAACAATAGATAAATCTCCACGATTGTATAGATTTTG
TRINITY_DN29_c0_g1_i4         TTCAGGAGCCACGTACAATCCGTGAACAATAGATAAATCTCCACGATTGTATAGATTTTG
TRINITY_DN29_c0_g1_i1         TTCAGGAGCCACGTACAATCCGTGAACAATAGATAAATCTCCACGATTGTATAGATTTTG
TRINITY_DN7_c0_g2_i1          TTCAGGAGCCACGTACAATCCGTGAACAATAGATAAATCTCCACGATTGTATAGATTTTG
TRINITY_DN7_c0_g2_i4          TTCAGGAGCCACGTACAATCCGTGAACAATAGATAAATCTCCACGATTGTATAGATTTTG
TRINITY_DN7_c0_g2_i3          TTCAGGAGCCACGTACAATCCGTGAACAATAGATAAATCTCCACGATTGTATAGATTTTG
```

```
TRINITY_DN7_c0_g2_i2          TTCAGGAGCCACGTACAATCCGTGAACAATAGATAAATCTCCACGATTGTATAGATTTTG
                              ************************************************************

MpDNV_mRNA_seq_consensus      ATTGTAATTGTCGTCGGCCAGCAACCATGTCTTTTCGTATTCCACGTGAAGCCTGTCAAA
TRINITY_DN34_c0_g2_i3         ATTGTAATTGTCGTCGGCCAGCAACCATGTCTTTTCGTATTCCACGTGAAGCCTGTCAAA
TRINITY_DN34_c0_g2_i2         ATTGTAATTGTCGTCGGCCAGCAACCATGTCTTTTCGTATTCCACGTGAAGCCTGTCAAA
TRINITY_DN34_c0_g2_i1         ATTGTAATTGTCGTCGGCCAGCAACCATGTCTTTTCGTATTCCACGTGAAGCCTGTCAAA
TRINITY_DN34_c0_g2_i4         ATTGTAATTGTCGTCGGCCAGCAACCATGTCTTTTCGTATTCCACGTGAAGCCTGTCAAA
TRINITY_DN29_c0_g1_i2         ATTGTAATTGTCGTCGGCCAGCAACCATGTCTTTTCGTATTCCACGTGAAGCCTGTCAAA
TRINITY_DN29_c0_g1_i3         ATTGTAATTGTCGTCGGCCAGCAACCATGTCTTTTCGTATTCCACGTGAAGCCTGTCAAA
TRINITY_DN29_c0_g1_i4         ATTGTAATTGTCGTCGGCCAGCAACCATGTCTTTTCGTATTCCACGTGAAGCCTGTCAAA
TRINITY_DN29_c0_g1_i1         ATTGTAATTGTCGTCGGCCAGCAACCATGTCTTTTCGTATTCCACGTGAAGCCTGTCAAA
TRINITY_DN7_c0_g2_i1          ATTGTAATTGTCGTCGGCCAGCAACCATGTCTTTTCGTATTCCACGTGAAGCCTGTCAAA
TRINITY_DN7_c0_g2_i4          ATTGTAATTGTCGTCGGCCAGCAACCATGTCTTTTCGTATTCCACGTGAAGCCTGTCAAA
TRINITY_DN7_c0_g2_i3          ATTGTAATTGTCGTCGGCCAGCAACCATGTCTTTTCGTATTCCACGTGAAGCCTGTCAAA
TRINITY_DN7_c0_g2_i2          ATTGTAATTGTCGTCGGCCAGCAACCATGTCTTTTCGTATTCCACGTGAAGCCTGTCAAA
                              ************************************************************

MpDNV_mRNA_seq_consensus      CGCTGTTCTGCTTTGCGGACATCCAAAACTAACTGTCATCTCAGTTTCGAAATCCCATGT
TRINITY_DN34_c0_g2_i3         CGCTGTTCTGCTTTGCGGACATCCAAAACTAACTGTCATCTCAGTTTCGAAATCCCATGT
TRINITY_DN34_c0_g2_i2         CGCTGTTCTGCTTTGCGGACATCCAAAACTAACTGTCATCTCAGTTTCGAAATCCCATGT
TRINITY_DN34_c0_g2_i1         CGCTGTTCTGCTTTGCGGACATCCAAAACTAACTGTCATCTCAGTTTCGAAATCCCATGT
TRINITY_DN34_c0_g2_i4         CGCTGTTCTGCTTTGCGGACATCCAAAACTAACTGTCATCTCAGTTTCGAAATCCCATGT
TRINITY_DN29_c0_g1_i2         CGCTGTTCTGCTTTGCGGACATCCAAAACTAACTGTCATCTCAGTTTCGAAATCCCATGT
TRINITY_DN29_c0_g1_i3         CGCTGTTCTGCTTTGCGGACATCCAAAACTAACTGTCATCTCAGTTTCGAAATCCCATGT
TRINITY_DN29_c0_g1_i4         CGCTGTTCTGCTTTGCGGACATCCAAAACTAACTGTCATCTCAGTTTCGAAATCCCATGT
TRINITY_DN29_c0_g1_i1         CGCTGTTCTGCTTTGCGGACATCCAAAACTAACTGTCATCTCAGTTTCGAAATCCCATGT
TRINITY_DN7_c0_g2_i1          CGCTGTTCTGCTTTGCGGACATCCAAAACTAACTGTCATCTCAGTTTCGAAATCCCATGT
TRINITY_DN7_c0_g2_i4          CGCTGTTCTGCTTTGCGGACATCCAAAACTAACTGTCATCTCAGTTTCGAAATCCCATGT
TRINITY_DN7_c0_g2_i3          CGCTGTTCTGCTTTGCGGACATCCAAAACTAACTGTCATCTCAGTTTCGAAATCCCATGT
TRINITY_DN7_c0_g2_i2          CGCTGTTCTGCTTTGCGGACATCCAAAACTAACTGTCATCTCAGTTTCGAAATCCCATGT
                              ************************************************************

MpDNV_mRNA_seq_consensus      GCATTCTACGTCAGTAAAATTTACAGGGACTATACTATTAGTGGCAGTAGTTAACTTATG
TRINITY_DN34_c0_g2_i3         GCATTCTACGTCAGTAAAATTTACAGGGACTATACTATTAGTGGCAGTAGTTAACTTATG
TRINITY_DN34_c0_g2_i2         GCATTCTACGTCAGTAAAATTTACAGGGACTATACTATTAGTGGCAGTAGTTAACTTATG
TRINITY_DN34_c0_g2_i1         GCATTCTACGTCAGTAAAATTTACAGGGACTATACTATTAGTGGCAGTAGTTAACTTATG
TRINITY_DN34_c0_g2_i4         GCATTCTACGTCAGTAAAATTTACAGGGACTATACTATTAGTGGCAGTAGTTAACTTATG
TRINITY_DN29_c0_g1_i2         GCATTCTACGTCAGTAAAATTTACAGGGACTATACTATTAGTGGCAGTAGTTAACTTATG
TRINITY_DN29_c0_g1_i3         GCATTCTACGTCAGTAAAATTTACAGGGACTATACTATTAGTGGCAGTAGTTAACTTATG
TRINITY_DN29_c0_g1_i4         GCATTCTACGTCAGTAAAATTTACAGGGACTATACTATTAGTGGCAGTAGTTAACTTATG
TRINITY_DN29_c0_g1_i1         GCATTCTACGTCAGTAAAATTTACAGGGACTATACTATTAGTGGCAGTAGTTAACTTATG
TRINITY_DN7_c0_g2_i1          GCATTCTACGTCAGTAAAATTTACAGGGACTATACTATTAGTGGCAGTAGTTAACTTATG
TRINITY_DN7_c0_g2_i4          GCATTCTACGTCAGTAAAATTTACAGGGACTATACTATTAGTGGCAGTAGTTAACTTATG
TRINITY_DN7_c0_g2_i3          GCATTCTACGTCAGTAAAATTTACAGGGACTATACTATTAGTGGCAGTAGTTAACTTATG
TRINITY_DN7_c0_g2_i2          GCATTCTACGTCAGTAAAATTTACAGGGACTATACTATTAGTGGCAGTAGTTAACTTATG
                              ************************************************************

MpDNV_mRNA_seq_consensus      CACTGGATATATTCCTACATGTACACTAGGTTGTACTTTATTAGAATATTCATGTTTATC
TRINITY_DN34_c0_g2_i3         CACTGGATATATTCCTACATGTACACTAGGTTGTACTTTATTAGAATATTCATGTTTATC
TRINITY_DN34_c0_g2_i2         CACTGGATATATTCCTACATGTACACTAGGTTGTACTTTATTAGAATATTCATGTTTATC
TRINITY_DN34_c0_g2_i1         CACTGGATATATTCCTACATGTACACTAGGTTGTACTTTATTAGAATATTCATGTTTATC
TRINITY_DN34_c0_g2_i4         CACTGGATATATTCCTACATGTACACTAGGTTGTACTTTATTAGAATATTCATGTTTATC
TRINITY_DN29_c0_g1_i2         CACTGGATATATTCCTACATGTACACTAGGTTGTACTTTATTAGAATATTCATGTTTATC
TRINITY_DN29_c0_g1_i3         CACTGGATATATTCCTACATGTACACTAGGTTGTACTTTATTAGAATATTCATGTTTATC
TRINITY_DN29_c0_g1_i4         CACTGGATATATTCCTACATGTACACTAGGTTGTACTTTATTAGAATATTCATGTTTATC
TRINITY_DN29_c0_g1_i1         CACTGGATATATTCCTACATGTACACTAGGTTGTACTTTATTAGAATATTCATGTTTATC
TRINITY_DN7_c0_g2_i1          CACTGGATATATTCCTACATGTACACTAGGTTGTACTTTATTAGAATATTCATGTTTATC
TRINITY_DN7_c0_g2_i4          CACTGGATATATTCCTACATGTACACTAGGTTGTACTTTATTAGAATATTCATGTTTATC
TRINITY_DN7_c0_g2_i3          CACTGGATATATTCCTACATGTACACTAGGTTGTACTTTATTAGAATATTCATGTTTATC
TRINITY_DN7_c0_g2_i2          CACTGGATATATTCCTACATGTACACTAGGTTGTACTTTATTAGAATATTCATGTTTATC
                              ************************************************************

MpDNV_mRNA_seq_consensus      GAACATACGAAAATATTGGCCTACGTCTATGTTAGTACAATATCTATCATTGCCGAATAT
TRINITY_DN34_c0_g2_i3         GAACATACGAAAATATTGGCCTACGTCTATGTTAGTACAATATCTATCATTGCCGAATAT
TRINITY_DN34_c0_g2_i2         GAACATACGAAAATATTGGCCTACGTCTATGTTAGTACAATATCTATCATTGCCGAATAT
TRINITY_DN34_c0_g2_i1         GAACATACGAAAATATTGGCCTACGTCTATGTTAGTACAATATCTATCATTGCCGAATAT
TRINITY_DN34_c0_g2_i4         GAACATACGAAAATATTGGCCTACGTCTATGTTAGTACAATATCTATCATTGCCGAATAT
TRINITY_DN29_c0_g1_i2         GAACATACGAAAATATTGGCCTACGTCTATGTTAGTACAATATCTATCATTGCCGAATAT
TRINITY_DN29_c0_g1_i3         GAACATACGAAAATATTGGCCTACGTCTATGTTAGTACAATATCTATCATTGCCGAATAT
TRINITY_DN29_c0_g1_i4         GAACATACGAAAATATTGGCCTACGTCTATGTTAGTACAATATCTATCATTGCCGAATAT
TRINITY_DN29_c0_g1_i1         GAACATACGAAAATATTGGCCTACGTCTATGTTAGTACAATATCTATCATTGCCGAATAT
TRINITY_DN7_c0_g2_i1          GAACATACGAAAATATTGGCCTACGTCTATGTTAGTACAATATCTATCATTGCCGAATAT
TRINITY_DN7_c0_g2_i4          GAACATACGAAAATATTGGCCTACGTCTATGTTAGTACAATATCTATCATTGCCGAATAT
TRINITY_DN7_c0_g2_i3          GAACATACGAAAATATTGGCCTACGTCTATGTTAGTACAATATCTATCATTGCCGAATAT
TRINITY_DN7_c0_g2_i2          GAACATACGAAAATATTGGCCTACGTCTATGTTAGTACAATATCTATCATTGCCGAATAT
                              ************************************************************

MpDNV_mRNA_seq_consensus      GCGTTTCCAACCGTTTTCACCCACAAATGTCAATTTTTGATCCGATCTCCTTTCGAACAA
TRINITY_DN34_c0_g2_i3         GCGTTTCCAACCGTTTTCACCCACAAATGTCAATTTTTGATCCGATCTCCTTTCGAACAA
TRINITY_DN34_c0_g2_i2         GCGTTTCCAACCGTTTTCACCCACAAATGTCAATTTTTGATCCGATCTCCTTTCGAACAA
TRINITY_DN34_c0_g2_i1         GCGTTTCCAACCGTTTTCACCCACAAATGTCAATTTTTGATCCGATCTCCTTTCGAACAA
TRINITY_DN34_c0_g2_i4         GCGTTTCCAACCGTTTTCACCCACAAATGTCAATTTTTGATCCGATCTCCTTTCGAACAA
TRINITY_DN29_c0_g1_i2         GCGTTTCCAACCGTTTTCACCCACAAATGTCAATTTTTGATCCGATCTCCTTTCGAACAA
TRINITY_DN29_c0_g1_i3         GCGTTTCCAACCGTTTTCACCCACAAATGTCAATTTTTGATCCGATCTCCTTTCGAACAA
TRINITY_DN29_c0_g1_i4         GCGTTTCCAACCGTTTTCACCCACAAATGTCAATTTTTGATCCGATCTCCTTTCGAACAA
TRINITY_DN29_c0_g1_i1         GCGTTTCCAACCGTTTTCACCCACAAATGTCAATTTTTGATCCGATCTCCTTTCGAACAA
TRINITY_DN7_c0_g2_i1          GCGTTTCCAACCGTTTTCACCCACAAATGTCAATTTTTGATCCGATCTCCTTTCGAACAA
```

```
TRINITY_DN7_c0_g2_i4          GCGTTTCCAACCGTTTTCACCCACAAATGTCAATTTTTGATCCGATCTCCTTTCGAACAA
TRINITY_DN7_c0_g2_i3          GCGTTTCCAACCGTTTTCACCCACAAATGTCAATTTTTGATCCGATCTCCTTTCGAACAA
TRINITY_DN7_c0_g2_i2          GCGTTTCCAACCGTTTTCACCCACAAATGTCAATTTTTGATCCGATCTCCTTTCGAACAA
                              ************************************************************

MpDNV_mRNA_seq_consensus      GCCACTGGCAGCGTTTATAAAATCTTGACACTCTGGTTCGTTACCAGCATTGCGTAACAT
TRINITY_DN34_c0_g2_i3         GCCACTGGCAGCGTTTATAAAATCTTGACACTCTGGTTCGTTACCAGCATTGCGTAACAT
TRINITY_DN34_c0_g2_i2         GCCACTGGCAGCGTTTATAAAATCTTGACACTCTGGTTCGTTACCAGCATTGCGTAACAT
TRINITY_DN34_c0_g2_i1         GCCACTGGCAGCGTTTATAAAATCTTGACACTCTGGTTCGTTACCAGCATTGCGTAACAT
TRINITY_DN34_c0_g2_i4         GCCACTGGCAGCGTTTATAAAATCTTGACACTCTGGTTCGTTACCAGCATTGCGTAACAT
TRINITY_DN29_c0_g1_i2         GCCACTGGCAGCGTTTATAAAATCTTGACACTCTGGTTCGTTACCAGCATTGCGTAACAT
TRINITY_DN29_c0_g1_i3         GCCACTGGCAGCGTTTATAAAATCTTGACACTCTGGTTCGTTACCAGCATTGCGTAACAT
TRINITY_DN29_c0_g1_i4         GCCACTGGCAGCGTTTATAAAATCTTGACACTCTGGTTCGTTACCAGCATTGCGTAACAT
TRINITY_DN29_c0_g1_i1         GCCACTGGCAGCGTTTATAAAATCTTGACACTCTGGTTCGTTACCAGCATTGCGTAACAT
TRINITY_DN7_c0_g2_i1          GCCACTGGCAGCGTTTATAAAATCTTGACACTCTGGTTCGTTACCAGCATTGCGTAACAT
TRINITY_DN7_c0_g2_i4          GCCACTGGCAGCGTTTATAAAATCTTGACACTCTGGTTCGTTACCAGCATTGCGTAACAT
TRINITY_DN7_c0_g2_i3          GCCACTGGCAGCGTTTATAAAATCTTGACACTCTGGTTCGTTACCAGCATTGCGTAACAT
TRINITY_DN7_c0_g2_i2          GCCACTGGCAGCGTTTATAAAATCTTGACACTCTGGTTCGTTACCAGCATTGCGTAACAT
                              ************************************************************

MpDNV_mRNA_seq_consensus      AGCAAATTTACTGGAATTAGGCTCCTGTACGCTGACACACGCATCGAATATTGGATCGTG
TRINITY_DN34_c0_g2_i3         AGCAAATTTACTGGAATTAGGCTCCTGTACGCTGACACACGCATCGAATATTGGATCGTG
TRINITY_DN34_c0_g2_i2         AGCAAATTTACTGGAATTAGGCTCCTGTACGCTGACACACGCATCGAATATTGGATCGTG
TRINITY_DN34_c0_g2_i1         AGCAAATTTACTGGAATTAGGCTCCTGTACGCTGACACACGCATCGAATATTGGATCGTG
TRINITY_DN34_c0_g2_i4         AGCAAATTTACTGGAATTAGGCTCCTGTACGCTGACACACGCATCGAATATTGGATCGTG
TRINITY_DN29_c0_g1_i2         AGCAAATTTACTGGAATTAGGCTCCTGTACGCTGACACACGCATCGAATATTGGATCGTG
TRINITY_DN29_c0_g1_i3         AGCAAATTTACTGGAATTAGGCTCCTGTACGCTGACACACGCATCGAATATTGGATCGTG
TRINITY_DN29_c0_g1_i4         AGCAAATTTACTGGAATTAGGCTCCTGTACGCTGACACACGCATCGAATATTGGATCGTG
TRINITY_DN29_c0_g1_i1         AGCAAATTTACTGGAATTAGGCTCCTGTACGCTGACACACGCATCGAATATTGGATCGTG
TRINITY_DN7_c0_g2_i1          AGCAAATTTACTGGAATTAGGCTCCTGTACGCTGACACACGCATCGAATATTGGATCGTG
TRINITY_DN7_c0_g2_i4          AGCAAATTTACTGGAATTAGGCTCCTGTACGCTGACACACGCATCGAATATTGGATCGTG
TRINITY_DN7_c0_g2_i3          AGCAAATTTACTGGAATTAGGCTCCTGTACGCTGACACACGCATCGAATATTGGATCGTG
TRINITY_DN7_c0_g2_i2          AGCAAATTTACTGGAATTAGGCTCCTGTACGCTGACACACGCATCGAATATTGGATCGTG
                              ************************************************************

MpDNV_mRNA_seq_consensus      TGGTAGAGTGAGTATACCGACATGAGGCTTGTACGAATAATTAAGTACAGTGGTGCCGAC
TRINITY_DN34_c0_g2_i3         TGGTAGAGTGAGTATACCGACATGAGGCTTGTACGAATAATTAAGTACAGTGGTGCCGAC
TRINITY_DN34_c0_g2_i2         TGGTAGAGTGAGTATACCGACATGAGGCTTGTACGAATAATTAAGTACAGTGGTGCCGAC
TRINITY_DN34_c0_g2_i1         TGGTAGAGTGAGTATACCGACATGAGGCTTGTACGAATAATTAAGTACAGTGGTGCCGAC
TRINITY_DN34_c0_g2_i4         TGGTAGAGTGAGTATACCGACATGAGGCTTGTACGAATAATTAAGTACAGTGGTGCCGAC
TRINITY_DN29_c0_g1_i2         TGGTAGAGTGAGTATACCGACATGAGGCTTGTACGAATAATTAAGTACAGTGGTGCCGAC
TRINITY_DN29_c0_g1_i3         TGGTAGAGTGAGTATACCGACATGAGGCTTGTACGAATAATTAAGTACAGTGGTGCCGAC
TRINITY_DN29_c0_g1_i4         TGGTAGAGTGAGTATACCGACATGAGGCTTGTACGAATAATTAAGTACAGTGGTGCCGAC
TRINITY_DN29_c0_g1_i1         TGGTAGAGTGAGTATACCGACATGAGGCTTGTACGAATAATTAAGTACAGTGGTGCCGAC
TRINITY_DN7_c0_g2_i1          TGGTAGAGTGAGTATACCGACATGAGGCTTGTACGAATAATTAAGTACAGTGGTGCCGAC
TRINITY_DN7_c0_g2_i4          TGGTAGAGTGAGTATACCGACATGAGGCTTGTACGAATAATTAAGTACAGTGGTGCCGAC
TRINITY_DN7_c0_g2_i3          TGGTAGAGTGAGTATACCGACATGAGGCTTGTACGAATAATTAAGTACAGTGGTGCCGAC
TRINITY_DN7_c0_g2_i2          TGGTAGAGTGAGTATACCGACATGAGGCTTGTACGAATAATTAAGTACAGTGGTGCCGAC
                              ************************************************************

MpDNV_mRNA_seq_consensus      AGTAAACGAAGCGTCGACCTTGGTGATGTGTTCATTAAACCGTTCCCATCCGAGTTTTCC
TRINITY_DN34_c0_g2_i3         AGTAAACGAAGCGTCGACCTTGGTGATGTGTTCATTAAACCGTTCCCATCCGAGTTTTCC
TRINITY_DN34_c0_g2_i2         AGTAAACGAAGCGTCGACCTTGGTGATGTGTTCATTAAACCGTTCCCATCCGAGTTTTCC
TRINITY_DN34_c0_g2_i1         AGTAAACGAAGCGTCGACCTTGGTGATGTGTTCATTAAACCGTTCCCATCCGAGTTTTCC
TRINITY_DN34_c0_g2_i4         AGTAAACGAAGCGTCGACCTTGGTGATGTGTTCATTAAACCGTTCCCATCCGAGTTTTCC
TRINITY_DN29_c0_g1_i2         AGTAAACGAAGCGTCGACCTTGGTGATGTGTTCATTAAACCGTTCCCATCCGAGTTTTCC
TRINITY_DN29_c0_g1_i3         AGTAAACGAAGCGTCGACCTTGGTGATGTGTTCATTAAACCGTTCCCATCCGAGTTTTCC
TRINITY_DN29_c0_g1_i4         AGTAAACGAAGCGTCGACCTTGGTGATGTGTTCATTAAACCGTTCCCATCCGAGTTTTCC
TRINITY_DN29_c0_g1_i1         AGTAAACGAAGCGTCGACCTTGGTGATGTGTTCATTAAACCGTTCCCATCCGAGTTTTCC
TRINITY_DN7_c0_g2_i1          AGTAAACGAAGCGTCGACCTTGGTGATGTGTTCATTAAACCGTTCCCATCCGAGTTTTCC
TRINITY_DN7_c0_g2_i4          AGTAAACGAAGCGTCGACCTTGGTGATGTGTTCATTAAACCGTTCCCATCCGAGTTTTCC
TRINITY_DN7_c0_g2_i3          AGTAAACGAAGCGTCGACCTTGGTGATGTGTTCATTAAACCGTTCCCATCCGAGTTTTCC
TRINITY_DN7_c0_g2_i2          AGTAAACGAAGCGTCGACCTTGGTGATGTGTTCATTAAACCGTTCCCATCCGAGTTTTCC
                              ************************************************************

MpDNV_mRNA_seq_consensus      AGAATTATGATTTCTATTTACAAAATTACAAAAATAACTGTTATACATCAACGGTAAGTT
TRINITY_DN34_c0_g2_i3         AGAATTATGATTTCTATTTACAAAATTACAAAAATAACTGTTATACATCAACGGTAAGTT
TRINITY_DN34_c0_g2_i2         AGAATTATGATTTCTATTTACAAAATTACAAAAATAACTGTTATACATCAACGGTAAGTT
TRINITY_DN34_c0_g2_i1         AGAATTATGATTTCTATTTACAAAATTACAAAAATAACTGTTATACATCAACGGTAAGTT
TRINITY_DN34_c0_g2_i4         AGAATTATGATTTCTATTTACAAAATTACAAAAATAACTGTTATACATCAACGGTAAGTT
TRINITY_DN29_c0_g1_i2         AGAATTATGATTTCTATTTACAAAATTACAAAAATAACTGTTATACATCAACGGTAAGTT
TRINITY_DN29_c0_g1_i3         AGAATTATGATTTCTATTTACAAAATTACAAAAATAACTGTTATACATCAACGGTAAGTT
TRINITY_DN29_c0_g1_i4         AGAATTATGATTTCTATTTACAAAATTACAAAAATAACTGTTATACATCAACGGTAAGTT
TRINITY_DN29_c0_g1_i1         AGAATTATGATTTCTATTTACAAAATTACAAAAATAACTGTTATACATCAACGGTAAGTT
TRINITY_DN7_c0_g2_i1          AGAATTATGATTTCTATTTACAAAATTACAAAAATAACTGTTATACATCAACGGTAAGTT
TRINITY_DN7_c0_g2_i4          AGAATTATGATTTCTATTTACAAAATTACAAAAATAACTGTTATACATCAACGGTAAGTT
TRINITY_DN7_c0_g2_i3          AGAATTATGATTTCTATTTACAAAATTACAAAAATAACTGTTATACATCAACGGTAAGTT
TRINITY_DN7_c0_g2_i2          AGAATTATGATTTCTATTTACAAAATTACAAAAATAACTGTTATACATCAACGGTAAGTT
                              ************************************************************

MpDNV_mRNA_seq_consensus      CATGTAGGACGCAGGCAGTTCATATCCCTCTTTAGCAGAGAATGTACCATCTAGATCTTG
TRINITY_DN34_c0_g2_i3         CATGTAGGACGCAGGCAGTTCATATCCCTCTTTAGCAGAGAATGTACCATCTAGATCTTG
TRINITY_DN34_c0_g2_i2         CATGTAGGACGCAGGCAGTTCATATCCCTCTTTAGCAGAGAATGTACCATCTAGATCTTG
TRINITY_DN34_c0_g2_i1         CATGTAGGACGCAGGCAGTTCATATCCCTCTTTAGCAGAGAATGTACCATCTAGATCTTG
TRINITY_DN34_c0_g2_i4         CATGTAGGACGCAGGCAGTTCATATCCCTCTTTAGCAGAGAATGTACCATCTAGATCTTG
TRINITY_DN29_c0_g1_i2         CATGTAGGACGCAGGCAGTTCATATCCCTCTTTAGCAGAGAACGTACCATCTAGATCTTG
TRINITY_DN29_c0_g1_i3         CATGTAGGACGCAGGCAGTTCATATCCCTCTTTAGCAGAGAACGTACCATCTAGATCTTG
TRINITY_DN29_c0_g1_i4         CATGTAGGACGCAGGCAGTTCATATCCCTCTTTAGCAGAGAACGTACCATCTAGATCTTG
```

```
TRINITY_DN29_c0_g1_i1        CATGTAGGACGCAGGCAGTTCATATCCCTCTTTAGCAGAGAACGTACCATCTAGATCTTG
TRINITY_DN7_c0_g2_i1         CATGTAGGACGCAGGCAGTTCATATCCCTCTTTAGCAGAGAATGTACCATCTAGATCTTG
TRINITY_DN7_c0_g2_i4         CATGTAGGACGCAGGCAGTTCATATCCCTCTTTAGCAGAGAATGTACCATCTAGATCTTG
TRINITY_DN7_c0_g2_i3         CATGTAGGACGCAGGCAGTTCATATCCCTCTTTAGCAGAGAATGTACCATCTAGATCTTG
TRINITY_DN7_c0_g2_i2         CATGTAGGACGCAGGCAGTTCATATCCCTCTTTAGCAGAGAATGTACCATCTAGATCTTG
                             ****************************************** *****************

MpDNV_mRNA_seq_consensus     ACCGTACAATTTGCGAACGAATTCTTTCATAGACGACTGATCGTTCTCGGTATTCGTTGG
TRINITY_DN34_c0_g2_i3        ACCGTACAATTTGCGAACGAATTCTTTCATAGACGACTGATCGTTCTCGGTATTCGTTGG
TRINITY_DN34_c0_g2_i2        ACCGTACAATTTGCGAACGAATTCTTTCATAGACGACTGATCGTTCTCGGTATTCGTTGG
TRINITY_DN34_c0_g2_i1        ACCGTACAATTTGCGAACGAATTCTTTCATAGACGACTGATCGTTCTCGGTATTCGTTGG
TRINITY_DN34_c0_g2_i4        ACCGTACAATTTGCGAACGAATTCTTTCATAGACGACTGATCGTTCTCGGTATTCGTTGG
TRINITY_DN29_c0_g1_i2        ACCGTACAATTTGCGAACGAATTCTTTCATAGACGACTGATCGTTCTCGGTATTCGTTGG
TRINITY_DN29_c0_g1_i3        ACCGTACAATTTGCGAACGAATTCTTTCATAGACGACTGATCGTTCTCGGTATTCGTTGG
TRINITY_DN29_c0_g1_i4        ACCGTACAATTTGCGAACGAATTCTTTCATAGACGACTGATCGTTCTCGGTATTCGTTGG
TRINITY_DN29_c0_g1_i1        ACCGTACAATTTGCGAACGAATTCTTTCATAGACGACTGATCGTTCTCGGTATTCGTTGG
TRINITY_DN7_c0_g2_i1         ACCGTACAATTTGCGAACGAATTCTTTCATAGACGACTGATCGTTCTCGGTATTCGTTGG
TRINITY_DN7_c0_g2_i4         ACCGTACAATTTGCGAACGAATTCTTTCATAGACGACTGATCGTTCTCGGTATTCGTTGG
TRINITY_DN7_c0_g2_i3         ACCGTACAATTTGCGAACGAATTCTTTCATAGACGACTGATCGTTCTCGGTATTCGTTGG
TRINITY_DN7_c0_g2_i2         ACCGTACAATTTGCGAACGAATTCTTTCATAGACGACTGATCGTTCTCGGTATTCGTTGG
                             ************************************************************

MpDNV_mRNA_seq_consensus     ATCCATGGGCTCCGATGAATTGCCAAATGTGAGTTTTCGGTTAAATCCCCTTGTTTTTAA
TRINITY_DN34_c0_g2_i3        ATCCATGGGCTCCGATGAATTGCCAAATGTGAGTTTTCGGTTAAATCCCCTTGTTTTTAA
TRINITY_DN34_c0_g2_i2        ATCCATGGGCTCCGATGAATTGCCAAATGTGAGTTTTCGGTTAAATCCCCTTGTTTTTAA
TRINITY_DN34_c0_g2_i1        ATCCATGGGCTCCGATGAATTGCCAAATGTGAGTTTTCGGTTAAATCCCCTTGTTTTTAA
TRINITY_DN34_c0_g2_i4        ATCCATGGGCTCCGATGAATTGCCAAATGTGAGTTTTCGGTTAAATCCCCTTGTTTTTAA
TRINITY_DN29_c0_g1_i2        ATCCATGGGCTCCGATGAATTGCCAAATGTGAGTTTTCGGTTAAATCCCCTTGTTTTTAA
TRINITY_DN29_c0_g1_i3        ATCCATGGGCTCCGATGAATTGCCAAATGTGAGTTTTCGGTTAAATCCCCTTGTTTTTAA
TRINITY_DN29_c0_g1_i4        ATCCATGGGCTCCGATGAATTGCCAAATGTGAGTTTTCGGTTAAATCCCCTTGTTTTTAA
TRINITY_DN29_c0_g1_i1        ATCCATGGGCTCCGATGAATTGCCAAATGTGAGTTTTCGGTTAAATCCCCTTGTTTTTAA
TRINITY_DN7_c0_g2_i1         ATCCATGGGCTCCGATGAATTGCCAAATGTGAGTTTTCGGTTAAATCCCCTTGTTTTTAA
TRINITY_DN7_c0_g2_i4         ATCCATGGGCTCCGATGAATTGCCAAATGTGAGTTTTCGGTTAAATCCCCTTGTTTTTAA
TRINITY_DN7_c0_g2_i3         ATCCATGGGCTCCGATGAATTGCCAAATGTGAGTTTTCGGTTAAATCCCCTTGTTTTTAA
TRINITY_DN7_c0_g2_i2         ATCCATGGGCTCCGATGAATTGCCAAATGTGAGTTTTCGGTTAAATCCCCTTGTTTTTAA
                             ************************************************************

MpDNV_mRNA_seq_consensus     ATTCAAAGCGTCCGCTTTTACCATAAATTTGTTCTGATTCAATGTGGCCAGGGAGCTCGT
TRINITY_DN34_c0_g2_i3        ATTCAAAGCGTCCGCTTTTACCATAAATTTGTTCTGATTCAATGTGGCCAGGGAGCTCGT
TRINITY_DN34_c0_g2_i2        ATTCAAAGCGTCCGCTTTTACCATAAATTTGTTCTGATTCAATGTGGCCAGGGAGCTCGT
TRINITY_DN34_c0_g2_i1        ATTCAAAGCGTCCGCTTTTACCATAAATTTGTTCTGATTCAATGTGGCCAGGGAGCTCGT
TRINITY_DN34_c0_g2_i4        ATTCAAAGCGTCCGCTTTTACCATAAATTTGTTCTGATTCAATGTGGCCAGGGAGCTCGT
TRINITY_DN29_c0_g1_i2        ATTCAAAGCGTCCGCTTTTACCATAAATTTGTTCTGATTCAATGTGGCCAGGGAGCTCGT
TRINITY_DN29_c0_g1_i3        ATTCAAAGCGTCCGCTTTTACCATAAATTTGTTCTGATTCAATGTGGCCAGGGAGCTCGT
TRINITY_DN29_c0_g1_i4        ATTCAAAGCGTCCGCTTTTACCATAAATTTGTTCTGATTCAATGTGGCCAGGGAGCTCGT
TRINITY_DN29_c0_g1_i1        ATTCAAAGCGTCCGCTTTTACCATAAATTTGTTCTGATTCAATGTGGCCAGGGAGCTCGT
TRINITY_DN7_c0_g2_i1         ATTCAAAGCGTCCGCTTTTACCATAAATTTGTTCTGATTCAATGTGGCCAGGGAGCTCGT
TRINITY_DN7_c0_g2_i4         ATTCAAAGCGTCCGCTTTTACCATAAATTTGTTCTGATTCAATGTGGCCAGGGAGCTCGT
TRINITY_DN7_c0_g2_i3         ATTCAAAGCGTCCGCTTTTACCATAAATTTGTTCTGATTCAATGTGGCCAGGGAGCTCGT
TRINITY_DN7_c0_g2_i2         ATTCAAAGCGTCCGCTTTTACCATAAATTTGTTCTGATTCAATGTGGCCAGGGAGCTCGT
                             ************************************************************

MpDNV_mRNA_seq_consensus     AGAGCTATTGGTTTCAAAAGCTGTGCGAGGATTACGCATGACACCCTTGCATTTGACTTC
TRINITY_DN34_c0_g2_i3        AGAGCTATTGGTTTCAAAAGCTGTGCGAGGATTACGCATGACACCCTTGCATTTGACTTC
TRINITY_DN34_c0_g2_i2        AGAGCTATTGGTTTCAAAAGCTGTGCGAGGATTACGCATGACACCCTTGCATTTGACTTC
TRINITY_DN34_c0_g2_i1        AGAGCTATTGGTTTCAAAAGCTGTGCGAGGATTACGCATGACACCCTTGCATTTGACTTC
TRINITY_DN34_c0_g2_i4        AGAGCTATTGGTTTCAAAAGCTGTGCGAGGATTACGCATGACACCCTTGCATTTGACTTC
TRINITY_DN29_c0_g1_i2        AGAGCTATTGGTTTCAAAAGCTGTGCGAGGATTACGCATGACACCCTTGCATTTGACTTC
TRINITY_DN29_c0_g1_i3        AGAGCTATTGGTTTCAAAAGCTGTGCGAGGATTACGCATGACACCCTTGCATTTGACTTC
TRINITY_DN29_c0_g1_i4        AGAGCTATTGGTTTCAAAAGCTGTGCGAGGATTACGCATGACACCCTTGCATTTGACTTC
TRINITY_DN29_c0_g1_i1        AGAGCTATTGGTTTCAAAAGCTGTGCGAGGATTACGCATGACACCCTTGCATTTGACTTC
TRINITY_DN7_c0_g2_i1         AGAGCTATTGGTTTCAAAAGCTGTGCGAGGATTACGCATGACACCCTTGCATTTGACTTC
TRINITY_DN7_c0_g2_i4         AGAGCTATTGGTTTCAAAAGCTGTGCGAGGATTACGCATGACACCCTTGCATTTGACTTC
TRINITY_DN7_c0_g2_i3         AGAGCTATTGGTTTCAAAAGCTGTGCGAGGATTACGCATGACACCCTTGCATTTGACTTC
TRINITY_DN7_c0_g2_i2         AGAGCTATTGGTTTCAAAAGCTGTGCGAGGATTACGCATGACACCCTTGCATTTGACTTC
                             ************************************************************

MpDNV_mRNA_seq_consensus     AGTGCATATAGCACCTCTGGGTAAAAATTTAAACTCGGACGGCGACATATAGAACCAAGG
TRINITY_DN34_c0_g2_i3        AGTGCATATAGCACCTCTGGGTAAAAATTTAAACTCGGACGGCGACATATAGAACCAAGG
TRINITY_DN34_c0_g2_i2        AGTGCATATAGCACCTCTGGGTAAAAATTTAAACTCGGACGGCGACATATAGAACCAAGG
TRINITY_DN34_c0_g2_i1        AGTGCATATAGCACCTCTGGGTAAAAATTTAAACTCGGACGGCGACATATAGAACCAAGG
TRINITY_DN34_c0_g2_i4        AGTGCATATAGCACCTCTGGGTAAAAATTTAAACTCGGACGGCGACATATAGAACCAAGG
TRINITY_DN29_c0_g1_i2        AGTGCATATAGCACCTCTGGGTAAAAATTTAAACTCGGACGGCGACATATAGAACCAAGG
TRINITY_DN29_c0_g1_i3        AGTGCATATAGCACCTCTGGGTAAAAATTTAAACTCGGACGGCGACATATAGAACCAAGG
TRINITY_DN29_c0_g1_i4        AGTGCATATAGCACCTCTGGGTAAAAATTTAAACTCGGACGGCGACATATAGAACCAAGG
TRINITY_DN29_c0_g1_i1        AGTGCATATAGCACCTCTGGGTAAAAATTTAAACTCGGACGGCGACATATAGAACCAAGG
TRINITY_DN7_c0_g2_i1         AGTGCATATAGCACCTCTGGGTAAAAATTTAAACTCGGACGGCGACATATAGAACCAAGG
TRINITY_DN7_c0_g2_i4         AGTGCATATAGCACCTCTGGGTAAAAATTTAAACTCGGACGGCGACATATAGAACCAAGG
TRINITY_DN7_c0_g2_i3         AGTGCATATAGCACCTCTGGGTAAAAATTTAAACTCGGACGGCGACATATAGAACCAAGG
TRINITY_DN7_c0_g2_i2         AGTGCATATAGCACCTCTGGGTAAAAATTTAAACTCGGACGGCGACATATAGAACCAAGG
                             ************************************************************

MpDNV_mRNA_seq_consensus     TTGGTCCACGGGCACACTCATTAAACTCGTTGTCCCAAGATAGGCGTTATCAGTGCTAAG
TRINITY_DN34_c0_g2_i3        TTGGTCCACGGGCACACTCATTAAACTCGTTGTCCCAAGATAGGCGTTATCAGTGCTAAG
TRINITY_DN34_c0_g2_i2        TTGGTCCACGGGCACACTCATTAAACTCGTTGTCCCAAGATAGGCGTTATCAGTGCTAAG
TRINITY_DN34_c0_g2_i1        TTGGTCCACGGGCACACTCATTAAACTCGTTGTCCCAAGATAGGCGTTATCAGTGCTAAG
TRINITY_DN34_c0_g2_i4        TTGGTCCACGGGCACACTCATTAAACTCGTTGTCCCAAGATAGGCGTTATCAGTGCTAAG
TRINITY_DN29_c0_g1_i2        TTGGTCCACGGGCACACTCATTAAACTCGTTGTCCCAAGATAGGCGTTATCAGTGCTAAG
```

```
TRINITY_DN29_c0_g1_i3        TTGGTCCACGGGCACACTCATTAAACTCGTTGTCCCAAGATAGGCGTTATCAGTGCTAAG
TRINITY_DN29_c0_g1_i4        TTGGTCCACGGGCACACTCATTAAACTCGTTGTCCCAAGATAGGCGTTATCAGTGCTAAG
TRINITY_DN29_c0_g1_i1        TTGGTCCACGGGCACACTCATTAAACTCGTTGTCCCAAGATAGGCGTTATCAGTGCTAAG
TRINITY_DN7_c0_g2_i1         TTGGTCCACGGGCACACTCATTAAACTCGTTGTCCCAAGATAGGCGTTATCAGTGCTAAG
TRINITY_DN7_c0_g2_i4         TTGGTCCACGGGCACACTCATTAAACTCGTTGTCCCAAGATAGGCGTTATCAGTGCTAAG
TRINITY_DN7_c0_g2_i3         TTGGTCCACGGGCACACTCATTAAACTCGTTGTCCCAAGATAGGCGTTATCAGTGCTAAG
TRINITY_DN7_c0_g2_i2         TTGGTCCACGGGCACACTCATTAAACTCGTTGTCCCAAGATAGGCGTTATCAGTGCTAAG
                             ************************************************************

MpDNV_mRNA_seq_consensus     ATATCGCAACACTTTCCAACCACATCCGTAACTGATGAAAGTATGTACTTTTCGGTAAAC
TRINITY_DN34_c0_g2_i3        ATATCGCAACACTTTCCAACCACATCCGTAACTGATGAAAGTATGTACTTTTCGGTAAAC
TRINITY_DN34_c0_g2_i2        ATATCGCAACACTTTCCAACCACATCCGTAACTGATGAAAGTATGTACTTTTCGGTAAAC
TRINITY_DN34_c0_g2_i1        ATATCGCAACACTTTCCAACCACATCCGTAACTGATGAAAGTATGTACTTTTCGGTAAAC
TRINITY_DN34_c0_g2_i4        ATATCGCAACACTTTCCAACCACATCCGTAACTGATGAAAGTATGTACTTTTCGGTAAAC
TRINITY_DN29_c0_g1_i2        ATATCGCAACACTTTCCAACCACATCCGTAACTGATGAAAGTATGTACTTTTCGGTAAAC
TRINITY_DN29_c0_g1_i3        ATATCGCAACACTTTCCAACCACATCCGTAACTGATGAAAGTATGTACTTTTCGGTAAAC
TRINITY_DN29_c0_g1_i4        ATATCGCAACACTTTCCAACCACATCCGTAACTGATGAAAGTATGTACTTTTCGGTAAAC
TRINITY_DN29_c0_g1_i1        ATATCGCAACACTTTCCAACCACATCCGTAACTGATGAAAGTATGTACTTTTCGGTAAAC
TRINITY_DN7_c0_g2_i1         ATATCGCAACACTTTCCAACCACATCCGTAACTGATGAAAGTATGTACTTTTCGGTAAAC
TRINITY_DN7_c0_g2_i4         ATATCGCAACACTTTCCAACCACATCCGTAACTGATGAAAGTATGTACTTTTCGGTAAAC
TRINITY_DN7_c0_g2_i3         ATATCGCAACACTTTCCAACCACATCCGTAACTGATGAAAGTATGTACTTTTCGGTAAAC
TRINITY_DN7_c0_g2_i2         ATATCGCAACACTTTCCAACCACATCCGTAACTGATGAAAGTATGTACTTTTCGGTAAAC
                             ************************************************************

MpDNV_mRNA_seq_consensus     CATAGTATATCCTCCCGTGTGACCGATTGGCCTAGGTATAACAGCGTTTTCAGAGCTAGG
TRINITY_DN34_c0_g2_i3        CATAGTATATCCTCCCGTGTGACCGATTGGCCTAGGTATAACAGCGTTTTCAGAGCTAGG
TRINITY_DN34_c0_g2_i2        CATAGTATATCCTCCCGTGTGACCGATTGGCCTAGGTATAACAGCGTTTTCAGAGCTAGG
TRINITY_DN34_c0_g2_i1        CATAGTATATCCTCCCGTGTGACCGATTGGCCTAGGTATAACAGCGTTTTCAGAGCTAGG
TRINITY_DN34_c0_g2_i4        CATAGTATATCCTCCCGTGTGACCGATTGGCCTAGGTATAACAGCGTTTTCAGAGCTAGG
TRINITY_DN29_c0_g1_i2        CATAGTATATCCTCCCGTGTGACCGATTGGCCTAGGTATAACAGCGTTTTCAGAGCTAGG
TRINITY_DN29_c0_g1_i3        CATAGTATATCCTCCCGTGTGACCGATTGGCCTAGGTATAACAGCGTTTTCAGAGCTAGG
TRINITY_DN29_c0_g1_i4        CATAGTATATCCTCCCGTGTGACCGATTGGCCTAGGTATAACAGCGTTTTCAGAGCTAGG
TRINITY_DN29_c0_g1_i1        CATAGTATATCCTCCCGTGTGACCGATTGGCCTAGGTATAACAGCGTTTTCAGAGCTAGG
TRINITY_DN7_c0_g2_i1         CATAGTATATCCTCCCGTGTGACCGATTGGCCTAGGTATAACAGCGTTTTCAGAGCTAGG
TRINITY_DN7_c0_g2_i4         CATAGTATATCCTCCCGTGTGACCGATTGGCCTAGGTATAACAGCGTTTTCAGAGCTAGG
TRINITY_DN7_c0_g2_i3         CATAGTATATCCTCCCGTGTGACCGATTGGCCTAGGTATAACAGCGTTTTCAGAGCTAGG
TRINITY_DN7_c0_g2_i2         CATAGTATATCCTCCCGTGTGACCGATTGGCCTAGGTATAACAGCGTTTTCAGAGCTAGG
                             ************************************************************

MpDNV_mRNA_seq_consensus     ATTGCCAGTATCTGGGTCCCCATCGTTCGCAGCAGCCGTCCCAGGAAGCGAGGTGCCACC
TRINITY_DN34_c0_g2_i3        ATTGCCAGTATCTGGGTCCCCATCGTTCGCAGCAGCCGTCCCAGGAAGCGAGGTGCCACC
TRINITY_DN34_c0_g2_i2        ATTGCCAGTATCTGGGTCCCCATCGTTCGCAGCAGCCGTCCCAGGAAGCGAGGTGCCACC
TRINITY_DN34_c0_g2_i1        ATTGCCAGTATCTGGGTCCCCATCGTTCGCAGCAGCCGTCCCAGGAAGCGAGGTGCCACC
TRINITY_DN34_c0_g2_i4        ATTGCCAGTATCTGGGTCCCCATCGTTCGCAGCAGCCGTCCCAGGAAGCGAGGTGCCACC
TRINITY_DN29_c0_g1_i2        ATTGCCAGTATCTGGGTCCCCATCGTTCGCAGCAGCCGTCCCAGGAAGCGAGGTGCCACC
TRINITY_DN29_c0_g1_i3        ATTGCCAGTATCTGGGTCCCCATCGTTCGCAGCAGCCGTCCCAGGAAGCGAGGTGCCACC
TRINITY_DN29_c0_g1_i4        ATTGCCAGTATCTGGGTCCCCATCGTTCGCAGCAGCCGTCCCAGGAAGCGAGGTGCCACC
TRINITY_DN29_c0_g1_i1        ATTGCCAGTATCTGGGTCCCCATCGTTCGCAGCAGCCGTCCCAGGAAGCGAGGTGCCACC
TRINITY_DN7_c0_g2_i1         ATTGCCAGTATCTGGGTCCCCATCGTTCGCAGCAGCCGTCCCAGGAAGCGAGGTGCCACC
TRINITY_DN7_c0_g2_i4         ATTGCCAGTATCTGGGTCCCCATCGTTCGCAGCAGCCGTCCCAGGAAGCGAGGTGCCACC
TRINITY_DN7_c0_g2_i3         ATTGCCAGTATCTGGGTCCCCATCGTTCGCAGCAGCCGTCCCAGGAAGCGAGGTGCCACC
TRINITY_DN7_c0_g2_i2         ATTGCCAGTATCTGGGTCCCCATCGTTCGCAGCAGCCGTCCCAGGAAGCGAGGTGCCACC
                             ************************************************************

MpDNV_mRNA_seq_consensus     CGCACGTTTAGGAGCGGGTTCAGAACCGGAGCTATCGGCAGGTCGTTTGCTACTTGATCC
TRINITY_DN34_c0_g2_i3        CGCACGTTTAGGAGCGGGTTCAGAACCGGAGCTATCGGCAGGTCGTTTGCTACTTGATCC
TRINITY_DN34_c0_g2_i2        CGCACGTTTAGGAGCGGGTTCAGAACCGGAGCTATCGGCAGGTCGTTTGCTACTTGATCC
TRINITY_DN34_c0_g2_i1        CGCACGTTTAGGAGCGGGTTCAGAACCGGAGCTATCGGCAGGTCGTTTGCTACTTGATCC
TRINITY_DN34_c0_g2_i4        CGCACGTTTAGGAGCGGGTTCAGAACCGGAGCTATCGGCAGGTCGTTTGCTACTTGATCC
TRINITY_DN29_c0_g1_i2        CGCACGTTTAGGAGCGGGTTCAGAACCGGAGCTATCGGCAGGTCGTTTGCTACTTGATCC
TRINITY_DN29_c0_g1_i3        CGCACGTTTAGGAGCGGGTTCAGAACCGGAGCTATCGGCAGGTCGTTTGCTACTTGATCC
TRINITY_DN29_c0_g1_i4        CGCACGTTTAGGAGCGGGTTCAGAACCGGAGCTATCGGCAGGTCGTTTGCTACTTGATCC
TRINITY_DN29_c0_g1_i1        CGCACGTTTAGGAGCGGGTTCAGAACCGGAGCTATCGGCAGGTCGTTTGCTACTTGATCC
TRINITY_DN7_c0_g2_i1         CGCACGTTTAGGAGCGGGTTCAGAACCGGAGCTATCGGCAGGTCGTTTGCTACTTGATCC
TRINITY_DN7_c0_g2_i4         CGCACGTTTAGGAGCGGGTTCAGAACCGGAGCTATCGGCAGGTCGTTTGCTACTTGATCC
TRINITY_DN7_c0_g2_i3         CGCACGTTTAGGAGCGGGTTCAGAACCGGAGCTATCGGCAGGTCGTTTGCTACTTGATCC
TRINITY_DN7_c0_g2_i2         CGCACGTTTAGGAGCGGGTTCAGAACCGGAGCTATCGGCAGGTCGTTTGCTACTTGATCC
                             ************************************************************

MpDNV_mRNA_seq_consensus     GCTAGAGCTTGCCATGGATCCCGATGCGCTTCCCCCGTTGCCGTTGCTAGTGCTGTTGTC
TRINITY_DN34_c0_g2_i3        GCTAGAGCTTGCCATGGATCCCGATGCGCTTCCCCCGTTGCCGTTGCTAGTGCTGTTGTC
TRINITY_DN34_c0_g2_i2        GCTAGAGCTTGCCATGGATCCCGATGCGCTTCCCCCGTTGCCGTTGCTAGTGCTGTTGTC
TRINITY_DN34_c0_g2_i1        GCTAGAGCTTGCCATGGATCCCGATGCGCTTCCCCCGTTGCCGTTGCTAGTGCTGTTGTC
TRINITY_DN34_c0_g2_i4        GCTAGAGCTTGCCATGGATCCCGATGCGCTTCCCCCGTTGCCGTTGCTAGTGCTGTTGTC
TRINITY_DN29_c0_g1_i2        GCTAGAGCTTGCCATGGATCCCGATGCGCTTCCCCCGTTGCCGTTGCTAGTGCTGTTGTC
TRINITY_DN29_c0_g1_i3        GCTAGAGCTTGCCATGGATCCCGATGCGCTTCCCCCGTTGCCGTTGCTAGTGCTGTTGTC
TRINITY_DN29_c0_g1_i4        GCTAGAGCTTGCCATGGATCCCGATGCGCTTCCCCCGTTGCCGTTGCTAGTGCTGTTGTC
TRINITY_DN29_c0_g1_i1        GCTAGAGCTTGCCATGGATCCCGATGCGCTTCCCCCGTTGCCGTTGCTAGTGCTGTTGTC
TRINITY_DN7_c0_g2_i1         GCTAGAGCTTGCCATGGATCCCGATGCGCTTCCCCCGTTGCCGTTGCTAGTGCTGTTGTC
TRINITY_DN7_c0_g2_i4         GCTAGAGCTTGCCATGGATCCCGATGCGCTTCCCCCGTTGCCGTTGCTAGTGCTGTTGTC
TRINITY_DN7_c0_g2_i3         GCTAGAGCTTGCCATGGATCCCGATGCGCTTCCCCCGTTGCCGTTGCTAGTGCTGTTGTC
TRINITY_DN7_c0_g2_i2         GCTAGAGCTTGCCATGGATCCCGATGCGCTTCCCCCGTTGCCGTTGCTAGTGCTGTTGTC
                             ************************************************************

MpDNV_mRNA_seq_consensus     CGGGGCTGAGGTGTTTGCCACGTGGTCGCCACTCGCTTCACCGCTGTTAGGATTGCCGTT
TRINITY_DN34_c0_g2_i3        CGGGGCTGAGGTGTTTGCCACGTGGTCGCCACTCGCTTCACCGCTGTTAGGATTGCCGTT
TRINITY_DN34_c0_g2_i2        CGGGGCTGAGGTGTTTGCCACGTGGTCGCCACTCGCTTCACCGCTGTTAGGATTGCCGTT
TRINITY_DN34_c0_g2_i1        CGGGGCTGAGGTGTTTGCCACGTGGTCGCCACTCGCTTCACCGCTGTTAGGATTGCCGTT
```

```
TRINITY_DN34_c0_g2_i4          CGGGGCTGAGGTGTTTGCCACGTGGTCGCCACTCGCTTCACCGCTGTTAGGATTGCCGTT
TRINITY_DN29_c0_g1_i2          CGGGGCTGAGGTGTTTGCCACGTGGTCGCCACTCGCTTCACCGCTGTTAGGATTGCCGTT
TRINITY_DN29_c0_g1_i3          CGGGGCTGAGGTGTTTGCCACGTGGTCGCCACTCGCTTCACCGCTGTTAGGATTGCCGTT
TRINITY_DN29_c0_g1_i4          CGGGGCTGAGGTGTTTGCCACGTGGTCGCCACTCGCTTCACCGCTGTTAGGATTGCCGTT
TRINITY_DN29_c0_g1_i1          CGGGGCTGAGGTGTTTGCCACGTGGTCGCCACTCGCTTCACCGCTGTTAGGATTGCCGTT
TRINITY_DN7_c0_g2_i1           CGGGGCTGAGGTGTTTGCCACGTGGTCGCCACTCGCTTCACCGCTGTTAGGATTGCCGTT
TRINITY_DN7_c0_g2_i4           CGGGGCTGAGGTGTTTGCCACGTGGTCGCCACTCGCTTCACCGCTGTTAGGATTGCCGTT
TRINITY_DN7_c0_g2_i3           CGGGGCTGAGGTGTTTGCCACGTGGTCGCCACTCGCTTCACCGCTGTTAGGATTGCCGTT
TRINITY_DN7_c0_g2_i2           CGGGGCTGAGGTGTTTGCCACGTGGTCGCCACTCGCTTCACCGCTGTTAGGATTGCCGTT
                               ************************************************************

MpDNV_mRNA_seq_consensus       ACCGTCGTAGTCCCAATCCAAATCAGGATCCTCCTCCACGCCACCTAAAACATACGGTGG 3' splice site
TRINITY_DN34_c0_g2_i3          ACCGTCGTAGTCCCAATCCAAATCAGGATCCTCCTCCACGCCAC---------------- spliced intron 1
TRINITY_DN34_c0_g2_i2          ACCGTCGTAGTCCCAATCCAAATCAGGATCCTCCTCCACGCCACCTAAAACATACGGTGG VP unspliced
TRINITY_DN34_c0_g2_i1          ACCGTCGTAGTCCCAATCCAAATCAGGATCCTCCTCCACGCCAC---------------- spliced intron 1
TRINITY_DN34_c0_g2_i4          ACCGTCGTAGTCCCAATCCAAATCAGGATCCTCCTCCACGCCACCTAAAACATACGGTGG VP unspliced
TRINITY_DN29_c0_g1_i2          ACCGTCGTAGTCCCAATCCAAATCAGGATCCTCCTCCACGCCACCTAAAACATACGGTGG VP unspliced
TRINITY_DN29_c0_g1_i3          ACCGTCGTAGTCCCAATCCAAATCAGGATCCTCCTCCACGCCAC---------------- spliced intron 1
TRINITY_DN29_c0_g1_i4          ACCGTCGTAGTCCCAATCCAAATCAGGATCCTCCTCCACGCCACCTAAAACATACGGTGG VP unspliced
TRINITY_DN29_c0_g1_i1          ACCGTCGTAGTCCCAATCCAAATCAGGATCCTCCTCCACGCCAC---------------- spliced intron 1
TRINITY_DN7_c0_g2_i1           ACCGTCGTAGTCCCAATCCAAATCAGGATCCTCCTCCACGCCAC---------------- spliced intron 1
TRINITY_DN7_c0_g2_i4           ACCGTCGTAGTCCCAATCCAAATCAGGATCCTCCTCCACGCCACCTAAAACATACGGTGG VP unspliced
TRINITY_DN7_c0_g2_i3           ACCGTCGTAGTCCCAATCCAAATCAGGATCCTCCTCCACGCCAC---------------- spliced intron 1
TRINITY_DN7_c0_g2_i2           ACCGTCGTAGTCCCAATCCAAATCAGGATCCTCCTCCACGCCACCTAAAACATACGGTGG VP unspliced
                               ********************************************

MpDNV_mRNA_seq_consensus       TAATGAACGACGTGCTAAAGCCTTATTGTATTGTTCTAGCGCGTAACGCTGCTGCGATGA
TRINITY_DN34_c0_g2_i3          ------------------------------------------------------------
TRINITY_DN34_c0_g2_i2          TAATGAACGACGTGCTAAAGCCTTATTGTATTGTTCTAGCGCGTAACGCTGCTGCGATGA
TRINITY_DN34_c0_g2_i1          ------------------------------------------------------------
TRINITY_DN34_c0_g2_i4          TAATGAACGACGTGCTAAAGCCTTATTGTATTGTTCTAGCGCGTAACGCTGCTGCGATGA
TRINITY_DN29_c0_g1_i2          TAATGAACGACGTGCTAAAGCCTTATTGTATTGTTCTAGCGCGTAACGCTGCTGCGATGA
TRINITY_DN29_c0_g1_i3          ------------------------------------------------------------
TRINITY_DN29_c0_g1_i4          TAATGAACGACGTGCTAAAGCCTTATTGTATTGTTCTAGCGCGTAACGCTGCTGCGATGA
TRINITY_DN29_c0_g1_i1          ------------------------------------------------------------
TRINITY_DN7_c0_g2_i1           ------------------------------------------------------------
TRINITY_DN7_c0_g2_i4           TAATGAACGACGTGCTAAAGCCTTATTGTATTGTTCTAGCGCGTAACGCTGCTGCGATGA
TRINITY_DN7_c0_g2_i3           ------------------------------------------------------------
TRINITY_DN7_c0_g2_i2           TAATGAACGACGTGCTAAAGCCTTATTGTATTGTTCTAGCGCGTAACGCTGCTGCGATGA


MpDNV_mRNA_seq_consensus       ATTTAAATTTTGCCAATTGGCCCGCTCCGGTGGAGGAGGACCTAAATAACGAAATTCTCG
TRINITY_DN34_c0_g2_i3          ------------------------------------------------------------
TRINITY_DN34_c0_g2_i2          ATTTAAATTTTGCCAATTGGCCCGCTCCGGTGGAGGAGGACCTAAATAACGAAATTCTCG
TRINITY_DN34_c0_g2_i1          ------------------------------------------------------------
TRINITY_DN34_c0_g2_i4          ATTTAAATTTTGCCAATTGGCCCGCTCCGGTGGAGGAGGACCTAAATAACGAAATTCTCG
TRINITY_DN29_c0_g1_i2          ATTTAAATTTTGCCAATTGGCCCGCTCCGGTGGAGGAGGACCTAAATAACGAAATTCTCG
TRINITY_DN29_c0_g1_i3          ------------------------------------------------------------
TRINITY_DN29_c0_g1_i4          ATTTAAATTTTGCCAATTGGCCCGCTCCGGTGGAGGAGGACCTAAATAACGAAATTCTCG
TRINITY_DN29_c0_g1_i1          ------------------------------------------------------------
TRINITY_DN7_c0_g2_i1           ------------------------------------------------------------
TRINITY_DN7_c0_g2_i4           ATTTAAATTTTGCCAATTGGCCCGCTCCGGTGGAGGAGGACCTAAATAACGAAATTCTCG
TRINITY_DN7_c0_g2_i3           ------------------------------------------------------------
TRINITY_DN7_c0_g2_i2           ATTTAAATTTTGCCAATTGGCCCGCTCCGGTGGAGGAGGACCTAAATAACGAAATTCTCG


MpDNV_mRNA_seq_consensus       CATACTTACGGCTACTGTCTGCTGCCGCTAAATACGGTGTTGTATCAAAATATGTTCCGC 5' splice site
TRINITY_DN34_c0_g2_i3  APFV10 ---------GGCTACTGTCTGCTGCCGCTAAATACGGTGTTGTATCAAAATATGTTCCGC spliced intron 1
TRINITY_DN34_c0_g2_i2  APFV10 CATACTTACGGCTACTGTCTGCTGCCGCTAAATACGGTGTTGTATCAAAATATGTTCCGC VP unspliced
TRINITY_DN34_c0_g2_i1  APFV10 ---------GGCTACTGTCTGCTGCCGCTAAATACGGTGTTGTATCAAAATATGTTCCGC spliced intron 1
TRINITY_DN34_c0_g2_i4  APFV10 CATACTTACGGCTACTGTCTGCTGCCGCTAAATACGGTGTTGTATCAAAATATGTTCCGC VP unspliced
TRINITY_DN29_c0_g1_i2  APFV8  CATACTTACGGCTACTGTCTGCTGCCGCTAAATACGGTGTTGTATCAAAATATGTCCCGC VP unspliced
TRINITY_DN29_c0_g1_i3  APFV8  ---------GGCTACTGTCTGCTGCCGCTAAATACGGTGTTGTATCAAAATATGTCCCGC spliced intron 1
TRINITY_DN29_c0_g1_i4  APFV8  CATACTTACGGCTACTGTCTGCTGCCGCTAAATACGGTGTTGTATCAAAATATGTCCCGC VP unspliced
TRINITY_DN29_c0_g1_i1  APFV8  ---------GGCTACTGTCTGCTGCCGCTAAATACGGTGTTGTATCAAAATATGTCCCGC spliced intron 1
TRINITY_DN7_c0_g2_i1   APFV9  ---------GGCTACTGTCTGCTGCCGCTAAATACGGTGTTGTATCAAAATATGTCCCGC spliced intron 1
TRINITY_DN7_c0_g2_i4   APFV9  CATACTTACGGCTACTGTCTGCTGCCGCTAAATACGGTGTTGTATCAAAATATGTCCCGC VP unspliced
TRINITY_DN7_c0_g2_i3   APFV9  ---------GGCTACTGTCTGCTGCCGCTAAATACGGTGTTGTATCAAAATATGTCCCGC spliced intron 1
TRINITY_DN7_c0_g2_i2   APFV9  CATACTTACGGCTACTGTCTGCTGCCGCTAAATACGGTGTTGTATCAAAATATGTCCCGC VP unspliced
                                       ********************************************** ****

MpDNV_mRNA_seq_consensus       TAGTGTTAGGTTTGTTCGGGTATATTGCTGTCTCGCTGCCTGTCAAGAACTGATCGGCTA
TRINITY_DN34_c0_g2_i3          TAGTGTTAGGTTTGTTCGGGTATATTGCTGTCTCGCTGCCTGTCAAGAACTGATCGGCTA
TRINITY_DN34_c0_g2_i2          TAGTGTTAGGTTTGTTCGGGTATATTGCTGTCTCGCTGCCTGTCAAGAACTGATCGGCTA
TRINITY_DN34_c0_g2_i1          TAGTGTTAGGTTTGTTCGGGTATATTGCTGTCTCGCTGCCTGTCAAGAACTGATCGGCTA
TRINITY_DN34_c0_g2_i4          TAGTGTTAGGTTTGTTCGGGTATATTGCTGTCTCGCTGCCTGTCAAGAACTGATCGGCTA
TRINITY_DN29_c0_g1_i2          TAGTGTTAGGTTTGTTCGGGTATATTGCTGTCTCGCTGCCTGTCAAGAACTGATCGGCTA
TRINITY_DN29_c0_g1_i3          TAGTGTTAGGTTTGTTCGGGTATATTGCTGTCTCGCTGCCTGTCAAGAACTGATCGGCTA
TRINITY_DN29_c0_g1_i4          TAGTGTTAGGTTTGTTCGGGTATATTGCTGTCTCGCTGCCTGTCAAGAACTGATCGGCTA
TRINITY_DN29_c0_g1_i1          TAGTGTTAGGTTTGTTCGGGTATATTGCTGTCTCGCTGCCTGTCAAGAACTGATCGGCTA
TRINITY_DN7_c0_g2_i1           TAGTGTTAGGTTTGTTCGGGTATATTGCTGTCTCGCTGCCTGTCAAGAACTGATCGGCTA
TRINITY_DN7_c0_g2_i4           TAGTGTTAGGTTTGTTCGGGTATATTGCTGTCTCGCTGCCTGTCAAGAACTGATCGGCTA
TRINITY_DN7_c0_g2_i3           TAGTGTTAGGTTTGTTCGGGTATATTGCTGTCTCGCTGCCTGTCAAGAACTGATCGGCTA
TRINITY_DN7_c0_g2_i2           TAGTGTTAGGTTTGTTCGGGTATATTGCTGTCTCGCTGCCTGTCAAGAACTGATCGGCTA
                               ************************************************************

MpDNV_mRNA_seq_consensus       AATCGACACCACGCTTTGCGATCAATCCGAGAGCTGTCGCAATGTTGCCATAATATGGGT
TRINITY_DN34_c0_g2_i3          AATCGACACCACGCTTTGCGATCAATCCGAGAGCTGTCGCAATGTTGCCATAATATGGGT
```

```
TRINITY_DN34_c0_g2_i2         AATCGACACCACGCTTTGCGATCAATCCGAGAGCTGTCGCAATGTTGCCATAATATGGGT
TRINITY_DN34_c0_g2_i1         AATCGACACCACGCTTTGCGATCAATCCGAGAGCTGTCGCAATGTTGCCATAATATGGGT
TRINITY_DN34_c0_g2_i4         AATCGACACCACGCTTTGCGATCAATCCGAGAGCTGTCGCAATGTTGCCATAATATGGGT
TRINITY_DN29_c0_g1_i2         AATCGACACCACGCTTTGCGATCAATCCGAGAGCTGTCGCAATGTTGCCATAATATGGGT
TRINITY_DN29_c0_g1_i3         AATCGACACCACGCTTTGCGATCAATCCGAGAGCTGTCGCAATGTTGCCATAATATGGGT
TRINITY_DN29_c0_g1_i4         AATCGACACCACGCTTTGCGATCAATCCGAGAGCTGTCGCAATGTTGCCATAATATGGGT
TRINITY_DN29_c0_g1_i1         AATCGACACCACGCTTTGCGATCAATCCGAGAGCTGTCGCAATGTTGCCATAATATGGGT
TRINITY_DN7_c0_g2_i1          AATCGACACCACGCTTTGCGATCAATCCGAGAGCTGTCGCAATGTTGCCATAATATGGGT
TRINITY_DN7_c0_g2_i4          AATCGACACCACGCTTTGCGATCAATCCGAGAGCTGTCGCAATGTTGCCATAATATGGGT
TRINITY_DN7_c0_g2_i3          AATCGACACCACGCTTTGCGATCAATCCGAGAGCTGTCGCAATGTTGCCATAATATGGGT
TRINITY_DN7_c0_g2_i2          AATCGACACCACGCTTTGCGATCAATCCGAGAGCTGTCGCAATGTTGCCATAATATGGGT
                              ************************************************************

MpDNV_mRNA_seq_consensus      CGCTGACTTGCAGCGCTCCCCGTATCGCTTTTTTGTCTAATTCCCATATGTTATCCTTGC
TRINITY_DN34_c0_g2_i3         CGCTGACTTGCAGCGCTCCCCGTATCGCTTTTTTGTCTAATTCCCATATGTTATCCTTGC
TRINITY_DN34_c0_g2_i2         CGCTGACTTGCAGCGCTCCCCGTATCGCTTTTTTGTCTAATTCCCATATGTTATCCTTGC
TRINITY_DN34_c0_g2_i1         CGCTGACTTGCAGCGCTCCCCGTATCGCTTTTTTGTCTAATTCCCATATGTTATCCTTGC
TRINITY_DN34_c0_g2_i4         CGCTGACTTGCAGCGCTCCCCGTATCGCTTTTTTGTCTAATTCCCATATGTTATCCTTGC
TRINITY_DN29_c0_g1_i2         CGCTGACTTGCAGCGCTCCCCGTATCGCTTTTTTGTCTAATTCCCATATGTTATCCTTGC
TRINITY_DN29_c0_g1_i3         CGCTGACTTGCAGCGCTCCCCGTATCGCTTTTTTGTCTAATTCCCATATGTTATCCTTGC
TRINITY_DN29_c0_g1_i4         CGCTGACTTGCAGCGCTCCCCGTATCGCTTTTTTGTCTAATTCCCATATGTTATCCTTGC
TRINITY_DN29_c0_g1_i1         CGCTGACTTGCAGCGCTCCCCGTATCGCTTTTTTGTCTAATTCCCATATGTTATCCTTGC
TRINITY_DN7_c0_g2_i1          CGCTGACTTGCAGCGCTCCCCGTATCGCTTTTTTGTCTAATTCCCATATGTTATCCTTGC
TRINITY_DN7_c0_g2_i4          CGCTGACTTGCAGCGCTCCCCGTATCGCTTTTTTGTCTAATTCCCATATGTTATCCTTGC
TRINITY_DN7_c0_g2_i3          CGCTGACTTGCAGCGCTCCCCGTATCGCTTTTTTGTCTAATTCCCATATGTTATCCTTGC
TRINITY_DN7_c0_g2_i2          CGCTGACTTGCAGCGCTCCCCGTATCGCTTTTTTGTCTAATTCCCATATGTTATCCTTGC
                              ************************************************************

MpDNV_mRNA_seq_consensus      TAACATCCGTAGCATTAAAATAATCGGCATCGTGTTGAAATGCGACCTTATCTATATCAC
TRINITY_DN34_c0_g2_i3         TAACATCCGTAGCATTAAAATAATCGGCATCGTGTTGGAATGCGACCTTATCTATATCAC
TRINITY_DN34_c0_g2_i2         TAACATCCGTAGCATTAAAATAATCGGCATCGTGTTGGAATGCGACCTTATCTATATCAC
TRINITY_DN34_c0_g2_i1         TAACATCCGTAGCATTAAAATAATCGGCATCGTGTTGGAATGCGACCTTATCTATATCAC
TRINITY_DN34_c0_g2_i4         TAACATCCGTAGCATTAAAATAATCGGCATCGTGTTGGAATGCGACCTTATCTATATCAC
TRINITY_DN29_c0_g1_i2         TAACATCCGTAGCATTAAAATAATCGGCATCGTGTTGAAATGCGACCTTATCTATATCAC
TRINITY_DN29_c0_g1_i3         TAACATCCGTAGCATTAAAATAATCGGCATCGTGTTGAAATGCGACCTTATCTATATCAC
TRINITY_DN29_c0_g1_i4         TAACATCCGTAGCATTAAAATAATCGGCATCGTGTTGAAATGCGACCTTATCTATATCAC
TRINITY_DN29_c0_g1_i1         TAACATCCGTAGCATTAAAATAATCGGCATCGTGTTGAAATGCGACCTTATCTATATCAC
TRINITY_DN7_c0_g2_i1          TAACATCCGTAGCATTAAAATAATCGGCATCGTGTTGGAATGCGACCTTATCTATATCAC
TRINITY_DN7_c0_g2_i4          TAACATCCGTAGCATTAAAATAATCGGCATCGTGTTGGAATGCGACCTTATCTATATCAC
TRINITY_DN7_c0_g2_i3          TAACATCCGTAGCATTAAAATAATCGGCATCGTGTTGGAATGCGACCTTATCTATATCAC
TRINITY_DN7_c0_g2_i2          TAACATCCGTAGCATTAAAATAATCGGCATCGTGTTGGAATGCGACCTTATCTATATCAC
                              ************************************* **********************

MpDNV_mRNA_seq_consensus      TAGTAGGTAACGCCTTAGCATTAAAATGACCTTCACTGTCACACAACAGATTACCTGGAC
TRINITY_DN34_c0_g2_i3         TAGTAGGTAACGCCTTAGCATTAAAATGACCTTCACTGTCACACAACAGATTACCTGGAC
TRINITY_DN34_c0_g2_i2         TAGTAGGTAACGCCTTAGCATTAAAATGACCTTCACTGTCACACAACAGATTACCTGGAC
TRINITY_DN34_c0_g2_i1         TAGTAGGTAACGCCTTAGCATTAAAATGACCTTCACTGTCACACAACAGATTACCTGGAC
TRINITY_DN34_c0_g2_i4         TAGTAGGTAACGCCTTAGCATTAAAATGACCTTCACTGTCACACAACAGATTACCTGGAC
TRINITY_DN29_c0_g1_i2         TAGTAGGTAACGCCTTAGCATTAAAATGACCTTCACTGTCACACAACAGATTACCTGGAC
TRINITY_DN29_c0_g1_i3         TAGTAGGTAACGCCTTAGCATTAAAATGACCTTCACTGTCACACAACAGATTACCTGGAC
TRINITY_DN29_c0_g1_i4         TAGTAGGTAACGCCTTAGCATTAAAATGACCTTCACTGTCACACAACAGATTACCTGGAC
TRINITY_DN29_c0_g1_i1         TAGTAGGTAACGCCTTAGCATTAAAATGACCTTCACTGTCACACAACAGATTACCTGGAC
TRINITY_DN7_c0_g2_i1          TAGTAGGTAACGCCTTAGCATTAAAATGACCTTCACTGTCACACAACAGATTACCTGGAC
TRINITY_DN7_c0_g2_i4          TAGTAGGTAACGCCTTAGCATTAAAATGACCTTCACTGTCACACAACAGATTACCTGGAC
TRINITY_DN7_c0_g2_i3          TAGTAGGTAACGCCTTAGCATTAAAATGACCTTCACTGTCACACAACAGATTACCTGGAC
TRINITY_DN7_c0_g2_i2          TAGTAGGTAACGCCTTAGCATTAAAATGACCTTCACTGTCACACAACAGATTACCTGGAC
                              ************************************************************

MpDNV_mRNA_seq_consensus      CCACGAAGCTACTACCGAACCACGTGATACCTTCATTGCTAGTACCTGAAGCACCTAATT
TRINITY_DN34_c0_g2_i3         CCACGAAGCTACTACCGAACCACGTGATACCTTCATTGCTAGTACCTGAAGCACCTAATT
TRINITY_DN34_c0_g2_i2         CCACGAAGCTACTACCGAACCACGTGATACCTTCATTGCTAGTACCTGAAGCACCTAATT
TRINITY_DN34_c0_g2_i1         CCACGAAGCTACTACCGAACCACGTGATACCTTCATTGCTAGTACCTGAAGCACCTAATT
TRINITY_DN34_c0_g2_i4         CCACGAAGCTACTACCGAACCACGTGATACCTTCATTGCTAGTACCTGAAGCACCTAATT
TRINITY_DN29_c0_g1_i2         CCACGAAGCTACTACCGAACCACGTGATACCTTCATTGCTAGTACCTGAAGCACCTAATT
TRINITY_DN29_c0_g1_i3         CCACGAAGCTACTACCGAACCACGTGATACCTTCATTGCTAGTACCTGAAGCACCTAATT
TRINITY_DN29_c0_g1_i4         CCACGAAGCTACTACCGAACCACGTGATACCTTCATTGCTAGTACCTGAAGCACCTAATT
TRINITY_DN29_c0_g1_i1         CCACGAAGCTACTACCGAACCACGTGATACCTTCATTGCTAGTACCTGAAGCACCTAATT
TRINITY_DN7_c0_g2_i1          CCACGAAGCTACTACCGAACCACGTGATACCTTCATTGCTAGTACCTGAAGCACCTAATT
TRINITY_DN7_c0_g2_i4          CCACGAAGCTACTACCGAACCACGTGATACCTTCATTGCTAGTACCTGAAGCACCTAATT
TRINITY_DN7_c0_g2_i3          CCACGAAGCTACTACCGAACCACGTGATACCTTCATTGCTAGTACCTGAAGCACCTAATT
TRINITY_DN7_c0_g2_i2          CCACGAAGCTACTACCGAACCACGTGATACCTTCATTGCTAGTACCTGAAGCACCTAATT
                              ************************************************************

MpDNV_mRNA_seq_consensus      TAATACGTTTTCTCTCGTTATTCTCCTCTTCAACACTATAAAATTCGTCGTTTACCGACG
TRINITY_DN34_c0_g2_i3         TAATACGTTTTCTCTCGTTATTCTCCTCTTCAACACTATAAAATTCGTCGTTTACCGACG
TRINITY_DN34_c0_g2_i2         TAATACGTTTTCTCTCGTTATTCTCCTCTTCAACACTATAAAATTCGTCGTTTACCGACG
TRINITY_DN34_c0_g2_i1         TAATACGTTTTCTCTCGTTATTCTCCTCTTCAACACTATAAAATTCGTCGTTTACCGACG
TRINITY_DN34_c0_g2_i4         TAATACGTTTTCTCTCGTTATTCTCCTCTTCAACACTATAAAATTCGTCGTTTACCGACG
TRINITY_DN29_c0_g1_i2         TAATACGTTTTCTCTCGTTATTCTCCTCTTCAACACTATAAAATTCGTCGTTTACCGACG
TRINITY_DN29_c0_g1_i3         TAATACGTTTTCTCTCGTTATTCTCCTCTTCAACACTATAAAATTCGTCGTTTACCGACG
TRINITY_DN29_c0_g1_i4         TAATACGTTTTCTCTCGTTATTCTCCTCTTCAACACTATAAAATTCGTCGTTTACCGACG
TRINITY_DN29_c0_g1_i1         TAATACGTTTTCTCTCGTTATTCTCCTCTTCAACACTATAAAATTCGTCGTTTACCGACG
TRINITY_DN7_c0_g2_i1          TAATACGTTTTCTCTCGTTATTCTCCTCTTCAACACTATAAAATTCGTCGTTTACCGACG
TRINITY_DN7_c0_g2_i4          TAATACGTTTTCTCTCGTTATTCTCCTCTTCAACACTATAAAATTCGTCGTTTACCGACG
TRINITY_DN7_c0_g2_i3          TAATACGTTTTCTCTCGTTATTCTCCTCTTCAACACTATAAAATTCGTCGTTTACCGACG
TRINITY_DN7_c0_g2_i2          TAATACGTTTTCTCTCGTTATTCTCCTCTTCAACACTATAAAATTCGTCGTTTACCGACG
                              ************************************************************
```

```
MpDNV_mRNA_seq_consensus      ACATGTTAAACGTAACTGATTAAATATAAGTAAAATGTAAACACTTGAGTAAATAGTGAT
TRINITY_DN34_c0_g2_i3  APFV10 ACATGTTAAACGTAACTGATTAAATATAAGTCAAATGTAAACACCCGAGTAAAGAGTG-- VP 5'-end
TRINITY_DN34_c0_g2_i2         ACATGTTAAACGTAACTGATTAAATATAAGTCAAATGTAAACACCCGAGTAAAGAGTG-- VP 5'-end
TRINITY_DN34_c0_g2_i1         ACATGTTAAACGTAACTGATTAAATATAAGTCAAATGTAAACACCCGAGTAAAGAGTG-- VP 5'-end
TRINITY_DN34_c0_g2_i4         ACATGTTAAACGTAACTGATTAAATATAAGTCAAATGTAAACACCCGAGTAAAGAGTG-- VP 5'-end
TRINITY_DN29_c0_g1_i2  APFV8  ACATGTTAAACGTAACTGATTAAATATAAGTAAAATGTAAACACTTGAGTAAATAGTGAT
TRINITY_DN29_c0_g1_i3         ACATGTTAAACGTAACTGATTAAATATAAGTAAAATGTAAACACTTGAGTAAATAGTGAT
TRINITY_DN29_c0_g1_i4         ACATGTTAAACGTAACTGATTAAATATAAGTAAAATGTAAACACTTGAGTAAATAGTGAT
TRINITY_DN29_c0_g1_i1         ACATGTTAAACGTAACTGATTAAATATAAGTAAAATGTAAACACTTGAGTAAATAGTGAT
TRINITY_DN7_c0_g2_i1   APFV9  ACATGTTAAACGTAACTGATTAAATATAAGTAAAATGTAAACACTTGAGTAAATAGTGAT
TRINITY_DN7_c0_g2_i4          ACATGTTAAACGTAACTGATTAAATATAAGTAAAATGTAAACACTTGAGTAAATAGTGAT
TRINITY_DN7_c0_g2_i3          ACATGTTAAACGTAACTGATTAAATATAAGTAAAATGTAAACACTTGAGTAAATAGTGAT
TRINITY_DN7_c0_g2_i2          ACATGTTAAACGTAACTGATTAAATATAAGTAAAATGTAAACACTTGAGTAAATAGTGAT
                              ******************************* ************  ******* ****

MpDNV_mRNA_seq_consensus      GACGTCACCCCCACCTAGTCGCCTACTGGATACTTGTCTATCTTTTTATTATATAAAAG 3'ITR TATA-box
TRINITY_DN34_c0_g2_i3         ------------------------------------------------------------
TRINITY_DN34_c0_g2_i2         ------------------------------------------------------------
TRINITY_DN34_c0_g2_i1         ------------------------------------------------------------
TRINITY_DN34_c0_g2_i4         ------------------------------------------------------------
TRINITY_DN29_c0_g1_i2         GACGTCACCCCCACCTAGTCGCCTACTGGATACTTGTCTATCTTTTTATTATATAAAAAG
TRINITY_DN29_c0_g1_i3         GACGTCACCCCCACCTAGTCGCCTACTGGATACTTGTCTATCTTTTTATTATATAAAAAG
TRINITY_DN29_c0_g1_i4         GACGTCACCCCCACCTAGTCGCCTACTGGATACTTGTCTATCTTTTTATTATATAAAAAG
TRINITY_DN29_c0_g1_i1         GACGTCACCCCCACCTAGTCGCCTACTGGATACTTGTCTATCTTTTTATTATATAAAAAG
TRINITY_DN7_c0_g2_i1          GACGTCACCCCCACCTAGTCGCCTACTGGATACTTGTCTATCTTTTTATTATATAAAAAG
TRINITY_DN7_c0_g2_i4          GACGTCACCCCCACCTAGTCGCCTACTGGATACTTGTCTATCTTTTTATTATATAAAAAG
TRINITY_DN7_c0_g2_i3          GACGTCACCCCCACCTAGTCGCCTACTGGATACTTGTCTATCTTTTTATTATATAAAAAG
TRINITY_DN7_c0_g2_i2          GACGTCACCCCCACCTAGTCGCCTACTGGATACTTGTCTATCTTTTTATTATATAAAAAG

MpDNV_mRNA_seq_consensus      ATCCCCTACTAATAATAACCACTACCTACTTCCGGGGGGCGGGGGGTATCCTTTGTTATC
TRINITY_DN34_c0_g2_i3         ------------------------------------------------------------
TRINITY_DN34_c0_g2_i2         ------------------------------------------------------------
TRINITY_DN34_c0_g2_i1         ------------------------------------------------------------
TRINITY_DN34_c0_g2_i4         ------------------------------------------------------------
TRINITY_DN29_c0_g1_i2         ATCCCCCACTAATAATAACC----------------------------------------
TRINITY_DN29_c0_g1_i3         ATCCCCCACTAATAATAACC----------------------------------------
TRINITY_DN29_c0_g1_i4         ATCCCCCACTAATAATAACC----------------------------------------
TRINITY_DN29_c0_g1_i1         ATCCCCCACTAATAATAACC----------------------------------------
TRINITY_DN7_c0_g2_i1          ATCCCCTACTAATAATAACCACTACCTACTTCCGGGGGGCGGGGGGTATCCTTTGTTATC
TRINITY_DN7_c0_g2_i4          ATCCCCTACTAATAATAACCACTACCTACTTCCGGGGGGCGGGGGGTATCCTTTGTTATC
TRINITY_DN7_c0_g2_i3          ATCCCCTACTAATAATAACCACTACCTACTTCCGGGGGGCGGGGGGTATCCTTTGTTATC
TRINITY_DN7_c0_g2_i2          ATCCCCTACTAATAATAACCACTACCTACTTCCGGGGGGCGGGGGGTATCCTTTGTTATC

MpDNV_mRNA_seq_consensus      TATACTTATTGTCAACACTAGCCTTATCTTAGGGCGTGTCGGCCGACACGTGTCATGGCC
TRINITY_DN34_c0_g2_i3         ------------------------------------------------------------
TRINITY_DN34_c0_g2_i2         ------------------------------------------------------------
TRINITY_DN34_c0_g2_i1         ------------------------------------------------------------
TRINITY_DN34_c0_g2_i4         ------------------------------------------------------------
TRINITY_DN29_c0_g1_i2         ------------------------------AGGGCT------------AGTGTCATGGCC
TRINITY_DN29_c0_g1_i3         ------------------------------AGGGCT------------AGTGTCATGGCC
TRINITY_DN29_c0_g1_i4         ------------------------------AGGGCT------------AGTGTCATGGCC
TRINITY_DN29_c0_g1_i1         ------------------------------AGGGCT------------AGTGTCATGGCC
TRINITY_DN7_c0_g2_i1          TATACTTATTGTCAACACTAGCCTTATCTTAGGGCT------------AGTGTCATGGCC
TRINITY_DN7_c0_g2_i4          TATACTTATTGTCAACACTAGCCTTATCTTAGGGCT------------AGTGTCATGGCC
TRINITY_DN7_c0_g2_i3          TATACTTATTGTCAACACTAGCCTTATCTTAGGGCT------------AGTGTCATGGCC
TRINITY_DN7_c0_g2_i2          TATACTTATTGTCAACACTAGCCTTATCTTAGGGCT------------AGTGTCATGGCC

MpDNV_mRNA_seq_consensus      ATGACACT------------AGCCCTAAGATAAGGCTAGTGTTGACAATAAGTATAGATA
TRINITY_DN34_c0_g2_i3         ------------------------------------------------------------
TRINITY_DN34_c0_g2_i2         ------------------------------------------------------------
TRINITY_DN34_c0_g2_i1         ------------------------------------------------------------
TRINITY_DN34_c0_g2_i4         ------------------------------------------------------------
TRINITY_DN29_c0_g1_i2         ATGACACGTGTCGGCCGACACGCCCTAAGATAAGGCTAGTGTCGAC-------------- VP 5'-end
TRINITY_DN29_c0_g1_i3         ATGACACGTGTCGGCCGACACGCCCTAAGATAAGGCTAGTGTCGAC-------------- VP 5'-end
TRINITY_DN29_c0_g1_i4         ATGACACGTGTCGGCCGACACGCCCTAAGATAAGGCTAGTGTCGAC-------------- VP 5'-end
TRINITY_DN29_c0_g1_i1         ATGACACGTGTCGGCCGACACGCCCTAAGATAAGGCTAGTGTCGAC-------------- VP 5'-end
TRINITY_DN7_c0_g2_i1          ATGACACGTGTCGGCCGACACGCCCTAAGATAAGGCTAGTGTTGTCTCCAAG-------- VP 5'-end
TRINITY_DN7_c0_g2_i4          ATGACACGTGTCGGCCGACACGCCCTAAGATAAGGCTAGTGTTGTCTCCAAG-------- VP 5'-end
TRINITY_DN7_c0_g2_i3          ATGACACGTGTCGGCCGACACGCCCTAAGATAAGGCTAGTGTTGTCTCCAAG-------- VP 5'-end
TRINITY_DN7_c0_g2_i2          ATGACACGTGTCGGCCGACACGCCCTAAGATAAGGCTAGTGTTGTCTCCAAG-------- VP 5'-end
```

APFV-11-12-13 (Artificial Diet + TuYV virions)

```
MpDNV_DNA_RNA_seq_consensus     GTCATGGCCATGACACTAGCCCTAAGATAAGGCTAGTGTTGACAATAAGTATAGATAAC-
TRINITY_DN2570_c0_g2_i1 APFV-13 -------CCATGACACTAGCCCTAAGATAAGGCTAGTGTTGACAATAAGTATAGATAACA NS 5'-end
TRINITY_DN7_c0_g1_i2    APFV-11 ------------------------------------------------------------
TRINITY_DN7_c0_g1_i1    APFV-11 ------------------------------------------------------------
TRINITY_DN11_c0_g1_i4   APFV-12 ------------------------------------------------------------
TRINITY_DN11_c0_g1_i1   APFV-12 ------------------------------------------------------------

MpDNV_DNA_RNA_seq_consensus    AAGGATACCCCCCGCCCCCCGGAAGTAGGTAGTGGTTATTATTAGTAGGGGATCTTTTTA 5'ITR TATA-box
TRINITY_DN2570_c0_g2_i1        AAGGATACCCCCCGCCCCCCGGAAGTAGGTAGTGGTTATTATTAGTGGGGGATCTTTTTA
```

```
TRINITY_DN7_c0_g1_i2                 ------------------------------------------------------------
TRINITY_DN7_c0_g1_i1                 ------------------------------------------------------------
TRINITY_DN11_c0_g1_i4                ------------------------------------------------------------
TRINITY_DN11_c0_g1_i1                ------------------------------------------------------------


MpDNV_DNA_RNA_seq_consensus          TATAATAAAAAGATAGACAAGTATCCAGTAGGCGACTAGGTGGGGGTGACGTCATCACTA
TRINITY_DN2570_c0_g2_i1              TATAATAAAAAGATAGACAAGTATCCAGTAGGCGACTAGGTGGGGGTGACGTCATCACTA
TRINITY_DN7_c0_g1_i2                 ------------------------------------------------------------
TRINITY_DN7_c0_g1_i1                 ------------------------------------------------------------
TRINITY_DN11_c0_g1_i4                ------------------------------------------------------------
TRINITY_DN11_c0_g1_i1                ------------------------------------------------------------


MpDNV_DNA_RNA_seq_consensus          TTTACTCAAGTGTTTTGTTATTCGGCGTCAAGTGTTGTGTTGACACTTGAAAAGTGGCGT
TRINITY_DN2570_c0_g2_i1              TTTACTCAAGTGTTTTGTTATTCGGCGTCAAGTGTTGTGTTGACACTTGAAAAGTGGCGT
TRINITY_DN7_c0_g1_i2                 ------------------------------------------------------------
TRINITY_DN7_c0_g1_i1                 ------------------------------------------------------------
TRINITY_DN11_c0_g1_i4                ------------------------------------------------------------
TRINITY_DN11_c0_g1_i1                ------------------------------------------------------------


MpDNV_DNA_RNA_seq_consensus          GATTGATTATTTACAAGTACGTCAGATAAGATAAACATAAGTGGTACTTGACTCGGAATA
TRINITY_DN2570_c0_g2_i1  APFV-13 GATTGATTATTTACAAGTACGTCAGATAAGATAAACATAAGTGGTACTTGACTCGGAATA
TRINITY_DN7_c0_g1_i2     APFV-11 -ATTGATTATCTACAAGTACGTCAGATAAGATAAACATAAGTGGTACTTGACTCGGAATA NS 5'-end
TRINITY_DN7_c0_g1_i1             -ATTGATTATCTACAAGTACGTCAGATAAGATAAACATAAGTGGTACTTGACTCGGAATA NS 5'-end
TRINITY_DN11_c0_g1_i4    APFV-12 ---TGATTATCTACAAGTACGTCAGATAAGATAAACATAAGTGGTACTTGACTCGGAATA NS 5'-end
TRINITY_DN11_c0_g1_i1            ---TGATTATCTACAAGTACGTCAGATAAGATAAACATAAGTGGTACTTGACTCGGAATA NS 5'-end
                                    ******* *************************************************

MpDNV_DNA_RNA_seq_consensus          TATAATGAAGTGTGGAACCTACAACAATATCAGTACTCATCAAAATGTCAAACTCGCAAG
TRINITY_DN2570_c0_g2_i1              TATAATGAAGTGTGGAACCTACAACAATATCAGTACTCATCAAAATGTCAAACTCGCAAG
TRINITY_DN7_c0_g1_i2                 TATAATGAAGTGTGGAACCTACAACAATATCAGTACTCATCAAAATGTCAAACTCGCAAG
TRINITY_DN7_c0_g1_i1                 TATAATGAAGTGTGGAACCTACAACAATATCAGTACTCATCAAAATGTCAAACTCGCAAG
TRINITY_DN11_c0_g1_i4    APFV-12 TATAATGAAGTGTGGAACCTACAACAATGTCAGTACTCATCAAAATGTCAAACTCGCAAG
TRINITY_DN11_c0_g1_i1                TATAATGAAGTGTGGAACCTACAACAATGTCAGTACTCATCAAAATGTCAAACTCGCAAG
                                     **************************** *******************************

MpDNV_DNA_RNA_seq_consensus          AAGTGAACCAACAACAATTCAACGACGAGGAGGAAATACCATGTGGCCAACGGTATCCTA
TRINITY_DN2570_c0_g2_i1              AAGTGAACCAACAACAATTCAACGACGAGGAGGAAATACCATGTGGCCAACGGTATCCTA
TRINITY_DN7_c0_g1_i2                 AAGTGAACCAACAACAATTCAACGACGAGGAGGAAATACCATGTGGCCAACGGTATCCTA
TRINITY_DN7_c0_g1_i1                 AAGTGAACCAACAACAATTCAACGACGAGGAGGAAATACCATGTGGCCAACGGTATCCTA
TRINITY_DN11_c0_g1_i4                AAGTGAACCAACAACAATTCAACGACGAGGAGGAAATACCATGTGGCCAACGGTATCCTA
TRINITY_DN11_c0_g1_i1                AAGTGAACCAACAACAATTCAACGACGAGGAGGAAATACCATGTGGCCAACGGTATCCTA
                                     ************************************************************

MpDNV_DNA_RNA_seq_consensus          CAGCGGCGACGATTCAAACGGACCACCAACAGACTGGCGTGAATGGATCGACCCCGATGT
TRINITY_DN2570_c0_g2_i1              CAGCGGCGACGATTCAAACGGACCACCAACAGAGTGGCGTGAATGGATCGACCCCGATGT
TRINITY_DN7_c0_g1_i2                 CAGCGGCGACGATTCAAACGGACCACCAACAGAGTGGCGTGAATGGATCGACCCCGATGT
TRINITY_DN7_c0_g1_i1                 CAGCGGCGACGATTCAAACGGACCACCAACAGAGTGGCGTGAATGGATCGACCCCGATGT
TRINITY_DN11_c0_g1_i4                CAGCGGCGACGATTCAAACGGACCACCAACAGAGTGGCGTGAATGGATCGACCCCGATGT
TRINITY_DN11_c0_g1_i1                CAGCGGCGACGATTCAAACGGACCACCAACAGAGTGGCGTGAATGGATCGACCCCGATGT
                                     ********************************* **************************

MpDNV_DNA_RNA_seq_consensus          TCGAACTCAAATCGACAGCGCGCAAAGAATATGGGACAGCCATTTCAGCGGACACGGCAC
TRINITY_DN2570_c0_g2_i1              TCGAACTCAAATCGACAGCGCGCAAAGAATATGGGACAGCCATTTCAGCGGACACGGCAC
TRINITY_DN7_c0_g1_i2                 TCGAACTCAAATCGACAGCGCGCAAAGAATATGGGACAGCCATTTCAGCGGACACGGCAC
TRINITY_DN7_c0_g1_i1                 TCGAACTCAAATCGACAGCGCGCAAAGAATATGGGACAGCCATTTCAGCGGACACGGCAC
TRINITY_DN11_c0_g1_i4                TCGAACTCAAATCGACAGCGCGCAAAGAATATGGGACAGCCATTTCAGCGGACACGGCAC
TRINITY_DN11_c0_g1_i1                TCGAACTCAAATCGACAGCGCGCAAAGAATATGGGACAGCCATTTCAGCGGACACGGCAC
                                     ************************************************************

MpDNV_DNA_RNA_seq_consensus          TGACACTGAAGACGCTGTGCAAAGTGTTGGAGTGCAAGGCAACGGAACTAACGGTAACAT
TRINITY_DN2570_c0_g2_i1              TGACACTGAAGACGCTGTGCAAAGTGTTGGAGTGCAAGGCAACGGAACTAACGGTAACAT
TRINITY_DN7_c0_g1_i2                 TGACACTGAAGACGCTGTGCAAAGTGTTGGAGTGCAAGGCAACGGAACTAACGGTAACAT
TRINITY_DN7_c0_g1_i1                 TGACACTGAAGACGCTGTGCAAAGTGTTGGAGTGCAAGGCAACGGAACTAACGGTAACAT
TRINITY_DN11_c0_g1_i4                TGACACTGAAGACGCTGTGCAAAGTGTTGGAGTGCAAGGCAACGGAACTAACGGTAACAT
TRINITY_DN11_c0_g1_i1                TGACACTGAAGACGCTGTGCAAAGTGTTGGAGTGCAAGGCAACGGAACTAACGGTAACAT
                                     ************************************************************

MpDNV_DNA_RNA_seq_consensus          GCATTATTCCTCTGATCGCGTTGCTATTACCCGACCATATGTGGTCAGAGGCGACGAGCA
TRINITY_DN2570_c0_g2_i1              GCATTATTCCTCTGATCGCGTTGCTATTACCCGACCATATGTGGTCAGAGGCGACGAGCA
TRINITY_DN7_c0_g1_i2                 GCATTATTCCTCTGATCGCGTTGCTATTACCCGACCATATGTGGTCAGAGGCGACGAGCA
TRINITY_DN7_c0_g1_i1                 GCATTATTCCTCTGATCGCGTTGCTATTACCCGACCATATGTGGTCAGAGGCGACGAGCA
TRINITY_DN11_c0_g1_i4                GCATTATTCCTCTGATCGCGTTGCTATTACCCGACCATATGTGGTCAGAGGCGACGAGCA
TRINITY_DN11_c0_g1_i1                GCATTATTCCTCTGATCGCGTTGCTATTACCCGACCATATGTGGTCAGAGGCGACGAGCA
                                     ************************************************************

MpDNV_DNA_RNA_seq_consensus          GCGATCGCTGGCGATACCGACTGAGAAACGCGACCTACTGGATTTCCAATCCAACGCGGG
TRINITY_DN2570_c0_g2_i1              GCGATCGCTGGCGATACCGACTGAGAAACGCGACCTACTGGATTTCCAATCCAACGCGGG
TRINITY_DN7_c0_g1_i2                 GCGATCGCTGGCGATACCGACTGAGAAACGCGACCTACTGGATTTCCAATCCAACGCGGG
TRINITY_DN7_c0_g1_i1                 GCGATCGCTGGCGATACCGACTGAGAAACGCGACCTACTGGATTTCCAATCCAACGCGGG
TRINITY_DN11_c0_g1_i4                GCGATCGCTGGCGATACCGACTGAGAAACGCGACCTACTGGATTTCCAATCCAACGCGGG
TRINITY_DN11_c0_g1_i1                GCGATCGCTGGCGATACCGACTGAGAAACGCGACCTACTGGATTTCCAATCCAACGCGGG
                                     ************************************************************

MpDNV_DNA_RNA_seq_consensus          AATCCGAATATCAGGCTTTACTACGATGGATGGAGGAGAAAATGCCGATACCTTTAGCGA
TRINITY_DN2570_c0_g2_i1              AATCCGAATATCAGGCTTTACTACGATGGATGGAGGAGAAAATGCCGATACCTTTAGCGA
```

```
TRINITY_DN7_c0_g1_i2            AATCCGAATATCAGGCTTTACTACGATGGATGGAGGAGAAAATGCCGATACCTTTAGCGA
TRINITY_DN7_c0_g1_i1            AATCCGAATATCAGGCTTTACTACGATGGATGGAGGAGAAAATGCCGATACCTTTAGCGA
TRINITY_DN11_c0_g1_i4           AATCCGAATATCAGGCTTTACTACGATGGATGGAGGAGAAAATGCCGATACCTTTAGCGA
TRINITY_DN11_c0_g1_i1           AATCCGAATATCAGGCTTTACTACGATGGATGGAGGAGAAAATGCCGATACCTTTAGCGA
                                ************************************************************

MpDNV_DNA_RNA_seq_consensus     AATGGCAAGGTGCATGTCTACCGCGGTATGGGGAGGCAATGTCAGATATGTTTCTGACAT
TRINITY_DN2570_c0_g2_i1         AATGGCAAGGTGCATGTCTACCGCGGTATGGGGAGGCAATGTCAGATATGTTTCTGACAT
TRINITY_DN7_c0_g1_i2            AATGGCAAGGTGCATGTCTACCGCGGTATGGGGAGGCAATGTCAGATATGTTTCTGACAT
TRINITY_DN7_c0_g1_i1            AATGGCAAGGTGCATGTCTACCGCGGTATGGGGAGGCAATGTCAGATATGTTTCTGACAT
TRINITY_DN11_c0_g1_i4           AATGGCAAGGTGCATGTCTACCGCGGTATGGGGAGGCAATGTCAGATATGTTTCTGACAT
TRINITY_DN11_c0_g1_i1           AATGGCAAGGTGCATGTCTACCGCGGTATGGGGAGGCAATGTCAGATATGTTTCTGACAT
                                ************************************************************

MpDNV_DNA_RNA_seq_consensus     CCTTGTACCCGGAGGAAATTGGTCAATTAATGGAATTGTTGAGGACCTACGCCGGAGTAT
TRINITY_DN2570_c0_g2_i1         CCTTGTACCCGGAGGAAATTGGTCAATTAATGGAATTGTTGAGGACCTACGCCGGAGTAT
TRINITY_DN7_c0_g1_i2            CCTTGTACCCGGAGGAAATTGGTCAATTAATGGAATTGTTGAGGACCTACGCCGGAGTAT
TRINITY_DN7_c0_g1_i1            CCTTGTACCCGGAGGAAATTGGTCAATTAATGGAATTGTTGAGGACCTACGCCGGAGTAT
TRINITY_DN11_c0_g1_i4           CCTTGTACCCGGAGGAAATTGGTCAATTAATGGAATTGTTGAGGACCTACGCCGGAGTAT
TRINITY_DN11_c0_g1_i1           CCTTGTACCCGGAGGAAATTGGTCAATTAATGGAATTGTTGAGGACCTACGCCGGAGTAT
                                ************************************************************

MpDNV_DNA_RNA_seq_consensus     CGCAGAGCGAATGCCAAAATGCATGTACGTCGTCAGCGAACACGGCGACCACGTCCACGT
TRINITY_DN2570_c0_g2_i1         CGCAGAGCGAATGCCAAAATGCATGTACGTCGTCAGCGAACACGGCGACCACGTCCACGT
TRINITY_DN7_c0_g1_i2            CGCAGAGCGAATGCCAAAATGCATGTACGTCGTCAGCGAACACGGCGACCACGTCCACGT
TRINITY_DN7_c0_g1_i1            CGCAGAGCGAATGCCAAAATGCATGTACGTCGTCAGCGAACACGGCGACCACGTCCACGT
TRINITY_DN11_c0_g1_i4           CGCAGAGCGAATGCCAAAATGCATGTACGTCGTCAGCGAACACGGCGACCACGTCCACGT
TRINITY_DN11_c0_g1_i1           CGCAGAGCGAATGCCAAAATGCATGTACGTCGTCAGCGAACACGGCGACCACGTCCACGT
                                ************************************************************

MpDNV_DNA_RNA_seq_consensus     CGTACACACCTGTAACTACGCCACGGGCAGCTGTAGATGTAGTTTCTTACTCAACGCCGC
TRINITY_DN2570_c0_g2_i1         CGTACACACCTGTAACTACGCCACGGGCAGCTGTAGATGTAGTTTCTTACTCAACGCCGC
TRINITY_DN7_c0_g1_i2            CGTACACACCTGTAACTACGCCACGGGCAGCTGTAGATGTAGTTTCTTACTCAACGCCGC
TRINITY_DN7_c0_g1_i1            CGTACACACCTGTAACTACGCCACGGGCAGCTGTAGATGTAGTTTCTTACTCAACGCCGC
TRINITY_DN11_c0_g1_i4           CGTACACACCTGTAACTACGCCACGGGCAGCTGTAGATGTAGTTTCTTACTCAACGCCGC
TRINITY_DN11_c0_g1_i1           CGTACACACCTGTAACTACGCCACGGGCAGCTGTAGATGTAGTTTCTTACTCAACGCCGC
                                ************************************************************

MpDNV_DNA_RNA_seq_consensus     TGCTTTCCAACTATGTAGACGACGACGCATTCGAAAGAATGTTAGAGTCATCCAACTATC
TRINITY_DN2570_c0_g2_i1         TGCTTTCCAACTATGTAGACGACGACGCATTCGAAAGAATGTTAGAGTCATCCAACTATC
TRINITY_DN7_c0_g1_i2            TGCTTTCCAACTATGTAGACGACGACGCATTCGAAAGAATGTTAGAGTCATCCAACTATC
TRINITY_DN7_c0_g1_i1            TGCTTTCCAACTATGTAGACGACGACGCATTCGAAAGAATGTTAGAGTCATCCAACTATC
TRINITY_DN11_c0_g1_i4           TGCTTTCCAACTATGTAGACGACGACGCATTCGAAAGAATGTTAGAGTCATCCAACTATC
TRINITY_DN11_c0_g1_i1           TGCTTTCCAACTATGTAGACGACGACGCATTCGAAAGAATGTTAGAGTCATCCAACTATC
                                ************************************************************

MpDNV_DNA_RNA_seq_consensus     AGAAAGAGACTGGTCCCGTATCTTTCAATACTTATGTTCATCGCCCAGGAATGTCAAAGA
TRINITY_DN2570_c0_g2_i1         AGAAAGAGACTGGTCCCGTATCTTTCAATACTTATGTTCATCGCCCAGGAATGTCAAAGA
TRINITY_DN7_c0_g1_i2            AGAAAGAGACTGGTCCCGTATCTTTCAATACTTATGTTCATCGCCCAGGAATGTCAAAGA
TRINITY_DN7_c0_g1_i1            AGAAAGAGACTGGTCCCGTATCTTTCAATACTTATGTTCATCGCCCAGGAATGTCAAAGA
TRINITY_DN11_c0_g1_i4           AGAAAGAGACTGGTCCCGTATCTTTCAATACTTATGTTCATCGCCCAGGAATGTCAAAGA
TRINITY_DN11_c0_g1_i1           AGAAAGAGACTGGTCCCGTATCTTTCAATACTTATGTTCATCGCCCAGGAATGTCAAAGA
                                ************************************************************

MpDNV_DNA_RNA_seq_consensus     AGTGGGCGGCCAGTACTTTGATGGAGGATTACGTAGTAGATATTCACATATATCAGTTAG 5’splice site
TRINITY_DN2570_c0_g2_i1         AGTGGGCGGCCAGTACTTTGATGGAGGATTACGTAGTAGATATTCACATATATCAGTTAG NS unspliced
TRINITY_DN7_c0_g1_i2            AGTGGGCGGCCAGTACTTTGATGGAGGATTACGTAGTAGATATTCACATATATCAGTTAG NS unspliced
TRINITY_DN7_c0_g1_i1            AGTGGGCGGCCAGTACTTTGATGGAGGATTACGTAGTAGATATTCACATATATCA----- NS spliced
TRINITY_DN11_c0_g1_i4           AGTGGGCGGCCAGTACTTTGATGGAGGATTACGTAGTAGATATTCACATATATCAGTTAG NS unspliced
TRINITY_DN11_c0_g1_i1           AGTGGGCGGCCAGTACTTTGATGGAGGATTACGTAGTAGATATTCACATATATCA----- NS spliced
                                *******************************************************

MpDNV_DNA_RNA_seq_consensus     TATTGCGTAATTTTTTTATATACTTGGATTGGTTGACGGGTATAGAAGCGAATAGATGCG
TRINITY_DN2570_c0_g2_i1         TATTGCGTAATTTTTTTATATACTTGGATTGGTTGACGGGTATAGAAGCGAATAGATGCG
TRINITY_DN7_c0_g1_i2            TATTGCGTAATTTTTTTATATACTTGGATTGGTTGACGGGTATAGAAGCGAATAGATGCG
TRINITY_DN7_c0_g1_i1            ------------------------------------------------------------
TRINITY_DN11_c0_g1_i4           TATTGCGTAATTTTTTTATATACTTGGATTGGTTGACGGGTATAGAAGCGAATAGATGCG
TRINITY_DN11_c0_g1_i1           ------------------------------------------------------------

MpDNV_DNA_RNA_seq_consensus     TTTCATATATTAAAACTGGTATGCCTGGCAGGAGTTACGTTAAAAGCGTGATGCCAAAAA
TRINITY_DN2570_c0_g2_i1         TTTCATATATTAAAACTGGTATGCCTGGCAGGAGTTACGTTAAAAGCGTGATGCCAAAAA
TRINITY_DN7_c0_g1_i2            TTTCATATATTAAAACTGGTATGCCTGGCAGGAGTTACGTTAAAAGCGTGATGCCAAAAA
TRINITY_DN7_c0_g1_i1            ------------------------------------------------------------
TRINITY_DN11_c0_g1_i4           TTTCATATATTAAAACTGGTATGCCTGGCAGGAGTTACGTTAAAAGCGTGATGCCAAAAA
TRINITY_DN11_c0_g1_i1           ------------------------------------------------------------

MpDNV_DNA_RNA_seq_consensus     ATTATTGGCGTAATCCTAAAGAATTTCACTTAGCGCTTGATAAATTAGAGTTGCTCGTTA
TRINITY_DN2570_c0_g2_i1         ATTATTGGCGTAATCCTAAAGAATTTCACTTAGCGCTTGATAAATTAGAGTTGCTCGTTA
TRINITY_DN7_c0_g1_i2            ATTATTGGCGTAATCCTAAAGAATTTCACTTAGCGCTTGATAAATTAGAGTTGCTCGTTA
TRINITY_DN7_c0_g1_i1            ------------------------------------------------------------
TRINITY_DN11_c0_g1_i4           ATTATTGGCGTAATCCTAAAGAATTTCACTTAGCGCTTGATAAATTAGAGTTGCTCGTTA
TRINITY_DN11_c0_g1_i1           ------------------------------------------------------------

MpDNV_DNA_RNA_seq_consensus     AACGACATTTCGGTGATTTCACGTGTTTGGAAAATTTTTTACCGCAATATCGTTGGCATA
TRINITY_DN2570_c0_g2_i1         AACGACATTTCGGTGATTTCACGTGTTTGGAAAATTTTTTACCGCAATATCGTTGGCATA
```

```
TRINITY_DN7_c0_g1_i2           AACGACATTTCGGTGATTTCACGTGTTTGGAAAATTTTTTACCGCAATATCGTTGGCATA
TRINITY_DN7_c0_g1_i1           ------------------------------------------------------------
TRINITY_DN11_c0_g1_i4          AACGACATTTCGGTGATTTCACGTGTTTGGAAAATTTTTTACCGCAATATCGTTGGCATA
TRINITY_DN11_c0_g1_i1          ------------------------------------------------------------

MpDNV_DNA_RNA_seq_consensus    ATTGCATCACATGGTTTGTGAACGTGATGTCCTGTCCGTTGGACCCGTGGACTGTCGAAT
TRINITY_DN2570_c0_g2_i1        ATTGCATCACATGGTTTGTGAACGTGATGTCCTGTCCGTTGGACCCGTGGACTGTCGAAT
TRINITY_DN7_c0_g1_i2           ATTGCATCACATGGTTTGTGAACGTGATGTCCTGTCCGTTGGACCCGTGGACTGTCGAAT
TRINITY_DN7_c0_g1_i1           ------------------------------------------------------------
TRINITY_DN11_c0_g1_i4          ATTGCATCACATGGTTTGTGAACGTGATGTCCTGTCCGTTGGACCCGTGGACTGTCGAAT
TRINITY_DN11_c0_g1_i1          ------------------------------------------------------------

MpDNV_DNA_RNA_seq_consensus    GTGCTGGTTGTGGTTTTCAACATATTCCTAATATATCTAAATGCGAACTACTACGAGACT
TRINITY_DN2570_c0_g2_i1        GTGCTGGTTGTGGTTTTCAACATATTCCTAATATATCTAAATGCGAACTACTACGAGACT
TRINITY_DN7_c0_g1_i2           GTGCTGGTTGTGGTTTTCAACATATTCCTAATATATCTAAATGCGAACTACTACGAGACT
TRINITY_DN7_c0_g1_i1           ------------------------------------------------------------
TRINITY_DN11_c0_g1_i4          GTGCTGGTTGTGGTTTTCAACATATTCCTAATATATCTAAATGCGAACTACTACGAGACT
TRINITY_DN11_c0_g1_i1          ------------------------------------------------------------

MpDNV_DNA_RNA_seq_consensus    ATCCGTTCATGAACGAGGGCACTATAAATCATATACTAATCGAATGCATTAAAATTTACA
TRINITY_DN2570_c0_g2_i1        ATCCGTTCATGAACGAGGGCACTATAAATCATATACTAATCGAATGCATTAAAATTTACA
TRINITY_DN7_c0_g1_i2           ATCCGTTCATGAACGAGGGCACTATAAATCATATACTAATCGAATGCATTAAAATTTACA
TRINITY_DN7_c0_g1_i1           ------------------------------------------------------------
TRINITY_DN11_c0_g1_i4          ATCCGTTCATGAACGAGGGCACTATAAATCATATACTAATCGAATGCATTAAAATTTACA
TRINITY_DN11_c0_g1_i1          ------------------------------------------------------------

MpDNV_DNA_RNA_seq_consensus    AACAGGAAGGATCAAATACAGAATGTCGACCCGAACGATTGGTGGAAACATGCCTTCTGC 3’splice site
TRINITY_DN2570_c0_g2_i1        AACAGGAAGGATCAAATACAGAATGTCGACCCGAACGATTGGTGGAAACATGCCTTCTGC NS unspliced
TRINITY_DN7_c0_g1_i2           AACAGGAAGGATCAAATACAGAATGTCGACCCGAACGATTGGTGGAAACATGCCTTCTGC NS unspliced
TRINITY_DN7_c0_g1_i1           -----GAAGGATCAAATACAGAATGTCGACCCGAACGATTGGTGGAAACATGCCTTCTGC NS spliced
TRINITY_DN11_c0_g1_i4          AACAGGAAGGATCAAATACAGAATGTCGACCCGAACGATTGGTGGAAACATGCCTTCTGC NS unspliced
TRINITY_DN11_c0_g1_i1          -----GAAGGATCAAATACAGAATGTCGACCCGAACGATTGGTGGAAACATGCCTTCTGC NS spliced
                                    *******************************************************

MpDNV_DNA_RNA_seq_consensus    AGGGCTCGGGGGAGCTTCAATCTGAACAGTCCATACGTGAAAATGGCCAAAACCATACAA
TRINITY_DN2570_c0_g2_i1        AGGGCTCGGGGGAGCTTCAATCTGAACAGTCCATACGTGAAAATGGCCAAAACCATACAA
TRINITY_DN7_c0_g1_i2           AGGGCTCGGGGGAGCTTCAATCTGAACAGTCCATACGTGAAAATGGCCAAAACCATACAA
TRINITY_DN7_c0_g1_i1           AGGGCTCGGGGGAGCTTCAATCTGAACAGTCCATACGTGAAAATGGCCAAAACCATACAA
TRINITY_DN11_c0_g1_i4          AGGGCTCGGGGGAGCTTCAATCTGAACAGTCCATACGTGAAAATGGCCAAAACCATACAA
TRINITY_DN11_c0_g1_i1          AGGGCTCGGGGGAGCTTCAATCTGAACAGTCCATACGTGAAAATGGCCAAAACCATACAA
                               ************************************************************

MpDNV_DNA_RNA_seq_consensus    CAGGAAGTGTTTCGCAGGATGACCAAGATGGAGTACAAGATAACGGATCCACAAATGTTT
TRINITY_DN2570_c0_g2_i1        CAGGAAGTGTTTCGCAGGATGACCAAGATGGAGTACAAGATAACGGATCCACAAATGTTT
TRINITY_DN7_c0_g1_i2           CAGGAAGTGTTTCGCAGGATGACCAAGATGGAGTACAAGATAACGGATCCACAAATGTTT
TRINITY_DN7_c0_g1_i1           CAGGAAGTGTTTCGCAGGATGACCAAGATGGAGTACAAGATAACGGATCCACAAATGTTT
TRINITY_DN11_c0_g1_i4          CAGGAAGTGTTTCGCAGGATGACCAAGATGGAGTACAAGATAACGGATCCACAAATGTTT
TRINITY_DN11_c0_g1_i1          CAGGAAGTGTTTCGCAGGATGACCAAGATGGAGTACAAGATAACGGATCCACAAATGTTT
                               ************************************************************

MpDNV_DNA_RNA_seq_consensus    TTCAAGAAGAAGAAGAGGGCAGCCAAGAGGGCCCGCGGCAAAAGAAGTCGCGACGGAAGC
TRINITY_DN2570_c0_g2_i1        TTCAAGAAGAAGAAGAGGGCAGCCAAGAGGGCCCGCGGCAAAAGAAGTCGCGACGGAAGC
TRINITY_DN7_c0_g1_i2           TTCAAGAAGAAGAAGAGGGCAGCCAAGAGGGCCCGCGGCAAAAGAAGTCGCGACGGAAGC
TRINITY_DN7_c0_g1_i1           TTCAAGAAGAAGAAGAGGGCAGCCAAGAGGGCCCGCGGCAAAAGAAGTCGCGACGGAAGC
TRINITY_DN11_c0_g1_i4          TTCAAGAAGAAGAAGAGGGCAGCCAAGAGGGCCCGCGGCAAAAGAAGTCGCGACGGAAGC
TRINITY_DN11_c0_g1_i1          TTCAAGAAGAAGAAGAGGGCAGCCAAGAGGGCCCGCGGCAAAAGAAGTCGCGACGGAAGC
                               ************************************************************

MpDNV_DNA_RNA_seq_consensus    AGCGATTCCGAATGATTAGGGAAATAGATGTTAGTAAACCGCAATCTATCGAAGAACTTA
TRINITY_DN2570_c0_g2_i1        AGCGATTCCGAATGATTAGGGAAATAGATGTTAGTAAACCGCAATCTATCGAAGAACTTA
TRINITY_DN7_c0_g1_i2           AGCGATTCCGAATGATTAGGGAAATAGATGTTAGTAAACCGCAATCTATCGAAGAACTTA
TRINITY_DN7_c0_g1_i1           AGCGATTCCGAATGATTAGGGAAATAGATGTTAGTAAACCGCAATCTATCGAAGAACTTA
TRINITY_DN11_c0_g1_i4          AGCGATTCCGAATGATTAGGGAAATAGATGTTAGTAAACCGCAATCTATCGAAGAACTTA
TRINITY_DN11_c0_g1_i1          AGCGATTCCGAATGATTAGGGAAATAGATGTTAGTAAACCGCAATCTATCGAAGAACTTA
                               ************************************************************

MpDNV_DNA_RNA_seq_consensus    TATATAGGTATCCTTGTTGCCCCCCGGAAGCTTTCTATAACATTCCCGAATTTTACGCTA
TRINITY_DN2570_c0_g2_i1        TATATAGGTATCCTTGTTGCCCCCCGGAAGCTTTCTATAACATTCCCGAATTTTACGCTA
TRINITY_DN7_c0_g1_i2           TATATAGGTATCCTTGTTGCCCCCCGGAAGCTTTCTATAACATTCCCGAATTTTACGCTA
TRINITY_DN7_c0_g1_i1           TATATAGGTATCCTTGTTGCCCCCCGGAAGCTTTCTATAACATTCCCGAATTTTACGCTA
TRINITY_DN11_c0_g1_i4          TATATAGGTATCCTTGTTGCCCCCCGGAAGCTTTCTATAACATTCCCGAATTTTACGCTA
TRINITY_DN11_c0_g1_i1          TATATAGGTATCCTTGTTGCCCCCCGGAAGCTTTCTATAACATTCCCGAATTTTACGCTA
                               ************************************************************

MpDNV_DNA_RNA_seq_consensus    ATACAAATATAAACTGGATAGACATGAAAGATTTTAAAGTAACGATACCGTTACGTAATT
TRINITY_DN2570_c0_g2_i1        ATACAAATATAAACTGGATAGACATGAAAGATTTTAAAGTAACGATACCGTTACGTAATT
TRINITY_DN7_c0_g1_i2           ATACAAATATAAACTGGATAGACATGAAAGATTTTAAAGTAACGATACCGTTACGTAATT
TRINITY_DN7_c0_g1_i1           ATACAAATATAAACTGGATAGACATGAAAGATTTTAAAGTAACGATACCGTTACGTAATT
TRINITY_DN11_c0_g1_i4          ATACAAATATAAACTGGATAGACATGAAAGATTTTAAAGTAACGATACCGTTACGTAATT
TRINITY_DN11_c0_g1_i1          ATACAAATATAAACTGGATAGACATGAAAGATTTTAAAGTAACGATACCGTTACGTAATT
                               ************************************************************

MpDNV_DNA_RNA_seq_consensus    GGGCGGCCACTTTACGCAGATGGTCTATTCATGATTTCAATAAATATTATAATGACTCTA
TRINITY_DN2570_c0_g2_i1        GGGCGGCCACTTTACGCAGATGGTCTATTCATGATTTCAATAAATATTATAATGACTCTA
```

```
TRINITY_DN7_c0_g1_i2            GGGCGGCCACTTTACGCAGATGGTCTATTCATGATTTCAATAAATATTATAATGACTCTA
TRINITY_DN7_c0_g1_i1            GGGCGGCCACTTTACGCAGATGGTCTATTCATGATTTCAATAAATATTATAATGACTCTA
TRINITY_DN11_c0_g1_i4           GGGCGGCCACTTTACGCAGATGGTCTATTCATGATTTCAATAAATATTATAATGACTCTA
TRINITY_DN11_c0_g1_i1           GGGCGGCCACTTTACGCAGATGGTCTATTCATGATTTCAATAAATATTATAATGACTCTA
                                ************************************************************

MpDNV_DNA_RNA_seq_consensus     CAGTTTTTCCATATTTCAACGCGTACGGTTCAGATATTTGCAATATGTATTACAGTATTT
TRINITY_DN2570_c0_g2_i1         CAGTTTTTCCATATTTCAACGCGTACGGTTCAGATATTTGCAATATGTATTACAGTATTT
TRINITY_DN7_c0_g1_i2            CAGTTTTTCCATATTTCAACGCGTACGGTTCAGATATTTGCAATATGTATTACAGTATTT
TRINITY_DN7_c0_g1_i1            CAGTTTTTCCATATTTCAACGCGTACGGTTCAGATATTTGCAATATGTATTACAGTATTT
TRINITY_DN11_c0_g1_i4           CAGTTTTTCCATATTTCAACGCGTACGGTTCAGATATTTGCAATATGTATTACAGTATTT
TRINITY_DN11_c0_g1_i1           CAGTTTTTCCATATTTCAACGCGTACGGTTCAGATATTTGCAATATGTATTACAGTATTT
                                ************************************************************

MpDNV_DNA_RNA_seq_consensus     CGGAGAGTCTGACAATAGCAAAAGAATTACTCAACTATCAATTTGGCGATGACCCTGAAG
TRINITY_DN2570_c0_g2_i1         CGGAGAGTCTGACAATAGCAAAAGAATTACTCAACTATCAATTTGGCGATGACCCTGAAG
TRINITY_DN7_c0_g1_i2            CGGAGAGTCTGACAATAGCAAAAGAATTACTCAACTATCAATTTGGCGATGACCCTGAAG
TRINITY_DN7_c0_g1_i1            CGGAGAGTCTGACAATAGCAAAAGAATTACTCAACTATCAATTTGGCGATGACCCTGAAG
TRINITY_DN11_c0_g1_i4           CGGAGAGTCTGACAATAGCAAAAGAATTACTCAACTATCAATTTGGCGATGACCCTGAAG
TRINITY_DN11_c0_g1_i1           CGGAGAGTCTGACAATAGCAAAAGAATTACTCAACTATCAATTTGGCGATGACCCTGAAG
                                ************************************************************

MpDNV_DNA_RNA_seq_consensus     TAGTAATTGAATTTTTAACAACACTATATAATGTCATAGACAAAAGAGTTCCGAAATTAA
TRINITY_DN2570_c0_g2_i1         TAGTAATTGAATTTTTAACAACACTATATAATGTCATAGACAAAAGAGTTCCGAAATTAA
TRINITY_DN7_c0_g1_i2            TAGTAATTGAATTTTTAACAACACTATATAATGTCATAGACAAAAGAGTTCCGAAATTAA
TRINITY_DN7_c0_g1_i1            TAGTAATTGAATTTTTAACAACACTATATAATGTCATAGACAAAAGAGTTCCGAAATTAA
TRINITY_DN11_c0_g1_i4           TAGTAATTGAATTTTTAACAACACTATATAATGTCATAGACAAAAGAGTTCCGAAATTAA
TRINITY_DN11_c0_g1_i1           TAGTAATTGAATTTTTAACAACACTATATAATGTCATAGACAAAAGAGTTCCGAAATTAA
                                ************************************************************

MpDNV_DNA_RNA_seq_consensus     ATAGTATATGTATAAAAAGTCCCCCTTCGGCAGGTAAGAACTTTTTTTTCGATGCCGTTG
TRINITY_DN2570_c0_g2_i1         ATAGTATATGTATAAAAAGTCCCCCTTCGGCAGGTAAGAACTTTTTTTTCGATGCCGTTG
TRINITY_DN7_c0_g1_i2            ATAGTATATGTATAAAAAGTCCCCCTTCGGCAGGTAAGAACTTTTTTTTCGATGCCGTTG
TRINITY_DN7_c0_g1_i1            ATAGTATATGTATAAAAAGTCCCCCTTCGGCAGGTAAGAACTTTTTTTTCGATGCCGTTG
TRINITY_DN11_c0_g1_i4           ATAGTATATGTATAAAAAGTCCCCCTTCGGCAGGTAAGAACTTTTTTTTCGATGCCGTTG
TRINITY_DN11_c0_g1_i1           ATAGTATATGTATAAAAAGTCCCCCTTCGGCAGGTAAGAACTTTTTTTTCGATGCCGTTG
                                ************************************************************

MpDNV_DNA_RNA_seq_consensus     CATCGTATTTGCTTTCATATGGTATGTTCGGTACAGCCAATAAAAACAATAATTTTTCGT
TRINITY_DN2570_c0_g2_i1         CATCGTATTTGCTTTCATATGGTATGTTCGGTACAGCCAATAAAAACAATAATTTTTCGT
TRINITY_DN7_c0_g1_i2            CATCGTATTTGCTTTCATATGGTATGTTCGGTACAGCCAATAAAAACAATAATTTTTCGT
TRINITY_DN7_c0_g1_i1            CATCGTATTTGCTTTCATATGGTATGTTCGGTACAGCCAATAAAAACAATAATTTTTCGT
TRINITY_DN11_c0_g1_i4           CATCGTATTTGCTTTCATATGGCATGTTCGGTACAGCCAATAAAAACAATAATTTTTCGT
TRINITY_DN11_c0_g1_i1           CATCGTATTTGCTTTCATATGGCATGTTCGGTACAGCCAATAAAAACAATAATTTTTCGT
                                ********************** *************************************

MpDNV_DNA_RNA_seq_consensus     GGGCAGACGGAGCGGGTAAACGATTAGTTCTGTGGAACGAACCAAACTATGAACAATACC
TRINITY_DN2570_c0_g2_i1         GGGCAGACGGAGCGGGTAAACGATTAGTTCTGTGGAACGAACCAAACTACGAACAATACC
TRINITY_DN7_c0_g1_i2            GGGCAGACGGAGCGGGTAAACGATTAGTTCTGTGGAACGAACCAAACTATGAACAATACC
TRINITY_DN7_c0_g1_i1            GGGCAGACGGAGCGGGTAAACGATTAGTTCTGTGGAACGAACCAAACTATGAACAATACC
TRINITY_DN11_c0_g1_i4           GGGCAGACGGAGCGGGTAAACGATTAGTTCTGTGGAACGAACCAAACTATGAACAATACC
TRINITY_DN11_c0_g1_i1           GGGCAGACGGAGCGGGTAAACGATTAGTTCTGTGGAACGAACCAAACTATGAACAATACC
                                ************************************************* **********

MpDNV_DNA_RNA_seq_consensus     ATATAGAAAAAATAAAAGAACTTTTGGGGGGAGATACAACAAGAATACATGTCAAATATG
TRINITY_DN2570_c0_g2_i1         ATATAGAAAAAATAAAAGAACTTTTGGGGGGAGATACAACAAGAATACATGTCAAATATG
TRINITY_DN7_c0_g1_i2            ATATAGAAAAAATAAAAGAACTTTTGGGGGGAGATACAACAAGAATACATGTCAAATATG
TRINITY_DN7_c0_g1_i1            ATATAGAAAAAATAAAAGAACTTTTGGGGGGAGATACAACAAGAATACATGTCAAATATG
TRINITY_DN11_c0_g1_i4           ATATAGAAAAAATAAAAGAACTTTTGGGGGGAGATACAACAAGAATACATGTCAAATATG
TRINITY_DN11_c0_g1_i1           ATATAGAAAAAATAAAAGAACTTTTGGGGGGAGATACAACAAGAATACATGTCAAATATG
                                ************************************************************

MpDNV_DNA_RNA_seq_consensus     CAAACGACGTTAGTGTGCAAAGGGTACCCATTATTATATTAACAAATAACCACTTGAATA
TRINITY_DN2570_c0_g2_i1         CAAACGACGTTAGTGTGCAAAGGGTACCCATTATTATATTAACAAATAACCACTTGAATA
TRINITY_DN7_c0_g1_i2            CAAACGACGTTAGTGTGCAAAGGGTACCCATTATTATATTAACAAATAACCACTTGAATA
TRINITY_DN7_c0_g1_i1            CAAACGACGTTAGTGTGCAAAGGGTACCCATTATTATATTAACAAATAACCACTTGAATA
TRINITY_DN11_c0_g1_i4           CAAACGACGTTAGTGTGCAAAGGGTACCCATTATTATATTAACAAATAACCACTTGAATA
TRINITY_DN11_c0_g1_i1           CAAACGACGTTAGTGTGCAAAGGGTACCCATTATTATATTAACAAATAACCACTTGAATA
                                ************************************************************

MpDNV_DNA_RNA_seq_consensus     TTATTAGTCACCCAGCTTTTAACGATAGATTGCGGAGTTACGAATGGATGTCAGCTGGCT
TRINITY_DN2570_c0_g2_i1         TTATTAGTCACCCAGCTTTTAACGATAGATTGCGGAGTTACGAATGGATGTCAGCTGGCT
TRINITY_DN7_c0_g1_i2            TTATTAGTCACCCAGCTTTTAACGATAGATTGCGGAGTTACGAATGGATGTCAGCTGGCT
TRINITY_DN7_c0_g1_i1            TTATTAGTCACCCAGCTTTTAACGATAGATTGCGGAGTTACGAATGGATGTCAGCTGGCT
TRINITY_DN11_c0_g1_i4           TTATTAGTCACCCAGCTTTTAACGATAGATTGCGGAGTTACGAATGGATGTCAGCTGGCT
TRINITY_DN11_c0_g1_i1           TTATTAGTCACCCAGCTTTTAACGATAGATTGCGGAGTTACGAATGGATGTCAGCTGGCT
                                ************************************************************

MpDNV_DNA_RNA_seq_consensus     TCTTGAAGGACTACGATAAAAAACTGCATCCGCTTATGTTTTATGAATTACTTAAAGATT
TRINITY_DN2570_c0_g2_i1         TCTTGAAGGACTACGATAAAAAACTGCATCCGCTTATGTTTTATGAATTACTTAAAGATT
TRINITY_DN7_c0_g1_i2            TCTTGAAGGACTACGATAAAAAACTGCATCCGCTTATGTTTTATGAATTACTTAAAGATT
TRINITY_DN7_c0_g1_i1            TCTTGAAGGACTACGATAAAAAACTGCATCCGCTTATGTTTTATGAATTACTTAAAGATT
TRINITY_DN11_c0_g1_i4           TCTTGAAGGACTACGATAAAAAACTGCATCCGCTTATGTTTTATGAATTACTTAAAGATT
TRINITY_DN11_c0_g1_i1           TCTTGAAGGACTACGATAAAAAACTGCATCCGCTTATGTTTTATGAATTACTTAAAGATT
                                ************************************************************

MpDNV_DNA_RNA_seq_consensus     ATGGTGTAATAAATGATAACAATATGATTGATTAATATATATATATAatatTTGATTTTTAT NS poly(A)
TRINITY_DN2570_c0_g2_i1 APFV-13 ATGGTGTAATAAATGATAACAATATGATTGATTAATATATATATATAAAAAAAAAAAAA-- tail
```

```
TRINITY_DN7_c0_g1_i2    APFV-11  ATGGTGTAATAAATGATAACAATATGATTGATTAATATATATATATATA--TTGATTAAAAAAAAAAAAAAAAA
TRINITY_DN7_c0_g1_i1    APFV-11  ATGGTGTAATAAATGATAACAATATGATTGATTAATATATATATATATA--TTGATTAAAAAAAAAAAAAAAAA
TRINITY_DN11_c0_g1_i4   APFV-12  ATGGTGTAATAAATGATAACAATATGATTGATTAATATATATATATATA--TTGATTTTTAAAAAAAAAA
TRINITY_DN11_c0_g1_i1   APFV-12  ATGGTGTAATAAATGATAACAATATGATTGATTAATATATATATATATA--TTGATTTTTAAAAAAAAAA
                                 ***********************************************          NS poly(A) tails


MpDNV_DNA_RNA_seq_consensus      GTGGGCGGCCAGTACTTTGATGGAGGATTACGTAGTAGATATTCACATATATCAGTTAGT
TRINITY_DN2404_c0_g1_i4          ------------------------------------------------------------
TRINITY_DN2404_c0_g1_i2          ------------------------------------------------------------
TRINITY_DN2404_c0_g1_i1          ------------------------------------------------------------
TRINITY_DN2404_c0_g1_i3          ------------------------------------------------------------
TRINITY_DN2570_c0_g3_i4          --------------------------------------------------GGTG
TRINITY_DN2570_c0_g3_i3          --------------------------------------------------GGTG
TRINITY_DN2570_c0_g3_i2          --------------------------------------------------GGTG
TRINITY_DN2570_c0_g3_i1          --------------------------------------------------GGTG
TRINITY_DN32_c0_g1_i1            ------------------------------------------------------------
TRINITY_DN32_c0_g1_i2            ------------------------------------------------------------
TRINITY_DN32_c0_g1_i4            ------------------------------------------------------------
TRINITY_DN32_c0_g1_i3            ------------------------------------------------------------


MpDNV_DNA_RNA_seq_consensus     TGGTGTAATAAATGATAACAATATGATTGATTAATATATATATATAtataTTGATTTTTATTTA NS poly(A)
TRINITY_DN2404_c0_g1_i4 APFV-11     -------------TTTTTTTTTTTGATTAATATATATATATATATATATTGATTTTTATTTA tails
TRINITY_DN2404_c0_g1_i2             -------------TTTTTTTTTTTGATTAATATATATATATATATATATTGATTTTTATTTA
TRINITY_DN2404_c0_g1_i1             -------------TTTTTTTTTTTGATTAATATATATATATATATATATTGATTTTTATTTA
TRINITY_DN2404_c0_g1_i3             -------------TTTTTTTTTTTGATTAATATATATATATATATATATTGATTTTTATTTA
TRINITY_DN2570_c0_g3_i4 APFV-13 GTCAATAATTTGTTTTTTTTTTTTTTTTGATTAATATATATATATATATATATTGATTTTTATTTA
TRINITY_DN2570_c0_g3_i3         GTCAATAATTTGTTTTTTTTTTTTTTTTGATTAATATATATATATATATATATTGATTTTTATTTA
TRINITY_DN2570_c0_g3_i2         GTCAATAATTTGTTTTTTTTTTTTTTTTGATTAATATATATATATATATATATTGATTTTTATTTA
TRINITY_DN2570_c0_g3_i1         GTCAATAATTTGTTTTTTTTTTTTTTTTGATTAATATATATATATATATATATTGATTTTTATTTA
TRINITY_DN32_c0_g1_i1   APFV-12     -----------TTTTTTTTTTTTGATTAATATATATATATATATATATTGATTTTTATTTA
TRINITY_DN32_c0_g1_i2               -----------TTTTTTTTTTTTGATTAATATATATATATATATATATTGATTTTTATTTA
TRINITY_DN32_c0_g1_i4               -----------TTTTTTTTTTTTGATTAATATATATATATATATATATTGATTTTTATTTA
TRINITY_DN32_c0_g1_i3               -----------TTTTTTTTTTTTGATTAATATATATATATATATATATTGATTTTTATTTA
                                                           *************************************

MpDNV_DNA_RNA_seq_consensus      CGTAAAAAATCTACAGGTTCATTGACAATGGGTATATTCATTGACCTTCGCCACAATGTT
TRINITY_DN2404_c0_g1_i4          CGTAAAAAATCTACAGGTTCATTGACAATGGGTATATTCATTGACCTTCGCCACAATGTT
TRINITY_DN2404_c0_g1_i2          CGTAAAAAATCTACAGGTTCATTGACAATGGGTATATTCATTGACCTTCGCCACAATGTT
TRINITY_DN2404_c0_g1_i1          CGTAAAAAATCTACAGGTTCATTGACAATGGGTATATTCATTGACCTTCGCCACAATGTT
TRINITY_DN2404_c0_g1_i3          CGTAAAAAATCTACAGGTTCATTGACAATGGGTATATTCATTGACCTTCGCCACAATGTT
TRINITY_DN2570_c0_g3_i4          CGTAAAAAATCTACAGGTTCATTGACAATGGGTATATTCATTGACCTTCGCCACAATGTT
TRINITY_DN2570_c0_g3_i3          CGTAAAAAATCTACAGGTTCATTGACAATGGGTATATTCATTGACCTTCGCCACAATGTT
TRINITY_DN2570_c0_g3_i2          CGTAAAAAATCTACAGGTTCATTGACAATGGGTATATTCATTGACCTTCGCCACAATGTT
TRINITY_DN2570_c0_g3_i1          CGTAAAAAATCTACAGGTTCATTGACAATGGGTATATTCATTGACCTTCGCCACAATGTT
TRINITY_DN32_c0_g1_i1            CGTAAAAAATCTACAGGTTCATTGACAATGGGTATATTCATTGACCTTCGCCACAATGTT
TRINITY_DN32_c0_g1_i2            CGTAAAAAATCTACAGGTTCATTGACAATGGGTATATTCATTGACCTTCGCCACAATGTT
TRINITY_DN32_c0_g1_i4            CGTAAAAAATCTACAGGTTCATTGACAATGGGTATATTCATTGACCTTCGCCACAATGTT
TRINITY_DN32_c0_g1_i3            CGTAAAAAATCTACAGGTTCATTGACAATGGGTATATTCATTGACCTTCGCCACAATGTT
                                 ************************************************************

MpDNV_DNA_RNA_seq_consensus      AAATCACGGCAAAAAGCTAAATACGTTGAATATGACTTGAACAATCGTTCCTCTGGCCAC
TRINITY_DN2404_c0_g1_i4          AAATCACGGCAAAAAGCTAAATACGTTGAATATGACTTGAACAATCGTTCCTCTGGCCAC
TRINITY_DN2404_c0_g1_i2          AAATCACGGCAAAAAGCTAAATACGTTGAATATGACTTGAACAATCGTTCCTCTGGCCAC
TRINITY_DN2404_c0_g1_i1          AAATCACGGCAAAAAGCTAAATACGTTGAATATGACTTGAACAATCGTTCCTCTGGCCAC
TRINITY_DN2404_c0_g1_i3          AAATCACGGCAAAAAGCTAAATACGTTGAATATGACTTGAACAATCGTTCCTCTGGCCAC
TRINITY_DN2570_c0_g3_i4          AAATCACGGCAAAAAGCTAAATACGTTGAATATGACTTGAACAATCGTTCCTCTGGCCAC
TRINITY_DN2570_c0_g3_i3          AAATCACGGCAAAAAGCTAAATACGTTGAATATGACTTGAACAATCGTTCCTCTGGCCAC
TRINITY_DN2570_c0_g3_i2          AAATCACGGCAAAAAGCTAAATACGTTGAATATGACTTGAACAATCGTTCCTCTGGCCAC
TRINITY_DN2570_c0_g3_i1          AAATCACGGCAAAAAGCTAAATACGTTGAATATGACTTGAACAATCGTTCCTCTGGCCAC
TRINITY_DN32_c0_g1_i1            AAATCACGGCAAAAAGCTAAATACGTTGAATATGACTTGAACAATCGTTCCTCTGGCCAC
TRINITY_DN32_c0_g1_i2            AAATCACGGCAAAAAGCTAAATACGTTGAATATGACTTGAACAATCGTTCCTCTGGCCAC
TRINITY_DN32_c0_g1_i4            AAATCACGGCAAAAAGCTAAATACGTTGAATATGACTTGAACAATCGTTCCTCTGGCCAC
TRINITY_DN32_c0_g1_i3            AAATCACGGCAAAAAGCTAAATACGTTGAATATGACTTGAACAATCGTTCCTCTGGCCAC
                                 ************************************************************

MpDNV_DNA_RNA_seq_consensus      CAATTCGGATTATTGTAGTCGCCCCAAGAAGTGTTCATGATTTCTTCGGTTGTGGGTGGA
TRINITY_DN2404_c0_g1_i4          CAATTCGGATTATTGTAGTCGCCCCAAGAAGTGTTCATGATTTCTTCGGTTGTGGGTGGA
TRINITY_DN2404_c0_g1_i2          CAATTCGGATTATTGTAGTCGCCCCAAGAAGTGTTCATGATTTCTTCGGTTGTGGGTGGA
TRINITY_DN2404_c0_g1_i1          CAATTCGGATTATTGTAGTCGCCCCAAGAAGTGTTCATGATTTCTTCGGTTGTGGGTGGA
TRINITY_DN2404_c0_g1_i3          CAATTCGGATTATTGTAGTCGCCCCAAGAAGTGTTCATGATTTCTTCGGTTGTGGGTGGA
TRINITY_DN2570_c0_g3_i4          CAATTCGGATTATTGTAGTCGCCCCAAGAAGTGTTCATGATTTCTTCGGTTGTGGGTGGA
TRINITY_DN2570_c0_g3_i3          CAATTCGGATTATTGTAGTCGCCCCAAGAAGTGTTCATGATTTCTTCGGTTGTGGGTGGA
TRINITY_DN2570_c0_g3_i2          CAATTCGGATTATTGTAGTCGCCCCAAGAAGTGTTCATGATTTCTTCGGTTGTGGGTGGA
TRINITY_DN2570_c0_g3_i1          CAATTCGGATTATTGTAGTCGCCCCAAGAAGTGTTCATGATTTCTTCGGTTGTGGGTGGA
TRINITY_DN32_c0_g1_i1            CAATTCGGATTATTGTAGTCGCCCCAAGAAGTGTTCATGATTTCTTCGGTTGTGGGTGGA
TRINITY_DN32_c0_g1_i2            CAATTCGGATTATTGTAGTCGCCCCAAGAAGTGTTCATGATTTCTTCGGTTGTGGGTGGA
TRINITY_DN32_c0_g1_i4            CAATTCGGATTATTGTAGTCGCCCCAAGAAGTGTTCATGATTTCTTCGGTTGTGGGTGGA
TRINITY_DN32_c0_g1_i3            CAATTCGGATTATTGTAGTCGCCCCAAGAAGTGTTCATGATTTCTTCGGTTGTGGGTGGA
                                 ************************************************************

MpDNV_DNA_RNA_seq_consensus      GTAGTGGTATATCGATGCAAATTAATTAGGGTTTCTTTTGACGCACGATCACTTTCTCTG 3'splice site
TRINITY_DN2404_c0_g1_i4          GTAGTGGTATATCGATGCAAATTAATTAGGGTTTCTTTTGACGCACGATCACTTTCTCTG VP unspliced
TRINITY_DN2404_c0_g1_i2          GTAGTGGTATATCGATGCAAATTAATTAGGGTTTCTTTTGACGCACGATCACTTTCTCTG VP unspliced
TRINITY_DN2404_c0_g1_i1          GTAGTGGTATATCGATGCAAATTAATTAGGGTTTCTTTTGACGCACGATCACTTTCT---
TRINITY_DN2404_c0_g1_i3          GTAGTGGTATATCGATGCAAATTAATTAGGGTTTCTTTTGACGCACGATCACTTTCT--- VP spliced 2
TRINITY_DN2570_c0_g3_i4          GTAGTGGTATATCGATGCAAATTAATTAGGGTTTCTTTTGACGCACGATCACTTTCTCTG VP unspliced
```

```
TRINITY_DN2570_c0_g3_i3        GTAGTGGTATATCGATGCAAATTAATTAGGGTTTCTTTTGACGCACGATCACTTTCTCTG VP unspliced
TRINITY_DN2570_c0_g3_i2        GTAGTGGTATATCGATGCAAATTAATTAGGGTTTCTTTTGACGCACGATCACTTTCT--- VP spliced 2
TRINITY_DN2570_c0_g3_i1        GTAGTGGTATATCGATGCAAATTAATTAGGGTTTCTTTTGACGCACGATCACTTTCT--- VP spliced 2
TRINITY_DN32_c0_g1_i1          GTAGTGGTATATCGATGCAAATTAATTAGGGTTTCTTTTGACGCACGATCACTTTCTCTG
TRINITY_DN32_c0_g1_i2          GTAGTGGTATATCGATGCAAATTAATTAGGGTTTCTTTTGACGCACGATCACTTTCTCTG
TRINITY_DN32_c0_g1_i4          GTAGTGGTATATCGATGCAAATTAATTAGGGTTTCTTTTGACGCACGATCACTTTCT--- VP spliced 2
TRINITY_DN32_c0_g1_i3          GTAGTGGTATATCGATGCAAATTAATTAGGGTTTCTTTTGACGCACGATCACTTTCT--- VP spliced 2
                               *********************************************************

MpDNV_DNA_RNA_seq_consensus    TAAACATAATTGGGTATTTTAATAGGTGCTTTGACTCGAACCTCATGGATGTTTGACGCT
TRINITY_DN2404_c0_g1_i4        TAAACATAATTGGGTATTTTAATAGGTGCTTTGACTCGAACCTCATGGATGTTTGACGCT
TRINITY_DN2404_c0_g1_i2        TAAACATAATTGGGTATTTTAATAGGTGCTTTGACTCGAACCTCATGGATGTTTGACGCT
TRINITY_DN2404_c0_g1_i1        ------------------------------------------------------------
TRINITY_DN2404_c0_g1_i3        ------------------------------------------------------------
TRINITY_DN2570_c0_g3_i4        TAAACATAATTGGGTATTTTAATAGGTGCTTTGACTCGAACCTCATGGATGTTTGACGCT
TRINITY_DN2570_c0_g3_i3        TAAACATAATTGGGTATTTTAATAGGTGCTTTGACTCGAACCTCATGGATGTTTGACGCT
TRINITY_DN2570_c0_g3_i2        ------------------------------------------------------------
TRINITY_DN2570_c0_g3_i1        ------------------------------------------------------------
TRINITY_DN32_c0_g1_i1          TAAACATAATTGGGTATTTTAATAGGTGCTTTGACTCGAACCTCATGGATGTTTGACGCT
TRINITY_DN32_c0_g1_i2          TAAACATAATTGGGTATTTTAATAGGTGCTTTGACTCGAACCTCATGGATGTTTGACGCT
TRINITY_DN32_c0_g1_i4          ------------------------------------------------------------
TRINITY_DN32_c0_g1_i3          ------------------------------------------------------------

MpDNV_DNA_RNA_seq_consensus    TCAACCTCCATGGGGTCGTCGTCCATATTTACCCGAACAGACGTAGGTACAGGTGTAGTG 5'splice site
TRINITY_DN2404_c0_g1_i4        TCAACCTCCATGGGGTCGTCGTCCATATTTACCCGAACAGACGTAGGTACAGGTGTAGTG VP unspliced
TRINITY_DN2404_c0_g1_i2        TCAACCTCCATGGGGTCGTCGTCCATATTTACCCGAACAGACGTAGGTACAGGTGTAGTG VP unspliced
TRINITY_DN2404_c0_g1_i1        --------------------------------CCGAACAGACGTAGGTACAGGTGTAGTG VP spliced 2
TRINITY_DN2404_c0_g1_i3        --------------------------------CCGAACAGACGTAGGTACAGGTGTAGTG VP spliced 2
TRINITY_DN2570_c0_g3_i4        TCAACCTCCATGGGGTCGTCGTCCATATTTACCCGAACAGACGTAGGTACAGGTGTAGTG VP unspliced
TRINITY_DN2570_c0_g3_i3        TCAACCTCCATGGGGTCGTCGTCCATATTTACCCGAACAGACGTAGGTACAGGTGTAGTG VP unspliced
TRINITY_DN2570_c0_g3_i2        --------------------------------CCGAACAGACGTAGGTACAGGTGTAGTG VP spliced 2
TRINITY_DN2570_c0_g3_i1        --------------------------------CCGAACAGACGTAGGTACAGGTGTAGTG VP spliced 2
TRINITY_DN32_c0_g1_i1          TCAACCTCCATGGGGTCGTCGTCCATATTTACCCGAACAGACGTAGGTACAGGTGTAGTG VP unspliced
TRINITY_DN32_c0_g1_i2          TCAACCTCCATGGGGTCGTCGTCCATATTTACCCGAACAGACGTAGGTACAGGTGTAGTG VP unspliced
TRINITY_DN32_c0_g1_i4          --------------------------------CCGAACAGACGTAGGTACAGGTGTAGTG VP spliced 2
TRINITY_DN32_c0_g1_i3          --------------------------------CCGAACAGACGTAGGTACAGGTGTAGTG VP spliced 2
                                                               ****************************

MpDNV_DNA_RNA_seq_consensus    ACAGTTGCGTCGACTGGAGTAGTAAATGAATCAATGTTCTTTGTAGTGGAAGCTTTGCCC
TRINITY_DN2404_c0_g1_i4        ACAGTTGCGTCGACTGGAGTAGTAAATGAATCAATGTTCTTTGTAGTGGAAGCTTTGCCC
TRINITY_DN2404_c0_g1_i2        ACAGTTGCGTCGACTGGAGTAGTAAATGAATCAATGTTCTTTGTAGTGGAAGCTTTGCCC
TRINITY_DN2404_c0_g1_i1        ACAGTTGCGTCGACTGGAGTAGTAAATGAATCAATGTTCTTTGTAGTGGAAGCTTTGCCC
TRINITY_DN2404_c0_g1_i3        ACAGTTGCGTCGACTGGAGTAGTAAATGAATCAATGTTCTTTGTAGTGGAAGCTTTGCCC
TRINITY_DN2570_c0_g3_i4        ACAGTTGCGTCGACTGGAGTAGTAAATGAATCAATGTTCTTTGTAGTGGAAGCTTTGCCC
TRINITY_DN2570_c0_g3_i3        ACAGTTGCGTCGACTGGAGTAGTAAATGAATCAATGTTCTTTGTAGTGGAAGCTTTGCCC
TRINITY_DN2570_c0_g3_i2        ACAGTTGCGTCGACTGGAGTAGTAAATGAATCAATGTTCTTTGTAGTGGAAGCTTTGCCC
TRINITY_DN2570_c0_g3_i1        ACAGTTGCGTCGACTGGAGTAGTAAATGAATCAATGTTCTTTGTAGTGGAAGCTTTGCCC
TRINITY_DN32_c0_g1_i1          ACAGTTGCGTCGACTGGAGTAGTAAATGAATCAATGTTCTTTGTAGTGGAAGCTTTGCCC
TRINITY_DN32_c0_g1_i2          ACAGTTGCGTCGACTGGAGTAGTAAATGAATCAATGTTCTTTGTAGTGGAAGCTTTGCCC
TRINITY_DN32_c0_g1_i4          ACAGTTGCGTCGACTGGAGTAGTAAATGAATCAATGTTCTTTGTAGTGGAAGCTTTGCCC
TRINITY_DN32_c0_g1_i3          ACAGTTGCGTCGACTGGAGTAGTAAATGAATCAATGTTCTTTGTAGTGGAAGCTTTGCCC
                               ************************************************************

MpDNV_DNA_RNA_seq_consensus    ATTTCTTTAGTTAGTCCCGAAGAGTGTGCAGCGGCGTTTTGATTGTCAGCTACTTTTTCA
TRINITY_DN2404_c0_g1_i4        ATTTCTTTAGTTAGTCCCGAAGAGTGTGCAGCGGCGTTTTGATTGTCAGCTACTTTTTCA
TRINITY_DN2404_c0_g1_i2        ATTTCTTTAGTTAGTCCCGAAGAGTGTGCAGCGGCGTTTTGATTGTCAGCTACTTTTTCA
TRINITY_DN2404_c0_g1_i1        ATTTCTTTAGTTAGTCCCGAAGAGTGTGCAGCGGCGTTTTGATTGTCAGCTACTTTTTCA
TRINITY_DN2404_c0_g1_i3        ATTTCTTTAGTTAGTCCCGAAGAGTGTGCAGCGGCGTTTTGATTGTCAGCTACTTTTTCA
TRINITY_DN2570_c0_g3_i4        ATTTCTTTAGTTAGTCCCGAAGAGTGTGCAGCGGCGTTTTGATTGTCAGCTACTTTTTCA
TRINITY_DN2570_c0_g3_i3        ATTTCTTTAGTTAGTCCCGAAGAGTGTGCAGCGGCGTTTTGATTGTCAGCTACTTTTTCA
TRINITY_DN2570_c0_g3_i2        ATTTCTTTAGTTAGTCCCGAAGAGTGTGCAGCGGCGTTTTGATTGTCAGCTACTTTTTCA
TRINITY_DN2570_c0_g3_i1        ATTTCTTTAGTTAGTCCCGAAGAGTGTGCAGCGGCGTTTTGATTGTCAGCTACTTTTTCA
TRINITY_DN32_c0_g1_i1          ATTTCTTTAGTTAGTCCCGAAGAGTGTGCAGCGGCGTTTTGATTGTCAGCTACTTTTTCA
TRINITY_DN32_c0_g1_i2          ATTTCTTTAGTTAGTCCCGAAGAGTGTGCAGCGGCGTTTTGATTGTCAGCTACTTTTTCA
TRINITY_DN32_c0_g1_i4          ATTTCTTTAGTTAGTCCCGAAGAGTGTGCAGCGGCGTTTTGATTGTCAGCTACTTTTTCA
TRINITY_DN32_c0_g1_i3          ATTTCTTTAGTTAGTCCCGAAGAGTGTGCAGCGGCGTTTTGATTGTCAGCTACTTTTTCA
                               ************************************************************

MpDNV_DNA_RNA_seq_consensus    GGAGCCACGTACAATCCGTGAACAATAGATAAATCTCCACGATTGTATAGATTTTGATTG
TRINITY_DN2404_c0_g1_i4        GGAGCCACGTACAATCCGTGAACAATAGATAAATCTCCACGATTGTATAGATTTTGATTG
TRINITY_DN2404_c0_g1_i2        GGAGCCACGTACAATCCGTGAACAATAGATAAATCTCCACGATTGTATAGATTTTGATTG
TRINITY_DN2404_c0_g1_i1        GGAGCCACGTACAATCCGTGAACAATAGATAAATCTCCACGATTGTATAGATTTTGATTG
TRINITY_DN2404_c0_g1_i3        GGAGCCACGTACAATCCGTGAACAATAGATAAATCTCCACGATTGTATAGATTTTGATTG
TRINITY_DN2570_c0_g3_i4        GGAGCCACGTACAATCCGTGAACAATAGATAAATCTCCACGATTGTATAGATTTTGATTG
TRINITY_DN2570_c0_g3_i3        GGAGCCACGTACAATCCGTGAACAATAGATAAATCTCCACGATTGTATAGATTTTGATTG
TRINITY_DN2570_c0_g3_i2        GGAGCCACGTACAATCCGTGAACAATAGATAAATCTCCACGATTGTATAGATTTTGATTG
TRINITY_DN2570_c0_g3_i1        GGAGCCACGTACAATCCGTGAACAATAGATAAATCTCCACGATTGTATAGATTTTGATTG
TRINITY_DN32_c0_g1_i1          GGAGCCACGTACAATCCGTGAACAATAGATAAATCTCCACGATTGTATAGATTTTGATTG
TRINITY_DN32_c0_g1_i2          GGAGCCACGTACAATCCGTGAACAATAGATAAATCTCCACGATTGTATAGATTTTGATTG
TRINITY_DN32_c0_g1_i4          GGAGCCACGTACAATCCGTGAACAATAGATAAATCTCCACGATTGTATAGATTTTGATTG
TRINITY_DN32_c0_g1_i3          GGAGCCACGTACAATCCGTGAACAATAGATAAATCTCCACGATTGTATAGATTTTGATTG
                               ************************************************************

MpDNV_DNA_RNA_seq_consensus    TAATTGTCGTCGGCCAGCAACCATGTCTTTTCGTATTCCACGTGAAGCCTGTCAAACGCT
TRINITY_DN2404_c0_g1_i4        TAATTGTCGTCGGCCAGCAACCATGTCTTTTCGTATTCCACGTGAAGCCTGTCAAACGCT
TRINITY_DN2404_c0_g1_i2        TAATTGTCGTCGGCCAGCAACCATGTCTTTTCGTATTCCACGTGAAGCCTGTCAAACGCT
TRINITY_DN2404_c0_g1_i1        TAATTGTCGTCGGCCAGCAACCATGTCTTTTCGTATTCCACGTGAAGCCTGTCAAACGCT
```

```
TRINITY_DN2404_c0_g1_i3         TAATTGTCGTCGGCCAGCAACCATGTCTTTTCGTATTCCACGTGAAGCCTGTCAAACGCT
TRINITY_DN2570_c0_g3_i4         TAATTGTCGTCGGCCAGCAACCATGTCTTTTCGTATTCCACGTGAAGCCTGTCAAACGCT
TRINITY_DN2570_c0_g3_i3         TAATTGTCGTCGGCCAGCAACCATGTCTTTTCGTATTCCACGTGAAGCCTGTCAAACGCT
TRINITY_DN2570_c0_g3_i2         TAATTGTCGTCGGCCAGCAACCATGTCTTTTCGTATTCCACGTGAAGCCTGTCAAACGCT
TRINITY_DN2570_c0_g3_i1         TAATTGTCGTCGGCCAGCAACCATGTCTTTTCGTATTCCACGTGAAGCCTGTCAAACGCT
TRINITY_DN32_c0_g1_i1           TAATTGTCGTCGGCCAGCAACCATGTCTTTTCGTATTCCACGTGAAGCCTGTCAAACGCT
TRINITY_DN32_c0_g1_i2           TAATTGTCGTCGGCCAGCAACCATGTCTTTTCGTATTCCACGTGAAGCCTGTCAAACGCT
TRINITY_DN32_c0_g1_i4           TAATTGTCGTCGGCCAGCAACCATGTCTTTTCGTATTCCACGTGAAGCCTGTCAAACGCT
TRINITY_DN32_c0_g1_i3           TAATTGTCGTCGGCCAGCAACCATGTCTTTTCGTATTCCACGTGAAGCCTGTCAAACGCT
                                ************************************************************

MpDNV_DNA_RNA_seq_consensus     GTTCTGCTTTGCGGACATCCAAAACTAACTGTCATCTCAGTTTCGAAATCCCATGTGCAT
TRINITY_DN2404_c0_g1_i4         GTTCTGCTTTGCGGACATCCAAAACTAACTGTCATCTCAGTTTCGAAATCCCATGTGCAT
TRINITY_DN2404_c0_g1_i2         GTTCTGCTTTGCGGACATCCAAAACTAACTGTCATCTCAGTTTCGAAATCCCATGTGCAT
TRINITY_DN2404_c0_g1_i1         GTTCTGCTTTGCGGACATCCAAAACTAACTGTCATCTCAGTTTCGAAATCCCATGTGCAT
TRINITY_DN2404_c0_g1_i3         GTTCTGCTTTGCGGACATCCAAAACTAACTGTCATCTCAGTTTCGAAATCCCATGTGCAT
TRINITY_DN2570_c0_g3_i4         GTTCTGCTTTGCGGACATCCAAAACTAACTGTCATCTCAGTTTCGAAATCCCATGTGCAT
TRINITY_DN2570_c0_g3_i3         GTTCTGCTTTGCGGACATCCAAAACTAACTGTCATCTCAGTTTCGAAATCCCATGTGCAT
TRINITY_DN2570_c0_g3_i2         GTTCTGCTTTGCGGACATCCAAAACTAACTGTCATCTCAGTTTCGAAATCCCATGTGCAT
TRINITY_DN2570_c0_g3_i1         GTTCTGCTTTGCGGACATCCAAAACTAACTGTCATCTCAGTTTCGAAATCCCATGTGCAT
TRINITY_DN32_c0_g1_i1           GTTCTGCTTTGCGGACATCCAAAACTAACTGTCATCTCAGTTTCGAAATCCCATGTGCAT
TRINITY_DN32_c0_g1_i2           GTTCTGCTTTGCGGACATCCAAAACTAACTGTCATCTCAGTTTCGAAATCCCATGTGCAT
TRINITY_DN32_c0_g1_i4           GTTCTGCTTTGCGGACATCCAAAACTAACTGTCATCTCAGTTTCGAAATCCCATGTGCAT
TRINITY_DN32_c0_g1_i3           GTTCTGCTTTGCGGACATCCAAAACTAACTGTCATCTCAGTTTCGAAATCCCATGTGCAT
                                ************************************************************

MpDNV_DNA_RNA_seq_consensus     TCTACGTCAGTAAAATTTACAGGGACTATACTATTAGTGGCAGTAGTTAACTTATGCACT
TRINITY_DN2404_c0_g1_i4         TCTACGTCAGTAAAATTTACAGGGACTATACTATTAGTGGCAGTAGTTAACTTATGCACT
TRINITY_DN2404_c0_g1_i2         TCTACGTCAGTAAAATTTACAGGGACTATACTATTAGTGGCAGTAGTTAACTTATGCACT
TRINITY_DN2404_c0_g1_i1         TCTACGTCAGTAAAATTTACAGGGACTATACTATTAGTGGCAGTAGTTAACTTATGCACT
TRINITY_DN2404_c0_g1_i3         TCTACGTCAGTAAAATTTACAGGGACTATACTATTAGTGGCAGTAGTTAACTTATGCACT
TRINITY_DN2570_c0_g3_i4         TCTACGTCAGTAAAATTTACAGGGACTATACTATTAGTGGCAGTAGTTAACTTATGCACT
TRINITY_DN2570_c0_g3_i3         TCTACGTCAGTAAAATTTACAGGGACTATACTATTAGTGGCAGTAGTTAACTTATGCACT
TRINITY_DN2570_c0_g3_i2         TCTACGTCAGTAAAATTTACAGGGACTATACTATTAGTGGCAGTAGTTAACTTATGCACT
TRINITY_DN2570_c0_g3_i1         TCTACGTCAGTAAAATTTACAGGGACTATACTATTAGTGGCAGTAGTTAACTTATGCACT
TRINITY_DN32_c0_g1_i1           TCTACGTCAGTAAAATTTACAGGGACTATACTATTAGTGGCAGTAGTTAACTTATGCACT
TRINITY_DN32_c0_g1_i2           TCTACGTCAGTAAAATTTACAGGGACTATACTATTAGTGGCAGTAGTTAACTTATGCACT
TRINITY_DN32_c0_g1_i4           TCTACGTCAGTAAAATTTACAGGGACTATACTATTAGTGGCAGTAGTTAACTTATGCACT
TRINITY_DN32_c0_g1_i3           TCTACGTCAGTAAAATTTACAGGGACTATACTATTAGTGGCAGTAGTTAACTTATGCACT
                                ************************************************************

MpDNV_DNA_RNA_seq_consensus     GGATATATTCCTACATGTACACTAGGTTGTACTTTATTAGAATATTCATGTTTATCGAAC
TRINITY_DN2404_c0_g1_i4         GGATATATTCCTACATGTACACTAGGTTGTACTTTATTAGAATATTCATGTTTATCGAAC
TRINITY_DN2404_c0_g1_i2         GGATATATTCCTACATGTACACTAGGTTGTACTTTATTAGAATATTCATGTTTATCGAAC
TRINITY_DN2404_c0_g1_i1         GGATATATTCCTACATGTACACTAGGTTGTACTTTATTAGAATATTCATGTTTATCGAAC
TRINITY_DN2404_c0_g1_i3         GGATATATTCCTACATGTACACTAGGTTGTACTTTATTAGAATATTCATGTTTATCGAAC
TRINITY_DN2570_c0_g3_i4         GGATATATTCCTACATGTACACTAGGTTGTACTTTATTAGAATATTCATGTTTATCGAAC
TRINITY_DN2570_c0_g3_i3         GGATATATTCCTACATGTACACTAGGTTGTACTTTATTAGAATATTCATGTTTATCGAAC
TRINITY_DN2570_c0_g3_i2         GGATATATTCCTACATGTACACTAGGTTGTACTTTATTAGAATATTCATGTTTATCGAAC
TRINITY_DN2570_c0_g3_i1         GGATATATTCCTACATGTACACTAGGTTGTACTTTATTAGAATATTCATGTTTATCGAAC
TRINITY_DN32_c0_g1_i1           GGATATATTCCTACATGTACACTAGGTTGTACTTTATTAGAATATTCATGTTTATCGAAC
TRINITY_DN32_c0_g1_i2           GGATATATTCCTACATGTACACTAGGTTGTACTTTATTAGAATATTCATGTTTATCGAAC
TRINITY_DN32_c0_g1_i4           GGATATATTCCTACATGTACACTAGGTTGTACTTTATTAGAATATTCATGTTTATCGAAC
TRINITY_DN32_c0_g1_i3           GGATATATTCCTACATGTACACTAGGTTGTACTTTATTAGAATATTCATGTTTATCGAAC
                                ************************************************************

MpDNV_DNA_RNA_seq_consensus     ATACGAAAATATTGGCCTACGTCTATGTTAGTACAATATCTATCATTGCCGAATATGCGT
TRINITY_DN2404_c0_g1_i4         ATACGAAAATATTGGCCTACGTCTATGTTAGTACAATATCTATCATTGCCGAATATGCGT
TRINITY_DN2404_c0_g1_i2         ATACGAAAATATTGGCCTACGTCTATGTTAGTACAATATCTATCATTGCCGAATATGCGT
TRINITY_DN2404_c0_g1_i1         ATACGAAAATATTGGCCTACGTCTATGTTAGTACAATATCTATCATTGCCGAATATGCGT
TRINITY_DN2404_c0_g1_i3         ATACGAAAATATTGGCCTACGTCTATGTTAGTACAATATCTATCATTGCCGAATATGCGT
TRINITY_DN2570_c0_g3_i4         ATACGAAAATATTGGCCTACGTCTATGTTAGTACAATATCTATCATTGCCGAATATGCGT
TRINITY_DN2570_c0_g3_i3         ATACGAAAATATTGGCCTACGTCTATGTTAGTACAATATCTATCATTGCCGAATATGCGT
TRINITY_DN2570_c0_g3_i2         ATACGAAAATATTGGCCTACGTCTATGTTAGTACAATATCTATCATTGCCGAATATGCGT
TRINITY_DN2570_c0_g3_i1         ATACGAAAATATTGGCCTACGTCTATGTTAGTACAATATCTATCATTGCCGAATATGCGT
TRINITY_DN32_c0_g1_i1           ATACGAAAATATTGGCCTACGTCTATGTTAGTACAATATCTATCATTGCCGAATATGCGT
TRINITY_DN32_c0_g1_i2           ATACGAAAATATTGGCCTACGTCTATGTTAGTACAATATCTATCATTGCCGAATATGCGT
TRINITY_DN32_c0_g1_i4           ATACGAAAATATTGGCCTACGTCTATGTTAGTACAATATCTATCATTGCCGAATATGCGT
TRINITY_DN32_c0_g1_i3           ATACGAAAATATTGGCCTACGTCTATGTTAGTACAATATCTATCATTGCCGAATATGCGT
                                ************************************************************

MpDNV_DNA_RNA_seq_consensus     TTCCAACCGTTTTCACCCACAAATGTCAATTTTTGATCCGATCTCCTTTCGAACAAGCCA
TRINITY_DN2404_c0_g1_i4         TTCCAACCGTTTTCACCCACAAATGTCAATTTTTGATCCGATCTCCTTTCGAACAAGCCA
TRINITY_DN2404_c0_g1_i2         TTCCAACCGTTTTCACCCACAAATGTCAATTTTTGATCCGATCTCCTTTCGAACAAGCCA
TRINITY_DN2404_c0_g1_i1         TTCCAACCGTTTTCACCCACAAATGTCAATTTTTGATCCGATCTCCTTTCGAACAAGCCA
TRINITY_DN2404_c0_g1_i3         TTCCAACCGTTTTCACCCACAAATGTCAATTTTTGATCCGATCTCCTTTCGAACAAGCCA
TRINITY_DN2570_c0_g3_i4         TTCCAACCGTTTTCACCCACAAATGTCAATTTTTGATCCGATCTCCTTTCGAACAAGCCA
TRINITY_DN2570_c0_g3_i3         TTCCAACCGTTTTCACCCACAAATGTCAATTTTTGATCCGATCTCCTTTCGAACAAGCCA
TRINITY_DN2570_c0_g3_i2         TTCCAACCGTTTTCACCCACAAATGTCAATTTTTGATCCGATCTCCTTTCGAACAAGCCA
TRINITY_DN2570_c0_g3_i1         TTCCAACCGTTTTCACCCACAAATGTCAATTTTTGATCCGATCTCCTTTCGAACAAGCCA
TRINITY_DN32_c0_g1_i1           TTCCAACCGTTTTCACCCACAAATGTCAATTTTTGATCCGATCTCCTTTCGAACAAGCCA
TRINITY_DN32_c0_g1_i2           TTCCAACCGTTTTCACCCACAAATGTCAATTTTTGATCCGATCTCCTTTCGAACAAGCCA
TRINITY_DN32_c0_g1_i4           TTCCAACCGTTTTCACCCACAAATGTCAATTTTTGATCCGATCTCCTTTCGAACAAGCCA
TRINITY_DN32_c0_g1_i3           TTCCAACCGTTTTCACCCACAAATGTCAATTTTTGATCCGATCTCCTTTCGAACAAGCCA
                                ************************************************************

MpDNV_DNA_RNA_seq_consensus     CTGGCAGCGTTTATAAAATCTTGACACTCTGGTTCGTTACCAGCATTGCGTAACATAGCA
TRINITY_DN2404_c0_g1_i4         CTGGCAGCGTTTATAAAATCTTGACACTCTGGTTCGTTACCAGCATTGCGTAACATAGCA
```

```
TRINITY_DN2404_c0_g1_i2          CTGGCAGCGTTTATAAAATCTTGACACTCTGGTTCGTTACCAGCATTGCGTAACATAGCA
TRINITY_DN2404_c0_g1_i1          CTGGCAGCGTTTATAAAATCTTGACACTCTGGTTCGTTACCAGCATTGCGTAACATAGCA
TRINITY_DN2404_c0_g1_i3          CTGGCAGCGTTTATAAAATCTTGACACTCTGGTTCGTTACCAGCATTGCGTAACATAGCA
TRINITY_DN2570_c0_g3_i4          CTGGCAGCGTTTATAAAATCTTGACACTCTGGTTCGTTACCAGCATTGCGTAACATAGCA
TRINITY_DN2570_c0_g3_i3          CTGGCAGCGTTTATAAAATCTTGACACTCTGGTTCGTTACCAGCATTGCGTAACATAGCA
TRINITY_DN2570_c0_g3_i2          CTGGCAGCGTTTATAAAATCTTGACACTCTGGTTCGTTACCAGCATTGCGTAACATAGCA
TRINITY_DN2570_c0_g3_i1          CTGGCAGCGTTTATAAAATCTTGACACTCTGGTTCGTTACCAGCATTGCGTAACATAGCA
TRINITY_DN32_c0_g1_i1            CTGGCAGCGTTTATAAAATCTTGACACTCTGGTTCGTTACCAGCATTGCGTAACATAGCA
TRINITY_DN32_c0_g1_i2            CTGGCAGCGTTTATAAAATCTTGACACTCTGGTTCGTTACCAGCATTGCGTAACATAGCA
TRINITY_DN32_c0_g1_i4            CTGGCAGCGTTTATAAAATCTTGACACTCTGGTTCGTTACCAGCATTGCGTAACATAGCA
TRINITY_DN32_c0_g1_i3            CTGGCAGCGTTTATAAAATCTTGACACTCTGGTTCGTTACCAGCATTGCGTAACATAGCA
                                 ************************************************************

MpDNV_DNA_RNA_seq_consensus      AATTTACTGGAATTAGGCTCCTGTACGCTGACACACGCATCGAATATTGGATCGTGTGGT
TRINITY_DN2404_c0_g1_i4          AATTTACTGGAATTAGGCTCCTGTACGCTGACACACGCATCGAATATTGGATCGTGTGGT
TRINITY_DN2404_c0_g1_i2          AATTTACTGGAATTAGGCTCCTGTACGCTGACACACGCATCGAATATTGGATCGTGTGGT
TRINITY_DN2404_c0_g1_i1          AATTTACTGGAATTAGGCTCCTGTACGCTGACACACGCATCGAATATTGGATCGTGTGGT
TRINITY_DN2404_c0_g1_i3          AATTTACTGGAATTAGGCTCCTGTACGCTGACACACGCATCGAATATTGGATCGTGTGGT
TRINITY_DN2570_c0_g3_i4          AATTTACTGGAATTAGGCTCCTGTACGCTGACACACGCATCGAATATTGGATCGTGTGGT
TRINITY_DN2570_c0_g3_i3          AATTTACTGGAATTAGGCTCCTGTACGCTGACACACGCATCGAATATTGGATCGTGTGGT
TRINITY_DN2570_c0_g3_i2          AATTTACTGGAATTAGGCTCCTGTACGCTGACACACGCATCGAATATTGGATCGTGTGGT
TRINITY_DN2570_c0_g3_i1          AATTTACTGGAATTAGGCTCCTGTACGCTGACACACGCATCGAATATTGGATCGTGTGGT
TRINITY_DN32_c0_g1_i1            AATTTACTGGAATTAGGCTCCTGTACGCTGACACACGCATCGAATATTGGATCGTGTGGT
TRINITY_DN32_c0_g1_i2            AATTTACTGGAATTAGGCTCCTGTACGCTGACACACGCATCGAATATTGGATCGTGTGGT
TRINITY_DN32_c0_g1_i4            AATTTACTGGAATTAGGCTCCTGTACGCTGACACACGCATCGAATATTGGATCGTGTGGT
TRINITY_DN32_c0_g1_i3            AATTTACTGGAATTAGGCTCCTGTACGCTGACACACGCATCGAATATTGGATCGTGTGGT
                                 ************************************************************

MpDNV_DNA_RNA_seq_consensus      AGAGTGAGTATACCGACATGAGGCTTGTACGAATAATTAAGTACAGTGGTGCCGACAGTA
TRINITY_DN2404_c0_g1_i4          AGAGTGAGTATACCGACATGAGGCTTGTACGAATAATTAAGTACAGTGGTGCCGACAGTA
TRINITY_DN2404_c0_g1_i2          AGAGTGAGTATACCGACATGAGGCTTGTACGAATAATTAAGTACAGTGGTGCCGACAGTA
TRINITY_DN2404_c0_g1_i1          AGAGTGAGTATACCGACATGAGGCTTGTACGAATAATTAAGTACAGTGGTGCCGACAGTA
TRINITY_DN2404_c0_g1_i3          AGAGTGAGTATACCGACATGAGGCTTGTACGAATAATTAAGTACAGTGGTGCCGACAGTA
TRINITY_DN2570_c0_g3_i4          AGAGTGAGTATACCGACATGAGGCTTGTACGAATAATTAAGTACAGTGGTGCCGACAGTA
TRINITY_DN2570_c0_g3_i3          AGAGTGAGTATACCGACATGAGGCTTGTACGAATAATTAAGTACAGTGGTGCCGACAGTA
TRINITY_DN2570_c0_g3_i2          AGAGTGAGTATACCGACATGAGGCTTGTACGAATAATTAAGTACAGTGGTGCCGACAGTA
TRINITY_DN2570_c0_g3_i1          AGAGTGAGTATACCGACATGAGGCTTGTACGAATAATTAAGTACAGTGGTGCCGACAGTA
TRINITY_DN32_c0_g1_i1            AGAGTGAGTATACCGACATGAGGCTTGTACGAATAATTAAGTACAGTGGTGCCGACAGTA
TRINITY_DN32_c0_g1_i2            AGAGTGAGTATACCGACATGAGGCTTGTACGAATAATTAAGTACAGTGGTGCCGACAGTA
TRINITY_DN32_c0_g1_i4            AGAGTGAGTATACCGACATGAGGCTTGTACGAATAATTAAGTACAGTGGTGCCGACAGTA
TRINITY_DN32_c0_g1_i3            AGAGTGAGTATACCGACATGAGGCTTGTACGAATAATTAAGTACAGTGGTGCCGACAGTA
                                 ************************************************************

MpDNV_DNA_RNA_seq_consensus      AACGAAGCGTCGACCTTGGTGATGTGTTCATTAAACCGTTCCCATCCGAGTTTTCCAGAA
TRINITY_DN2404_c0_g1_i4          AACGAAGCGTCGACCTTGGTGATGTGTTCATTAAACCGTTCCCATCCGAGTTTTCCAGAA
TRINITY_DN2404_c0_g1_i2          AACGAAGCGTCGACCTTGGTGATGTGTTCATTAAACCGTTCCCATCCGAGTTTTCCAGAA
TRINITY_DN2404_c0_g1_i1          AACGAAGCGTCGACCTTGGTGATGTGTTCATTAAACCGTTCCCATCCGAGTTTTCCAGAA
TRINITY_DN2404_c0_g1_i3          AACGAAGCGTCGACCTTGGTGATGTGTTCATTAAACCGTTCCCATCCGAGTTTTCCAGAA
TRINITY_DN2570_c0_g3_i4          AACGAAGCGTCGACCTTGGTGATGTGTTCATTAAACCGTTCCCATCCGAGTTTTCCAGAA
TRINITY_DN2570_c0_g3_i3          AACGAAGCGTCGACCTTGGTGATGTGTTCATTAAACCGTTCCCATCCGAGTTTTCCAGAA
TRINITY_DN2570_c0_g3_i2          AACGAAGCGTCGACCTTGGTGATGTGTTCATTAAACCGTTCCCATCCGAGTTTTCCAGAA
TRINITY_DN2570_c0_g3_i1          AACGAAGCGTCGACCTTGGTGATGTGTTCATTAAACCGTTCCCATCCGAGTTTTCCAGAA
TRINITY_DN32_c0_g1_i1            AACGAAGCGTCGACCTTGGTGATGTGTTCATTAAACCGTTCCCATCCGAGTTTTCCAGAA
TRINITY_DN32_c0_g1_i2            AACGAAGCGTCGACCTTGGTGATGTGTTCATTAAACCGTTCCCATCCGAGTTTTCCAGAA
TRINITY_DN32_c0_g1_i4            AACGAAGCGTCGACCTTGGTGATGTGTTCATTAAACCGTTCCCATCCGAGTTTTCCAGAA
TRINITY_DN32_c0_g1_i3            AACGAAGCGTCGACCTTGGTGATGTGTTCATTAAACCGTTCCCATCCGAGTTTTCCAGAA
                                 ************************************************************

MpDNV_DNA_RNA_seq_consensus      TTATGATTTCTATTTACAAAATTACAAAAATAACTGTTATACATCAACGGTAAGTTCATG
TRINITY_DN2404_c0_g1_i4          TTATGATTTCTATTTACAAAATTACAAAAATAACTGTTATACATCAACGGTAAGTTCATG
TRINITY_DN2404_c0_g1_i2          TTATGATTTCTATTTACAAAATTACAAAAATAACTGTTATACATCAACGGTAAGTTCATG
TRINITY_DN2404_c0_g1_i1          TTATGATTTCTATTTACAAAATTACAAAAATAACTGTTATACATCAACGGTAAGTTCATG
TRINITY_DN2404_c0_g1_i3          TTATGATTTCTATTTACAAAATTACAAAAATAACTGTTATACATCAACGGTAAGTTCATG
TRINITY_DN2570_c0_g3_i4          TTATGATTTCTATTTACAAAATTACAAAAATAACTGTTATACATCAACGGTAAGTTCATG
TRINITY_DN2570_c0_g3_i3          TTATGATTTCTATTTACAAAATTACAAAAATAACTGTTATACATCAACGGTAAGTTCATG
TRINITY_DN2570_c0_g3_i2          TTATGATTTCTATTTACAAAATTACAAAAATAACTGTTATACATCAACGGTAAGTTCATG
TRINITY_DN2570_c0_g3_i1          TTATGATTTCTATTTACAAAATTACAAAAATAACTGTTATACATCAACGGTAAGTTCATG
TRINITY_DN32_c0_g1_i1            TTATGATTTCTATTTACAAAATTACAAAAATAACTGTTATACATCAACGGTAAGTTCATG
TRINITY_DN32_c0_g1_i2            TTATGATTTCTATTTACAAAATTACAAAAATAACTGTTATACATCAACGGTAAGTTCATG
TRINITY_DN32_c0_g1_i4            TTATGATTTCTATTTACAAAATTACAAAAATAACTGTTATACATCAACGGTAAGTTCATG
TRINITY_DN32_c0_g1_i3            TTATGATTTCTATTTACAAAATTACAAAAATAACTGTTATACATCAACGGTAAGTTCATG
                                 ************************************************************

MpDNV_DNA_RNA_seq_consensus      TAGGACGCAGGCAGTTCATATCCCTCTTTAGCAGAGAATGTACCATCTAGATCTTGACCG
TRINITY_DN2404_c0_g1_i4          TAGGACGCAGGCAGTTCATATCCCTCTTTAGCAGAGAATGTACCATCTAGATCTTGACCG
TRINITY_DN2404_c0_g1_i2          TAGGACGCAGGCAGTTCATATCCCTCTTTAGCAGAGAATGTACCATCTAGATCTTGACCG
TRINITY_DN2404_c0_g1_i1          TAGGACGCAGGCAGTTCATATCCCTCTTTAGCAGAGAATGTACCATCTAGATCTTGACCG
TRINITY_DN2404_c0_g1_i3          TAGGACGCAGGCAGTTCATATCCCTCTTTAGCAGAGAATGTACCATCTAGATCTTGACCG
TRINITY_DN2570_c0_g3_i4          TAGGACGCAGGCAGTTCATATCCCTCTTTAGCAGAGAATGTACCATCTAGATCTTGACCG
TRINITY_DN2570_c0_g3_i3          TAGGACGCAGGCAGTTCATATCCCTCTTTAGCAGAGAATGTACCATCTAGATCTTGACCG
TRINITY_DN2570_c0_g3_i2          TAGGACGCAGGCAGTTCATATCCCTCTTTAGCAGAGAATGTACCATCTAGATCTTGACCG
TRINITY_DN2570_c0_g3_i1          TAGGACGCAGGCAGTTCATATCCCTCTTTAGCAGAGAATGTACCATCTAGATCTTGACCG
TRINITY_DN32_c0_g1_i1            TAGGACGCAGGCAGTTCATATCCCTCTTTAGCAGAGAATGTACCATCTAGATCTTGACCG
TRINITY_DN32_c0_g1_i2            TAGGACGCAGGCAGTTCATATCCCTCTTTAGCAGAGAATGTACCATCTAGATCTTGACCG
TRINITY_DN32_c0_g1_i4            TAGGACGCAGGCAGTTCATATCCCTCTTTAGCAGAGAATGTACCATCTAGATCTTGACCG
TRINITY_DN32_c0_g1_i3            TAGGACGCAGGCAGTTCATATCCCTCTTTAGCAGAGAATGTACCATCTAGATCTTGACCG
                                 ************************************************************
```

```
MpDNV_DNA_RNA_seq_consensus      TACAATTTGCGAACGAATTCTTTCATAGACGACTGATCGTTCTCGGTATTCGTTGGATCC
TRINITY_DN2404_c0_g1_i4          TACAATTTGCGAACGAATTCTTTCATAGACGACTGATCGTTCTCGGTATTCGTTGGATCC
TRINITY_DN2404_c0_g1_i2          TACAATTTGCGAACGAATTCTTTCATAGACGACTGATCGTTCTCGGTATTCGTTGGATCC
TRINITY_DN2404_c0_g1_i1          TACAATTTGCGAACGAATTCTTTCATAGACGACTGATCGTTCTCGGTATTCGTTGGATCC
TRINITY_DN2404_c0_g1_i3          TACAATTTGCGAACGAATTCTTTCATAGACGACTGATCGTTCTCGGTATTCGTTGGATCC
TRINITY_DN2570_c0_g3_i4          TACAATTTGCGAACGAATTCTTTCATAGACGACTGATCGTTCTCGGTATTCGTTGGATCC
TRINITY_DN2570_c0_g3_i3          TACAATTTGCGAACGAATTCTTTCATAGACGACTGATCGTTCTCGGTATTCGTTGGATCC
TRINITY_DN2570_c0_g3_i2          TACAATTTGCGAACGAATTCTTTCATAGACGACTGATCGTTCTCGGTATTCGTTGGATCC
TRINITY_DN2570_c0_g3_i1          TACAATTTGCGAACGAATTCTTTCATAGACGACTGATCGTTCTCGGTATTCGTTGGATCC
TRINITY_DN32_c0_g1_i1            TACAATTTGCGAACGAATTCTTTCATAGACGACTGATCGTTCTCGGTATTCGTTGGATCC
TRINITY_DN32_c0_g1_i2            TACAATTTGCGAACGAATTCTTTCATAGACGACTGATCGTTCTCGGTATTCGTTGGATCC
TRINITY_DN32_c0_g1_i4            TACAATTTGCGAACGAATTCTTTCATAGACGACTGATCGTTCTCGGTATTCGTTGGATCC
TRINITY_DN32_c0_g1_i3            TACAATTTGCGAACGAATTCTTTCATAGACGACTGATCGTTCTCGGTATTCGTTGGATCC
                                 ************************************************************

MpDNV_DNA_RNA_seq_consensus      ATGGGCTCCGATGAATTGCCAAATGTGAGTTTTCGGTTAAATCCCCTTGTTTTTAAATTC
TRINITY_DN2404_c0_g1_i4          ATGGGCTCCGATGAATTGCCAAATGTGAGTTTTCGGTTAAATCCCCTTGTTTTTAAATTC
TRINITY_DN2404_c0_g1_i2          ATGGGCTCCGATGAATTGCCAAATGTGAGTTTTCGGTTAAATCCCCTTGTTTTTAAATTC
TRINITY_DN2404_c0_g1_i1          ATGGGCTCCGATGAATTGCCAAATGTGAGTTTTCGGTTAAATCCCCTTGTTTTTAAATTC
TRINITY_DN2404_c0_g1_i3          ATGGGCTCCGATGAATTGCCAAATGTGAGTTTTCGGTTAAATCCCCTTGTTTTTAAATTC
TRINITY_DN2570_c0_g3_i4          ATGGGCTCCGATGAATTGCCAAATGTGAGTTTTCGGTTAAATCCCCTTGTTTTTAAATTC
TRINITY_DN2570_c0_g3_i3          ATGGGCTCCGATGAATTGCCAAATGTGAGTTTTCGGTTAAATCCCCTTGTTTTTAAATTC
TRINITY_DN2570_c0_g3_i2          ATGGGCTCCGATGAATTGCCAAATGTGAGTTTTCGGTTAAATCCCCTTGTTTTTAAATTC
TRINITY_DN2570_c0_g3_i1          ATGGGCTCCGATGAATTGCCAAATGTGAGTTTTCGGTTAAATCCCCTTGTTTTTAAATTC
TRINITY_DN32_c0_g1_i1            ATGGGCTCCGATGAATTGCCAAATGTGAGTTTTCGGTTAAATCCCCTTGTTTTTAAATTC
TRINITY_DN32_c0_g1_i2            ATGGGCTCCGATGAATTGCCAAATGTGAGTTTTCGGTTAAATCCCCTTGTTTTTAAATTC
TRINITY_DN32_c0_g1_i4            ATGGGCTCCGATGAATTGCCAAATGTGAGTTTTCGGTTAAATCCCCTTGTTTTTAAATTC
TRINITY_DN32_c0_g1_i3            ATGGGCTCCGATGAATTGCCAAATGTGAGTTTTCGGTTAAATCCCCTTGTTTTTAAATTC
                                 ************************************************************

MpDNV_DNA_RNA_seq_consensus      AAAGCGTCCGCTTTTACCATAAATTTGTTCTGATTCAATGTGGCCAGGGAGCTCGTAGAG
TRINITY_DN2404_c0_g1_i4          AAAGCGTCCGCTTTTACCATAAATTTGTTCTGATTCAATGTGGCCAGGGAGCTCGTAGAG
TRINITY_DN2404_c0_g1_i2          AAAGCGTCCGCTTTTACCATAAATTTGTTCTGATTCAATGTGGCCAGGGAGCTCGTAGAG
TRINITY_DN2404_c0_g1_i1          AAAGCGTCCGCTTTTACCATAAATTTGTTCTGATTCAATGTGGCCAGGGAGCTCGTAGAG
TRINITY_DN2404_c0_g1_i3          AAAGCGTCCGCTTTTACCATAAATTTGTTCTGATTCAATGTGGCCAGGGAGCTCGTAGAG
TRINITY_DN2570_c0_g3_i4          AAAGCGTCCGCTTTTACCATAAATTTGTTCTGATTCAATGTGGCCAGGGAGCTCGTAGAG
TRINITY_DN2570_c0_g3_i3          AAAGCGTCCGCTTTTACCATAAATTTGTTCTGATTCAATGTGGCCAGGGAGCTCGTAGAG
TRINITY_DN2570_c0_g3_i2          AAAGCGTCCGCTTTTACCATAAATTTGTTCTGATTCAATGTGGCCAGGGAGCTCGTAGAG
TRINITY_DN2570_c0_g3_i1          AAAGCGTCCGCTTTTACCATAAATTTGTTCTGATTCAATGTGGCCAGGGAGCTCGTAGAG
TRINITY_DN32_c0_g1_i1            AAAGCGTCCGCTTTTACCATAAATTTGTTCTGATTCAATGTGGCCAGGGAGCTCGTAGAG
TRINITY_DN32_c0_g1_i2            AAAGCGTCCGCTTTTACCATAAATTTGTTCTGATTCAATGTGGCCAGGGAGCTCGTAGAG
TRINITY_DN32_c0_g1_i4            AAAGCGTCCGCTTTTACCATAAATTTGTTCTGATTCAATGTGGCCAGGGAGCTCGTAGAG
TRINITY_DN32_c0_g1_i3            AAAGCGTCCGCTTTTACCATAAATTTGTTCTGATTCAATGTGGCCAGGGAGCTCGTAGAG
                                 ************************************************************

MpDNV_DNA_RNA_seq_consensus      CTATTGGTTTCAAAAGCTGTGCGAGGATTACGCATGACACCCTTGCATTTGACTTCAGTG
TRINITY_DN2404_c0_g1_i4          CTATTGGTTTCAAAAGCTGTGCGAGGATTACGCATGACACCCTTGCATTTGACTTCAGTG
TRINITY_DN2404_c0_g1_i2          CTATTGGTTTCAAAAGCTGTGCGAGGATTACGCATGACACCCTTGCATTTGACTTCAGTG
TRINITY_DN2404_c0_g1_i1          CTATTGGTTTCAAAAGCTGTGCGAGGATTACGCATGACACCCTTGCATTTGACTTCAGTG
TRINITY_DN2404_c0_g1_i3          CTATTGGTTTCAAAAGCTGTGCGAGGATTACGCATGACACCCTTGCATTTGACTTCAGTG
TRINITY_DN2570_c0_g3_i4          CTATTGGTTTCAAAAGCTGTGCGAGGATTACGCATGACACCCTTGCATTTGACTTCAGTG
TRINITY_DN2570_c0_g3_i3          CTATTGGTTTCAAAAGCTGTGCGAGGATTACGCATGACACCCTTGCATTTGACTTCAGTG
TRINITY_DN2570_c0_g3_i2          CTATTGGTTTCAAAAGCTGTGCGAGGATTACGCATGACACCCTTGCATTTGACTTCAGTG
TRINITY_DN2570_c0_g3_i1          CTATTGGTTTCAAAAGCTGTGCGAGGATTACGCATGACACCCTTGCATTTGACTTCAGTG
TRINITY_DN32_c0_g1_i1            CTATTGGTTTCAAAAGCTGTGCGAGGATTACGCATGACACCCTTGCATTTGACTTCAGTG
TRINITY_DN32_c0_g1_i2            CTATTGGTTTCAAAAGCTGTGCGAGGATTACGCATGACACCCTTGCATTTGACTTCAGTG
TRINITY_DN32_c0_g1_i4            CTATTGGTTTCAAAAGCTGTGCGAGGATTACGCATGACACCCTTGCATTTGACTTCAGTG
TRINITY_DN32_c0_g1_i3            CTATTGGTTTCAAAAGCTGTGCGAGGATTACGCATGACACCCTTGCATTTGACTTCAGTG
                                 ************************************************************

MpDNV_DNA_RNA_seq_consensus      CATATAGCACCTCTGGGTAAAAATTTAAACTCGGACGGCGACATATAGAACCAAGGTTGG
TRINITY_DN2404_c0_g1_i4          CATATAGCACCTCTGGGTAAAAATTTAAACTCGGACGGCGACATATAGAACCAAGGTTGG
TRINITY_DN2404_c0_g1_i2          CATATAGCACCTCTGGGTAAAAATTTAAACTCGGACGGCGACATATAGAACCAAGGTTGG
TRINITY_DN2404_c0_g1_i1          CATATAGCACCTCTGGGTAAAAATTTAAACTCGGACGGCGACATATAGAACCAAGGTTGG
TRINITY_DN2404_c0_g1_i3          CATATAGCACCTCTGGGTAAAAATTTAAACTCGGACGGCGACATATAGAACCAAGGTTGG
TRINITY_DN2570_c0_g3_i4          CATATAGCACCTCTGGGTAAAAATTTAAACTCGGACGGCGACATATAGAACCAAGGTTGG
TRINITY_DN2570_c0_g3_i3          CATATAGCACCTCTGGGTAAAAATTTAAACTCGGACGGCGACATATAGAACCAAGGTTGG
TRINITY_DN2570_c0_g3_i2          CATATAGCACCTCTGGGTAAAAATTTAAACTCGGACGGCGACATATAGAACCAAGGTTGG
TRINITY_DN2570_c0_g3_i1          CATATAGCACCTCTGGGTAAAAATTTAAACTCGGACGGCGACATATAGAACCAAGGTTGG
TRINITY_DN32_c0_g1_i1            CATATAGCACCTCTGGGTAAAAATTTAAACTCGGACGGCGACATATAGAACCAAGGTTGG
TRINITY_DN32_c0_g1_i2            CATATAGCACCTCTGGGTAAAAATTTAAACTCGGACGGCGACATATAGAACCAAGGTTGG
TRINITY_DN32_c0_g1_i4            CATATAGCACCTCTGGGTAAAAATTTAAACTCGGACGGCGACATATAGAACCAAGGTTGG
TRINITY_DN32_c0_g1_i3            CATATAGCACCTCTGGGTAAAAATTTAAACTCGGACGGCGACATATAGAACCAAGGTTGG
                                 ************************************************************

MpDNV_DNA_RNA_seq_consensus      TCCACGGGCACACTCATTAAACTCGTTGTCCCAAGATAGGCGTTATCAGTGCTAAGATAT
TRINITY_DN2404_c0_g1_i4          TCCACGGGCACACTCATTAAACTCGTTGTCCCAAGATAGGCGTTATCAGTGCTAAGATAT
TRINITY_DN2404_c0_g1_i2          TCCACGGGCACACTCATTAAACTCGTTGTCCCAAGATAGGCGTTATCAGTGCTAAGATAT
TRINITY_DN2404_c0_g1_i1          TCCACGGGCACACTCATTAAACTCGTTGTCCCAAGATAGGCGTTATCAGTGCTAAGATAT
TRINITY_DN2404_c0_g1_i3          TCCACGGGCACACTCATTAAACTCGTTGTCCCAAGATAGGCGTTATCAGTGCTAAGATAT
TRINITY_DN2570_c0_g3_i4          TCCACGGGCACACTCATTAAACTCGTTGTCCCAAGATAGGCGTTATCAGTGCTAAGATAT
TRINITY_DN2570_c0_g3_i3          TCCACGGGCACACTCATTAAACTCGTTGTCCCAAGATAGGCGTTATCAGTGCTAAGATAT
TRINITY_DN2570_c0_g3_i2          TCCACGGGCACACTCATTAAACTCGTTGTCCCAAGATAGGCGTTATCAGTGCTAAGATAT
TRINITY_DN2570_c0_g3_i1          TCCACGGGCACACTCATTAAACTCGTTGTCCCAAGATAGGCGTTATCAGTGCTAAGATAT
TRINITY_DN32_c0_g1_i1            TCCACGGGCACACTCATTAAACTCGTTGTCCCAAGATAGGCGTTATCAGTGCTAAGATAT
TRINITY_DN32_c0_g1_i2            TCCACGGGCACACTCATTAAACTCGTTGTCCCAAGATAGGCGTTATCAGTGCTAAGATAT
TRINITY_DN32_c0_g1_i4            TCCACGGGCACACTCATTAAACTCGTTGTCCCAAGATAGGCGTTATCAGTGCTAAGATAT
TRINITY_DN32_c0_g1_i3            TCCACGGGCACACTCATTAAACTCGTTGTCCCAAGATAGGCGTTATCAGTGCTAAGATAT
```

```
                                    ************************************************************

MpDNV_DNA_RNA_seq_consensus         CGCAACACTTTCCAACCACATCCGTAACTGATGAAAGTATGTACTTTTCGGTAAACCATA
TRINITY_DN2404_c0_g1_i4             CGCAACACTTTCCAACCACATCCGTAACTGATGAAAGTATGTACTTTTCGGTAAACCATA
TRINITY_DN2404_c0_g1_i2             CGCAACACTTTCCAACCACATCCGTAACTGATGAAAGTATGTACTTTTCGGTAAACCATA
TRINITY_DN2404_c0_g1_i1             CGCAACACTTTCCAACCACATCCGTAACTGATGAAAGTATGTACTTTTCGGTAAACCATA
TRINITY_DN2404_c0_g1_i3             CGCAACACTTTCCAACCACATCCGTAACTGATGAAAGTATGTACTTTTCGGTAAACCATA
TRINITY_DN2570_c0_g3_i4             CGCAACACTTTCCAACCACATCCGTAACTGATGAAAGTATGTACTTTTCGGTAAACCATA
TRINITY_DN2570_c0_g3_i3             CGCAACACTTTCCAACCACATCCGTAACTGATGAAAGTATGTACTTTTCGGTAAACCATA
TRINITY_DN2570_c0_g3_i2             CGCAACACTTTCCAACCACATCCGTAACTGATGAAAGTATGTACTTTTCGGTAAACCATA
TRINITY_DN2570_c0_g3_i1             CGCAACACTTTCCAACCACATCCGTAACTGATGAAAGTATGTACTTTTCGGTAAACCATA
TRINITY_DN32_c0_g1_i1               CGCAACACTTTCCAACCACATCCGTAACTGATGAAAGTATGTACTTTTCGGTAAACCATA
TRINITY_DN32_c0_g1_i2               CGCAACACTTTCCAACCACATCCGTAACTGATGAAAGTATGTACTTTTCGGTAAACCATA
TRINITY_DN32_c0_g1_i4               CGCAACACTTTCCAACCACATCCGTAACTGATGAAAGTATGTACTTTTCGGTAAACCATA
TRINITY_DN32_c0_g1_i3               CGCAACACTTTCCAACCACATCCGTAACTGATGAAAGTATGTACTTTTCGGTAAACCATA
                                    ************************************************************

MpDNV_DNA_RNA_seq_consensus         GTATATCCTCCCGTGTGACCGATTGGCCTAGGTATAACAGCGTTTTCAGAGCTAGGATTG
TRINITY_DN2404_c0_g1_i4             GTATATCCTCCCGTGTGACCGATTGGCCTAGGTATAACAGCGTTTTCAGAGCTAGGATTG
TRINITY_DN2404_c0_g1_i2             GTATATCCTCCCGTGTGACCGATTGGCCTAGGTATAACAGCGTTTTCAGAGCTAGGATTG
TRINITY_DN2404_c0_g1_i1             GTATATCCTCCCGTGTGACCGATTGGCCTAGGTATAACAGCGTTTTCAGAGCTAGGATTG
TRINITY_DN2404_c0_g1_i3             GTATATCCTCCCGTGTGACCGATTGGCCTAGGTATAACAGCGTTTTCAGAGCTAGGATTG
TRINITY_DN2570_c0_g3_i4             GTATATCCTCCCGTGTGACCGATTGGCCTAGGTATAACAGCGTTTTCAGAGCTAGGATTG
TRINITY_DN2570_c0_g3_i3             GTATATCCTCCCGTGTGACCGATTGGCCTAGGTATAACAGCGTTTTCAGAGCTAGGATTG
TRINITY_DN2570_c0_g3_i2             GTATATCCTCCCGTGTGACCGATTGGCCTAGGTATAACAGCGTTTTCAGAGCTAGGATTG
TRINITY_DN2570_c0_g3_i1             GTATATCCTCCCGTGTGACCGATTGGCCTAGGTATAACAGCGTTTTCAGAGCTAGGATTG
TRINITY_DN32_c0_g1_i1               GTATATCCTCCCGTGTGACCGATTGGCCTAGGTATAACAGCGTTTTCAGAGCTAGGATTG
TRINITY_DN32_c0_g1_i2               GTATATCCTCCCGTGTGACCGATTGGCCTAGGTATAACAGCGTTTTCAGAGCTAGGATTG
TRINITY_DN32_c0_g1_i4               GTATATCCTCCCGTGTGACCGATTGGCCTAGGTATAACAGCGTTTTCAGAGCTAGGATTG
TRINITY_DN32_c0_g1_i3               GTATATCCTCCCGTGTGACCGATTGGCCTAGGTATAACAGCGTTTTCAGAGCTAGGATTG
                                    ************************************************************

MpDNV_DNA_RNA_seq_consensus         CCAGTATCTGGGTCCCCATCGTTCGCAGCAGCCGTCCCAGGAAGCGAGGTGCCACCCGCA
TRINITY_DN2404_c0_g1_i4             CCAGTATCTGGGTCCCCATCGTTCGCAGCAGCCGTCCCAGGAAGCGAGGTGCCACCCGCA
TRINITY_DN2404_c0_g1_i2             CCAGTATCTGGGTCCCCATCGTTCGCAGCAGCCGTCCCAGGAAGCGAGGTGCCACCCGCA
TRINITY_DN2404_c0_g1_i1             CCAGTATCTGGGTCCCCATCGTTCGCAGCAGCCGTCCCAGGAAGCGAGGTGCCACCCGCA
TRINITY_DN2404_c0_g1_i3             CCAGTATCTGGGTCCCCATCGTTCGCAGCAGCCGTCCCAGGAAGCGAGGTGCCACCCGCA
TRINITY_DN2570_c0_g3_i4             CCAGTATCTGGGTCCCCATCGTTCGCAGCAGCCGTCCCAGGAAGCGAGGTGCCACCCGCA
TRINITY_DN2570_c0_g3_i3             CCAGTATCTGGGTCCCCATCGTTCGCAGCAGCCGTCCCAGGAAGCGAGGTGCCACCCGCA
TRINITY_DN2570_c0_g3_i2             CCAGTATCTGGGTCCCCATCGTTCGCAGCAGCCGTCCCAGGAAGCGAGGTGCCACCCGCA
TRINITY_DN2570_c0_g3_i1             CCAGTATCTGGGTCCCCATCGTTCGCAGCAGCCGTCCCAGGAAGCGAGGTGCCACCCGCA
TRINITY_DN32_c0_g1_i1               CCAGTATCTGGGTCCCCATCGTTCGCAGCAGCCGTCCCAGGAAGCGAGGTGCCACCCGCA
TRINITY_DN32_c0_g1_i2               CCAGTATCTGGGTCCCCATCGTTCGCAGCAGCCGTCCCAGGAAGCGAGGTGCCACCCGCA
TRINITY_DN32_c0_g1_i4               CCAGTATCTGGGTCCCCATCGTTCGCAGCAGCCGTCCCAGGAAGCGAGGTGCCACCCGCA
TRINITY_DN32_c0_g1_i3               CCAGTATCTGGGTCCCCATCGTTCGCAGCAGCCGTCCCAGGAAGCGAGGTGCCACCCGCA
                                    ************************************************************

MpDNV_DNA_RNA_seq_consensus         CGTTTAGGAGCGGGTTCAGAACCGGAGCTATCGGCAGGTCGTTTGCTACTTGATCCGCTA
TRINITY_DN2404_c0_g1_i4             CGTTTAGGAGCGGGTTCAGAACCGGAGCTATCGGCAGGTCGTTTGCTACTTGATCCGCTA
TRINITY_DN2404_c0_g1_i2             CGTTTAGGAGCGGGTTCAGAACCGGAGCTATCGGCAGGTCGTTTGCTACTTGATCCGCTA
TRINITY_DN2404_c0_g1_i1             CGTTTAGGAGCGGGTTCAGAACCGGAGCTATCGGCAGGTCGTTTGCTACTTGATCCGCTA
TRINITY_DN2404_c0_g1_i3             CGTTTAGGAGCGGGTTCAGAACCGGAGCTATCGGCAGGTCGTTTGCTACTTGATCCGCTA
TRINITY_DN2570_c0_g3_i4             CGTTTAGGAGCGGGTTCAGAACCGGAGCTATCGGCAGGTCGTTTGCTACTTGATCCGCTA
TRINITY_DN2570_c0_g3_i3             CGTTTAGGAGCGGGTTCAGAACCGGAGCTATCGGCAGGTCGTTTGCTACTTGATCCGCTA
TRINITY_DN2570_c0_g3_i2             CGTTTAGGAGCGGGTTCAGAACCGGAGCTATCGGCAGGTCGTTTGCTACTTGATCCGCTA
TRINITY_DN2570_c0_g3_i1             CGTTTAGGAGCGGGTTCAGAACCGGAGCTATCGGCAGGTCGTTTGCTACTTGATCCGCTA
TRINITY_DN32_c0_g1_i1               CGTTTAGGAGCGGGTTCAGAACCGGAGCTATCGGCAGGTCGTTTGCTACTTGATCCGCTA
TRINITY_DN32_c0_g1_i2               CGTTTAGGAGCGGGTTCAGAACCGGAGCTATCGGCAGGTCGTTTGCTACTTGATCCGCTA
TRINITY_DN32_c0_g1_i4               CGTTTAGGAGCGGGTTCAGAACCGGAGCTATCGGCAGGTCGTTTGCTACTTGATCCGCTA
TRINITY_DN32_c0_g1_i3               CGTTTAGGAGCGGGTTCAGAACCGGAGCTATCGGCAGGTCGTTTGCTACTTGATCCGCTA
                                    ************************************************************

MpDNV_DNA_RNA_seq_consensus         GAGCTTGCCATGGATCCCGATGCGCTTCCCCCGTTGCCGTTGCTAGTGCTGTTGTCCGGG
TRINITY_DN2404_c0_g1_i4  APFV-11   GAGCTTGCCATGGATCCCGATACGCTTCCCCCGTTGCCGTTGCTAGTGCTGTTGTCCGGG
TRINITY_DN2404_c0_g1_i2             GAGCTTGCCATGGATCCCGATACGCTTCCCCCGTTGCCGTTGCTAGTGCTGTTGTCCGGG
TRINITY_DN2404_c0_g1_i1             GAGCTTGCCATGGATCCCGATACGCTTCCCCCGTTGCCGTTGCTAGTGCTGTTGTCCGGG
TRINITY_DN2404_c0_g1_i3             GAGCTTGCCATGGATCCCGATACGCTTCCCCCGTTGCCGTTGCTAGTGCTGTTGTCCGGG
TRINITY_DN2570_c0_g3_i4             GAGCTTGCCATGGATCCCGATGCGCTTCCCCCGTTGCCGTTGCTAGTGCTGTTGTCCGGG
TRINITY_DN2570_c0_g3_i3             GAGCTTGCCATGGATCCCGATGCGCTTCCCCCGTTGCCGTTGCTAGTGCTGTTGTCCGGG
TRINITY_DN2570_c0_g3_i2             GAGCTTGCCATGGATCCCGATGCGCTTCCCCCGTTGCCGTTGCTAGTGCTGTTGTCCGGG
TRINITY_DN2570_c0_g3_i1             GAGCTTGCCATGGATCCCGATGCGCTTCCCCCGTTGCCGTTGCTAGTGCTGTTGTCCGGG
TRINITY_DN32_c0_g1_i1               GAGCTTGCCATGGATCCCGATGCGCTTCCCCCGTTGCCGTTGCTAGTGCTGTTGTCCGGG
TRINITY_DN32_c0_g1_i2               GAGCTTGCCATGGATCCCGATGCGCTTCCCCCGTTGCCGTTGCTAGTGCTGTTGTCCGGG
TRINITY_DN32_c0_g1_i4               GAGCTTGCCATGGATCCCGATGCGCTTCCCCCGTTGCCGTTGCTAGTGCTGTTGTCCGGG
TRINITY_DN32_c0_g1_i3               GAGCTTGCCATGGATCCCGATGCGCTTCCCCCGTTGCCGTTGCTAGTGCTGTTGTCCGGG
                                    ********************* **************************************

MpDNV_DNA_RNA_seq_consensus         GCTGAGGTGTTTGCCACGTGGTCGCCACTCGCTTCACCGCTGTTAGGATTGCCGTTACCG
TRINITY_DN2404_c0_g1_i4             GCTGAGGTGTTTGCCACGTGGTCGCCACTCGCTTCACCGCTGTTAGGATTGCCGTTACCG
TRINITY_DN2404_c0_g1_i2             GCTGAGGTGTTTGCCACGTGGTCGCCACTCGCTTCACCGCTGTTAGGATTGCCGTTACCG
TRINITY_DN2404_c0_g1_i1             GCTGAGGTGTTTGCCACGTGGTCGCCACTCGCTTCACCGCTGTTAGGATTGCCGTTACCG
TRINITY_DN2404_c0_g1_i3             GCTGAGGTGTTTGCCACGTGGTCGCCACTCGCTTCACCGCTGTTAGGATTGCCGTTACCG
TRINITY_DN2570_c0_g3_i4             GCTGAGGTGTTTGCCACGTGGTCGCCACTCGCTTCACCGCTGTTAGGATTGCCGTTACCG
TRINITY_DN2570_c0_g3_i3             GCTGAGGTGTTTGCCACGTGGTCGCCACTCGCTTCACCGCTGTTAGGATTGCCGTTACCG
TRINITY_DN2570_c0_g3_i2             GCTGAGGTGTTTGCCACGTGGTCGCCACTCGCTTCACCGCTGTTAGGATTGCCGTTACCG
TRINITY_DN2570_c0_g3_i1             GCTGAGGTGTTTGCCACGTGGTCGCCACTCGCTTCACCGCTGTTAGGATTGCCGTTACCG
TRINITY_DN32_c0_g1_i1               GCTGAGGTGTTTGCCACGTGGTCGCCACTCGCTTCACCGCTGTTAGGATTGCCGTTACCG
TRINITY_DN32_c0_g1_i2               GCTGAGGTGTTTGCCACGTGGTCGCCACTCGCTTCACCGCTGTTAGGATTGCCGTTACCG
```

```
TRINITY_DN32_c0_g1_i4           GCTGAGGTGTTTGCCACGTGGTCGCCACTCGCTTCACCGCTGTTAGGATTGCCGTTACCG
TRINITY_DN32_c0_g1_i3           GCTGAGGTGTTTGCCACGTGGTCGCCACTCGCTTCACCGCTGTTAGGATTGCCGTTACCG
                                ************************************************************

MpDNV_DNA_RNA_seq_consensus     TCGTAGTCCCAATCCAAATCAGGATCCTCCTCCACGCCACCTAAAACATACGGTGGTAAT 3'splice site
TRINITY_DN2404_c0_g1_i4         TCGTAGTCCCAATCCAAATCAGGATCCTCCTCCACGCCACCTAAAACATACGGTGGTAAT VP unspliced
TRINITY_DN2404_c0_g1_i2         TCGTAGTCCCAATCCAAATCAGGATCCTCCTCCACGCCAC-------------------- VP spliced 1
TRINITY_DN2404_c0_g1_i1         TCGTAGTCCCAATCCAAATCAGGATCCTCCTCCACGCCAC-------------------- VP spliced 1
TRINITY_DN2404_c0_g1_i3         TCGTAGTCCCAATCCAAATCAGGATCCTCCTCCACGCCACCTAAAACATACGGTGGTAAT VP unspliced
TRINITY_DN2570_c0_g3_i4         TCGTAGTCCCAATCCAAATCAGGATCCTCCTCCACGCCACCTAAAACATACGGTGGTAAT VP unspliced
TRINITY_DN2570_c0_g3_i3         TCGTAGTCCCAATCCAAATCAGGATCCTCCTCCACGCCAC-------------------- VP spliced 1
TRINITY_DN2570_c0_g3_i2         TCGTAGTCCCAATCCAAATCAGGATCCTCCTCCACGCCACCTAAAACATACGGTGGTAAT VP unspliced
TRINITY_DN2570_c0_g3_i1         TCGTAGTCCCAATCCAAATCAGGATCCTCCTCCACGCCAC-------------------- VP spliced 1
TRINITY_DN32_c0_g1_i1           TCGTAGTCCCAATCCAAATCAGGATCCTCCTCCACGCCAC-------------------- VP spliced 1
TRINITY_DN32_c0_g1_i2           TCGTAGTCCCAATCCAAATCAGGATCCTCCTCCACGCCACCTAAAACATACGGTGGTAAT VP unspliced
TRINITY_DN32_c0_g1_i4           TCGTAGTCCCAATCCAAATCAGGATCCTCCTCCACGCCACCTAAAACATACGGTGGTAAT VP unspliced
TRINITY_DN32_c0_g1_i3           TCGTAGTCCCAATCCAAATCAGGATCCTCCTCCACGCCAC-------------------- VP spliced 1
                                ****************************************

MpDNV_DNA_RNA_seq_consensus     GAACGACGTGCTAAAGCCTTATTGTATTGTTCTAGCGCGTAACGCTGCTGCGATGAATTT
TRINITY_DN2404_c0_g1_i4         GAACGACGTGCTAAAGCCTTATTGTATTGTTCTAGCGCGTAACGCTGCTGCGATGAATTT
TRINITY_DN2404_c0_g1_i2         ------------------------------------------------------------
TRINITY_DN2404_c0_g1_i1         ------------------------------------------------------------
TRINITY_DN2404_c0_g1_i3         GAACGACGTGCTAAAGCCTTATTGTATTGTTCTAGCGCGTAACGCTGCTGCGATGAATTT
TRINITY_DN2570_c0_g3_i4         GAACGACGTGCTAAAGCCTTATTGTATTGTTCTAGCGCGTAACGCTGCTGCGATGAATTT
TRINITY_DN2570_c0_g3_i3         ------------------------------------------------------------
TRINITY_DN2570_c0_g3_i2         GAACGACGTGCTAAAGCCTTATTGTATTGTTCTAGCGCGTAACGCTGCTGCGATGAATTT
TRINITY_DN2570_c0_g3_i1         ------------------------------------------------------------
TRINITY_DN32_c0_g1_i1           ------------------------------------------------------------
TRINITY_DN32_c0_g1_i2           GAACGACGTGCTAAAGCCTTATTGTATTGTTCTAGCGCGTAACGCTGCTGCGATGAATTT
TRINITY_DN32_c0_g1_i4           GAACGACGTGCTAAAGCCTTATTGTATTGTTCTAGCGCGTAACGCTGCTGCGATGAATTT
TRINITY_DN32_c0_g1_i3           ------------------------------------------------------------

MpDNV_DNA_RNA_seq_consensus     AAATTTTGCCAATTGGCCCGCTCCGGTGGAGGAGGACCTAAATAACGAAATTCTCGCATA
TRINITY_DN2404_c0_g1_i4         AAATTTTGCCAATTGGCCCGCTCCGGTGGAGGAGGACCTAAATAACGAAATTCTCGCATA
TRINITY_DN2404_c0_g1_i2         ------------------------------------------------------------
TRINITY_DN2404_c0_g1_i1         ------------------------------------------------------------
TRINITY_DN2404_c0_g1_i3         AAATTTTGCCAATTGGCCCGCTCCGGTGGAGGAGGACCTAAATAACGAAATTCTCGCATA
TRINITY_DN2570_c0_g3_i4         AAATTTTGCCAATTGGCCCGCTCCGGTGGAGGAGGACCTAAATAACGAAATTCTCGCATA
TRINITY_DN2570_c0_g3_i3         ------------------------------------------------------------
TRINITY_DN2570_c0_g3_i2         AAATTTTGCCAATTGGCCCGCTCCGGTGGAGGAGGACCTAAATAACGAAATTCTCGCATA
TRINITY_DN2570_c0_g3_i1         ------------------------------------------------------------
TRINITY_DN32_c0_g1_i1           ------------------------------------------------------------
TRINITY_DN32_c0_g1_i2           AAATTTTGCCAATTGGCCCGCTCCGGTGGAGGAGGACCTAAATAACGAAATTCTCGCATA
TRINITY_DN32_c0_g1_i4           AAATTTTGCCAATTGGCCCGCTCCGGTGGAGGAGGACCTAAATAACGAAATTCTCGCATA
TRINITY_DN32_c0_g1_i3           ------------------------------------------------------------

MpDNV_DNA_RNA_seq_consensus     CTTACGGCTACTGTCTGCTGCCGCTAAATACGGTGTTGTATCAAAATATGTTCCGCTAGT 5'splice site
TRINITY_DN2404_c0_g1_i4         CTTACGGCTACTGTCTGCTGCCGCTAAATACGGTGTTGTATCAAAATATGTTCCGCTAGT VP unspliced
TRINITY_DN2404_c0_g1_i2         -----GGCTACTGTCTGCTGCCGCTAAATACGGTGTTGTATCAAAATATGTTCCGCTAGT VP spliced 1
TRINITY_DN2404_c0_g1_i1         -----GGCTACTGTCTGCTGCCGCTAAATACGGTGTTGTATCAAAATATGTTCCGCTAGT VP spliced 1
TRINITY_DN2404_c0_g1_i3         CTTACGGCTACTGTCTGCTGCCGCTAAATACGGTGTTGTATCAAAATATGTTCCGCTAGT VP unspliced
TRINITY_DN2570_c0_g3_i4         CTTACGGCTACTGTCTGCTGCCGCTAAATACGGTGTTGTATCAAAATATGTTCCGCTAGT VP unspliced
TRINITY_DN2570_c0_g3_i3         -----GGCTACTGTCTGCTGCCGCTAAATACGGTGTTGTATCAAAATATGTTCCGCTAGT VP spliced 1
TRINITY_DN2570_c0_g3_i2         CTTACGGCTACTGTCTGCTGCCGCTAAATACGGTGTTGTATCAAAATATGTTCCGCTAGT VP unspliced
TRINITY_DN2570_c0_g3_i1         -----GGCTACTGTCTGCTGCCGCTAAATACGGTGTTGTATCAAAATATGTTCCGCTAGT VP spliced 1
TRINITY_DN32_c0_g1_i1           -----GGCTACTGTCTGCTGCCGCTAAATACGGTGTTGTATCAAAATATGTTCCGCTAGT VP spliced 1
TRINITY_DN32_c0_g1_i2           CTTACGGCTACTGTCTGCTGCCGCTAAATACGGTGTTGTATCAAAATATGTTCCGCTAGT VP unspliced
TRINITY_DN32_c0_g1_i4           CTTACGGCTACTGTCTGCTGCCGCTAAATACGGTGTTGTATCAAAATATGTTCCGCTAGT VP unspliced
TRINITY_DN32_c0_g1_i3           -----GGCTACTGTCTGCTGCCGCTAAATACGGTGTTGTATCAAAATATGTTCCGCTAGT VP spliced 1
                                     *******************************************************

MpDNV_DNA_RNA_seq_consensus     GTTAGGTTTGTTCGGGTATATTGCTGTCTCGCTGCCTGTCAAGAACTGATCGGCTAAATC
TRINITY_DN2404_c0_g1_i4         GTTAGGTTTGTTCGGGTATATTGCTGTCTCGCTGCCTGTCAAGAACTGATCGGCTAAATC
TRINITY_DN2404_c0_g1_i2         GTTAGGTTTGTTCGGGTATATTGCTGTCTCGCTGCCTGTCAAGAACTGATCGGCTAAATC
TRINITY_DN2404_c0_g1_i1         GTTAGGTTTGTTCGGGTATATTGCTGTCTCGCTGCCTGTCAAGAACTGATCGGCTAAATC
TRINITY_DN2404_c0_g1_i3         GTTAGGTTTGTTCGGGTATATTGCTGTCTCGCTGCCTGTCAAGAACTGATCGGCTAAATC
TRINITY_DN2570_c0_g3_i4         GTTAGGTTTGTTCGGGTATATTGCTGTCTCGCTGCCTGTCAAGAACTGATCGGCTAAATC
TRINITY_DN2570_c0_g3_i3         GTTAGGTTTGTTCGGGTATATTGCTGTCTCGCTGCCTGTCAAGAACTGATCGGCTAAATC
TRINITY_DN2570_c0_g3_i2         GTTAGGTTTGTTCGGGTATATTGCTGTCTCGCTGCCTGTCAAGAACTGATCGGCTAAATC
TRINITY_DN2570_c0_g3_i1         GTTAGGTTTGTTCGGGTATATTGCTGTCTCGCTGCCTGTCAAGAACTGATCGGCTAAATC
TRINITY_DN32_c0_g1_i1           GTTAGGTTTGTTCGGGTATATTGCTGTCTCGCTGCCTGTCAAGAACTGATCGGCTAAATC
TRINITY_DN32_c0_g1_i2           GTTAGGTTTGTTCGGGTATATTGCTGTCTCGCTGCCTGTCAAGAACTGATCGGCTAAATC
TRINITY_DN32_c0_g1_i4           GTTAGGTTTGTTCGGGTATATTGCTGTCTCGCTGCCTGTCAAGAACTGATCGGCTAAATC
TRINITY_DN32_c0_g1_i3           GTTAGGTTTGTTCGGGTATATTGCTGTCTCGCTGCCTGTCAAGAACTGATCGGCTAAATC
                                ************************************************************

MpDNV_DNA_RNA_seq_consensus     GACACCACGCTTTGCGATCAATCCGAGAGCTGTCGCAATGTTGCCATAATATGGGTCGCT
TRINITY_DN2404_c0_g1_i4         GACACCACGCTTTGCGATCAATCCGAGAGCTGTCGCAATGTTGCCATAATATGGGTCGCT
TRINITY_DN2404_c0_g1_i2         GACACCACGCTTTGCGATCAATCCGAGAGCTGTCGCAATGTTGCCATAATATGGGTCGCT
TRINITY_DN2404_c0_g1_i1         GACACCACGCTTTGCGATCAATCCGAGAGCTGTCGCAATGTTGCCATAATATGGGTCGCT
TRINITY_DN2404_c0_g1_i3         GACACCACGCTTTGCGATCAATCCGAGAGCTGTCGCAATGTTGCCATAATATGGGTCGCT
TRINITY_DN2570_c0_g3_i4         GACACCACGCTTTGCGATCAATCCGAGAGCTGTCGCAATGTTGCCATAATATGGGTCGCT
TRINITY_DN2570_c0_g3_i3         GACACCACGCTTTGCGATCAATCCGAGAGCTGTCGCAATGTTGCCATAATATGGGTCGCT
TRINITY_DN2570_c0_g3_i2         GACACCACGCTTTGCGATCAATCCGAGAGCTGTCGCAATGTTGCCATAATATGGGTCGCT
TRINITY_DN2570_c0_g3_i1         GACACCACGCTTTGCGATCAATCCGAGAGCTGTCGCAATGTTGCCATAATATGGGTCGCT
TRINITY_DN32_c0_g1_i1           GACACCACGCTTTGCGATCAATCCGAGAGCTGTCGCAATGTTGCCATAATATGGGTCGCT
TRINITY_DN32_c0_g1_i2           GACACCACGCTTTGCGATCAATCCGAGAGCTGTCGCAATGTTGCCATAATATGGGTCGCT
```

```
TRINITY_DN32_c0_g1_i4           GACACCACGCTTTGCGATCAATCCGAGAGCTGTCGCAATGTTGCCATAATATGGGTCGCT
TRINITY_DN32_c0_g1_i3           GACACCACGCTTTGCGATCAATCCGAGAGCTGTCGCAATGTTGCCATAATATGGGTCGCT
                                ************************************************************

MpDNV_DNA_RNA_seq_consensus     GACTTGCAGCGCTCCCCGTATCGCTTTTTTGTCTAATTCCCATATGTTATCCTTGCTAAC
TRINITY_DN2404_c0_g1_i4         GACTTGCAGCGCTCCCCGTATCGCTTTTTTGTCTAATTCCCATATGTTATCCTTGCTAAC
TRINITY_DN2404_c0_g1_i2         GACTTGCAGCGCTCCCCGTATCGCTTTTTTGTCTAATTCCCATATGTTATCCTTGCTAAC
TRINITY_DN2404_c0_g1_i1         GACTTGCAGCGCTCCCCGTATCGCTTTTTTGTCTAATTCCCATATGTTATCCTTGCTAAC
TRINITY_DN2404_c0_g1_i3         GACTTGCAGCGCTCCCCGTATCGCTTTTTTGTCTAATTCCCATATGTTATCCTTGCTAAC
TRINITY_DN2570_c0_g3_i4         GACTTGCAGCGCTCCCCGTATCGCTTTTTTGTCTAATTCCCATATGTTATCCTTGCTAAC
TRINITY_DN2570_c0_g3_i3         GACTTGCAGCGCTCCCCGTATCGCTTTTTTGTCTAATTCCCATATGTTATCCTTGCTAAC
TRINITY_DN2570_c0_g3_i2         GACTTGCAGCGCTCCCCGTATCGCTTTTTTGTCTAATTCCCATATGTTATCCTTGCTAAC
TRINITY_DN2570_c0_g3_i1         GACTTGCAGCGCTCCCCGTATCGCTTTTTTGTCTAATTCCCATATGTTATCCTTGCTAAC
TRINITY_DN32_c0_g1_i1           GACTTGCAGCGCTCCCCGTATCGCTTTTTTGTCTAATTCCCATATGTTATCCTTGCTAAC
TRINITY_DN32_c0_g1_i2           GACTTGCAGCGCTCCCCGTATCGCTTTTTTGTCTAATTCCCATATGTTATCCTTGCTAAC
TRINITY_DN32_c0_g1_i4           GACTTGCAGCGCTCCCCGTATCGCTTTTTTGTCTAATTCCCATATGTTATCCTTGCTAAC
TRINITY_DN32_c0_g1_i3           GACTTGCAGCGCTCCCCGTATCGCTTTTTTGTCTAATTCCCATATGTTATCCTTGCTAAC
                                ************************************************************

MpDNV_DNA_RNA_seq_consensus     ATCCGTAGCATTAAAATAATCGGCATCGTGTTGAAATGCGACCTTATCTATATCACTAGT
TRINITY_DN2404_c0_g1_i4 APFV-11 ATCCGTAGCATTAAAATAATCGGCATCGTGTTGAAATGCGACCTTATCTATATCACTAGT
TRINITY_DN2404_c0_g1_i2         ATCCGTAGCATTAAAATAATCGGCATCGTGTTGAAATGCGACCTTATCTATATCACTAGT
TRINITY_DN2404_c0_g1_i1         ATCCGTAGCATTAAAATAATCGGCATCGTGTTGAAATGCGACCTTATCTATATCACTAGT
TRINITY_DN2404_c0_g1_i3         ATCCGTAGCATTAAAATAATCGGCATCGTGTTGAAATGCGACCTTATCTATATCACTAGT
TRINITY_DN2570_c0_g3_i4 APFV-13 ATCCGTAGCATTAAAATAATCGGCATCGTGTTGGAATGCGACCTTATCTATATCACTAGT
TRINITY_DN2570_c0_g3_i3         ATCCGTAGCATTAAAATAATCGGCATCGTGTTGGAATGCGACCTTATCTATATCACTAGT
TRINITY_DN2570_c0_g3_i2         ATCCGTAGCATTAAAATAATCGGCATCGTGTTGGAATGCGACCTTATCTATATCACTAGT
TRINITY_DN2570_c0_g3_i1         ATCCGTAGCATTAAAATAATCGGCATCGTGTTGGAATGCGACCTTATCTATATCACTAGT
TRINITY_DN32_c0_g1_i1   APFV-12 ATCCGTAGCATTAAAATAATCGGCATCGTGTTGGAATGCGACCTTATCTATATCACTAGT
TRINITY_DN32_c0_g1_i2           ATCCGTAGCATTAAAATAATCGGCATCGTGTTGGAATGCGACCTTATCTATATCACTAGT
TRINITY_DN32_c0_g1_i4           ATCCGTAGCATTAAAATAATCGGCATCGTGTTGGAATGCGACCTTATCTATATCACTAGT
TRINITY_DN32_c0_g1_i3           ATCCGTAGCATTAAAATAATCGGCATCGTGTTGGAATGCGACCTTATCTATATCACTAGT
                                ********************************* **************************

MpDNV_DNA_RNA_seq_consensus     AGGTAACGCCTTAGCATTAAAATGACCTTCACTGTCACACAACAGATTACCTGGACCCAC
TRINITY_DN2404_c0_g1_i4         AGGTAACGCCTTAGCATTAAAATGACCTTCACTGTCACACAACAGATTACCTGGACCCAC
TRINITY_DN2404_c0_g1_i2         AGGTAACGCCTTAGCATTAAAATGACCTTCACTGTCACACAACAGATTACCTGGACCCAC
TRINITY_DN2404_c0_g1_i1         AGGTAACGCCTTAGCATTAAAATGACCTTCACTGTCACACAACAGATTACCTGGACCCAC
TRINITY_DN2404_c0_g1_i3         AGGTAACGCCTTAGCATTAAAATGACCTTCACTGTCACACAACAGATTACCTGGACCCAC
TRINITY_DN2570_c0_g3_i4         AGGTAACGCCTTAGCATTAAAATGACCTTCACTGTCACACAACAGATTACCTGGACCCAC
TRINITY_DN2570_c0_g3_i3         AGGTAACGCCTTAGCATTAAAATGACCTTCACTGTCACACAACAGATTACCTGGACCCAC
TRINITY_DN2570_c0_g3_i2         AGGTAACGCCTTAGCATTAAAATGACCTTCACTGTCACACAACAGATTACCTGGACCCAC
TRINITY_DN2570_c0_g3_i1         AGGTAACGCCTTAGCATTAAAATGACCTTCACTGTCACACAACAGATTACCTGGACCCAC
TRINITY_DN32_c0_g1_i1           AGGTAACGCCTTAGCATTAAAATGACCTTCACTGTCACACAACAGATTACCTGGACCCAC
TRINITY_DN32_c0_g1_i2           AGGTAACGCCTTAGCATTAAAATGACCTTCACTGTCACACAACAGATTACCTGGACCCAC
TRINITY_DN32_c0_g1_i4           AGGTAACGCCTTAGCATTAAAATGACCTTCACTGTCACACAACAGATTACCTGGACCCAC
TRINITY_DN32_c0_g1_i3           AGGTAACGCCTTAGCATTAAAATGACCTTCACTGTCACACAACAGATTACCTGGACCCAC
                                ************************************************************

MpDNV_DNA_RNA_seq_consensus     GAAGCTACTACCGAACCACGTGATACCTTCATTGCTAGTACCTGAAGCACCTAATTTAAT
TRINITY_DN2404_c0_g1_i4         GAAGCTACTACCGAACCACGTGATACCTTCATTGCTAGTACCTGAAGCACCTAATTTAAT
TRINITY_DN2404_c0_g1_i2         GAAGCTACTACCGAACCACGTGATACCTTCATTGCTAGTACCTGAAGCACCTAATTTAAT
TRINITY_DN2404_c0_g1_i1         GAAGCTACTACCGAACCACGTGATACCTTCATTGCTAGTACCTGAAGCACCTAATTTAAT
TRINITY_DN2404_c0_g1_i3         GAAGCTACTACCGAACCACGTGATACCTTCATTGCTAGTACCTGAAGCACCTAATTTAAT
TRINITY_DN2570_c0_g3_i4         GAAGCTACTACCGAACCACGTGATACCTTCATTGCTAGTACCTGAAGCACCTAATTTAAT
TRINITY_DN2570_c0_g3_i3         GAAGCTACTACCGAACCACGTGATACCTTCATTGCTAGTACCTGAAGCACCTAATTTAAT
TRINITY_DN2570_c0_g3_i2         GAAGCTACTACCGAACCACGTGATACCTTCATTGCTAGTACCTGAAGCACCTAATTTAAT
TRINITY_DN2570_c0_g3_i1         GAAGCTACTACCGAACCACGTGATACCTTCATTGCTAGTACCTGAAGCACCTAATTTAAT
TRINITY_DN32_c0_g1_i1           GAAGCTACTACCGAACCACGTGATACCTTCATTGCTAGTACCTGAAGCACCTAATTTAAT
TRINITY_DN32_c0_g1_i2           GAAGCTACTACCGAACCACGTGATACCTTCATTGCTAGTACCTGAAGCACCTAATTTAAT
TRINITY_DN32_c0_g1_i4           GAAGCTACTACCGAACCACGTGATACCTTCATTGCTAGTACCTGAAGCACCTAATTTAAT
TRINITY_DN32_c0_g1_i3           GAAGCTACTACCGAACCACGTGATACCTTCATTGCTAGTACCTGAAGCACCTAATTTAAT
                                ************************************************************

MpDNV_DNA_RNA_seq_consensus     ACGTTTTCTCTCGTTATTCTCCTCTTCAACACTATAAAATTCGTCGTTTACCGACGACAT
TRINITY_DN2404_c0_g1_i4         ACGTTTTCTCTCGTTATTCTCCTCTTCAACACTATAAAATTCGTCGTTTACCGACGACAT
TRINITY_DN2404_c0_g1_i2         ACGTTTTCTCTCGTTATTCTCCTCTTCAACACTATAAAATTCGTCGTTTACCGACGACAT
TRINITY_DN2404_c0_g1_i1         ACGTTTTCTCTCGTTATTCTCCTCTTCAACACTATAAAATTCGTCGTTTACCGACGACAT
TRINITY_DN2404_c0_g1_i3         ACGTTTTCTCTCGTTATTCTCCTCTTCAACACTATAAAATTCGTCGTTTACCGACGACAT
TRINITY_DN2570_c0_g3_i4         ACGTTTTCTCTCGTTATTCTCCTCTTCAACACTATAAAATTCGTCGTTTACCGACGACAT
TRINITY_DN2570_c0_g3_i3         ACGTTTTCTCTCGTTATTCTCCTCTTCAACACTATAAAATTCGTCGTTTACCGACGACAT
TRINITY_DN2570_c0_g3_i2         ACGTTTTCTCTCGTTATTCTCCTCTTCAACACTATAAAATTCGTCGTTTACCGACGACAT
TRINITY_DN2570_c0_g3_i1         ACGTTTTCTCTCGTTATTCTCCTCTTCAACACTATAAAATTCGTCGTTTACCGACGACAT
TRINITY_DN32_c0_g1_i1           ACGTTTTCTCTCGTTATTCTCCTCTTCAACACTATAAAATTCGTCGTTTACCGACGACAT
TRINITY_DN32_c0_g1_i2           ACGTTTTCTCTCGTTATTCTCCTCTTCAACACTATAAAATTCGTCGTTTACCGACGACAT
TRINITY_DN32_c0_g1_i4           ACGTTTTCTCTCGTTATTCTCCTCTTCAACACTATAAAATTCGTCGTTTACCGACGACAT
TRINITY_DN32_c0_g1_i3           ACGTTTTCTCTCGTTATTCTCCTCTTCAACACTATAAAATTCGTCGTTTACCGACGACAT
                                ************************************************************

MpDNV_DNA_RNA_seq_consensus     GTTAAACGTAACTGATTAAATATAAGTAAAATGTAAACACTTGAGTAAATAGTGATGACG
TRINITY_DN2404_c0_g1_i4         GTTAAACGTAACTGATTAAATATAAGTAAAATGTAAACACTTGAGTAAATAGTGATGACG
TRINITY_DN2404_c0_g1_i2         GTTAAACGTAACTGATTAAATATAAGTAAAATGTAAACACTTGAGTAAATAGTGATGACG
TRINITY_DN2404_c0_g1_i1         GTTAAACGTAACTGATTAAATATAAGTAAAATGTAAACACTTGAGTAAATAGTGATGACG
TRINITY_DN2404_c0_g1_i3         GTTAAACGTAACTGATTAAATATAAGTAAAATGTAAACACTTGAGTAAATAGTGATGACG
TRINITY_DN2570_c0_g3_i4         GTTAAACGTAACTGATTAAATATAAGTAAAATGTAAACACTTGAGTAAATAGTGATGACG
TRINITY_DN2570_c0_g3_i3         GTTAAACGTAACTGATTAAATATAAGTAAAATGTAAACACTTGAGTAAATAGTGATGACG
TRINITY_DN2570_c0_g3_i2         GTTAAACGTAACTGATTAAATATAAGTAAAATGTAAACACTTGAGTAAATAGTGATGACG
TRINITY_DN2570_c0_g3_i1         GTTAAACGTAACTGATTAAATATAAGTAAAATGTAAACACTTGAGTAAATAGTGATGACG
```

```
TRINITY_DN32_c0_g1_i1            GTTAAACGTAACTGATTAAATATAAGTAAAATGTAAACACTTGAGTAAATAGTGATGACG
TRINITY_DN32_c0_g1_i2            GTTAAACGTAACTGATTAAATATAAGTAAAATGTAAACACTTGAGTAAATAGTGATGACG
TRINITY_DN32_c0_g1_i4            GTTAAACGTAACTGATTAAATATAAGTAAAATGTAAACACTTGAGTAAATAGTGATGACG
TRINITY_DN32_c0_g1_i3            GTTAAACGTAACTGATTAAATATAAGTAAAATGTAAACACTTGAGTAAATAGTGATGACG
                                 ************************************************************

MpDNV_DNA_RNA_seq_consensus      TCACCCCCACCTAGTCGCCTACTGGATACTTGTCTATCTTTTTATTATATAAAAGATCC 3'-ITR TATA-box
TRINITY_DN2404_c0_g1_i4  APFV-11 TCACCCCCACCCAGTCGCCCACTGGATACCCGTCTATCCCCTTACCACACAAAAGATCC
TRINITY_DN2404_c0_g1_i2          TCACCCCCACCCAGTCGCCCACTGGATACCCGTCTATCCCCTTACCACACAAAAGATCC
TRINITY_DN2404_c0_g1_i1          TCACCCCCACCCAGTCGCCCACTGGATACCCGTCTATCCCCTTACCACACAAAAGATCC
TRINITY_DN2404_c0_g1_i3          TCACCCCCACCCAGTCGCCCACTGGATACCCGTCTATCCCCTTACCACACAAAAGATCC
TRINITY_DN2570_c0_g3_i4  APFV-13 TCACCCCCACCTAGTCGCCTACTGGATACTTGTCTATCTTTTTATTATATAAAAGATCC
TRINITY_DN2570_c0_g3_i3          TCACCCCCACCTAGTCGCCTACTGGATACTTGTCTATCTTTTTATTATATAAAAGATCC
TRINITY_DN2570_c0_g3_i2          TCACCCCCACCTAGTCGCCTACTGGATACTTGTCTATCTTTTTATTATATAAAAGATCC
TRINITY_DN2570_c0_g3_i1          TCACCCCCACCTAGTCGCCTACTGGATACTTGTCTATCTTTTTATTATATAAAAGATCC
TRINITY_DN32_c0_g1_i1    APFV-12 TCACCCCCACCTAGTCGCCTACTGGATACTACTT---------------CAGGGTCATCG
TRINITY_DN32_c0_g1_i2            TCACCCCCACCTAGTCGCCTACTGGATACTACTT---------------CAGGGTCATCG
TRINITY_DN32_c0_g1_i4            TCACCCCCACCTAGTCGCCTACTGGATACTACTT---------------CAGGGTCATCG
TRINITY_DN32_c0_g1_i3            TCACCCCCACCTAGTCGCCTACTGGATACTACTT---------------CAGGGTCATCG
                                 *********** ******* *********   *                 *     ***

MpDNV_DNA_RNA_seq_consensus      CCTACTAATAATAACCACTACCTACTTCCGGGGGGCGGGGGGTATCCTTTGTTATCTATA
TRINITY_DN2404_c0_g1_i4          CCC--------------------------------------------------------- VP 5'-end
TRINITY_DN2404_c0_g1_i2          CCC--------------------------------------------------------- VP 5'-end
TRINITY_DN2404_c0_g1_i1          CCC--------------------------------------------------------- VP 5'-end
TRINITY_DN2404_c0_g1_i3          CCC--------------------------------------------------------- VP 5'-end
TRINITY_DN2570_c0_g3_i4          CCCACTAATAATAACCACTACCTACTTCCGGGGGGCGGGGGGTATCCTTTGTTATCTATA
TRINITY_DN2570_c0_g3_i3          CCCACTAATAATAACCACTACCTACTTCCGGGGGGCGGGGGGTATCCTTTGTTATCTATA
TRINITY_DN2570_c0_g3_i2          CCCACTAATAATAACCACTACCTACTTCCGGGGGGCGGGGGGTATCCTTTGTTATCTATA
TRINITY_DN2570_c0_g3_i1          CCCACTAATAATAACCACTACCTACTTCCGGGGGGCGGGGGGTATCCTTTGTTATCTATA
TRINITY_DN32_c0_g1_i1            C----------------------------------------------------------- VP 5'-end
TRINITY_DN32_c0_g1_i2            C----------------------------------------------------------- VP 5'-end
TRINITY_DN32_c0_g1_i4            C----------------------------------------------------------- VP 5'-end
TRINITY_DN32_c0_g1_i3            C----------------------------------------------------------- VP 5'-end
                                 *

MpDNV_DNA_RNA_seq_consensus      CTTATTGTCAACACTAGCCTTATCTTAGGGCGTGTCGGCCGACACGTGTCATGGCCATGA
TRINITY_DN2404_c0_g1_i4          ------------------------------------------------------------
TRINITY_DN2404_c0_g1_i2          ------------------------------------------------------------
TRINITY_DN2404_c0_g1_i1          ------------------------------------------------------------
TRINITY_DN2404_c0_g1_i3          ------------------------------------------------------------
TRINITY_DN2570_c0_g3_i4          CTTATTGTCAACACTAGCCTTATCTTAGGGCTAGTGTCATGG------------------ VP 5'-end
TRINITY_DN2570_c0_g3_i3          CTTATTGTCAACACTAGCCTTATCTTAGGGCTAGTGTCATGG------------------ VP 5'-end
TRINITY_DN2570_c0_g3_i2          CTTATTGTCAACACTAGCCTTATCTTAGGGCTAGTGTCATGG------------------ VP 5'-end
TRINITY_DN2570_c0_g3_i1          CTTATTGTCAACACTAGCCTTATCTTAGGGCTAGTGTCATGG------------------ VP 5'-end
TRINITY_DN32_c0_g1_i1            ------------------------------------------------------------
TRINITY_DN32_c0_g1_i2            ------------------------------------------------------------
TRINITY_DN32_c0_g1_i4            ------------------------------------------------------------
TRINITY_DN32_c0_g1_i3            ------------------------------------------------------------
```

APFV-14-15-16 (*A. thaliana* TuYV-infected)

```
TRINITY_DN7_c0_g2_i1  APFV-14 --------------------------ATAAGGCTAGTGTTGACAATAAGTATAGATAACA VP 5'-end
MpDNV_mRNA_seq_consensus      GTCATGGCCATGACACTAGCCCTAAGATAAGGCTAGTGTTGACAATAAGTATAGATAACA
TRINITY_DN266_c0_g2_i1 APFV16 -----------------------------------------------------GATAACA VP 5'-end
TRINITY_DN5346_c0_g1_i1 APFV15------------------------------------------------------------
TRINITY_DN5346_c0_g1_i2 APFV15------------------------------------------------------------

TRINITY_DN7_c0_g2_i1          A-GGATACCCCCCGCCCCCCGGAAGTAGGTAGTGGTTATTATTAGTGGGGGATCTTTTTA
MpDNV_mRNA_seq_consensus      A-GGATACCCCCCGCCCCCCGGAAGTAGGTAGTGGTTATTATTAGTAGGGGATCTTTTTA 5'ITR TATA-box
TRINITY_DN266_c0_g2_i1        AAGGATACCCCCCGCCCCCCGGAAGGAGGTAGTGGTTATTATTAGTAGGGGATCTTTTTA
TRINITY_DN5346_c0_g1_i1       ------------------------------------------------------------
TRINITY_DN5346_c0_g1_i2       ------------------------------------------------------------

TRINITY_DN7_c0_g2_i1          TATAATAAAAAGATAGGCAAGTATCCAGTAGGCGACTAGGTGGGGGTGACGTCATCACTA
MpDNV_mRNA_seq_consensus      TATAATAAAAAGATAGACAAGTATCCAGTAGGCGACTAGGTGGGGGTGACGTCATCACTA
TRINITY_DN266_c0_g2_i1        TATAATAAAAAGATAGACAAGTATCCAGTAGGCGACTAGGTGGGGGTGACGTCATCACTA
TRINITY_DN5346_c0_g1_i1       -----------------------------AGGCGACTAGGTGGGGGTGACGTCATCACTA VP 5'-end
TRINITY_DN5346_c0_g1_i2       -----------------------------AGGCGACTAGGTGGGGGTGACGTCATCACTA VP 5'-end
                                                           *******************************

TRINITY_DN7_c0_g2_i1          TTTACTCAAGTGTTTTGTTATTCGGCGTCAAGTGTTGTGTTGACACTTGAAAAGTGGCGT
MpDNV_mRNA_seq_consensus      TTTACTCAAGTGTTTTGTTATTCGGCGTCAAGTGTTGTGTTGACACTTGAAAAGTGGCGT
TRINITY_DN266_c0_g2_i1        TTTACTCAAGTGTTTTGTTATTCGGCGTCAAGTGTTGTGTTGACACTTGAAAAGTGGCGT
TRINITY_DN5346_c0_g1_i1       TTTACTCAAGTGTTTTGTTATTCGGCGTCAAGTGTTGTGTTGACACTTGAAAAGTGGCGT
TRINITY_DN5346_c0_g1_i2       TTTACTCAAGTGTTTTGTTATTCGGCGTCAAGTGTTGTGTTGACACTTGAAAAGTGGCGT
                              ************************************************************

TRINITY_DN7_c0_g2_i1   APFV14 GATTGATTATTTACAAGTACGTCAGATAAGATAAACATAAGTGGTACTTGACTCGGAATA
MpDNV_mRNA_seq_consensus      GATTGATTATTTACAAGTACGTCAGATAAGATAAACATAAGTGGTACTTGACTCGGAATA
TRINITY_DN266_c0_g2_i1 APFV16 GATTGATTATCTACAAGTACGTCAGATAAGATAAACATAAGTGGTACTTGACTCGGAATA
TRINITY_DN5346_c0_g1_i1APFV15 GATTGATTATCTACAAGTACGTCAGATAAGATAAACATAAGTGGTACTTGACTCGGAATA
TRINITY_DN5346_c0_g1_i2APFV15 GATTGATTATCTACAAGTACGTCAGATAAGATAAACATAAGTGGTACTTGACTCGGAATA
                              ********** *************************************************
```

```
TRINITY_DN7_c0_g2_i1          TATAATGAAGTGTGGAACCTACAACAATATCAGTACTCATCAAAATGTCAAACTCGCAAG
MpDNV_mRNA_seq_consensus      TATAATGAAGTGTGGAACCTACAACAATATCAGTACTCATCAAAATGTCAAACTCGCAAG
TRINITY_DN266_c0_g2_i1        TATAATGAAGTGTGGAACCTACAACAATATCAGTACTCATCAAAATGTCAAACTCGCAAG
TRINITY_DN5346_c0_g1_i1       TATAATGAAGTGTGGAACCTACAACAATATCAGTACTCATCAAAATGTCAAACTCGCAAG
TRINITY_DN5346_c0_g1_i2       TATAATGAAGTGTGGAACCTACAACAATATCAGTACTCATCAAAATGTCAAACTCGCAAG
                              ************************************************************

TRINITY_DN7_c0_g2_i1          AAGTGAACCAACAACAATTCAACGACGAGGAGGAAATACCATGTGGCCAACGGTATCCTA
MpDNV_mRNA_seq_consensus      AAGTGAACCAACAACAATTCAACGACGAGGAGGAAATACCATGTGGCCAACGGTATCCTA
TRINITY_DN266_c0_g2_i1        AAGTGAACCAACAACAATTCAACGACGAGGAGGAAATACCATGTGGCCAACGGTATCCTA
TRINITY_DN5346_c0_g1_i1       AAGTGAACCAACAACAATTCAACGACGAGGAGGAAATACCATGTGGCCAACGGTATCCTA
TRINITY_DN5346_c0_g1_i2       AAGTGAACCAACAACAATTCAACGACGAGGAGGAAATACCATGTGGCCAACGGTATCCTA
                              ************************************************************

TRINITY_DN7_c0_g2_i1          CAGCGGCGACGATTCAAACGGACCACCAACAGACTGGCGTGAATGGATCGACCCCGATGT
MpDNV_mRNA_seq_consensus      CAGCGGCGACGATTCAAACGGACCACCAACAGACTGGCGTGAATGGATCGACCCCGATGT
TRINITY_DN266_c0_g2_i1        CAGCGGCGACGATTCAAACGGACCACCAACAGAGTGGCGTGAATGGATCGACCCCGATGT
TRINITY_DN5346_c0_g1_i1       CAGCGGCGACGATTCAAACGGACCACCAACAGAGTGGCGTGAATGGATCGACCCCGATGT
TRINITY_DN5346_c0_g1_i2       CAGCGGCGACGATTCAAACGGACCACCAACAGAGTGGCGTGAATGGATCGACCCCGATGT
                              ********************************* **************************

TRINITY_DN7_c0_g2_i1          TCGAACTCAAATCGACAGCGCGCAAAGAATATGGGACAGCCATTTCAGCGGACACGGCAC
MpDNV_mRNA_seq_consensus      TCGAACTCAAATCGACAGCGCGCAAAGAATATGGGACAGCCATTTCAGCGGACACGGCAC
TRINITY_DN266_c0_g2_i1        TCGAACTCAAATCGACAGCGCGCAAAGAATATGGGACAGCCATTTCAGCGGACACGGCAC
TRINITY_DN5346_c0_g1_i1       TCGAACTCAAATCGACAGCGCGCAAAGAATATGGGACAGCCATTTCAGCGGACACGGCAC
TRINITY_DN5346_c0_g1_i2       TCGAACTCAAATCGACAGCGCGCAAAGAATATGGGACAGCCATTTCAGCGGACACGGCAC
                              ************************************************************

TRINITY_DN7_c0_g2_i1          TGACACTGAAGACGCTGTGCAAAGTGTTGGAGTGCAAGGCAACGGAACTAACGGTAACAT
MpDNV_mRNA_seq_consensus      TGACACTGAAGACGCTGTGCAAAGTGTTGGAGTGCAAGGCAACGGAACTAACGGTAACAT
TRINITY_DN266_c0_g2_i1        TGACACTGAAGACGCTGTGCAAAGTGTTGGAGTGCAAGGCAACGGAACTAACGGTAACAT
TRINITY_DN5346_c0_g1_i1       TGACACTGAAGACGCTGTGCAAAGTGTTGGAGTGCAAGGCAACGGAACTAACGGTAACAT
TRINITY_DN5346_c0_g1_i2       TGACACTGAAGACGCTGTGCAAAGTGTTGGAGTGCAAGGCAACGGAACTAACGGTAACAT
                              ************************************************************

TRINITY_DN7_c0_g2_i1          GCATTATTCCTCTGATCGCGTTGCTATTACCCGACCATATGTGGTCAGAGGCGACGAGCA
MpDNV_mRNA_seq_consensus      GCATTATTCCTCTGATCGCGTTGCTATTACCCGACCATATGTGGTCAGAGGCGACGAGCA
TRINITY_DN266_c0_g2_i1        GCATTATTCCTCTGATCGCGTTGCTATTACCCGACCATATGTGGTCAGAGGCGACGAGCA
TRINITY_DN5346_c0_g1_i1       GCATTATTCCTCTGATCGCGTTGCTATTACCCGACCATATGTGGTCAGAGGCGACGAGCA
TRINITY_DN5346_c0_g1_i2       GCATTATTCCTCTGATCGCGTTGCTATTACCCGACCATATGTGGTCAGAGGCGACGAGCA
                              ************************************************************

TRINITY_DN7_c0_g2_i1          GCGATCGCTGGCGATACCGACTGAGAAACGCGACCTACTGGATTTCCAATCCAACGCGGG
MpDNV_mRNA_seq_consensus      GCGATCGCTGGCGATACCGACTGAGAAACGCGACCTACTGGATTTCCAATCCAACGCGGG
TRINITY_DN266_c0_g2_i1        GCGATCGCTGGCGATACCGACTGAGAAACGCGACCTACTGGATTTCCAATCCAACGCGGG
TRINITY_DN5346_c0_g1_i1       GCGATCGCTGGCGATACCGACTGAGAAACGCGACCTACTGGATTTCCAATCCAACGCGGG
TRINITY_DN5346_c0_g1_i2       GCGATCGCTGGCGATACCGACTGAGAAACGCGACCTACTGGATTTCCAATCCAACGCGGG
                              ************************************************************

TRINITY_DN7_c0_g2_i1          AATCCGAATATCAGGCTTTACTACGATGGATGGAGGAGAAAATGCCGATACCTTTAGCGA
MpDNV_mRNA_seq_consensus      AATCCGAATATCAGGCTTTACTACGATGGATGGAGGAGAAAATGCCGATACCTTTAGCGA
TRINITY_DN266_c0_g2_i1        AATCCGAATATCAGGCTTTACTACGATGGATGGAGGAGAAAATGCCGATACCTTTAGCGA
TRINITY_DN5346_c0_g1_i1       AATCCGAATATCAGGCTTTACTACGATGGATGGAGGAGAAAATGCCGATACCTTTAGCGA
TRINITY_DN5346_c0_g1_i2       AATCCGAATATCAGGCTTTACTACGATGGATGGAGGAGAAAATGCCGATACCTTTAGCGA
                              ************************************************************

TRINITY_DN7_c0_g2_i1          AATGGCAAGGTGCATGTCTACCGCGGTATGGGGAGGCAATGTCAGATATGTTTCTGACAT
MpDNV_mRNA_seq_consensus      AATGGCAAGGTGCATGTCTACCGCGGTATGGGGAGGCAATGTCAGATATGTTTCTGACAT
TRINITY_DN266_c0_g2_i1        AATGGCAAGGTGCATGTCTACCGCGGTATGGGGAGGCAATGTCAGATATGTTTCTGACAT
TRINITY_DN5346_c0_g1_i1       AATGGCAAGGTGCATGTCTACCGCGGTATGGGGAGGCAATGTCAGATATGTTTCTGACAT
TRINITY_DN5346_c0_g1_i2       AATGGCAAGGTGCATGTCTACCGCGGTATGGGGAGGCAATGTCAGATATGTTTCTGACAT
                              ************************************************************

TRINITY_DN7_c0_g2_i1          CCTTGTACCCGGAGGAAATTGGTCAATTAATGGAATTGTTGAGGACCTACGCCGGAGTAT
MpDNV_mRNA_seq_consensus      CCTTGTACCCGGAGGAAATTGGTCAATTAATGGAATTGTTGAGGACCTACGCCGGAGTAT
TRINITY_DN266_c0_g2_i1        CCTTGTACCCGGAGGAAATTGGTCAATTAATGGAATTGTTGAGGACCTACGCCGGAGTAT
TRINITY_DN5346_c0_g1_i1       CCTTGTACCCGGAGGAAATTGGTCAATTAATGGAATTGTTGAGGACCTACGCCGGAGTAT
TRINITY_DN5346_c0_g1_i2       CCTTGTACCCGGAGGAAATTGGTCAATTAATGGAATTGTTGAGGACCTACGCCGGAGTAT
                              ************************************************************

TRINITY_DN7_c0_g2_i1          CGCAGAGCGAATGCCAAAATGCATGTACGTCGTCAGCGAACACGGCGACCACGTCCACGT
MpDNV_mRNA_seq_consensus      CGCAGAGCGAATGCCAAAATGCATGTACGTCGTCAGCGAACACGGCGACCACGTCCACGT
TRINITY_DN266_c0_g2_i1        CGCAGAGCGAATGCCAAAATGCATGTACGTCGTCAGCGAACACGGCGACCACGTCCACGT
TRINITY_DN5346_c0_g1_i1       CGCAGAGCGAATGCCAAAATGCATGTACGTCGTCAGCGAACACGGCGACCACGTCCACGT
TRINITY_DN5346_c0_g1_i2       CGCAGAGCGAATGCCAAAATGCATGTACGTCGTCAGCGAACACGGCGACCACGTCCACGT
                              ************************************************************

TRINITY_DN7_c0_g2_i1          CGTACACACCTGTAACTACGCCACGGGCAGCTGTAGATGTAGTTTCTTACTCAACGCCGC
MpDNV_mRNA_seq_consensus      CGTACACACCTGTAACTACGCCACGGGCAGCTGTAGATGTAGTTTCTTACTCAACGCCGC
TRINITY_DN266_c0_g2_i1        CGTACACACCTGTAACTACGCCACGGGCAGCTGTAGATGTAGTTTCTTACTCAACGCCGC
TRINITY_DN5346_c0_g1_i1       CGTACACACCTGTAACTACGCCACGGGCAGCTGTAGATGTAGTTTCTTACTCAACGCCGC
TRINITY_DN5346_c0_g1_i2       CGTACACACCTGTAACTACGCCACGGGCAGCTGTAGATGTAGTTTCTTACTCAACGCCGC
                              ************************************************************

TRINITY_DN7_c0_g2_i1          TGCTTTCCAACTATGTAGACGACGACGCATTCGAAAGAATGTTAGAGTCATCCAACTATC
MpDNV_mRNA_seq_consensus      TGCTTTCCAACTATGTAGACGACGACGCATTCGAAAGAATGTTAGAGTCATCCAACTATC
TRINITY_DN266_c0_g2_i1        TGCTTTCCAACTATGTAGACGACGACGCATTCGAAAGAATGTTAGAGTCATCCAACTATC
TRINITY_DN5346_c0_g1_i1       TGCTTTCCAACTATGTAGACGACGACGCATTCGAAAGAATGTTAGAGTCATCCAACTATC
```

```
TRINITY_DN5346_c0_g1_i2       TGCTTTCCAACTATGTAGACGACGACGCATTCGAAAGAATGTTAGAGTCATCCAACTATC
                              ************************************************************

TRINITY_DN7_c0_g2_i1          AGAAAGAGACTGGTCCCGTATCTTTCAATACTTATGTTCATCGCCCAGGAATGTCAAAGA
MpDNV_mRNA_seq_consensus      AGAAAGAGACTGGTCCCGTATCTTTCAATACTTATGTTCATCGCCCAGGAATGTCAAAGA
TRINITY_DN266_c0_g2_i1        AGAAAGAGACTGGTCCCGTATCTTTCAATACTTATGTTCATCGCCCAGGAATGTCAAAGA
TRINITY_DN5346_c0_g1_i1       AGAAAGAGACTGGTCCCGTATCTTTCAATACTTATGTTCATCGCCCAGGAATGTCAAAGA
TRINITY_DN5346_c0_g1_i2       AGAAAGAGACTGGTCCCGTATCTTTCAATACTTATGTTCATCGCCCAGGAATGTCAAAGA
                              ************************************************************

TRINITY_DN7_c0_g2_i1          AGTGGGCGGCCAGTACTTTGATGGAGGATTACGTAGTAGATATTCACATATATCAGTTAG NS uspliced
MpDNV_mRNA_seq_consensus      AGTGGGCGGCCAGTACTTTGATGGAGGATTACGTAGTAGATATTCACATATATCAGTTAG 5'splice site
TRINITY_DN266_c0_g2_i1        AGTGGGCGGCCAGTACTTTGATGGAGGATTACGTAGTAGATATTCACATATATCAGTTAG NS uspliced
TRINITY_DN5346_c0_g1_i1       AGTGGGCGGCCAGTACTTTGATGGAGGATTACGTAGTAGATATTCACATATATCA----- NS spliced
TRINITY_DN5346_c0_g1_i2       AGTGGGCGGCCAGTACTTTGATGGAGGATTACGTAGTAGATATTCACATATATCAGTTAG NS uspliced
                              *******************************************************

TRINITY_DN7_c0_g2_i1          TATTGCGTAATTTTTTTATATACTTGGATTGGTTGACGGGTATAGAAGCGAATAGATGCG
MpDNV_mRNA_seq_consensus      TATTGCGTAATTTTTTTATATACTTGGATTGGTTGACGGGTATAGAAGCGAATAGATGCG
TRINITY_DN266_c0_g2_i1        TATTGCGTAATTTTTTTATATACTTGGATTGGTTGACGGGTATAGAAGCGAATAGATGCG
TRINITY_DN5346_c0_g1_i1       ------------------------------------------------------------
TRINITY_DN5346_c0_g1_i2       TATTGCGTAATTTTTTTATATACTTGGATTGGTTGACGGGTATAGAAGCGAATAGATGCG

TRINITY_DN7_c0_g2_i1          TTTCATATATTAAAACTGGTATGCCTGGCAGGAGTTACGTTAAAAGCGTGATGCCAAAAA
MpDNV_mRNA_seq_consensus      TTTCATATATTAAAACTGGTATGCCTGGCAGGAGTTACGTTAAAAGCGTGATGCCAAAAA
TRINITY_DN266_c0_g2_i1        TTTCATATATTAAAACTGGTATGCCTGGCAGGAGTTACGTTAAAAGCGTGATGCCAAAAA
TRINITY_DN5346_c0_g1_i1       ------------------------------------------------------------
TRINITY_DN5346_c0_g1_i2       TTTCATATATTAAAACTGGTATGCCTGGCAGGAGTTACGTTAAAAGCGTGATGCCAAAAA

TRINITY_DN7_c0_g2_i1          ATTATTGGCGTAATCCTAAAGAATTTCACTTAGCGCTTGATAAATTAGAGTTGCTCGTTA
MpDNV_mRNA_seq_consensus      ATTATTGGCGTAATCCTAAAGAATTTCACTTAGCGCTTGATAAATTAGAGTTGCTCGTTA
TRINITY_DN266_c0_g2_i1        ATTATTGGCGTAATCCTAAAGAATTTCACTTAGCGCTTGATAAATTAGAGTTGCTCGTTA
TRINITY_DN5346_c0_g1_i1       ------------------------------------------------------------
TRINITY_DN5346_c0_g1_i2       ATTATTGGCGTAATCCTAAAGAATTTCACTTAGCGCTTGATAAATTAGAGTTGCTCGTTA

TRINITY_DN7_c0_g2_i1          AACGACATTTCGGTGATTTCACGTGTTTGGAAAATTTTTTACCGCAATATCGTTGGCATA
MpDNV_mRNA_seq_consensus      AACGACATTTCGGTGATTTCACGTGTTTGGAAAATTTTTTACCGCAATATCGTTGGCATA
TRINITY_DN266_c0_g2_i1        AACGACATTTCGGTGATTTCACGTGTTTGGAAAATTTTTTACCGCAATATCGTTGGCATA
TRINITY_DN5346_c0_g1_i1       ------------------------------------------------------------
TRINITY_DN5346_c0_g1_i2       AACGACATTTCGGTGATTTCACGTGTTTGGAAAATTTTTTACCGCAATATCGTTGGCATA

TRINITY_DN7_c0_g2_i1          ATTGCATCACATGGTTTGTGAACGTGATGTCCTGTCCGTTGGACCCGTGGACTGTCGAAT
MpDNV_mRNA_seq_consensus      ATTGCATCACATGGTTTGTGAACGTGATGTCCTGTCCGTTGGACCCGTGGACTGTCGAAT
TRINITY_DN266_c0_g2_i1        ATTGCATCACATGGTTTGTGAACGTGATGTCCTGTCCGTTGGACCCGTGGACTGTCGAAT
TRINITY_DN5346_c0_g1_i1       ------------------------------------------------------------
TRINITY_DN5346_c0_g1_i2       ATTGCATCACATGGTTTGTGAACGTGATGTCCTGTCCGTTGGACCCGTGGACTGTCGAAT

TRINITY_DN7_c0_g2_i1          GTGCTGGTTGTGGTTTTCAACATATTCCTAATATATCTAAATGCGAACTACTACGAGACT
MpDNV_mRNA_seq_consensus      GTGCTGGTTGTGGTTTTCAACATATTCCTAATATATCTAAATGCGAACTACTACGAGACT
TRINITY_DN266_c0_g2_i1        GTGCTGGTTGTGGTTTTCAACATATTCCTAATATATCTAAATGCGAACTACTACGAGACT
TRINITY_DN5346_c0_g1_i1       ------------------------------------------------------------
TRINITY_DN5346_c0_g1_i2       GTGCTGGTTGTGGTTTTCAACATATTCCTAATATATCTAAATGCGAACTACTACGAGACT

TRINITY_DN7_c0_g2_i1          ATCCGTTCATGAACGAGGGCACTATAAATCATATACTAATCGAATGCATTAAAATTTACA
MpDNV_mRNA_seq_consensus      ATCCGTTCATGAACGAGGGCACTATAAATCATATACTAATCGAATGCATTAAAATTTACA
TRINITY_DN266_c0_g2_i1        ATCCGTTCATGAACGAGGGCACTATAAATCATATACTAATCGAATGCATTAAAATTTACA
TRINITY_DN5346_c0_g1_i1       ------------------------------------------------------------
TRINITY_DN5346_c0_g1_i2       ATCCGTTCATGAACGAGGGCACTATAAATCATATACTAATCGAATGCATTAAAATTTACA

TRINITY_DN7_c0_g2_i1          AACAGGAAGGATCAAATACAGAATGTCGACCCGAACGATTGGTGGAAACATGCCTTCTGC NS uspliced
MpDNV_mRNA_seq_consensus      AACAGGAAGGATCAAATACAGAATGTCGACCCGAACGATTGGTGGAAACATGCCTTCTGC 5'splice site
TRINITY_DN266_c0_g2_i1        AACAGGAAGGATCAAATACAGAATGTCGACCCGAACGATTGGTGGAAACATGCCTTCTGC NS uspliced
TRINITY_DN5346_c0_g1_i1       -----GAAGGATCAAATACAGAATGTCGACCCGAACGATTGGTGGAAACATGCCTTCTGC NS spliced
TRINITY_DN5346_c0_g1_i2       AACAGGAAGGATCAAATACAGAATGTCGACCCGAACGATTGGTGGAAACATGCCTTCTGC NS uspliced
                                   *******************************************************

TRINITY_DN7_c0_g2_i1          AGGGCTCGGGGGAGCTTCAATCTGAACAGTCCATACGTGAAAATGGCCAAAACCATACAA
MpDNV_mRNA_seq_consensus      AGGGCTCGGGGGAGCTTCAATCTGAACAGTCCATACGTGAAAATGGCCAAAACCATACAA
TRINITY_DN266_c0_g2_i1        AGGGCTCGGGGGAGCTTCAATCTGAACAGTCCATACGTGAAAATGGCCAAAACCATACAA
TRINITY_DN5346_c0_g1_i1       AGGGCTCGGGGGAGCTTCAATCTGAACAGTCCATACGTGAAAATGGCCAAAACCATACAA
TRINITY_DN5346_c0_g1_i2       AGGGCTCGGGGGAGCTTCAATCTGAACAGTCCATACGTGAAAATGGCCAAAACCATACAA
                              ************************************************************

TRINITY_DN7_c0_g2_i1          CAGGAAGTGTTTCGCAGGATGACCAAGATGGAGTACAAGATAACGGATCCACAAATGTTT
MpDNV_mRNA_seq_consensus      CAGGAAGTGTTTCGCAGGATGACCAAGATGGAGTACAAGATAACGGATCCACAAATGTTT
TRINITY_DN266_c0_g2_i1        CAGGAAGTGTTTCGCAGGATGACCAAGATGGAGTACAAGATAACGGATCCACAAATGTTT
TRINITY_DN5346_c0_g1_i1       CAGGAAGTGTTTCGCAGGATGACCAAGATGGAGTACAAGATAACGGATCCACAAATGTTT
TRINITY_DN5346_c0_g1_i2       CAGGAAGTGTTTCGCAGGATGACCAAGATGGAGTACAAGATAACGGATCCACAAATGTTT
                              ************************************************************

TRINITY_DN7_c0_g2_i1          TTCAAGAAGAAGAAGAGGGCAGCCAAGAGGGCCCCGCGGCAAAAGAAGTCGCGACGGAAGC
```

```
MpDNV_mRNA_seq_consensus       TTCAAGAAGAAGAAGAGGGCAGCCAAGAGGGCCCGCGGCAAAAGAAGTCGCGACGGAAGC
TRINITY_DN266_c0_g2_i1         TTCAAGAAGAAGAAGAGGGCAGCCAAGAGGGCCCGCGGCAAAAGAAGTCGCGACGGAAGC
TRINITY_DN5346_c0_g1_i1        TTCAAGAAGAAGAAGAGGGCAGCCAAGAGGGCCCGCGGCAAAAGAAGTCGCGACGGAAGC
TRINITY_DN5346_c0_g1_i2        TTCAAGAAGAAGAAGAGGGCAGCCAAGAGGGCCCGCGGCAAAAGAAGTCGCGACGGAAGC
                               ************************************************************

TRINITY_DN7_c0_g2_i1           AGCGATTCCGAATGATTAGGGAAATAGATGTTAGTAAACCGCAATCTATCGAAGAACTTA
MpDNV_mRNA_seq_consensus       AGCGATTCCGAATGATTAGGGAAATAGATGTTAGTAAACCGCAATCTATCGAAGAACTTA
TRINITY_DN266_c0_g2_i1         AGCGATTCCGAATGATTAGGGAAATAGATGTTAGTAAACCGCAATCTATCGAAGAACTTA
TRINITY_DN5346_c0_g1_i1        AGCGATTCCGAATGATTAGGGAAATAGATGTTAGTAAACCGCAATCTATCGAAGAACTTA
TRINITY_DN5346_c0_g1_i2        AGCGATTCCGAATGATTAGGGAAATAGATGTTAGTAAACCGCAATCTATCGAAGAACTTA
                               ************************************************************

TRINITY_DN7_c0_g2_i1           TATATAGGTATCCTTGTTGCCCCCCGGAAGCTTTCTATAACATTCCCGAATTTTACGCTA
MpDNV_mRNA_seq_consensus       TATATAGGTATCCTTGTTGCCCCCCGGAAGCTTTCTATAACATTCCCGAATTTTACGCTA
TRINITY_DN266_c0_g2_i1         TATATAGGTATCCTTGTTGCCCCCCGGAAGCTTTCTATAACATTCCCGAATTTTACGCTA
TRINITY_DN5346_c0_g1_i1        TATATAGGTATCCTTGTTGCCCCCCGGAAGCTTTCTATAACATTCCCGAATTTTACGCTA
TRINITY_DN5346_c0_g1_i2        TATATAGGTATCCTTGTTGCCCCCCGGAAGCTTTCTATAACATTCCCGAATTTTACGCTA
                               ************************************************************

TRINITY_DN7_c0_g2_i1           ATACAAATATAAACTGGATAGACATGAAAGATTTTAAAGTAACGATACCGTTACGTAATT
MpDNV_mRNA_seq_consensus       ATACAAATATAAACTGGATAGACATGAAAGATTTTAAAGTAACGATACCGTTACGTAATT
TRINITY_DN266_c0_g2_i1         ATACAAATATAAACTGGATAGACATGAAAGATTTTAAAGTAACGATACCGTTACGTAATT
TRINITY_DN5346_c0_g1_i1        ATACAAATATAAACTGGATAGACATGAAAGATTTTAAAGTAACGATACCGTTACGTAATT
TRINITY_DN5346_c0_g1_i2        ATACAAATATAAACTGGATAGACATGAAAGATTTTAAAGTAACGATACCGTTACGTAATT
                               ************************************************************

TRINITY_DN7_c0_g2_i1           GGGCGGCCACTTTACGCAGATGGTCTATTCATGATTTCAATAAATATTATAATGACTCTA
MpDNV_mRNA_seq_consensus       GGGCGGCCACTTTACGCAGATGGTCTATTCATGATTTCAATAAATATTATAATGACTCTA
TRINITY_DN266_c0_g2_i1         GGGCGGCCACTTTACGCAGATGGTCTATTCATGATTTCAATAAATATTATAATGACTCTA
TRINITY_DN5346_c0_g1_i1        GGGCGGCCACTTTACGCAGATGGTCTATTCATGATTTCAATAAATATTATAATGACTCTA
TRINITY_DN5346_c0_g1_i2        GGGCGGCCACTTTACGCAGATGGTCTATTCATGATTTCAATAAATATTATAATGACTCTA
                               ************************************************************

TRINITY_DN7_c0_g2_i1           CAGTTTTTCCATATTTCAACGCGTACGGTTCAGATATTTGCAATATGTATTACAGTATTT
MpDNV_mRNA_seq_consensus       CAGTTTTTCCATATTTCAACGCGTACGGTTCAGATATTTGCAATATGTATTACAGTATTT
TRINITY_DN266_c0_g2_i1         CAGTTTTTCCATATTTCAACGCGTACGGTTCAGATATTTGCAATATGTATTACAGTATTT
TRINITY_DN5346_c0_g1_i1        CAGTTTTTCCATATTTCAACGCGTACGGTTCAGATATTTGCAATATGTATTACAGTATTT
TRINITY_DN5346_c0_g1_i2        CAGTTTTTCCATATTTCAACGCGTACGGTTCAGATATTTGCAATATGTATTACAGTATTT
                               ************************************************************

TRINITY_DN7_c0_g2_i1           CGGAGAGTCTGACAATAGCAAAAGAATTACTCAACTATCAATTTGGCGATGACCCTGAAG
MpDNV_mRNA_seq_consensus       CGGAGAGTCTGACAATAGCAAAAGAATTACTCAACTATCAATTTGGCGATGACCCTGAAG
TRINITY_DN266_c0_g2_i1         CGGAGAGTCTGACAATAGCAAAAGAATTACTCAACTATCAATTTGGCGATGACCCTGAAG
TRINITY_DN5346_c0_g1_i1        CGGAGAGTCTGACAATAGCAAAAGAATTACTCAACTATCAATTTGGCGATGACCCTGAAG
TRINITY_DN5346_c0_g1_i2        CGGAGAGTCTGACAATAGCAAAAGAATTACTCAACTATCAATTTGGCGATGACCCTGAAG
                               ************************************************************

TRINITY_DN7_c0_g2_i1           TAGTAATTGAATTTTTAACAACACTATATAATGTCATAGACAAAAGAGTTCCGAAATTAA
MpDNV_mRNA_seq_consensus       TAGTAATTGAATTTTTAACAACACTATATAATGTCATAGACAAAAGAGTTCCGAAATTAA
TRINITY_DN266_c0_g2_i1         TAGTAATTGAATTTTTAACAACACTATATAATGTCATAGACAAAAGAGTTCCGAAATTAA
TRINITY_DN5346_c0_g1_i1        TAGTAATTGAATTTTTAACAACACTATATAATGTCATAGACAAAAGAGTTCCGAAATTAA
TRINITY_DN5346_c0_g1_i2        TAGTAATTGAATTTTTAACAACACTATATAATGTCATAGACAAAAGAGTTCCGAAATTAA
                               ************************************************************

TRINITY_DN7_c0_g2_i1           ATAGTATATGTATAAAAAGTCCCCCTTCGGCAGGTAAGAACTTTTTTTTCGATGCCGTTG
MpDNV_mRNA_seq_consensus       ATAGTATATGTATAAAAAGTCCCCCTTCGGCAGGTAAGAACTTTTTTTTCGATGCCGTTG
TRINITY_DN266_c0_g2_i1         ATAGTATATGTATAAAAAGTCCCCCTTCGGCAGGTAAGAACTTTTTTTTCGATGCCGTTG
TRINITY_DN5346_c0_g1_i1        ATAGTATATGTATAAAAAGTCCCCCTTCGGCAGGTAAGAACTTTTTTTTCGATGCCGTTG
TRINITY_DN5346_c0_g1_i2        ATAGTATATGTATAAAAAGTCCCCCTTCGGCAGGTAAGAACTTTTTTTTCGATGCCGTTG
                               ************************************************************

TRINITY_DN7_c0_g2_i1           CATCGTATTTGCTTTCATATGGCATGTTCGGTACAGCCAATAAAAACAATAATTTTTCGT
MpDNV_mRNA_seq_consensus       CATCGTATTTGCTTTCATATGGTATGTTCGGTACAGCCAATAAAAACAATAATTTTTCGT
TRINITY_DN266_c0_g2_i1 APFV16 CATCGTATTTGCTTTCATATGGTATGTTCGGTACAGCCAATAAAAACAATAATTTTTCGT
TRINITY_DN5346_c0_g1_i1        CATCGTATTTGCTTTCATATGGCATGTTCGGTACAGCCAATAAAAACAATAATTTTTCGT
TRINITY_DN5346_c0_g1_i2        CATCGTATTTGCTTTCATATGGCATGTTCGGTACAGCCAATAAAAACAATAATTTTTCGT
                               ********************** *************************************

TRINITY_DN7_c0_g2_i1           GGGCAGACGGAGCGGGTAAACGATTAGTTCTGTGGAACGAACCAAACTATGAACAATACC
MpDNV_mRNA_seq_consensus       GGGCAGACGGAGCGGGTAAACGATTAGTTCTGTGGAACGAACCAAACTATGAACAATACC
TRINITY_DN266_c0_g2_i1         GGGCAGACGGAGCGGGTAAACGATTAGTTCTGTGGAACGAACCAAACTATGAACAATACC
TRINITY_DN5346_c0_g1_i1        GGGCAGACGGAGCGGGTAAACGATTAGTTCTGTGGAACGAACCAAACTATGAACAATACC
TRINITY_DN5346_c0_g1_i2        GGGCAGACGGAGCGGGTAAACGATTAGTTCTGTGGAACGAACCAAACTATGAACAATACC
                               ************************************************************

TRINITY_DN7_c0_g2_i1           ATATAGAAAAAATAAAAGAACTTTTGGGGGGAGATACAACAAGAATACATGTCAAATATG
MpDNV_mRNA_seq_consensus       ATATAGAAAAAATAAAAGAACTTTTGGGGGGAGATACAACAAGAATACATGTCAAATATG
TRINITY_DN266_c0_g2_i1         ATATAGAAAAAATAAAAGAACTTTTGGGGGGAGATACAACAAGAATACATGTCAAATATG
TRINITY_DN5346_c0_g1_i1        ATATAGAAAAAATAAAAGAACTTTTGGGGGGAGATACAACAAGAATACATGTCAAATATG
TRINITY_DN5346_c0_g1_i2        ATATAGAAAAAATAAAAGAACTTTTGGGGGGAGATACAACAAGAATACATGTCAAATATG
                               ************************************************************

TRINITY_DN7_c0_g2_i1           CAAACGACGTTAGTGTGCAAAGGGTACCCATTATTATATTAACAAATAACCACTTGAATA
MpDNV_mRNA_seq_consensus       CAAACGACGTTAGTGTGCAAAGGGTACCCATTATTATATTAACAAATAACCACTTGAATA
TRINITY_DN266_c0_g2_i1         CAAACGACGTTAGTGTGCAAAGGGTACCCATTATTATATTAACAAATAACCACTTGAATA
TRINITY_DN5346_c0_g1_i1        CAAACGACGTTAGTGTGCAAAGGGTACCCATTATTATATTAACAAATAACCACTTGAATA
TRINITY_DN5346_c0_g1_i2        CAAACGACGTTAGTGTGCAAAGGGTACCCATTATTATATTAACAAATAACCACTTGAATA
```

```
                              ************************************************************

TRINITY_DN7_c0_g2_i1          TTATTAGTCACCCAGCTTTTAACGATAGATTGCGGAGTTACGAATGGATGTCAGCTGGCT
MpDNV_mRNA_seq_consensus      TTATTAGTCACCCAGCTTTTAACGATAGATTGCGGAGTTACGAATGGATGTCAGCTGGCT
TRINITY_DN266_c0_g2_i1        TTATTAGTCACCCAGCTTTTAACGATAGATTGCGGAGTTACGAATGGATGTCAGCTGGCT
TRINITY_DN5346_c0_g1_i1       TTATTAGTCACCCAGCTTTTAACGATAGATTGCGGAGTTACGAATGGATGTCAGCTGGCT
TRINITY_DN5346_c0_g1_i2       TTATTAGTCACCCAGCTTTTAACGATAGATTGCGGAGTTACGAATGGATGTCAGCTGGCT
                              ************************************************************

TRINITY_DN7_c0_g2_i1          TCTTGAAGGACTACGATAAAAAACTGCATCCGCTTATGTTTTATGAATTACTTAAAGATT
MpDNV_mRNA_seq_consensus      TCTTGAAGGACTACGATAAAAAACTGCATCCGCTTATGTTTTATGAATTACTTAAAGATT
TRINITY_DN266_c0_g2_i1        TCTTGAAGGACTACGATAAAAAACTGCATCCGCTTATGTTTTATGAATTACTTAAAGATT
TRINITY_DN5346_c0_g1_i1       TCTTGAAGGACTACGATAAAAAACTGCATCCGCTTATGTTTTATGAATTACTTAAAGATT
TRINITY_DN5346_c0_g1_i2       TCTTGAAGGACTACGATAAAAAACTGCATCCGCTTATGTTTTATGAATTACTTAAAGATT
                              ************************************************************

TRINITY_DN7_c0_g2_i1  APFV14  ATGGTGTAATAAATGATAACAATATGATTGATTAATATATATATATAAAAAAAAAAAAAA
MpDNV_mRNA_seq_consensus      ATGGTGTAATAAATGATAACAATATGATTGATTAATATATATATATATATATTGATTTTT NS poly(A)
TRINITY_DN266_c0_g2_i1 APFV16 ATGGTGTAATAAATGATAACAATATGATTGATTAATATATATATATATATATTGATTTTT
TRINITY_DN5346_c0_g1_i1APFV15 ATGGTGTAATAAATGATAACAATATGATTGATTAATATATATATATAAAAAAAAAA--- tail
TRINITY_DN5346_c0_g1_i2       ATGGTGTAATAAATGATAACAATATGATTGATTAATATATATATATAAAAAAAAAA--- tail
                              *********************************************** * * *

TRINITY_DN7_c0_g2_i1          AATTCAAAAAAAAAAAAAAAAA-------------------------------------- tail
MpDNV_mRNA_seq_consensus      ATTTACGTAAAAAATCTACAGGTTCATTGACAATGGGTATATTCATTGACCTTCGCCACA
TRINITY_DN266_c0_g2_i1        ATTTACGTAAAAAAAAAA------------------------------------------ tail
TRINITY_DN5346_c0_g1_i1       ------------------------------------------------------------
TRINITY_DN5346_c0_g1_i2       ------------------------------------------------------------


TRINITY_DN266_c0_g1_i1APFV-16 -------------TTTTTTTTTTTTTTTGATTAATATATATATATATATATTGATTTTTA tail
TRINITY_DN266_c0_g1_i2        -------------TTTTTTTTTTTTTTTGATTAATATATATATATATATATTGATTTTTA tail
TRINITY_DN266_c0_g1_i4        -------------TTTTTTTTTTTTTTTGATTAATATATATATATATATATTGATTTTTA tail
TRINITY_DN266_c0_g1_i3        -------------TTTTTTTTTTTTTTTGATTAATATATATATATATATATTGATTTTTA tail
MpDNV_mRNA_seq_consensus      TGGTGTAATAAATGATAACAATATGATTGATTAATATATATATATATATATTGATTTTTA VP poly(A)
TRINITY_DN7_c0_g1_i4  APFV-14 ------------TTTTTTTTTTTTTTTTGATTAATATATATATATATATATTGATTTTTA tail
TRINITY_DN7_c0_g1_i2          ------------TTTTTTTTTTTTTTTTGATTAATATATATATATATATATTGATTTTTA tail
TRINITY_DN7_c0_g1_i1          ------------TTTTTTTTTTTTTTTTGATTAATATATATATATATATATTGATTTTTA tail
TRINITY_DN7_c0_g1_i3          ------------TTTTTTTTTTTTTTTTGATTAATATATATATATATATATTGATTTTTA tail
TRINITY_DN18_c0_g1_i1 APFV-15 ------------------TTTTTTTTTTGATTAATATATATATATATATATTGATTTTTA tail
TRINITY_DN18_c0_g1_i2         ------------------TTTTTTTTTTGATTAATATATATATATATATATTGATTTTTA tail
TRINITY_DN18_c0_g1_i4         ------------------TTTTTTTTTTGATTAATATATATATATATATATTGATTTTTA tail
TRINITY_DN18_c0_g1_i3         ------------------TTTTTTTTTTGATTAATATATATATATATATATTGATTTTTA tail
                                                          **********************************

TRINITY_DN266_c0_g1_i1        TTTACGTAAAAAATCTACAGGTTCATTGACAATGGGTATATTCATTGACCTTCGCCACAA
TRINITY_DN266_c0_g1_i2        TTTACGTAAAAAATCTACAGGTTCATTGACAATGGGTATATTCATTGACCTTCGCCACAA
TRINITY_DN266_c0_g1_i4        TTTACGTAAAAAATCTACAGGTTCATTGACAATGGGTATATTCATTGACCTTCGCCACAA
TRINITY_DN266_c0_g1_i3        TTTACGTAAAAAATCTACAGGTTCATTGACAATGGGTATATTCATTGACCTTCGCCACAA
MpDNV_mRNA_seq_consensus      TTTACGTAAAAAATCTACAGGTTCATTGACAATGGGTATATTCATTGACCTTCGCCACAA
TRINITY_DN7_c0_g1_i4          TTTACGTAAAAAATCTACAGGTTCATTGACAATGGGTATATTCATTGACCTTCGCCACAA
TRINITY_DN7_c0_g1_i2          TTTACGTAAAAAATCTACAGGTTCATTGACAATGGGTATATTCATTGACCTTCGCCACAA
TRINITY_DN7_c0_g1_i1          TTTACGTAAAAAATCTACAGGTTCATTGACAATGGGTATATTCATTGACCTTCGCCACAA
TRINITY_DN7_c0_g1_i3          TTTACGTAAAAAATCTACAGGTTCATTGACAATGGGTATATTCATTGACCTTCGCCACAA
TRINITY_DN18_c0_g1_i1         TTTACGTAAAAAATCTACAGGTTCATTGACAATGGGTATATTCATTGACCTTCGCCACAA
TRINITY_DN18_c0_g1_i2         TTTACGTAAAAAATCTACAGGTTCATTGACAATGGGTATATTCATTGACCTTCGCCACAA
TRINITY_DN18_c0_g1_i4         TTTACGTAAAAAATCTACAGGTTCATTGACAATGGGTATATTCATTGACCTTCGCCACAA
TRINITY_DN18_c0_g1_i3         TTTACGTAAAAAATCTACAGGTTCATTGACAATGGGTATATTCATTGACCTTCGCCACAA
                              ************************************************************

TRINITY_DN266_c0_g1_i1        TGTTAAATCACGGCAAAAAGCTAAATACGTTGAATATGACTTGAACAATCGTTCCTCTGG
TRINITY_DN266_c0_g1_i2        TGTTAAATCACGGCAAAAAGCTAAATACGTTGAATATGACTTGAACAATCGTTCCTCTGG
TRINITY_DN266_c0_g1_i4        TGTTAAATCACGGCAAAAAGCTAAATACGTTGAATATGACTTGAACAATCGTTCCTCTGG
TRINITY_DN266_c0_g1_i3        TGTTAAATCACGGCAAAAAGCTAAATACGTTGAATATGACTTGAACAATCGTTCCTCTGG
MpDNV_mRNA_seq_consensus      TGTTAAATCACGGCAAAAAGCTAAATACGTTGAATATGACTTGAACAATCGTTCCTCTGG
TRINITY_DN7_c0_g1_i4          TGTTAAATCACGGCAAAAAGCTAAATACGTTGAATATGACTTGAACAATCGTTCCTCTGG
TRINITY_DN7_c0_g1_i2          TGTTAAATCACGGCAAAAAGCTAAATACGTTGAATATGACTTGAACAATCGTTCCTCTGG
TRINITY_DN7_c0_g1_i1          TGTTAAATCACGGCAAAAAGCTAAATACGTTGAATATGACTTGAACAATCGTTCCTCTGG
TRINITY_DN7_c0_g1_i3          TGTTAAATCACGGCAAAAAGCTAAATACGTTGAATATGACTTGAACAATCGTTCCTCTGG
TRINITY_DN18_c0_g1_i1         TGTTAAATCACGGCAAAAAGCTAAATACGTTGAATATGACTTGAACAATCGTTCCTCTGG
TRINITY_DN18_c0_g1_i2         TGTTAAATCACGGCAAAAAGCTAAATACGTTGAATATGACTTGAACAATCGTTCCTCTGG
TRINITY_DN18_c0_g1_i4         TGTTAAATCACGGCAAAAAGCTAAATACGTTGAATATGACTTGAACAATCGTTCCTCTGG
TRINITY_DN18_c0_g1_i3         TGTTAAATCACGGCAAAAAGCTAAATACGTTGAATATGACTTGAACAATCGTTCCTCTGG
                              ************************************************************

TRINITY_DN266_c0_g1_i1        CCACCAATTCGGATTATTGTAGTCGCCCCAAGAAGTGTTCATGATTTCTTCGGTTGTGGG
TRINITY_DN266_c0_g1_i2        CCACCAATTCGGATTATTGTAGTCGCCCCAAGAAGTGTTCATGATTTCTTCGGTTGTGGG
TRINITY_DN266_c0_g1_i4        CCACCAATTCGGATTATTGTAGTCGCCCCAAGAAGTGTTCATGATTTCTTCGGTTGTGGG
TRINITY_DN266_c0_g1_i3        CCACCAATTCGGATTATTGTAGTCGCCCCAAGAAGTGTTCATGATTTCTTCGGTTGTGGG
MpDNV_mRNA_seq_consensus      CCACCAATTCGGATTATTGTAGTCGCCCCAAGAAGTGTTCATGATTTCTTCGGTTGTGGG
TRINITY_DN7_c0_g1_i4          CCACCAATTCGGATTATTGTAGTCGCCCCAAGAAGTGTTCATGATTTCTTCGGTTGTGGG
TRINITY_DN7_c0_g1_i2          CCACCAATTCGGATTATTGTAGTCGCCCCAAGAAGTGTTCATGATTTCTTCGGTTGTGGG
TRINITY_DN7_c0_g1_i1          CCACCAATTCGGATTATTGTAGTCGCCCCAAGAAGTGTTCATGATTTCTTCGGTTGTGGG
TRINITY_DN7_c0_g1_i3          CCACCAATTCGGATTATTGTAGTCGCCCCAAGAAGTGTTCATGATTTCTTCGGTTGTGGG
TRINITY_DN18_c0_g1_i1         CCACCAATTCGGATTATTGTAGTCGCCCCAAGAAGTGTTCATGATTTCTTCGGTTGTGGG
TRINITY_DN18_c0_g1_i2         CCACCAATTCGGATTATTGTAGTCGCCCCAAGAAGTGTTCATGATTTCTTCGGTTGTGGG
TRINITY_DN18_c0_g1_i4         CCACCAATTCGGATTATTGTAGTCGCCCCAAGAAGTGTTCATGATTTCTTCGGTTGTGGG
TRINITY_DN18_c0_g1_i3         CCACCAATTCGGATTATTGTAGTCGCCCCAAGAAGTGTTCATGATTTCTTCGGTTGTGGG
```

```
                            ************************************************************

TRINITY_DN266_c0_g1_i1      TGGAGTAGTGGTATATCGATGCAAATTAATTAGGGTTTCTTTTGACGCACGATCACTTTC
TRINITY_DN266_c0_g1_i2      TGGAGTAGTGGTATATCGATGCAAATTAATTAGGGTTTCTTTTGACGCACGATCACTTTC
TRINITY_DN266_c0_g1_i4      TGGAGTAGTGGTATATCGATGCAAATTAATTAGGGTTTCTTTTGACGCACGATCACTTTC
TRINITY_DN266_c0_g1_i3      TGGAGTAGTGGTATATCGATGCAAATTAATTAGGGTTTCTTTTGACGCACGATCACTTTC
MpDNV_mRNA_seq_consensus    TGGAGTAGTGGTATATCGATGCAAATTAATTAGGGTTTCTTTTGACGCACGATCACTTTC
TRINITY_DN7_c0_g1_i4        TGGAGTAGTGGTATATCGATGCAAATTAATTAGGGTTTCTTTTGACGCACGATCACTTTC
TRINITY_DN7_c0_g1_i2        TGGAGTAGTGGTATATCGATGCAAATTAATTAGGGTTTCTTTTGACGCACGATCACTTTC
TRINITY_DN7_c0_g1_i1        TGGAGTAGTGGTATATCGATGCAAATTAATTAGGGTTTCTTTTGACGCACGATCACTTTC
TRINITY_DN7_c0_g1_i3        TGGAGTAGTGGTATATCGATGCAAATTAATTAGGGTTTCTTTTGACGCACGATCACTTTC
TRINITY_DN18_c0_g1_i1       TGGAGTAGTGGTATATCGATGCAAATTAATTAGGGTTTCTTTTGACGCACGATCACTTTC
TRINITY_DN18_c0_g1_i2       TGGAGTAGTGGTATATCGATGCAAATTAATTAGGGTTTCTTTTGACGCACGATCACTTTC
TRINITY_DN18_c0_g1_i4       TGGAGTAGTGGTATATCGATGCAAATTAATTAGGGTTTCTTTTGACGCACGATCACTTTC
TRINITY_DN18_c0_g1_i3       TGGAGTAGTGGTATATCGATGCAAATTAATTAGGGTTTCTTTTGACGCACGATCACTTTC
                            ************************************************************

TRINITY_DN266_c0_g1_i1      TCTGTAAACATAATTGGGTATTTTAATAGGTGCTTTGACTCGAACCTCATGGATGTTTGA VP unspliced
TRINITY_DN266_c0_g1_i2      TCTGTAAACATAATTGGGTATTTTAATAGGTGCTTTGACTCGAACCTCATGGATGTTTGA VP unspliced
TRINITY_DN266_c0_g1_i4      T----------------------------------------------------------- VP spliced 2
TRINITY_DN266_c0_g1_i3      T----------------------------------------------------------- VP spliced 2
MpDNV_mRNA_seq_consensus    TCTGTAAACATAATTGGGTATTTTAATAGGTGCTTTGACTCGAACCTCATGGATGTTTGA 3’splice site
TRINITY_DN7_c0_g1_i4        TCTGTAAACATAATTGGGTATTTTAATAGGTGCTTTGACTCGAACCTCATGGATGTTTGA VP unspliced
TRINITY_DN7_c0_g1_i2        TCTGTAAACATAATTGGGTATTTTAATAGGTGCTTTGACTCGAACCTCATGGATGTTTGA VP unspliced
TRINITY_DN7_c0_g1_i1        T----------------------------------------------------------- VP spliced 2
TRINITY_DN7_c0_g1_i3        T----------------------------------------------------------- VP spliced 2
TRINITY_DN18_c0_g1_i1       TCTGTAAACATAATTGGGTATTTTAATAGGTGCTTTGACTCGAACCTCATGGATGTTTGA VP unspliced
TRINITY_DN18_c0_g1_i2       TCTGTAAACATAATTGGGTATTTTAATAGGTGCTTTGACTCGAACCTCATGGATGTTTGA VP unspliced
TRINITY_DN18_c0_g1_i4       T----------------------------------------------------------- VP spliced 2
TRINITY_DN18_c0_g1_i3       T----------------------------------------------------------- VP spliced 2
                            *

TRINITY_DN266_c0_g1_i1      CGCTTCAACCTCCATGGGGTCGTCGTCCATATTTACCCGAACAGACGTAGGTACAGGTGT VP unspliced
TRINITY_DN266_c0_g1_i2      CGCTTCAACCTCCATGGGGTCGTCGTCCATATTTACCCGAACAGACGTAGGTACAGGTGT VP unspliced
TRINITY_DN266_c0_g1_i4      ------------------------------------CCGAACAGACGTAGGTACAGGTGT VP spliced 2
TRINITY_DN266_c0_g1_i3      ------------------------------------CCGAACAGACGTAGGTACAGGTGT VP spliced 2
MpDNV_mRNA_seq_consensus    CGCTTCAACCTCCATGGGGTCGTCGTCCATATTTACCCGAACAGACGTAGGTACAGGTGT 5’splice site
TRINITY_DN7_c0_g1_i4        CGCTTCAACCTCCATGGGGTCGTCGTCCATATTTACCCGAACAGACGTAGGTACAGGTGT VP unspliced
TRINITY_DN7_c0_g1_i2        CGCTTCAACCTCCATGGGGTCGTCGTCCATATTTACCCGAACAGACGTAGGTACAGGTGT VP unspliced
TRINITY_DN7_c0_g1_i1        ------------------------------------CCGAACAGACGTAGGTACAGGTGT VP spliced 2
TRINITY_DN7_c0_g1_i3        ------------------------------------CCGAACAGACGTAGGTACAGGTGT VP spliced 2
TRINITY_DN18_c0_g1_i1       CGCTTCAACCTCCATGGGGTCGTCGTCCATATTTACCCGAACAGACGTAGGTACAGGTGT VP unspliced
TRINITY_DN18_c0_g1_i2       CGCTTCAACCTCCATGGGGTCGTCGTCCATATTTACCCGAACAGACGTAGGTACAGGTGT VP unspliced
TRINITY_DN18_c0_g1_i4       ------------------------------------CCGAACAGACGTAGGTACAGGTGT
TRINITY_DN18_c0_g1_i3       ------------------------------------CCGAACAGACGTAGGTACAGGTGT
                                                                ************************

TRINITY_DN266_c0_g1_i1      AGTGACAGTTGCGTCGACTGGAGTAGTAAATGAATCAATGTTCTTTGTAGTGGAAGCTTT
TRINITY_DN266_c0_g1_i2      AGTGACAGTTGCGTCGACTGGAGTAGTAAATGAATCAATGTTCTTTGTAGTGGAAGCTTT
TRINITY_DN266_c0_g1_i4      AGTGACAGTTGCGTCGACTGGAGTAGTAAATGAATCAATGTTCTTTGTAGTGGAAGCTTT
TRINITY_DN266_c0_g1_i3      AGTGACAGTTGCGTCGACTGGAGTAGTAAATGAATCAATGTTCTTTGTAGTGGAAGCTTT
MpDNV_mRNA_seq_consensus    AGTGACAGTTGCGTCGACTGGAGTAGTAAATGAATCAATGTTCTTTGTAGTGGAAGCTTT
TRINITY_DN7_c0_g1_i4        AGTGACAGTTGCGTCGACTGGAGTAGTAAATGAATCAATGTTCTTTGTAGTGGAAGCTTT
TRINITY_DN7_c0_g1_i2        AGTGACAGTTGCGTCGACTGGAGTAGTAAATGAATCAATGTTCTTTGTAGTGGAAGCTTT
TRINITY_DN7_c0_g1_i1        AGTGACAGTTGCGTCGACTGGAGTAGTAAATGAATCAATGTTCTTTGTAGTGGAAGCTTT
TRINITY_DN7_c0_g1_i3        AGTGACAGTTGCGTCGACTGGAGTAGTAAATGAATCAATGTTCTTTGTAGTGGAAGCTTT
TRINITY_DN18_c0_g1_i1       AGTGACAGTTGCGTCGACTGGAGTAGTAAATGAATCAATGTTCTTTGTAGTGGAAGCTTT
TRINITY_DN18_c0_g1_i2       AGTGACAGTTGCGTCGACTGGAGTAGTAAATGAATCAATGTTCTTTGTAGTGGAAGCTTT
TRINITY_DN18_c0_g1_i4       AGTGACAGTTGCGTCGACTGGAGTAGTAAATGAATCAATGTTCTTTGTAGTGGAAGCTTT
TRINITY_DN18_c0_g1_i3       AGTGACAGTTGCGTCGACTGGAGTAGTAAATGAATCAATGTTCTTTGTAGTGGAAGCTTT
                            ************************************************************

TRINITY_DN266_c0_g1_i1      GCCCATTTCTTTAGTTAGTCCCGAAGAGTGTGCAGCGGCGTTTTGATTGTCAGCTACTTT
TRINITY_DN266_c0_g1_i2      GCCCATTTCTTTAGTTAGTCCCGAAGAGTGTGCAGCGGCGTTTTGATTGTCAGCTACTTT
TRINITY_DN266_c0_g1_i4      GCCCATTTCTTTAGTTAGTCCCGAAGAGTGTGCAGCGGCGTTTTGATTGTCAGCTACTTT
TRINITY_DN266_c0_g1_i3      GCCCATTTCTTTAGTTAGTCCCGAAGAGTGTGCAGCGGCGTTTTGATTGTCAGCTACTTT
MpDNV_mRNA_seq_consensus    GCCCATTTCTTTAGTTAGTCCCGAAGAGTGTGCAGCGGCGTTTTGATTGTCAGCTACTTT
TRINITY_DN7_c0_g1_i4        GCCCATTTCTTTAGTTAGTCCCGAAGAGTGTGCAGCGGCGTTTTGATTGTCAGCTACTTT
TRINITY_DN7_c0_g1_i2        GCCCATTTCTTTAGTTAGTCCCGAAGAGTGTGCAGCGGCGTTTTGATTGTCAGCTACTTT
TRINITY_DN7_c0_g1_i1        GCCCATTTCTTTAGTTAGTCCCGAAGAGTGTGCAGCGGCGTTTTGATTGTCAGCTACTTT
TRINITY_DN7_c0_g1_i3        GCCCATTTCTTTAGTTAGTCCCGAAGAGTGTGCAGCGGCGTTTTGATTGTCAGCTACTTT
TRINITY_DN18_c0_g1_i1       GCCCATTTCTTTAGTTAGTCCCGAAGAGTGTGCAGCGGCGTTTTGATTGTCAGCTACTTT
TRINITY_DN18_c0_g1_i2       GCCCATTTCTTTAGTTAGTCCCGAAGAGTGTGCAGCGGCGTTTTGATTGTCAGCTACTTT
TRINITY_DN18_c0_g1_i4       GCCCATTTCTTTAGTTAGTCCCGAAGAGTGTGCAGCGGCGTTTTGATTGTCAGCTACTTT
TRINITY_DN18_c0_g1_i3       GCCCATTTCTTTAGTTAGTCCCGAAGAGTGTGCAGCGGCGTTTTGATTGTCAGCTACTTT
                            ************************************************************

TRINITY_DN266_c0_g1_i1      TTCAGGAGCCACGTACAATCCGTGAACAATAGATAAATCTCCACGATTGTATAGATTTTG
TRINITY_DN266_c0_g1_i2      TTCAGGAGCCACGTACAATCCGTGAACAATAGATAAATCTCCACGATTGTATAGATTTTG
TRINITY_DN266_c0_g1_i4      TTCAGGAGCCACGTACAATCCGTGAACAATAGATAAATCTCCACGATTGTATAGATTTTG
TRINITY_DN266_c0_g1_i3      TTCAGGAGCCACGTACAATCCGTGAACAATAGATAAATCTCCACGATTGTATAGATTTTG
MpDNV_mRNA_seq_consensus    TTCAGGAGCCACGTACAATCCGTGAACAATAGATAAATCTCCACGATTGTATAGATTTTG
TRINITY_DN7_c0_g1_i4        TTCAGGAGCCACGTACAATCCGTGAACAATAGATAAATCTCCACGATTGTATAGATTTTG
TRINITY_DN7_c0_g1_i2        TTCAGGAGCCACGTACAATCCGTGAACAATAGATAAATCTCCACGATTGTATAGATTTTG
TRINITY_DN7_c0_g1_i1        TTCAGGAGCCACGTACAATCCGTGAACAATAGATAAATCTCCACGATTGTATAGATTTTG
TRINITY_DN7_c0_g1_i3        TTCAGGAGCCACGTACAATCCGTGAACAATAGATAAATCTCCACGATTGTATAGATTTTG
TRINITY_DN18_c0_g1_i1       TTCAGGAGCCACGTACAATCCGTGAACAATAGATAAATCTCCACGATTGTATAGATTTTG
TRINITY_DN18_c0_g1_i2       TTCAGGAGCCACGTACAATCCGTGAACAATAGATAAATCTCCACGATTGTATAGATTTTG
```

```
TRINITY_DN18_c0_g1_i4        TTCAGGAGCCACGTACAATCCGTGAACAATAGATAAATCTCCACGATTGTATAGATTTTG
TRINITY_DN18_c0_g1_i3        TTCAGGAGCCACGTACAATCCGTGAACAATAGATAAATCTCCACGATTGTATAGATTTTG
                             ************************************************************

TRINITY_DN266_c0_g1_i1       ATTGTAATTGTCGTCGGCCAGCAACCATGTCTTTTCGTATTCCACGTGAAGCCTGTCAAA
TRINITY_DN266_c0_g1_i2       ATTGTAATTGTCGTCGGCCAGCAACCATGTCTTTTCGTATTCCACGTGAAGCCTGTCAAA
TRINITY_DN266_c0_g1_i4       ATTGTAATTGTCGTCGGCCAGCAACCATGTCTTTTCGTATTCCACGTGAAGCCTGTCAAA
TRINITY_DN266_c0_g1_i3       ATTGTAATTGTCGTCGGCCAGCAACCATGTCTTTTCGTATTCCACGTGAAGCCTGTCAAA
MpDNV_mRNA_seq_consensus     ATTGTAATTGTCGTCGGCCAGCAACCATGTCTTTTCGTATTCCACGTGAAGCCTGTCAAA
TRINITY_DN7_c0_g1_i4         ATTGTAATTGTCGTCGGCCAGCAACCATGTCTTTTCGTATTCCACGTGAAGCCTGTCAAA
TRINITY_DN7_c0_g1_i2         ATTGTAATTGTCGTCGGCCAGCAACCATGTCTTTTCGTATTCCACGTGAAGCCTGTCAAA
TRINITY_DN7_c0_g1_i1         ATTGTAATTGTCGTCGGCCAGCAACCATGTCTTTTCGTATTCCACGTGAAGCCTGTCAAA
TRINITY_DN7_c0_g1_i3         ATTGTAATTGTCGTCGGCCAGCAACCATGTCTTTTCGTATTCCACGTGAAGCCTGTCAAA
TRINITY_DN18_c0_g1_i1        ATTGTAATTGTCGTCGGCCAGCAACCATGTCTTTTCGTATTCCACGTGAAGCCTGTCAAA
TRINITY_DN18_c0_g1_i2        ATTGTAATTGTCGTCGGCCAGCAACCATGTCTTTTCGTATTCCACGTGAAGCCTGTCAAA
TRINITY_DN18_c0_g1_i4        ATTGTAATTGTCGTCGGCCAGCAACCATGTCTTTTCGTATTCCACGTGAAGCCTGTCAAA
TRINITY_DN18_c0_g1_i3        ATTGTAATTGTCGTCGGCCAGCAACCATGTCTTTTCGTATTCCACGTGAAGCCTGTCAAA
                             ************************************************************

TRINITY_DN266_c0_g1_i1       CGCTGTTCTGCTTTGCGGACATCCAAAACTAACTGTCATCTCAGTTTCGAAATCCCATGT
TRINITY_DN266_c0_g1_i2       CGCTGTTCTGCTTTGCGGACATCCAAAACTAACTGTCATCTCAGTTTCGAAATCCCATGT
TRINITY_DN266_c0_g1_i4       CGCTGTTCTGCTTTGCGGACATCCAAAACTAACTGTCATCTCAGTTTCGAAATCCCATGT
TRINITY_DN266_c0_g1_i3       CGCTGTTCTGCTTTGCGGACATCCAAAACTAACTGTCATCTCAGTTTCGAAATCCCATGT
MpDNV_mRNA_seq_consensus     CGCTGTTCTGCTTTGCGGACATCCAAAACTAACTGTCATCTCAGTTTCGAAATCCCATGT
TRINITY_DN7_c0_g1_i4         CGCTGTTCTGCTTTGCGGACATCCAAAACTAACTGTCATCTCAGTTTCGAAATCCCATGT
TRINITY_DN7_c0_g1_i2         CGCTGTTCTGCTTTGCGGACATCCAAAACTAACTGTCATCTCAGTTTCGAAATCCCATGT
TRINITY_DN7_c0_g1_i1         CGCTGTTCTGCTTTGCGGACATCCAAAACTAACTGTCATCTCAGTTTCGAAATCCCATGT
TRINITY_DN7_c0_g1_i3         CGCTGTTCTGCTTTGCGGACATCCAAAACTAACTGTCATCTCAGTTTCGAAATCCCATGT
TRINITY_DN18_c0_g1_i1        CGCTGTTCTGCTTTGCGGACATCCAAAACTAACTGTCATCTCAGTTTCGAAATCCCATGT
TRINITY_DN18_c0_g1_i2        CGCTGTTCTGCTTTGCGGACATCCAAAACTAACTGTCATCTCAGTTTCGAAATCCCATGT
TRINITY_DN18_c0_g1_i4        CGCTGTTCTGCTTTGCGGACATCCAAAACTAACTGTCATCTCAGTTTCGAAATCCCATGT
TRINITY_DN18_c0_g1_i3        CGCTGTTCTGCTTTGCGGACATCCAAAACTAACTGTCATCTCAGTTTCGAAATCCCATGT
                             ************************************************************

TRINITY_DN266_c0_g1_i1       GCATTCTACGTCAGTAAAATTTACAGGGACTATACTATTAGTGGCAGTAGTTAACTTATG
TRINITY_DN266_c0_g1_i2       GCATTCTACGTCAGTAAAATTTACAGGGACTATACTATTAGTGGCAGTAGTTAACTTATG
TRINITY_DN266_c0_g1_i4       GCATTCTACGTCAGTAAAATTTACAGGGACTATACTATTAGTGGCAGTAGTTAACTTATG
TRINITY_DN266_c0_g1_i3       GCATTCTACGTCAGTAAAATTTACAGGGACTATACTATTAGTGGCAGTAGTTAACTTATG
MpDNV_mRNA_seq_consensus     GCATTCTACGTCAGTAAAATTTACAGGGACTATACTATTAGTGGCAGTAGTTAACTTATG
TRINITY_DN7_c0_g1_i4         GCATTCTACGTCAGTAAAATTTACAGGGACTATACTATTAGTGGCAGTAGTTAACTTATG
TRINITY_DN7_c0_g1_i2         GCATTCTACGTCAGTAAAATTTACAGGGACTATACTATTAGTGGCAGTAGTTAACTTATG
TRINITY_DN7_c0_g1_i1         GCATTCTACGTCAGTAAAATTTACAGGGACTATACTATTAGTGGCAGTAGTTAACTTATG
TRINITY_DN7_c0_g1_i3         GCATTCTACGTCAGTAAAATTTACAGGGACTATACTATTAGTGGCAGTAGTTAACTTATG
TRINITY_DN18_c0_g1_i1        GCATTCTACGTCAGTAAAATTTACAGGGACTATACTATTAGTGGCAGTAGTTAACTTATG
TRINITY_DN18_c0_g1_i2        GCATTCTACGTCAGTAAAATTTACAGGGACTATACTATTAGTGGCAGTAGTTAACTTATG
TRINITY_DN18_c0_g1_i4        GCATTCTACGTCAGTAAAATTTACAGGGACTATACTATTAGTGGCAGTAGTTAACTTATG
TRINITY_DN18_c0_g1_i3        GCATTCTACGTCAGTAAAATTTACAGGGACTATACTATTAGTGGCAGTAGTTAACTTATG
                             ************************************************************

TRINITY_DN266_c0_g1_i1       CACTGGATATATTCCTACATGTACACTAGGTTGTACTTTATTAGAATATTCATGTTTATC
TRINITY_DN266_c0_g1_i2       CACTGGATATATTCCTACATGTACACTAGGTTGTACTTTATTAGAATATTCATGTTTATC
TRINITY_DN266_c0_g1_i4       CACTGGATATATTCCTACATGTACACTAGGTTGTACTTTATTAGAATATTCATGTTTATC
TRINITY_DN266_c0_g1_i3       CACTGGATATATTCCTACATGTACACTAGGTTGTACTTTATTAGAATATTCATGTTTATC
MpDNV_mRNA_seq_consensus     CACTGGATATATTCCTACATGTACACTAGGTTGTACTTTATTAGAATATTCATGTTTATC
TRINITY_DN7_c0_g1_i4         CACTGGATATATTCCTACATGTACACTAGGTTGTACTTTATTAGAATATTCATGTTTATC
TRINITY_DN7_c0_g1_i2         CACTGGATATATTCCTACATGTACACTAGGTTGTACTTTATTAGAATATTCATGTTTATC
TRINITY_DN7_c0_g1_i1         CACTGGATATATTCCTACATGTACACTAGGTTGTACTTTATTAGAATATTCATGTTTATC
TRINITY_DN7_c0_g1_i3         CACTGGATATATTCCTACATGTACACTAGGTTGTACTTTATTAGAATATTCATGTTTATC
TRINITY_DN18_c0_g1_i1        CACTGGATATATTCCTACATGTACACTAGGTTGTACTTTATTAGAATATTCATGTTTATC
TRINITY_DN18_c0_g1_i2        CACTGGATATATTCCTACATGTACACTAGGTTGTACTTTATTAGAATATTCATGTTTATC
TRINITY_DN18_c0_g1_i4        CACTGGATATATTCCTACATGTACACTAGGTTGTACTTTATTAGAATATTCATGTTTATC
TRINITY_DN18_c0_g1_i3        CACTGGATATATTCCTACATGTACACTAGGTTGTACTTTATTAGAATATTCATGTTTATC
                             ************************************************************

TRINITY_DN266_c0_g1_i1       GAACATACGAAAATATTGGCCTACGTCTATGTTAGTACAATATCTATCATTGCCGAATAT
TRINITY_DN266_c0_g1_i2       GAACATACGAAAATATTGGCCTACGTCTATGTTAGTACAATATCTATCATTGCCGAATAT
TRINITY_DN266_c0_g1_i4       GAACATACGAAAATATTGGCCTACGTCTATGTTAGTACAATATCTATCATTGCCGAATAT
TRINITY_DN266_c0_g1_i3       GAACATACGAAAATATTGGCCTACGTCTATGTTAGTACAATATCTATCATTGCCGAATAT
MpDNV_mRNA_seq_consensus     GAACATACGAAAATATTGGCCTACGTCTATGTTAGTACAATATCTATCATTGCCGAATAT
TRINITY_DN7_c0_g1_i4         GAACATACGAAAATATTGGCCTACGTCTATGTTAGTACAATATCTATCATTGCCGAATAT
TRINITY_DN7_c0_g1_i2         GAACATACGAAAATATTGGCCTACGTCTATGTTAGTACAATATCTATCATTGCCGAATAT
TRINITY_DN7_c0_g1_i1         GAACATACGAAAATATTGGCCTACGTCTATGTTAGTACAATATCTATCATTGCCGAATAT
TRINITY_DN7_c0_g1_i3         GAACATACGAAAATATTGGCCTACGTCTATGTTAGTACAATATCTATCATTGCCGAATAT
TRINITY_DN18_c0_g1_i1        GAACATACGAAAATATTGGCCTACGTCTATGTTAGTACAATATCTATCATTGCCGAATAT
TRINITY_DN18_c0_g1_i2        GAACATACGAAAATATTGGCCTACGTCTATGTTAGTACAATATCTATCATTGCCGAATAT
TRINITY_DN18_c0_g1_i4        GAACATACGAAAATATTGGCCTACGTCTATGTTAGTACAATATCTATCATTGCCGAATAT
TRINITY_DN18_c0_g1_i3        GAACATACGAAAATATTGGCCTACGTCTATGTTAGTACAATATCTATCATTGCCGAATAT
                             ************************************************************

TRINITY_DN266_c0_g1_i1       GCGTTTCCAACCGTTTTCACCCACAAATGTCAATTTTTGATCCGATCTCCTTTCGAACAA
TRINITY_DN266_c0_g1_i2       GCGTTTCCAACCGTTTTCACCCACAAATGTCAATTTTTGATCCGATCTCCTTTCGAACAA
TRINITY_DN266_c0_g1_i4       GCGTTTCCAACCGTTTTCACCCACAAATGTCAATTTTTGATCCGATCTCCTTTCGAACAA
TRINITY_DN266_c0_g1_i3       GCGTTTCCAACCGTTTTCACCCACAAATGTCAATTTTTGATCCGATCTCCTTTCGAACAA
MpDNV_mRNA_seq_consensus     GCGTTTCCAACCGTTTTCACCCACAAATGTCAATTTTTGATCCGATCTCCTTTCGAACAA
TRINITY_DN7_c0_g1_i4         GCGTTTCCAACCGTTTTCACCCACAAATGTCAATTTTTGATCCGATCTCCTTTCGAACAA
TRINITY_DN7_c0_g1_i2         GCGTTTCCAACCGTTTTCACCCACAAATGTCAATTTTTGATCCGATCTCCTTTCGAACAA
TRINITY_DN7_c0_g1_i1         GCGTTTCCAACCGTTTTCACCCACAAATGTCAATTTTTGATCCGATCTCCTTTCGAACAA
TRINITY_DN7_c0_g1_i3         GCGTTTCCAACCGTTTTCACCCACAAATGTCAATTTTTGATCCGATCTCCTTTCGAACAA
```

```
TRINITY_DN18_c0_g1_i1         GCGTTTCCAACCGTTTTCACCCACAAATGTCAATTTTTGATCCGATCTCCTTTCGAACAA
TRINITY_DN18_c0_g1_i2         GCGTTTCCAACCGTTTTCACCCACAAATGTCAATTTTTGATCCGATCTCCTTTCGAACAA
TRINITY_DN18_c0_g1_i4         GCGTTTCCAACCGTTTTCACCCACAAATGTCAATTTTTGATCCGATCTCCTTTCGAACAA
TRINITY_DN18_c0_g1_i3         GCGTTTCCAACCGTTTTCACCCACAAATGTCAATTTTTGATCCGATCTCCTTTCGAACAA
                              ************************************************************

TRINITY_DN266_c0_g1_i1        GCCACTGGCAGCGTTTATAAAATCTTGACACTCTGGTTCGTTACCAGCATTGCGTAACAT
TRINITY_DN266_c0_g1_i2        GCCACTGGCAGCGTTTATAAAATCTTGACACTCTGGTTCGTTACCAGCATTGCGTAACAT
TRINITY_DN266_c0_g1_i4        GCCACTGGCAGCGTTTATAAAATCTTGACACTCTGGTTCGTTACCAGCATTGCGTAACAT
TRINITY_DN266_c0_g1_i3        GCCACTGGCAGCGTTTATAAAATCTTGACACTCTGGTTCGTTACCAGCATTGCGTAACAT
MpDNV_mRNA_seq_consensus      GCCACTGGCAGCGTTTATAAAATCTTGACACTCTGGTTCGTTACCAGCATTGCGTAACAT
TRINITY_DN7_c0_g1_i4          GCCACTGGCAGCGTTTATAAAATCTTGACACTCTGGTTCGTTACCAGCATTGCGTAACAT
TRINITY_DN7_c0_g1_i2          GCCACTGGCAGCGTTTATAAAATCTTGACACTCTGGTTCGTTACCAGCATTGCGTAACAT
TRINITY_DN7_c0_g1_i1          GCCACTGGCAGCGTTTATAAAATCTTGACACTCTGGTTCGTTACCAGCATTGCGTAACAT
TRINITY_DN7_c0_g1_i3          GCCACTGGCAGCGTTTATAAAATCTTGACACTCTGGTTCGTTACCAGCATTGCGTAACAT
TRINITY_DN18_c0_g1_i1         GCCACTGGCAGCGTTTATAAAATCTTGACACTCTGGTTCGTTACCAGCATTGCGTAACAT
TRINITY_DN18_c0_g1_i2         GCCACTGGCAGCGTTTATAAAATCTTGACACTCTGGTTCGTTACCAGCATTGCGTAACAT
TRINITY_DN18_c0_g1_i4         GCCACTGGCAGCGTTTATAAAATCTTGACACTCTGGTTCGTTACCAGCATTGCGTAACAT
TRINITY_DN18_c0_g1_i3         GCCACTGGCAGCGTTTATAAAATCTTGACACTCTGGTTCGTTACCAGCATTGCGTAACAT
                              ************************************************************

TRINITY_DN266_c0_g1_i1        AGCAAATTTACTGGAATTAGGCTCCTGTACGCTGACACACGCATCGAATATTGGATCGTG
TRINITY_DN266_c0_g1_i2        AGCAAATTTACTGGAATTAGGCTCCTGTACGCTGACACACGCATCGAATATTGGATCGTG
TRINITY_DN266_c0_g1_i4        AGCAAATTTACTGGAATTAGGCTCCTGTACGCTGACACACGCATCGAATATTGGATCGTG
TRINITY_DN266_c0_g1_i3        AGCAAATTTACTGGAATTAGGCTCCTGTACGCTGACACACGCATCGAATATTGGATCGTG
MpDNV_mRNA_seq_consensus      AGCAAATTTACTGGAATTAGGCTCCTGTACGCTGACACACGCATCGAATATTGGATCGTG
TRINITY_DN7_c0_g1_i4          AGCAAATTTACTGGAATTAGGCTCCTGTACGCTGACACACGCATCGAATATTGGATCGTG
TRINITY_DN7_c0_g1_i2          AGCAAATTTACTGGAATTAGGCTCCTGTACGCTGACACACGCATCGAATATTGGATCGTG
TRINITY_DN7_c0_g1_i1          AGCAAATTTACTGGAATTAGGCTCCTGTACGCTGACACACGCATCGAATATTGGATCGTG
TRINITY_DN7_c0_g1_i3          AGCAAATTTACTGGAATTAGGCTCCTGTACGCTGACACACGCATCGAATATTGGATCGTG
TRINITY_DN18_c0_g1_i1         AGCAAATTTACTGGAATTAGGCTCCTGTACGCTGACACACGCATCGAATATTGGATCGTG
TRINITY_DN18_c0_g1_i2         AGCAAATTTACTGGAATTAGGCTCCTGTACGCTGACACACGCATCGAATATTGGATCGTG
TRINITY_DN18_c0_g1_i4         AGCAAATTTACTGGAATTAGGCTCCTGTACGCTGACACACGCATCGAATATTGGATCGTG
TRINITY_DN18_c0_g1_i3         AGCAAATTTACTGGAATTAGGCTCCTGTACGCTGACACACGCATCGAATATTGGATCGTG
                              ************************************************************

TRINITY_DN266_c0_g1_i1        TGGTAGAGTGAGTATACCGACATGAGGCTTGTACGAATAATTAAGTACAGTGGTGCCGAC
TRINITY_DN266_c0_g1_i2        TGGTAGAGTGAGTATACCGACATGAGGCTTGTACGAATAATTAAGTACAGTGGTGCCGAC
TRINITY_DN266_c0_g1_i4        TGGTAGAGTGAGTATACCGACATGAGGCTTGTACGAATAATTAAGTACAGTGGTGCCGAC
TRINITY_DN266_c0_g1_i3        TGGTAGAGTGAGTATACCGACATGAGGCTTGTACGAATAATTAAGTACAGTGGTGCCGAC
MpDNV_mRNA_seq_consensus      TGGTAGAGTGAGTATACCGACATGAGGCTTGTACGAATAATTAAGTACAGTGGTGCCGAC
TRINITY_DN7_c0_g1_i4          TGGTAGAGTGAGTATACCGACATGAGGCTTGTACGAATAATTAAGTACAGTGGTGCCGAC
TRINITY_DN7_c0_g1_i2          TGGTAGAGTGAGTATACCGACATGAGGCTTGTACGAATAATTAAGTACAGTGGTGCCGAC
TRINITY_DN7_c0_g1_i1          TGGTAGAGTGAGTATACCGACATGAGGCTTGTACGAATAATTAAGTACAGTGGTGCCGAC
TRINITY_DN7_c0_g1_i3          TGGTAGAGTGAGTATACCGACATGAGGCTTGTACGAATAATTAAGTACAGTGGTGCCGAC
TRINITY_DN18_c0_g1_i1         TGGTAGAGTGAGTATACCGACATGAGGCTTGTACGAATAATTAAGTACAGTGGTGCCGAC
TRINITY_DN18_c0_g1_i2         TGGTAGAGTGAGTATACCGACATGAGGCTTGTACGAATAATTAAGTACAGTGGTGCCGAC
TRINITY_DN18_c0_g1_i4         TGGTAGAGTGAGTATACCGACATGAGGCTTGTACGAATAATTAAGTACAGTGGTGCCGAC
TRINITY_DN18_c0_g1_i3         TGGTAGAGTGAGTATACCGACATGAGGCTTGTACGAATAATTAAGTACAGTGGTGCCGAC
                              ************************************************************

TRINITY_DN266_c0_g1_i1        AGTAAACGAAGCGTCGACCTTGGTGATGTGTTCATTAAACCGTTCCCATCCGAGTTTTCC
TRINITY_DN266_c0_g1_i2        AGTAAACGAAGCGTCGACCTTGGTGATGTGTTCATTAAACCGTTCCCATCCGAGTTTTCC
TRINITY_DN266_c0_g1_i4        AGTAAACGAAGCGTCGACCTTGGTGATGTGTTCATTAAACCGTTCCCATCCGAGTTTTCC
TRINITY_DN266_c0_g1_i3        AGTAAACGAAGCGTCGACCTTGGTGATGTGTTCATTAAACCGTTCCCATCCGAGTTTTCC
MpDNV_mRNA_seq_consensus      AGTAAACGAAGCGTCGACCTTGGTGATGTGTTCATTAAACCGTTCCCATCCGAGTTTTCC
TRINITY_DN7_c0_g1_i4          AGTAAACGAAGCGTCGACCTTGGTGATGTGTTCATTAAACCGTTCCCATCCGAGTTTTCC
TRINITY_DN7_c0_g1_i2          AGTAAACGAAGCGTCGACCTTGGTGATGTGTTCATTAAACCGTTCCCATCCGAGTTTTCC
TRINITY_DN7_c0_g1_i1          AGTAAACGAAGCGTCGACCTTGGTGATGTGTTCATTAAACCGTTCCCATCCGAGTTTTCC
TRINITY_DN7_c0_g1_i3          AGTAAACGAAGCGTCGACCTTGGTGATGTGTTCATTAAACCGTTCCCATCCGAGTTTTCC
TRINITY_DN18_c0_g1_i1         AGTAAACGAAGCGTCGACCTTGGTGATGTGTTCATTAAACCGTTCCCATCCGAGTTTTCC
TRINITY_DN18_c0_g1_i2         AGTAAACGAAGCGTCGACCTTGGTGATGTGTTCATTAAACCGTTCCCATCCGAGTTTTCC
TRINITY_DN18_c0_g1_i4         AGTAAACGAAGCGTCGACCTTGGTGATGTGTTCATTAAACCGTTCCCATCCGAGTTTTCC
TRINITY_DN18_c0_g1_i3         AGTAAACGAAGCGTCGACCTTGGTGATGTGTTCATTAAACCGTTCCCATCCGAGTTTTCC
                              ************************************************************

TRINITY_DN266_c0_g1_i1        AGAATTATGATTTCTATTTACAAAATTACAAAAATAACTGTTATACATCAACGGTAAGTT
TRINITY_DN266_c0_g1_i2        AGAATTATGATTTCTATTTACAAAATTACAAAAATAACTGTTATACATCAACGGTAAGTT
TRINITY_DN266_c0_g1_i4        AGAATTATGATTTCTATTTACAAAATTACAAAAATAACTGTTATACATCAACGGTAAGTT
TRINITY_DN266_c0_g1_i3        AGAATTATGATTTCTATTTACAAAATTACAAAAATAACTGTTATACATCAACGGTAAGTT
MpDNV_mRNA_seq_consensus      AGAATTATGATTTCTATTTACAAAATTACAAAAATAACTGTTATACATCAACGGTAAGTT
TRINITY_DN7_c0_g1_i4          AGAATTATGATTTCTATTTACAAAATTACAAAAATAACTGTTATACATCAACGGTAAGTT
TRINITY_DN7_c0_g1_i2          AGAATTATGATTTCTATTTACAAAATTACAAAAATAACTGTTATACATCAACGGTAAGTT
TRINITY_DN7_c0_g1_i1          AGAATTATGATTTCTATTTACAAAATTACAAAAATAACTGTTATACATCAACGGTAAGTT
TRINITY_DN7_c0_g1_i3          AGAATTATGATTTCTATTTACAAAATTACAAAAATAACTGTTATACATCAACGGTAAGTT
TRINITY_DN18_c0_g1_i1         AGAATTATGATTTCTATTTACAAAATTACAAAAATAACTGTTATACATCAACGGTAAGTT
TRINITY_DN18_c0_g1_i2         AGAATTATGATTTCTATTTACAAAATTACAAAAATAACTGTTATACATCAACGGTAAGTT
TRINITY_DN18_c0_g1_i4         AGAATTATGATTTCTATTTACAAAATTACAAAAATAACTGTTATACATCAACGGTAAGTT
TRINITY_DN18_c0_g1_i3         AGAATTATGATTTCTATTTACAAAATTACAAAAATAACTGTTATACATCAACGGTAAGTT
                              ************************************************************

TRINITY_DN266_c0_g1_i1        CATGTAGGACGCAGGCAGTTCATATCCCTCTTTAGCAGAGAATGTACCATCTAGATCTTG
TRINITY_DN266_c0_g1_i2        CATGTAGGACGCAGGCAGTTCATATCCCTCTTTAGCAGAGAATGTACCATCTAGATCTTG
TRINITY_DN266_c0_g1_i4        CATGTAGGACGCAGGCAGTTCATATCCCTCTTTAGCAGAGAATGTACCATCTAGATCTTG
TRINITY_DN266_c0_g1_i3        CATGTAGGACGCAGGCAGTTCATATCCCTCTTTAGCAGAGAATGTACCATCTAGATCTTG
MpDNV_mRNA_seq_consensus      CATGTAGGACGCAGGCAGTTCATATCCCTCTTTAGCAGAGAATGTACCATCTAGATCTTG
TRINITY_DN7_c0_g1_i4          CATGTAGGACGCAGGCAGTTCATATCCCTCTTTAGCAGAGAATGTACCATCTAGATCTTG
TRINITY_DN7_c0_g1_i2          CATGTAGGACGCAGGCAGTTCATATCCCTCTTTAGCAGAGAATGTACCATCTAGATCTTG
```

```
TRINITY_DN7_c0_g1_i1          CATGTAGGACGCAGGCAGTTCATATCCCTCTTTAGCAGAGAATGTACCATCTAGATCTTG
TRINITY_DN7_c0_g1_i3          CATGTAGGACGCAGGCAGTTCATATCCCTCTTTAGCAGAGAATGTACCATCTAGATCTTG
TRINITY_DN18_c0_g1_i1         CATGTAGGACGCAGGCAGTTCATATCCCTCTTTAGCAGAGAATGTACCATCTAGATCTTG
TRINITY_DN18_c0_g1_i2         CATGTAGGACGCAGGCAGTTCATATCCCTCTTTAGCAGAGAATGTACCATCTAGATCTTG
TRINITY_DN18_c0_g1_i4         CATGTAGGACGCAGGCAGTTCATATCCCTCTTTAGCAGAGAATGTACCATCTAGATCTTG
TRINITY_DN18_c0_g1_i3         CATGTAGGACGCAGGCAGTTCATATCCCTCTTTAGCAGAGAATGTACCATCTAGATCTTG
                              ************************************************************

TRINITY_DN266_c0_g1_i1        ACCGTACAATTTGCGAACGAATTCTTTCATAGACGACTGATCGTTCTCGGTATTCGTTGG
TRINITY_DN266_c0_g1_i2        ACCGTACAATTTGCGAACGAATTCTTTCATAGACGACTGATCGTTCTCGGTATTCGTTGG
TRINITY_DN266_c0_g1_i4        ACCGTACAATTTGCGAACGAATTCTTTCATAGACGACTGATCGTTCTCGGTATTCGTTGG
TRINITY_DN266_c0_g1_i3        ACCGTACAATTTGCGAACGAATTCTTTCATAGACGACTGATCGTTCTCGGTATTCGTTGG
MpDNV_mRNA_seq_consensus      ACCGTACAATTTGCGAACGAATTCTTTCATAGACGACTGATCGTTCTCGGTATTCGTTGG
TRINITY_DN7_c0_g1_i4          ACCGTACAATTTGCGAACGAATTCTTTCATAGACGACTGATCGTTCTCGGTATTCGTTGG
TRINITY_DN7_c0_g1_i2          ACCGTACAATTTGCGAACGAATTCTTTCATAGACGACTGATCGTTCTCGGTATTCGTTGG
TRINITY_DN7_c0_g1_i1          ACCGTACAATTTGCGAACGAATTCTTTCATAGACGACTGATCGTTCTCGGTATTCGTTGG
TRINITY_DN7_c0_g1_i3          ACCGTACAATTTGCGAACGAATTCTTTCATAGACGACTGATCGTTCTCGGTATTCGTTGG
TRINITY_DN18_c0_g1_i1         ACCGTACAATTTGCGAACGAATTCTTTCATAGACGACTGATCGTTCTCGGTATTCGTTGG
TRINITY_DN18_c0_g1_i2         ACCGTACAATTTGCGAACGAATTCTTTCATAGACGACTGATCGTTCTCGGTATTCGTTGG
TRINITY_DN18_c0_g1_i4         ACCGTACAATTTGCGAACGAATTCTTTCATAGACGACTGATCGTTCTCGGTATTCGTTGG
TRINITY_DN18_c0_g1_i3         ACCGTACAATTTGCGAACGAATTCTTTCATAGACGACTGATCGTTCTCGGTATTCGTTGG
                              ************************************************************

TRINITY_DN266_c0_g1_i1        ATCCATGGGCTCCGATGAATTGCCAAATGTGAGTTTTCGGTTAAATCCCCTTGTTTTTAA
TRINITY_DN266_c0_g1_i2        ATCCATGGGCTCCGATGAATTGCCAAATGTGAGTTTTCGGTTAAATCCCCTTGTTTTTAA
TRINITY_DN266_c0_g1_i4        ATCCATGGGCTCCGATGAATTGCCAAATGTGAGTTTTCGGTTAAATCCCCTTGTTTTTAA
TRINITY_DN266_c0_g1_i3        ATCCATGGGCTCCGATGAATTGCCAAATGTGAGTTTTCGGTTAAATCCCCTTGTTTTTAA
MpDNV_mRNA_seq_consensus      ATCCATGGGCTCCGATGAATTGCCAAATGTGAGTTTTCGGTTAAATCCCCTTGTTTTTAA
TRINITY_DN7_c0_g1_i4          ATCCATGGGCTCCGATGAATTGCCAAATGTGAGTTTTCGGTTAAATCCCCTTGTTTTTAA
TRINITY_DN7_c0_g1_i2          ATCCATGGGCTCCGATGAATTGCCAAATGTGAGTTTTCGGTTAAATCCCCTTGTTTTTAA
TRINITY_DN7_c0_g1_i1          ATCCATGGGCTCCGATGAATTGCCAAATGTGAGTTTTCGGTTAAATCCCCTTGTTTTTAA
TRINITY_DN7_c0_g1_i3          ATCCATGGGCTCCGATGAATTGCCAAATGTGAGTTTTCGGTTAAATCCCCTTGTTTTTAA
TRINITY_DN18_c0_g1_i1         ATCCATGGGCTCCGATGAATTGCCAAATGTGAGTTTTCGGTTAAATCCCCTTGTTTTTAA
TRINITY_DN18_c0_g1_i2         ATCCATGGGCTCCGATGAATTGCCAAATGTGAGTTTTCGGTTAAATCCCCTTGTTTTTAA
TRINITY_DN18_c0_g1_i4         ATCCATGGGCTCCGATGAATTGCCAAATGTGAGTTTTCGGTTAAATCCCCTTGTTTTTAA
TRINITY_DN18_c0_g1_i3         ATCCATGGGCTCCGATGAATTGCCAAATGTGAGTTTTCGGTTAAATCCCCTTGTTTTTAA
                              ************************************************************

TRINITY_DN266_c0_g1_i1        ATTCAAAGCGTCCGCTTTTACCATAAATTTGTTCTGATTCAATGTGGCCAGGGAGCTCGT
TRINITY_DN266_c0_g1_i2        ATTCAAAGCGTCCGCTTTTACCATAAATTTGTTCTGATTCAATGTGGCCAGGGAGCTCGT
TRINITY_DN266_c0_g1_i4        ATTCAAAGCGTCCGCTTTTACCATAAATTTGTTCTGATTCAATGTGGCCAGGGAGCTCGT
TRINITY_DN266_c0_g1_i3        ATTCAAAGCGTCCGCTTTTACCATAAATTTGTTCTGATTCAATGTGGCCAGGGAGCTCGT
MpDNV_mRNA_seq_consensus      ATTCAAAGCGTCCGCTTTTACCATAAATTTGTTCTGATTCAATGTGGCCAGGGAGCTCGT
TRINITY_DN7_c0_g1_i4          ATTCAAAGCGTCCGCTTTTACCATAAATTTGTTCTGATTCAATGTGGCCAGGGAGCTCGT
TRINITY_DN7_c0_g1_i2          ATTCAAAGCGTCCGCTTTTACCATAAATTTGTTCTGATTCAATGTGGCCAGGGAGCTCGT
TRINITY_DN7_c0_g1_i1          ATTCAAAGCGTCCGCTTTTACCATAAATTTGTTCTGATTCAATGTGGCCAGGGAGCTCGT
TRINITY_DN7_c0_g1_i3          ATTCAAAGCGTCCGCTTTTACCATAAATTTGTTCTGATTCAATGTGGCCAGGGAGCTCGT
TRINITY_DN18_c0_g1_i1         ATTCAAAGCGTCCGCTTTTACCATAAATTTGTTCTGATTCAATGTGGCCAGGGAGCTCGT
TRINITY_DN18_c0_g1_i2         ATTCAAAGCGTCCGCTTTTACCATAAATTTGTTCTGATTCAATGTGGCCAGGGAGCTCGT
TRINITY_DN18_c0_g1_i4         ATTCAAAGCGTCCGCTTTTACCATAAATTTGTTCTGATTCAATGTGGCCAGGGAGCTCGT
TRINITY_DN18_c0_g1_i3         ATTCAAAGCGTCCGCTTTTACCATAAATTTGTTCTGATTCAATGTGGCCAGGGAGCTCGT
                              ************************************************************

TRINITY_DN266_c0_g1_i1        AGAGCTATTGGTTTCAAAAGCTGTGCGAGGATTACGCATGACACCCTTGCATTTGACTTC
TRINITY_DN266_c0_g1_i2        AGAGCTATTGGTTTCAAAAGCTGTGCGAGGATTACGCATGACACCCTTGCATTTGACTTC
TRINITY_DN266_c0_g1_i4        AGAGCTATTGGTTTCAAAAGCTGTGCGAGGATTACGCATGACACCCTTGCATTTGACTTC
TRINITY_DN266_c0_g1_i3        AGAGCTATTGGTTTCAAAAGCTGTGCGAGGATTACGCATGACACCCTTGCATTTGACTTC
MpDNV_mRNA_seq_consensus      AGAGCTATTGGTTTCAAAAGCTGTGCGAGGATTACGCATGACACCCTTGCATTTGACTTC
TRINITY_DN7_c0_g1_i4          AGAGCTATTGGTTTCAAAAGCTGTGCGAGGATTACGCATGACACCCTTGCATTTGACTTC
TRINITY_DN7_c0_g1_i2          AGAGCTATTGGTTTCAAAAGCTGTGCGAGGATTACGCATGACACCCTTGCATTTGACTTC
TRINITY_DN7_c0_g1_i1          AGAGCTATTGGTTTCAAAAGCTGTGCGAGGATTACGCATGACACCCTTGCATTTGACTTC
TRINITY_DN7_c0_g1_i3          AGAGCTATTGGTTTCAAAAGCTGTGCGAGGATTACGCATGACACCCTTGCATTTGACTTC
TRINITY_DN18_c0_g1_i1         AGAGCTATTGGTTTCAAAAGCTGTGCGAGGATTACGCATGACACCCTTGCATTTGACTTC
TRINITY_DN18_c0_g1_i2         AGAGCTATTGGTTTCAAAAGCTGTGCGAGGATTACGCATGACACCCTTGCATTTGACTTC
TRINITY_DN18_c0_g1_i4         AGAGCTATTGGTTTCAAAAGCTGTGCGAGGATTACGCATGACACCCTTGCATTTGACTTC
TRINITY_DN18_c0_g1_i3         AGAGCTATTGGTTTCAAAAGCTGTGCGAGGATTACGCATGACACCCTTGCATTTGACTTC
                              ************************************************************

TRINITY_DN266_c0_g1_i1        AGTGCATATAGCACCTCTGGGTAAAAATTTAAACTCGGACGGCGACATATAGAACCAAGG
TRINITY_DN266_c0_g1_i2        AGTGCATATAGCACCTCTGGGTAAAAATTTAAACTCGGACGGCGACATATAGAACCAAGG
TRINITY_DN266_c0_g1_i4        AGTGCATATAGCACCTCTGGGTAAAAATTTAAACTCGGACGGCGACATATAGAACCAAGG
TRINITY_DN266_c0_g1_i3        AGTGCATATAGCACCTCTGGGTAAAAATTTAAACTCGGACGGCGACATATAGAACCAAGG
MpDNV_mRNA_seq_consensus      AGTGCATATAGCACCTCTGGGTAAAAATTTAAACTCGGACGGCGACATATAGAACCAAGG
TRINITY_DN7_c0_g1_i4          AGTGCATATAGCACCTCTGGGTAAAAATTTAAACTCGGACGGCGACATATAGAACCAAGG
TRINITY_DN7_c0_g1_i2          AGTGCATATAGCACCTCTGGGTAAAAATTTAAACTCGGACGGCGACATATAGAACCAAGG
TRINITY_DN7_c0_g1_i1          AGTGCATATAGCACCTCTGGGTAAAAATTTAAACTCGGACGGCGACATATAGAACCAAGG
TRINITY_DN7_c0_g1_i3          AGTGCATATAGCACCTCTGGGTAAAAATTTAAACTCGGACGGCGACATATAGAACCAAGG
TRINITY_DN18_c0_g1_i1         AGTGCATATAGCACCTCTGGGTAAAAATTTAAACTCGGACGGCGACATATAGAACCAAGG
TRINITY_DN18_c0_g1_i2         AGTGCATATAGCACCTCTGGGTAAAAATTTAAACTCGGACGGCGACATATAGAACCAAGG
TRINITY_DN18_c0_g1_i4         AGTGCATATAGCACCTCTGGGTAAAAATTTAAACTCGGACGGCGACATATAGAACCAAGG
TRINITY_DN18_c0_g1_i3         AGTGCATATAGCACCTCTGGGTAAAAATTTAAACTCGGACGGCGACATATAGAACCAAGG
                              ************************************************************

TRINITY_DN266_c0_g1_i1        TTGGTCCACGGGCACACTCATTAAACTCGTTGTCCCAAGATAGGCGTTATCAGTGCTAAG
TRINITY_DN266_c0_g1_i2        TTGGTCCACGGGCACACTCATTAAACTCGTTGTCCCAAGATAGGCGTTATCAGTGCTAAG
TRINITY_DN266_c0_g1_i4        TTGGTCCACGGGCACACTCATTAAACTCGTTGTCCCAAGATAGGCGTTATCAGTGCTAAG
TRINITY_DN266_c0_g1_i3        TTGGTCCACGGGCACACTCATTAAACTCGTTGTCCCAAGATAGGCGTTATCAGTGCTAAG
MpDNV_mRNA_seq_consensus      TTGGTCCACGGGCACACTCATTAAACTCGTTGTCCCAAGATAGGCGTTATCAGTGCTAAG
```

```
TRINITY_DN7_c0_g1_i4          TTGGTCCACGGGCACACTCATTAAACTCGTTGTCCCAAGATAGGCGTTATCAGTGCTAAG
TRINITY_DN7_c0_g1_i2          TTGGTCCACGGGCACACTCATTAAACTCGTTGTCCCAAGATAGGCGTTATCAGTGCTAAG
TRINITY_DN7_c0_g1_i1          TTGGTCCACGGGCACACTCATTAAACTCGTTGTCCCAAGATAGGCGTTATCAGTGCTAAG
TRINITY_DN7_c0_g1_i3          TTGGTCCACGGGCACACTCATTAAACTCGTTGTCCCAAGATAGGCGTTATCAGTGCTAAG
TRINITY_DN18_c0_g1_i1         TTGGTCCACGGGCACACTCATTAAACTCGTTGTCCCAAGATAGGCGTTATCAGTGCTAAG
TRINITY_DN18_c0_g1_i2         TTGGTCCACGGGCACACTCATTAAACTCGTTGTCCCAAGATAGGCGTTATCAGTGCTAAG
TRINITY_DN18_c0_g1_i4         TTGGTCCACGGGCACACTCATTAAACTCGTTGTCCCAAGATAGGCGTTATCAGTGCTAAG
TRINITY_DN18_c0_g1_i3         TTGGTCCACGGGCACACTCATTAAACTCGTTGTCCCAAGATAGGCGTTATCAGTGCTAAG
                              ************************************************************

TRINITY_DN266_c0_g1_i1        ATATCGCAACACTTTCCAACCACATCCGTAACTGATGAAAGTATGTACTTTTCGGTAAAC
TRINITY_DN266_c0_g1_i2        ATATCGCAACACTTTCCAACCACATCCGTAACTGATGAAAGTATGTACTTTTCGGTAAAC
TRINITY_DN266_c0_g1_i4        ATATCGCAACACTTTCCAACCACATCCGTAACTGATGAAAGTATGTACTTTTCGGTAAAC
TRINITY_DN266_c0_g1_i3        ATATCGCAACACTTTCCAACCACATCCGTAACTGATGAAAGTATGTACTTTTCGGTAAAC
MpDNV_mRNA_seq_consensus      ATATCGCAACACTTTCCAACCACATCCGTAACTGATGAAAGTATGTACTTTTCGGTAAAC
TRINITY_DN7_c0_g1_i4          ATATCGCAACACTTTCCAACCACATCCGTAACTGATGAAAGTATGTACTTTTCGGTAAAC
TRINITY_DN7_c0_g1_i2          ATATCGCAACACTTTCCAACCACATCCGTAACTGATGAAAGTATGTACTTTTCGGTAAAC
TRINITY_DN7_c0_g1_i1          ATATCGCAACACTTTCCAACCACATCCGTAACTGATGAAAGTATGTACTTTTCGGTAAAC
TRINITY_DN7_c0_g1_i3          ATATCGCAACACTTTCCAACCACATCCGTAACTGATGAAAGTATGTACTTTTCGGTAAAC
TRINITY_DN18_c0_g1_i1         ATATCGCAACACTTTCCAACCACATCCGTAACTGATGAAAGTATGTACTTTTCGGTAAAC
TRINITY_DN18_c0_g1_i2         ATATCGCAACACTTTCCAACCACATCCGTAACTGATGAAAGTATGTACTTTTCGGTAAAC
TRINITY_DN18_c0_g1_i4         ATATCGCAACACTTTCCAACCACATCCGTAACTGATGAAAGTATGTACTTTTCGGTAAAC
TRINITY_DN18_c0_g1_i3         ATATCGCAACACTTTCCAACCACATCCGTAACTGATGAAAGTATGTACTTTTCGGTAAAC
                              ************************************************************

TRINITY_DN266_c0_g1_i1        CATAGTATATCCTCCCGTGTGACCGATTGGCCTAGGTATAACAGCGTTTTCAGAGCTAGG
TRINITY_DN266_c0_g1_i2        CATAGTATATCCTCCCGTGTGACCGATTGGCCTAGGTATAACAGCGTTTTCAGAGCTAGG
TRINITY_DN266_c0_g1_i4        CATAGTATATCCTCCCGTGTGACCGATTGGCCTAGGTATAACAGCGTTTTCAGAGCTAGG
TRINITY_DN266_c0_g1_i3        CATAGTATATCCTCCCGTGTGACCGATTGGCCTAGGTATAACAGCGTTTTCAGAGCTAGG
MpDNV_mRNA_seq_consensus      CATAGTATATCCTCCCGTGTGACCGATTGGCCTAGGTATAACAGCGTTTTCAGAGCTAGG
TRINITY_DN7_c0_g1_i4          CATAGTATATCCTCCCGTGTGACCGATTGGCCTAGGTATAACAGCGTTTTCAGAGCTAGG
TRINITY_DN7_c0_g1_i2          CATAGTATATCCTCCCGTGTGACCGATTGGCCTAGGTATAACAGCGTTTTCAGAGCTAGG
TRINITY_DN7_c0_g1_i1          CATAGTATATCCTCCCGTGTGACCGATTGGCCTAGGTATAACAGCGTTTTCAGAGCTAGG
TRINITY_DN7_c0_g1_i3          CATAGTATATCCTCCCGTGTGACCGATTGGCCTAGGTATAACAGCGTTTTCAGAGCTAGG
TRINITY_DN18_c0_g1_i1         CATAGTATATCCTCCCGTGTGACCGATTGGCCTAGGTATAACAGCGTTTTCAGAGCTAGG
TRINITY_DN18_c0_g1_i2         CATAGTATATCCTCCCGTGTGACCGATTGGCCTAGGTATAACAGCGTTTTCAGAGCTAGG
TRINITY_DN18_c0_g1_i4         CATAGTATATCCTCCCGTGTGACCGATTGGCCTAGGTATAACAGCGTTTTCAGAGCTAGG
TRINITY_DN18_c0_g1_i3         CATAGTATATCCTCCCGTGTGACCGATTGGCCTAGGTATAACAGCGTTTTCAGAGCTAGG
                              ************************************************************

TRINITY_DN266_c0_g1_i1        ATTGCCAGTATCTGGGTCCCCATCGTTCGCAGCAGCCGTCCCAGGAAGCGAGGTGCCACC
TRINITY_DN266_c0_g1_i2        ATTGCCAGTATCTGGGTCCCCATCGTTCGCAGCAGCCGTCCCAGGAAGCGAGGTGCCACC
TRINITY_DN266_c0_g1_i4        ATTGCCAGTATCTGGGTCCCCATCGTTCGCAGCAGCCGTCCCAGGAAGCGAGGTGCCACC
TRINITY_DN266_c0_g1_i3        ATTGCCAGTATCTGGGTCCCCATCGTTCGCAGCAGCCGTCCCAGGAAGCGAGGTGCCACC
MpDNV_mRNA_seq_consensus      ATTGCCAGTATCTGGGTCCCCATCGTTCGCAGCAGCCGTCCCAGGAAGCGAGGTGCCACC
TRINITY_DN7_c0_g1_i4          ATTGCCAGTATCTGGGTCCCCATCGTTCGCAGCAGCCGTCCCAGGAAGCGAGGTGCCACC
TRINITY_DN7_c0_g1_i2          ATTGCCAGTATCTGGGTCCCCATCGTTCGCAGCAGCCGTCCCAGGAAGCGAGGTGCCACC
TRINITY_DN7_c0_g1_i1          ATTGCCAGTATCTGGGTCCCCATCGTTCGCAGCAGCCGTCCCAGGAAGCGAGGTGCCACC
TRINITY_DN7_c0_g1_i3          ATTGCCAGTATCTGGGTCCCCATCGTTCGCAGCAGCCGTCCCAGGAAGCGAGGTGCCACC
TRINITY_DN18_c0_g1_i1         ATTGCCAGTATCTGGGTCCCCATCGTTCGCAGCAGCCGTCCCAGGAAGCGAGGTGCCACC
TRINITY_DN18_c0_g1_i2         ATTGCCAGTATCTGGGTCCCCATCGTTCGCAGCAGCCGTCCCAGGAAGCGAGGTGCCACC
TRINITY_DN18_c0_g1_i4         ATTGCCAGTATCTGGGTCCCCATCGTTCGCAGCAGCCGTCCCAGGAAGCGAGGTGCCACC
TRINITY_DN18_c0_g1_i3         ATTGCCAGTATCTGGGTCCCCATCGTTCGCAGCAGCCGTCCCAGGAAGCGAGGTGCCACC
                              ************************************************************

TRINITY_DN266_c0_g1_i1        CGCACGTTTAGGAGCGGGTTCAGAACCGGAGCTATCGGCAGGTCGTTTGCTACTTGATCC
TRINITY_DN266_c0_g1_i2        CGCACGTTTAGGAGCGGGTTCAGAACCGGAGCTATCGGCAGGTCGTTTGCTACTTGATCC
TRINITY_DN266_c0_g1_i4        CGCACGTTTAGGAGCGGGTTCAGAACCGGAGCTATCGGCAGGTCGTTTGCTACTTGATCC
TRINITY_DN266_c0_g1_i3        CGCACGTTTAGGAGCGGGTTCAGAACCGGAGCTATCGGCAGGTCGTTTGCTACTTGATCC
MpDNV_mRNA_seq_consensus      CGCACGTTTAGGAGCGGGTTCAGAACCGGAGCTATCGGCAGGTCGTTTGCTACTTGATCC
TRINITY_DN7_c0_g1_i4          CGCACGTTTAGGAGCGGGTTCAGAACCGGAGCTATCGGCAGGTCGTTTGCTACTTGATCC
TRINITY_DN7_c0_g1_i2          CGCACGTTTAGGAGCGGGTTCAGAACCGGAGCTATCGGCAGGTCGTTTGCTACTTGATCC
TRINITY_DN7_c0_g1_i1          CGCACGTTTAGGAGCGGGTTCAGAACCGGAGCTATCGGCAGGTCGTTTGCTACTTGATCC
TRINITY_DN7_c0_g1_i3          CGCACGTTTAGGAGCGGGTTCAGAACCGGAGCTATCGGCAGGTCGTTTGCTACTTGATCC
TRINITY_DN18_c0_g1_i1         CGCACGTTTAGGAGCGGGTTCAGAACCGGAGCTATCGGCAGGTCGTTTGCTACTTGATCC
TRINITY_DN18_c0_g1_i2         CGCACGTTTAGGAGCGGGTTCAGAACCGGAGCTATCGGCAGGTCGTTTGCTACTTGATCC
TRINITY_DN18_c0_g1_i4         CGCACGTTTAGGAGCGGGTTCAGAACCGGAGCTATCGGCAGGTCGTTTGCTACTTGATCC
TRINITY_DN18_c0_g1_i3         CGCACGTTTAGGAGCGGGTTCAGAACCGGAGCTATCGGCAGGTCGTTTGCTACTTGATCC
                              ************************************************************

TRINITY_DN266_c0_g1_i1        GCTAGAGCTTGCCATGGATCCCGATGCGCTTCCCCCGTTGCCGTTGCTAGTGCTGTTGTC
TRINITY_DN266_c0_g1_i2        GCTAGAGCTTGCCATGGATCCCGATGCGCTTCCCCCGTTGCCGTTGCTAGTGCTGTTGTC
TRINITY_DN266_c0_g1_i4        GCTAGAGCTTGCCATGGATCCCGATGCGCTTCCCCCGTTGCCGTTGCTAGTGCTGTTGTC
TRINITY_DN266_c0_g1_i3        GCTAGAGCTTGCCATGGATCCCGATGCGCTTCCCCCGTTGCCGTTGCTAGTGCTGTTGTC
MpDNV_mRNA_seq_consensus      GCTAGAGCTTGCCATGGATCCCGATGCGCTTCCCCCGTTGCCGTTGCTAGTGCTGTTGTC
TRINITY_DN7_c0_g1_i4          GCTAGAGCTTGCCATGGATCCCGATGCGCTTCCCCCGTTGCCGTTGCTAGTGCTGTTGTC
TRINITY_DN7_c0_g1_i2          GCTAGAGCTTGCCATGGATCCCGATGCGCTTCCCCCGTTGCCGTTGCTAGTGCTGTTGTC
TRINITY_DN7_c0_g1_i1          GCTAGAGCTTGCCATGGATCCCGATGCGCTTCCCCCGTTGCCGTTGCTAGTGCTGTTGTC
TRINITY_DN7_c0_g1_i3          GCTAGAGCTTGCCATGGATCCCGATGCGCTTCCCCCGTTGCCGTTGCTAGTGCTGTTGTC
TRINITY_DN18_c0_g1_i1         GCTAGAGCTTGCCATGGATCCCGATGCGCTTCCCCCGTTGCCGTTGCTAGTGCTGTTGTC
TRINITY_DN18_c0_g1_i2         GCTAGAGCTTGCCATGGATCCCGATGCGCTTCCCCCGTTGCCGTTGCTAGTGCTGTTGTC
TRINITY_DN18_c0_g1_i4         GCTAGAGCTTGCCATGGATCCCGATGCGCTTCCCCCGTTGCCGTTGCTAGTGCTGTTGTC
TRINITY_DN18_c0_g1_i3         GCTAGAGCTTGCCATGGATCCCGATGCGCTTCCCCCGTTGCCGTTGCTAGTGCTGTTGTC
                              ************************************************************

TRINITY_DN266_c0_g1_i1        CGGGGCTGAGGTGTTTGCCACGTGGTCGCCACTCGCTTCACCGCTGTTAGGATTGCCGTT
TRINITY_DN266_c0_g1_i2        CGGGGCTGAGGTGTTTGCCACGTGGTCGCCACTCGCTTCACCGCTGTTAGGATTGCCGTT
TRINITY_DN266_c0_g1_i4        CGGGGCTGAGGTGTTTGCCACGTGGTCGCCACTCGCTTCACCGCTGTTAGGATTGCCGTT
```

```
TRINITY_DN266_c0_g1_i3        CGGGGCTGAGGTGTTTGCCACGTGGTCGCCACTCGCTTCACCGCTGTTAGGATTGCCGTT
MpDNV_mRNA_seq_consensus      CGGGGCTGAGGTGTTTGCCACGTGGTCGCCACTCGCTTCACCGCTGTTAGGATTGCCGTT
TRINITY_DN7_c0_g1_i4          CGGGGCTGAGGTGTTTGCCACGTGGTCGCCACTCGCTTCACCGCTGTTAGGATTGCCGTT
TRINITY_DN7_c0_g1_i2          CGGGGCTGAGGTGTTTGCCACGTGGTCGCCACTCGCTTCACCGCTGTTAGGATTGCCGTT
TRINITY_DN7_c0_g1_i1          CGGGGCTGAGGTGTTTGCCACGTGGTCGCCACTCGCTTCACCGCTGTTAGGATTGCCGTT
TRINITY_DN7_c0_g1_i3          CGGGGCTGAGGTGTTTGCCACGTGGTCGCCACTCGCTTCACCGCTGTTAGGATTGCCGTT
TRINITY_DN18_c0_g1_i1         CGGGGCTGAGGTGTTTGCCACGTGGTCGCCACTCGCTTCACCGCTGTTAGGATTGCCGTT
TRINITY_DN18_c0_g1_i2         CGGGGCTGAGGTGTTTGCCACGTGGTCGCCACTCGCTTCACCGCTGTTAGGATTGCCGTT
TRINITY_DN18_c0_g1_i4         CGGGGCTGAGGTGTTTGCCACGTGGTCGCCACTCGCTTCACCGCTGTTAGGATTGCCGTT
TRINITY_DN18_c0_g1_i3         CGGGGCTGAGGTGTTTGCCACGTGGTCGCCACTCGCTTCACCGCTGTTAGGATTGCCGTT
                              ************************************************************

TRINITY_DN266_c0_g1_i1        ACCGTCGTAGTCCCAATCCAAATCAGGATCCTCCTCCACGCCAC---------------- VP spliced 1
TRINITY_DN266_c0_g1_i2        ACCGTCGTAGTCCCAATCCAAATCAGGATCCTCCTCCACGCCACCTAAAACATACGGTGG VP unspliced
TRINITY_DN266_c0_g1_i4        ACCGTCGTAGTCCCAATCCAAATCAGGATCCTCCTCCACGCCACCTAAAACATACGGTGG VP unspliced
TRINITY_DN266_c0_g1_i3        ACCGTCGTAGTCCCAATCCAAATCAGGATCCTCCTCCACGCCAC---------------- VP spliced 1
MpDNV_mRNA_seq_consensus      ACCGTCGTAGTCCCAATCCAAATCAGGATCCTCCTCCACGCCACCTAAAACATACGGTGG 3'splice site
TRINITY_DN7_c0_g1_i4          ACCGTCGTAGTCCCAATCCAAATCAGGATCCTCCTCCACGCCACCTAAAACATACGGTGG VP unspliced
TRINITY_DN7_c0_g1_i2          ACCGTCGTAGTCCCAATCCAAATCAGGATCCTCCTCCACGCCAC---------------- VP spliced 1
TRINITY_DN7_c0_g1_i1          ACCGTCGTAGTCCCAATCCAAATCAGGATCCTCCTCCACGCCACCTAAAACATACGGTGG VP unspliced
TRINITY_DN7_c0_g1_i3          ACCGTCGTAGTCCCAATCCAAATCAGGATCCTCCTCCACGCCAC---------------- VP spliced 1
TRINITY_DN18_c0_g1_i1         ACCGTCGTAGTCCCAATCCAAATCAGGATCCTCCTCCACGCCAC---------------- VP spliced 1
TRINITY_DN18_c0_g1_i2         ACCGTCGTAGTCCCAATCCAAATCAGGATCCTCCTCCACGCCACCTAAAACATACGGTGG VP unspliced
TRINITY_DN18_c0_g1_i4         ACCGTCGTAGTCCCAATCCAAATCAGGATCCTCCTCCACGCCAC---------------- VP spliced 1
TRINITY_DN18_c0_g1_i3         ACCGTCGTAGTCCCAATCCAAATCAGGATCCTCCTCCACGCCACCTAAAACATACGGTGG VP unspliced
                              ********************************************

TRINITY_DN266_c0_g1_i1        ------------------------------------------------------------
TRINITY_DN266_c0_g1_i2        TAATGAACGACGTGCTAAAGCCTTATTGTATTGTTCTAGCGCGTAACGCTGCTGCGATGA
TRINITY_DN266_c0_g1_i4        TAATGAACGACGTGCTAAAGCCTTATTGTATTGTTCTAGCGCGTAACGCTGCTGCGATGA
TRINITY_DN266_c0_g1_i3        ------------------------------------------------------------
MpDNV_mRNA_seq_consensus      TAATGAACGACGTGCTAAAGCCTTATTGTATTGTTCTAGCGCGTAACGCTGCTGCGATGA
TRINITY_DN7_c0_g1_i4          TAATGAACGACGTGCTAAAGCCTTATTGTATTGTTCTAGCGCGTAACGCTGCTGCGATGA
TRINITY_DN7_c0_g1_i2          ------------------------------------------------------------
TRINITY_DN7_c0_g1_i1          TAATGAACGACGTGCTAAAGCCTTATTGTATTGTTCTAGCGCGTAACGCTGCTGCGATGA
TRINITY_DN7_c0_g1_i3          ------------------------------------------------------------
TRINITY_DN18_c0_g1_i1         ------------------------------------------------------------
TRINITY_DN18_c0_g1_i2         TAATGAACGACGTGCTAAAGCCTTATTGTATTGTTCTAGCGCGTAACGCTGCTGCGATGA
TRINITY_DN18_c0_g1_i4         ------------------------------------------------------------
TRINITY_DN18_c0_g1_i3         TAATGAACGACGTGCTAAAGCCTTATTGTATTGTTCTAGCGCGTAACGCTGCTGCGATGA


TRINITY_DN266_c0_g1_i1        ------------------------------------------------------------
TRINITY_DN266_c0_g1_i2        ATTTAAATTTTGCCAATTGGCCCGCTCCGGTGGAGGAGGACCTAAATAACGAAATTCTCG
TRINITY_DN266_c0_g1_i4        ATTTAAATTTTGCCAATTGGCCCGCTCCGGTGGAGGAGGACCTAAATAACGAAATTCTCG
TRINITY_DN266_c0_g1_i3        ------------------------------------------------------------
MpDNV_mRNA_seq_consensus      ATTTAAATTTTGCCAATTGGCCCGCTCCGGTGGAGGAGGACCTAAATAACGAAATTCTCG
TRINITY_DN7_c0_g1_i4          ATTTAAATTTTGCCAATTGGCCCGCTCCGGTGGAGGAGGACCTAAATAACGAAATTCTCG
TRINITY_DN7_c0_g1_i2          ------------------------------------------------------------
TRINITY_DN7_c0_g1_i1          ATTTAAATTTTGCCAATTGGCCCGCTCCGGTGGAGGAGGACCTAAATAACGAAATTCTCG
TRINITY_DN7_c0_g1_i3          ------------------------------------------------------------
TRINITY_DN18_c0_g1_i1         ------------------------------------------------------------
TRINITY_DN18_c0_g1_i2         ATTTAAATTTTGCCAATTGGCCCGCTCCGGTGGAGGAGGACCTAAATAACGAAATTCTCG
TRINITY_DN18_c0_g1_i4         ------------------------------------------------------------
TRINITY_DN18_c0_g1_i3         ATTTAAATTTTGCCAATTGGCCCGCTCCGGTGGAGGAGGACCTAAATAACGAAATTCTCG


TRINITY_DN266_c0_g1_i1        ---------GGCTACTGTCTGCTGCCGCTAAATACGGTGTTGTATCAAAATATGTTCCGC VP spliced 1
TRINITY_DN266_c0_g1_i2        CATACTTACGGCTACTGTCTGCTGCCGCTAAATACGGTGTTGTATCAAAATATGTTCCGC VP unspliced
TRINITY_DN266_c0_g1_i4        CATACTTACGGCTACTGTCTGCTGCCGCTAAATACGGTGTTGTATCAAAATATGTTCCGC VP unspliced
TRINITY_DN266_c0_g1_i3        ---------GGCTACTGTCTGCTGCCGCTAAATACGGTGTTGTATCAAAATATGTTCCGC VP spliced 1
MpDNV_mRNA_seq_consensus      CATACTTACGGCTACTGTCTGCTGCCGCTAAATACGGTGTTGTATCAAAATATGTTCCGC 5'splice site
TRINITY_DN7_c0_g1_i4          CATACTTACGGCTACTGTCTGCTGCCGCTAAATACGGTGTTGTATCAAAATATGTTCCGC VP unspliced
TRINITY_DN7_c0_g1_i2          ---------GGCTACTGTCTGCTGCCGCTAAATACGGTGTTGTATCAAAATATGTTCCGC VP spliced 1
TRINITY_DN7_c0_g1_i1          CATACTTACGGCTACTGTCTGCTGCCGCTAAATACGGTGTTGTATCAAAATATGTTCCGC VP unspliced
TRINITY_DN7_c0_g1_i3          ---------GGCTACTGTCTGCTGCCGCTAAATACGGTGTTGTATCAAAATATGTTCCGC VP spliced 1
TRINITY_DN18_c0_g1_i1         ---------GGCTACTGTCTGCTGCCGCTAAATACGGTGTTGTATCAAAATATGTTCCGC VP spliced 1
TRINITY_DN18_c0_g1_i2         CATACTTACGGCTACTGTCTGCTGCCGCTAAATACGGTGTTGTATCAAAATATGTTCCGC VP unspliced
TRINITY_DN18_c0_g1_i4         ---------GGCTACTGTCTGCTGCCGCTAAATACGGTGTTGTATCAAAATATGTTCCGC VP spliced 1
TRINITY_DN18_c0_g1_i3         CATACTTACGGCTACTGTCTGCTGCCGCTAAATACGGTGTTGTATCAAAATATGTTCCGC VP unspliced
                                       ***************************************************

TRINITY_DN266_c0_g1_i1        TAGTGTTAGGTTTGTTCGGGTATATTGCTGTCTCGCTGCCTGTCAAGAACTGATCGGCTA
TRINITY_DN266_c0_g1_i2        TAGTGTTAGGTTTGTTCGGGTATATTGCTGTCTCGCTGCCTGTCAAGAACTGATCGGCTA
TRINITY_DN266_c0_g1_i4        TAGTGTTAGGTTTGTTCGGGTATATTGCTGTCTCGCTGCCTGTCAAGAACTGATCGGCTA
TRINITY_DN266_c0_g1_i3        TAGTGTTAGGTTTGTTCGGGTATATTGCTGTCTCGCTGCCTGTCAAGAACTGATCGGCTA
MpDNV_mRNA_seq_consensus      TAGTGTTAGGTTTGTTCGGGTATATTGCTGTCTCGCTGCCTGTCAAGAACTGATCGGCTA
TRINITY_DN7_c0_g1_i4          TAGTGTTAGGTTTGTTCGGGTATATTGCTGTCTCGCTGCCTGTCAAGAACTGATCGGCTA
TRINITY_DN7_c0_g1_i2          TAGTGTTAGGTTTGTTCGGGTATATTGCTGTCTCGCTGCCTGTCAAGAACTGATCGGCTA
TRINITY_DN7_c0_g1_i1          TAGTGTTAGGTTTGTTCGGGTATATTGCTGTCTCGCTGCCTGTCAAGAACTGATCGGCTA
TRINITY_DN7_c0_g1_i3          TAGTGTTAGGTTTGTTCGGGTATATTGCTGTCTCGCTGCCTGTCAAGAACTGATCGGCTA
TRINITY_DN18_c0_g1_i1         TAGTGTTAGGTTTGTTCGGGTATATTGCTGTCTCGCTGCCTGTCAAGAACTGATCGGCTA
TRINITY_DN18_c0_g1_i2         TAGTGTTAGGTTTGTTCGGGTATATTGCTGTCTCGCTGCCTGTCAAGAACTGATCGGCTA
TRINITY_DN18_c0_g1_i4         TAGTGTTAGGTTTGTTCGGGTATATTGCTGTCTCGCTGCCTGTCAAGAACTGATCGGCTA
TRINITY_DN18_c0_g1_i3         TAGTGTTAGGTTTGTTCGGGTATATTGCTGTCTCGCTGCCTGTCAAGAACTGATCGGCTA
                              ************************************************************

TRINITY_DN266_c0_g1_i1        AATCGACACCACGCTTTGCGATCAATCCGAGAGCTGTCGCAATGTTGCCATAATATGGGT
```

```
TRINITY_DN266_c0_g1_i2        AATCGACACCACGCTTTGCGATCAATCCGAGAGCTGTCGCAATGTTGCCATAATATGGGT
TRINITY_DN266_c0_g1_i4        AATCGACACCACGCTTTGCGATCAATCCGAGAGCTGTCGCAATGTTGCCATAATATGGGT
TRINITY_DN266_c0_g1_i3        AATCGACACCACGCTTTGCGATCAATCCGAGAGCTGTCGCAATGTTGCCATAATATGGGT
MpDNV_mRNA_seq_consensus      AATCGACACCACGCTTTGCGATCAATCCGAGAGCTGTCGCAATGTTGCCATAATATGGGT
TRINITY_DN7_c0_g1_i4          AATCGACACCACGCTTTGCGATCAATCCGAGAGCTGTCGCAATGTTGCCATAATATGGGT
TRINITY_DN7_c0_g1_i2          AATCGACACCACGCTTTGCGATCAATCCGAGAGCTGTCGCAATGTTGCCATAATATGGGT
TRINITY_DN7_c0_g1_i1          AATCGACACCACGCTTTGCGATCAATCCGAGAGCTGTCGCAATGTTGCCATAATATGGGT
TRINITY_DN7_c0_g1_i3          AATCGACACCACGCTTTGCGATCAATCCGAGAGCTGTCGCAATGTTGCCATAATATGGGT
TRINITY_DN18_c0_g1_i1         AATCGACACCACGCTTTGCGATCAATCCGAGAGCTGTCGCAATGTTGCCATAATATGGGT
TRINITY_DN18_c0_g1_i2         AATCGACACCACGCTTTGCGATCAATCCGAGAGCTGTCGCAATGTTGCCATAATATGGGT
TRINITY_DN18_c0_g1_i4         AATCGACACCACGCTTTGCGATCAATCCGAGAGCTGTCGCAATGTTGCCATAATATGGGT
TRINITY_DN18_c0_g1_i3         AATCGACACCACGCTTTGCGATCAATCCGAGAGCTGTCGCAATGTTGCCATAATATGGGT
                              ************************************************************

TRINITY_DN266_c0_g1_i1        CGCTGACTTGCAGCGCTCCCCGTATCGCTTTTTTGTCTAATTCCCATATGTTATCCTTGC
TRINITY_DN266_c0_g1_i2        CGCTGACTTGCAGCGCTCCCCGTATCGCTTTTTTGTCTAATTCCCATATGTTATCCTTGC
TRINITY_DN266_c0_g1_i4        CGCTGACTTGCAGCGCTCCCCGTATCGCTTTTTTGTCTAATTCCCATATGTTATCCTTGC
TRINITY_DN266_c0_g1_i3        CGCTGACTTGCAGCGCTCCCCGTATCGCTTTTTTGTCTAATTCCCATATGTTATCCTTGC
MpDNV_mRNA_seq_consensus      CGCTGACTTGCAGCGCTCCCCGTATCGCTTTTTTGTCTAATTCCCATATGTTATCCTTGC
TRINITY_DN7_c0_g1_i4          CGCTGACTTGCAGCGCTCCCCGTATCGCTTTTTTGTCTAATTCCCATATGTTATCCTTGC
TRINITY_DN7_c0_g1_i2          CGCTGACTTGCAGCGCTCCCCGTATCGCTTTTTTGTCTAATTCCCATATGTTATCCTTGC
TRINITY_DN7_c0_g1_i1          CGCTGACTTGCAGCGCTCCCCGTATCGCTTTTTTGTCTAATTCCCATATGTTATCCTTGC
TRINITY_DN7_c0_g1_i3          CGCTGACTTGCAGCGCTCCCCGTATCGCTTTTTTGTCTAATTCCCATATGTTATCCTTGC
TRINITY_DN18_c0_g1_i1         CGCTGACTTGCAGCGCTCCCCGTATCGCTTTTTTGTCTAATTCCCATATGTTATCCTTGC
TRINITY_DN18_c0_g1_i2         CGCTGACTTGCAGCGCTCCCCGTATCGCTTTTTTGTCTAATTCCCATATGTTATCCTTGC
TRINITY_DN18_c0_g1_i4         CGCTGACTTGCAGCGCTCCCCGTATCGCTTTTTTGTCTAATTCCCATATGTTATCCTTGC
TRINITY_DN18_c0_g1_i3         CGCTGACTTGCAGCGCTCCCCGTATCGCTTTTTTGTCTAATTCCCATATGTTATCCTTGC
                              ************************************************************

TRINITY_DN266_c0_g1_i1APFV-16 TAACATCCGTAGCATTAAAATAATCGGCATCGTGTTGAAATGCGACCTTATCTATATCAC
TRINITY_DN266_c0_g1_i2        TAACATCCGTAGCATTAAAATAATCGGCATCGTGTTGAAATGCGACCTTATCTATATCAC
TRINITY_DN266_c0_g1_i4        TAACATCCGTAGCATTAAAATAATCGGCATCGTGTTGAAATGCGACCTTATCTATATCAC
TRINITY_DN266_c0_g1_i3        TAACATCCGTAGCATTAAAATAATCGGCATCGTGTTGAAATGCGACCTTATCTATATCAC
MpDNV_mRNA_seq_consensus      TAACATCCGTAGCATTAAAATAATCGGCATCGTGTTGAAATGCGACCTTATCTATATCAC
TRINITY_DN7_c0_g1_i4  APFV-14 TAACATCCGTAGCATTAAAATAATCGGCATCGTGTTGGAATGCGACCTTATCTATATCAC
TRINITY_DN7_c0_g1_i2          TAACATCCGTAGCATTAAAATAATCGGCATCGTGTTGGAATGCGACCTTATCTATATCAC
TRINITY_DN7_c0_g1_i1          TAACATCCGTAGCATTAAAATAATCGGCATCGTGTTGGAATGCGACCTTATCTATATCAC
TRINITY_DN7_c0_g1_i3          TAACATCCGTAGCATTAAAATAATCGGCATCGTGTTGGAATGCGACCTTATCTATATCAC
TRINITY_DN18_c0_g1_i1 APFV-15 TAACATCCGTAGCATTAAAATAATCGGCATCGTGTTGGAATGCGACCTTATCTATATCAC
TRINITY_DN18_c0_g1_i2         TAACATCCGTAGCATTAAAATAATCGGCATCGTGTTGGAATGCGACCTTATCTATATCAC
TRINITY_DN18_c0_g1_i4         TAACATCCGTAGCATTAAAATAATCGGCATCGTGTTGGAATGCGACCTTATCTATATCAC
TRINITY_DN18_c0_g1_i3         TAACATCCGTAGCATTAAAATAATCGGCATCGTGTTGGAATGCGACCTTATCTATATCAC
                              ************************************* **********************

TRINITY_DN266_c0_g1_i1        TAGTAGGTAACGCCTTAGCATTAAAATGACCTTCACTGTCACACAACAGATTACCTGGAC
TRINITY_DN266_c0_g1_i2        TAGTAGGTAACGCCTTAGCATTAAAATGACCTTCACTGTCACACAACAGATTACCTGGAC
TRINITY_DN266_c0_g1_i4        TAGTAGGTAACGCCTTAGCATTAAAATGACCTTCACTGTCACACAACAGATTACCTGGAC
TRINITY_DN266_c0_g1_i3        TAGTAGGTAACGCCTTAGCATTAAAATGACCTTCACTGTCACACAACAGATTACCTGGAC
MpDNV_mRNA_seq_consensus      TAGTAGGTAACGCCTTAGCATTAAAATGACCTTCACTGTCACACAACAGATTACCTGGAC
TRINITY_DN7_c0_g1_i4          TAGTAGGTAACGCCTTAGCATTAAAATGACCTTCACTGTCACACAACAGATTACCTGGAC
TRINITY_DN7_c0_g1_i2          TAGTAGGTAACGCCTTAGCATTAAAATGACCTTCACTGTCACACAACAGATTACCTGGAC
TRINITY_DN7_c0_g1_i1          TAGTAGGTAACGCCTTAGCATTAAAATGACCTTCACTGTCACACAACAGATTACCTGGAC
TRINITY_DN7_c0_g1_i3          TAGTAGGTAACGCCTTAGCATTAAAATGACCTTCACTGTCACACAACAGATTACCTGGAC
TRINITY_DN18_c0_g1_i1         TAGTAGGTAACGCCTTAGCATTAAAATGACCTTCACTGTCACACAACAGATTACCTGGAC
TRINITY_DN18_c0_g1_i2         TAGTAGGTAACGCCTTAGCATTAAAATGACCTTCACTGTCACACAACAGATTACCTGGAC
TRINITY_DN18_c0_g1_i4         TAGTAGGTAACGCCTTAGCATTAAAATGACCTTCACTGTCACACAACAGATTACCTGGAC
TRINITY_DN18_c0_g1_i3         TAGTAGGTAACGCCTTAGCATTAAAATGACCTTCACTGTCACACAACAGATTACCTGGAC
                              ************************************************************

TRINITY_DN266_c0_g1_i1        CCACGAAGCTACTACCGAACCACGTGATACCTTCATTGCTAGTACCTGAAGCACCTAATT
TRINITY_DN266_c0_g1_i2        CCACGAAGCTACTACCGAACCACGTGATACCTTCATTGCTAGTACCTGAAGCACCTAATT
TRINITY_DN266_c0_g1_i4        CCACGAAGCTACTACCGAACCACGTGATACCTTCATTGCTAGTACCTGAAGCACCTAATT
TRINITY_DN266_c0_g1_i3        CCACGAAGCTACTACCGAACCACGTGATACCTTCATTGCTAGTACCTGAAGCACCTAATT
MpDNV_mRNA_seq_consensus      CCACGAAGCTACTACCGAACCACGTGATACCTTCATTGCTAGTACCTGAAGCACCTAATT
TRINITY_DN7_c0_g1_i4          CCACGAAGCTACTACCGAACCACGTGATACCTTCATTGCTAGTACCTGAAGCACCTAATT
TRINITY_DN7_c0_g1_i2          CCACGAAGCTACTACCGAACCACGTGATACCTTCATTGCTAGTACCTGAAGCACCTAATT
TRINITY_DN7_c0_g1_i1          CCACGAAGCTACTACCGAACCACGTGATACCTTCATTGCTAGTACCTGAAGCACCTAATT
TRINITY_DN7_c0_g1_i3          CCACGAAGCTACTACCGAACCACGTGATACCTTCATTGCTAGTACCTGAAGCACCTAATT
TRINITY_DN18_c0_g1_i1         CCACGAAGCTACTACCGAACCACGTGATACCTTCATTGCTAGTACCTGAAGCACCTAATT
TRINITY_DN18_c0_g1_i2         CCACGAAGCTACTACCGAACCACGTGATACCTTCATTGCTAGTACCTGAAGCACCTAATT
TRINITY_DN18_c0_g1_i4         CCACGAAGCTACTACCGAACCACGTGATACCTTCATTGCTAGTACCTGAAGCACCTAATT
TRINITY_DN18_c0_g1_i3         CCACGAAGCTACTACCGAACCACGTGATACCTTCATTGCTAGTACCTGAAGCACCTAATT
                              ************************************************************

TRINITY_DN266_c0_g1_i1        TAATACGTTTTCTCTCGTTATTCTCCTCTTCAACACTATAAAATTCGTCGTTTACCGACG
TRINITY_DN266_c0_g1_i2        TAATACGTTTTCTCTCGTTATTCTCCTCTTCAACACTATAAAATTCGTCGTTTACCGACG
TRINITY_DN266_c0_g1_i4        TAATACGTTTTCTCTCGTTATTCTCCTCTTCAACACTATAAAATTCGTCGTTTACCGACG
TRINITY_DN266_c0_g1_i3        TAATACGTTTTCTCTCGTTATTCTCCTCTTCAACACTATAAAATTCGTCGTTTACCGACG
MpDNV_mRNA_seq_consensus      TAATACGTTTTCTCTCGTTATTCTCCTCTTCAACACTATAAAATTCGTCGTTTACCGACG
TRINITY_DN7_c0_g1_i4          TAATACGTTTTCTCTCGTTATTCTCCTCTTCAACACTATAAAATTCGTCGTTTACCGACG
TRINITY_DN7_c0_g1_i2          TAATACGTTTTCTCTCGTTATTCTCCTCTTCAACACTATAAAATTCGTCGTTTACCGACG
TRINITY_DN7_c0_g1_i1          TAATACGTTTTCTCTCGTTATTCTCCTCTTCAACACTATAAAATTCGTCGTTTACCGACG
TRINITY_DN7_c0_g1_i3          TAATACGTTTTCTCTCGTTATTCTCCTCTTCAACACTATAAAATTCGTCGTTTACCGACG
TRINITY_DN18_c0_g1_i1         TAATACGTTTTCTCTCGTTATTCTCCTCTTCAACACTATAAAATTCGTCGTTTACCGACG
TRINITY_DN18_c0_g1_i2         TAATACGTTTTCTCTCGTTATTCTCCTCTTCAACACTATAAAATTCGTCGTTTACCGACG
TRINITY_DN18_c0_g1_i4         TAATACGTTTTCTCTCGTTATTCTCCTCTTCAACACTATAAAATTCGTCGTTTACCGACG
TRINITY_DN18_c0_g1_i3         TAATACGTTTTCTCTCGTTATTCTCCTCTTCAACACTATAAAATTCGTCGTTTACCGACG
                              ************************************************************
```

```
TRINITY_DN266_c0_g1_i1        ACATGTTAAACGTAACTGATTAAATATAAGTAAAATGTAAACACTTGAGTAAATAGTGAT
TRINITY_DN266_c0_g1_i2        ACATGTTAAACGTAACTGATTAAATATAAGTAAAATGTAAACACTTGAGTAAATAGTGAT
TRINITY_DN266_c0_g1_i4        ACATGTTAAACGTAACTGATTAAATATAAGTAAAATGTAAACACTTGAGTAAATAGTGAT
TRINITY_DN266_c0_g1_i3        ACATGTTAAACGTAACTGATTAAATATAAGTAAAATGTAAACACTTGAGTAAATAGTGAT
MpDNV_mRNA_seq_consensus      ACATGTTAAACGTAACTGATTAAATATAAGTAAAATGTAAACACTTGAGTAAATAGTGAT
TRINITY_DN7_c0_g1_i4          ACATGTTAAACGTAACTGATTAAATATAAGTAAAATGTAAACACTTGAGTAAATAGTGAT
TRINITY_DN7_c0_g1_i2          ACATGTTAAACGTAACTGATTAAATATAAGTAAAATGTAAACACTTGAGTAAATAGTGAT
TRINITY_DN7_c0_g1_i1          ACATGTTAAACGTAACTGATTAAATATAAGTAAAATGTAAACACTTGAGTAAATAGTGAT
TRINITY_DN7_c0_g1_i3          ACATGTTAAACGTAACTGATTAAATATAAGTAAAATGTAAACACTTGAGTAAATAGTGAT
TRINITY_DN18_c0_g1_i1         ACATGTTAAACGTAACTGATTAAATATAAGTAAAATGTAAACACTTGAGTAAATAGTGAT
TRINITY_DN18_c0_g1_i2         ACATGTTAAACGTAACTGATTAAATATAAGTAAAATGTAAACACTTGAGTAAATAGTGAT
TRINITY_DN18_c0_g1_i4         ACATGTTAAACGTAACTGATTAAATATAAGTAAAATGTAAACACTTGAGTAAATAGTGAT
TRINITY_DN18_c0_g1_i3         ACATGTTAAACGTAACTGATTAAATATAAGTAAAATGTAAACACTTGAGTAAATAGTGAT
                              ************************************************************

TRINITY_DN266_c0_g1_i1        GACGTCACCCCCACCTAGTCGCCTACTGGATACTTGTCTATCTTTTTATTATATAAAAAG
TRINITY_DN266_c0_g1_i2        GACGTCACCCCCACCTAGTCGCCTACTGGATACTTGTCTATCTTTTTATTATATAAAAAG
TRINITY_DN266_c0_g1_i4        GACGTCACCCCCACCTAGTCGCCTACTGGATACTTGTCTATCTTTTTATTATATAAAAAG
TRINITY_DN266_c0_g1_i3        GACGTCACCCCCACCTAGTCGCCTACTGGATACTTGTCTATCTTTTTATTATATAAAAAG
MpDNV_mRNA_seq_consensus      GACGTCACCCCCACCTAGTCGCCTACTGGATACTTGTCTATCTTTTTATTATATAAAAAG
TRINITY_DN7_c0_g1_i4  APFV-14 GACGTCACCCCCACCTAGTCGCCTACTGGATACTTGCCTATCTTTTTATTATATAAAAAG 3'-ITR TATA-box
TRINITY_DN7_c0_g1_i2          GACGTCACCCCCACCTAGTCGCCTACTGGATACTTGCCTATCTTTTTATTATATAAAAAG
TRINITY_DN7_c0_g1_i1          GACGTCACCCCCACCTAGTCGCCTACTGGATACTTGCCTATCTTTTTATTATATAAAAAG
TRINITY_DN7_c0_g1_i3          GACGTCACCCCCACCTAGTCGCCTACTGGATACTTGCCTATCTTTTTATTATATAAAAAG
TRINITY_DN18_c0_g1_i1         GACGTCACCCCCACCTAGTCGCCTACTGGATACTTGTCTATCTTTTTATTATATAAAAAG
TRINITY_DN18_c0_g1_i2         GACGTCACCCCCACCTAGTCGCCTACTGGATACTTGTCTATCTTTTTATTATATAAAAAG
TRINITY_DN18_c0_g1_i4         GACGTCACCCCCACCTAGTCGCCTACTGGATACTTGTCTATCTTTTTATTATATAAAAAG
TRINITY_DN18_c0_g1_i3         GACGTCACCCCCACCTAGTCGCCTACTGGATACTTGTCTATCTTTTTATTATATAAAAAG
                              ************************************ ***********************

TRINITY_DN266_c0_g1_i1APFV-16 ATCCCCTACTAATAATAACCACTACCTCCTTCCGGGGGGCGGGGGGTATCCTTTGTTATC VP 5'-end
TRINITY_DN266_c0_g1_i2        ATCCCCTACTAATAATAACCACTACCTCCTTCCGGGGGGCGGGGGGTATCCTTTGTTATC VP 5'-end
TRINITY_DN266_c0_g1_i4        ATCCCCTACTAATAATAACCACTACCTCCTTCCGGGGGGCGGGGGGTATCCTTTGTTATC VP 5'-end
TRINITY_DN266_c0_g1_i3        ATCCCCTACTAATAATAACCACTACCTCCTTCCGGGGGGCGGGGGGTATCCTTTGTTATC VP 5'-end
MpDNV_mRNA_seq_consensus      ATCCCCTACTAATAATAACCACTACCTACTTCCGGGGGGCGGGGGGTATCCTTTGTTATC
TRINITY_DN7_c0_g1_i4  APFV-14 ATCCCCCACTAATAATAACCACTACCTACTTCCGGGGGGCGGGGGGTATCC-TTGTTATC
TRINITY_DN7_c0_g1_i2          ATCCCCCACTAATAATAACCACTACCTACTTCCGGGGGGCGGGGGGTATCC-TTGTTATC
TRINITY_DN7_c0_g1_i1          ATCCCCCACTAATAATAACCACTACCTACTTCCGGGGGGCGGGGGGTATCC-TTGTTATC
TRINITY_DN7_c0_g1_i3          ATCCCCCACTAATAATAACCACTACCTACTTCCGGGGGGCGGGGGGTATCC-TTGTTATC
TRINITY_DN18_c0_g1_i1 APFV-15 ATCCCCCACTAATAATAACCACTACCTACTTCCGGGGGGCGGGGGGTATCCTTTGTTATC
TRINITY_DN18_c0_g1_i2         ATCCCCCACTAATAATAACCACTACCTACTTCCGGGGGGCGGGGGGTATCCTTTGTTATC
TRINITY_DN18_c0_g1_i4         ATCCCCCACTAATAATAACCACTACCTACTTCCGGGGGGCGGGGGGTATCCTTTGTTATC
TRINITY_DN18_c0_g1_i3         ATCCCCCACTAATAATAACCACTACCTACTTCCGGGGGGCGGGGGGTATCCTTTGTTATC
                              ****** ******************** *********************** ********

TRINITY_DN266_c0_g1_i1        ------------------------------------------------------------
TRINITY_DN266_c0_g1_i2        ------------------------------------------------------------
TRINITY_DN266_c0_g1_i4        ------------------------------------------------------------
TRINITY_DN266_c0_g1_i3        ------------------------------------------------------------
MpDNV_mRNA_seq_consensus      TATACTTATTGTCAACACTAGCCTTATCTTAGGGCGTGTCGGCCGACACGTGTCATGGCC
TRINITY_DN7_c0_g1_i4          TATACTTATTGTCAACACTAGCCTTAT--------------------------------- VP 5'-end
TRINITY_DN7_c0_g1_i2          TATACTTATTGTCAACACTAGCCTTAT--------------------------------- VP 5'-end
TRINITY_DN7_c0_g1_i1          TATACTTATTGTCAACACTAGCCTTAT--------------------------------- VP 5'-end
TRINITY_DN7_c0_g1_i3          TATACTTATTGTCAACACTAGCCTTAT--------------------------------- VP 5'-end
TRINITY_DN18_c0_g1_i1         TATACTTATTGTCAACACTAGCC------------------------------------- VP 5'-end
TRINITY_DN18_c0_g1_i2         TATACTTATTGTCAACACTAGCC------------------------------------- VP 5'-end
TRINITY_DN18_c0_g1_i4         TATACTTATTGTCAACACTAGCC------------------------------------- VP 5'-end
TRINITY_DN18_c0_g1_i3         TATACTTATTGTCAACACTAGCC------------------------------------- VP 5'-end
```
